# Identification of polyphosphate-binding proteins in *Escherichia coli* uncovers targets involved in translation control and ribosome biogenesis

**DOI:** 10.1128/mbio.00500-25

**Published:** 2025-07-07

**Authors:** Kanchi Baijal, Brianna Kore, Iryna Abramchuk, Alix Denoncourt, Shauna Han, Abby Simms, Amy Dagenais, Abagail R. Long, Adam D. Rudner, Mathieu Lavallée-Adam, Michael J. Gray, Michael Downey

**Affiliations:** 1 Department of Cellular and Molecular Medicine, University of Ottawa 153010 , Ottawa, Ontario, Canada; 2 University of Ottawa, Ottawa Institute of Systems Biology 175134, Ottawa, Ontario, Canada; 3 Department of Biochemistry, Microbiology and Immunology, University of Ottawa 151173, Ottawa, Ontario, Canada; 4 Department of Microbiology, University of Alabama at Birmingham 318277, Birmingham, Alabama, USA; Yale School of Medicine, New Haven, Connecticut, USA; Philipps University Marburg, Marburg, Germany

**Keywords:** polyphosphate, *E. coli*, polyP-binding proteins, ribosome, stress response, PPK, SrmB, YihI, YjeQ (RsgA), SmpB, Rnr (VacB), InfB, Rne

## Abstract

In many bacteria, polyphosphate kinase (PPK) enzymes use ATP to synthesize polyphosphate (polyP) in response to cellular stress. These chains of inorganic phosphates are joined by high-energy bonds and can reach hundreds of residues in length. PolyP plays diverse functions in helping bacteria adjust to changing environmental conditions. However, the molecular mechanisms underlying these functions are poorly understood. In eukaryotic cells, polyacidic serine- and lysine-rich (PASK) motifs of proteins can mediate binding to polyP chains. Whereas PASK motifs are relatively common in yeast and human cells, we report that these sequences are rare in bacteria commonly used for polyP research. Thus, to identify novel polyP-binding proteins in *Escherichia coli*, we carried out a screen and identified seven novel targets with links to translation control and ribosome biogenesis. For two targets, the GTPase activating protein YihI and the ribonuclease Rnr, we mapped the regions of polyP interaction to non-PASK sequences and identified lysine residues critical for binding. We found that deletion of *rnr* suppressed the slow-growth phenotype of Δ*ppk* mutants grown on minimal media. Conversely, *ppk* deletion resulted in decreased Rnr protein expression. These phenotypes were dependent on the polyP-binding region of Rnr but independent of polyP binding itself, suggesting a complex interplay between PPK and Rnr function in *E. coli*. Overall, our work provides new insights into the scope of polyP-binding proteins and extends the connections between polyP and the regulation of protein translation in *E. coli*.

**IMPORTANCE:**

In bacteria, polyphosphate (polyP) molecules are important regulators of cellular stress responses. Accordingly, cells that cannot make polyP display defects in processes that are important for bacterial survival, infection, and antibiotic resistance. The molecular mechanisms by which polyP exerts its functions are poorly understood. In eukaryotic cells, there has been much interest in the identification and characterization of polyP-binding proteins that act as effectors of polyP *in vivo*. By comparison, much less is known about polyP-binding proteins in bacteria. In this study, we take advantage of large-scale collections of *Escherichia coli* strains expressing epitope-tagged proteins to carry out the first systematic search for bacterial polyP-binding proteins. We describe seven novel polyP-binding proteins with links to ribosome biogenesis or translation. We further identify a complex genetic and molecular interplay between polyphosphate kinase, the enzyme that makes polyP, and the polyP-binding protein RNase R. Given the importance of translational control for bacteria survival, investigation of these pathways is expected to reveal new targets that can be leveraged for therapeutic exploration.

## INTRODUCTION

Polyphosphate (polyP) chains are multifunctional polymers composed of three to hundreds of phosphate monomers linked by high-energy phosphoanhydride bonds (1). Although polyP molecules are found broadly across prokaryotic and eukaryotic cells, the mechanisms of polyP synthesis differ between species (2). In bacteria*,* polyP is synthesized by the polyphosphate kinase (PPK) enzymes, usually in response to cellular stresses such as starvation (3) or treatment with oxidizing agents (4). While some bacteria, such as the gram-negative *Escherichia coli*, have only one PPK enzyme, others express both PPK1 and PPK2 proteins (2). Compared to PPK2, PPK1 is the dominant polyP-synthesizing enzyme in bacteria and preferentially uses ATP as a substrate (5). PPK1 enzymes can also catalyze the reverse reaction to generate ATP from ADP and polyP (6), although the extent to which this activity regulates pools of polyP *in vivo* is uncertain. Alternatively, polyP molecules can be degraded into free inorganic phosphate (Pi) via the action of the exopolyphosphatase PPX, which cleaves phosphoanhydride bonds beginning at the ends of polyP chains (7). Bacterial cells mutated for *ppk* genes show defects in stress and antibiotic resistance (4, 8, 9), reduced biofilm formation (10), and decreased ability to infect host cells (11). These phenotypes underlie efforts to develop PPK inhibitors as a new tool in the fight against antimicrobial resistance. PPK enzymes are also present in some lower eukaryotic organisms, including the slime mold *Dictyostelium discoideum*, having been acquired by horizontal gene transfer (12, 13).

In yeast, polyP chains are synthesized by the vacuole-bound vacuolar transporter chaperone (VTC) complex (14). VTC activity is coupled to polyP transport into the vacuole lumen and its sequestration there (15). The VTC complex (and presumably polyP) has been linked to ion and phosphate homeostasis (16, 17), cell cycle control (18), microautophagy (19), and the regulation of protein translation (20). There are no mammalian homologs of VTC or PPK proteins, and the mechanism of polyP synthesis remains poorly defined in higher eukaryotes such as humans (21, 22). There is one report that the mitochondrial F_o_F1 ATPase can synthesize polyP (23), but it is unclear if this activity impacts the total cellular levels of the polymer. The levels of polyP in human cells are generally thought to be lower than those measured in microorganisms (21), although this assertion has recently been challenged (24). Regardless, diverse roles for polyP have been suggested in mammalian cells including cell signaling (25–27), protein folding (28), energy metabolism (29), and blood clotting (30). While polyP could impact cell function through diverse mechanisms, there is particular interest in roles mediated by its interaction with protein targets (reviewed in reference 31). Previous work in eukaryotes has collectively identified dozens of polyP-binding partners (20, 32–37). In bacteria, however, examples of polyP-interacting proteins are less common. In *E. coli*, during stress, polyP serves as a molecular adaptor for the Lon protease to promote the degradation of ribosomal proteins as well as the DnaA replication initiation protein (38, 39). PolyP binding to CsgA plays a role in the regulation of biofilm formation (28). Finally, polyP also binds to the chaperone Hfq to promote its tight interaction with DNA and regulate its phase separation (40). Beyond *E. coli*, the regulation of stress responses by polyP-binding proteins is a common theme. For example, in *Helicobacter pylori*, polyP binding to sigma 80 is thought to directly regulate a transcriptional program to help bacteria adapt to starvation (41). Since the deletion of *ppk* homologs in many bacteria impacts diverse molecular pathways (10, 40, 42, 43), we speculated that additional polyP-binding proteins remain to be found.

In this work, we report the use of an untargeted proteomic screen to identify seven novel polyP-binding proteins in *E. coli*. Remarkably, all seven of these targets are linked to ribosome biogenesis and protein translation. For two proteins, YihI and Rnr (RNase R), we mapped the region of polyP binding to lysine-rich sequences of the proteins that are important for ribosome- and translation-related functions. Unexpectedly, while Rnr levels are downregulated in Δ*ppk* mutants relative to wild-type controls grown in minimal media, deletion of the *rnr* gene or truncation of the polyP-binding region suppresses the slow-growth phenotype of Δ*ppk* mutants under the same conditions. However, mutational analysis revealed that these effects are likely independent of Rnr binding to polyP, suggesting the possibility that polyP impacts Rnr function through both direct and indirect mechanisms. Together, our work extends the scope of polyP-protein interactions in *E. coli* and identifies new avenues for exploration of the PPK-dependent regulation of ribosome biogenesis and protein translation *in vivo*.

## RESULTS

### The landscape of PASK-containing proteins in bacteria

In eukaryotic cells, we have been particularly interested in the interaction of polyP with polyacidic serine- and lysine-rich (PASK) motifs of target proteins. In *Saccharomyces cerevisiae*, for example, there are 427 PASK-containing proteins, and work from our group and others has validated polyP binding to 27 of these (20, 36, 44). While the interaction between polyP and PASK-containing proteins was originally proposed to be covalent (36), recent work challenges this assertion, suggesting a non-covalent interaction with positively charged PASK lysines instead (34). Regardless of the mechanism at play, we reasoned that PASK-containing proteins would be excellent candidates for novel polyP effectors in bacteria.

**Fig 1:**
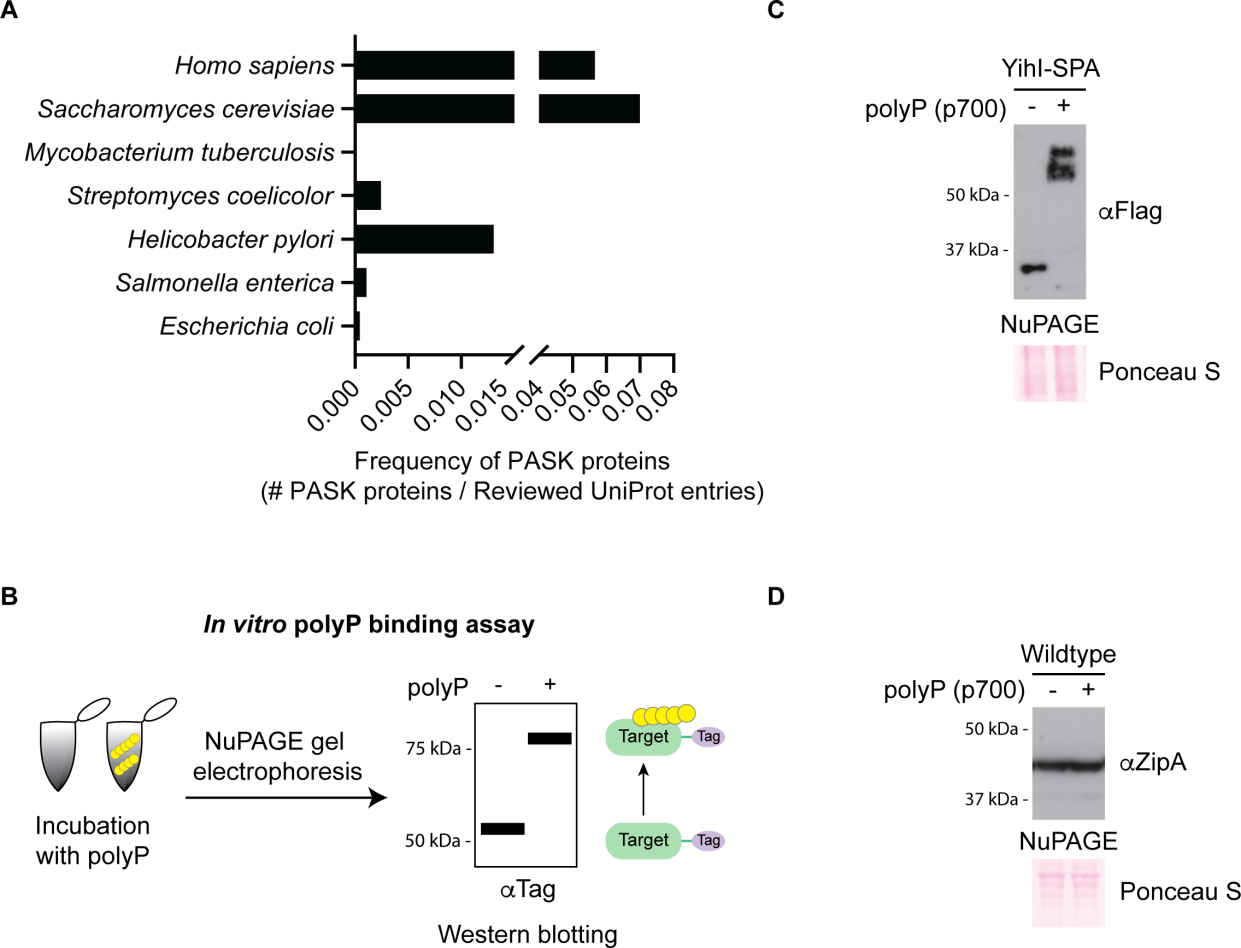
Characterization of PASK sequences in *E. coli*. (**A**) Frequency of PASK motifs in bacteria. The number of proteins containing one or more PASK motifs (75% D/E/S/K content with at least one lysine within a 20 amino acid window) from reviewed proteomes of the indicated species was normalized by the total number of reviewed UniProt entries of each species. Underlying data for panel A can be found in Source Data 1. (**B**) Schematic of the *in vitro* polyP-binding assay. Whole-cell extracts incubated in the absence or presence of synthetic polyP (p700) were resolved using a Bis-Tris gel (sold under the name NuPAGE) electrophoresis. Target proteins were visualized by western blotting using an antibody toward an epitope tag or the endogenous protein. Proteins that have slower migration in the presence of polyP compared to in its absence are thought to bind polyP. (**C and D**) *In vitro* polyP binding to (**C**) YihI-SPA and (**D**) ZipA. Assays were conducted as described in panel B. In both cases, samples were resolved using NuPAGE and transferred to a PVDF membrane. YihI-SPA and ZipA were detected using anti-Flag or anti-ZipA antibodies, respectively. Ponceau S was used to show that samples migrated equally. Images are representative of results from ≥3 experiments.

To investigate the occurrence of PASK motifs in bacteria, we searched the proteomes of both gram-negative and positive species. We did so using a program we call PASKMotifFinder, which was also recently used to find PASK-containing proteins in *Trypanosomes* (45). We defined a PASK motif as a protein subsequence of 20 amino acids containing at least 75% D/E/S/K residues and one lysine residue, consistent with the definition we used previously for eukaryotic cells (20). We found that compared to yeast and human cells, PASK-containing proteins are rare in both reviewed (Fig. 1A) and unreviewed (Fig. S1A) UniProt database entries (46) from proteomes of bacterial species commonly used for polyP research.

### *E. coli* YihI is a novel polyP-binding protein

We focused on the only two PASK-containing proteins in *E. coli*, ZipA and YihI, that were identified using PASKMotifFinder with a D/E/S/K residue threshold of 75% (Table S1). ZipA is an essential protein required for cell division (47), and YihI is an activating protein for the essential GTPase Der (48). Together, these two proteins represent 0.05% of the total proteome—a stark contrast to the situation in *S. cerevisiae*, where the fraction of PASK-containing proteins is 7.3% (20). To determine if ZipA and YihI interact with polyP, we looked for polyP-induced electrophoretic shifts (hereafter termed “polyP shifts”) on bis-tris polyacrylamide gels, which are sold commercially under the NuPAGE brand name (Fig. 1B). This technique has previously been used to characterize polyP binding to PASK motifs (20, 34, 36, 49). To conduct these *in vitro* polyP-binding assays, we incubated whole-cell extracts from SPA-tagged and wild-type strains with polyP of 700 units in length (p700). The YihI-SPA fusion protein was detected using an anti-Flag antibody. In contrast, for ZipA detection, we used a previously published antibody (50) that we first validated in Fig. S1B. In this assay, YihI-SPA, but not ZipA, demonstrated the characteristic polyP shift indicative of polyP binding (Fig. 1C and D), and this effect was dependent on the concentration of polyP used (Fig. S1C).

### Characterization of the YihI PASK-like motif

**Fig 2:**
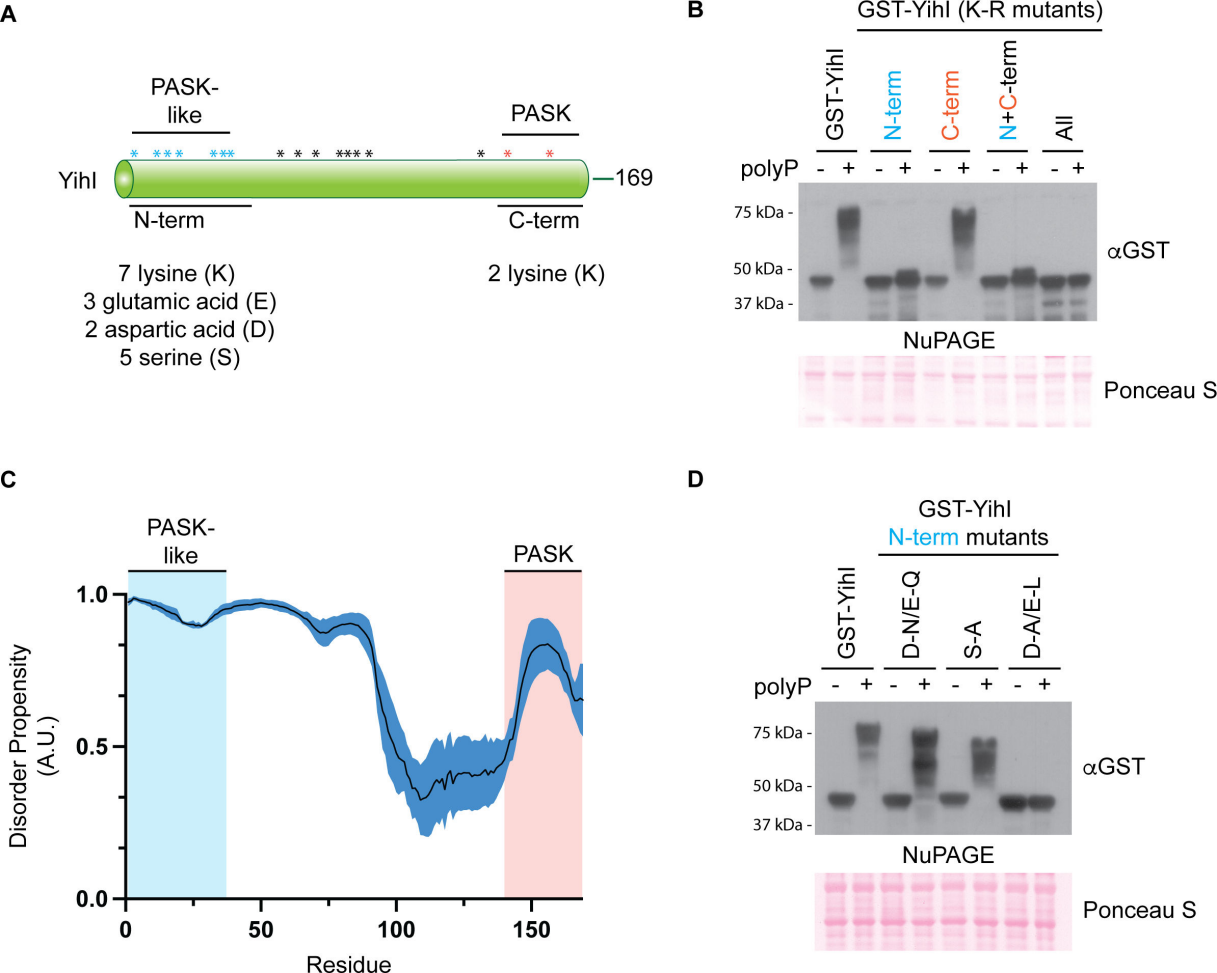
PolyP binds a disordered lysine-rich region of YihI. (**A**) Schematic and amino acid distribution of full-length YihI (169 residues total). YihI has a C-terminal PASK motif and an N-terminal PASK-like region. The indicated amino acids distributed across the PASK or PASK-like sequences were targeted for mutagenesis experiments. An asterisk (*) is used to display the distribution of PASK (orange), PASK-like (blue), and other (black) lysine residues within YihI. (**B**) PolyP binds primarily via the N-terminus of YihI. *In vitro* polyP-binding assay was conducted (as described in Fig. 1B) using whole-cell extract expressing wild-type or **K-R** mutated GST-YihI. (**C**) The disorder propensity of YihI shows that the N- and C-termini are unstructured (>0.5). The graph shows the average (±SE) of computational prediction scores, represented as arbitrary units (A.U.), that were obtained using NetSurfP-3.0 (54), Metapredict (55), and IUPred3 (56). Underlying data for panel C can be found in Source Data 3. (**D**) The N-terminal PASK amino acids play a structural role in promoting polyP binding. Various GST-YihI mutants were grown and analyzed as described in panel **B**. D-N/E-Q = aspartic acid to asparagine/glutamic acid to glutamine; S-A = serine to alanine; D-A/E-L = aspartic acid to alanine/glutamic acid to leucine. For both panels **B** and **D**, samples were resolved using NuPAGE, transferred to a PVDF membrane, and probed using an anti-GST antibody. Ponceau S was used to show that samples migrated equally. Images are representative of results from ≥3 experiments.

Next, we aimed to further investigate how the YihI PASK was contributing to polyP binding. Previous work showed that mutation of lysine residues to arginine (K-R) abolished the polyP shift on NuPAGE gels for other PASK-containing proteins (20, 36, 51, 52). Therefore, we used GST-YihI fusion proteins to test if this held true for YihI. Our bioinformatics analysis located the PASK motif to the C-terminus of the YihI protein (Fig. 2A). Surprisingly, mutation of the two lysine residues in this region failed to prevent polyP interaction, suggesting that YihI does not bind to polyP via its defined PASK motif (Fig. 2B). We noticed that the N-terminus of YihI is also lysine rich (Fig. 2A). This region was only identified as a hit using the PASKMotifFinder program when the D/E/S/K ratio threshold was lowered to 50% (Table S1). Mutation of seven N-terminal lysines to arginine severely abrogated the polyP shift (Fig. 2B). Therefore, we conclude that YihI interacts with polyP primarily through this region. A YihI mutant where all lysines were replaced with arginine residues completely lost its ability to bind polyP as judged by NuPAGE analysis (Fig. 2B), suggesting that other lysines may also contribute to polyP binding, at least in the absence of those in the N-terminus. While the N-terminus does not fit the formal definition of a PASK motif, it does contain a number of serine (5) and acidic (5) residues in addition to seven lysine residues required for polyP interaction (Fig. 2A). Therefore, we refer to this region as “PASK-like.” Notably, both the N- and C-termini of YihI are disordered (Fig. 2C), which is fitting for the molecular chaperone and scaffold-like functions of polyP (4, 53). Additionally, the N-terminus of YihI may play regulatory functions. For example, truncation mutants lacking residues 1–45 show enhanced binding to Der and activation of Der’s GTP hydrolysis activity (48), suggesting an overall inhibitory role for the N-terminus of YihI. We speculate that polyP binding may regulate these functions *in vivo*.

Previous mutagenesis work on yeast targets demonstrated that serine (S) residues in PASK motifs are not required for polyP binding (36). In contrast, mutation of acidic residues (aspartic and glutamic acid) to alanine or leucine (D-A/E-L) prevented polyP interaction (49). In both cases, analogous N-terminal mutations resulted in a similar impact on polyP binding to GST-YihI (Fig. 2D). To test if polyP binding depends on the negative charge of these acidic residues, we also mutated aspartic and glutamic acids to asparagine and glutamine (D-N/E-Q), respectively. With these changes, GST-YihI was still able to bind to polyP (Fig. 2D), suggesting that negative charge *per se* is not required for polyP interaction, at least for this “PASK-like” region of YihI (see “Discussion”).

### Novel non-PASK polyP-binding proteins in *E. coli*

To extend our search for polyP-binding proteins in bacteria, we took advantage of two sets of *E. coli* strains where individual open-reading frames are expressed as fusion proteins with C-terminal SPA (781 strains) or TAP (243 strains) epitope tags (Table S2) (57). We generated protein extracts from these strains and carried out *in vitro* polyP-binding assays, as described above (Fig. 1B). The SPA tag (58) contains a 3Flag epitope, and the TAP tag has a protein A moiety that is recognized by most mouse antibodies. Therefore, we used a mouse anti-Flag antibody to detect both SPA- and TAP-tagged targets after NuPAGE gel electrophoresis and western blotting.

**Fig 3:**
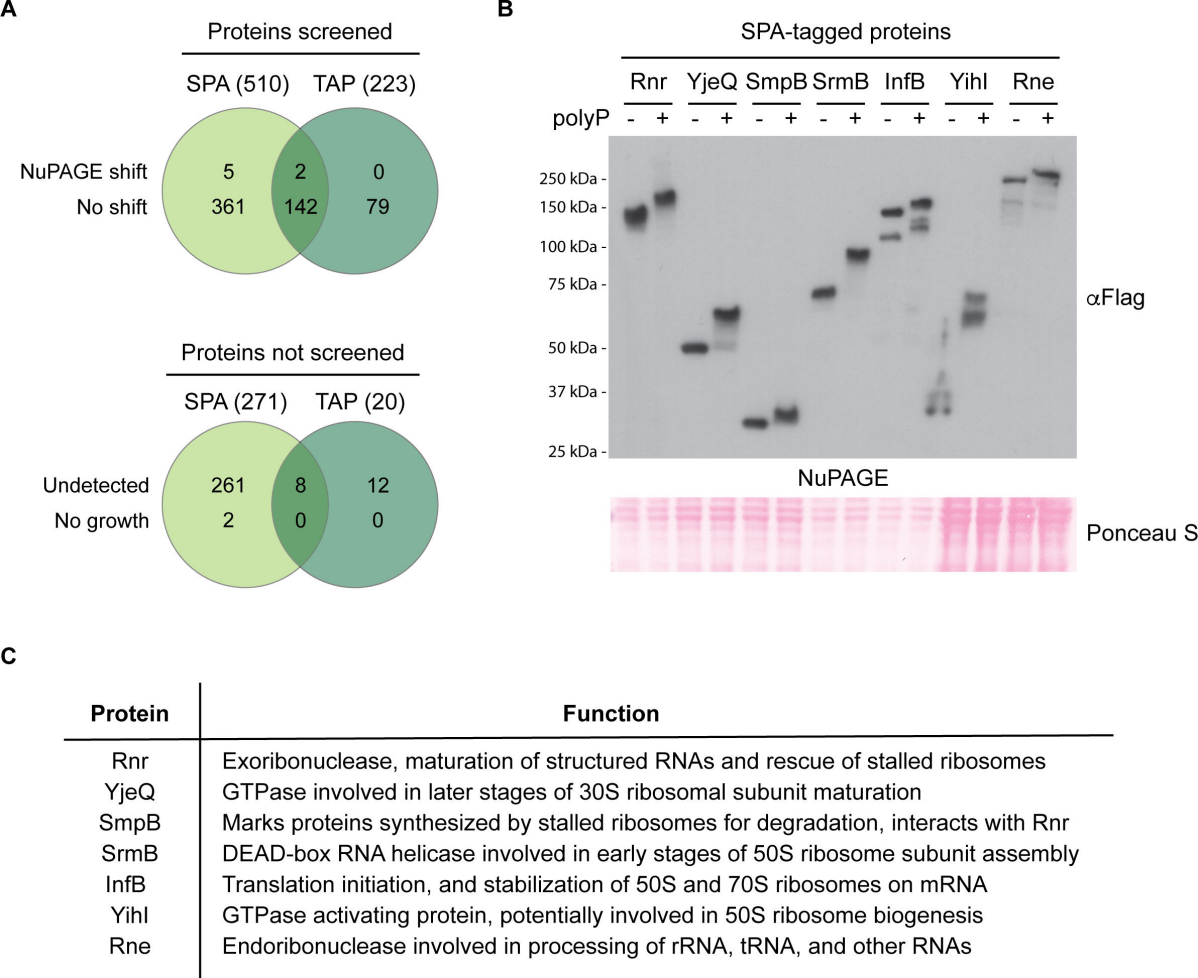
A screen for novel polyP-binding proteins in *E. coli*. (**A**) A total of 589 unique *E. coli* proteins were screened for polyP binding. Together, the SPA and TAP collection sets contain a total of 1,024 strains with C-terminal epitope tags encoded at the chromosomal loci of relevant open reading frames. Of these, 152 proteins are redundantly tagged between the SPA and TAP collection sets, and 291 (283 non-redundant) could not be screened for polyP binding because they could not be detected by western blotting, or the strain did not grow. (**B**) Seven novel polyP-binding proteins were identified by the screen. Proteins that shifted from the screen were reconfirmed using the *in vitro* polyP-binding assay. Samples were resolved using NuPAGE, transferred to a PVDF membrane, and probed using an anti-Flag antibody which detects the SPA-tag. Ponceau S was used to show that samples migrated equally. Images are representative of results from ≥3 experiments. (**C**) The seven polyP-binding proteins are involved in ribosome biogenesis or translation processes. General descriptions of each protein’s functions are provided.

After accounting for redundancy between the two epitope-tagged sets and proteins that were not detected by western blotting, we evaluated polyP binding for a total of 589 unique proteins using this assay (Fig. 3A; Table S2). Seven of these (1.2% of total proteins screened) shifted on NuPAGE gels in the presence of polyP (Fig. 3B). With 4,288 predicted open reading frames in *E. coli*, we anticipate that at least ~50 proteins from *E. coli* would undergo a polyP shift in this assay. This value is likely an underestimate of the total number of polyP-binding proteins in *E. coli*, as work with human polyP interactors demonstrated that not all undergo polyP shifts on NuPAGE gels (32). Indeed, we observed that the Lon protease, a well-characterized polyP-binding protein from *E. coli* (38, 39), does not shift on NuPAGE gels even in the presence of high concentrations of polyP (Fig. S2A). Likewise, polyP failed to cause a shift on NuPAGE gels for SPA-tagged DnaA and Hfq (Fig. S2B), which are also reported to bind polyP (39, 40). We suggest that NuPAGE analysis may discriminate proteins that interact with polyP in a denatured or unfolded state (see “Discussion”).

Intriguingly, all seven polyP binders identified in our work have links to ribosome assembly or function (Fig. 3C). This finding is consistent with the enrichment of this same category in our yeast polyP-PASK interaction study (20), as well as the remodeling of nucleoli, the site of ribosome biogenesis, in human cells ectopically expressing bacterial PPK to produce high levels of polyP (59). Altogether, this suggests the possibility of evolutionarily conserved roles for polyP in the regulation of translation.

### Interaction of target proteins with endogenous polyP

To test if native polyP was also able to bind our newly identified targets, we switched SPA-tagged strains grown in lysogeny broth (LB) media to MOPS minimal media to induce nutrient starvation and polyP accumulation prior to protein extraction and NuPAGE analysis. Out of the seven targets, SrmB-SPA and YihI-SPA consistently displayed an obvious MOPS-induced polyP shift, while Rnr-SPA did so occasionally (Fig. S3A). This result is perhaps surprising considering that the chain lengths of polyP that accumulate during MOPS appear to be larger than the p700 chains used in our *in vitro* assays (Fig. S3B). We speculate that long-chain bacterial polyP is organized *in vivo* in a manner that in some instances hinders its interaction with protein targets. In support of this idea, we found that a large fraction of polyP that accumulates during MOPS treatment is resistant to ectopically expressed yeast Ppx1 (*Sc*Ppx1), a highly active exopolyphosphatase (Fig. S3C). This finding is reminiscent of the situation in mammalian cell culture where *Sc*Ppx1 treatment results in a partial but not complete loss of the polyP signal in nuclear polyP foci detected using the PPBD-Xpress tag probe (60). In contrast, *Sc*Ppx1 overexpression in yeast appears to completely degrade the non-vacuolar pool of polyP synthesized by *E. coli* PPK expression (61).

### Identification of functional interactions between polyP-binding proteins and PPK

**Fig 4:**
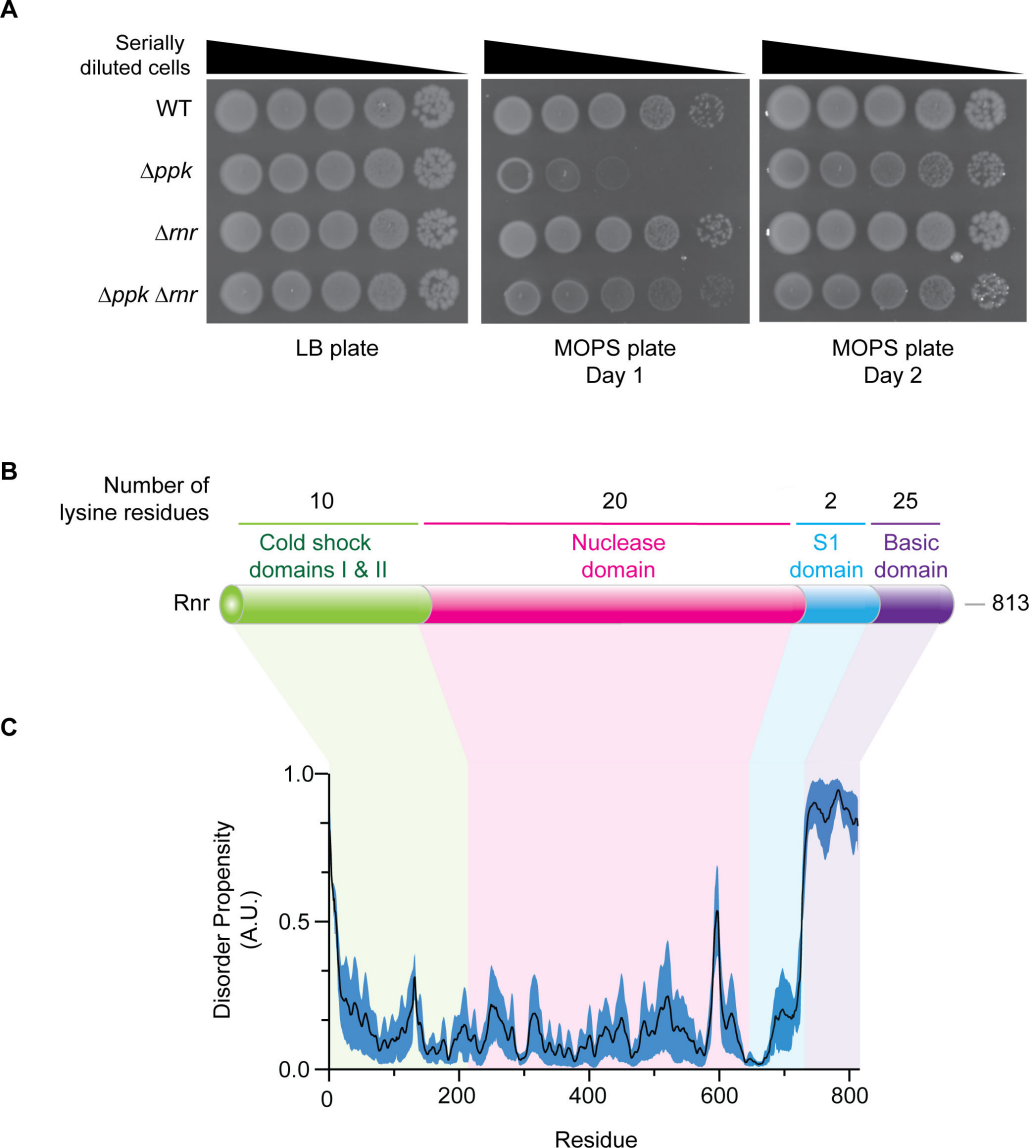
Rnr is functionally regulated by polyP. (**A**) Loss of *rnr* partially rescues the slow-growth phenotype of *ppk* mutants. The indicated strains were serially diluted and spotted onto LB or MOPS plates and incubated at 37°C as indicated. Images are representative of results from ≥3 experiments. (**B**) Schematic of the functional domains of full-length Rnr (813 residues total). Rnr has two cold shock domains (residues 1–216), a nuclease domain (residues 217–643), an S1 domain (residues 644–730), and a basic domain (residues 731–813). (**C**) The basic domain of Rnr has a high disorder propensity (>0.5). The graph shows the average (±SE) of computational prediction scores, represented as arbitrary units (A.U.), that were obtained using NetSurfP-3.0 (54), Metapredict (55), and IUPred3 (56). Underlying data for panel C can be found in Source Data 3.

We reasoned that some genes encoding polyP-binding proteins could influence the growth of ∆*ppk* mutants under conditions where polyP is important for cell growth or viability (Fig. S4A). As previously reported and consistent with work from other groups (3, 62), we found that Δ*ppk* mutants displayed a slow-growth phenotype on MOPS minimal media relative to wild-type controls (Fig. 4A; Fig. S4A). This phenotype is likely attributable to an extended lag phase and the increased doubling time in ∆*ppk* mutant cells (42). Consistently, we observed that deletion of *rnr* improved the growth of ∆*ppk* mutant cells on MOPS media (Fig. 4A; Fig. S4A) without impacting polyP levels in wild-type cells (Fig. S4B). While the rescue was not complete, we conclude that in ∆*ppk* mutants, one or more activities of Rnr hinder cell growth during nutrient limitation. Therefore, we were interested in investigating if this role was related to Rnr’s ability to bind polyP.

Rnr (also referred to as VacB in literature) is a 3′–5′ exoribonuclease that plays a role in maintaining RNA homeostasis in cells (63, 64). It primarily targets rRNAs and structured RNAs, including RNA duplexes, but not DNA (63, 65–67). Rnr has a complex role *in vivo*. It is thought to play a role in RNA turnover and the recycling of excess rRNA during stress, such as starvation, cold shock, and entry into the stationary phase (68–70). It has also been proposed to participate in trans-translation through its role in the maturation of tmRNA (71), which binds SmpB (another polyP-binding protein identified by our screen) (72) and is required for tagging abnormal peptides and releasing stalled ribosomes (73–75). Additionally, in an SmpB-dependent manner (76), Rnr degrades “non-stop” transcripts that result in ribosome stalling (75, 77).

These complex functions and interactions of Rnr are mediated by various domains that work together in a coordinated manner. For example, Rnr possesses two cold shock domains with helicase activity (78), cold shock-specific functions (78), and a role in substrate binding (65), as well as a catalytic core termed the ribonuclease domain where reduction reactions take place (65, 79) (Fig. 4B). It also possesses S1 and basic domains that are involved in protein stabilization (80), substrate positioning (65), and ribosome binding (81) (Fig. 4B). Intriguingly, the basic domain of Rnr is both disordered (Fig. 4C) and lysine rich, hinting at a possible role in polyP binding.

### The S1 + basic domain of Rnr binds polyP

**Fig 5:**
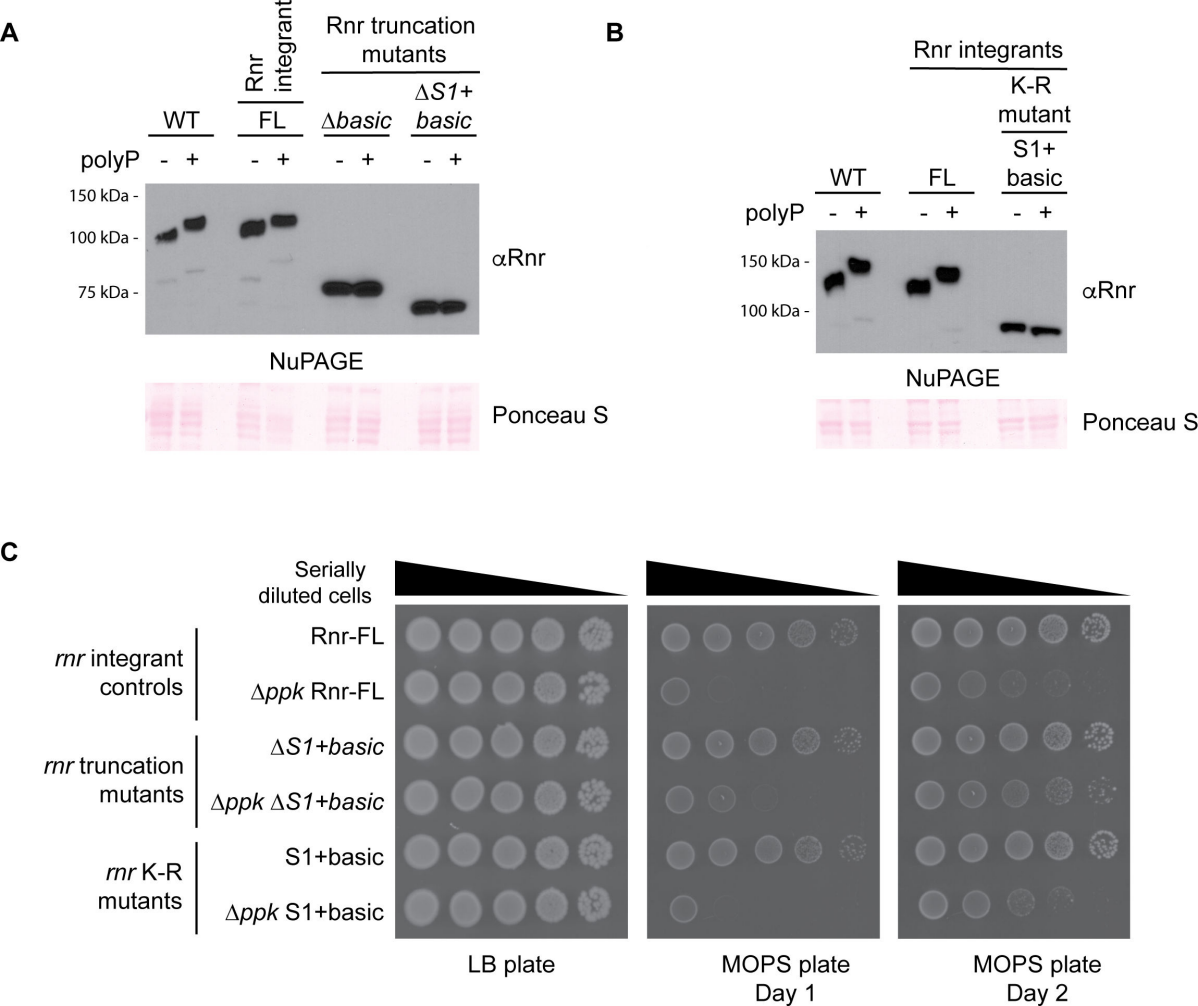
The Rnr S1 and basic domains are involved in polyP binding. (**A and B**) The characteristic NuPAGE shift is abrogated when the S1 and basic domains of Rnr are (**A**) truncated or (**B**) mutated. An *in vitro* polyP-binding assay was conducted using the indicated chromosomally truncated or **K-R** mutated Rnr strains. Full length (FL) represents the wild-type Rnr protein expressed in a background that is isogenic to the truncated and mutated strains (see “*Bacterial strains*” section of the “*Materials and Methods*” for details on how these strains were constructed). Samples were resolved using NuPAGE, transferred to a PVDF membrane, and probed using an anti-Rnr antibody. Ponceau S was used to show that samples migrated equally. Images are representative of results from ≥3 experiments. (**C**) Truncation but not K-R mutation of the S1 + basic polyP-binding domain partially rescues the slow-growth phenotype of *ppk* mutants. The indicated strains were serially diluted and spotted onto LB or MOPS plates and incubated at 37°C as indicated. Images are representative of results from ≥3 experiments.

To map the region of Rnr required for interaction with polyP, we expressed its individual domains as GST-fusion proteins and carried out *in vitro* polyP-binding assays as described for YihI. These experiments demonstrated that the C-terminus of the protein (S1 + basic domain) was responsible for polyP binding (Fig. S5A). Indeed, deletion of this region from chromosomally expressed Rnr (detected using an anti-Rnr antibody, validated in Fig. S5B) resulted in a loss of the polyP shift (Fig. 5A), as did mutation of 27 S1 + basic lysines (Fig. 5B). Since NuPAGE assays determine protein-polyP interactions under largely denaturing conditions, we also tested if polyP interacts with Rnr in its folded state. To do this, Rnr-3Flag was immunoprecipitated under non-denaturing conditions and incubated with polyP prior to washing and elution with sample buffer. In this experiment, unbound polyP is expected to be removed prior to NuPAGE analysis (Fig. S5C). Immunoprecipitated Rnr incubated with polyP shifted on NuPAGE gels after washing, suggesting that polyP can also bind to Rnr when folded (Fig. S5C).

In growth assays, deletion of the Rnr S1 + basic domain, but not the basic domain on its own, improved the growth of ∆*ppk* mutants on MOPS (Fig. 5C; Fig. S5D), suggesting that together, these regions mediate toxicity in MOPS media in the absence of polyP. If polyP binding to the S1 + basic domain functions to promote the growth of wild-type cells on MOPS media, we predicted that under these conditions, wild-type cells expressing the K-R mutant should display a slow-growth phenotype, similar to ∆*ppk* mutants. However, the K-R mutant grew similarly to wild-type cells on MOPS media (Fig. 5C). The simplest model, therefore, suggests that Rnr’s ability to bind polyP is not related to the genetic interaction between ∆*rnr* and ∆*ppk* that is described above.

### Complex regulation of Rnr by PPK and polyP

**Fig 6:**
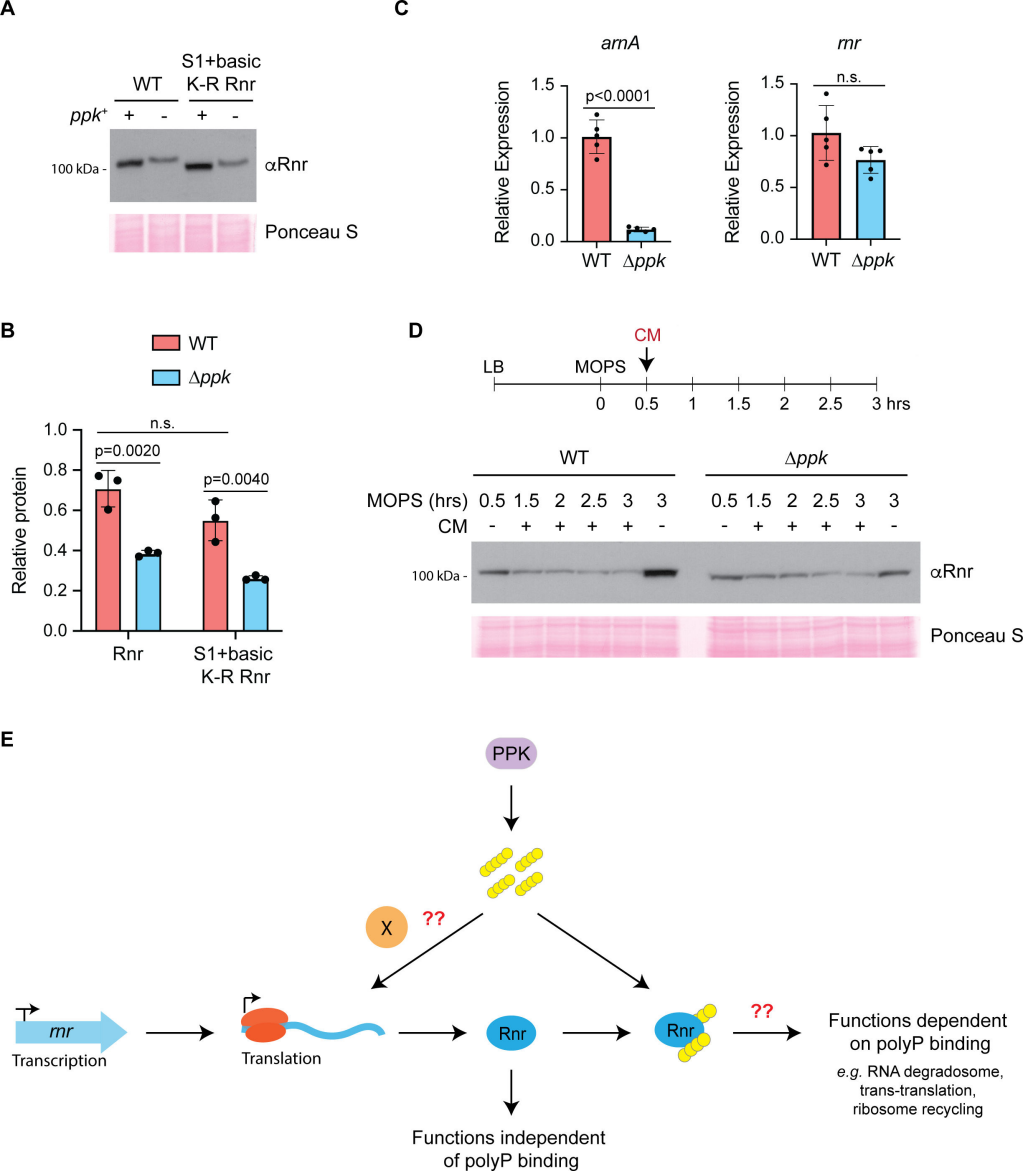
PPK regulates Rnr expression at the translational level. (**A and B**) Western blot and quantification of WT and K-R mutated Rnr levels. Cells grown in LB were nutrient downshifted to MOPS media for 3 hours. Samples were resolved using a 4%–20% Criterion TGX Stain-Free gel, transferred to a PVDF membrane, and probed using an anti-Rnr antibody. Ponceau S was used to show equal loading. Three replicates of each condition were used for quantification as described in the “*Materials and Methods*” section. Mean values with SD are shown. Indicated are the *P*-values and nonsignificant differences (n.s.), calculated using two-way ANOVA with Tukey’s post hoc analysis. Underlying data for panel B can be found in Source Data 4. Western blot images are representative of results from ≥3 experiments. (**C**) Reverse transcriptase quantitative PCR measurements of Rnr transcript levels in WT and ∆*ppk* mutants. Cells were grown as described for panel A and B. Transcript levels were normalized to *yqfB,* and *arnA* served as a PPK-dependent positive control. Mean values with SD are shown. Indicated are the *P*-values and nonsignificant differences (n.s.), calculated using a two-tailed unpaired *t*-test with Welch’s correction. Underlying data for panel C can be found in Source Data 5. (**D**) Rnr turnover in WT and ∆*ppk* mutants during growth in MOPS media. Cells grown in LB and shifted to MOPS media for 30 minutes were treated with chloramphenicol (CM) to stop translation. Untreated controls were used to evaluate Rnr expression in the absence of translation shutoff. Cells were harvested at the indicated time points. Samples were resolved using 10% SDS-PAGE, transferred to a PVDF membrane, and probed using an anti-Rnr antibody. Images are representative of results from ≥3 experiments. (**E**) Model of how polyP regulates Rnr function. PolyP produced by PPK regulates translation but not transcription of Rnr through an unknown mechanism. Additionally, direct binding of polyP may regulate functions of Rnr that have yet to be investigated.

To continue the search for a function of Rnr’s polyP binding, we next investigated if the interaction could impact Rnr expression. In the exponential phase, Rnr is rapidly degraded as a result of acetylation at lysine 544 (K544) (82). This degradation is thought to be mediated through an interaction with SmpB, via the Rnr C-terminus, which results in the recruitment of HslUV and Lon proteases (80, 83). In contrast, Rnr is stabilized during the stationary phase and under stress conditions (84), and its activity increases upon carbon, nitrogen, and phosphorus starvation (69). Therefore, since protein binding to polyP has been shown to modulate protein degradation (39), we evaluated Rnr expression in wild-type and ∆*ppk* mutants during nutrient starvation in MOPS media, where polyP levels in wild-type cells are normally high. We found that Rnr levels were reproducibly decreased in ∆*ppk* mutants (Fig. 6A and B; Fig. S6). However, like the genetic interactions described above, this effect was not directly mediated by polyP binding to Rnr, because truncation and K-R mutants that failed to interact with polyP (Fig. 5A and B) still showed decreased expression in the ∆*ppk* mutant background (Fig. 6A and B; Fig. S6). This raises the question, what is the source of decreased Rnr protein levels in ∆*ppk* mutants? Analysis of *rnr* transcript levels did not reveal a significant difference in expression between wild-type and ∆*ppk* mutants (Fig. 6C). As such, we focused on the possibility that Rnr expression was regulated post-transcriptionally. To test this, we treated cells with chloramphenicol to stop translation after a shift to MOPS media and monitored Rnr levels over time. From these experiments, we were able to make two conclusions. First, between wild-type and ∆*ppk* mutants, there was little difference in the stability of Rnr synthesized before chloramphenicol treatment (Fig. 6D). Second, wild-type Rnr levels failed to increase to the levels observed in the absence of chloramphenicol, where translation is allowed to proceed (Fig. 6D). Therefore, we conclude that PPK most likely plays an indirect role in regulating Rnr expression via the regulation of protein translation. Also, while polyP can interact directly with Rnr, the function of this binding event remains a question for future investigations (Fig. 6E).

## DISCUSSION

In this work, we have identified seven novel polyP-binding proteins in *E. coli* and provided additional evidence to support an evolutionarily conserved role for polyP in the regulation of protein translation.

Previous work in eukaryotic cells has demonstrated that PASK motifs, often found in predicted disordered regions, are a frequent site for polyP binding. To our surprise, PASK-containing proteins are rare in bacterial models commonly used for polyP research. Of the seven proteins we identified as polyP binders, only YihI has a PASK motif that fits our previous definitions of 75% D/E/S/K with at least one lysine in a 20 amino acid window. However, this motif makes at most a minor contribution to YihI’s polyP-binding activity. Instead, we mapped this function to the N-terminus of the protein, which we refer to as PASK-like. Similar to what was described for PASK motifs, mutation of YihI’s N-terminal lysine residues to arginine abolished polyP interaction as judged by the NuPAGE shift assay, whereas mutation of serines to alanines had little impact. Since arginine holds a greater positive charge than lysine, these experiments demonstrate that the concentration of positive charge is not the sole determinant of polyP binding. On the other hand, mutation of the acidic residues, glutamic and aspartic acid to leucine or alanine, abolished polyP interaction. However, mutation of these same residues to uncharged glutamine and asparagine had no effect. Our interpretation of these data is that the negative charge of the PASK-like region is dispensable for polyP interaction. Instead, one possibility is that the glutamic and aspartic acid residues provide a structural context for polyP binding with surrounding lysine residues, and this unique context is preserved with the glutamine and asparagine substitutions. We surmise that the impact of these substitutions will also hold true for canonical PASK motifs in proteins such as yeast Nsr1 and Top1 (36), but this remains to be tested. Indeed, it is currently unclear if there is a tangible difference between PASK and PASK-like motifs in the way that they interact with polyP molecules. When the D/E/S/K ratio threshold was lowered to 60% and 50%, the PASKMotifFinder program uncovered three and seven out of the seven hits identified by our screen, respectively. However, the significance of this observation is not clear given the identification of a large number of proteins (e.g., 872 at the 50% threshold; Table S1). In addition to PASK motifs, polyP has also been shown to bind polyHistidine (35) and polyLysine stretches (34). While long stretches of histidine or lysine were not found in our targets, we cannot discount the possibility that other linear polyP interacting motifs exist, and it will therefore be important to systematically map the binding region for each target.

A limitation of our work is that our detection of polyP-protein interactions relied on the previously described NuPAGE “polyP shift” assay. Not all polyP-binding proteins shift upon NuPAGE analysis in the presence of polyP, as demonstrated with our experiments using purified Lon protease and SPA-tagged Hfq and DnaA. As such, we have no doubt that additional polyP-binding proteins in *E. coli* remain to be identified. In particular, polyP interactions that require folded protein structures would be missed in our assay, as these would likely be denatured during NuPAGE analysis. On the other hand, the ability of targets identified here to interact with polyP under denaturing conditions suggests that polyP may play a role in their folding or re-folding after cellular stress. Indeed, this chaperone-like activity for polyP has been described previously for the *E. coli* CsgA protein involved in biofilm production (28) and globally to stabilize proteins that become insoluble in response to oxidative stress (4).

Our work adds to a growing body of evidence for an evolutionarily conserved role for polyP in regulating protein translation. McInerney et al. found that ∆*ppk* mutant strains have disrupted polysome profiles (85). Furthermore, polyP promotes translation fidelity *in vitro*, and Δ*ppk* mutants have increased mistranslation *in vivo* (85). In this regard, it is noteworthy that all of our newly identified polyP-binding proteins are linked in some way to ribosome biogenesis or translational control. We speculate that polyP binding to these proteins may therefore play a role in reprogramming translation during stress, and this should be a priority for future analysis. Alternatively, polyP may help to stabilize critical regulators of translation so that they are ready to act upon a return to favorable growth conditions. Most of our new targets are highly conserved across other bacterial species (Table S3) and, in some cases, are expressed in pathogens (86–90). While most lysines within the polyP-binding region of YihI are conserved, this is not the case for Rnr (Fig. S7A and B). Therefore, it will be important to test whether polyP interacts with their homologs via these motifs. Moreover, it may be the case that the overall number of lysines, rather than their exact position, is a critical determinant for polyP binding.

Two recent studies complement our finding that polyP binds to proteins involved in translation. First, Guan et al. published an exciting preprint where polyP was found to organize “HP-bodies” in *E. coli* (91). These HP-bodies modulate RNA stability during stress and appear to co-localize with components of the RNA degradosome, which includes Rne (uncovered in our analysis) (91). Our other hits SmpB and InfB are also associated with these bodies (91). Second, very recent work also identifies Rnr and SrmB as polyP-binding proteins in *Pseudomonas aeruginosa*, and SrmB interaction with polyP is proposed to modulate virulence (92).

In addition to identifying novel polyP-binding proteins linked to translation, we found evidence for a bidirectional regulation between PPK and Rnr. Namely, while PPK promotes Rnr expression during starvation, Rnr is also detrimental to growth in ∆*ppk* mutant cells grown on MOPS media. We propose a model where Rnr’s various molecular functions must be carefully balanced during cellular stress, and this balance is lost in the absence of PPK (Fig. 6E). Importantly, mutation of the S1 + basic domain lysine residues of Rnr to prevent polyP binding did not impact Rnr protein levels or growth characteristics in an otherwise wild-type background. The simplest explanation for these observations is that the described bidirectional regulation is indirect in that it does not depend on the Rnr-polyP interaction. Alternatively, the regulation may become relevant under situations where growth is already compromised, as is the case in Δ*ppk* mutant cells.

Since polyP binds to Rnr in both its denatured and folded states, it is possible that polyP impacts Rnr biology at multiple levels, and additional work will be required to tease out specific molecular functions. For example, based on the C-terminal binding of polyP, it may disrupt functions associated with the S1 and basic domains, which includes an intrinsically disordered segment. As observed for Nsr1 and Top1 in yeast (36), polyP binding may disrupt the interaction between tmRNA-SmpB and Rnr, which is mediated via the basic domain (80). This, in turn, could play a role in stabilizing Rnr in some contexts, potentially through cross regulation with a previously reported acetylation at K544 that is known to promote Rnr turnover (82). Additionally, polyP interaction with the S1 domain could alter Rnr substrate selectivity (67). Very likely, polyP binding to Rnr is part of a broader function for polyP in adapting to cellular stress. We note, for example, that Rnr also plays a role in the RNA degradosome in conjunction with Rne (93), another polyP-binding protein identified in our screen. As such, we do not discount the possibility that dramatic phenotypes would only be observed after mutating polyP-binding motifs on multiple proteins involved in the processes of ribosome biogenesis or translation.

*In vivo*, the local subcellular distribution of polyP may govern whether a protein interacts with it. Moreover, we demonstrate here that a large fraction of intracellular polyP is resistant to degradation via overexpression of the highly active yeast Ppx1 (*Sc*Ppx1). As such, some polyP may be inaccessible to potential protein interactors *in vivo*. It is tempting to speculate that this property is dictated by the ability of polyP to phase separate *in vivo*, and the investigation of this property and its relationship to protein-polyP interactions is deserving of further attention. Another important area for future investigation will be to determine how polyP-protein interactions are reversed upon return to normal growth conditions. We speculate that bacterial PPX enzymes may play a critical role in this process. Indeed, this activity has been demonstrated previously for yeast Ppx1 (36). Alternatively, in the presence of ADP, PPK itself may drive the conversion of protein-bound polyP to ATP. This would relieve polyP-dependent modulation of translation while providing ATP pools required for renewed efforts toward ribosome biogenesis and growth.

## MATERIALS AND METHODS

### General information about strains and plasmids

All bacterial strains and plasmids, as well as their sources, used in this work are listed in Table S4. Plasmids were sequenced using Sanger sequencing (Genome Quebec) or Nanopore sequencing (Plasmidsaurus). All plasmids (Table S4) generated for this work will be made available from Addgene (https://www.addgene.org/) upon final publication. The sequences of oligonucleotides used for cloning or genetic manipulations are available upon request.

### Bacterial strains

Unless otherwise indicated, the MG1655 strain background was used for all experiments. All lab-generated strains and PCR-confirmed strains from the SPA-collection set used in this study are listed in Table S4. The Dharmacon Collection of SPA- and TAP-tagged *E. coli* strains (DY330 background) was obtained from Horizon Discovery and has been described previously (57). The strains list for both of these collection sets can be found online under the *Resources* tab (https://horizondiscovery.com/en/non-mammalian-research-tools/products/e-coli-tagged-orfs#description).

Chromosomally tagged and deletion strains were generated using the lambda-red homologous recombination system using pKD46 (induced with 0.2% arabinose) (94) or pSIM6 (induced with a temperature shift to 42°C for 12 minutes) (95). Respectively, the kanamycin deletion and 3Flag-kanamycin tagging cassettes were amplified from pKD4 (94) and pSUB11 (96) plasmids. Rnr truncations were made by using forward primers that introduced a premature stop codon and led to recombination that deleted the end of the gene, replacing the region with the KanR selection cassette. For the basic and S1 + basic mutants, a stop codon was introduced after residue 2,190 and 1,929, respectively. An FRT scar was also introduced at the end of full-length (FL) Rnr to control for polar effects (97). This strain is referred to as FL and is isogenic to the *rnr* truncation mutants (ΔS1 + basic and Δbasic). For genetic experiments, cells were made electrocompetent, and plasmids or double-stranded DNA used for recombineering were transformed into cells via electroporation (98). Antibiotics were added when appropriate: kanamycin (50 µg/mL) and ampicillin (100 µg/mL). As needed, the resistance markers used for the selection of positive transformants were removed using the pCP20 FLP-recombinase system (99). Epitope tag insertions and deletions were confirmed by PCR, followed by western blotting.

### Plasmids

The GST-YihI wild-type and mutant sequence plasmids were purchased from GenScript. The respective YihI sequences were cloned between the EcoRI and NotI sites. These vectors are called: pYihI, pYihI-N-term K-R, pYihI-C-term K-R, pYihI-N+C-term K-R, pYihI-All K-R, pYihI-N-term D-N/E-Q, pYihI-N-term S-A, and pYihI-N-term D-A/E-L.

The Rnr cold shock domains I and II (residues 1–216), nuclease domain (residues 217–643), and S1 + basic domains (residues 644–813) were cloned into pGEX4T1 between the EcoRI and NotI restriction sites using Gibson Assembly Cloning. These vectors are called pRnr-CSD, pRnr-ND, and pRnr-S1BD, respectively.

The *ScPPX1* plasmid was constructed by amplifying the *S. cerevisiae PPX1* sequence from pET-15b-His-*PPX1* and cloning it between EcoRI and SalI sites of pBAD18. The cloned vector is called p*ScPPX1*.

### Bacterial growth conditions

#### General growth conditions

SPA- and TAP-tagged strains (57) (DY330 background) were grown at 30°C, while all other strains in the MG1655 background were grown at 37°C. Unless otherwise specified, strains were grown in LB media.

#### Nutrient downshift

Starvation experiments were performed as previously described (42). Briefly, overnight cultures grown in LB were diluted to 0.1 OD_600_ in LB media and grown to the mid-exponential phase (~0.6 OD_600_) before being switched to MOPS minimal media. Cells were pelleted and washed once with 1× PBS to remove trace LB before resuspension in freshly prepared MOPS media (1× MOPS—Teknova, 0.1 mM K_2_HPO_4_, and 0.4% glucose). Cells were grown in MOPS media for the indicated amount of time. Typically, we see peak polyP accumulation after 3 hours in MOPS media. For western blotting and polyP extractions, 3 and 5 OD_600_ equivalent of cells were harvested by centrifugation, respectively.

#### Chloramphenicol chase assay

To examine Rnr protein turnover, cells were grown in LB media to mid-exponential phase and then shifted to MOPS media as described above. After 30 minutes in MOPS media at 37°C, 300 µg/mL chloramphenicol was added to the culture to halt translation. Cells (3 OD_600_ equivalent) were harvested at the indicated time points for western blotting. In parallel, cells harvested from an untreated culture were used to examine expression profiles in the absence of translation shut-off.

### PASKMotifFinder software

The PASKMotifFinder software used to search for PASK motifs was implemented in Java and is platform independent. The code is open source and freely available at the following GitHub repository: https://github.com/LavalleeAdamLab/PASKMotifFinder/. The software uses a sliding window approach to scan subsequences of 20 amino acids throughout the proteome and identifies regions where D, E, S, and/or K amino acids make up at least 75% of the window (i.e., 15 amino acids) and contain at least one K. The program was run on the *E. coli* (strain K12) proteome UP000000625 and other proteomes listed in Source Data 1. All proteomes were downloaded on 13 January 2025 from UniProt release version 2024_06. Source Data 2 presents the *E. coli* PASK-containing proteins and their motifs, identified using 75%, 60%, and 50% D/E/S/K residue thresholds.

### *In vitro* polyP-binding assay

*In vitro* assays were conducted using whole-cell extract or purified proteins.

Whole-cell extract was prepared as described under “*Western blotting: protein extraction*” using 200 µL of overnight culture. For the polyP-binding assay, 10 µL of whole-cell extract was incubated at room temperature in the presence of 10 mM sodium phosphate pH 6.0 (control matching the pH of the polyP) or p700 (Kerafast) polyP for 20 minutes. For the concentration shift assay, control reactions contained sodium phosphate matching the highest concentration of polyP used. All control (minus polyP) and reaction samples were boiled for 10 minutes and loaded onto a NuPAGE Bis-Tris Mini Protein Gel, 4%–12%, 1.5 mm. See “Materials and Methods” on “*Western blotting*” for the subsequent steps used for visualizing proteins.

Purified Rts1 and Lon protease (purchased from SinoBio) were used for the *in vitro* polyP-binding assay, as described previously (20). Briefly, the purified proteins (0.032 mg of each) were incubated at room temperature with increasing concentrations of p700 (5, 10, 15, and 20 mM) or 20 mM sodium phosphate pH 6.0 (negative control) for 20 minutes. All control (minus polyP) and reaction samples were boiled for 10 minutes and loaded onto a NuPAGE Bis-Tris Mini Protein Gel, 4%–12%, 1.5 mm. The gel was stained using the Invitrogen Colloidal Blue Staining Kit following the manufacturer’s protocols.

### Western blotting

#### Protein extraction

As indicated, 200 µL of an overnight culture or 1.5–3 OD_600_ equivalent of cells was harvested by centrifugation for analysis by western blotting. Cells were resuspended in 100 µL of sample buffer (800 µL sample buffer stock [160 mM Tris-HCl pH 6.8, 30% glycerol, 6% SDS, and 0.004% bromophenol blue] + 100 µL 1 M dithiothreitol (DTT), and 100 µL 1.5 M Tris-HCl pH 8.8), boiled for 10 minutes, and then centrifuged at 13,000 rpm for 2 minutes to remove insoluble material. The supernatant was transferred to a fresh tube. To normalize for equal loading, 10 µL and 13 µL of extract from wild-type and Δ*ppk* mutant cells were loaded per blot, respectively.

#### Gel electrophoresis and transfer

NuPAGE or SDS-PAGE gels were used to resolve protein extracts. SDS-PAGE was primarily used to visualize protein levels, while NuPAGE gels were solely used to detect polyP-dependent shifts of our candidate proteins. After electrophoretic separation, proteins were transferred onto PVDF membranes and visualized by western blotting using the indicated antibodies. SDS-PAGE and NuPAGE buffer recipes have been described previously (20).

#### Western blotting

Membranes were blocked for 20 minutes with shaking using 5% milk in TBST and washed three times for 10 minutes after both primary and secondary antibody incubations. See Table S4 for incubation conditions for each antibody. Both SPA- and TAP-tags were detected using an anti-Flag antibody, which was then detected using a goat anti-mouse secondary coupled to HRP. After probing, target proteins were detected using Immobilon Western Chemiluminescent HRP Substrate and exposure to autoradiography film from Thomas Scientific. Scanned images were opened in Photoshop, and linear brightness and contrast adjustments were made to lighten the image background. Adjustments were applied evenly across the entire image prior to cropping and labeling. For all western blots, staining with Ponceau S was used to verify equal loading, protein migration, and even transfer across the PVDF membrane.

#### Quantification

Rnr expression was evaluated by running three biological replicates of each condition on the same gel and quantifying signal intensities using Image Lab Software 6.1. Using the software, the entire lane was highlighted on the scanned western blot (gray scale), and Rnr-specific bands were manually indicated. For each biological replicate, the expression of Rnr was normalized to the ponceau (gray scale), which was also quantified by highlighting the entire lane. Statistical analysis was carried out with GraphPad Prism version 9.1.2. A two-way ANOVA was used with Tukey’s post hoc analysis. See Source Data 4 for underlying data.

### Mapping polyP-binding domains

YihI (wild-type and mutated sequences) and Rnr domains were cloned into pGEX4T1 and transformed into BL21 for expression. Overnight cultures harboring the plasmids were diluted 1/100 in LB + ampicillin and grown to the mid-exponential phase (~2 hours). Cells were induced with 0.1 mM IPTG for 2 hours and 1.5 OD_600_ equivalent of cells were harvested. Whole-cell extract was prepared by resuspending pellets in 100 µL of sample buffer and was used to conduct *in vitro* polyP-binding assays (described above).

### YihI and Rnr disorder predictions

The amino acid sequences for YihI (UniProt accession: B1XAM2) and Rnr (UniProt accession: P21499) were entered into the following disorder prediction programs: NetSurfP-3.0 (https://services.healthtech.dtu.dk/services/NetSurfP-3.0/) (54), Metapredict online (v3.0) (https://metapredict.net/) (55), and IUPred3 (https://iupred3.elte.hu/) (56). These programs were selected to account for variations between prediction algorithms and prevent bias (100, 101). Disorder scores from the three programs were averaged and graphed with the SE envelope using GraphPad Prism as described by Pastic et al. (102). See Source Data 3 for individual prediction scores.

### Screen for polyP-binding proteins

#### Bacterial growth

SPA and TAP collection sets (57) were pinned onto LB + kanamycin plates and grown overnight at 30°C. The next day, grown colonies were inoculated into 3 mL LB + kanamycin and grown at 30°C overnight.

#### Protein extraction

From the overnight cultures, 200 µL of cells were pelleted and used to prepare whole-cell extract as described in the “*Western blotting*” section of the “Materials and Methods.”

#### *In vitro* polyP-binding assay

The extracts were screened as described above. In brief, whole-cell extract of the tagged strains was incubated in the absence or presence of polyP (modal size p700) and resolved using NuPAGE (as described in the “*Western blotting*” section of the “Materials and Methods”).

#### Confirming positive hits

Positive candidates (along with Hfq-SPA and DnaA-SPA) were streaked for single colonies, and the correct position of the tag was confirmed by PCR. Primers used in these confirmation assays are available upon request. These proteins were re-screened using sodium phosphate pH 6.0 (matching the pH of p700) as a control. With the exception of the screen, sodium phosphate pH 6.0 was used as a control for all *in vitro* polyP-binding assays. All screened strains are listed in Table S2**.**

### Ppx1 overexpression assay

Strains harboring the pBAD18 (empty vector) and *ScPPX1* plasmids were grown in the presence of ampicillin at all stages. Overnight cultures were diluted into LB media and induced with 0.5% arabinose, grown to mid-exponential phase, and then nutrient downshifted into MOPS media (as described above under “*Growth conditions*”) for 3 hours. The only variation is that for the MOPS media, 0.5% arabinose was included, and glucose (0.4%) was replaced with glycerol (0.5%) as the carbon source. For western blotting and polyP extraction, 3 and 5 OD_600_ equivalents of cells were harvested, respectively.

### PolyP extraction

#### Extraction

PolyP extractions were performed as described previously (42) and have been briefly summarized with similar wording here. Five OD_600_ equivalents of cells were used for polyP extractions. Cell pellets were resuspended in 100 mM LiCl, 10 mM EDTA, 10 mM Tris-HCl pH 7.4, and 0.2% SDS. PolyP was extracted using the phenol/chloroform method and precipitated overnight at −20°C in 100% ethanol containing 120 mM sodium acetate. Precipitated polyP was pelleted by centrifugation, resuspended in 30 µL sterile water, and stored at −80°C.

#### Gel analysis

Extracted polyP, mixed 1:1 with loading dye (10 mM Tris-HCl [pH 7], 1 mM EDTA, 30% glycerol, and bromophenol blue) was resolved using a 15.8% TBE-urea gel (5.25 g urea, 7.9 mL 30% acrylamide, 3 mL 5× TBE, 150 µL 10% APS, and 15 µL TEMED) run at 100 V for 1 hour and 45 minutes in 1× TBE. The gel was then stained in a fixing solution (25% methanol and 5% glycerol) containing 0.05% toluidine blue and then de-stained in a fixing solution without toluidine blue. For the polyP standards, 6 µL of each chain length at the specified concentration, p130 (1.25 mM) and p700 (1 mM), was mixed 1:1 with loading dye, and 10 µL was loaded into the gel.

### Growth assays

Spot tests were conducted as described previously, with the details reiterated here (42). The indicated strains were streaked on LB plates and incubated overnight at 37°C. The next day, single colonies were resuspended in 100 µL of sterile water and serially diluted 10-fold five times. Next, 5 µL of each dilution was spotted onto LB or MOPS (1× MOPS, 0.4% glucose, and 0.1 mM K_2_HPO_4_) plates and incubated at 37°C. To prepare MOPS plates, 10× MOPS, glucose, and K_2_HPO_4_ were added after autoclaving water + agar. LB plates were imaged after one overnight, while MOPS plates were imaged post-day 1 and -day 2. Spot tests were imaged using ImageQuant LAS 4000 and edited across the entire image by making minor linear brightness and contrast adjustments in Photoshop to lighten the background.

### Immunoprecipitation and *in vitro* polyP-binding assay

Overnight culture of the Rnr-3Flag tagged strain was diluted to 0.1 OD_600_ in 300 mL of LB and grown to the mid-exponential phase. Fifty OD_600_ equivalent cells were harvested on ice and stored at −80°C until used for immunoprecipitation. Cells were resuspended in 700 µL buffer A (50 mM HEPES pH 7.9, 150 mM NaCl, 1 mM EDTA, 0.5% Triton X-100, 5% glycerol, 1 mM PMSF, and Roche cOmplete protease inhibitor cocktail tablet) and sonicated (Misonix 3000) on ice for three cycles of 10 seconds at power level 3 with 30-second rest in between. The lysate was cleared by centrifugation for 15 minutes at 15,000 rpm at 4°C and then incubated with 5 µL of a 50% anti-FLAG M2 magnetic bead slurry (Sigma Aldrich M8823-1ML) for 1 hour at 4°C. Next, the beads and bound proteins were washed three times with 1 mL of buffer A using cut pipette tips. Beads were then resuspended in 10 mM sodium phosphate (pH 6.0) or p700 in a final volume of 250 µL of buffer B (same as buffer A, but with 0.05% Triton X-100 and no protease inhibitor tablet) and incubated at room temperature with end-to-end rotation for 20 minutes. Excess polyP was then washed away using three 1 mL washes with buffer B. Finally, the bead mixture was transferred to a new tube before eluting proteins in 60 µL 2× sample buffer containing no DTT by incubating at 65°C for 10 minutes. This transfer step was important to remove polyP that remained bound to the tube. Finally, the eluted sample was transferred to a new tube, DTT was added to a final concentration of 100 mM, and the sample was boiled at 100°C for 10 minutes prior to resolving (20 µL per sample) on NuPAGE gels.

### Chromosomal Rnr lysine to arginine mutants

Gene fragments encoding lysine to arginine mutants for the S1 + basic domain were purchased from Twist Bioscience. Toward the 5′ and 3′ ends, the fragments had homology needed for the recombineering transformation and homology toward the beginning of the pKD4 cassette, respectively. In a separate PCR reaction, the KanR cassette was amplified from pKD4. This reaction used forward and reverse primers introducing homology toward the K-R fragments and homology needed for the recombineering transformation, respectively. Next, in a two-step PCR, the two fragments (K-R gene fragments + KanR cassette) were combined at a 1:1 molar ratio and amplified. The final products were gel extracted and transformed into *rnr*-∆basic mutants by electroporation. Correct integration of the K-R mutations was confirmed by Premium PCR sequencing from Plasmidsaurus.

### Anti-GST antibody purification

The anti-GST antibody was purified from sera collected from rabbits injected with a GST-Cdc26 fusion protein (103). Prior to anti-Cdc26 antibody purification on a Cdc26 affinity column, the sera was cleared of anti-GST antibodies on a 50 mL GST affinity column, as described (103). Anti-GST antibodies were eluted with 100 mM glycine, pH 2.1, neutralized in 2 M Tris-base, and dialyzed in antibody storage buffer (1× PBS, 500 mM NaCl, and 50% glycerol).

### Anti-Rnr antibody

An antibody toward Rnr was raised by immunizing New Zealand NZW female rabbits with the purified GST-Rnr nuclease domain (amino acids 649–1,929). This domain was chosen for immunization as it does not bind polyP and therefore would not impact the detection of truncated or mutant Rnr. The pGEX4T1 vector was cloned with a sequence encoding the Rnr nuclease domain (amino acids 649–1,929) using standard Gibson assembly. The oligonucleotides used for this strategy are available upon request. The vector was transformed into BL21 DE3 pLysS *E. coli* and plated on LB + ampicillin + chloramphenicol. Overnight cultures of cells harboring the vector were diluted to 0.1 OD_600_ and grown at 30°C until they reached an OD_600_ of 0.4–0.6. Cells were then induced with 0.25 mM IPTG for 4 hours prior to harvesting and freezing at −80°C in 40 mL of freezing buffer (1× PBS).

#### GST-fusion purification

Frozen cell lysates were thawed in a water bath, and then immediately transferred onto ice to prevent degradation. Next, 40 mL of 1× PBS containing 2 mM EDTA, 2 mM EGTA, 2 mM PMSF, 30 mM DTT, and 1 M NaCl was added to bring the final volume of the cell slurry to 80 mL. Lysozyme was added at a final concentration of 200 µg/mL, and the lysate was incubated on ice for 30 minutes before disruption using the Misonix sonicator (three cycles of 1 minute at power level 7, with 2 minutes rest on ice in between). Triton X-100 was added after sonication to a final concentration of 0.5%. The lysate was centrifuged at 40,000 × *g* for 30 minutes, then cleared through a 0.45-micron filter, and then batch bound for 2 hours to glutathione agarose beads that had been equilibrated in wash buffer with detergent (1× PBS, 0.1% NP-40, 0.5 M NaCl, 1 mM DTT, 1 mM EDTA, 1 mM EGTA, and 1 mM PMSF). The GST-bound beads were washed with 15-column volumes of wash buffer with detergent and 5-column volumes with wash buffer without detergent (no NP-40). The column was eluted with 20 mL elution buffer (50 mM Tris-HCl pH 8.0, 0.5 M NaCl, 10 mM glutathione, 1 mM DTT, and 1 mM PMSF) and dialyzed into the storage buffer (1× PBS, 100 mM NaCl, and 15% glycerol). The dialysis buffer was changed three times. The purified protein was concentrated using an Amicon Ultra-15 centrifugal filter and quantified against serially diluted BSA standards using gel electrophoresis and Coomassie staining and quantification. The final protein was aliquoted and stored at −80°C.

#### Injection preparation

For the first immunization, 675 µL of purified protein, at a final concentration of 2 mg/mL, was combined with 75 µL penicillin (10 U/mL)/streptomycin (10 µg/mL) and 750 µL Freund’s Complete Adjuvant. In contrast, Freund’s Incomplete Adjuvant was used to prepare subsequent booster injections.

#### Injections

For both primary and booster immunizations, four 100 µL subcutaneous and two 250 µL intramuscular injections were administered. The booster was administered 4 weeks after the initial immunization, and the rabbits were terminally bled 4 weeks later. All antigen injections and blood collections were administered under general anesthesia to minimize pain and suffering. Rabbits were first sedated with injectable sedatives butorphanol and midazolam and induced into general anesthesia with inhaled isoflurane from a precision vaporizer.

#### Antibody validation

Sera was validated in the lab by western blotting, using wild-type and Δ*rnr* (negative control) *E. coli* whole-cell extracts (Fig. S5B).

### Reverse transcriptase quantitative PCR

Reverse transcriptase quantitative PCR analysis was conducted as previously described (42) and briefly reiterated here. Five biological replicates of each strain were grown using the nutrient downshift method described under “*Bacterial growth conditions.*” Cells were harvested from 1 mL of culture and resuspended in RNA*later* solution (Invitrogen) for short-term storage at 4°C. RNA*later* was removed prior to RNA extraction using the Thermo Scientific GeneJET RNA Purification Kit (K0731). Extracted RNA (10 µg) was DNase treated following the manufacturer’s instructions (Invitrogen Ambion DNase I [RNase-free]), and DNase was removed using phenol-chloroform extraction. At each step, RNA was quantified by NanoDrop. Next, 1 µg of RNA was additionally DNase treated and reverse transcribed using the All-in-One 5× RT MasterMix (Applied Biological Materials) using the following cycle: 37°C 15 minutes, 25°C 10 minutes, 60°C 20 minutes, and 85°C 5 minutes. Following the RT reaction, cDNA was diluted 1/5, and working aliquots were stored at −80°C. Quantitative PCR reactions were conducted using iQ Syber Green Supermix, in technical replicates of three and in a final volume of 10 µL, under the following conditions: 95°C for 3 minutes and 39 cycles of 95°C for 15 seconds, 63°C for 30 seconds, and 72°C for 30 seconds. Standard curves were created using serially diluted genomic DNA to assess primer efficiency (see Table S4 for primer information). As previously described (42), *yqfB* was used as a normalization control. Gene expression changes were calculated using the ΔΔC_T_ method using Bio-Rad CFX Maestro 2.3 version 5.3.002.1030 software. For *yqfB* and *arnA* expression, one technical replicate from biological replicate #2 was omitted due to the technical error from the machine. Statistical analysis on plotted Log2[FC] values was carried out with GraphPad Prism version 9.1.2 using a two-tailed unpaired *t*-test with Welch’s correction (See Source Data 5 for plotted and Log2[FC] values).

### Sequence similarity comparison analyses

Protein sequences of the seven hits were individually blasted against the *Salmonella enterica* (taxid: 28,901), *Helicobacter pylori* (taxid: 210), *Streptomyces coelicolor* (taxid: 1,902), and *Mycobacterium tuberculosis* (taxid: 1,773) proteomes using NCBI Blast (https://blast.ncbi.nlm.nih.gov/Blast.cgi?PAGE=Proteins). Search settings were as follows: standard databases, non-redundant protein sequences (nr), and blastp (protein-protein BLAST). For each comparison, a single result with the greatest query coverage and, second, lowest *E*-value is presented in Table S3.

### Evolutionary conservation analysis with ConSurf

The evolutionary conservation of amino acid residues in YihI and Rnr was assessed using ConSurf (104). Homologous sequences were identified using the CSI-BLAST (105) algorithm against the UniRef90 protein database (106) (*E*-value cutoff of 0.0001). To ensure phylogenetic diversity while minimizing redundancy, 150 homologs were sampled at even intervals from the resulting hits, which were sorted by *E*-value. A maximum sequence identity of 95% was used to filter out redundant sequences, and a minimal sequence identity of 35% with the query was used to filter out weak hits. Homolog hits were aligned using the MAFFT-L-INS-i (107, 108) algorithm. The rate of evolution at each residue was calculated using the Rate4Site (67) Bayesian method, with the best-fitting substitution model selected automatically. See Source Data 6 for plotted ConSurf scores.

## SUPPLEMENTAL MATERIAL

Source Data S2 mbio.00500-25-s0001.pdf PASK analysis using DESK ratios of 0.75, 0.6, and 0.5.

Supplemental Figures mbio.00500-25-s0002.pdf Figures S1–S7.

Source Data mbio.00500-25-s0003.xlsx Source Data S1 and S3–S6.

Supplemental Tables mbio.00500-25-s0004.xlsx Tables S1–S4.

ASM does not own the copyrights to Supplemental Material that may be linked to, or accessed through, an article. The authors have granted ASM a non-exclusive, world-wide license to publish the Supplemental Material files. Please contact the corresponding author directly for reuse.
