# Supplementary material for: Identification of polyphosphate-binding proteins in Escherichia coli uncovers targets involved in translation control and ribosome biogenesis
Source: mBio. 2025 Jul 7;16(8):e00500-25. doi: 10.1128/mbio.00500-25 (PMC12345225; doi:10.1128/mbio.00500-25)
Supplement: Source Data S2 — PASK analysis using DESK ratios of 0.75, 0.6, and 0.5. [file mbio.00500-25-s0001.pdf]

SD2: PASK analysis using DESK ratio of 0.5 (50%), 0.6 (60%) and 0.75 (75%).

PASK RATIO 0.75

```
sp|P0A8H6|YIHI_ECOLI Der GTPase-activating protein YihI OS=Escherichia
coli (strain K12) OX=83333 GN=yihI PE=1 SV=1

0     MKPSSSNSRSKGHAKARRKTREELDQEARDRKRQKKRRGHAPGSRAAGGN

50    TTSGSKGQNAPKDPRIGSKTPIPLGVTEKVTKQHKPKSEKPMLSPQAELE
                                                 *********
100   LLETDERLDALLERLEAGETLSAEEQSWVDAKLDRIDELMQKLGLSYDDD
      ***********
150   EEEEEDEKQEDMMRLLRGN

sp|P77173|ZIPA_ECOLI Cell division protein ZipA OS=Escherichia coli
(strain K12) OX=83333 GN=zipA PE=1 SV=3
                                                 *********
0     MMQDLRLILIIVGAIAIIALLVHGFWTSRKERSSMFRDRPLKRMKSKRDD
      ***********
50    DSYDEDVEDDEGVGEVRVHRVNHAPANAQEHEAARPSPQHQYQPPYASAQ

100   PRQPVQQPPEAQVPPQHAPHPAQPVQQPAYQPQPEQPLQQPVSPQVAPAP

150   QPVHSAPQPAQQAFQPAEPVAAPQPEPVAEPAPVMDKPKRKEAVIIMNVA

200   AHHGSELNGELLLNSIQQAGFIFGDMNIYHRHLSPDGSGPALFSLANMVK

250   PGTFDPEMKDFTTPGVTIFMQVPSYGDELQNFKLMLQSAQHIADEVGGVV

300   LDDQRRMMTPQKLREYQDIIREVKDANA
```

PASK RATIO 0.6

sp|P76072|STFR_ECOLI Prophage side tail fiber protein homolog StfR OS=Escherichia coli (strain K12) OX=83333 GN=stfR PE=3 SV=2

```
0     MAVKISGVLKDGTGKPVQNCTIQLKAKRNSTTVVVNTLASENPDEAGRYS

50    MDVEYGQYSVILLVEGFPPSHAGTITVYEDSQPGTLNDFLGAMTEDDARP

100   EALRRFELMVEEVARNASAVAQNTAAAKKSASDASTSAREAATHAADAAD

150   SARAASTSAGQAASSAQSASSSAGTASTKATEASKSAAAAESSKSAAATS
                                             ***********
200   AGAAKTSETNASASLQSAATSASTATTKASEAATSARDAAASKEAAKSSE
      *************
250   TNASSSASSAASSATAAGNSAKAAKTSETNARSSETAAGQSASAAAGSKT

300   AAASSASAASTSAGQASASATAAGKSAESAASSASTATTKAGEATEQASA
                     ***********************************
350   AARSASAAKTSETNAKASETSAESSKTAAASSASSAASSASSASASKDEA
      *
400   TRQASAAKSSATTASTKATEAAGSATAAAQSKSTAESAATRAETAAKRAE

450   DIASAVALEDASTTKKGIVQLSSATNSTSETLAATPKAVKSAYDNAEKRL

500   QKDQNGADIPDKGCFLNNINAVSKTDFADKRGMRYVRVNAPAGATSGKYY

550   PVVVMRSAGSVSELASRVIITTATRTAGDPMNNCEFNGFVMPGGWTDRGR

600   YAYGMFWQYQNNERAIHSIMMSNKGDDLRSVFYVDGAAFPVFAFIEDGLS

650   ISAPGADLVVNDTTYKFGATNPATECIAADVILDFKSGRGFYESHSLIVN

700   DNLSCKKLFATDEIVARGGNQIRMIGGEYGALWRNDGAKTYLLLTNQGDV

750   YGGWNTLRPFAIDNATGELVIGTKLSASLNGNALTATKLQTPRRVSGVEF

800   DGSKDITLTAAHVAAFARRATDTYADADGGVPWNAESGAYNVTRSGDSYI

850   LVNFYTGVGSCRTLQMKAHYRNGGLFYRSSRDGYGFEEDWAEVYTSKNLP

900   PESYPVGAPIPWPSDTVPSGYALMQGQAFDKSAYPKLAAAYPSGVIPDMR

950   GWTIKGKPASGRAVLSQEQDGIKSHTHSASASSTDLGTKTTSSFDYGTKS

1000  TNNTGAHTHSVSGSTNSAGAHTHSLANVNTASANSGAGSASTRLSVVHNQ

1050  NYATSSAGAHTHSLSGTAASAGAHAHTVGIGAHTHSVAIGSHGHTITVNA

1100  AGNAENTVKNIAFNYIVRLA
```

sp|P26266|FEPE_ECOLI Ferric enterobactin transport protein FepE OS=Escherichia coli (strain K12) OX=83333 GN=fepE PE=1 SV=2

0 MSSLNIKQGSDAHFPDYPLASPSNNEIDLLNLISVLWRAKKTVMAVVFAF

50 ACAGLLISFILPQKWTSAAVVTPPEPVQWQELEKSFTKLRVLDLDIKIDR
*****
100 TEAFNLFIKKFQSVSLLEEYLRSSPYVMDQLKEAKIDELDLHRAIVALSE
****
150 KMKAVDDNASKKKDEPSLYTSWTLSFTAPTSEEAQTVLSGYIDYISTLVV

200 KESLENVRNKLEIKTQFEKEKLAQDRIKTKNQLDANIQRLNYSLDIANAA

250 GIKKPVYSNGQAVKDDPDFSISLGADGIERKLEIEKAVTDVAELNGELRN

300 RQYLVEQLTKAHVNDVNFTPFKYQLSPSLPVKKDGPGKAIIVILSALIGG

350 MVACGGVLLRYAMASRKQDAMMADHLV

sp|P0AE63|CHAB_ECOLI Putative cation transport regulator ChaB OS=Escherichia coli (strain K12) OX=83333 GN=chaB PE=3 SV=1
****
0 MPYKTKSDLPESVKHVLPSHAQDIYKEAFNSAWDQYKDKEDRRDDASREE

50 TAHKVAWAAVKHEYAKGDDDKWHKKS

sp|P23325|ARPA_ECOLI Ankyrin repeat protein A OS=Escherichia coli (strain K12) OX=83333 GN=arpA PE=4 SV=3

0 MITRIPRSSFSANINNTAQTNEHQTLSELFYKELEDKFSGKELATPLLKS

50 FSENCRQNGRHIFSNKDFVIKFSTSVLQADKKEITIINKNENTTLTQTIA
****
100 PIFEKYLMEILPQRSDTLDKQELNLKSDRKEKEFPRIKLNGQCYFPGRPQ

150 NRIVCRHIAAQYINDIYQNVDYKPHQDDYSSAEKFLTHFNKKCKNQTLAL

200 VSSRPEGRCVAACGDFGLVMKAYFDKMESNGISVMAAILLVDNHALTVRL

250 RIKNTTEGCTHYVVSVYDPNVTNDKIRIMSESKENIKHYSLMDFMNVDYS

300 LLKWSNDHVINQSVAIIPALPKEQLLMLKGSVDEITPPLSPATMNLLMAI

350 GQNHQLTQLMIQLQKMPELHRTEMLTAYNSINLPGLYLAINYGNADIVET

400 IFNSLSETGYEGLLSKKNLMHILEAKDKNGFSGLFLAISRKDKNVVTSIL

450 NALPKLAATHHLDNEQVYKFLSAKNRTSSHVLYHVMANGDADMLKIVLNA

500 LPLLIRTCHLTKEQVLDLLKAKDFYGCPGLYLAMQNGHSDIVKVILEALP

550 SLAQEINISASDIVDLLTAKSLARDTGLFMAMQRGHMNVINTIFNALPTL

600 FNTFKFDKKNMKPLLLANNSNEYPGLFSAIQHKQQNVVETVYLALSDHAR

650 LFGFTAEDIMDFWQHKAPQKYSAFELAFEFGHRVIAELILNTLNKMAESF

700   GFTDNPRYIAEKNYMEALLKKASPHTVR

sp|P32672|PTFC2_ECOLI Fructose-like permease IIC component 2 OS=Escherichia coli (strain K12) OX=83333 GN=frwC PE=3 SV=1

0   MNELVQILKNTRQHLMTGVSHMIPFVVSGGILLAVSVMLYGKGAVPDAVA

50   DPNLKKLFDIGVAGLTLMVPFLAAYIGYSIAERSALAPCAIGAWVGNSFG

100   AGFFGALIAGIIGGIVVHYLKKIPVHKVLRSVMPIFIIPIVGTLITAGIM

150   MWGLGEPVGALTNSLTQWLQGMQQGSIVMLAVIMGLMLAFDMGGPVNKVA

200   YAFMLICVAQGVYTVVAIAAVGICIPPLGMGLATLIGRKNFSAEERETGK

250   AALVMGCVGVTEGAIPFAAADPLRVIPSIMVGSVCGAVTAALVGAQCYAG
                                       ****
300   WGGLIVLPVVEGKLGYIAAVAVGAVVTAVCVNVLKSLARKNGSSTDEKED
      ****
350   DLDLDFEIN

sp|P39321|TAMB_ECOLI Translocation and assembly module subunit TamB OS=Escherichia coli (strain K12) OX=83333 GN=tamB PE=1 SV=2

0   MSLWKKISLGVVIVILLLLGSVAFLVGTTSGLHLVFKAADRWVPGLDIGK

50   VTGGWRDLTLSDVRYEQPGVAVKAGNLHLAVGLECLWNSSVCINDLALKD
        ****
100   IQVNIDSKKMPPSEQVEEEEDSGPLDLSTPYPITLTRVALDNVNIKIDDT

150   TVSVMDFTSGLNWQEKTLTLKPTSLKGLLIALPKVAEVAQEEVVEPKIEN

200   PQPDEKPLGETLKDLFSRPVLPEMTDVHLPLNLNIEEFKGEQLRVTGDTD

250   ITVSTMLLKVSSIDGNTKLDALDIDSSQGIVNASGTAQLSDNWPVDITLN

300   STLNVEPLKGEKVKLKMGGALREQLEIGVNLSGPVDMDLRAQTRLAEAGL

350   PLNVEVNSKQLYWPFTGEKQYQADDLKLKLTGKMTDYTLSMRTAVKGQEI

400   PPATITLDAKGNEQQVNLDKLTVAALEGKTELKALLDWQQAISWRGELTL

450   NGINTAKEFPDWPSKLNGLIKTRGSLYGGTWQMDVPELKLTGNVKQNKVN

500   VDGTLKGNSYMQWMIPGLHLELGPNSAEVKGELGVKDLNLDATINAPGLD

550   NALPGLGGTAKGLVKVRGTVEAPQLLADITARGLRWQELSVAQVRVEGDI

600   KSTDQIAGKLDVRVEQISQPDVNINLVTLNAKGSEKQHELQLRIQGEPVS

650   GQLNLAGSFDRKEERWKGTLSNTRFQTPVGPWSLTRDIALDYRNKEQKIS

700   IGPHCWLNPNAELCVPQTIDAGAEGRAVVNLNRFDLAMLKPFMPETTQAS

750   GIFTGKADVAWDTTKEGLPQGSITLSGRNVQVTQTVNDAALPVAFQTLNL

800   TAELRNNRAELGWTIRLTNNGQFDGQVQVTDPQGRRNLGGNVNIRNFNLA

850   MINPIFTRGEKAAGMVSANLRLGGDVQSPQLFGQLQVTGVDIDGNFMPFD

900   MQPSQLAVNFNGMRSTLAGTVRTQQGEIYLNGDADWSQIENWRARVTAKG

950   SKVRITVPPMVRMDVSPDVVFEATPNLFTLDGRVDVPWARIVVHDLPESA

1000 VGVSSDVVMLNDNLQPEEPKTASIPINSNLIVHVGNNVRIDAFGLKARLT

1050 GDLNVVQDKQGLGLNGQINIPEGRFHAYGQDLIVRKGELLFSGPPDQPYL

1100 NIEAIRNPDATEDDVIAGVRVTGLADEPKAEIFSDPAMSQQAALSYLLRG

1150 QGLESDQSDSAAMTSMLIGLGVAQSGQIVGKIGETFGVSNLALDTQGVGD

1200 SSQVVVSGYVLPGLQVKYGVGIFDSIATLTLRYRLMPKLYLEAVSGVDQA

1250 LDLLYQFEF

sp|P64499|YEBO_ECOLI Uncharacterized protein YebO OS=Escherichia coli (strain K12) OX=83333 GN=yebO PE=4 SV=1

```
0     MNEVVNSGVMNIASLVVSVVVLLIGLILWFFINRASSRTNEQIELLEALL
                   *************************
50    DQQKRQNALLRRLCEANEPEKADKKTVESQKSVEDEDIIRLVAER
```

sp|P00959|SYM_ECOLI Methionine--tRNA ligase OS=Escherichia coli (strain K12) OX=83333 GN=metG PE=1 SV=2

```
0     MTQVAKKILVTCALPYANGSIHLGHMLEHIQADVWVRYQRMRGHEVNFIC

50    ADDAHGTPIMLKAQQLGITPEQMIGEMSQEHQTDFAGFNISYDNYHSTHS

100   EENRQLSELIYSRLKENGFIKNRTISQLYDPEKGMFLPDRFVKGTCPKCK

150   SPDQYGDNCEVCGATYSPTELIEPKSVVSGATPVMRDSEHFFFDLPSFSE

200   MLQAWTRSGALQEQVANKMQEWFESGLQQWDISRDAPYFGFEIPNAPGKY
                        *******************
250   FYVWLDAPIGYMGSFKNLCDKRGDSVSFDEYWKKDSTAELYHFIGKDIVY

300   FHSLFWPAMLEGSNFRKPSNLFVHGYVTVNGAKMSKSRGTFIKASTWLNH

350   FDADSLRYYYTAKLSSRIDDIDLNLEDFVQRVNADIVNKVVNLASRNAGF

400   INKRFDGVLASELADPQLYKTFTDAAEVIGEAWESREFGKAVREIMALAD

450   LANRYVDEQAPWVVAKQEGRDADLQAICSMGINLFRVLMTYLKPVLPKLT

500   ERAEAFLNTELTWDGIQQPLLGHKVNPFKALYNRIDMRQVEALVEASKEE
```

550 VKAAAAPVTGPLADDPIQETITFDDFAKVDLRVALIENAEFVEGSDKLLR

600 LTLDLGGEKRNVFSGIRSAYPDPQALIGRHTIMVANLAPRKMRFGISEGM

650 VMAAGPGGKDIFLLSPDAGAKPGHQVK

sp|P0C066|MLTC_ECOLI Membrane-bound lytic murein transglycosylase C OS=Escherichia coli (strain K12) OX=83333 GN=mltC PE=1 SV=1

0 MKKYLALALIAPLLISCSTTKKGDTYNEAWVKDTNGFDILMGQFAHNIEN

50 IWGFKEVVIAGPKDYVKYTDQYQTRSHINFDDGTITIETIAGTEPAAHLR
****

100 RAIIKTLLMGDDPSSVDLYSDVDDITISKEPFLYGQVVDNTGQPIRWEGR

150 ASNFADYLLKNRLKSRSNGLRIIYSVTINMVPNHLDKRAHKYLGMVRQAS

200 RKYGVDESLILAIMQTESSFNPYAVSRSDALGLMQVVQHTAGKDVFRSQG

250 KSGTPSRSFLFDPASNIDTGTAYLAMLNNVYLGGIDNPTSRRYAVITAYN

300 GGAGSVLRVFSNDKIQAANIINTMTPGDVYQTLTTRHPSAESRRYLYKVN

350 TAQKSYRRR

sp|P0AFB1|NLPI_ECOLI Lipoprotein NlpI OS=Escherichia coli (strain K12) OX=83333 GN=nlpI PE=1 SV=1

0 MKPFLRWCFVATALTLAGCSNTSWRKSEVLAVPLQPTLQQEVILARMEQI

50 LASRALTDDERAQLLYERGVLYDSLGLRALARNDFSQALAIRPDMPEVFN

100 YLGIYLTQAGNFDAAYEAFDSVLELDPTYNYAHLNRGIALYYGGRDKLAQ
****

150 DDLLAFYQDDPNDPFRSLWLYLAEQKLDEKQAKEVLKQHFEKSDKEQWGW

200 NIVEFYLGNISEQTLMERLKADATDNTSLAEHLSETNFYLGKYYLSLGDL

250 DSATALFKLAVANNVHNFVEHRYALLELSLLGQDQDDLAESDQQ

sp|P76573|YFGI_ECOLI Uncharacterized protein YfgI OS=Escherichia coli (strain K12) OX=83333 GN=yfgI PE=4 SV=1

0 MKKVFLCAILASLSYPAIASSLQDQLSAVAEAEQQGKNEEQRQHDEWVAE
****

50 RNREIQQEKQRRANAQAAANKRAATAAANKKARQDKLDAEASADKKRDQS
****

100 YEDELRSLEIQKQKLALAKEEARVKRENEFIDQELKHKAAQTDVVQSEAD

150 ANRNMTEGGRDLMKSVGKAEENKSDSWFN

sp|P27297|BAX_ECOLI Protein bax OS=Escherichia coli (strain K12) OX=83333 GN=bax PE=4 SV=3

****
0 MILTPIRRYGAMILMLLTLVFSSEVLAKTHTTTASQKSHLTKASNKQVSS
****
50 KQEYSRNSAKSSSLPDLRKYPSGTPRKKAFLRTVMPYITSQNAAITAERN

100 WLISKQYQGQWSPAERARLKDIAKRYKVKWSGNTRKIPWNTLLERVDIIP

150 TSMVATMAAAESGWGTSKLARNNNNLFGMKCMKGRCTNAPGKVKGYSQFS

200 SVKESVSAYVTNLNTHPAYSSFRKSRAQLRKADQEVTATAMIHKLKGYST

250 KGKSYNNYLFAMYQDNQRLIAAHM

sp|P43674|YCAL_ECOLI Metalloprotease YcaL OS=Escherichia coli (strain K12) OX=83333 GN=ycaL PE=1 SV=3

0 MKNTKLLLAIATSAALLTGCQNTHGIDTNMAISSGLNAYKAATLSDADAK

50 AIANQGCAEMDSGNQVASKSSKYGKRLAKIAKALGNNINGTPVNYKVYMT

100 SDVNAWAMANGCVRVYSGLMDMMNDNEIEGVLGHELGHVALGHSLAEMKA
****
150 SYAIVAARDAISATSGVASQLSRSQLGDIAEGAINAKYSRDKESEADDFS
****
200 FDLLKKRGISTQGLVGSFETLASLDGGRTQSMFDSHPPSTERAQHIRDRI

250 ASGK

sp|P31667|RPNA_ECOLI Recombination-promoting nuclease RpnA OS=Escherichia coli (strain K12) OX=83333 GN=rpnA PE=1 SV=1
****
0 MSKKQSSTPHDALFKLFLRQPDTARDFLAFHLPAPIHALCDMKTLKLESS
****
50 SFIDDDLRESYSDVLWSVKTEQGPGYIYCLIEHQSTSNKLIAFRMMRYAI

100 AAMQNHLDAGYKTLPMVVPLLFYHGIESPYPYSLCWLDCFADPKLARQLY

150 ASAFPLIDVTVMPDDEIMQHRRMALLELIQKHIRQRDLMGLVEQMACLLS

200 SGYANDRQIKGLFNYILQTGDAVRFNDFIDGVAERSPKHKESLMTIAERL

250 RQEGEQSKALHIAKIMLESGVPLADIMRFTGLSEEELAAASQ

sp|P0A8H8|YACG_ECOLI DNA gyrase inhibitor YacG OS=Escherichia coli (strain K12) OX=83333 GN=yacG PE=1 SV=1
****
0 MSETITVNCPTCGKTVVWGEISPFRPFCSKRCQLIDLGEWAAEEKRIPSS
****
50 GDLSESDDWSEEPKQ

sp|P0AES6|GYRB_ECOLI DNA gyrase subunit B OS=Escherichia coli (strain K12) OX=83333 GN=gyrB PE=1 SV=2

0 MSNSYDSSSIKVLKGLDAVRKRPGMYIGDTDDGTGLHHMVFEVVDNAIDE

50 ALAGHCKEIIVTIHADNSVSVQDDGRGIPTGIHPEEGVSAAEVIMTVLHA

100 GGKFDDNSYKVSGGLHGVGVSVVNALSQKLELVIQREGKIHRQIYEHGVP

150 QAPLAVTGETEKTGTMVRFWPSLETFTNVTEFEYEILAKRLRELSFLNSG

200 VSIRLRDKRDGKEDHFHYEGGIKAFVEYLNKNKTPIHPNIFYFSTEKDGI

250 GVEVALQWNDGFQENIYCFTNNIPQRDGGTHLAGFRAAMTRTLNAYMDKE
****

300 GYSKKAKVSATGDDAREGLIAVVSVKVPDPKFSSQTKDKLVSSEVKSAVE

350 QQMNELLAEYLLENPTDAKIVVGKIIDAARAREAARRAREMTRRKGALDL

400 AGLPGKLADCQERDPALSELYLVEGDSAGGSAKQGRNRKNQAILPLKGKI

450 LNVEKARFDKMLSSQEVATLITALGCGIGRDEYNPDKLRYHSIIIMTDAD

500 VDGSHIRTLLLTFFYRQMPEIVERGHVYIAQPPLYKVKKGKQEQYIKDDE

550 AMDQYQISIALDGATLHTNASAPALAGEALEKLVSEYNATQKMINRMERR

600 YPKAMLKELIYQPTLTEADLSDEQTVTRWVNALVSELNDKEQHGSQWKFD

650 VHTNAEQNLFEPIVRVRTHGVDTDYPLDHEFITGGEYRRICTLGEKLRGL

700 LEEDAFIERGERRQPVASFEQALDWLVKESRRGLSIQRYKGLGEMNPEQL

750 WETTMDPESRRMLRVTVKDAIAADQLFTTLMGDAVEPRRAFIEENALKAA

800 NIDI

sp|P71297|YAGN_ECOLI Uncharacterized protein YagN OS=Escherichia coli (strain K12) OX=83333 GN=yagN PE=4 SV=1

0 MATPATVSIEPTLAAIRARWCINSSKTTQSFNDPASMEEVVEYLKGTYSA
****

50 LRKSVACAKLKILHLKQRMQNATNFLARLMSCKNQASRSHHSTAKSAKSA
****

100 LSSDSGDGSDPDPEPETFPSAFITTPTNSIMLKAFFANISITEVAK

sp|P0AEC5|BARA_ECOLI Signal transduction histidine-protein kinase BarA OS=Escherichia coli (strain K12) OX=83333 GN=barA PE=1 SV=1

0 MTNYSLRARMMILILAPTVLIGLLLSIFFVVHRYNDLQRQLEDAGASIIE

50 PLAVSTEYGMSLQNRESIGQLISVLHRRHSDIVRAISVYDENNRLFVTSN
****

100 FHLDPSSMQLGSNVPFPRQLTVTRDGDIMILRTPIISESYSPDESPSSDA
******

150 KNSQNMLGYIALELDLKSVRLQQYKEIFISSVMMLFCIGIALIFGWRLMR

200 DVTGPIRNMVNTVDRIRRGQLDSRVEGFMLGELDMLKNGINSMAMSLAAY

250   HEEMQHNIDQATSDLRETLEQMEIQNVELDLAKKRAQEAARIKSEFLANM

300   SHELRTPLNGVIGFTRLTLKTELTPTQRDHLNTIERSANNLLAIINDVLD

350   FSKLEAGKLILESIPFPLRSTLDEVVTLLAHSSHDKGLELTLNIKSDVPD

400   NVIGDPLRLQQIITNLVGNAIKFTENGNIDILVEKRALSNTKVQIEVQIR

450   DTGIGIPERDQSRLFQAFRQADASISRRHGGTGLGLVITQKLVNEMGGDI

500   SFHSQPNRGSTFWFHINLDLNPNIIIEGPSTQCLAGKRLAYVEPNSAAAQ

550   CTLDILSETPLEVVYSPTFSALPPAHYDMMLLGIAVTFREPLTMQHERLA

600   KAVSMTDFLMLALPCHAQVNAEKLKQDGIGACLLKPLTPTRLLPALTEFC

650   HHKQNTLLPVTDESKLAMTVMAVDDNPANLKLIGALLEDMVQHVELCDSG

700   HQAVERAKQMPFDLILMDIQMPDMDGIRACELIHQLPHQQQTPVIAVTAH

750   AMAGQKEKLLGAGMSDYLAKPIEEERLHNLLLRYKPGSGISSRVVTPEVN

800   EIVVNPNATLDWQLALRQAAGKTDLARDMLQMLLDFLPEVRNKVEEQLVG

850   ENPEGLVDLIHKLHGSCGYSGVPRMKNLCQLIEQQLRSGTKEEDLEPELL

900   ELLDEMDNVAREASKILG

sp|P0A6V1|GLGC_ECOLI Glucose-1-phosphate adenylyltransferase
OS=Escherichia coli (strain K12) OX=83333 GN=glgC PE=1 SV=2

0   MVSLEKNDHLMLARQLPLKSVALILAGGRGTRLKDLTNKRAKPAVHFGGK

50   FRIIDFALSNCINSGIRRMGVITQYQSHTLVQHIQRGWSFFNEEMNEFVD

100   LLPAQQRMKGENWYRGTADAVTQNLDIIRRYKAEYVVILAGDHIYKQDYS

150   RMLIDHVEKGARCTVACMPVPIEEASAFGVMAVDENDKIIEFVEKPANPP
                             ****
200   SMPNDPSKSLASMGIYVFDADYLYELLEEDDRDENSSHDFGKDLIPKITE

250   AGLAYAHPFPLSCVQSDPDAEPYWRDVGTLEAYWKANLDLASVVPELDMY

300   DRNWPIRTYNESLPPAKFVQDRSGSHGMTLNSLVSGGCVISGSVVVQSVL

350   FSRVRVNSFCNIDSAVLLPEVWVGRSCRLRRCVIDRACVIPEGMVIGENA

400   EEDARRFYRSEEGIVLVTREMLRKLGHKQER

sp|P26218|BGLH_ECOLI Cryptic outer membrane porin BglH OS=Escherichia
coli (strain K12) OX=83333 GN=bglH PE=1 SV=3
                                                    ****
0   MFRRNLITSAILLMAPLAFSAQSLAESLTVEQRLELLEKALRETQSELKK

```
      ************
50    YKDEEKKKYTPATVNRSVSTNDQGYAANPFPTSSAAKPDAVLVKNEEKNA

100   SETGSIYSSMTLKDFSKFVKDEIGFSYNGYYRSGWGTASHGSPKSWAIGS

150   LGRFGNEYSGWFDLQLKQRVYNENGKRVDAVVMMDGNVGQQYSTGWFGDN

200   AGGENYMQFSDMYVTTKGFLPFAPEADFWVGKHGAPKIEIQMLDWKTQRT

250   DAAAGVGLENWKVGPGKIDIALVREDVDDYDRSLQNKQQINTNTIDLRYK

300   DIPLWDKATLMVSGRYVTANESASEKDNQDNNGYYDWKDTWMFGTSLTQK

350   FDKGGFNEFSFLVANNSIASNFGRYAGASPFTTFNGRYYGDHTGGTAVRL

400   TSQGEAYIGDHFIVANAIVYSFGNDIYSYETGAHSDFESIRAVVRPAYIW

450   DQYNQTGVELGYFTQQNKDANSNKFNESGYKTTLFHTFKVNTSMLTSRPE

500   IRFYATYIKALENELDGFTFEDNKDDQFAVGAQAEIWW
```

sp|P02919|PBPB_ECOLI Penicillin-binding protein 1B OS=Escherichia coli (strain K12) OX=83333 GN=mrcB PE=1 SV=2

```
                         *******************************
0     MAGNDREPIGRKGKPTRPVKQKVSRRRYEDDDDYDDYDDYEDEEPMPRKG
      *
50    KGKGKGRKPRGKRGWLWLLLKLAIVFAVLIAIYGVYLDQKIRSRIDGKVW

100   QLPAAVYGRMVNLEPDMTISKNEMVKLLEATQYRQVSKMTRPGEFTVQAN

150   SIEMIRRPFDFPDSKEGQVRARLTFDGDHLATIVNMENNRQFGFFRLDPR

200   LITMISSPNGEQRLFVPRSGFPDLLVDTLLATEDRHFYEHDGISLYSIGR

250   AVLANLTAGRTVQGASTLTQQLVKNLFLSSERSYWRKANEAYMALIMDAR

300   YSKDRILELYMNEVYLGQSGDNEIRGFPLASLYYFGRPVEELSLDQQALL

350   VGMVKGASIYNPWRNPKLALERRNLVLRLLQQQQIIDQELYDMLSARPLG

400   VQPRGGVISPQPAFMQLVRQELQAKLGDKVKDLSGVKIFTTFDSVAQDAA

450   EKAAVEGIPALKKQRKLSDLETAIVVVDRFSGEVRAMVGGSEPQFAGYNR

500   AMQARRSIGSLAKPATYLTALSQPKIYRLNTWIADAPIALRQPNGQVWSP

550   QNDDRRYSESGRVMLVDALTRSMNVPTVNLGMALGLPAVTETWIKLGVPK

600   DQLHPVPAMLLGALNLTPIEVAQAFQTIASGGNRAPLSALRSVIAEDGKV

650   LYQSFPQAERAVPAQAAYLTLWTMQQVVQRGTGRQLGAKYPNLHLAGKTG

700   TTNNNVDTWFAGIDGSTVTITWVGRDNNQPTKLYGASGAMSIYQRYLANQ
```

750   TPTPLNLVPPEDIADMGVDYDGNFVCSGGMRILPVWTSDPQSLCQQSEMQ

800   QQPSGNPFDQSSQPQQQPQQQPAQQEQKDSDGVAGWIKDMFGSN

sp|P10408|SECA_ECOLI Protein translocase subunit SecA OS=Escherichia coli (strain K12) OX=83333 GN=secA PE=1 SV=2

0   MLIKLLTKVFGSRNDRTLRRMRKVVNIINAMEPEMEKLSDEELKGKTAEF

50   RARLEKGEVLENLIPEAFAVVREASKRVFGMRHFDVQLLGGMVLNERCIA

100   EMRTGEGKTLTATLPAYLNALTGKGVHVVTVNDYLAQRDAENNRPLFEFL

150   GLTVGINLPGMPAPAKREAYAADITYGTNNEYGFDYLRDNMAFSPEERVQ
                                                       *
200   RKLHYALVDEVDSILIDEARTPLIISGPAEDSSEMYKRVNKIIPHLIRQE
      ****
250   KEDSETFQGEGHFSVDEKSRQVNLTERGLVLIEELLVKEGIMDEGESLYS

300   PANIMLMHHVTAALRAHALFTRDVDYIVKDGEVIIVDEHTGRTMQGRRWS

350   DGLHQAVEAKEGVQIQNENQTLASITFQNYFRLYEKLAGMTGTADTEAFE

400   FSSIYKLDTVVVPTNRPMIRKDLPDLVYMTEAEKIQAIIEDIKERTAKGQ

450   PVLVGTISIEKSELVSNELTKAGIKHNVLNAKFHANEAAIVAQAGYPAAV

500   TIATNMAGRGTDIVLGGSWQAEVAALENPTAEQIEKIKADWQVRHDAVLE

550   AGGLHIIGTERHESRRIDNQLRGRSGRQGDAGSSRFYLSMEDALMRIFAS

600   DRVSGMMRKLGMKPGEAIEHPWVTKAIANAQRKVESRNFDIRKQLLEYDD

650   VANDQRRAIYSQRNELLDVSDVSETINSIREDVFKATIDAYIPPQSLEEM

700   WDIPGLQERLKNDFDLDLPIAEWLDKEPELHEETLRERILAQSIEVYQRK

750   EEVVGAEMMRHFEKGVMLQTLDSLWKEHLAAMDYLRQGIHLRGYAQKDPK

800   QEYKRESFSMFAAMLESLKYEVISTLSKVQVRMPEEVEELEQQRRMEAER

850   LAQMQQLSHQDDDSAAAAALAAQTGERKVGRNDPCPCGSGKKYKQCHGRL

900   Q

sp|P33230|RCBA_ECOLI Double-strand break reduction protein OS=Escherichia coli (strain K12) OX=83333 GN=rcbA PE=1 SV=2
                         ****
0   MYKITATIEKEGGTPTNWTRYSKSKLTKSECEKMLSGKKEAGVSREQKVK

50   LINFNCEKLQSSRIALYSN

sp|P76069|YDAY_ECOLI Protein YdaY OS=Escherichia coli (strain K12) OX=83333 GN=ydaY PE=1 SV=1

                    ****
0   MSRSSDNDQYRSRNALIRRHIEKMDASLHVGTKEFDISKVSEVDSVDDLL
      **
50   IDNAARYLLKDWKGVGELVNGVEVALEYTAERGIALLKQNPELYWQILAE

100   AASIAQGKEQQKQDTIKKP

sp|P0AEC3|ARCB_ECOLI Aerobic respiration control sensor protein ArcB OS=Escherichia coli (strain K12) OX=83333 GN=arcB PE=1 SV=1

0   MKQIRLLAQYYVDLMMKLGLVRFSMLLALALVVLAIVVQMAVTMVLHGQV

50   ESIDVIRSIFFGLLITPWAVYFLSVVVEQLEESRQRLSRLVQKLEEMRER

100   DLSLNVQLKDNIAQLNQEIAVREKAEAELQETFGQLKIEIKEREETQIQL

150   EQQSSFLRSFLDASPDLVFYRNEDKEFSGCNRAMELLTGKSEKQLVHLKP

200   ADVYSPEAAAKVIETDEKVFRHNVSLTYEQWLDYPDGRKACFEIRKVPYY

250   DRVGKRHGLMGFGRDITERKRYQDALERASRDKTTFISTISHELRTPLNG

300   IVGLSRILLDTELTAEQEKYLKTIHVSAVTLGNIFNDIIDMDKMERRKVQ

350   LDNQPVDFTSFLADLENLSALQAQQKGLRFNLEPTLPLPHQVITDGTRLR

400   QILWNLISNAVKFTQQGQVTVRVRYDEGDMLHFEVEDSGIGIPQDELDKI

450   FAMYYQVKDSHGGKPATGTGIGLAVSRRLAKNMGGDITVTSEQGKGSTFT

500   LTIHAPSVAEEVDDAFDEDDMPLPALNVLLVEDIELNVIVARSVLEKLGN

550   SVDVAMTGKAALEMFKPGEYDLVLLDIQLPDMTGLDISRELTKRYPREDL
                                            ****
600   PPLVALTANVLKDKQEYLNAGMDDVLSKPLSVPALTAMIKKFWDTQDDEE
      ****
650   STVTTEENSKSEALLDIPMLEQYLELVGPKLITDGLAVFEKMMPGYVSVL

700   ESNLTAQDKKGIVEEGHKIKGAAGSVGLRHLQQLGQQIQSPDLPAWEDNV

750   GEWIEEMKEEWRHDVEVLKAWVAKATKK

sp|P77359|DJLC_ECOLI Uncharacterized J domain-containing protein DjlC OS=Escherichia coli (strain K12) OX=83333 GN=djlC PE=4 SV=1

0   MKTCWQILEIESTTQIDIIRQAYLARLPLCHPETDPQGFKALRQAYEEAL

50   RLAVNPVEEADDEEKDAAAEHEILRAFRTLLDSESDRFQPSAWQKFIQQL

100   NTWNMEDVDQLRWPLCAIAIEARYLSLNCASLLAERLNWHSFNDSEGMDE

150   EEREAFLEAIQAGDCFDFLSLLEYPIALQNQTVEYYFALERCCRYHPDYV

200   TAFLAMEGPWLIPDDAKLHRKLLRWYSSVQTGMAELIPVAQQWQTEEPES

```
250   EDARYYLCAQRLYCGEGESLLADLCAYWESYPSTQADNLLLQWSKRHCPD

300   YFALLVMVIEARSMVDAQGQPLKYVPGESARTRLLWAEILHSGKLSPLGQ

350   SFIESLFFKRKAWAWWKSRVGSETEQDSPFLDLYRVAEQVVLEAFPKQEM
                                  **********************
400   LARLNTRLEGGDAHPLEAIVTRMLLTKVKLEPEDEDVDEPTPENHEEKND
      ****
450   EGEKPQSITSIIKISLTVLVIGYALGKIAMLFS
```

sp|P21177|FADB_ECOLI Fatty acid oxidation complex subunit alpha OS=Escherichia coli (strain K12) OX=83333 GN=fadB PE=1 SV=2

```
0     MLYKGDTLYLDWLEDGIAELVFDAPGSVNKLDTATVASLGEAIGVLEQQS

50    DLKGLLLRSNKAAFIVGADITEFLSLFLVPEEQLSQWLHFANSVFNRLED

100   LPVPTIAAVNGYALGGGCECVLATDYRLATPDLRIGLPETKLGIMPGFGG

150   SVRMPRMLGADSALEIIAAGKDVGADQALKIGLVDGVVKAEKLVEGAKAV

200   LRQAINGDLDWKAKRQPKLEPLKLSKIEATMSFTIAKGMVAQTAGKHYPA

250   PITAVKTIEAAARFGREEALNLENKSFVPLAHTNEARALVGIFLNDQYVK

300   GKAKKLTKDVETPKQAAVLGAGIMGGGIAYQSAWKGVPVVMKDINDKSLT

350   LGMTEAAKLLNKQLERGKIDGLKLAGVISTIHPTLDYAGFDRVDIVVEAV

400   VENPKVKKAVLAETEQKVRQDTVLASNTSTIPISELANALERPENFCGMH

450   FFNPVHRMPLVEIIRGEKSSDETIAKVVAWASKMGKTPIVVNDCPGFFVN

500   RVLFPYFAGFSQLLRDGADFRKIDKVMEKQFGWPMGPAYLLDVVGIDTAH
                                                 ***********
550   HAQAVMAAGFPQRMQKDYRDAIDALFDANRFGQKNGLGFWRYKEDSKGKP
      **************
600   KKEEDAAVEDLLAEVSQPKRDFSEEEIIARMMIPMVNEVVRCLEEGIIAT

650   PAEADMALVYGLGFPPFHGGAFRWLDTLGSAKYLDMAQQYQHLGPLYEVP

700   EGLRNKARHNEPYYPPVEPARPVGDLKTA
```

sp|P11557|DAMX_ECOLI Cell division protein DamX OS=Escherichia coli (strain K12) OX=83333 GN=damX PE=1 SV=2

```
0     MDEFKPEDELKPDPSDRRTGRSRQSSERSERTERGEPQINFDDIELDDTD
           ***********************
50    DRRPTRAQKERNEEPEIEEEIDESEDETVDEERVERRPRKRKKAASKPAS

100   RQYMMMGVGILVLLLLIIGIGSALKAPSTTSSDQTASGEKSIDLAGNATD

150   QANGVQPAPGTTSAENTQQDVSLPPISSTPTQGQTPVATDGQQRVEVQGD
```

200   LNNALTQPQNQQQLNNVAVNSTLPTEPATVAPVRNGNASRDTAKTQTAER

250   PSTTRPARQQAVIEPKKPQATVKTEPKPVAQTPKRTEPAAPVASTKAPAA

300   TSTPAPKETATTAPVQTASPAQTTATPAAGAKTAGNVGSLKSAPSSHYTL

350   QLSSSSNYDNLNGWAKKENLKNYVVYETTRNGQPWYVLVSGVYASKEEAK

400   KAVSTLPADVQAKNPWAKPLRQVQADLK

sp|P0AE45|YTFL_ECOLI UPF0053 inner membrane protein YtfL OS=Escherichia coli (strain K12) OX=83333 GN=ytfL PE=1 SV=1

0   MLNSILVILCLIAVSAFFSMSEISLAASRKIKLKLLADEGNINAQRVLNM

50   QENPGMFFTVVQIGLNAVAILGGIVGDAAFSPAFHSLFSRYMSAELSEQL

100   SFILSFSLVTGMFILFADLTPKRIGMIAPEAVALRIINPMRFCLYVCTPL

150   VWFFNGLANIIFRIFKLPMVRKDDITSDDIYAVVEAGALAGVLRKQEHEL

200   IENVFELESRTVPSSMTPRENVIWFDLHEDEQSLKNKVAEHPHSKFLVCN

250   EDIDHIIGYVDSKDLLNRVLANQSLALNSGVQIRNTLIVPDTLTLSEALE

300   SFKTAGEDFAVIMNEYALVVGIITLNDVMTTLMGDLVGQGLEEQIVARDE

350   NSWLIDGGTPIDDVMRVLDIDEFPQSGNYETIGGFMMFMLRKIPKRTDSV
                                 ****
400   KFAGYKFEVVDIDNYRIDQLLVTRIDSKATALSPKLPDAKDKEESVA

sp|P37327|YFDC_ECOLI Inner membrane protein YfdC OS=Escherichia coli (strain K12) OX=83333 GN=yfdC PE=1 SV=1
      ****
0   MDNDKIDQHSDEIEVESEEKERGKKIEIDEDRLPSRAMAIHEHIRQDGEK

50   ELERDAMALLWSAIAAGLSMGASLLAKGIFQVELEGVPGSFLLENLGYTF

100   GFIIVIMARQQLFTENTVTAVLPVMQKPTMSNVGLLIRLWGVVLLGNILG

150   TGIAAWAFEYMPIFNEETRDAFVKIGMDVMKNTPSEMFANAIISGWLIAT

200   MVWMFPAAGAAKIVVIILMTWLIALGDTTHIVVGSVEILYLVFNGTLHWS

250   DFIWPFALPTLAGNICGGTFIFALMSHAQIRNDMSNKRKAEARQKAERAE

300   NIKKNYKNPA

sp|P54746|MNGB_ECOLI Mannosylglycerate hydrolase OS=Escherichia coli (strain K12) OX=83333 GN=mngB PE=1 SV=2

0   MKAVSRVHITPHMHWDREWYFTTEESRILLVNNMEEILCRLEQDNEYKYY

50 VLDGQTAILEDYFAVKPENKDRVKKQVEAGKLIIGPWYTQTDTTIVSAES

100 IVRNLMYGMRDCLAFGEPMKIGYLPDSFGMSGQLPHIYNGFGITRTMFWR

150 GCSERHGTDKTEFLWQSSDGSEVTAQVLPLGYAIGKYLPADENGLRKRLD

200 SYFDVLEKASVTKEILLPNGHDQMPLQQNIFEVMDKLREIYPQRKFVMSR

250 FEEVFEKIEAQRDNLATLKGEFIDGKYMRVHRTIGSTRMDIKIAHARIEN

300 KIVNLLEPLATLAWTLGFEYHHGLLEKMWKEILKNHAHDSIGCCCSDKVH

350 REIVARFELAEDMADNLIRFYMRKIADNMPQSDADKLVLFNLMPWPREEV

400 INTTVRLRASQFNLRDDRGQPVPYFIRHAREIDPGLIDRQIVHYGNYDPF

450 MEFDIQINQIVPSMGYRTLYIEANQPGNVIAAKSDAEGILENAFWQIALN
****

500 EDGSLQLVDKDSGVRYDRVLQIEESSDDGDEYDYSPAKEEWVITAANAKP

550 QCDIIHEAWQSRAVIRYDMAVPLNLSERSARQSTGRVGVVLVVTLSHNSR

600 RIDVDINLDNQADDHRLRVLVPTPFNTDSVLADTQFGSLTRPVNDSAMNN

650 WQQEGWKEAPVPVWNMLNYVALQEGRNGMAVFSEGLREFEVIGEEKKTFA

700 ITLLRGVGLLGKEDLLLRPGRPSGIKMPVPDSQLRGLLSCRLSLLSYTGT

750 PTAAGVAQQARAWLTPVQCYNKIPWDVMKLNKAGFNVPESYSLLKMPPVG

800 CLISALKKAEDRQEVILRLFNPAESATCDATVAFSREVISCSETMMDEHI

850 TTEENQGSNLSGPFLPGQSRTFSYRLA

sp|P21499|RNR_ECOLI Ribonuclease R OS=Escherichia coli (strain K12) OX=83333 GN=rnr PE=1 SV=2

0 MSQDPFQEREAEKYANPIPSREFILEHLTKREKPASRDELAVELHIEGEE

50 QLEGLRRRLRAMERDGQLVFTRRQCYALPERLDLVKGTVIGHRDGYGFLR

100 VEGRKDDLYLSSEQMKTCIHGDQVLAQPLGADRKGRREARIVRVLVPKTS

150 QIVGRYFTEAGVGFVVPDDSRLSFDILIPPDQIMGARMGFVVVVELTQRP

200 TRRTKAVGKIVEVLGDNMGTGMAVDIALRTHEIPYIWPQAVEQQVAGLKE

250 EVPEEAKAGRVDLRDLPLVTIDGEDARDFDDAVYCEKKRGGGWRLWVAIA

300 DVSYYVRPSTPLDREARNRGTSVYFPSQVIPMLPEVLSNGLCSLNPQVDR

350 LCMVCEMTVSSKGRLTGYKFYEAVMSSHARLTYTKVWHILQGDQDLREQY

400 APLVKHLEELHNLYKVLDKAREERGGISFESEEAKFIFNAERRIERIEQT

450   QRNDAHKLIEECMILANISAARFVEKAKEPALFRIHDKPSTEAITSFRSV

500   LAELGLELPGGNKPEPRDYAELLESVADRPDAEMLQTMLLRSMKQAIYDP

550   ENRGHFGLALQSYAHFTSPIRRYPDLTLHRAIKYLLAKEQGHQGNTTETG

600   GYHYSMEEMLQLGQHCSMAERRADEATRDVADWLKCDFMLDQVGNVFKGV

650   ISSVTGFGFFVRLDDLFIDGLVHVSSLDNDYYRFDQVGQRLMGESSGQTY

700   RLGDRVEVRVEAVNMDERKIDFSLISSERAPRNVGKTAREKAKKGDAGKK
                           ****
750   GGKRRQVGKKVNFEPDSAFRGEKKTKPKAAKKDARKAKKPSAKTQKIAAA

800   TKAKRAAKKKVAE

sp|P0A6P5|DER_ECOLI GTPase Der OS=Escherichia coli (strain K12) OX=83333 GN=der PE=1 SV=1

0   MVPVVALVGRPNVGKSTLFNRLTRTRDALVADFPGLTRDRKYGRAEIEGR

50   EFICIDTGGIDGTEDGVETRMAEQSLLAIEEADVVLFMVDARAGLMPADE

100   AIAKHLRSREKPTFLVANKTDGLDPDQAVVDFYSLGLGEIYPIAASHGRG
                                        ****
150   VLSLLEHVLLPWMEDLAPQEEVDEDAEYWAQFEAEENGEEEEEDDFDPQS
      ****
200   LPIKLAIVGRPNVGKSTLTNRILGEERVVVYDMPGTTRDSIYIPMERDGR

250   EYVLIDTAGVRKRGKITDAVEKFSVIKTLQAIEDANVVMLVIDAREGISD

300   QDLSLLGFILNSGRSLVIVVNKWDGLSQEVKEQVKETLDFRLGFIDFARV

350   HFISALHGSGVGNLFESVREAYDSSTRRVGTSMLTRIMTMAVEDHQPPLV

400   RGRRVKLKYAHAGGYNPPIVVIHGNQVKDLPDSYKRYLMNYFRKSLDVMG

450   SPIRIQFKEGENPYANKRNTLTPTQMRKRKRLMKHIKKNK

sp|P64581|YQJD_ECOLI Uncharacterized protein YqjD OS=Escherichia coli (strain K12) OX=83333 GN=yqjD PE=1 SV=1
                 ****
0   MSKEHTTEHLRAELKSLSDTLEEVLSSSGEKSKEELSKIRSKAEQALKQS

50   RYRLGETGDAIAKQTRVAAARADEYVRENPWTGVGIGAAIGVVLGVLLSR

100   R

sp|P52005|TORY_ECOLI Cytochrome c-type protein TorY OS=Escherichia coli (strain K12) OX=83333 GN=torY PE=1 SV=2

0   MRGKKRIGLLFLLIAVVVGGGGLLLAQKVLHKTSDTAFCLSCHSMSKPFE
                                         ****

```
50    EYQGTVHFSNQKGIRAECADCHIPKSGMDYLFAKLKASKDIYHEFVSGKI
      *******
100   DSDDKFEAHRQEMAETVWKELKATDSATCRSCHSFDAMDIASQSESAQKM

150   HNKAQKDSETCIDCHKGIAHFPPEIKMDDNAAHELESQAATSVTNGAHIY

200   PFKTSHIGELATVNPGTDLTVVDASGKQPIVLLQGYQMQGSENTLYLAAG

250   QRLALATLSEEGIKALTVNGEWQADEYGNQWRQASLQGALTDPALADRKP

300   LWQYAEKLDDTYCAGCHAPIAADHYTVNAWPSIAKGMGARTSMSENELDI

350   LTRYFQYNAKDITEKQ
```

sp|P0A894|RAPZ_ECOLI RNase adapter protein RapZ OS=Escherichia coli (strain K12) OX=83333 GN=rapZ PE=1 SV=1

```
0     MVLMIVSGRSGSGKSVALRALEDMGFYCVDNLPVVLLPDLARTLADREIS

50    AAVSIDVRNMPESPEIFEQAMSNLPDAFSPQLLFLDADRNTLIRRYSDTR
          *********************
100   RLHPLSSKNLSLESAIDKESDLLEPLRSRADLIVDTSEMSVHELAEMLRT

150   RLLGKRERELTMVFESFGFKHGIPIDADYVFDVRFLPNPHWDPKLRPMTG

200   LDKPVAAFLDRHTEVHNFIYQTRSYLELWLPMLETNNRSYLTVAIGCTGG

250   KHRSVYIAEQLADYFRSRGKNVQSRHRTLEKRKP
```

sp|P31069|KCH_ECOLI Voltage-gated potassium channel Kch OS=Escherichia coli (strain K12) OX=83333 GN=kch PE=1 SV=1

```
0     MSHWATFKQTATNLWVTLRHDILALAVFLNGLLIFKTIYGMSVNLLDIFH

50    IKAFSELDLSLLANAPLFMLGVFLVLNSIGLLFRAKLAWAISIIILLLIAL

100   IYTLHFYPWLKFSIGFCIFTLVFLLILRKDFSHSSAAAGTIFAFISFTTL

150   LFYSTYGALYLSEGFNPRIESLMTAFYFSIETMSTVGYGDIVPVSESARL

200   FTISVIISGITVFATSMTSIFGPLIRGGFNKLVKGNNHTMHRKDHFIVCG

250   HSILAINTILQLNQRGQNVTVISNLPEDDIKQLEQRLGDNADVIPGDSND

300   SSVLKKAGIDRCRAILALSDNDADNAFVVLSAKDMSSDVKTVLAVSDSKN
                                                     ***
350   LNKIKMVHPDIILSPQLFGSEILARVLNGEEINNDMLVSMLLNSGHGIFS
      *****************
400   DNDELETKADSKESAQK
```

sp|P77804|YDGA_ECOLI Protein YdgA OS=Escherichia coli (strain K12) OX=83333 GN=ydgA PE=1 SV=1

```
0     MNKSLVAVGVIVALGVVWTGGAWYTGKKIETHLEDMVAQANAQLKLTAPE
```

```
50    SNLEVSYQNYHRGVFSSQLQLLVKPIAGKENPWIKSGQSVIFNESVDHGP
                                                     *
100   FPLAQLKKLNLIPSMASIQTTLVNNEVSKPLFDMAKGETPFEINSRIGYS
      *********************
150   GDSSSDISLKPLNYEQKDEKVAFSGGEFQLNADRDGKAISLSGEAQSGRI

200   DAVNEYNQKVQLTFNNLKTDGSSTLASFGERVGNQKLSLEKMTISVEGKE

250   LALLEGMEISGKSDLVNDGKTINSQLDYSLNSLKVQNQDLGSGKLTLKVG

300   QIDGEAWHQFSQQYNAQTQALLAQPEIANNPELYQEKVTEAFFSALPLML

350   KGDPVITIAPLSWKNSQGESALNLSLFLKDPATTKEAPQTLAQEVDRSVK

400   SLDAKLTIPVDMATEFMTQVAKLEGYQEDQAKKLAKQQVEGASAMGQMFR

450   LTTLQDNTITTSLQYANGQITLNGQKMSLEDFVGMFAMPALNVPAVPAIP

500   QQ
```

sp|P76272|YEBT_ECOLI Intermembrane transport protein YebT OS=Escherichia coli (strain K12) OX=83333 GN=yebT PE=1 SV=2

```
0     MSQETPASTTEAQIKNKRRISPFWLLPFIALMIASWLIWDSYQDRGNTVT
                                 ***********************
50    IDFMSADGIVPGRTPVRYQGVEVGTVQDISLSDDLRKIEVKVSIKSDMKD
      *
100   ALREETQFWLVTPKASLAGVSGLDALVGGNYIGMMPGKGKEQDHFVALDT

150   QPKYRLDNGDLMIHLQAPDLGSLNSGSLVYFRKIPVGKVYDYAINPNKQG

200   VVIDVLIERRFTDLVKKGSRFWNVSGVDANVSISGAKVKLESLAALVNGA

250   IAFDSPEESKPAEAEDTFGLYEDLAHSQRGVIIKLELPSGAGLTADSTPL

300   MYQGLEVGQLTKLDLNPGGKVTGEMTVDPSVVTLLRENTRIELRNPKLSL

350   SDANLSALLTGKTFELVPGDGEPRKEFVVVPGEKALLHEPDVLTLTLTAP

400   ESYGIDAGQPLILHGVQVGQVIDRKLTSKGVTFTVAIEPQHRELVKGDSK

450   FVVNSRVDVKVGLDGVEFLGASASEWINGGIRILPGDKGEMKASYPLYAN

500   LEKALENSLSDLPTTTVSLSAETLPDVQAGSVVLYRKFEVGEVITVRPRA

550   NAFDIDLHIKPEYRNLLTSNSVFWAEGGAKVQLNGSGLTVQASPLSRALK

600   GAISFDNLSGASASQRKGDKRILYASETAARAVGGQITLHAFDAGKLAVG

650   MPIRYLGIDIGQIQTLDLITARNEVQAKAVLYPEYVQTFARGGTRFSVVT

700   PQISAAGVEHLDTILQPYINVEPGRGNPRRDFELQEATITDSRYLDGLSI
```

750   IVEAPEAGSLGIGTPVLFRGLEVGTVTGMTLGTLSDRVMIAMRISKRYQH

800   LVRNNSVFWLASGYSLDFGLTGGVVKTGTFNQFIRGGIAFATPPGTPLAP

850   KAQEGKHFLLQESEPKEWREWGTALPK

sp|P00490|PHSM_ECOLI Maltodextrin phosphorylase OS=Escherichia coli (strain K12) OX=83333 GN=malP PE=1 SV=7

0   MSQPIFNDKQFQEALSRQWQRYGLNSAAEMTPRQWWLAVSEALAEMLRAQ

50   PFAKPVANQRHVNYISMEFLIGRLTGNNLLNLGWYQDVQDSLKAYDINLT

100   DLLEEEIDPALGNGGLGRLAACFLDSMATVGQSATGYGLNYQYGLFRQSF

150   VDGKQVEAPDDWHRSNYPWFRHNEALDVQVGIGGKVTKDGRWEPEFTITG

200   QAWDLPVVGYRNGVAQPLRLWQATHAHPFDLTKFNDGDFLRAEQQGINAE

250   KLTKVLYPNDNHTAGKKLRLMQQYFQCACSVADILRRHHLAGRKLHELAD

300   YEVIQLNDTHPTIAIPELLRVLIDEHQMSWDDAWAITSKTFAYTNHTLMP

350   EALERWDVKLVKGLLPRHMQIINEINTRFKTLVEKTWPGDEKVWAKLAVV

400   HDKQVHMANLCVVGGFAVNGVAALHSDLVVKDLFPEYHQLWPNKFHNVTN

450   GITPRRWIKQCNPALAALLDKSLQKEWANDLDQLINLEKFADDAKFRQQY

500   REIKQANKVRLAEFVKVRTGIEINPQAIFDIQIKRLHEYKRQHLNLLHIL

550   ALYKEIRENPQADRVPRVFLFGAKAAPGYYLAKNIIFAINKVADVINNDP

600   LVGDKLKVVFLPDYCVSAAEKLIPAADISEQISTAGKEASGTGNMKLALN
                                                    **
650   GALTVGTLDGANVEIAEKVGEENIFIFGHTVEQVKAILAKGYDPVKWRKK
      ****
700   DKVLDAVLKELESGKYSDGDKHAFDQMLHSIGKQGGDPYLVMADFAAYVE

750   AQKQVDVLYRDQEAWTRAAILNTARCGMFSSDRSIRDYQARIWQAKR

sp|P69428|TATA_ECOLI Sec-independent protein translocase protein TatA OS=Escherichia coli (strain K12) OX=83333 GN=tatA PE=1 SV=1
                                          ****
0   MGGISIWQLLIIAVIVVLLFGTKKLGSIGSDLGASIKGFKKAMSDDEPKQ
      ****
50   DKTSQDADFTAKTIADKQADTNQEQAKTEDAKRHDKEQV

sp|P0AEY1|MARC_ECOLI UPF0056 inner membrane protein MarC OS=Escherichia coli (strain K12) OX=83333 GN=marC PE=1 SV=1

0   MLDLFKAIGLGLVVLLPLANPLTTVALFLGLAGNMNSAERNRQSLMASVY
                                                 ****
50   VFAIMMVAYYAGQLVMDTFGISIPGLRIAGGLIVAFIGFRMLFPQQKAID

****
100   SPEAKSKSEELEDEPSANIAFVPLAMPSTAGPGTIAMIISSASTVRQSST

150   FADWVLMVAPPLIFFLVAVILWGSLRSSGAIMRLVGKGGIEAISRLMGFL

200   LVCMGVQFIINGILEIIKTYH

sp|P0A9Q5|ACCD_ECOLI Acetyl-coenzyme A carboxylase carboxyl transferase subunit beta OS=Escherichia coli (strain K12) OX=83333 GN=accD PE=1 SV=1

0   MSWIERIKSNITPTRKASIPEGVWTKCDSCGQVLYRAELERNLEVCPKCD
****
50   HHMRMTARNRLHSLLDEGSLVELGSELEPKDVLKFRDSKKYKDRLASAQK
******
100   ETGEKDALVVMKGTLYGMPVVAAAFEFAFMGGSMGSVVGARFVRAVEQAL

150   EDNCPLICFSASGGARMQEALMSLMQMAKTSAALAKMQERGLPYISVLTD

200   PTMGGVSASFAMLGDLNIAEPKALIGFAGPRVIEQTVREKLPPGFQRSEF

250   LIEKGAIDMIVRRPEMRLKLASILAKLMNLPAPNPEAPREGVVVPPVPDQ

300   EPEA

sp|P06612|TOP1_ECOLI DNA topoisomerase 1 OS=Escherichia coli (strain K12) OX=83333 GN=topA PE=1 SV=2
****
0   MGKALVIVESPAKAKTINKYLGSDYVVKSSVGHIRDLPTSGSAAKKSADS
****
50   TSTKTAKKPKKDERGALVNRMGVDPWHNWEAHYEVLPGKEKVVSELKQLA

100   EKADHIYLATDLDREGEAIAWHLREVIGGDDARYSRVVFNEITKNAIRQA

150   FNKPGELNIDRVNAQQARRFMDRVVGYMVSPLLWKKIARGLSAGRVQSVA

200   VRLVVEREREIKAFVPEEFWEVDASTTTPSGEALALQVTHQNDKPFRPVN

250   KEQTQAAVSLLEKARYSVLEREDKPTTSKPGAPFITSTLQQAASTRLGFG

300   VKKTMMMAQRLYEAGYITYMRTDSTNLSQDAVNMVRGYISDNFGKKYLPE

350   SPNQYASKENSQEAHEAIRPSDVNVMAESLKDMEADAQKLYQLIWRQFVA

400   CQMTPAKYDSTTLTVGAGDFRLKARGRILRFDGWTKVMPALRKGDEDRIL

450   PAVNKGDALTLVELTPAQHFTKPPARFSEASLVKELEKRGIGRPSTYASI

500   ISTIQDRGYVRVENRRFYAEKMGEIVTDRLEENFRELMNYDFTAQMENSL

550   DQVANHEAEWKAVLDHFFSDFTQQLDKAEKDPEEGGMRPNQMVLTSIDCP

600   TCGRKMGIRTASTGVFLGCSGYALPPKERCKTTINLVPENEVLNVLEGED

650   AETNALRAKRRCPKCGTAMDSYLIDPKRKLHVCGNNPTCDGYEIEEGEFR

```
700   IKGYDGPIVECEKCGSEMHLKMGRFGKYMACTNEECKNTRKILRNGEVAP

750   PKEDPVPLPELPCEKSDAYFVLRDGAAGVFLAANTFPKSRETRAPLVEEL

800   YRFRDRLPEKLRYLADAPQQDPEGNKTMVRFSRKTKQQYVSSEKDGKATG

850   WSAFYVDGKWVEGKK
```

sp|P0A8H6|YIHI_ECOLI Der GTPase-activating protein YihI OS=Escherichia coli (strain K12) OX=83333 GN=yihI PE=1 SV=1

```
0     MKPSSSNSRSKGHAKARRKTREELDQEARDRKRQKKRRGHAPGSRAAGGN

50    TTSGSKGQNAPKDPRIGSKTPIPLGVTEKVTKQHKPKSEKPMLSPQAELE
                                         ***************
100   LLETDERLDALLERLEAGETLSAEEQSWVDAKLDRIDELMQKLGLSYDDD
      ******************
150   EEEEEDEKQEDMMRLLRGN
```

sp|P0AAT9|YBEL_ECOLI Uncharacterized protein YbeL OS=Escherichia coli (strain K12) OX=83333 GN=ybeL PE=4 SV=1

```
0     MNKVAQYYRELVASLSERLRNGERDIDALVEQARERVIKTGELTRTEVDE
           *******************************
50    LTRAVRRDLEEFAMSYEESLKEESDSVFMRVIKESLWQELADITDKTQLE

100   WREVFQDLNHHGVYHSGEVVGLGNLVCEKCHFHLPIYTPEVLTLCPKCGH

150   DQFQRRPFEP
```

sp|P63284|CLPB_ECOLI Chaperone protein ClpB OS=Escherichia coli (strain K12) OX=83333 GN=clpB PE=1 SV=1

```
0     MRLDRLTNKFQLALADAQSLALGHDNQFIEPLHLMSALLNQEGGSVSPLL

50    TSAGINAGQLRTDINQALNRLPQVEGTGGDVQPSQDLVRVLNLCDKLAQK

100   RGDNFISSELFVLAALESRGTLADILKAAGATTANITQAIEQMRGGESVN

150   DQGAEDQRQALKKYTIDLTERAEQGKLDPVIGRDEEIRRTIQVLQRRTKN

200   NPVLIGEPGVGKTAIVEGLAQRIINGEVPEGLKGRRVLALDMGALVAGAK

250   YRGEFEERLKGVLNDLAKQEGNVILFIDELHTMVGAGKADGAMDAGNMLK

300   PALARGELHCVGATTLDEYRQYIEKDAALERRFQKVFVAEPSVEDTIAIL

350   RGLKERYELHHHVQITDPAIVAAATLSHRYIADRQLPDKAIDLIDEAASS
                                   *********************
400   IRMQIDSKPEELDRLDRRIIQLKLEQQALMKESDEASKKRLDMLNEELSD
      ********************
450   KERQYSELEEEWKAEKASLSGTQTIKAELEQAKIAIEQARRVGDLARMSE
```

```
500   LQYGKIPELEKQLEAATQLEGKTMRLLRNKVTDAEIAEVLARWTGIPVSR

550   MMESEREKLLRMEQELHHRVIGQNEAVDAVSNAIRRSRAGLADPNRPIGS

600   FLFLGPTGVGKTELCKALANFMFDSDEAMVRIDMSEFMEKHSVSRLVGAP

650   PGYVGYEEGGYLTEAVRRRPYSVILLDEVEKAHPDVFNILLQVLDDGRLT

700   DGQGRTVDFRNTVVIMTSNLGSDLIQERFGELDYAHMKELVLGVVSHNFR

750   PEFINRIDEVVVFHPLGEQHIASIAQIQLKRLYKRLEERGYEIHISDEAL

800   KLLSENGYDPVYGARPLKRAIQQQIENPLAQQILSGELVPGKVIRLEVNE

850   DRIVAVQ
```

sp|P31801|CHAA_ECOLI Sodium-potassium/proton antiporter ChaA OS=Escherichia coli (strain K12) OX=83333 GN=chaA PE=1 SV=1

```
0     MSNAQEAVKTRHKETSLIFPVLALVVLFLWGSSQTLPVVIAINLLALIGI

50    LSSAFSVVRHADVLAHRLGEPYGSLILSLSVVILEVSLISALMATGDAAP

100   TLMRDTLYSIIMIVTGGLVGFSLLLGGRKFATQYMNLFGIKQYLIALFPL
                                                         *
150   AIIVLVFPMALPAANFSTGQALLVALISAAMYGVFLLIQTKTHQSLFVYE
      *********************
200   HEDDSDDDDPHHGKPSAHSSLWHAIWLIIHLIAVIAVTKMNASSLETLLD

250   SMNAPVAFTGFLVALLILSPEGLGALKAVLNNQVQRAMNLFFGSVLATIS

300   LTVPVVTLIAFMTGNELQFALGAPEMVVMVASLVLCHISFSTGRTNVLNG

350   AAHLALFAAYLMTIFA
```

sp|P24202|MRR_ECOLI Mrr restriction system protein OS=Escherichia coli (strain K12) OX=83333 GN=mrr PE=1 SV=1

```
0     MTVPTYDKFIEPVLRYLATKPEGAAARDVHEAAADALGLDDSQRAKVITS

50    GQLVYKNRAGWAHDRLKRAGLSQSLSRGKWCLTPAGFDWVASHPQPMTEQ
                           ************************
100   ETNHLAFAFVNVKLKSRPDAVDLDPKADSPDHEELAKSSPDDRLDQALKE

150   LRDAVADEVLENLLQVSPSRFEVIVLDVLHRLGYGGHRDDLQRVGGTGDG

200   GIDGVISLDKLGLEKVYVQAKRWQNTVGRPELQAFYGALAGQKAKRGVFI

250   TTSGFTSQARDFAQSVEGMVLVDGERLVHLMIENEVGVSSRLLKVPKLDM

300   DYFE
```

sp|P0A6Z3|HTPG_ECOLI Chaperone protein HtpG OS=Escherichia coli (strain K12) OX=83333 GN=htpG PE=1 SV=1

```
0     MKGQETRGFQSEVKQLLHLMIHSLYSNKEIFLRELISNASDAADKLRFRA

50    LSNPDLYEGDGELRVRVSFDKDKRTLTISDNGVGMTRDEVIDHLGTIAKS

100   GTKSFLESLGSDQAKDSQLIGQFGVGFYSAFIVADKVTVRTRAAGEKPEN

150   GVFWESAGEGEYTVADITKEDRGTEITLHLREGEDEFLDDWRVRSIISKY

200   SDHIALPVEIEKREEKDGETVISWEKINKAQALWTRNKSEITDEEYKEFY

250   KHIAHDFNDPLTWSHNRVEGKQEYTSLLYIPSQAPWDMWNRDHKHGLKLY

300   VQRVFIMDDAEQFMPNYLRFVRGLIDSSDLPLNVSREILQDSTVTRNLRN

350   ALTKRVLQMLEKLAKDDAEKYQTFWQQFGLVLKEGPAEDFANQEAIAKLL

400   RFASTHTDSSAQTVSLEDYVSRMKEGQEKIYYITADSYAAAKSSPHLELL
                                       *********************
450   RKKGIEVLLLSDRIDEWMMNYLTEFDGKPFQSVSKVDESLEKLADEVDES
      ********
500   AKEAEKALTPFIDRVKALLGERVKDVRLTHRLTDTPAIVSTDADEMSTQM

550   AKLFAAAGQKVPEVKYIFELNPDHVLVKRAADTEDEAKFSEWVELLLDQA

600   LLAERGTLEDPNLFIRRMNQLLVS

sp|P0DMC9|RCSA_ECOLI Transcriptional regulatory protein RcsA
OS=Escherichia coli (strain K12) OX=83333 GN=rcsA PE=1 SV=1

0     MSTIIMDLCSYTRLGLTGYLLSRGVKKREINDIETVDDLAIACDSQRPSV

50    VFINEDCFIHDASNSQRIKLIINQHPNTLFIVFMAIANVHFDEYLLVRKN
         *********************
100   LLISSKSIKPESLDDILGDILKKETTITSFLNMPTLSLSRTESSMLRMWM

150   AGQGTIQISDQMNIKAKTVSSHKGNIKRKIKTHNKQVIYHVVRLTDNVTN

200   GIFVNMR

sp|P00550|PTM3C_ECOLI PTS system mannitol-specific EIICBA component
OS=Escherichia coli (strain K12) OX=83333 GN=mtlA PE=1 SV=1

0     MSSDIKIKVQSFGRFLSNMVMPNIGAFIAWGIITALFIPTGWLPNETLAK

50    LVGPMITYLLPLLIGYTGGKLVGGERGGVVGAITTMGVIVGADMPMFLGS

100   MIAGPLGGWCIKHFDRWVDGKIKSGFEMLVNNFSAGIIGMILAILAFLGI

150   GPIVEALSKMLAAGVNFMVVHDMLPLASIFVEPAKILFLNNAINHGIFSP

200   LGIQQSHELGKSIFFLIEANPGPGMGVLLAYMFFGRGSAKQSAGGAAIIH

250   FLGGIHEIYFPYVLMNPRLILAVILGGMTGVFTLTILGGGLVSPASPGSI
```

300 LAVLAMTPKGAYFANIAGVCAAMAVSFVVSAILLKTSKVKEEDDIEAATR

350 RMQDMKAESKGASPLSAGDVTNDLSHVRKIIVACDAGMGSSAMGAGVLRK

400 KIQDAGLSQISVTNSAINNLPPDVDLVITHRDLTERAMRQVPQAQHISLT
****

450 NFLDSGLYTSLTERLVAAQRHTANEEKVKDSLKDSFDDSSANLFKLGAEN

500 IFLGRKAATKEEAIRFAGEQLVKGGYVEPEYVQAMLDREKLTPTYLGESI

550 AVPHGTVEAKDRVLKTGVVFCQYPEGVRFGEEEDDIARLVIGIAARNNEH

600 IQVITSLTNALDDESVIERLAHTTSVDEVLELLAGRK

sp|Q2EET2|YPFN_ECOLI UPF0370 protein YpfN OS=Escherichia coli (strain K12) OX=83333 GN=ypfN PE=3 SV=1

*****
0 MDWLAKYWWILVIVFLVGVLLNVIKDLKRVDHKKFLANKPELPPHRDFND
****
50 KWDDDDDWPKKDQPKK

sp|P32128|YIHF_ECOLI Uncharacterized protein YihF OS=Escherichia coli (strain K12) OX=83333 GN=yihF PE=3 SV=3

0 MIRKSATGVIVALAVIWGGGTWYTGTQIQPGVEKFIKDFNDAKKKGEHAY

50 DMTLSYQNFDKGFFNSRFQMQMTFDNGAPDLNIKPGQKVVFDVDVEHGPL

100 PITMLMHGNVIPALAAAKVNLVNNELTQPLFIAAKNKSPVEATLRFAFGG

150 SFSTTLDVAPAEYGKFSFGEGQFTFNGDGSSLSNLDIEGKVEDIVLQLSP

200 MNKVTAKSFTIDSLARLEEKKFPVGESESKFNQINIINHGEDVAQIDAFV

250 AKTRLDRVKDKDYINVNLTYELDKLTKGNQQLGSGEWSLIAESIDPSAVR

300 QFIIQYNIAMQKQLAAHPELANDEVALQEVNAALFKEYLPLLQKSEPTIK
****
350 QPVRWKNALGELNANLDISIADPAKSSSSTNKDIKSLNFDVKLPLNVVTE

400 TAKQLNLSEGMDAEKAQKQADKQISGMMTLGQMFQLITIDNNTASLQLRY

450 TPGKVVFNGQEMSEEEFMSRAGRFVH

sp|P39295|YJFM_ECOLI Uncharacterized protein YjfM OS=Escherichia coli (strain K12) OX=83333 GN=yjfM PE=4 SV=1

0 MARKRKSRNNSKIGHGAISRIGRPNNPFEPCRNRYAQKYLTLALMGGAAF

50 FVLKGCSDSSDVDNDGDGTFYATVQDCIDDGNNADICARGWNNAKTAFYA

100 DVPKNMTQQNCQSKYENCYYDNVEQSWIPVVSGFLLSRVIRKDRDEPFVY
****

150 NSGGSSFASRPVWRSTSGDYSWRSGSGKKESYSSGGFTTKKASTVSRGGY

200 GRSSSARGHWGG

sp|P75882|GFCD_ECOLI Uncharacterized lipoprotein GfcD OS=Escherichia coli (strain K12) OX=83333 GN=gfcD PE=3 SV=1

0 MKKNSYLLSCLAIAVSSACHAEVLTYPDPLGSSQSDFGGTGLLQMPNARI
****
50 APEGEFSVNYRDNDQYRFYSTSVALFPWLEGTIRYTDVRTRKYSQWEDFS
****
100 GDQSYKDKSFDFKLRLWEEGYWLPQVAFGKRDIAGTGLFDGEYLVASKQA

150 GPFDFTLGMAWGYAGNAGNITNPFCRVSDKYCHRAESHDAGDISFSDIFR

200 GPASIFGGIEYQTPWNPLRLKLEYDGNNYQNDFAGKLPQASHFNVGAVYR

250 AASWADLNLSYERGNTLMFGFTLRTNFNDLRPALRDTPKPAYQPAPESEG

300 LQYTTVANQLTALKYNAGFDAPEIQLRDKTLYMSGQQYKYRDSREAVDRA

350 NRILVNNLPQGVEKISVTQKREHMAMVTTETDVASLRKQLAGTAPGQSEP

400 LQQQRVEAEDLSAFGRGYRIREDRFSYSFNPTLSQSLGGPEDFYMFQLGL

450 MSSARYWFTDHLLLDGGIFTNIYNNYDKFKSSLLPADSTLPRVRTHIRDY

500 VRNDVYLNNLQANYFADLGNGFYGQVYGGYLETMYAGVGSELLYRPLDAC

550 WALGVDVNYVKQRDWDNMMRFTDYSTPTGFVTAYWNPPTLNGVLMKLSVG

600 QYLAKDKGATIDVAKRFDSGVAVGVWAAISNVSKDDYGEGGFSKGFYISI

650 PFDLMTIGPNRNRAVVSWTPLTRDGGQMLSRKYQLYPMTAEREVPVGQ

sp|P69423|TATC_ECOLI Sec-independent protein translocase protein TatC OS=Escherichia coli (strain K12) OX=83333 GN=tatC PE=1 SV=2

0 MSVEDTQPLITHLIELRKRLLNCIIAVIVIFLCLVYFANDIYHLVSAPLI

50 KQLPQGSTMIATDVASPFFTPIKLTFMVSLILSAPVILYQVWAFIAPALY

100 KHERRLVVPLLVSSSLLFYIGMAFAYFVVFPLAFGFLANTAPEGVQVSTD

150 IASYLSFVMALFMAFGVSFEVPVAIVLLCWMGITSPEDLRKKRPYVLVGA
****
200 FVVGMLLTPPDVFSQTLLAIPMYCLFEIGVFFSRFYVGKGRNREEENDAE
****
250 AESEKTEE

sp|P76190|MEPH_ECOLI Murein DD-endopeptidase MepH OS=Escherichia coli (strain K12) OX=83333 GN=mepH PE=1 SV=1

0 MARINRISITLCALLFTTLPLTPMAHASKQARESSATTHITKKADKKKST

```
               ******************************
50    ATTKKTQKTAKKAASKSTTKSKTASSVKKSSITASKNAKTRSKHAVNKTA

100   SASFTEKCTKRKGYKSHCVKVKNAASGTLADAHKAKVQKATKVAMNKLMQ

150   QIGKPYRWGGSSPRTGFDCSGLVYYAYKDLVKIRIPRTANEMYHLRDAAP

200   IERSELKNGDLVFFRTQGRGTADHVGVYVGNGKFIQSPRTGQEIQITSLS

250   EDYWQRHYVGARRVMTPKTLR
```

sp|P76128|DDPA_ECOLI Probable D,D-dipeptide-binding periplasmic protein DdpA OS=Escherichia coli (strain K12) OX=83333 GN=ddpA PE=2 SV=1

```
0     MKRSISFRPTLLALVLATNFPVAHAAVPKDMLVIGKAADPQTLDPAVTID
                           ***********************
50    NNDWTVTYPSYQRLVQYKTDGDKGSTDVEGDLASSWKASDDQKEWTFTLK

100   DNAKFADGTPVTAEAVKLSFERLLKIGQGPAEAFPKDLKIDAPDEHTVKF

150   TLSQPFAPFLYTLANDGASIINPAVLKEHAADDARGFLAQNTAGSGPFML

200   KSWQKGQQLVLVPNPHYPGNKPNFKRVSVKIIGESASRRLQLSRGDIDIA

250   DALPVDQLNALKQENKVNVAEYPSLRVTYLYLNNSKAPLNQADLRRAISW

300   STDYQGMVNGILSGNGKQMRGPIPEGMWGYDATAMQYNHDETKAKAEWDK

350   VTSKPTSLTFLYSDNDPNWEPIALATQSSLNKLGIIVKLEKLANATMRDR

400   VGKGDYDIAIGNWSPDFADPYMFMNYWFESDKKGLPGNRSFYENSEVDKL

450   LRNALATTDQTQRTRDYQQAQKIVIDDAAYVYLFQKNYQLAMNKEVKGFV

500   FNPMLEQVFNINTMSK
```

sp|P0ABA6|ATPG_ECOLI ATP synthase gamma chain OS=Escherichia coli (strain K12) OX=83333 GN=atpG PE=1 SV=1

```
0     MAGAKEIRSKIASVQNTQKITKAMEMVAASKMRKSQDRMAASRPYAETMR

50    KVIGHLAHGNLEYKHPYLEDRDVKRVGYLVVSTDRGLCGGLNINLFKKLL

100   AEMKTWTDKGVQCDLAMIGSKGVSFFNSVGGNVVAQVTGMGDNPSLSELI
                                                 *******
150   GPVKVMLQAYDEGRLDKLYIVSNKFINTMSQVPTISQLLPLPASDDDDLK
      *************
200   HKSWDYLYEPDPKALLDTLLRRYVESQVYQGVVENLASEQAARMVAMKAA

250   TDNGGSLIKELQLVYNKARQASITQELTEIVSGAAAV
```

sp|P77609|FLXA_ECOLI Protein FlxA OS=Escherichia coli (strain K12) OX=83333 GN=flxA PE=1 SV=1

```
0     MSVTIQGNTSTVISNNSAPEGTSEIAKITRQIQVLTEKLGKISSEEGMTT
                                 ***********************
50    QQKKEMAALVQKQIESLWAQLEQLLRQQAEKKNEDATVQPDKKEEKKDDT
      *
100   NTAGTIDIYV
```

sp|P0AAG3|GLTL_ECOLI Glutamate/aspartate import ATP-binding protein GltL OS=Escherichia coli (strain K12) OX=83333 GN=gltL PE=3 SV=1

```
0     MITLKNVSKWYGHFQVLTDCSTEVKKGEVVVVCGPSGSGKSTLIKTVNGL

50    EPVQQGEITVDGIVVNDKKTDLAKLRSRVGMVFQHFELFPHLSIIENLTL

100   AQVKVLKRDKAPAREKALKLLERVGLSAHANKFPAQLSGGQQQRVAIARA

150   LCMDPIAMLFDEPTSALDPEMINEVLDVMVELANEGMTMMVVTHEMGFAR
               **************************
200   KVANRVIFMDEGKIVEDSPKDAFFDDPKSDRAKDFLAKILH
```

sp|P0ACS9|ACRR_ECOLI HTH-type transcriptional regulator AcrR OS=Escherichia coli (strain K12) OX=83333 GN=acrR PE=1 SV=1

```
0     MARKTKQEAQETRQHILDVALRLFSQQGVSSTSLGEIAKAAGVTRGAIYW
        ********************
50    HFKDKSDLFSEIWELSESNIGELELEYQAKFPGDPLSVLREILIHVLEST

100   VTEERRRLLMEIIFHKCEFVGEMAVVQQAQRNLCLESYDRIEQTLKHCIE

150   AKMLPADLMTRRAAIIMRGYISGLMENWLFAPQSFDLKKEARDYVAILLE

200   MYLLCPTLRNPATNE
```

sp|P0AES9|HDEA_ECOLI Acid stress chaperone HdeA OS=Escherichia coli (strain K12) OX=83333 GN=hdeA PE=1 SV=1

```
0     MKKVLGVILGGLLLLPVVSNAADAQKAADNKKPVNSWTCEDFLAVDESFQ
                                             ***********
50    PTAVGFAEALNNKDKPEDAVLDVQGIATVTPAIVQACTQDKQANFKDKVK
      *********
100   GEWDKIKKDM
```

sp|P37629|YHIL_ECOLI Putative uncharacterized protein YhiL OS=Escherichia coli (strain K12) OX=83333 GN=yhiL PE=5 SV=3

```
0     MKAIDNQIRNISSSHQDKHSDKVNSHQHHGKVDKTHRAKIVEFDKLDNDS

50    QIDNDFGLHIIYFLQHGHWKVNDRSHQMEKVWFYNSEPSIDIQEYNRFAD

100   NTTDTFIFTIIPDNNHVLKLSSPITVTVECKGGYYFINSSGDKSDIIYKV
                                ********************
150   DGLSIIARNFFTLLSGNFKPDWRWDVSKETFTKEKFDSYVKSVFSKIDFY

200   KQCGVINPQNANTAYFGDTDGRVGAVLYALLVSGHIGIREKGWSLLCELL
```

250 KHEEMASSAYKHKNNKVLYDLLNTRDMILNELHQHVFLKDDAITPCIFLG

300 DHTGDRFSTIFGDKYILTLLNSMRNMEGNKDSRINKNVVVLAGNHEINFN

350 GNYTARLANHKLSAGDTYNLIKTLDVCNYDSERQVLTSHHGIIRDEEKKC

400 YCLGALQVPFNQMKNPTDPEELANIFNKKHKEHMDDPLFHLIRSNTLKPT

450 PVYANYFDNTTDFRPARERIFICGETLKGEDPSKYIRQKYGHHGPGVDHN

500 QQFDNGIMGLNSLKEARDKNNKIIYSSGLSCFQLH

sp|P00895|TRPE_ECOLI Anthranilate synthase component 1 OS=Escherichia coli (strain K12) OX=83333 GN=trpE PE=1 SV=2

****
0 MQTQKPTLELLTCEGAYRDNPTALFHQLCGDRPATLLLESADIDSKDDLK
****
50 SLLLVDSALRITALGDTVTIQALSGNGEALLALLDNALPAGVESEQSPNC

100 RVLRFPPVSPLLDEDARLCSLSVFDAFRLLQNLLNVPKEEREAMFFGGLF

150 SYDLVAGFEDLPQLSAENNCPDFCFYLAETLMVIDHQKKSTRIQASLFAP

200 NEEEKQRLTARLNELRQQLTEAAPPLPVVSVPHMRCECNQSDEEFGGVVR

250 LLQKAIRAGEIFQVVPSRRFSLPCPSPLAAYYVLKKSNPSPYMFFMQDND

300 FTLFGASPESSLKYDATSRQIEIYPIAGTRPRGRRADGSLDRDLDSRIEL

350 EMRTDHKELSEHLMLVDLARNDLARICTPGSRYVADLTKVDRYSYVMHLV

400 SRVVGELRHDLDALHAYRACMNMGTLSGAPKVRAMQLIAEAEGRRRGSYG

450 GAVGYFTAHGDLDTCIVIRSALVENGIATVQAGAGVVLDSVPQSEADETR

500 NKARAVLRAIATAHHAQETF

sp|P23485|FECR_ECOLI Protein FecR OS=Escherichia coli (strain K12) OX=83333 GN=fecR PE=2 SV=1

0 MNPLLTDSRRQALRSASHWYAVLSGERVSPQQEARWQQWYEQDQDNQWAW

50 QQVENLRNQLGGVPGDVASRALHDTRLTRRHVMKGLLLLLGAGGGWQLWQ

100 SETGEGLRADYRTAKGTVSRQQLEDGSLLTLNTQSAADVRFDAHQRTVRL

150 WYGEIAITTAKDALQRPFRVLTRQGQLTALGTEFTVRQQDNFTQLDVQQH
****
200 AVEVLLASAPAQKRIVNAGESLQFSASEFGAVKPLDDESTSWTKDILSFS
**
250 DKPLGEVIATLTRYRNGVLRCDPAVAGLRLSGTFPLKNTDAILNVIAQTL

300 PVKIQSITRYWINISPL

sp|P0A8X0|YJGA_ECOLI UPF0307 protein YjgA OS=Escherichia coli (strain K12) OX=83333 GN=yjgA PE=1 SV=1

        ****

0 MTKQPEDWLDDVPGDDIEDEDDEIIWVSKSEIKRDAEELKRLGAEIVDLG

50 KNALDKIPLDADLRAAIELAQRIKMEGRRRQLQLIGKMLRQRDVEPIRQA

100 LDKLKNRHNQQVVLFHKLENLRDRLIDQGDDAIAEVLNLWPDADRQQLRT

150 LIRNAKKEKEGNKPPKSARQIFQYLRELAENEG

sp|P15032|RECE_ECOLI Exodeoxyribonuclease 8 OS=Escherichia coli (strain K12) OX=83333 GN=recE PE=1 SV=3

0 MSTKPLFLLRKAKKSSGEPDVVLWASNDFESTCATLDYLIVKSGKKLSSY

50 FKAVATNFPVVNDLPAEGEIDFTWSERYQLSKDSMTWELKPGAAPDNAHY

100 QGNTNVNGEDMTEIEENMLLPISGQELPIRWLAQHGSEKPVTHVSRDGLQ

150 ALHIARAEELPAVTALAVSHKTSLLDPLEIRELHKLVRDTDKVFPNPGNS

200 NLGLITAFFEAYLNADYTDRGLLTKEWMKGNRVSHITRTASGANAGGGNL

250 TDRGEGFVHDLTSLARDVATGVLARSMDLDIYNLHPAHAKRIEEIIAENK

300 PPFSVFRDKFITMPGGLDYSRAIVVASVKEAPIGIEVIPAHVTEYLNKVL

350 TETDHANPDPEIVDIACGRSSAPMPQRVTEEGKQDDEEKPQPSGTTAVEQ

400 GEAETMEPDATEHHQDTQPLDAQSQVNSVDAKYQELRAELHEARKNIPSK

450 NPVDDDKLLAASRGEFVDGISDPNDPKWVKGIQTRDCVYQNQPETEKTSP

****

500 DMNQPEPVVQQEPEIACNACGQTGGDNCPDCGAVMGDATYQETFDEESQV

****

550 EAKENDPEEMEGAEHPHNENAGSDPHRDCSDETGEVADPVIVEDIEPGIY

600 YGISNENYHAGPGISKSQLDDIADTPALYLWRKNAPVDTTKTKTLDLGTA

650 FHCRVLEPEEFSNRFIVAPEFNRRTNAGKEEEKAFLMECASTGKTVITAE

700 EGRKIELMYQSVMALPLGQWLVESAGHAESSIYWEDPETGILCRCRPDKI

750 IPEFHWIMDVKTTADIQRFKTAYYDYRYHVQDAFYSDGYEAQFGVQPTFV

800 FLVASTTIECGRYPVEIFMMGEEAKLAGQQEYHRNLRTLSDCLNTDEWPA

850 IKTLSLPRWAKEYAND

sp|P39352|YJHB_ECOLI Putative metabolite transport protein YjhB OS=Escherichia coli (strain K12) OX=83333 GN=yjhB PE=1 SV=2

0 MATAWYKQVNPPQRKALFSAWLGYVFDGFDFMMIFYILHIIKADLGITDI

```
50    QATLIGTVAFIARPIGGGFFGAMADKYGRKPMMMWAIFIYSVGTGLSGIA

100   TNLYMLAVCRFIVGLGMSGEYACASTYAVESWPKNLQSKASAFLVSGFSV
                                        ****************
150   GNIIAAQIIPQFAEVYGWRNSFFIGLLPVLLVLWIRKSAPESQEWIEDKY
      ******
200   KDKSTFLSVFRKPHLSISMIVFLVCFCLFGANWPINGLLPSYLADNGVNT

250   VVISTLMTIAGLGTLTGTIFFGFVGDKIGVKKAFVVGLITSFIFLCPLFF

300   ISVKNSSLIGLCLFGLMFTNLGIAGLVPKFIYDYFPTKLRGLGTGLIYNL

350   GATGGMAAPVLATYISGYYGLGVSLFIVTVAFSALLILLVGFDIPGKIYK

400   LSVAK
```

sp|P24218|INTD_ECOLI Prophage integrase IntD OS=Escherichia coli (strain K12) OX=83333 GN=intD PE=3 SV=1

```
0     MSLFRRNEIWYASYSLPGGKRIKESLGTKDKRQAQELHDKRKAELWRVEK
               ********************************
50    LGDLPDVTFEEACLRWLEEKADKKSLDSDKSRIEFWLEHFEGIRLKDISE

100   AKIYSAVSRMHNRKTKEIWKQKVQAAIRKGKELPVYEPKPVSTQTKAKHL

150   AMIKAILRAAERDWKWLEKAPVIKIPAVRNKRVRWLEKEEAKRLIDECPE

200   PLKSVVKFALATGLRKSNIINLEWQQIDMQRRVAWVNPEESKSNRAIGVA

250   LNDTACKVLRDQIGKHHKWVFVHTKAAKRADGTSTPAVRKMRIDSKTSWL

300   SACRRAGIEDFRFHDLRHTWASWLIQSGVPLSVLQEMGGWESIEMVRRYA

350   HLAPNHLTEHARKIDDIFGDNVPNMSHSEIMEDIKKA
```

sp|P37651|GUN_ECOLI Endoglucanase OS=Escherichia coli (strain K12) OX=83333 GN=bcsZ PE=1 SV=1

```
0     MNVLRSGIVTMLLLAAFSVQAACTWPAWEQFKKDYISQEGRVIDPSDARK
                                                      **
50    ITTSEGQSYGMFSALAANDRAAFDNILDWTQNNLAQGSLKERLPAWLWGK
      *******************
100   KENSKWEVLDSNSASDGDVWMAWSLLEAGRLWKEQRYTDIGSALLKRIAR

150   EEVVTVPGLGSMLLPGKVGFAEDNSWRFNPSYLPPTLAQYFTRFGAPWTT

200   LRETNQRLLLETAPKGFSPDWVRYEKDKGWQLKAEKTLISSYDAIRVYMW

250   VGMMPDSDPQKARMLNRFKPMATFTEKNGYPPEKVDVATGKAQGKGPVGF

300   SAAMLPFLQNRDAQAVQRQRVADNFPGSDAYYNYVLTLFGQGWDQHRFRF

350   STKGELLPDWGQECANSH
```

sp|P42641|OBG_ECOLI GTPase ObgE/CgtA OS=Escherichia coli (strain K12) OX=83333 GN=obgE PE=1 SV=1

```
0      MKFVDEASILVVAGDGGNGCVSFRREKYIPKGGPDGGDGGDGGDVWMEAD

50     ENLNTLIDYRFEKSFRAERGQNGASRDCTGKRGKDVTIKVPVGTRVIDQG

100    TGETMGDMTKHGQRLLVAKGGWHGLGNTRFKSSVNRTPRQKTNGTPGDKR

150    ELLLELMLLADVGMLGMPNAGKSTFIRAVSAAKPKVADYPFTTLVPSLGV

200    VRMDNEKSFVVADIPGLIEGAAEGAGLGIRFLKHLERCRVLLHLIDIDPI

250    DGTDPVENARIIISELEKYSQDLATKPRWLVFNKIDLLDKVEAEEKAKAI

300    AEALGWEDKYYLISAASGLGVKDLCWDVMTFIIENPVVQAEEAKQPEKVE
                          *********************
350    FMWDDYHRQQLEEIAEEDDEDWDDDWDEDDEEGVEFIYKR
```

sp|P0CK95|ACFD_ECOLI Putative lipoprotein AcfD homolog OS=Escherichia coli (strain K12) OX=83333 GN=yghJ PE=3 SV=1

```
0      MNKKFKYKKSLLAAILSATLLAGCDGGGSGSSSDTPPVDSGTGSLPEVKP

50     DPTPNPEPTPEPTPDPEPTPEPIPDPEPTPEPEPEPVPTKTGYLTLGGSQ
                                                       **
100    RVTGATCNGESSDGFTFKPGEDVTCVAGNTTIATFNTQSEAARSLRAVEK
       *******************
150    VSFSLEDAQELAGSDDKKSNAVSLVTSSNSCPANTEQVCLTFSSVIESKR

200    FDSLYKQIDLAPEEFKKLVNEEVENNAATDKAPSTHTSPVVPVTTPGTKP

250    DLNASFVSANAEQFYQYQPTEIILSEGRLVDSQGYGVAGVNYYTNSGRGV

300    TGENGEFSFSWGETISFGIDTFELGSVRGNKSTIALTELGDEVRGANIDQ

350    LIHRYSTTGQNNTRVVPDDVRKVFAEYPNVINEIINLSLSNGATLGEGEQ

400    VVNLPNEFIEQFNTGQAKEIDTAICAKTDGCNEARWFSLTTRNVNDGQIQ

450    GVINKLWGVDTNYKSVSKFHVFHDSTNFYGSTGNARGQAVVNISNAAFPI

500    LMARNDKNYWLAFGEKRAWDKNELAYITEAPSLVEPENVTRDTATFNLPF

550    ISLGQVGEGKLMVIGNPHYNSILRCPNGYSWNGGVNKDGQCTLNSDPDDM

600    KNFMENVLRYLSDDKWKPDAKASMTVGTNLDTVYFKRHGQVTGNSAAFDF

650    HPDFAGISVEHLSSYGDLDPQEMPLLILNGFEYVTQVGNDPYAIPLRADT

700    SKPKLTQQDVTDLIAYLNKGGSVLIMENVMSNLKEESASGFVRLLDAAGL

750    SMALNKSVVNNDPQGYPNRVRQQRATGIWVYERYPAVDGALPYTIDSKTG
```

800   EVKWKYQVENKPDDKPKLEVASWLEDVDGKQETRYAFIDEADHKTEDSLK

850   AAKEKIFAAFPGLKECTNPAYHYEVNCLEYRPGTGVPVTGGMYVPQYTQL

900   SLNADTAKAMVQAADLGTNIQRLYQHELYFRTNGRKGERLSSVDLERLYQ

950   NMSVWLWNDTSYRYEEGKNDELGFKTFTEFLNCYANDAYAGGTKCSADLK

1000 KSLVDNNMIYGDGSSKAGMMNPSYPLNYMEKPLTRLMLGRSWWDLNIKVD

1050 VEKYPGAVSEEGQNVTETISLYSNPTKWFAGNMQSTGLWAPAQKEVTIKS

1100 NANVPVTVTVALADDLTGREKHEVALNRPPRVTKTYSLDASGTVKFKVPY

1150 GGLIYIKGNSSTNESASFTFTGVVKAPFYKDGAWKNDLNSPAPLGELESD

1200 AFVYTTPKKNLNASNYTGGLEQFANDLDTFASSMNDFYGRDSEDGKHRMF

1250 TYKNLPGHKHRFTNDVQISIGDAHSGYPVMNSSFSPNSTTLPTTPLNDWL

1300 IWHEVGHNAAETPLTVPGATEVANNVLALYMQDRYLGKMNRVADDITVAP

1350 EYLEESNNQAWARGGAGDRLLMYAQLKEWAEKNFDIKKWYPDGTPLPEFY

1400 SEREGMKGWNLFQLMHRKARGDEVSNDKFGGKNYCAESNGNAADTLMLCA

1450 SWVAQTDLSEFFKKWNPGANAYQLPGASEMSFEGGVSQSAYNTLASLDLP

1500 KPEQGPETINQVTEHKMSAE

sp|P77718|THII_ECOLI tRNA sulfurtransferase OS=Escherichia coli (strain K12) OX=83333 GN=thiI PE=1 SV=1

0   MKFIIKLFPEITIKSQSVRLRFIKILTGNIRNVLKHYDETLAVVRHWDNI

50   EVRAKDENQRLAIRDALTRIPGIHHILEVEDVPFTDMHDIFEKALVQYRD

100   QLEGKTFCVRVKRRGKHDFSSIDVERYVGGGLNQHIESARVKLTNPDVTV

150   HLEVEDDRLLLIKGRYEGIGGFPIGTQEDVLSLISGGFDSGVSSYMLMRR

200   GCRVHYCFFNLGGAAHEIGVRQVAHYLWNRFGSSHRVRFVAINFEPVVGE

250   ILEKIDDGQMGVILKRMMVRAASKVAERYGVQALVTGEALGQVSSQTLTN
                                                    ****
300   LRLIDNVSDTLILRPLISYDKEHIINLARQIGTEDFARTMPEYCGVISKS
      ****
350   PTVKAVKSKIEAEEEKFDFSILDKVVEEANNVDIREIAQQTEQEVVEVET

400   VNGFGPNDVILDIRSIDEQEDKPLKVEGIDVVSLPFYKLSTKFGDLDQNK

450   TWLLWCERGVMSRLQALYLREQGFNNVKVYRP

sp|P75694|YAHO_ECOLI Uncharacterized protein YahO OS=Escherichia coli (strain K12) OX=83333 GN=yahO PE=3 SV=1

                                                 *****
0   MKIISKMLVGALALAVTNVYAAELMTKAEFEKVESQYEKIGDISTSNEMS
      ****
50   TADAKEDLIKKADEKGADVLVLTSGQTDNKIHGTANIYKKK

sp|P0A805|RRF_ECOLI Ribosome-recycling factor OS=Escherichia coli (strain K12) OX=83333 GN=frr PE=1 SV=1

0   MISDIRKDAEVRMDKCVEAFKTQISKIRTGRASPSLLDGIVVEYYGTPTP

50   LRQLASVTVEDSRTLKINVFDRSMSPAVEKAIMASDLGLNPNSAGSDIRV
                                          ****
100   PLPPLTEERRKDLTKIVRGEAEQARVAVRNVRRDANDKVKALLKDKEISE
      ****
150   DDDRRSQDDVQKLTDAAIKKIEAALADKEAELMQF

sp|P39172|ZNUA_ECOLI High-affinity zinc uptake system protein ZnuA OS=Escherichia coli (strain K12) OX=83333 GN=znuA PE=1 SV=4

0   MLHKKTLLFAALSAALWGGATQAADAAVVASLKPVGFIASAIADGVTETE

50   VLLPDGASEHDYSLRPSDVKRLQNADLVVWVGPEMEAFMQKPVSKLPGAK
                   ****
100   QVTIAQLEDVKPLLMKSIHGDDDDHDHAEKSDEDHHHGDFNMHLWLSPEI

150   ARATAVAIHGKLVELMPQSRAKLDANLKDFEAQLASTETQVGNELAPLKG

200   KGYFVFHDAYGYFEKQFGLTPLGHFTVNPEIQPGAQRLHEIRTQLVEQKA

250   TCVFAEPQFRPAVVESVARGTSVRMGTLDPLGTNIKLGKTSYSEFLSQLA

300   NQYASCLKGD

sp|P32106|YIBG_ECOLI Uncharacterized protein YibG OS=Escherichia coli (strain K12) OX=83333 GN=yibG PE=3 SV=1

                                            ****
0   MKACLLLFFYFSFICQLHGADVKIKQNESMMGSTAMTYDLSEEKLMKLKY
      ****
50   KSQHGDSEASFRLYQYYCFTKNNIYKQLRFLERSASQGNVTAQFNYGVFL

100   SDTNPTLSEYYNLNRAIYWMEFAVNNGNIDAKSKLQELKKLKRMDRRKNK

150   ENP

sp|P32140|SQUS_ECOLI Sulfoquinovose isomerase OS=Escherichia coli (strain K12) OX=83333 GN=yihS PE=1 SV=2

0   MKWFNTLSHNRWLEQETDRIFDFGKNSVVPTGFGWLGNKGQIKEEMGTHL

50   WITARMLHVYSVAAAMGRPGAYSLVDHGIKAMNGALRDKKYGGWYACVND
                                                   ***
100   EGVVDASKQGYQHFFALLGAASAVTTGHPEARKLLDYTIEIIEKYFWSEE

****
150 EQMCLESWDEAFSKTEEYRGGNANMHAVEAFLIVYDVTHDKKWLDRAIRV

200 ASVIIHDVARNNHYRVNEHFDTQWNPLPDYNKDNPAHRFRAFGGTPGHWI

250 EWGRLMLHIHAALEARCEQPPAWLLEDAKGLFNATVRDAWAPDGADGIVY

300 TVDWEGKPVVRERVRWPIVEAMGTAYALYTVTGDRQYETWYQTWWEYCIK

350 YLMDYENGSWWQELDADNKVTTKVWDGKQDIYHLLHCLVIPRIPLAPGMA

400 PAVAAGLLDINAK

sp|P0A6Y8|DNAK_ECOLI Chaperone protein DnaK OS=Escherichia coli (strain K12) OX=83333 GN=dnaK PE=1 SV=2

0 MGKIIGIDLGTTNSCVAIMDGTTPRVLENAEGDRTTPSIIAYTQDGETLV

50 GQPAKRQAVTNPQNTLFAIKRLIGRRFQDEEVQRDVSIMPFKIIAADNGD

100 AWVEVKGQKMAPPQISAEVLKKMKKTAEDYLGEPVTEAVITVPAYFNDAQ

150 RQATKDAGRIAGLEVKRIINEPTAAALAYGLDKGTGNRTIAVYDLGGGTF

200 DISIIEIDEVDGEKTFEVLATNGDTHLGGEDFDSRLINYLVEEFKKDQGI

250 DLRNDPLAMQRLKEAAEKAKIELSSAQQTDVNLPYITADATGPKHMNIKV

300 TRAKLESLVEDLVNRSIEPLKVALQDAGLSVSDIDDVILVGGQTRMPMVQ

350 KKVAEFFGKEPRKDVNPDEAVAIGAAVQGGVLTGDVKDVLLLDVTPLSLG

400 IETMGGVMTTLIAKNTTIPTKHSQVFSTAEDNQSAVTIHVLQGERKRAAD

450 NKSLGQFNLDGINPAPRGMPQIEVTFDIDADGILHVSAKDKNSGKEQKIT

500 IKASSGLNEDEIQKMVRDAEANAEADRKFEELVQTRNQGDHLLHSTRKQV

550 EEAGDKLPADDKTAIESALTALETALKGEDKAAIEAKMQELAQVSQKLME
****
600 IAQQQHAQQQTAGADASANNAKDDDVVDAEFEEVKDKK

sp|P19317|NARW_ECOLI Probable nitrate reductase molybdenum cofactor assembly chaperone NarW OS=Escherichia coli (strain K12) OX=83333 GN=narW PE=1 SV=1

0 MQILKVIGLLMEYPDELLWECKEDALALIRRDAPMLTDFTHNLLNAPLLD

50 KQAEWCEVFDRGRTTSLLLFEHVHAESRDRGQAMVDLLAEYEKVGLQLDC

100 RELPDYLPLYLEYLSVLPDDQAKEGLLNVAPILALLGGRLKQREAPWYAL
****
150 FDALLQLAGSSLSSDSVTKQVNSEERDDTRQALDAVWEEEQVKFIEDNAT

200   ACDSSPLNQYQRRFSQDVAPQYVDISAGGGK

sp|P76612|YPJB_ECOLI Protein YpjB OS=Escherichia coli (strain K12) OX=83333 GN=ypjB PE=4 SV=1

      ****
0   MESRNSYENKIDEISSLSESKEHPIDIQEKKDAFVNEFKGVLFDKNTRSS

50   ELLFNFYECCYKFLPRAQPQDKIDSYNSALQAFSIFCSSTLTHNNIGFDF

100   KLFPEVKLSGEHLETVFKYKNGDDVREIAKINITLQKEEGGLYNLRGLDF

150   KGCFFSGQNFSNYDIQYVNWGTSLFDVDTPCIFNAPAYNKSNEKSLKPVS

200   ENGLSGVLTDRNNKIKLITGVAPFDDILFMDDDFDDSSSEDDPVENSPVV

250   TSPVVSSSKSSFQ

sp|P21507|SRMB_ECOLI ATP-dependent RNA helicase SrmB OS=Escherichia coli (strain K12) OX=83333 GN=srmB PE=1 SV=1

0   MTVTTFSELELDESLLEALQDKGFTRPTAIQAAAIPPALDGRDVLGSAPT

50   GTGKTAAYLLPALQHLLDFPRKKSGPPRILILTPTRELAMQVSDHARELA

100   KHTHLDIATITGGVAYMNHAEVFSENQDIVVATTGRLLQYIKEENFDCRA

150   VETLILDEADRMLDMGFAQDIEHIAGETRWRKQTLLFSATLEGDAIQDFA

200   ERLLEDPVEVSANPSTRERKKIHQWYYRADDLEHKTALLVHLLKQPEATR

250   SIVFVRKRERVHELANWLREAGINNCYLEGEMVQGKRNEAIKRLTEGRVN

300   VLVATDVAARGIDIPDVSHVFNFDMPRSGDTYLHRIGRTARAGRKGTAIS
                                               ****
350   LVEAHDHLLLGKVGRYIEEPIKARVIDELRPKTRAPSEKQTGKPSKKVLA
      ****
400   KRAEKKKAKEKEKPRVKKRHRDTKNIGKRRKPSGTGVPPQTTEE

sp|P75733|CHIP_ECOLI Chitoporin OS=Escherichia coli (strain K12) OX=83333 GN=chiP PE=1 SV=1

0   MRTFSGKRSTLALAIAGVTAMSGFMAMPEARAEGFIDDSTLTGGIYYWQR

50   ERDRKDVTDGDKYKTNLSHSTWNANLDFQSGYAADMFGLDIAAFTAIEMA
               ****
100   ENGDSSHPNEIAFSKSNKAYDEDWSGDKSGISLYKAAAKFKYGPVWARAG

150   YIQPTGQTLLAPHWSFMPGTYQGAEAGANFDYGDAGALSFSYMWTNEYKA

200   PWHLEMDEFYQNDKTTKVDYLHSFGAKYDFKNNFVLEAAFGQAEGYIDQY

250   FAKASYKFDIAGSPLTTSYQFYGTRDKVDDRSVNDLYDGTAWLQALTFGY

300   RAADVVDLRLEGTWVKADGQQGYFLQRMTPTYASSNGRLDIWWDNRSDFN

```
350   ANGEKAVFFGAMYDLKNWNLPGFAIGASYVYAWDAKPATWQSNPDAYYDK

400   NRTIEESAYSLDAVYTIQDGRAKGTMFKLHFTEYDNHSDIPSWGGGYGNI

450   FQDERDVKFMVIAPFTIF
```

sp|P36767|RDGC_ECOLI Recombination-associated protein RdgC OS=Escherichia coli (strain K12) OX=83333 GN=rdgC PE=1 SV=1

```
0     MLWFKNLMVYRLSREISLRAEEMEKQLASMAFTPCGSQDMAKMGWVPPMG
                                             ***********
50    SHSDALTHVANGQIVICARKEEKILPSPVIKQALEAKIAKLEAEQARKLK
      ***********
100   KTEKDSLKDEVLHSLLPRAFSRFSQTMMWIDTVNGLIMVDCASAKKAEDT

150   LALLRKSLGSLPVVPLSMENPIELTLTEWVRSGSAAQGFQLLDEAELKSL

200   LEDGGVIRAKKQDLTSEEITNHIEAGKVVTKLALDWQQRIQFVMCDDGSL

250   KRLKFCDELRDQNEDIDREDFAQRFDADFILMTGELAALIQNLIEGLGGE

300   AQR
```

sp|P00579|RPOD_ECOLI RNA polymerase sigma factor RpoD OS=Escherichia coli (strain K12) OX=83333 GN=rpoD PE=1 SV=2

```
0     MEQNPQSQLKLLVTRGKEQGYLTYAEVNDHLPEDIVDSDQIEDIIQMIND

50    MGIQVMEEAPDADDLMLAENTADEDAAEAAAQVLSSVESEIGRTTDPVRM

100   YMREMGTVELLTREGEIDIAKRIEDGINQVQCSVAEYPEAITYLLEQYDR

150   VEAEEARLSDLITGFVDPNAEEDLAPTATHVGSELSQEDLDDDEDEDEED
      *********************
200   GDDDSADDDNSIDPELAREKFAELRAQYVVTRDTIKAKGRSHATAQEEIL

250   KLSEVFKQFRLVPKQFDYLVNSMRVMMDRVRTQERLIMKLCVEQCKMPKK

300   NFITLFTGNETSDTWFNAAIAMNKPWSEKLHDVSEEVHRALQKLQQIEEE

350   TGLTIEQVKDINRRMSIGEAKARRAKKEMVEANLRLVISIAKKYTNRGLQ

400   FLDLIQEGNIGLMKAVDKFEYRRGYKFSTYATWWIRQAITRSIADQARTI

450   RIPVHMIETINKLNRISRQMLQEMGREPTPEELAERMLMPEDKIRKVLKI

500   AKEPISMETPIGDDEDSHLGDFIEDTTLELPLDSATTESLRAATHDVLAG

550   LTAREAKVLRMRFGIDMNTDYTLEEVGKQFDVTRERIRQIEAKALRKLRH

600   PSRSEVLRSFLDD
```

sp|P10442|RNH2_ECOLI Ribonuclease HII OS=Escherichia coli (strain K12) OX=83333 GN=rnhB PE=1 SV=2

```
                                                    ******
0     MIEFVYPHTQLVAGVDEVGRGPLVGAVVTAAVILDPARPIAGLNDSKKLS
      **************
50    EKRRLALYEEIKEKALSWSLGRAEPHEIDELNILHATMLAMQRAVAGLHI

100   APEYVLIDGNRCPKLPMPAMAVVKGDSRVPEISAASILAKVTRDAEMAAL

150   DIVFPQYGFAQHKGYPTAFHLEKLAEHGATEHHRRSFGPVKRALGLAS
```

sp|P0AGC0|UHPT_ECOLI Hexose-6-phosphate:phosphate antiporter OS=Escherichia coli (strain K12) OX=83333 GN=uhpT PE=1 SV=1

```
0     MLAFLNQVRKPTLDLPLEVRRKMWFKPFMQSYLVVFIGYLTMYLIRKNFN

50    IAQNDMISTYGLSMTQLGMIGLGFSITYGVGKTLVSYYADGKNTKQFLPF

100   MLILSAICMLGFSASMGSGSVSLFLMIAFYALSGFFQSTGGSCSYSTITK

150   WTPRRKRGTFLGFWNISHNLGGAGAAGVALFGANYLFDGHVIGMFIFPSI
                           *****************************
200   IALIVGFIGLRYGSDSPESYGLGKAEELFGEEISEEDKETESTDMTKWQI

250   FVEYVLKNKVIWLLCFANIFLYVVRIGIDQWSTVYAFQELKLSKAVAIQG

300   FTLFEAGALVGTLLWGWLSDLANGRRGLVACIALALIIATLGVYQHASNE

350   YIYLASLFALGFLVFGPQLLIGVAAVGFVPKKAIGAADGIKGTFAYLIGD

400   SFAKLGLGMIADGTPVFGLTGWAGTFAALDIAAIGCICLMAIVAVMEERK

450   IRREKKIQQLTVA
```

sp|P0AAD8|TDCC_ECOLI Threonine/serine transporter TdcC OS=Escherichia coli (strain K12) OX=83333 GN=tdcC PE=1 SV=1

```
       ********************
0     MSTSDSIVSSQTKQSSWRKSDTTWTLGLFGTAIGAGVLFFPIRAGFGGLI

50    PILLMLVLAYPIAFYCHRALARLCLSGSNPSGNITETVEEHFGKTGGVVI

100   TFLYFFAICPLLWIYGVTITNTFMTFWENQLGFAPLNRGFVALFLLLLMA

150   FVIWFGKDLMVKVMSYLVWPFIASLVLISLSLIPYWNSAVIDQVDLGSLS

200   LTGHDGILITVWLGISIMVFSFNFSPIVSSFVVSKREEYEKDFGRDFTER

250   KCSQIISRASMLMVAVVMFFAFSCLFTLSPANMAEAKAQNIPVLSYLANH

300   FASMTGTKTTFAITLEYAASIIALVAIFKSFFGHYLGTLEGLNGLVLKFG

350   YKGDKTKVSLGKLNTISMIFIMGSTWVVAYANPNILDLIEAMGAPIIASL

400   LCLLPMYAIRKAPSLAKYRGRLDNVFVTVIGLLTILNIVYKLF
```

sp|P11864|YHAC_ECOLI Uncharacterized protein YhaC OS=Escherichia coli (strain K12) OX=83333 GN=yhaC PE=4 SV=3

```
0     MFPVSSIGNDISSDLVRRKMNDLPESPTGNNLEALAPGIEKLKQTSIEMV

50    TLLNTLQPGGKCIITGDFQKELAYLQNVILYNVSSLRLDFLGYNAQIIQR

100   SDNTCELTINEPLKNQEISTGNININCPLKDIYNEIRRLNVIFSCGTGDI

150   VDLSSLDLRNVDLDYYDFTDKHMANTILNPFKLNSTNFTNANMFQVNFVS

200   STQNATISWDYLLKITPVLISISDMYSEEKIKFVESCLNEPGDITEEQLK

250   IMRFAIIKSIPRATLTDKLENELTKEIYKSSSKIINCLNRIKLTEMKEFS
                                       *****************
300   SEKIYDYIDIIIEDYENTKENAYLVVPQINYTMDLNIEDSSSEELLSDNT
      **********
350   LEKDENSPDNGFEVGEYNTYEAYNSEKQYFTREDYTYDYDLLNAI
```

sp|P77698|YBCK_ECOLI Uncharacterized protein YbcK OS=Escherichia coli (strain K12) OX=83333 GN=ybcK PE=4 SV=1

```
0     MKKAIAYMRFSSPGQMSGDSLNRQRRLIAEWLKVNSDYYLDTITYEDLGL

50    SAFKGKHAQSGAFSEFLDAIEHGYILPGTTLLVESLDRLSREKVGEAIER
                                             ***********
100   LKLILNHGIDVITLCDNTVYNIDSLNEPYSLIKAILIAQRANEESEIKSS
      *************
150   RVKLSWKKKRQDALESGTIMTASCPRWLSLDDKRTAFVPDPDRVKTIELI

200   FKLRMERRSLNAIAKYLNDHAVKNFSGKESAWGPSVIEKLLANKALIGIC

250   VPSYRARGKGISEIAGYYPRVISDDLFYAVQEIRLAPFGISNSSKNPMLI

300   NLLRTVMKCEACGNTMIVHAVSGSLHGYYVCPMRRLHRCDRPSIKRDLVD

350   YNIINELLFNCSKIQPVENKKDANETLELKIIELQMKINNLIVALSVAPE

400   VTAIAEKIRLLDKELRRASVSLKTLKSKGVNSFSDFYAIDLTSKNGRELC

450   RTLAYKTFEKIIINTDNKTCDIYFMNGIVFKHYPLMKVISAQQAISALKY

500   MVDGEIYF
```

sp|P0A707|IF3_ECOLI Translation initiation factor IF-3 OS=Escherichia coli (strain K12) OX=83333 GN=infC PE=1 SV=1

```
0     MKGGKRVQTARPNRINGEIRAQEVRLTGLEGEQLGIVSLREALEKAEEAG
                      ********************************
50    VDLVEISPNAEPPVCRIMDYGKFLYEKSKSSKEQKKKQKVIQVKEIKFRP

100   GTDEGDYQVKLRSLIRFLEEGDKAKITLRFRGREMAHQQIGMEVLNRVKD
```

150   DLQELAVVESFPTKIEGRQMIMVLAPKKKQ

sp|P76083|PAAH_ECOLI 3-hydroxyadipyl-CoA dehydrogenase OS=Escherichia coli (strain K12) OX=83333 GN=paaH PE=1 SV=1

0   MMINVQTVAVIGSGTMGAGIAEVAASHGHQVLLYDISAEALTRAIDGIHA

50   RLNSRVTRGKLTAETCERTLKRLIPVTDIHALAAADLVIEAASERLEVKK

100   ALFAQLAEVCPPQTLLTTNTSSISITAIAAEIKNPERVAGLHFFNPAPVM

150   KLVEVVSGLATAAEVVEQLCELTLSWGKQPVRCHSTPGFIVNRVARPYYS

200   EAWRALEEQVAAPEVIDAALRDGAGFPMGPLELTDLIGQDVNFAVTCSVF
                                                      **
250   NAFWQERRFLPSLVQQELVIGGRLGKKSGLGVYDWRAEREAVVGLEAVSD
      ****
300   SFSPMKVEKKSDGVTEIDDVLLIETQGETAQALAIRLARPVVVIDKMAGK

350   VVTIAAAAVNPDSATRKAIYYLQQQGKTVLQIADYPGMLIWRTVAMIINE

400   ALDALQKGVASEQDIDTAMRLGVNYPYGPLAWGAQLGWQRILRLLENLQH

450   HYGEERYRPCSLLRQRALLESGYES

sp|P0ACY9|YEBG_ECOLI Uncharacterized protein YebG OS=Escherichia coli (strain K12) OX=83333 GN=yebG PE=4 SV=1
                ****
0   MAVEVKYVVIREGEEKMSFTSKKEADAYDKMLDTADLLDTWLTNSPVQME

50   DEQREALSLWLAEQKDVLSTILKTGKLPSPQVVGAESEEEDASHAA

sp|P03825|GSPB_ECOLI Putative general secretion pathway protein B OS=Escherichia coli (strain K12) OX=83333 GN=gspB PE=2 SV=2

0   MFEFYIAAREQKETGHPGIFSRQKHSTIIYVICLLLICLWFAGMVLVGGY

50   ARQLWVLWIVKAEVTVEAETPAFKQSTQHYFFKKQPLPVVESVEEEDDPG
         ****
100   VAVENAPSSSEDEENTVEESEEKAGLRERVKNALNELER

sp|P23865|PRC_ECOLI Tail-specific protease OS=Escherichia coli (strain K12) OX=83333 GN=prc PE=1 SV=2

0   MNMFFRLTALAGLLAIAGQTFAVEDITRADQIPVLKEETQHATVSERVTS

50   RFTRSHYRQFDLDQAFSAKIFDRYLNLLDYSHNVLLASDVEQFAKKKTEL

100   GDELRSGKLDVFYDLYNLAQKRRFERYQYALSVLEKPMDFTGNDTYNLDR
                      ****
150   SKAPWPKNEAELNALWDSKVKFDELSLKLTGKTDKEIRETLTRRYKFAIR

200   RLAQTNSEDVFSLAMTAFAREIDPHTNYLSPRNTEQFNTEMSLSLEGIGA

250   VLQMDDDYTVINSMVAGGPAAKSKAISVGDKIVGVGQTGKPMVDVIGWRL

300   DDVVALIKGPKGSKVRLEILPAGKGTKTRTVTLTRERIRLEDRAVKMSVK

350   TVGKEKVGVLDIPGFYVGLTDDVKVQLQKLEKQNVSSVIIDLRSNGGGAL

400   TEAVSLSGLFIPAGPIVQVRDNNGKVREDSDTDGQVFYKGPLVVLVDRFS

450   ASASEIFAAAMQDYGRALVVGEPTFGKGTVQQYRSLNRIYDQMLRPEWPA

500   LGSVQYTIQKFYRVNGGSTQRKGVTPDIIMPTGNEETETGEKFEDNALPW

550   DSIDAATYVKSGDLTAFEPELLKEHNARIAKDPEFQNIMKDIARFNAMKD

600   KRNIVSLNYAVREKENNEDDATRLARLNERFKREGKPELKKLDDLPKDYQ

650   EPDPYLDETVNIALDLAKLEKARPAEQPAPVK

sp|P0AFR4|YCIO_ECOLI Uncharacterized protein YciO OS=Escherichia coli (strain K12) OX=83333 GN=yciO PE=1 SV=1

0   MSQFFYIHPDNPQQRLINQAVEIVRKGGVIVYPTDSGYALGCKIEDKNAM

50   ERICRIRQLPDGHNFTLMCRDLSELSTYSFVDNVAFRLMKNNTPGNYTFI
                                                       *
100   LKGTKEVPRRLLQEKRKTIGMRVPSNPIAQALLEALGEPMLSTSLMLPGS
      ****
150   EFTESDPEEIKDRLEKQVDLIIHGGYLGQKPTTVIDLTDDTPVVVREGVG

200   DVKPFL

sp|P0AGC5|MLTF_ECOLI Membrane-bound lytic murein transglycosylase F OS=Escherichia coli (strain K12) OX=83333 GN=mltF PE=1 SV=2

0   MKKLKINYLFIGILALLLAVALWPSIPWFGKADNRIAAIQARGELRVSTI

50   HTPLTYNEINGKPFGLDYELAKQFADYLGVKLKVTVRQNISQLFDDLDNG

100   NADLLAAGLVYNSERVKNYQPGPTYYSVSQQLVYKVGQYRPRTLGNLTAE
                       ****
150   QLTVAPGHVVVNDLQTLKETKFPELSWKVDDKKGSAELMEDVIEGKLDYT

200   IADSVAISLFQRVHPELAVALDITDEQPVTWFSPLDGDNTLSAALLDFFN

250   EMNEDGTLARIEEKYLGHGDDFDYVDTRTFLRAVDAVLPQLKPLFEKYAE

300   EIDWRLLAAIAYQESHWDAQATSPTGVRGMMMLTKNTAQSLGITDRTDAE

350   QSISGGVRYLQDMMSKVPESVPENERIWFALAAYNMGYAHMLDARALTAK

400   TKGNPDSWADVKQRLPLLSQKPYYSKLTYGYARGHEAYAYVENIRKYQIS

450   LVGYLQEKEKQATEAAMQLAQDYPAVSPTELGKEKFPFLSFLSQSSSNYL

```
500   THSPSLLFSRKGSEEKQN

sp|P08957|T1MK_ECOLI Type I restriction enzyme EcoKI M protein
OS=Escherichia coli (strain K12) OX=83333 GN=hsdM PE=1 SV=1

0     MNNNDLVAKLWKLCDNLRDGGVSYQNYVNELASLLFLKMCKETGQEAEYL

50    PEGYRWDDLKSRIGQEQLQFYRKMLVHLGEDDKKLVQAVFHNVSTTITEP

100   KQITALVSNMDSLDWYNGAHGKSRDDFGDMYEGLLQKNANETKSGAGQYF

150   TPRPLIKTIIHLLKPQPREVVQDPAAGTAGFLIEADRYVKSQTNDLDDLD

200   GDTQDFQIHRAFIGLELVPGTRRLALMNCLLHDIEGNLDHGGAIRLGNTL

250   GSDGENLPKAHIVATNPPFGSAAGTNITRTFVHPTSNKQLCFMQHIIETL

300   HPGGRAAVVVPDNVLFEGGKGTDIRRDLMDKCHLHTILRLPTGIFYAQGV

350   KTNVLFFTKGTVANPNQDKNCTDDVWVYDLRTNMPSFGKRTPFTDEHLQP

400   FERVYGEDPHGLSPRTEGEWSFNAEETEVADSEENKNTDQHLATSRWRKF
           ****************************
450   SREWIRTAKSDSLDISWLKDKDSIDADSLPEPDVLAAEAMGELVQALSEL

500   DALMRELGASDEADLQRQLLEEAFGGVKE

sp|P0AGJ5|YFIF_ECOLI Uncharacterized tRNA/rRNA methyltransferase YfiF
OS=Escherichia coli (strain K12) OX=83333 GN=yfiF PE=1 SV=1
           ********************
0     MNDEMKGKSGKVKVMYVRSDDDSDKRTHNPRTGKGGGRPGKSRADGGRRP

50    ARDDKQSQPRDRKWEDSPWRTVSRAPGDETPEKADHGGISGKSFIDPEVL

100   RRQRAEETRVYGENACQALFQSRPEAIVRAWFIQSVTPRFKEALRWMAAN

150   RKAYHVVDEAELTKASGTEHHGGVCFLIKKRNGTTVQQWVSQAGAQDCVL

200   ALENESNPHNLGGMMRSCAHFGVKGVVVQDAALLESGAAIRTAEGGAEHV

250   QPITGDNIVNVLDDFRQAGYTVVTTSSEQGKPLFKTSLPAKMVLVLGQEY

300   EGLPDAARDPNDLRVKIDGTGNVAGLNISVATGVLLGEWWRQNKA

sp|P04949|FLIC_ECOLI Flagellin OS=Escherichia coli (strain K12) OX=83333
GN=fliC PE=1 SV=2

0     MAQVINTNSLSLITQNNINKNQSALSSSIERLSSGLRINSAKDDAAGQAI

50    ANRFTSNIKGLTQAARNANDGISVAQTTEGALSEINNNLQRVRELTVQAT
         **********************
100   TGTNSESDLSSIQDEIKSRLDEIDRVSGQTQFNGVNVLAKNGSMKIQVGA

150   NDNQTITIDLKQIDAKTLGLDGFSVKNNDTVTTSAPVTAFGATTTNNIKL
```

200   TGITLSTEAATDTGGTNPASIEGVYTDNGNDYYAKITGGDNDGKYYAVTV

250   ANDGTVTMATGATANATVTDANTTKATTITSGGTPVQIDNTAGSATANLG

300   AVSLVKLQDSKGNDTDTYALKDTNGNLYAADVNETTGAVSVKTITYTDSS

350   GAASSPTAVKLGGDDGKTEVVDIDGKTYDSADLNGGNLQTGLTAGGEALT

400   AVANGKTTDPLKALDDAIASVDKFRSSLGAVQNRLDSAVTNLNNTTTNLS

450   EAQSRIQDADYATEVSNMSKAQIIQQAGNSVLAKANQVPQQVLSLLQG

sp|P32135|YIHN_ECOLI Inner membrane protein YihN OS=Escherichia coli (strain K12) OX=83333 GN=yihN PE=1 SV=1

0   MLTKKKWALFSLLTLCGGTIYKLPSLKDAFYIPMQEYFHLTNGQIGNAMS

50   VNSFVTTVGFFLSIYFADKLPRRYTMSFSLIATGLLGVYLTTMPGYWGIL

100   FVWALFGVTCDMMNWPVLLKSVSRLGNSEQQGRLFGFFETGRGIVDTVVA
****
150   FSALAVFTWFGSGLLGFKAGIWFYSLIVIAVGIIIFFVLNDKEEAPSVEV
****
200   KKEDGASKNTSMTSVLKDKTIWLIAFNVFFVYAVYCGLTFFIPFLKNIYL

250   LPVALVGAYGIINQYCLKMIGGPIGGMISDKILKSPSKYLCYTFIISTAA

300   LVLLIMLPHESMPVYLGMACTLGFGAIVFTQRAVFFAPIGEAKIAENKTG

350   AAMALGSFIGYAPAMFCFSLYGYILDLNPGIIGYKIVFGIMACFAFSGAV

400   VSVMLVKRISQRKKEMLAAEA

sp|Q2EES0|YNFO_ECOLI Uncharacterized protein YnfO OS=Escherichia coli (strain K12) OX=83333 GN=ynfO PE=4 SV=1
**
0   MSTKNRTRRTTTRNIRFPNQMIEQINIALEQKGSGNFSAWVIEACRRRLC
****
50   SEKRVSSEANKEKSDITELLRKQVRPD

sp|P37645|YHJG_ECOLI AsmA family protein YhjG OS=Escherichia coli (strain K12) OX=83333 GN=yhjG PE=3 SV=3

0   MSKAGKITAAISGAFLLLIVVAIILIATFDWNRLKPTINQKVSAELNRPF

50   AIRGDLGVVWERQKQETGWRSWVPWPHVHAEDIILGNPPDIPEVTMVHLP

100   RVEATLAPLALLTKTVWLPWIKLEKPDARLIRLSEKNNNWTFNLANDDNK

150   DANAKPSAWSFRLDNILFDQGRIAIDDKVSKADLEIFVDPLGKPLPFSEV

200   TGSKGKADKEKVGDYVFGLKAQGRYNGEPLTGTGKIGGMLALRGEGTPFP

250   VQADFRSGNTRVAFDGVVNDPMKMGGVDLRLKFSGDSLGDLYELTGVLLP

300   DTPPFETDGRLVAKIDTEKSSVFDYRGFNGRIGDSDIHGSLVYTTGKPRP
                            ****
350   KLEGDVESRQLRLADLGPLIGVDSGKGAEKSKRSEQKKGEKSVQPAGKVL

400   PYDRFETDKWDVMDADVRFKGRRIEHGSSLPISDLSTHIILKNADLRLQP

450   LKFGMAGGSIAANIHLEGDKKPMQGRADIQARRLKLKELMPDVELMQKTL

500   GEMNGDAELRGSGNSVAALLGNSNGNLKLLMNDGLVSRNLMEIVGLNVGN

550   YIVGAIFGDDEVRVNCAAANLNIANGVARPQIFAFDTENALINVTGTASF

600   ASEQLDLTIDPESKGIRIITLRSPLYVRGTFKNPQAGVKAGPLIARGAVA

650   AALATLVTPAAALLALISPSEGEANQCRTILSQMKK

sp|P0A9R4|FER_ECOLI 2Fe-2S ferredoxin OS=Escherichia coli (strain K12) OX=83333 GN=fdx PE=1 SV=2

0   MPKIVILPHQDLCPDGAVLEANSGETILDAALRNGIEIEHACEKSCACTT
           ****
50   CHCIVREGFDSLPESSEQEDDMLDKAWGLEPESRLSCQARVTDEDLVVEI

100   PRYTINHAREH

sp|P16917|RHSB_ECOLI Protein RhsB OS=Escherichia coli (strain K12) OX=83333 GN=rhsB PE=3 SV=4

0   MSGKPAARQGDMTQYGGSIVQGSAGVRIGAPTGVACSVCPGGVTSGHPVN

50   PLLGAKVLPGETDIALPGPLPFILSRTYSSYRTKTPAPVGSLGPGWKMPA

100   DIRLQLRDNTLILSDNGGRSLYFEHLFPGEDGYSRSESLWLVRGGVAKLD

150   EGHRLAALWQALPEELRLSPHRYLATNSPQGPWWLLGWCERVPEADEVLP

200   APLPPYRVLTGLVDRFGRTQTFHREAAGEFSGEITGVTDGAWRHFRLVLT

250   TQAQRAEEARQQAISGGTEPSAFPDTLPGYTEYGRDNGIRLSAVWLTHDP

300   EYPENLPAAPLVRYGWTPRGELAVVYDRSGKQVRSFTYDDKYRGRMVAHR

350   HTGRPEIRYRYDSDGRVTEQLNPAGLSYTYQYEKDRITITDSLDRREVLH

400   TQGEAGLKRVVKKEHADGSVTQSQFDAVGRLRAQTDAAGRTTEYSPDVVT

450   GLITRITTPDGRASAFYYNHHNQLTSATGPDGLELRREYDELGRLIQETA

500   PDGDITRYRYDNPHSDLPCATEDATGSRKTMTWSRYGQLLSFTDCSGYVT

550   RYDHDRFGQMTAVHREEGLSQYRAYDSRGQLIAVKDTQGHETRYEYNIAG

600 DLTAVIAPDGSRNGTQYDAWGKAVRTTQGGLTRSMEYDAAGRVIRLTSEN

650 GSHTTFRYDVLDRLIQETGFDGRTQRYHHDLTGKLIRSEDEGLVTHWHYD

700 EADRLTHRTVKGETAERWQYDERGWLTDISHISEGHRVAVHYRYDEKGRL

750 TGERQTVHHPQTEALLWQHETRHAYNAQGLANRCIPDSLPAVEWLTYGSG

800 YLAGMKLGDTPLVEYTRDRLHRETLRSFGRYELTTAYTPAGQLQSQHLNS

850 LLSDRDYTWNDNGELIRISSPRQTRSYSYSTTGRLTGVHTTAANLDIRIP

900 YATDPAGNRLPDPELHPDSTLSMWPDNRIARDAHYLYRYDRHGRLTEKTD

950 LIPEGVIRTDDERTHRYHYDSQHRLVHYTRTQYEEPLVESRYLYDPLGRR

1000 VAKRVWRRERDLTGWMSLSRKPQVTWYGWDGDRLTTIQNDRSRIQTIYQP

1050 GSFTPLIRVETATGELAKTQRRSLADALQQSGGEDGGSVVFPPVLVQMLD

1100 RLESEILADRVSEESRRWLASCGLTVEQMQNQMDPVYTPARKIHLYHCDH

1150 RGLPLALISTEGATAWCAEYDEWGNLLNEENPHQLQQLIRLPGQQYDEES

1200 GLYYNRHRYYDPLQGRYITQDPIGLKGGWNLYGYQLNPISDIDPLGLSMW

1250 EDAKSGACTNGLCGTLSAMIGPDKFDSIDSTAYDALNKINSQSICEDKEF

1300 AGLICKDNSGRYFSTAPNRGERKGSYPFNSPCPNGTEKVSAYHTHGADSH
****

1350 GEYWDEIFSGKDEKIVKSKDNNIKSFYLGTPSGNFKAIDNHGKEITNRKG

1400 LPNVCRVHGNM

sp|P46889|FTSK_ECOLI DNA translocase FtsK OS=Escherichia coli (strain K12) OX=83333 GN=ftsK PE=1 SV=2

0 MSQEYIEDKEVTLTKLSSGRRLLEALLILIVLFAVWLMAALLSFNPSDPS

50 WSQTAWHEPIHNLGGMPGAWLADTLFFIFGVMAYTIPVIIVGGCWFAWRH

100 QSSDEYIDYFAVSLRIIGVLALILTSCGLAAINADDIWYFASGGVIGSLL

150 STTLQPLLHSSGGTIALLCVWAAGLTLFTGWSWVTIAEKLGGWILNILTF
****

200 ASNRTRRDDTWVDEDEYEDDEEYEDENHGKQHESRRARILRGALARRKRL

250 AEKFINPMGRQTDAALFSGKRMDDDEEITYTARGVAADPDDVLFSGNRAT

300 QPEYDEYDPLLNGAPITEPVAVAAAATTATQSWAAPVEPVTQTPPVASVD

350 VPPAQPTVAWQPVPGPQTGEPVIAPAPEGYPQQSQYAQPAVQYNEPLQQP

400 VQPQQPYYAPAAEQPAQQPYYAPAPEQPVAGNAWQAEEQQSTFAPQSTYQ

450   TEQTYQQPAAQEPLYQQPQPVEQQPVVEPEPVVEETKPARPPLYYFEEVE

500   EKRAREREQLAAWYQPIPEPVKEPEPIKSSLKAPSVAAVPPVEAAAAVSP

550   LASGVKKATLATGAAATVAAPVFSLANSGGPRPQVKEGIGPQLPRPKRIR

600   VPTRRELASYGIKLPSQRAAEEKAREAQRNQYDSGDQYNDDEIDAMQQDE

650   LARQFAQTQQQRYGEQYQHDVPVNAEDADAAAEAELARQFAQTQQQRYSG

700   EQPAGANPFSLDDFEFSPMKALLDDGPHEPLFTPIVEPVQQPQQPVAPQQ

750   QYQQPQQPVPPQPQYQQPQQPVAPQPQYQQPQQPVAPQQQYQQPQQPVAP

800   QQQYQQPQQPVAPQPQDTLLHPLLMRNGDSRPLHKPTTPLPSLDLLTPPP

850   SEVEPVDTFALEQMARLVEARLADFRIKADVVNYSPGPVITRFELNLAPG

900   VKAARISNLSRDLARSLSTVAVRVVEVIPGKPYVGLELPNKKRQTVYLRE

950   VLDNAKFRDNPSPLTVVLGKDIAGEPVVADLAKMPHLLVAGTTGSGKSVG

1000 VNAMILSMLYKAQPEDVRFIMIDPKMLELSVYEGIPHLLTEVVTDMKDAA

1050 NALRWCVNEMERRYKLMSALGVRNLAGYNEKIAEADRMMRPIPDPYWKPG

1100 DSMDAQHPVLKKEPYIVVLVDEFADLMMTVGKKVEELIARLAQKARAAGI

1150 HLVLATQRPSVDVITGLIKANIPTRIAFTVSSKIDSRTILDQAGAESLLG

1200 MGDMLYSGPNSTLPVRVHGAFVRDQEVHAVVQDWKARGRPQYVDGITSDS

1250 ESEGGAGGFDGAEELDPLFDQAVQFVTEKRKASISGVQRQFRIGYNRAAR

1300 IIEQMEAQGIVSEQGHNGNREVLAPPPFD

sp|P0ABS1|DKSA_ECOLI RNA polymerase-binding transcription factor DksA
OS=Escherichia coli (strain K12) OX=83333 GN=dksA PE=1 SV=1

0   MQEGQNRKTSSLSILAIAGVEPYQEKPGEEYMNEAQLAHFRRILEAWRNQ
                                                 ****
50   LRDEVDRTVTHMQDEAANFPDPVDRAAQEEEFSLELRNRDRERKLIKKIE
      ****
100   KTLKKVEDEDFGYCESCGVEIGIRRLEARPTADLCIDCKTLAEIREKQMA

150   G

sp|P77196|YFCU_ECOLI Putative outer membrane usher protein YfcU
OS=Escherichia coli (strain K12) OX=83333 GN=yfcU PE=5 SV=3

0   MPDHSLFRLRILPWCIALAMSGSYSSVWAEDDIQFDSRFLELKGDTKIDL

50   KRFSSQGYVEPGKYNLQVQLNKQPLAEEYDIYWYAGEDDVSKSYACLTPE

```
100   LVAQFGLKEDVAKNLQWSHDGKCLKPGQLEGVEIKADLSQSALVISLPQA

150   YLEYTWPDWDPPSRWDDGISGIIADYSITAQTRHEENGGDDSNEISGNGT
                            ********************
200   VGVNLGPWRMRADWQTNYQHTRSNDDDDEFGGDDTQKKWEWSRYYAWRAL

250   PSLKAKLALGEDYLNSDIFDGFNYVGGSVSTDDQMLPPNLRGYAPDISGV

300   AHTTAKVTVSQMGRVIYETQVPAGPFRIQDLGDSVSGTLHIRIEEQNGQV

350   QEYDISTASMPYLTRPGQVRYKIMMGRPQEWGHHVEGGFFSGAEASWGIA

400   NGWSLYGGALGDENYQSAALGVGRDLSTFGAVAFDVTHSHTKLDKDTAYG

450   KGSLDGNSFRVSYSKDFDQLNSRVTFAGYRFSEENFMTMSEYLDASDSEM

500   VRTGNDKEMYTATYNQNFRDAGVSVYLNYTRHTYWDREEQTNYNIMLSHY

550   FNMGSIRNMSVSLTGYRYEYDNRADKGMYISLSMPWGDNSTVSYNGNYGS

600   GTDSSQVGYFSRVDDATHYQLNIGTSDKHTSVDGYYSHDGSLAQVDLSAN

650   YHEGQYTSAGLSLQGGATLTTHGGALHRTQNMGGTRLLIDADGVADVPVE

700   GNGAAVYTNMFGKAVVSDVNNYYRNQAYIDLNKLPENAEATQSVVQATLT

750   EGAIGYRKFAVISGQKAMAVLRLQDGSHPPFGAEVKNDNEQTVGLVDDDG

800   SVYLAGVKPGEHMSVFWSGVAHCDINLPDPLPADLFNGLLLPCQHKGNVA

850   PVVPDDIKPVIQEQTQQVTPTDPPVSVSANQ

sp|P32134|YIHM_ECOLI Uncharacterized protein YihM OS=Escherichia coli
(strain K12) OX=83333 GN=yihM PE=4 SV=1

0     MVTINNARKILQRVDTLPLYLHAYAFHLNMRLERVLPADLLDIASENNLR

50    GVKIHVLDGERFSLGNMDDKELSAFGDKARRLNLDIHIETSASDKASIDE

100   AVAIALKTGASSVRFYPRYEGNLRDVLSIIANDIAYVRETYQDSGLTFTI
        **********************
150   EQHEDLKSHELVSLVKESEMESLSLLFDFANMINANEHPIDALKTMAPHI

200   TQVHIKDALIVKEPGGLGHKACISGQGDMPFKALLTHLICLGDDEPQVTA

250   YGLEEEVDYYAPAFRFEDEDDNPWIPYRQMSETPLPENHLLDARLRKEKE

300   DAINQINHVRNVLQQIKQEANHLLNH

sp|P77173|ZIPA_ECOLI Cell division protein ZipA OS=Escherichia coli
(strain K12) OX=83333 GN=zipA PE=1 SV=3
                                          **************
0     MMQDLRLILIIVGAIAIIALLVHGFWTSRKERSSMFRDRPLKRMKSKRDD
```

```
      ****************
50    DSYDEDVEDDEGVGEVRVHRVNHAPANAQEHEAARPSPQHQYQPPYASAQ

100   PRQPVQQPPEAQVPPQHAPHPAQPVQQPAYQPQPEQPLQQPVSPQVAPAP

150   QPVHSAPQPAQQAFQPAEPVAAPQPEPVAEPAPVMDKPKRKEAVIIMNVA

200   AHHGSELNGELLLNSIQQAGFIFGDMNIYHRHLSPDGSGPALFSLANMVK

250   PGTFDPEMKDFTTPGVTIFMQVPSYGDELQNFKLMLQSAQHIADEVGGVV

300   LDDQRRMMTPQKLREYQDIIREVKDANA
```

PASK RATIO 0.5

sp|P0A823|SFSA_ECOLI Sugar fermentation stimulation protein A OS=Escherichia coli (strain K12) OX=83333 GN=sfsA PE=1 SV=1

```
0     MEFSPPLQRATLIQRYKRFLADVITPDGRELTLHCPNTGAMTGCATPGDT
                                                ************
50    VWYSTSDNTKRKYPHTWELTQSQSGAFICVNTLWANRLTKEAILNESISE
      *********************
100   LSGYSSLKSEVKYGAERSRIDFMLQADSRPDCYIEVKSVTLAENEQGYFP

150   DAVTERGQKHLRELMSVAAEGQRAVIFFAVLHSAITRFSPARHIDEKYAQ

200   LLSEAQQRGVEILAYKAEISAEGMALKKSLPVTL
```

sp|P37689|GPMI_ECOLI 2,3-bisphosphoglycerate-independent phosphoglycerate mutase OS=Escherichia coli (strain K12) OX=83333 GN=gpmI PE=1 SV=1

```
0     MLVSKKPMVLVILDGYGYREEQQDNAIFSAKTPVMDALWANRPHTLIDAS

50    GLEVGLPDRQMGNSEVGHVNLGAGRIVYQDLTRLDVEIKDRAFFANPVLT

100   GAVDKAKNAGKAVHIMGLLSAGGVHSHEDHIMAMVELAAERGAEKIYLHA
       *****************************
150   FLDGRDTPPRSAESSLKKFEEKFAALGKGRVASIIGRYYAMDRDNRWDRV

200   EKAYDLLTLAQGEFQADTAVAGLQAAYARDENDEFVKATVIRAEGQPDAA

250   MEDGDALIFMNFRADRAREITRAFVNADFDGFARKKVVNVDFVMLTEYAA

300   DIKTAVAYPPASLVNTFGEWMAKNDKTQLRISETEKYAHVTFFFNGGVEE

350   SFKGEDRILINSPKVATYDLQPEMSSAELTEKLVAAIKSGKYDTIICNYP

400   NGDMVGHTGVMEAAVKAVEALDHCVEEVAKAVESVGGQLLITADHGNAEQ

450   MRDPATGQAHTAHTNLPVPLIYVGDKNVKAVEGGKLSDIAPTMLSLMGME

500   IPQEMTGKPLFIVE
```

sp|P08331|CPDB_ECOLI 2',3'-cyclic-nucleotide 2'-phosphodiesterase/3'-nucleotidase OS=Escherichia coli (strain K12) OX=83333 GN=cpdB PE=1 SV=2

```
0     MIKFSATLLATLIAASVNAATVDLRIMETTDLHSNMMDFDYYKDTATEKF

50    GLVRTASLINDARNEVKNSVLVDNGDLIQGSPLADYMSAKGLKAGDIHPV

100   YKALNTLDYTVGTLGNHEFNYGLDYLKNALAGAKFPYVNANVIDARTKQP

150   MFTPYLIKDTEVVDKDGKKQTLKIGYIGVVPPQIMGWDKANLSGKVTVND

200   ITETVRKYVPEMREKGADVVVVLAHSGLSADPYKVMAENSVYYLSEIPGV
```

250 NAIMFGHAHAVFPGKDFADIEGADIAKGTLNGVPAVMPGMWGDHLGVVDL
****
300 QLSNDSGKWQVTQAKAEARPIYDIANKKSLAAEDSKLVETLKADHDATRQ

350 FVSKPIGKSADNMYSYLALVQDDPTVQVVNNAQKAYVEHYIQGDPDLAKL

400 PVLSAAAPFKVGGRKNDPASYVEVEKGQLTFRNAADLYLYPNTLIVVKAS

450 GKEVKEWLECSAGQFNQIDPNSTKPQSLINWDGFRTYNFDVIDGVNYQID

500 VTQPARYDGECQMINANAERIKNLTFNGKPIDPNAMFLVATNNYRAYGGK

550 FAGTGDSHIAFASPDENRSVLAAWIADESKRAGEIHPAADNNWRLAPIAG

600 DKKLDIRFETSPSDKAAAFIKEKGQYPMNKVATDDIGFAIYQVDLSK

sp|P0A9K1|PHOH_ECOLI Protein PhoH OS=Escherichia coli (strain K12) OX=83333 GN=phoH PE=1 SV=1

0 MVTSCTGHVLDNQRATTRGVFSSGSHLVTLHFQPHPFFSCVTDAVNGARS

50 RFSAFYPKANYGLQGSQPSDVRAHNRAANGACDEYKQLKVLSMGRQKAVI
****
100 KARREAKRVLRRDSRSHKQREEESVTSLVQMGGVEAIGMARDSRDTSPIL

150 ARNEAQLHYLKAIESKQLIFATGEAGCGKTWISAAKAAEALIHKDVDRII

200 VTRPVLQADEDLGFLPGDIAEKFAPYFRPVYDVLVRRLGASFMQYCLRPE

250 IGKVEIAPFAYMRGRTFENAVVILDEAQNVTAAQMKMFLTRLGENVTVIV

300 NGDITQCDLPRGVCSGLSDALERFEEDEMVGIVRFGKEDCVRSALCQRTL

350 HAYS

sp|Q47150|DINJ_ECOLI Antitoxin DinJ OS=Escherichia coli (strain K12) OX=83333 GN=dinJ PE=1 SV=1

0 MAANAFVRARIDEDLKNQAADVLAGMGLTISDLVRITLTKVAREKALPFD
****
50 LREPNQLTIQSIKNSEAGIDVHKAKDADDLFDKLGI

sp|P33226|TORC_ECOLI Cytochrome c-type protein TorC OS=Escherichia coli (strain K12) OX=83333 GN=torC PE=1 SV=3

0 MRKLWNALRRPSARWSVLALVAIGIVIGIALIVLPHVGIKVTSTTEFCVS

50 CHSMQPVYEEYKQSVHFQNASGVRAECHDCHIPPDIPGMVKRKLEASNDI

100 YQTFIAHSIDTPEKFEAKRAELAEREWARMKENNSATCRSCHNYDAMDHA

150 KQHPEAARQMKVAAKDNQSCIDCHKGIAHQLPDMSSGFRKQFDELRASAN
****
200 DSGDTLYSIDIKPIYAAKGDKEASGSLLPASEVKVLKRDGDWLQIEITGW

```
250   TESAGRQRVLTQFPGKRIFVASIRGDVQQQVKTLEKTTVADTNTEWSKLQ

300   ATAWMKKGDMVNDIKPIWAYADSLYNGTCNQCHGAPEIAHFDANGWIGTL

350   NGMIGFTSLDKREERTLLKYLQMNASDTAGKAHGDKKEEK

sp|P24255|RP54_ECOLI RNA polymerase sigma-54 factor OS=Escherichia coli
(strain K12) OX=83333 GN=rpoN PE=1 SV=2

0     MKQGLQLRLSQQLAMTPQLQQAIRLLQLSTLELQQELQQALESNPLLEQI
                 *********************
50    DTHEEIDTRETQDSETLDTADALEQKEMPEELPLDASWDTIYTAGTPSGT

100   SGDYIDDELPVYQGETTQTLQDYLMWQVELTPFSDTDRAIATSIVDAVDE
                ***********************
150   TGYLTVPLEDILESIGDEEIDIDEVEAVLKRIQRFDPVGVAAKDLRDCLL

200   IQLSQFDKTTPWLEEARLIISDHLDLLANHDFRTLMRVTRLKEDVLKEAV

250   NLIQSLDPRPGQSIQTGEPEYVIPDVLVRKHNGHWTVELNSDSIPRLQIN

300   QHYASMCNNARNDGDSQFIRSNLQDAKWLIKSLESRNDTLLRVSRCIVEQ

350   QQAFFEQGEEYMKPMVLADIAQAVEMHESTISRVTTQKYLHSPRGIFELK

400   YFFSSHVNTEGGGEASSTAIRALVKKLIAAENPAKPLSDSKLTSLLSEQG

450   IMVARRTVAKYRESLSIPPSNQRKQLV

sp|P0A6X7|IHFA_ECOLI Integration host factor subunit alpha OS=Escherichia
coli (strain K12) OX=83333 GN=ihfA PE=1 SV=1
          *****************************
0     MALTKAEMSEYLFDKLGLSKRDAKELVELFFEEIRRALENGEQVKLSGFG

50    NFDLRDKNQRPGRNPKTGEDIPITARRVVTFRPGQKLKSRVENASPKDE

sp|P07003|POXB_ECOLI Pyruvate dehydrogenase [ubiquinone] OS=Escherichia
coli (strain K12) OX=83333 GN=poxB PE=1 SV=1

0     MKQTVAAYIAKTLESAGVKRIWGVTGDSLNGLSDSLNRMGTIEWMSTRHE

50    EVAAFAAGAEAQLSGELAVCAGSCGPGNLHLINGLFDCHRNHVPVLAIAA

100   HIPSSEIGSGYFQETHPQELFRECSHYCELVSSPEQIPQVLAIAMRKAVL

150   NRGVSVVVLPGDVALKPAPEGATMHWYHAPQPVVTPEEEELRKLAQLLRY

200   SSNIALMCGSGCAGAHKELVEFAGKIKAPIVHALRGKEHVEYDNPYDVGM

250   TGLIGFSSGFHTMMNADTLVLLGTQFPYRAFYPTDAKIIQIDINPASIGA
                           *****************************
300   HSKVDMALVGDIKSTLRALLPLVEEKADRKFLDKALEDYRDARKGLDDLA
      ******
```

```
350   KPSEKAIHPQYLAQQISHFAADDAIFTCDVGTPTVWAARYLKMNGKRRLL

400   GSFNHGSMANAMPQALGAQATEPERQVVAMCGDGGFSMLMGDFLSVVQMK

450   LPVKIVVFNNSVLGFVAMEMKAGGYLTDGTELHDTNFARIAEACGITGIR

500   VEKASEVDEALQRAFSIDGPVLVDVVVAKEELAIPPQIKLEQAKGFSLYM

550   LRAIISGRGDEVIELAKTNWLR
```

sp|P0AF78|YJFJ_ECOLI Uncharacterized protein YjfJ OS=Escherichia coli (strain K12) OX=83333 GN=yjfJ PE=3 SV=1

```
          ********************
0     MGILKSLFTLGKSFISQAEESIEETQGVRMLEQHIRDAKAELDKAGKSRV
          **********************
50    DLLARVKLSHDKLKDLRERKASLEARALEALSKNVNPSLINEVAEEIARL

100   ENLITAEEQVLSNLEVSRDGVEKAVTATAQRIAQFEQQMEVVKATEAMQR

150   AQQAVTTSTVGASSSVSTAAESLKRLQTRQAERQARLDAAAQLEKVADGR

200   DLDEKLAEAGIGGSNKSSAQDVLARLQRQQGE
```

sp|P0AGB6|RPOE_ECOLI ECF RNA polymerase sigma-E factor OS=Escherichia coli (strain K12) OX=83333 GN=rpoE PE=1 SV=1

```
0     MSEQLTDQVLVERVQKGDQKAFNLLVVRYQHKVASLVSRYVPSGDVPDVV
                                                  ******
50    QEAFIKAYRALDSFRGDSAFYTWLYRIAVNTAKNYLVAQGRRPPSSDVDA
      **************
100   IEAENFESGGALKEISNPENLMLSEELRQIVFRTIESLPEDLRMAITLRE

150   LDGLSYEEIAAIMDCPVGTVRSRIFRAREAIDNKVQPLIRR
```

sp|P76072|STFR_ECOLI Prophage side tail fiber protein homolog StfR OS=Escherichia coli (strain K12) OX=83333 GN=stfR PE=3 SV=2

```
0     MAVKISGVLKDGTGKPVQNCTIQLKAKRNSTTVVVNTLASENPDEAGRYS

50    MDVEYGQYSVILLVEGFPPSHAGTITVYEDSQPGTLNDFLGAMTEDDARP

100   EALRRFELMVEEVARNASAVAQNTAAAKKSASDASTSAREAATHAADAAD
                      **********************************
150   SARAASTSAGQAASSAQSASSSAGTASTKATEASKSAAAAESSKSAAATS
      *                            *********************
200   AGAAKTSETNASASLQSAATSASTATTKASEAATSARDAAASKEAAKSSE
      ****************
250   TNASSSASSAASSATAAGNSAKAAKTSETNARSSETAAGQSASAAAGSKT

300   AAASSASAASTSAGQASASATAAGKSAESAASSASTATTKAGEATEQASA
         ***********************************************
350   AARSASAAKTSETNAKASETSAESSKTAAASSASSAASSASSASASKDEA
      ***********
400   TRQASAAKSSATTASTKATEAAGSATAAAQSKSTAESAATRAETAAKRAE
```

```
450   DIASAVALEDASTTKKGIVQLSSATNSTSETLAATPKAVKSAYDNAEKRL

500   QKDQNGADIPDKGCFLNNINAVSKTDFADKRGMRYVRVNAPAGATSGKYY

550   PVVVMRSAGSVSELASRVIITTATRTAGDPMNNCEFNGFVMPGGWTDRGR

600   YAYGMFWQYQNNERAIHSIMMSNKGDDLRSVFYVDGAAFPVFAFIEDGLS

650   ISAPGADLVVNDTTYKFGATNPATECIAADVILDFKSGRGFYESHSLIVN

700   DNLSCKKLFATDEIVARGGNQIRMIGGEYGALWRNDGAKTYLLLTNQGDV

750   YGGWNTLRPFAIDNATGELVIGTKLSASLNGNALTATKLQTPRRVSGVEF

800   DGSKDITLTAAHVAAFARRATDTYADADGGVPWNAESGAYNVTRSGDSYI

850   LVNFYTGVGSCRTLQMKAHYRNGGLFYRSSRDGYGFEEDWAEVYTSKNLP

900   PESYPVGAPIPWPSDTVPSGYALMQGQAFDKSAYPKLAAAYPSGVIPDMR
                     ********************
950   GWTIKGKPASGRAVLSQEQDGIKSHTHSASASSTDLGTKTTSSFDYGTKS

1000  TNNTGAHTHSVSGSTNSAGAHTHSLANVNTASANSGAGSASTRLSVVHNQ

1050  NYATSSAGAHTHSLSGTAASAGAHAHTVGIGAHTHSVAIGSHGHTITVNA

1100  AGNAENTVKNIAFNYIVRLA
```

sp|P0AFD6|NUOI_ECOLI NADH-quinone oxidoreductase subunit I OS=Escherichia coli (strain K12) OX=83333 GN=nuoI PE=1 SV=1

```
0     MTLKELLVGFGTQVRSIWMIGLHAFAKRETRMYPEEPVYLPPRYRGRIVL

50    TRDPDGEERCVACNLCAVACPVGCISLQKAETKDGRWYPEFFRINFSRCI

100   FCGLCEEACPTTAIQLTPDFEMGEYKRQDLVYEKEDLLISGPGKYPEYNF
            ************************
150   YRMAGMAIDGKDKGEAENEAKPIDVKSLLP
```

sp|P76177|YDGH_ECOLI Protein YdgH OS=Escherichia coli (strain K12) OX=83333 GN=ydgH PE=1 SV=1

```
0     MKLKNTLLASALLSAMAFSVNAATELTPEQAAAVKPFDRVVVTGRFNAIG

50    EAVKAVSRRADKEGAASFYVVDTSDFGNSGNWRVVADLYKADAEKAEETS

100   NRVINGVVELPKDQAVLIEPFDTVTVQGFYRSQPEVNDAITKAAKAKGAY
                             ********************
150   SFYIVRQIDANQGGNQRITAFIYKKDAKKRIVQSPDVIPADSEAGRAALA

200   AGGEAAKKVEIPGVATTASPSSEVGRFFETQSSKGGRYTVTLPDGTKVEE

250   LNKATAAMMVPFDSIKFSGNYGNMTEVSYQVAKRAAKKGAKYYHITRQWQ
```

```
300   ERGNNLTVSADLYK
```

sp|P26266|FEPE_ECOLI Ferric enterobactin transport protein FepE OS=Escherichia coli (strain K12) OX=83333 GN=fepE PE=1 SV=2

```
0     MSSLNIKQGSDAHFPDYPLASPSNNEIDLLNLISVLWRAKKTVMAVVFAF

50    ACAGLLISFILPQKWTSAAVVTPPEPVQWQELEKSFTKLRVLDLDIKIDR
                                                 *******
100   TEAFNLFIKKFQSVSLLEEYLRSSPYVMDQLKEAKIDELDLHRAIVALSE
      **************************
150   KMKAVDDNASKKKDEPSLYTSWTLSFTAPTSEEAQTVLSGYIDYISTLVV
      *********************
200   KESLENVRNKLEIKTQFEKEKLAQDRIKTKNQLDANIQRLNYSLDIANAA
                   ********************
250   GIKKPVYSNGQAVKDDPDFSISLGADGIERKLEIEKAVTDVAELNGELRN

300   RQYLVEQLTKAHVNDVNFTPFKYQLSPSLPVKKDGPGKAIIVILSALIGG

350   MVACGGVLLRYAMASRKQDAMMADHLV
```

sp|Q46863|YGIS_ECOLI Probable deoxycholate-binding periplasmic protein YgiS OS=Escherichia coli (strain K12) OX=83333 GN=ygiS PE=1 SV=1

```
0     MYTRNLLWLVSLVSAAPLYAADVPANTPLAPQQVFRYNNHSDPGTLDPQK

50    VEENTAAQIVLDLFEGLVWMDGEGQVQPAQAERWEILDGGKRYIFHLRSG

100   LQWSDGQPLTAEDFVLGWQRAVDPKTASPFAGYLAQAHINNAAAIVAGKA

150   DVTSLGVKATDDRTLEVTLEQPVPWFTTMLAWPTLFPVPHHVIAKHGDSW

200   SKPENMVYNGAFVLDQWVVNEKITARKNPKYRDAQHTVLQQVEYLALDNS

250   VTGYNRYRAGEVDLTWVPAQQIPAIEKSLPGELRIIPRLNSEYYNFNLEK

300   PPFNDVRVRRALYLTVDRQLIAQKVLGLRTPATTLTPPEVKGFSATTFDE

350   LQKPMSERVAMAKALLKQAGYDASHPLRFELFYNKYDLHEKTAIALSSEW
                                        ****************
400   KKWLGAQVTLRTMEWKTYLDARRAGDFMLSRQSWDATYNDASSFLNTLKS
      ****
450   DSEENVGHWKNAQYDALLNQATQITDATKRNALYQQAEVIINQQAPLIPI

500   YYQPLIKLLKPYVGGFPLHNPQDYVYSKELYIKAH
```

sp|P36661|YCCE_ECOLI Uncharacterized protein YccE OS=Escherichia coli (strain K12) OX=83333 GN=yccE PE=4 SV=2

```
0     MGSNIHGISCTANNYLKQAWNDIKNEYEKNQTYSITLFENTLVCFMRLYN
        **********************************
50    ELRRKVNEEDTPCLECESLEKEFEEMQNDNDLSLFMRILRTNDTQIYSGV
```

100   SGGITYTIQYVRDIDIVRVSLPGRASESITDFKGYYWYNFMEYIENINAC

150   DDVFSEYCFDDENISVQPERINTPGISDLDSDIDLSGISFIQRETNQALG

200   LKYAPVDGDGYCLLRAILVLKQHDYSWALVSYKMQKEVYNEFIKMVDKKT

250   IEALVDTAFYNLREDVKTLFGVDLQSDNQIQGQSSLMSWSFLFFKKQFID

300   SCLNNEKCILHLPEFIFNDNKNLLALDTDTSDRIKAVKNFLVVLSDSICS

350   LFIVNSNVASISLGNESFSTDEDLEYGYLMNTGNHYDVYLPPELFAQAYK

400   LNNKEMNAQLDYLNRYAI

sp|P43329|HRPA_ECOLI ATP-dependent RNA helicase HrpA OS=Escherichia coli (strain K12) OX=83333 GN=hrpA PE=3 SV=3

0   MTEQQKLTFTALQQRLDSLMLRDRLRFSRRLHGVKKVKNPDAQQAIFQEM

50   AKEIDQAAGKVLLREAARPEITYPDNLPVSQKKQDILEAIRDHQVVIVAG

100   ETGSGKTTQLPKICMELGRGIKGLIGHTQPRRLAARTVANRIAEELKTEP

150   GGCIGYKVRFSDHVSDNTMVKLMTDGILLAEIQQDRLLMQYDTIIIDEAH

200   ERSLNIDFLLGYLKELLPRRPDLKIIITSATIDPERFSRHFNNAPIIEVS

250   GRTYPVEVRYRPIVEEADDTERDQLQAIFDAVDELSQESHGDILIFMSGE

300   REIRDTADALNKLNLRHTEILPLYARLSNSEQNRVFQSHSGRRIVLATNV

350   AETSLTVPGIKYVIDPGTARISRYSYRTKVQRLPIEPISQASANQRKGRC

400   GRVSEGICIRLYSEDDFLSRPEFTDPEILRTNLASVILQMTALGLGDIAA

450   FPFVEAPDKRNIQDGVRLLEELGAITTDEQASAYKLTPLGRQLSQLPVDP
                                       ****
500   RLARMVLEAQKHGCVREAMIITSALSIQDPRERPMDKQQASDEKHRRFHD
      ******
550   KESDFLAFVNLWNYLGEQQKALSSNAFRRLCRTDYLNYLRVREWQDIYTQ

600   LRQVVKELGIPVNSEPAEYREIHIALLTGLLSHIGMKDADKQEYTGARNA

650   RFSIFPGSGLFKKPPKWVMVAELVETSRLWGRIAARIDPEWVEPVAQHLI

700   KRTYSEPHWERAQGAVMATEKVTVYGLPIVAARKVNYSQIDPALCRELFI
                             ****
750   RHALVEGDWQTRHAFFRENLKLRAEVEELEHKSRRRDILVDDETLFEFYD
                   ****
800   QRISHDVISARHFDSWWKKVSRETPDLLNFEKSMLIKEGAEKISKLDYPN

850   FWHQGNLKLRLSYQFEPGADADGVTVHIPLPLLNQVEESGFEWQIPGLRR

900   ELVIALIKSLPKPVRRNFVPAPNYAEAFLGRVKPLELPLLDSLERELRRM

****
950   TGVTVDREDWHWDQVPDHLKITFRVVDDKNKKLKEGRSLQDLKDALKGKV

1000 QETLSAVADDGIEQSGLHIWSFGQLPESYEQKRGNYKVKAWPALVDERDS

1050 VAIKLFDNPLEQKQAMWNGLRRLLLLNIPSPIKYLHEKLPNKAKLGLYFN

1100 PYGKVLELIDDCISCGVDKLIDANGGPVWTEEGFAALHEKVRAELNDTVV

1150 DIAKQVEQILTAVFNINKRLKGRVDMTMALGLSDIKAQMGGLVYRGFVTG

1200 NGFKRLGDTLRYLQAIEKRLEKLAVDPHRDRAQMLKVENVQQAWQQWINK

1250 LPPARREDEDVKEIRWMIEELRVSYFAQQLGTPYPISDKRILQAMEQISG

1300

sp|P08550|CVPA_ECOLI Colicin V production protein OS=Escherichia coli (strain K12) OX=83333 GN=cvpA PE=1 SV=1

0   MVWIDYAIIAVIAFSSLVSLIRGFVREALSLVTWGCAFFVASHYYTYLSV

50   WFTGFEDELVRNGIAIAVLFIATLIVGAIVNFVIGQLVEKTGLSGTDRVL

****
100   GVCFGALRGVLIVAAILFFLDSFTGVSKSEDWSKSQLIPQFSFIIRCFFD

150   YLQSSSSFLPRA

sp|P0AE85|CPXP_ECOLI Periplasmic protein CpxP OS=Escherichia coli (strain K12) OX=83333 GN=cpxP PE=1 SV=1

0   MRIVTAAVMASTLAVSSLSHAAEVGSGDNWHPGEELTQRSTQSHMFDGIS

50   LTEHQRQQMRDLMQQARHEQPPVNVSELETMHRLVTAENFDENAVRAQAE

****
100   KMANEQIARQVEMAKVRNQMYRLLTPEQQAVLNEKHQQRMEQLRDVTQWQ

****
150   KSSSLKLLSSSNSRSQ

sp|P76129|DOSP_ECOLI Oxygen sensor protein DosP OS=Escherichia coli (strain K12) OX=83333 GN=dosP PE=1 SV=4

0   MKLTDADNAADGIFFPALEQNMMGAVLINENDEVMFFNPAAEKLWGYKRE

****
50   EVIGNNIDMLIPRDLRPAHPEYIRHNREGGKARVEGMSRELQLEKKDGSK

****
100   IWTRFALSKVSAEGKVYYLALVRDASVEMAQKEQTRQLIIAVDHLDRPVI

150   VLDPERHIVQCNRAFTEMFGYCISEASGMQPDTLLNIPEFPADNRIRLQQ

200   LLWKTARDQDEFLLLTRTGEKIWIKASISPVYDVLAHLQNLVMTFSDITE

250   ERQIRQLEGNILAAMCSSPPFHEMGEIICRNIESVLNESHVSLFALRNGM

300   PIHWASSSHGAEIQNAQSWSATIRQRDGAPAGILQIKTSSGAETSAFIER

350   VADISQHMAALALEQEKSRQHIEQLIQFDPMTGLPNRNNLHNYLDDLVDK

400   AVSPVVYLIGVDHIQDVIDSLGYAWADQALLEVVNRFREKLKPDQYLCRI

450   EGTQFVLVSLENDVSNITQIADELRNVVSKPIMIDDKPFPLTLSIGISYD

500   LGKNRDYLLSTAHNAMDYIRKNGGNGWQFFSPAMNEMVKERLVLGAALKE

550   AISNNQLKLVYQPQIFAETGELYGIEALARWHDPLHGHVPPSRFIPLAEE

600   IGEIENIGRWVIAEACRQLAEWRSQNIHIPALSVNLSALHFRSNQLPNQV

650   SDAMHAWGIDGHQLTVEITESMMMEHDTEIFKRIQILRDMGVGLSVDDFG

700   TGFSGLSRLVSLPVTEIKIDKSFVDRCLTEKRILALLEAITSIGQSLNLT

750   VVAEGVETKEQFEMLRKIHCRVIQGYFFSRPLPAEEIPGWMSSVLPLKI

sp|P0A9Q7|ADHE_ECOLI Aldehyde-alcohol dehydrogenase OS=Escherichia coli (strain K12) OX=83333 GN=adhE PE=1 SV=2

0   MAVTNVAELNALVERVKKAQREYASFTQEQVDKIFRAAALAAADARIPLA

50   KMAVAESGMGIVEDKVIKNHFASEYIYNAYKDEKTCGVLSEDDTFGTITI

100   AEPIGIICGIVPTTNPTSTAIFKSLISLKTRNAIIFSPHPRAKDATNKAA

150   DIVLQAAIAAGAPKDLIGWIDQPSVELSNALMHHPDINLILATGGPGMVK

200   AAYSSGKPAIGVGAGNTPVVIDETADIKRAVASVLMSKTFDNGVICASEQ

250   SVVVVDSVYDAVRERFATHGGYLLQGKELKAVQDVILKNGALNAAIVGQP

300   AYKIAELAGFSVPENTKILIGEVTVVDESEPFAHEKLSPTLAMYRAKDFE

350   DAVEKAEKLVAMGGIGHTSCLYTDQDNQPARVSYFGQKMKTARILINTPA

400   SQGGIGDLYNFKLAPSLTLGCGSWGGNSISENVGPKHLINKKTVAKRAEN

450   MLWHKLPKSIYFRRGSLPIALDEVITDGHKRALIVTDRFLFNNGYADQIT

500   SVLKAAGVETEVFFEVEADPTLSIVRKGAELANSFKPDVIIALGGGSPMD

550   AAKIMWVMYEHPETHFEELALRFMDIRKRIYKFPKMGVKAKMIAVTTTSG

600   TGSEVTPFAVVTDDATGQKYPLADYALTPDMAIVDANLVMDMPKSLCAFG

650   GLDAVTHAMEAYVSVLASEFSDGQALQALKLLKEYLPASYHEGSKNPVAR

700   ERVHSAATIAGIAFANAFLGVCHSMAHKLGSQFHIPHGLANALLICNVIR

750   YNANDNPTKQTAFSQYDRPQARRRYAEIADHLGLSAPGDRTAAKIEKLLA

```
800   WLETLKAELGIPKSIREAGVQEADFLANVDKLSEDAFDDQCTGANPRYPL
                        ***********************
850   ISELKQILLDTYYGRDYVEGETAAKKEAAPAKAEKKAKKSA
```

sp|P0ACZ4|EVGA_ECOLI DNA-binding transcriptional activator EvgA
OS=Escherichia coli (strain K12) OX=83333 GN=evgA PE=1 SV=1

```
0     MNAIIIDDHPLAIAAIRNLLIKNDIEILAELTEGGSAVQRVETLKPDIVI

50    IDVDIPGVNGIQVLETLRKRQYSGIIIIVSAKNDHFYGKHCADAGANGFV
                                    ********************
100   SKKEGMNNIIAAIEAAKNGYCYFPFSLNRFVGSLTSDQQKLDSLSKQEIS
      **                  ********************
150   VMRYILDGKDNNDIAEKMFISNKTVSTYKSRLMEKLECKSLMDLYTFAQR

200   NKIG
```

sp|P0AE63|CHAB_ECOLI Putative cation transport regulator ChaB
OS=Escherichia coli (strain K12) OX=83333 GN=chaB PE=3 SV=1

```
                           *****************************
0     MPYKTKSDLPESVKHVLPSHAQDIYKEAFNSAWDQYKDKEDRRDDASREE
      **************************
50    TAHKVAWAAVKHEYAKGDDDKWHKKS
```

sp|P23325|ARPA_ECOLI Ankyrin repeat protein A OS=Escherichia coli (strain
K12) OX=83333 GN=arpA PE=4 SV=3

```
                           *****************************
0     MITRIPRSSFSANINNTAQTNEHQTLSELFYKELEDKFSGKELATPLLKS
      ****         ********************
50    FSENCRQNGRHIFSNKDFVIKFSTSVLQADKKEITIINKNENTTLTQTIA
                 ****************************
100   PIFEKYLMEILPQRSDTLDKQELNLKSDRKEKEFPRIKLNGQCYFPGRPQ

150   NRIVCRHIAAQYINDIYQNVDYKPHQDDYSSAEKFLTHFNKKCKNQTLAL

200   VSSRPEGRCVAACGDFGLVMKAYFDKMESNGISVMAAILLVDNHALTVRL
                             ********************
250   RIKNTTEGCTHYVVSVYDPNVTNDKIRIMSESKENIKHYSLMDFMNVDYS

300   LLKWSNDHVINQSVAIIPALPKEQLLMLKGSVDEITPPLSPATMNLLMAI

350   GQNHQLTQLMIQLQKMPELHRTEMLTAYNSINLPGLYLAINYGNADIVET

400   IFNSLSETGYEGLLSKKNLMHILEAKDKNGFSGLFLAISRKDKNVVTSIL

450   NALPKLAATHHLDNEQVYKFLSAKNRTSSHVLYHVMANGDADMLKIVLNA

500   LPLLIRTCHLTKEQVLDLLKAKDFYGCPGLYLAMQNGHSDIVKVILEALP

550   SLAQEINISASDIVDLLTAKSLARDTGLFMAMQRGHMNVINTIFNALPTL

600   FNTFKFDKKNMKPLLLANNSNEYPGLFSAIQHKQQNVVETVYLALSDHAR
```

```
650   LFGFTAEDIMDFWQHKAPQKYSAFELAFEFGHRVIAELILNTLNKMAESF

700   GFTDNPRYIAEKNYMEALLKKASPHTVR
```

sp|P25743|YCHE_ECOLI UPF0056 membrane protein YhcE OS=Escherichia coli (strain K12) OX=83333 GN=ychE PE=3 SV=2

```
0     MIQTFFDFPVYFKFFIGLFALVNPVGIIPVFISMTSYQTAAARNKTNLTA
                                                **********
50    NLSVAIILWISLFLGDTILQLFGISIDSFRIAGGILVVTIAMSMISGKLG
      ********************
100   EDKQNKQEKSETAVRESIGVVPLALPLMAGPGAISSTIVWGTRYHSISYL

150   FGFFVAIALFALCCWGLFRMAPWLVRVLRQTGINVITRIMGLLLMALGIE

200   FIVTGIKGIFPGLLN
```

sp|P10805|ENVY_ECOLI Porin thermoregulatory protein EnvY OS=Escherichia coli (strain K12) OX=83333 GN=envY PE=4 SV=2

```
0     MQLSSSEPCVVILTEKEVEVSVNNHATFTLPKNYLAAFACNNNVIELSTL

50    NHVLITHINRNIINDYLLFLNKNLTCVKPWSRLATPVIACHSRTPEVFRL

100   AANHSKQQPSRPCEAELTRALLFTVLSNFLEQSRFIALLMYILRSSVRDS
                         ********************
150   VCRIIQSDIQHYWNLRIVASSLCLSPSLLKKKLKNENTSYSQIVTECRMR

200   YAVQMLLMDNKNITQVAQLCGYSSTSYFISVFKAFYGLTPLNYLAKQRQK

250   VMW
```

sp|P37657|BCSE_ECOLI Cyclic di-GMP binding protein BcsE OS=Escherichia coli (strain K12) OX=83333 GN=bcsE PE=1 SV=1

```
0     MRDIVDPVFSIGISSLWDELRHMPAGGVWWFNVDRHEDAISLANQTIASQ
               **********************
50    AETAHVAVISMDSDPAKIFQLDDSQGPEKIKLFSMLNHEKGLYYLTRDLQ

100   CSIDPHNYLFILVCANNAWQNIPAERLRSWLDKMNKWSRLNHCSLLVINP

150   GNNNDKQFSLLLEEYRSLFGLASLRFQGDQHLLDIAFWCNEKGVSARQQL

200   SVQQQNGIWTLVQSEEAEIQPRSDEKRILSNVAVLEGAPPLSEHWQLFNN

250   NEVLFNEARTAQAATVVFSLQQNAQIEPLARSIHTLRRQRGSAMKILVRE

300   NTASLRATDERLLLACGANMVIPWNAPLSRCLTMIESVQGQKFSRYVPED

350   ITTLLSMTQPLKLRGFQKWDVFCNAVNNMMNNPLLPAHGKGVLVALRPVP

400   GIRVEQALTLCRPNRTGDIMTIGGNRLVLFLSFCRINDLDTALNHIFPLP

450   TGDIFSNRMVWFEDDQISAELVQMRLLAPEQWGMPLPLTQSSKPVINAEH
```

500 DGRHWRRIPEPMRLLDDAVERSS

sp|P33602|NUOG_ECOLI NADH-quinone oxidoreductase subunit G OS=Escherichia coli (strain K12) OX=83333 GN=nuoG PE=1 SV=4

0 MATIHVDGKEYEVNGADNLLEACLSLGLDIPYFCWHPALGSVGACRQCAV
****

50 KQYQNAEDTRGRLVMSCMTPASDGTFISIDDEEAKQFRESVVEWLMTNHP

100 HDCPVCEEGGNCHLQDMTVMTGHSFRRYRFTKRTHRNQDLGPFISHEMNR

150 CIACYRCVRYYKDYADGTDLGVYGAHDNVYFGRPEDGTLESEFSGNLVEI

200 CPTGVFTDKTHSERYNRKWDMQFAPSICQQCSIGCNISPGERYGELRRIE

250 NRYNGTVNHYFLCDRGRFGYGYVNLKDRPRQPVQRRGDDFITLNAEQAMQ

300 GAADILRQSKKVIGIGSPRASVESNFALRELVGEENFYTGIAHGEQERLQ

350 LALKVLREGGIYTPALREIESYDAVLVLGEDVTQTGARVALAVRQAVKGK

400 AREMAAAQKVADWQIAAILNIGQRAKHPLFVTNVDDTRLDDIAAWTYRAP

450 VEDQARLGFAIAHALDNSAPAVDGIEPELQSKIDVIVQALAGAKKPLIIS

500 GTNAGSLEVIQAAANVAKALKGRGADVGITMIARSVNSMGLGIMGGGSLE

550 EALTELETGRADAVVVLENDLHRHASAIRVNAALAKAPLVMVVDHQRTAI

600 MENAHLVLSAASFAESDGTVINNEGRAQRFFQVYDPAYYDSKTVMLESWR

650 WLHSLHSTLLSREVDWTQLDHVIDAVVAKIPELAGIKDAAPDATFRIRGQ

700 KLAREPHRYSGRTAMRANISVHEPRQPQDIDTMFTFSMEGNNQPTAHRSQ

750 VPFAWAPGWNSPQAWNKFQDEVGGKLRFGDPGVRLFETSENGLDYFTSVP

800 ARFQPQDGKWRIAPYYHLFGSDELSQRAPVFQSRMPQPYIKLNPADAAKL

850 GVNAGTRVSFSYDGNTVTLPVEIAEGLTAGQVGLPMGMSGIAPVLAGAHL

900 EDLKEAQQ

sp|P21513|RNE_ECOLI Ribonuclease E OS=Escherichia coli (strain K12) OX=83333 GN=rne PE=1 SV=6

0 MKRMLINATQQEELRVALVDGQRLYDLDIESPGHEQKKANIYKGKITRIE

50 PSLEAAFVDYGAERHGFLPLKEIAREYFPANYSAHGRPNIKDVLREGQEV

100 IVQIDKEERGNKGAALTTFISLAGSYLVLMPNNPRAGGISRRIEGDDRTE

150 LKEALASLELPEGMGLIVRTAGVGKSAEALQWDLSFRLKHWEAIKKAAES

200   RPAPFLIHQESNVIVRAFRDYLRQDIGEILIDNPKVLELARQHIAALGRP

250   DFSSKIKLYTGEIPLFSHYQIESQIESAFQREVRLPSGGSIVIDSTEALT

300   AIDINSARATRGGDIEETAFNTNLEAADEIARQLRLRDLGGLIVIDFIDM

350   TPVRHQRAVENRLREAVRQDRARIQISHISRFGLLEMSRQRLSPSLGESS
****

400   HHVCPRCSGTGTVRDNESLSLSILRLIEEEALKENTQEVHAIVPVPIASY

450   LLNEKRSAVNAIETRQDGVRCVIVPNDQMETPHYHVLRVRKGEETPTLSY

500   MLPKLHEEAMALPSEEEFAERKRPEQPALATFAMPDVPPAPTPAEPAAPV

550   VAPAPKAAPATPAAPAQPGLLSRFFGALKALFSGGEETKPTEQPAPKAEA

600   KPERQQDRRKPRQNNRRDRNERRDTRSERTEGSDNREENRRNRRQAQQQT

650   AETRESRQQAEVTEKARTADEQQAPRRERSRRRNDDKRQAQQEAKALNVE

700   EQSVQETEQEERVRPVQPRRKQRQLNQKVRYEQSVAEEAVVAPVVEETVA

750   AEPIVQEAPAPRTELVKVPLPVVAQTAPEQQEENNADNRDNGGMPRRSRR

800   SPRHLRVSGQRRRRYRDERYPTQSPMPLTVACASPELASGKVWIRYPIVR

850   PQDVQVEEQREQEEVHVQPMVTEVPVAAAIEPVVSAPVVEEVAGVVEAPV

900   QVAEPQPEVVETTHPEVIAAAVTEQPQVITESDVAVAQEVAEQAEPVVEP

950   QEETADIEEVVETAEVVVAEPEVVAQPAAPVVAEVAAEVETVAAVEPEVT

1000 VEHNHATAPMTRAPAPEYVPEAPRHSDWQRPTFAFEGKGAAGGHTATHHA

1050 SAAPARPQPVE

sp|P77495|PRPE_ECOLI Propionate--CoA ligase OS=Escherichia coli (strain K12) OX=83333 GN=prpE PE=3 SV=1

0   MSFSEFYQRSINEPEQFWAEQARRIDWQTPFTQTLDHSNPPFARWFCEGR

50   TNLCHNAIDRWLEKQPEALALIAVSSETEEERTFTFRQLHDEVNAVASML

100   RSLGVQRGDRVLVYMPMIAEAHITLLACARIGAIHSVVFGGFASHSVAAR

150   IDDAKPVLIVSADAGARGGKIIPYKKLLDDAISQAQHQPRHVLLVDRGLA

200   KMARVSGRDVDFASLRHQHIGARVPVAWLESNETSCILYTSGTTGKPKGV

250   QRDVGGYAVALATSMDTIFGGKAGSVFFCASDIGWVVGHSYIVYAPLLAG

300   MATIVYEGLPTWPDCGVWWTIVEKYQVSRMFSAPTAIRVLKKFPTAEIRK

350 HDLSSLEVLYLAGEPLDEPTASWVSNTLDVPVIDNYWQTESGWPIMAIAR

400 GLDDRPTRLGSPGVPMYGYNVQLLNEVTGEPCGVNEKGMLVVEGPLPPGC

450 IQTIWGDDGRFVKTYWSLFSRPVYATFDWGIRDADGYHFILGRTDDVINV
****

500 AGHRLGTREIEESISSHPGVAEVAVVGVKDALKGQVAVAFVIPKESDSLE
****

550 DRDVAHSQEKAIMALVDSQIGNFGRPAHVWFVSQLPKTRSGKMLRRTIQA

600 ICEGRDPGDLTTIDDPASLDQIRQAMEE

sp|P13009|METH_ECOLI Methionine synthase OS=Escherichia coli (strain K12) OX=83333 GN=metH PE=1 SV=5

0 MSSKVEQLRAQLNERILVLDGGMGTMIQSYRLNEADFRGERFADWPCDLK

50 GNNDLLVLSKPEVIAAIHNAYFEAGADIIETNTFNSTTIAMADYQMESLS

100 AEINFAAAKLARACADEWTARTPEKPRYVAGVLGPTNRTASISPDVNDPA

150 FRNITFDGLVAAYRESTKALVEGGADLILIETVFDTLNAKAAVFAVKTEF

200 EALGVELPIMISGTITDASGRTLSGQTTEAFYNSLRHAEALTFGLNCALG

250 PDELRQYVQELSRIAECYVTAHPNAGLPNAFGEYDLDADTMAKQIREWAQ

300 AGFLNIVGGCCGTTPQHIAAMSRAVEGLAPRKLPEIPVACRLSGLEPLNI
****

350 GEDSLFVNVGERTNVTGSAKFKRLIKEEKYSEALDVARQQVENGAQIIDI

400 NMDEGMLDAEAAMVRFLNLIAGEPDIARVPIMIDSSKWDVIEKGLKCIQG

450 KGIVNSISMKEGVDAFIHHAKLLRRYGAAVVVMAFDEQGQADTRARKIEI

500 CRRAYKILTEEVGFPPEDIIFDPNIFAVATGIEEHNNYAQDFIGACEDIK

550 RELPHALISGGVSNVSFSFRGNDPVREAIHAVFLYYAIRNGMDMGIVNAG

600 QLAIYDDLPAELRDAVEDVILNRRDDGTERLLELAEKYRGSKTDDTANAQ

650 QAEWRSWEVNKRLEYSLVKGITEFIEQDTEEARQQATRPIEVIEGPLMDG

700 MNVVGDLFGEGKMFLPQVVKSARVMKQAVAYLEPFIEASKEQGKTNGKMV

750 IATVKGDVHDIGKNIVGVVLQCNNYEIVDLGVMVPAEKILRTAKEVNADL

800 IGLSGLITPSLDEMVNVAKEMERQGFTIPLLIGGATTSKAHTAVKIEQNY

850 SGPTVYVQNASRTVGVVAALLSDTQRDDFVARTRKEYETVRIQHGRKKPR

900 TPPVTLEAARDNDFAFDWQAYTPPVAHRLGVQEVEASIETLRNYIDWTPF

950 FMTWSLAGKYPRILEDEVVGVEAQRLFKDANDMLDKLSAEKTLNPRGVVG

1000 LFPANRVGDDIEIYRDETRTHVINVSHHLRQQTEKTGFANYCLADFVAPK

1050 LSGKADYIGAFAVTGGLEEDALADAFEAQHDDYNKIMVKALADRLAEAFA

1100 EYLHERVRKVYWGYAPNENLSNEELIRENYQGIRPAPGYPACPEHTEKAT

1150 IWELLEVEKHTGMKLTESFAMWPGASVSGWYFSHPDSKYYAVAQIQRDQV

1200 EDYARRKGMSVTEVERWLAPNLGYDAD

sp|P37685|ALDB_ECOLI Aldehyde dehydrogenase B OS=Escherichia coli (strain K12) OX=83333 GN=aldB PE=1 SV=2

0   MTNNPPSAQIKPGEYGFPLKLKARYDNFIGGEWVAPADGEYYQNLTPVTG
             ****
50   QLLCEVASSGKRDIDLALDAAHKVKDKWAHTSVQDRAAILFKIADRMEQN

100   LELLATAETWDNGKPIRETSAADVPLAIDHFRYFASCIRAQEGGISEVDS

150   ETVAYHFHEPLGVVGQIIPWNFPLLMASWKMAPALAAGNCVVLKPARLTP

200   LSVLLLMEIVGDLLPPGVVNVVNGAGGVIGEYLATSKRIAKVAFTGSTEV

250   GQQIMQYATQNIIPVTLELGGKSPNIFFADVMDEEDAFFDKALEGFALFA

300   FNQGEVCTCPSRALVQESIYERFMERAIRRVESIRSGNPLDSVTQMGAQV

350   SHGQLETILNYIDIGKKEGADVLTGGRRKLLEGELKDGYYLEPTILFGQN

400   NMRVFQEEIFGPVLAVTTFKTMEEALELANDTQYGLGAGVWSRNGNLAYK

450   MGRGIQAGRVWTNCYHAYPAHAAFGGYKQSGIGRETHKMMLEHYQQTKCL

500   LVSYSDKPLGLF

sp|P39180|AG43_ECOLI Antigen 43 OS=Escherichia coli (strain K12) OX=83333 GN=flu PE=1 SV=3

0   MKRHLNTCYRLVWNHMTGAFVVASELARARGKRGGVAVALSLAAVTSLPV

50   LAADIVVHPGETVNGGTLANHDNQIVFGTTNGMTISTGLEYGPDNEANTG

100   GQWVQDGGTANKTTVTSGGLQRVNPGGSVSDTVISAGGGQSLQGRAVNTT

150   LNGGEQWMHEGAIATGTVINDKGWQVVKPGTVATDTVVNTGAEGGPDAEN

200   GDTGQFVRGDAVRTTINKNGRQIVRAEGTANTTVVYAGGDQTVHGHALDT

250   TLNGGYQYVHNGGTASDTVVNSDGWQIVKNGGVAGNTTVNQKGRLQVDAG

300   GTATNVTLKQGGALVTSTAATVTGINRLGAFSVVEGKADNVVLENGGRLD

350   VLTGHTATNTRVDDGGTLDVRNGGTATTVSMGNGGVLLADSGAAVSGTRS

400   DGKAFSIGGGQADALMLEKGSSFTLNAGDTATDTTVNGGLFTARGGTLAG

450   TTTLNNGAILTLSGKTVNNDTLTIREGDALLQGGSLTGNGSVEKSGSGTL

500   TVSNTTLTQKAVNLNEGTLTLNDSTVTTDVIAQRGTALKLTGSTVLNGAI

550   DPTNVTLASGATWNIPDNATVQSVVDDLSHAGQIHFTSTRTGKFVPATLK

600   VKNLNGQNGTISLRVRPDMAQNNADRLVIDGGRATGKTILNLVNAGNSAS

650   GLATSGKGIQVVEAINGATTEEGAFVQGNRLQAGAFNYSLNRDSDESWYL

700   RSENAYRAEVPLYASMLTQAMDYDRIVAGSRSHQTGVNGENNSVRLSIQG

750   GHLGHDNNGGIARGATPESSGSYGFVRLEGDLMRTEVAGMSVTAGVYGAA
       ****
800   GHSSVDVKDDDGSRAGTVRDDAGSLGGYLNLVHTSSGLWADIVAQGTRHS

850   MKASSDNNDFRARGWGWLGSLETGLPFSITDNLMLEPQLQYTWQGLSLDD

900   GKDNAGYVKFGHGSAQHVRAGFRLGSHNDMTFGEGTSSRAPLRDSAKHSV

950   SELPVNWWVQPSVIRTFSSRGDMRVGTSTAGSGMTFSPSQNGTSLDLQAG

1000 LEARVRENITLGVQAGYAHSVSGSSAEGYNGQATLNVTF

sp|P17410|CHBR_ECOLI HTH-type transcriptional regulator ChbR
OS=Escherichia coli (strain K12) OX=83333 GN=chbR PE=1 SV=1

0   MMQPVINAPEIATAREQQLFNGKNFHVFIYNKTESISGLHQHDYYEFTLV

50   LTGRYFQEINGKRVLLERGDFVFIPLGSHHQSFYEFGATRILNVGISKRF

100   FEQHYLPLLPYCFVASQVYRTNNAFLTYVETVISSLNFRETGLEEFVEMV
                          ****
150   TFYVINRLRHYREEQVIDDVPQWLKSTVEKMHDKEQFSESALENMVALSA
      **
200   KSQEYLTRATQRYYGKTPMQIINEIRINFAKKQLEMTNYSVTDIAFEAGY

250   SSPSLFIKTFKKLTSFTPKSYRKKLTEFNQ

sp|P0AE78|CORC_ECOLI Magnesium and cobalt efflux protein CorC
OS=Escherichia coli (strain K12) OX=83333 GN=corC PE=1 SV=1
      ****
0   MSDDNSHSSDTISNKKGFFSLLLSQLFHGEPKNRDELLALIRDSGQNDLI

50   DEDTRDMLEGVMDIADQRVRDIMIPRSQMITLKRNQTLDECLDVIIESAH

100   SRFPVISEDKDHIEGILMAKDLLPFMRSDAEAFSMDKVLRQAVVVPESKR

150   VDRMLKEFRSQRYHMAIVIDEFGGVSGLVTIEDILELIVGEIEDEYDEED

200   DIDFRQLSRHTWTVRALASIEDFNEAFGTHFSDEEVDTIGGLVMQAFGHL

```
250   PARGETIDIDGYQFKVAMADSRRIIQVHVKIPDDSPQPKLDE
```

sp|P0AD05|YECA_ECOLI Uncharacterized protein YecA OS=Escherichia coli (strain K12) OX=83333 GN=yecA PE=4 SV=1

```
0     MKTGPLNESELEWLDDILTKYNTDHAILDVAELDGLLTAVLSSPQEIEPE

50    QWLVAVWGGADYVPRWASEKEMTRFMNLAFQHMADTAERLNEFPEQFEPL
                                                     ***
100   FGLREVDGSELTIVEEWCFGYMRGVALSDWSTLPDSLKPALEAIALHGTE
      ********************
150   ENFERVEKMSPEAFEESVDAIRLAALDLHAYWMAHPQEKAVQQPIKAEEK

200   PGRNDPCPCGSGKKFKQCCLH
```

sp|P0AF34|YIIR_ECOLI Uncharacterized protein YiiR OS=Escherichia coli (strain K12) OX=83333 GN=yiiR PE=4 SV=1

```
0     MTIQQWLFSFKGRIGRRDFWIWIGLWFAGMLVLFSLAGKNLLDIQTAAFC

50    LVCLLWPTAAVTVKRLHDRGRSGAWAFLMIVAWMLLAGNWAILPGVWQWA
                              **********************
100   VGRFVPTLILVMMLIDLGAFVGTQGENKYGKDTQDVKYKADNKSSN
```

sp|P32672|PTFC2_ECOLI Fructose-like permease IIC component 2 OS=Escherichia coli (strain K12) OX=83333 GN=frwC PE=3 SV=1

```
0     MNELVQILKNTRQHLMTGVSHMIPFVVSGGILLAVSVMLYGKGAVPDAVA

50    DPNLKKLFDIGVAGLTLMVPFLAAYIGYSIAERSALAPCAIGAWVGNSFG

100   AGFFGALIAGIIGGIVVHYLKKIPVHKVLRSVMPIFIIPIVGTLITAGIM

150   MWGLGEPVGALTNSLTQWLQGMQQGSIVMLAVIMGLMLAFDMGGPVNKVA

200   YAFMLICVAQGVYTVVAIAAVGICIPPLGMGLATLIGRKNFSAEERETGK

250   AALVMGCVGVTEGAIPFAAADPLRVIPSIMVGSVCGAVTAALVGAQCYAG
                                    ********************
300   WGGLIVLPVVEGKLGYIAAVAVGAVVTAVCVNVLKSLARKNGSSTDEKED
      *********
350   DLDLDFEIN
```

sp|P33029|YEIQ_ECOLI Uncharacterized oxidoreductase YeiQ OS=Escherichia coli (strain K12) OX=83333 GN=yeiQ PE=3 SV=1

```
0     MNTIASVTLPHHVHAPRYDRQQLQSRIVHFGFGAFHRAHQALLTDRVLNA

50    QGGDWGICEISLFSGDQLMSQLRAQNHLYTVLEKGADGNQVIIVGAVHEC

100   LNAKLDSLAAIIEKFCEPQVAIVSLTITEKGYCIDPATGALDTSNPRIIH

150   DLQTPEEPHSAPGILVEALKRRRERGLTPFTVLSCDNIPDNGHVVKNAVL
```

```
200   GMAEKRSPELAGWIKEHVSFPGTMVDRIVPAATDESLVEISQHLGVNDPC

250   AISCEPFIQWVVEDNFVAGRPAWEVAGVQMVNDVLPWEEMKLRMLNGSHS

300   FLAYLGYLSGFAHISDCMQDRAFRHAARTLMLDEQAPTLQIKDVDLTQYA

350   DKLIARFANPALKHKTWQIAMDGSQKLPQRMLAGIRIHQGRETDWSLLAL
                         ********************
400   GVAGWMRYVSGVDDAGNAIDVRDPLSDKIRELVAGSSSEQRVTALLSLRE

450   VFGDDLPDNPHFVQAIEQAWQQIVQFGAHQALLNTLKI
```

sp|P39321|TAMB_ECOLI Translocation and assembly module subunit TamB OS=Escherichia coli (strain K12) OX=83333 GN=tamB PE=1 SV=2

```
0     MSLWKKISLGVVIVILLLLGSVAFLVGTTSGLHLVFKAADRWVPGLDIGK
                                                    **
50    VTGGWRDLTLSDVRYEQPGVAVKAGNLHLAVGLECLWNSSVCINDLALKD
      ****************************
100   IQVNIDSKKMPPSEQVEEEEDSGPLDLSTPYPITLTRVALDNVNIKIDDT

150   TVSVMDFTSGLNWQEKTLTLKPTSLKGLLIALPKVAEVAQEEVVEPKIEN

200   PQPDEKPLGETLKDLFSRPVLPEMTDVHLPLNLNIEEFKGEQLRVTGDTD
             *********************
250   ITVSTMLLKVSSIDGNTKLDALDIDSSQGIVNASGTAQLSDNWPVDITLN

300   STLNVEPLKGEKVKLKMGGALREQLEIGVNLSGPVDMDLRAQTRLAEAGL

350   PLNVEVNSKQLYWPFTGEKQYQADDLKLKLTGKMTDYTLSMRTAVKGQEI

400   PPATITLDAKGNEQQVNLDKLTVAALEGKTELKALLDWQQAISWRGELTL

450   NGINTAKEFPDWPSKLNGLIKTRGSLYGGTWQMDVPELKLTGNVKQNKVN

500   VDGTLKGNSYMQWMIPGLHLELGPNSAEVKGELGVKDLNLDATINAPGLD

550   NALPGLGGTAKGLVKVRGTVEAPQLLADITARGLRWQELSVAQVRVEGDI

600   KSTDQIAGKLDVRVEQISQPDVNINLVTLNAKGSEKQHELQLRIQGEPVS

650   GQLNLAGSFDRKEERWKGTLSNTRFQTPVGPWSLTRDIALDYRNKEQKIS

700   IGPHCWLNPNAELCVPQTIDAGAEGRAVVNLNRFDLAMLKPFMPETTQAS

750   GIFTGKADVAWDTTKEGLPQGSITLSGRNVQVTQTVNDAALPVAFQTLNL

800   TAELRNNRAELGWTIRLTNNGQFDGQVQVTDPQGRRNLGGNVNIRNFNLA

850   MINPIFTRGEKAAGMVSANLRLGGDVQSPQLFGQLQVTGVDIDGNFMPFD

900   MQPSQLAVNFNGMRSTLAGTVRTQQGEIYLNGDADWSQIENWRARVTAKG
```

950   SKVRITVPPMVRMDVSPDVVFEATPNLFTLDGRVDVPWARIVVHDLPESA

1000 VGVSSDVVMLNDNLQPEEPKTASIPINSNLIVHVGNNVRIDAFGLKARLT

1050 GDLNVVQDKQGLGLNGQINIPEGRFHAYGQDLIVRKGELLFSGPPDQPYL

1100 NIEAIRNPDATEDDVIAGVRVTGLADEPKAEIFSDPAMSQQAALSYLLRG

1150 QGLESDQSDSAAMTSMLIGLGVAQSGQIVGKIGETFGVSNLALDTQGVGD

1200 SSQVVVSGYVLPGLQVKYGVGIFDSIATLTLRYRLMPKLYLEAVSGVDQA

1250 LDLLYQFEF

sp|P76440|PRET_ECOLI NAD-dependent dihydropyrimidine dehydrogenase subunit PreT OS=Escherichia coli (strain K12) OX=83333 GN=preT PE=1 SV=1

0   MPQQNYLDELTPAFTSLLAIKEASRCLLCHDAPCSQACPAQTDPGKFIRS

50   IYFRNFKGAAETIRENNALGAVCARVCPTEKLCQSGCTRAGVDAPIDIGR

100   LQRFVTDFEQQTGMEIYQPGTKTLGKVAIIGAGPAGLQASVTLTNQGYDV

150   TIYEKEAHPGGWLRNGIPQFRLPQSVLDAEIARIEKMGVTIKCNNEVGNT

200   LTLEQLKAENRAVLVTVGLSSGSGLPLFEHSDVEIAVDFLQRARQAQGDI
****
250   SIPQSALIIGGGDVAMDVASTLKVLGCQAVTCVAREELDEFPASEKEFTS
*****
300   ARELGVSIIDGFTPVAVEGNKVTFKHVRLSGELTMAADKIILAVGQHARL

350   DAFAELEPQRNTIKTQNYQTRDPQVFAAGDIVEGDKTVVYAVKTGKEAAE

400   AIHHYLEGACSC

sp|P24555|PTRB_ECOLI Protease 2 OS=Escherichia coli (strain K12) OX=83333 GN=ptrB PE=1 SV=2

0   MLPKAARIPHAMTLHGDTRIDNYYWLRDDTRSQPEVLDYLQQENSYGHRV

50   MASQQALQDRILKEIIDRIPQREVSAPYIKNGYRYRHIYEPGCEYAIYQR

100   QSAFSEEWDEWETLLDANKRAAHSEFYSMGGMAITPDNTIMALAEDFLSR

150   RQYGIRFRNLETGNWYPELLDNVEPSFVWANDSWIFYYVRKHPVTLLPYQ
****
200   VWRHAIGTPASQDKLIYEEKDDTYYVSLHKTTSKHYVVIHLASATTSEVR

250   LLDAEMADAEPFVFLPRRKDHEYSLDHYQHRFYLRSNRHGKNFGLYRTRM

300   RDEQQWEELIPPRENIMLEGFTLFTDWLVVEERQRGLTSLRQINRKTREV

350   IGIAFDDPAYVTWIAYNPEPETARLRYGYSSMTTPDTLFELDMDTGERRV

```
400   LKQTEVPGFYAANYRSEHLWIVARDGVEVPVSLVYHRKHFRKGHNPLLVY

450   GYGSYGASIDADFSFSRLSLLDRGFVYAIVHVRGGGELGQQWYEDGKFLK

500   KKNTFNDYLDACDALLKLGYGSPSLCYAMGGSAGGMLMGVAINQRPELFH

550   GVIAQVPFVDVVTTMLDESIPLTTGEFEEWGNPQDPQYYEYMKSYSPYDN

600   VTAQAYPHLLVTTGLHDSQVQYWEPAKWVAKLRELKTDDHLLLLCTDMDS

650   GHGGKSGRFKSYEGVAMEYAFLVALAQGTLPATPAD

sp|P0AAV6|YBGS_ECOLI Uncharacterized protein YbgS OS=Escherichia coli
(strain K12) OX=83333 GN=ybgS PE=3 SV=1

0     MKMTKLATLFLTATLSLASGAALAADSGAQTNNGQANAAADAGQVAPDAR
                           **********************
50    ENVAPNNVDNNGVNTGSGGTMLHSDGSSMNNDGMTKDEEHKNTMCKDGRC

100   PDINKKVQTGDGINNDVDTKTDGTTQ

sp|P77585|YPDE_ECOLI Aminopeptidase YpdE OS=Escherichia coli (strain K12)
OX=83333 GN=ypdE PE=1 SV=1
       ********************
0     MDLSLLKALSEADAIASSEQEVRQILLEEADRLQKEVRFDGLGSVLIRLN

50    ESTGPKVMICAHMDEVGFMVRSISREGAIDVLPVGNVRMAARQLQPVRIT

100   TREECKIPGLLDGDRQGNDVSAMRVDIGARSYDEVMQAGIRPGDRVTFDT

150   TFQVLPHQRVMGKAFDDRLGCYLLVTLLRELHDAELPAEVWLVASSSEEV

200   GLRGGQTATRAVSPDVAIVLDTACWAKNFDYGAANHRQIGNGPMLVLSDK

250   SLIAPPKLTAWVETVAAEIGVPLQADMFSNGGTDGGAVHLTGTGVPTVVM

300   GPATRHGHCAASIADCRDILQMQQLLSALIQRLTRETVVQLTDFR

sp|P63201|GADW_ECOLI HTH-type transcriptional regulator GadW
OS=Escherichia coli (strain K12) OX=83333 GN=gadW PE=1 SV=1

0     MTHVCSVILIRRSFDIYHEQQKISLHNESILLLEKNLADDFAFCSPDTRR

50    LDIDELTVCHYLQNIRQLPRNLGLHSKDRLLINQSPPMPLVTAIFDSFNE

100   SGVNSPILSNMLYLSCLSMFSHKKELIPLLFNSISTVSGKVERLISFDIA
            ****************************
150   KRWYLRDIAERMYTSESLIKKKLQDENTCFSKILLASRMSMARRLLELRQ

200   IPLHTIAEKCGYSSTSYFINTFRQYYGVTPHQFAQHSPGTFS

sp|P0AAR0|TOMB_ECOLI Hha toxicity modulator TomB OS=Escherichia coli
(strain K12) OX=83333 GN=tomB PE=1 SV=1
```

```
0     MDEYSPKRHDIAQLKFLCETLYHDCLANLEESNHGWVNDPTSAINLQLNE
                  ********************
50    LIEHIATFALNYKIKYNEDNKLIEQIDEYLDDTFMLFSSYGINMQDLQKW

100   RKSGNRLFRCFVNATKENPASLSC
```

sp|P75822|YBJT_ECOLI Putative NAD(P)-binding protein YbjT OS=Escherichia coli (strain K12) OX=83333 GN=ybjT PE=3 SV=2

```
0     MPQRILVLGASGYIGQHLVRTLSQQGHQILAAARHVDRLAKLQLANVSCH

50    KVDLSWPDNLPALLQDIDTVYFLVHSMGEGGDFIAQERQVALNVRDALRE

100   VPVKQLIFLSSLQAPPHEQSDHLRARQATADILREANVPVTELRAGIIVG

150   AGSAAFEVMRDMVYNLPVLTPPRWVRSRTTPIALENLLHYLVALLDHPAS

200   EHRIFEAAGPEVLSYQQQFEHFMAVSGKRRWLIPIPLPTRWISVWFLNVI
                                         ***************
250   TSVPPTTARALIQGLKHDLLADDTALRALIPQRLIAFDDAVRSTLKEEEK
      ************
300   LVNSSDWGYDAQAFARWRPEYGYFAKQAGFTVKTSASLAALWQVVNQIGG

350   KERYFFGNILWQTRALMDRAIGHKLAKGRPEREYLQTGDAVDSWKVIVVE

400   PEKQLTLLFGMKAPGLGRLCFSLEDKGDYRTIDVRAFWHPHGMPGLFYWL

450   LMIPAHLFIFRGMAKQIARLAEQSTD
```

sp|P15639|PUR9_ECOLI Bifunctional purine biosynthesis protein PurH OS=Escherichia coli (strain K12) OX=83333 GN=purH PE=1 SV=1

```
0     MQQRRPVRRALLSVSDKAGIVEFAQALSARGVELLSTGGTARLLAEKGLP

50    VTEVSDYTGFPEMMDGRVKTLHPKVHGGILGRRGQDDAIMEEHQIQPIDM
                                               *********
100   VVVNLYPFAQTVAREGCSLEDAVENIDIGGPTMVRSAAKNHKDVAIVVKS
      *******************
150   SDYDAIIKEMDDNEGSLTLATRFDLAIKAFEHTAAYDSMIANYFGSMVPA

200   YHGESKEAAGRFPRTLNLNFIKKLDMRYGENSHQQAAFYIEENVKEASVA

250   TATQVQGKALSYNNIADTDAALECVKEFAEPACVIVKHANPCGVAIGNSI

300   LDAYDRAYKTDPTSAFGGIIAFNRELDAETAQAIISRQFVEVIIAPSASE

350   EALKITAAKQNVRVLTCGQWGERVPGLDFKRVNGGLLVQDRDLGMVGAEE

400   LRVVTKRQPSEQELRDALFCWKVAKFVKSNAIVYAKNNMTIGIGAGQMSR

450   VYSAKIAGIKAADEGLEVKGSSMASDAFFPFRDGIDAAAAAGVTCVIQPG

500   GSIRDDEVIAAADEHGIAMLFTDMRHFRH
```

```
sp|P0A9J0|RNG_ECOLI Ribonuclease G OS=Escherichia coli (strain K12)
OX=83333 GN=rng PE=1 SV=2

0     MTAELLVNVTPSETRVAYIDGGILQEIHIEREARRGIVGNIYKGRVSRVL

50    PGMQAAFVDIGLDKAAFLHASDIMPHTECVAGEEQKQFTVRDISELVRQG
                                          **************
100   QDLMVQVVKDPLGTKGARLTTDITLPSRYLVFMPGASHVGVSQRIESESE
      ****************
150   RERLKKVVAEYCDEQGGFIIRTAAEGVGEAELASDAAYLKRVWTKVMERK

200   KRPQTRYQLYGELALAQRVLRDFADAELDRIRVDSRLTYEALLEFTSEYI

250   PEMTSKLEHYTGRQPIFDLFDVENEIQRALERKVELKSGGYLIIDQTEAM

300   TTVDINTGAFVGHRNLDDTIFNTNIEATQAIARQLRLRNLGGIIIIDFID

350   MNNEDHRRRVLHSLEQALSKDRVKTSVNGFSALGLVEMTRKRTRESIEHV

400   LCNECPTCHGRGTVKTVETVCYEIMREIVRVHHAYDSDRFLVYASPAVAE

450   ALKGEESHSLAEVEIFVGKQVKVQIEPLYNQEQFDVVMM

sp|P77658|YNAA_ECOLI Protein YnaA OS=Escherichia coli (strain K12)
OX=83333 GN=ynaA PE=4 SV=1

0     MAEQTSRLAIIIDSTGAKNNADNLTSSLVKMTQAGETAANSAGKVTKATE
                             ***************************
50    DEKNALAKLKAAIDPVGAAIDTVGRRYSELKKFFDKGLIDKEEYEFLVRK
      *********
100   LNETTEELSGVAQAQREAEKAGKLAAAQQEAQAQAFQRMLDKIDPLAAAL

150   RNLEQQHDELNAAFASGKINGSQFENYSRKIQETRRELTGEAQAEREAAK

200   AHDEQVVALQRLIAQLDPVGTAFNRLVEQQKQLNEAKAKGMLSPEMYEEL

250   SGKLRAMRSELEVTQSQLSKTGMSAKQTAFAMRMLPAQMTDIVVGLSTGQ

300   SPFMVLMQQGGHSAMQETRIASLPVTAIPAGMKIPCVTAGSA

sp|P64499|YEBO_ECOLI Uncharacterized protein YebO OS=Escherichia coli
(strain K12) OX=83333 GN=yebO PE=4 SV=1

0     MNEVVNSGVMNIASLVVSVVVLLIGLILWFFINRASSRTNEQIELLEALL
                 *********************************
50    DQQKRQNALLRRLCEANEPEKADKKTVESQKSVEDEDIIRLVAER

sp|P00959|SYM_ECOLI Methionine--tRNA ligase OS=Escherichia coli (strain
K12) OX=83333 GN=metG PE=1 SV=2

0     MTQVAKKILVTCALPYANGSIHLGHMLEHIQADVWVRYQRMRGHEVNFIC

50    ADDAHGTPIMLKAQQLGITPEQMIGEMSQEHQTDFAGFNISYDNYHSTHS
```

```
100   EENRQLSELIYSRLKENGFIKNRTISQLYDPEKGMFLPDRFVKGTCPKCK

150   SPDQYGDNCEVCGATYSPTELIEPKSVVSGATPVMRDSEHFFFDLPSFSE

200   MLQAWTRSGALQEQVANKMQEWFESGLQQWDISRDAPYFGFEIPNAPGKY
                   ******************************
250   FYVWLDAPIGYMGSFKNLCDKRGDSVSFDEYWKKDSTAELYHFIGKDIVY

300   FHSLFWPAMLEGSNFRKPSNLFVHGYVTVNGAKMSKSRGTFIKASTWLNH

350   FDADSLRYYYTAKLSSRIDDIDLNLEDFVQRVNADIVNKVVNLASRNAGF

400   INKRFDGVLASELADPQLYKTFTDAAEVIGEAWESREFGKAVREIMALAD

450   LANRYVDEQAPWVVAKQEGRDADLQAICSMGINLFRVLMTYLKPVLPKLT

500   ERAEAFLNTELTWDGIQQPLLGHKVNPFKALYNRIDMRQVEALVEASKEE

550   VKAAAAPVTGPLADDPIQETITFDDFAKVDLRVALIENAEFVEGSDKLLR

600   LTLDLGGEKRNVFSGIRSAYPDPQALIGRHTIMVANLAPRKMRFGISEGM

650   VMAAGPGGKDIFLLSPDAGAKPGHQVK

sp|P0C066|MLTC_ECOLI Membrane-bound lytic murein transglycosylase C
OS=Escherichia coli (strain K12) OX=83333 GN=mltC PE=1 SV=1

0     MKKYLALALIAPLLISCSTTKKGDTYNEAWVKDTNGFDILMGQFAHNIEN

50    IWGFKEVVIAGPKDYVKYTDQYQTRSHINFDDGTITIETIAGTEPAAHLR
          ******************************
100   RAIIKTLLMGDDPSSVDLYSDVDDITISKEPFLYGQVVDNTGQPIRWEGR

150   ASNFADYLLKNRLKSRSNGLRIIYSVTINMVPNHLDKRAHKYLGMVRQAS

200   RKYGVDESLILAIMQTESSFNPYAVSRSDALGLMQVVQHTAGKDVFRSQG

250   KSGTPSRSFLFDPASNIDTGTAYLAMLNNVYLGGIDNPTSRRYAVITAYN

300   GGAGSVLRVFSNDKIQAANIINTMTPGDVYQTLTTRHPSAESRRYLYKVN

350   TAQKSYRRR

sp|P0AEW6|INGK_ECOLI Inosine-guanosine kinase OS=Escherichia coli (strain
K12) OX=83333 GN=gsk PE=1 SV=1

0     MKFPGKRKSKHYFPVNARDPLLQQFQPENETSAAWVVGIDQTLVDIEAKV

50    DDEFIERYGLSAGHSLVIEDDVAEALYQELKQKNLITHQFAGGTIGNTMH

100   NYSVLADDRSVLLGVMCSNIEIGSYAYRYLCNTSSRTDLNYLQGVDGPIG

150   RCFTLIGESGERTFAISPGHMNQLRAESIPEDVIAGASALVLTSYLVRCK
```

200   PGEPMPEATMKAIEYAKKYNVPVVLTLGTKFVIAENPQWWQQFLKDHVSI
         ****
250   LAMNEDEAEALTGESDPLLASDKALDWVDLVLCTAGPIGLYMAGFTEDEA

300   KRKTQHPLLPGAIAEFNQYEFSRAMRHKDCQNPLRVYSHIAPYMGGPEKI

350   MNTNGAGDGALAALLHDITANSYHRSNVPNSSKHKFTWLTYSSLAQVCKY

400   ANRVSYQVLNQHSPRLTRGLPEREDSLEESYWDR

sp|P77293|GTRB_ECOLI Prophage bactoprenol glucosyl transferase homolog OS=Escherichia coli (strain K12) OX=83333 GN=yfdH PE=1 SV=1
                              ****
0   MKISLVVPVFNEEEAIPIFYKTVREFEELKSYEVEIVFINDGSKDATESI

50   INALAVSDPLVVPLSFTRNFGKEPALFAGLDHATGDAIIPIDVDLQDPIE

100   VIPHLIEKWQAGADMVLAKRSDRSTDGRLKRKTAEWFYKLHNKISNPKIE

150   ENVGDFRLMSRDVVENIKLMPERNLFMKGILSWVGGKTDIVEYVRAERIA

200   GDTKFNGWKLWNLALEGITSFSTFPLRIWTYIGLVVASVAFIYGAWMILD

250   TIIFGNAVRGYPSLLVSILFLGGIQMIGIGVLGEYIGRTYIETKKRPKYI

300   IKRVKK

sp|P05100|3MG1_ECOLI DNA-3-methyladenine glycosylase 1 OS=Escherichia coli (strain K12) OX=83333 GN=tag PE=1 SV=1

0   MERCGWVSQDPLYIAYHDNEWGVPETDSKKLFEMICLEGQQAGLSWITVL

50   KKRENYRACFHQFDPVKVAAMQEEDVERLVQDAGIIRHRGKIQAIIGNAR
                                     ****
100   AYLQMEQNGEPFVDFVWSFVNHQPQVTQATTLSEIPTSTSASDALSKALK
      **
150   KRGFKFVGTTICYSFMQACGLVNDHVVGCCCYPGNKP

sp|P08997|MASY_ECOLI Malate synthase A OS=Escherichia coli (strain K12) OX=83333 GN=aceB PE=1 SV=1

0   MTEQATTTDELAFTRPYGEQEKQILTAEAVEFLTELVTHFTPQRNKLLAA

50   RIQQQQDIDNGTLPDFISETASIRDADWKIRGIPADLEDRRVEITGPVER

100   KMVINALNANVKVFMADFEDSLAPDWNKVIDGQINLRDAVNGTISYTNEA

150   GKIYQLKPNPAVLICRVRGLHLPEKHVTWRGEAIPGSLFDFALYFFHNYQ

200   ALLAKGSGPYFYLPKTQSWQEAAWWSEVFSYAEDRFNLPRGTIKATLLIE

250   TLPAVFQMDEILHALRDHIVGLNCGRWDYIFSYIKTLKNYPDRVLPDRQA
                                        ****
300   VTMDKPFLNAYSRLLIKTCHKRGAFAMGGMAAFIPSKDEEHNNQVLNKVK

```
      ******
350   ADKSLEANNGHDGTWIAHPGLADTAMAVFNDILGSRKNQLEVMREQDAPI

400   TADQLLAPCDGERTEEGMRANIRVAVQYIEAWISGNGCVPIYGLMEDAAT

450   AEISRTSIWQWIHHQKTLSNGKPVTKALFRQMLGEEMKVIASELGEERFS

500   QGRFDDAARLMEQITTSDELIDFLTLPGYRLLA
```

sp|P0AES4|GYRA_ECOLI DNA gyrase subunit A OS=Escherichia coli (strain K12) OX=83333 GN=gyrA PE=1 SV=1

```
0     MSDLAREITPVNIEEELKSSYLDYAMSVIVGRALPDVRDGLKPVHRRVLY

50    AMNVLGNDWNKAYKKSARVVGDVIGKYHPHGDSAVYDTIVRMAQPFSLRY

100   MLVDGQGNFGSIDGDSAAAMRYTEIRLAKIAHELMADLEKETVDFVDNYD

150   GTEKIPDVMPTKIPNLLVNGSSGIAVGMATNIPPHNLTEVINGCLAYIDD

200   EDISIEGLMEHIPGPDFPTAAIINGRRGIEEAYRTGRGKVYIRARAEVEV
                                 ***********************
250   DAKTGRETIIVHEIPYQVNKARLIEKIAELVKEKRVEGISALRDESDKDG
      **
300   MRIVIEVKRDAVGEVVLNNLYSQTQLQVSFGINMVALHHGQPKIMNLKDI

350   IAAFVRHRREVVTRRTIFELRKARDRAHILEALAVALANIDPIIELIRHA

400   PTPAEAKTALVANPWQLGNVAAMLERAGDDAARPEWLEPEFGVRDGLYYL

450   TEQQAQAILDLRLQKLTGLEHEKLLDEYKELLDQIAELLRILGSADRLME

500   VIREELELVREQFGDKRRTEITANSADINLEDLITQEDVVVTLSHQGYVK

550   YQPLSEYEAQRRGGKGKSAARIKEEDFIDRLLVANTHDHILCFSSRGRVY

600   SMKVYQLPEATRGARGRPIVNLLPLEQDERITAILPVTEFEEGVKVFMAT
                                                **********
650   ANGTVKKTVLTEFNRLRTAGKVAIKLVDGDELIGVDLTSGEDEVMLFSAE
      **********
700   GKVVRFKESSVRAMGCNTTGVRGIRLGEGDKVVSLIVPRGDGAILTATQN

750   GYGKRTAVAEYPTKSRATKGVISIKVTERNGLVVGAVQVDDCDQIMMITD

800   AGTLVRTRVSEISIVGRNTQGVILIRTAEDENVVGLQRVAEPVDEEDLDT

850   IDGSAAEGDDEIAPEVDVDDEPEEE
```

sp|P0A7J7|RL11_ECOLI 50S ribosomal protein L11 OS=Escherichia coli (strain K12) OX=83333 GN=rplK PE=1 SV=2

```
0     MAKKVQAYVKLQVAAGMANPSPPVGPALGQQGVNIMEFCKAFNAKTDSIE
                                    ********************
50    KGLPIPVVITVYADRSFTFVTKTPPAAVLLKKAAGIKSGSGKPNKDKVGK
```

100   ISRAQLQEIAQTKAADMTGADIEAMTRSIEGTARSMGLVVED

sp|P0AFQ7|YCFH_ECOLI Uncharacterized metal-dependent hydrolase YcfH OS=Escherichia coli (strain K12) OX=83333 GN=ycfH PE=1 SV=1

          ****
0   MFLVDSHCHLDGLDYESLHKDVDDVLAKAAARDVKFCLAVATTLPGYLHM

50   RDLVGERDNVVFSCGVHPLNQNDPYDVEDLRRLAAEEGVVALGETGLDYY

100   YTPETKVRQQESFIHHIQIGRELNKPVIVHTRDARADTLAILREEKVTDC

150   GGVLHCFTEDRETAGKLLDLGFYISFSGIVTFRNAEQLRDAARYVPLDRL

200   LVETDSPYLAPVPHRGKENQPAMVRDVAEYMAVLKGVAVEELAQVTTDNF

250   ARLFHIDASRLQSIR

sp|P06988|HISX_ECOLI Histidinol dehydrogenase OS=Escherichia coli (strain K12) OX=83333 GN=hisD PE=1 SV=5

0   MSFNTIIDWNSCTAEQQRQLLMRPAISASESITRTVNDILDNVKARGDEA
                    ****
50   LREYSAKFDKTTVTALKVSAEEIAAASERLSDELKQAMAVAVKNIETFHT

100   AQKLPPVDVETQPGVRCQQVTRPVASVGLYIPGGSAPLFSTVLMLATPAS

150   IAGCKKVVLCSPPPIADEILYAAQLCGVQDVFNVGGAQAIAALAFGTESV

200   PKVDKIFGPGNAFVTEAKRQVSQRLDGAAIDMPAGPSEVLVIADSGATPD

250   FVASDLLSQAEHGPDSQVILLTPAADMARRVAEAVERQLAELPRAETARQ

300   ALNASRLIVTKDLAQCVEISNQYGPEHLIIQTRNARELVDSITSAGSVFL

350   GDWSPESAGDYASGTNHVLPTYGYTATCSSLGLADFQKRMTVQELSKEGF

400   SALASTIETLAAAERLTAHKNAVTLRVNALKEQA

sp|P75990|BLUF_ECOLI Blue light- and temperature-regulated antirepressor BluF OS=Escherichia coli (strain K12) OX=83333 GN=bluF PE=1 SV=1

0   MLTTLIYRSHIRDDEPVKKIEEMVSIANRRNMQSDVTGILLFNGSHFFQL

50   LEGPEEQVKMIYRAICQDPRHYNIVELLCDYAPARRFGKAGMELFDLRLH
                                                ****
100   ERDDVLQAVFDKGTSKFQLTYDDRALQFFRTFVLATEQSTYFEIPAEDSW
      ****
150   LFIADGSDKELDSCALSPTINDHFAFHPIVDPLSRRIIAFEAIVQKNEDS

200   PSAIAVGQRKDGEIYTADLKSKALAFTMAHALELGDKMISINLLPMTLVN
                              ****
250   EPDAVSFLLNEIKANALVPEQIIVEFTESEVISRFDEFAEAIKSLKAAGI

300 SVAIDHFGAGFAGLLLLSRFQPDRIKISQELITNVHKSGPRQAIIQAIIK

350 CCTSLEIQVSAMGVATPEEWMWLESAGIEMFQGDLFAKAKLNGIPSIAWP

400 EKK

sp|P06959|ODP2_ECOLI Dihydrolipoyllysine-residue acetyltransferase component of pyruvate dehydrogenase complex OS=Escherichia coli (strain K12) OX=83333 GN=aceF PE=1 SV=3

****

0 MAIEIKVPDIGADEVEITEILVKVGDKVEAEQSLITVEGDKASMEVPSPQ

50 AGIVKEIKVSVGDKTQTGALIMIFDSADGAADAAPAQAEEKKEAAPAAAP

****

100 AAAAAKDVNVPDIGSDEVEVTEILVKVGDKVEAEQSLITVEGDKASMEVP

150 APFAGTVKEIKVNVGDKVSTGSLIMVFEVAGEAGAAAPAAKQEAAPAAAP

200 APAAGVKEVNVPDIGGDEVEVTEVMVKVGDKVAAEQSLITVEGDKASMEV

250 PAPFAGVVKELKVNVGDKVKTGSLIMIFEVEGAAPAAAPAKQEAAAPAPA

300 AKAEAPAAAPAAKAEGKSEFAENDAYVHATPLIRRLAREFGVNLAKVKGT

350 GRKGRILREDVQAYVKEAIKRAEAAPAATGGGIPGMLPWPKVDFSKFGEI

400 EEVELGRIQKISGANLSRNWVMIPHVTHFDKTDITELEAFRKQQNEEAAK

450 RKLDVKITPVVFIMKAVAAALEQMPRFNSSLSEDGQRLTLKKYINIGVAV

500 DTPNGLVVPVFKDVNKKGIIELSRELMTISKKARDGKLTAGEMQGGCFTI

550 SSIGGLGTTHFAPIVNAPEVAILGVSKSAMEPVWNGKEFVPRLMLPISLS

600 FDHRVIDGADGARFITIINNTLSDIRRLVM

sp|P0AFP2|ATL_ECOLI DNA base-flipping protein OS=Escherichia coli (strain K12) OX=83333 GN=atl PE=1 SV=1

****

0 MLVSCAMRLHSGVFPDYAEKLPQEEKMEKEDSFPQRVWQIVAAIPEGYVT

50 TYGDVAKLAGSPRAARQVGGVLKRLPEGSTLPWHRVVNRHGTISLTGPDL

100 QRQRQALLAEGVMVSGSGQIDLQRYRWNY

sp|P76550|YFFS_ECOLI Uncharacterized protein YffS OS=Escherichia coli (strain K12) OX=83333 GN=yffS PE=4 SV=2

0 MSYEIKICDILKGAAMEGQYKGAQRGAKCEEIANELTRRGVKNNKGEVIT

50 KGGVSHWLEGRREPNFDTLAELCDMFGVYALMPMRGGKWIRVHPEDRGEM

100 ELREAVAERDAIIDDLKARIAELEAALANKQVPAEAEEMGGEKVEEVAAE

150   QAPNDEKEMGAKEWVNPNPKKYSVGMLCQVLAAMGGEYLGNNAGLQQKIT
                                     ****
200   VLDNDGNRKPISNGAFYRLIEQAKGRGLISVEQEIKHKKDENGNQIGKGK
      *****
250   KGDKLITLLPNWIDKLGDE

sp|P0ABD5|ACCA_ECOLI Acetyl-coenzyme A carboxylase carboxyl transferase subunit alpha OS=Escherichia coli (strain K12) OX=83333 GN=accA PE=1 SV=2
                   ****
0   MSLNFLDFEQPIAELEAKIDSLTAVSRQDEKLDINIDEEVHRLREKSVEL

50   TRKIFADLGAWQIAQLARHPQRPYTLDYVRLAFDEFDELAGDRAYADDKA

100   IVGGIARLDGRPVMIIGHQKGRETKEKIRRNFGMPAPEGYRKALRLMQMA

150   ERFKMPIITFIDTPGAYPGVGAEERGQSEAIARNLREMSRLGVPVVCTVI

200   GEGGSGGALAIGVGDKVNMLQYSTYSVISPEGCASILWKSADKAPLAAEA

250   MGIIAPRLKELKLIDSIIPEPLGGAHRNPEAMAASLKAQLLADLADLDVL

300   STEDLKNRRYQRLMSYGYA

sp|P19926|AGP_ECOLI Glucose-1-phosphatase OS=Escherichia coli (strain K12) OX=83333 GN=agp PE=1 SV=1

0   MNKTLIAAAVAGIVLLASNAQAQTVPEGYQLQQVLMMSRHNLRAPLANNG

50   SVLEQSTPNKWPEWDVPGGQLTTKGGVLEVYMGHYMREWLAEQGMVKSGE

100   CPPPYTVYAYANSLQRTVATAQFFITGAFPGCDIPVHHQEKMGTMDPTFN
          ****
150   PVITDDSAAFSEQAVAAMEKELSKLQLTDSYQLLEKIVNYKDSPACKEKQ

200   QCSLVDGKNTFSAKYQQEPGVSGPLKVGNSLVDAFTLQYYEGFPMDQVAW

250   GEIKSDQQWKVLSKLKNGYQDSLFTSPEVARNVAKPLVSYIDKALVTDRT

300   SAPKITVLVGHDSNIASLLTALDFKPYQLHDQNERTPIGGKIVFQRWHDS

350   KANRDLMKIEYVYQSAEQLRNADALTLQAPAQRVTLELSGCPIDADGFCP

400   MDKFDSVLNEAVK

sp|P07648|RECC_ECOLI RecBCD enzyme subunit RecC OS=Escherichia coli (strain K12) OX=83333 GN=recC PE=1 SV=1

0   MLRVYHSNRLDVLEALMEFIVERERLDDPFEPEMILVQSTGMAQWLQMTL

50   SQKFGIAANIDFPLPASFIWDMFVRVLPEIPKESAFNKQSMSWKLMTLLP
                  ****
100   QLLEREDFTLLRHYLTDDSDKRKLFQLSSKAADLFDQYLVYRPDWLAQWE

150   TGHLVEGLGEAQAWQAPLWKALVEYTHQLGQPRWHRANLYQRFIETLESA

```
200   TTCPPGLPSRVFICGISALPPVYLQALQALGKHIEIHLLFTNPCRYYWGD

250   IKDPAYLAKLLTRQRRHSFEDRELPLFRDSENAGQLFNSDGEQDVGNPLL

300   ASWGKLGRDYIYLLSDLESSQELDAFVDVTPDNLLHNIQSDILELENRAV
         **********************
350   AGVNIEEFSRSDNKRPLDPLDSSITFHVCHSPQREVEVLHDRLLAMLEED

400   PTLTPRDIIVMVADIDSYSPFIQAVFGSAPADRYLPYAISDRRARQSHPV

450   LEAFISLLSLPDSRFVSEDVLALLDVPVLAARFDITEEGLRYLRQWVNES

500   GIRWGIDDDNVRELELPATGQHTWRFGLTRMLLGYAMESAQGEWQSVLPY

550   DESSGLIAELVGHLASLLMQLNIWRRGLAQERPLEEWLPVCRDMLNAFFL

600   PDAETEAAMTLIEQQWQAIIAEGLGAQYGDAVPLSLLRDELAQRLDQERI

650   SQRFLAGPVNICTLMPMRSIPFKVVCLLGMNDGVYPRQLAPLGFDLMSQK

700   PKRGDRSRRDDDRYLFLEALISAQQKLYISYIGRSIQDNSERFPSVLVQE

750   LIDYIGQSHYLPGDEALNCDESEARVKAHLTCLHTRMPFDPQNYQPGERQ

800   SYAREWLPAASQAGKAHSEFVQPLPFTLPETVPLETLQRFWAHPVRAFFQ

850   MRLQVNFRTEDSEIPDTEPFILEGLSRYQINQQLLNALVEQDDAERLFRR

900   FRAAGDLPYGAFGEIFWETQCQEMQQLADRVIACRQPGQSMEIDLACNGV

950   QITGWLPQVQPDGLLRWRPSLLSVAQGMQLWLEHLVYCASGGNGESRLFL

1000  RKDGEWRFPPLAAEQALHYLSQLIEGYREGMSAPLLVLPESGGAWLKTCY

1050  DAQNDAMLDDDSTLQKARTKFLQAYEGNMMVRGEGDDIWYQRLWRQLTPE

1100  TMEAIVEQSQRFLLPLFRFNQS

sp|P42913|YRAH_ECOLI Uncharacterized fimbrial-like protein YraH
OS=Escherichia coli (strain K12) OX=83333 GN=yraH PE=2 SV=1
                                 *************************
0     MNKVTKTAIAGLLALFAGNAAATDGEIVFDGEILKSACEINDSDKKIEVA
      *
50    LGHYNAEQFRNIGERSPKIPFTIPLVNCPMTGWEHDNGNVEASFRLWLET

100   RDNGTVPNFPNLAKVGSFAGIAATGVGIRIDDAESGNIMPLNAMGNDNTV

150   YQIPAESNGIVNVDLIAYYVSTVVPSEITPGEADAIVNVTLDYR

sp|P37634|RLMJ_ECOLI Ribosomal RNA large subunit methyltransferase J
OS=Escherichia coli (strain K12) OX=83333 GN=rlmJ PE=1 SV=1
                        ********************
0     MLSYRHSFHAGNHADVLKHTVQSLIIESLKEKDKPFLYLDTHAGAGRYQL
```

```
50    GSEHAERTGEYLEGIARIWQQDDLPAELEAYINVVKHFNRSGQLRYYPGS

100   PLIARLLLREQDSLQLTELHPSDYPLLRSEFQKDSRARVEKADGFQQLKA

150   KLPPVSRRGLILIDPPYEMKTDYQAVVSGIAEGYKRFATGIYALWYPVVL

200   RQQIKRMIHDLEATGIRKILQIELAVLPDSDRRGMTASGMIVINPPWKLE

250   QQMNNVLPWLHSKLVPAGTGHATVSWIVPE
```

sp|P0AGL7|RSME_ECOLI Ribosomal RNA small subunit methyltransferase E OS=Escherichia coli (strain K12) OX=83333 GN=rsmE PE=1 SV=1

```
                                                 *******
0     MRIPRIYHPEPLTSHSHIALCEDAANHIGRVLRMGPGQALQLFDGSNQVF
      ****************************
50    DAEITSASKKSVEVKVLEGQIDDRESPLHIHLGQVMSRGEKMEFTIQKSI

100   ELGVSLITPLFSERCGVKLDSERLNKKLQQWQKIAIAACEQCGRNRVPEI

150   RPAMDLEAWCAEQDEGLKLNLHPRASNSINTLPLPVERVRLLIGPEGGLS

200   ADEIAMTARYQFTDILLGPRVLRTETTALTAITALQVRFGDLG
```

sp|P77374|YNFE_ECOLI Putative dimethyl sulfoxide reductase chain YnfE OS=Escherichia coli (strain K12) OX=83333 GN=ynfE PE=1 SV=1

```
0     MSKNERMVGISRRTLVKSTAIGSLALAAGGFSLPFTLRNAAAAVQQAREK

50    VVWGACSVNCGSRCALRLHVKDNEVTWVETDNTGSDEYGNHQVRACLRGR
                               ********************
100   SIRRRINHPDRLNYPMKRVGKRGEGKFERISWDEALDTIASSLKKTVEQY

150   GNEAVYIQYSSGIVGGNMTRSSPSASAVKRLMNCYGGSLNQYGSYSTAQI

200   SCAMPYTYGSNDGNSTTDIENSKLVVMFGNNPAETRMSGGGITYLLEKAR

250   EKSNAKMIVIDPRYTDTAAGREDEWLPIRPGTDAALVAGIAWVLINENLV

300   DQPFLDKYCVGYDEKTLPADAPKNGHYKAYILGEGDDKTAKTPQWASQIT

350   GIPEDRIIKLAREIGTAKPAYICQGWGPQRQANGELTARAIAMLPILTGN

400   VGISGGNSGARESTYTITIERLPVLDNPVKTSISCFSWTDAIDHGPQMTA

450   IRDGVRGKDKLDVPIKFIWNYAGNTLVNQHSDINKTHEILQDESKCEMIV

500   VIENFMTSSAKYADILLPDLMTVEQEDIIPNDYAGNMGYLIFLQPVTSEK

550   FERKPIYWILSEVAKRLGPDVYQKFTEGRTQEQWLQHLYAKMLAKDPALP

600   SYDELKKMGIYKRKDPNGHFVAYKAFRDDPEANPLKTPSGKIEIYSSRLA

650   EIARTWELEKDEVISPLPVYASTFEGWNSPERRTFPLQLFGFHYKSRTHS
```

700   TYGNIDLLKAACRQEVWINPIDAQKRGIANGDMVRVFNHRGEVRLPAKVT

750   PRILPGVSAMGQGAWHEANMSGDKIDHGGCVNTLTTLRPSPLAKGNPQHT

800   NLVEIEKI

sp|P0A7I4|RF3_ECOLI Peptide chain release factor RF3 OS=Escherichia coli (strain K12) OX=83333 GN=prfC PE=1 SV=2

0   MTLSPYLQEVAKRRTFAIISHPDAGKTTITEKVLLFGQAIQTAGTVKGRG

50   SNQHAKSDWMEMEKQRGISITTSVMQFPYHDCLVNLLDTPGHEDFSEDTY

100   RTLTAVDCCLMVIDAAKGVEDRTRKLMEVTRLRDTPILTFMNKLDRDIRD

150   PMELLDEVENELKIGCAPITWPIGCGKLFKGVYHLYKDETYLYQSGKGHT

200   IQEVRIVKGLNNPDLDAAVGEDLAQQLRDELELVKGASNEFDKELFLAGE

250   ITPVFFGTALGNFGVDHMLDGLVEWAPAPMPRQTDTRTVEASEDKFTGFV

300   FKIQANMDPKHRDRVAFMRVVSGKYEKGMKLRQVRTAKDVVISDALTFMA

350   GDRSHVEEAYPGDILGLHNHGTIQIGDTFTQGEMMKFTGIPNFAPELFRR

400   IRLKDPLKQKQLLKGLVQLSEEGAVQVFRPISNNDLIVGAVGVLQFDVVV
****
450   ARLKSEYNVEAVYESVNVATARWVECADAKKFEEFKRKNESQLALDGGDN

500   LAYIATSMVNLRLAQERYPDVQFHQTREH

sp|P0A6T1|G6PI_ECOLI Glucose-6-phosphate isomerase OS=Escherichia coli (strain K12) OX=83333 GN=pgi PE=1 SV=1

0   MKNINPTQTAAWQALQKHFDEMKDVTIADLFAKDGDRFSKFSATFDDQML

50   VDYSKNRITEETLAKLQDLAKECDLAGAIKSMFSGEKINRTENRAVLHVA

100   LRNRSNTPILVDGKDVMPEVNAVLEKMKTFSEAIISGEWKGYTGKAITDV

150   VNIGIGGSDLGPYMVTEALRPYKNHLNMHFVSNVDGTHIAEVLKKVNPET

200   TLFLVASKTFTTQETMTNAHSARDWFLKAAGDEKHVAKHFAALSTNAKAV

250   GEFGIDTANMFEFWDWVGGRYSLWSAIGLSIVLSIGFDNFVELLSGAHAM

300   DKHFSTTPAEKNLPVLLALIGIWYNNFFGAETEAILPYDQYMHRFAAYFQ

350   QGNMESNGKYVDRNGNVVDYQTGPIIWGEPGTNGQHAFYQLIHQGTKMVP

400   CDFIAPAITHNPLSDHHQKLLSNFFAQTEALAFGKSREVVEQEYRDQGKD

450   PATLDYVVPFKVFEGNRPTNSILLREITPFSLGALIALYEHKIFTQGVIL

```
                     ******************************
500   NIFTFDQWGVELGKQLANRILPELKDDKEISSHDSSTNGLINRYKAWRG
```

sp|P0AFB1|NLPI_ECOLI Lipoprotein NlpI OS=Escherichia coli (strain K12) OX=83333 GN=nlpI PE=1 SV=1

```
0     MKPFLRWCFVATALTLAGCSNTSWRKSEVLAVPLQPTLQQEVILARMEQI

50    LASRALTDDERAQLLYERGVLYDSLGLRALARNDFSQALAIRPDMPEVFN

100   YLGIYLTQAGNFDAAYEAFDSVLELDPTYNYAHLNRGIALYYGGRDKLAQ
                            ***************************
150   DDLLAFYQDDPNDPFRSLWLYLAEQKLDEKQAKEVLKQHFEKSDKEQWGW

200   NIVEFYLGNISEQTLMERLKADATDNTSLAEHLSETNFYLGKYYLSLGDL

250   DSATALFKLAVANNVHNFVEHRYALLELSLLGQDQDDLAESDQQ
```

sp|P76573|YFGI_ECOLI Uncharacterized protein YfgI OS=Escherichia coli (strain K12) OX=83333 GN=yfgI PE=4 SV=1

```
0     MKKVFLCAILASLSYPAIASSLQDQLSAVAEAEQQGKNEEQRQHDEWVAE
                                ************************
50    RNREIQQEKQRRANAQAAANKRAATAAANKKARQDKLDAEASADKKRDQS
      **************************************
100   YEDELRSLEIQKQKLALAKEEARVKRENEFIDQELKHKAAQTDVVQSEAD
           ************************
150   ANRNMTEGGRDLMKSVGKAEENKSDSWFN
```

sp|P27297|BAX_ECOLI Protein bax OS=Escherichia coli (strain K12) OX=83333 GN=bax PE=4 SV=3

```
                                       *****************
0     MILTPIRRYGAMILMLLTLVFSSEVLAKTHTTTASQKSHLTKASNKQVSS
      **********************
50    KQEYSRNSAKSSSLPDLRKYPSGTPRKKAFLRTVMPYITSQNAAITAERN

100   WLISKQYQGQWSPAERARLKDIAKRYKVKWSGNTRKIPWNTLLERVDIIP

150   TSMVATMAAAESGWGTSKLARNNNNLFGMKCMKGRCTNAPGKVKGYSQFS

200   SVKESVSAYVTNLNTHPAYSSFRKSRAQLRKADQEVTATAMIHKLKGYST

250   KGKSYNNYLFAMYQDNQRLIAAHM
```

sp|P0ACF8|HNS_ECOLI DNA-binding protein H-NS OS=Escherichia coli (strain K12) OX=83333 GN=hns PE=1 SV=2

```
                               *********************
0     MSEALKILNNIRTLRAQARECTLETLEEMLEKLEVVVNERREEESAAAAE

50    VEERTRKLQQYREMLIADGIDPNELLNSLAAVKSGTKAKRAQRPAKYSYV

100   DENGETKTWTGQGRTPAVIKKAMDEQGKSLDDFLIKQ
```

sp|P0A6H1|CLPX_ECOLI ATP-dependent Clp protease ATP-binding subunit ClpX OS=Escherichia coli (strain K12) OX=83333 GN=clpX PE=1 SV=2

```
0     MTDKRKDGSGKLLYCSFCGKSQHEVRKLIAGPSVYICDECVDLCNDIIRE

50    EIKEVAPHRERSALPTPHEIRNHLDDYVIGQEQAKKVLAVAVYNHYKRLR

100   NGDTSNGVELGKSNILLIGPTGSGKTLLAETLARLLDVPFTMADATTLTE
                                     *******************
150   AGYVGEDVENIIQKLLQKCDYDVQKAQRGIVYIDEIDKISRKSDNPSITR
      ******
200   DVSGEGVQQALLKLIEGTVAAVPPQGGRKHPQQEFLQVDTSKILFICGGA
                             ************************
250   FAGLDKVISHRVETGSGIGFGATVKAKSDKASEGELLAQVEPEDLIKFGL

300   IPEFIGRLPVVATLNELSEEALIQILKEPKNALTKQYQALFNLEGVDLEF
                                     *******************
350   RDEALDAIAKKAMARKTGARGLRSIVEAALLDTMYDLPSMEDVEKVVIDE
      **********
400   SVIDGQSKPLLIYGKPEAQQASGE
```

sp|P45807|YBAM_ECOLI Uncharacterized protein YbaM OS=Escherichia coli (strain K12) OX=83333 GN=ybaM PE=4 SV=1

```
                                      ******************
0     MSLENAPDDVKLAVDLIVLLEENQIPASTVLRALDIVKRDYEKKLTRDDE
      ***
50    AEK
```

sp|P43674|YCAL_ECOLI Metalloprotease YcaL OS=Escherichia coli (strain K12) OX=83333 GN=ycaL PE=1 SV=3

```
0     MKNTKLLLAIATSAALLTGCQNTHGIDTNMAISSGLNAYKAATLSDADAK

50    AIANQGCAEMDSGNQVASKSSKYGKRLAKIAKALGNNINGTPVNYKVYMT

100   SDVNAWAMANGCVRVYSGLMDMMNDNEIEGVLGHELGHVALGHSLAEMKA
                                 ***********************
150   SYAIVAARDAISATSGVASQLSRSQLGDIAEGAINAKYSRDKESEADDFS
      ************
200   FDLLKKRGISTQGLVGSFETLASLDGGRTQSMFDSHPPSTERAQHIRDRI

250   ASGK
```

sp|P77339|YAFT_ECOLI Uncharacterized lipoprotein YafT OS=Escherichia coli (strain K12) OX=83333 GN=yafT PE=3 SV=1

```
                ********************************
0     MNSKKLCCICVLFSLLAGCASESSIDEKKKKAQVTQSNINKNTPQQLTDK

50    DLFGNETTLAVSEEDIQAALDGDEFRVPLNSPVILVQSGNRAPETIMQEE

100   MRKYYTVSTFSGIPDRQKPLTCNKNKDKNENEDVASAENMNWMQALRFVA
                      ************************
150   AKGHQKAIIVYQDMLQTGKYDSALKSTVWSDYKNDKLTDAISLRYLVRFT
```

```
200   LVDVATGEWATWSPVNYEYKVLPPLPDKNEASTTDMTEQQIMQLKQKTYK

250   AMVKDLVNRYQ
```

sp|P25907|YDBD_ECOLI Uncharacterized protein YdbD OS=Escherichia coli (strain K12) OX=83333 GN=ydbD PE=4 SV=2

```
0     MLKARNCGWIRLLPLFMLSLPVQAELRCVANAVDIESFFSAATAEDKQQV

50    EQAINSSVNLVPFGLSASNWKVHRGDLVVEGNIESNQKLIVLGNLTVKGN

100   ISTFSLSNPWVILGNVTATNIVADSPLLITGSINASGLVFIDSYYDNPST

150   IKGSINARGIFINDIIAPVVASSTNSEFMVRASDKHDTENVKKALMIINP

200   DAYYWGLINDEDALKEIFKRSNIRMAGNVCNQMKKEALFRPKPSPELVQE

250   LQMLDEGKVAAFEGRDIATFDLAVMRTLPRLKGISANLRKQLINSNDEQT

300   IESMARYMPDNEILELTDQQLGYQPVVLGLLDREPLSVEIMTRMSRLPDG

350   VGPLNLALRENLPLDIVMTLAKRDWDMIIQELYKDAWLLPESIIDGYIRS

400   DDSSIRQVGAGGQLTYNQAMQLANDSSNNVVTSLAFKLAEMKHHGQLLRM

450   TPQESDKVAGYLYQKFENDDDLIRVLFLALPDNLQFNFVKRMEKKSPAYF

500   CCRDMQVIHSDAALQRLLTRFNDPEGWSNLAKNQYLSTSMKQKIWQRALS
            ********************
550   HRKNNPKADSDAYETSADMILSELISHGEVDDQMLLNATALIRSDDWDFL

600   ESALISWDNLPAVVLKELQQNTPRNDIWAKFFLRQENSSRAQVDEALRVY

650   YALDPDALAQLDVLAKQPDRIWWSTLAKSNLTFFKFGALNNRHTPPAVLA

700   AEIDPEWWIVAMNNPRFPVDVLKARLKRDPLLALELVNPELDLVRQLALN

750   GKTRAIREQAMRKLDELY
```

sp|P63389|YHES_ECOLI Uncharacterized ABC transporter ATP-binding protein YheS OS=Escherichia coli (strain K12) OX=83333 GN=yheS PE=1 SV=1

```
0     MIVFSSLQIRRGVRVLLDNATATINPGQKVGLVGKNGCGKSTLLALLKNE

50    ISADGGSYTFPGSWQLAWVNQETPALPQAALEYVIDGDREYRQLEAQLHD

100   ANERNDGHAIATIHGKLDAIDAWSIRSRAASLLHGLGFSNEQLERPVSDF

150   SGGWRMRLNLAQALICRSDLLLLDEPTNHLDLDAVIWLEKWLKSYQGTLI

200   LISHDRDFLDPIVDKIIHIEQQSMFEYTGNYSSFEVQRATRLAQQQAMYE

250   SQQERVAHLQSYIDRFRAKATKAKQAQSRIKMLERMELIAPAHVDNPFRF
```

```
300   SFRAPESLPNPLLKMEKVSAGYGDRIILDSIKLNLVPGSRIGLLGRNGAG

350   KSTLIKLLAGELAPVSGEIGLAKGIKLGYFAQHQLEYLRADESPIQHLAR

400   LAPQELEQKLRDYLGGFGFQGDKVTEETRRFSGGEKARLVLALIVWQRPN

450   LLLLDEPTNHLDLDMRQALTEALIEFEGALVVVSHDRHLLRSTTDDLYLV

500   HDRKVEPFDGDLEDYQQWLSDVQKQENQTDEAPKENANSAQARKDQKRRE
                                  **********************
550   AELRAQTQPLRKEIARLEKEMEKLNAQLAQAEEKLGDSELYDQSRKAELT
      *
600   ACLQQQASAKSGLEECEMAWLEAQEQLEQMLLEGQSN
```

sp|P0ADK8|YIBL_ECOLI Uncharacterized protein YibL OS=Escherichia coli (strain K12) OX=83333 GN=yibL PE=1 SV=1

```
                            ***************************
0     MKEVEKNEIKRLSDRLDAIRHQQADLSLVEAADKYAELEKEKATLEAEIA

50    RLREVHSQKLSKEAQKLMKMPFQRAITKKEQADMGKLKKSVRGLVVVHPM

100   TALGREMGLQEMTGFSKTAF
```

sp|P15038|HELD_ECOLI DNA helicase IV OS=Escherichia coli (strain K12) OX=83333 GN=helD PE=1 SV=2

```
0     MELKATTLGKRLAQHPYDRAVILNAGIKVSGDRHEYLIPFNQLLAIHCKR

50    GLVWGELEFVLPDEKVVRLHGTEWGETQRFYHHLDAHWRRWSGEMSEIAS

100   GVLRQQLDLIATRTGENKWLTREQTSGVQQQIRQALSALPLPVNRLEEFD

150   NCREAWRKCQAWLKDIESARLQHNQAYTEAMLTEYADFFRQVESSPLNPA

200   QARAVVNGEHSLLVLAGAGSGKTSVLVARAGWLLARGEASPEQILLLAFG

250   RKAAEEMDERIRERLHTEDITARTFHALALHIIQQGSKKVPIVSKLENDT

300   AARHELFIAEWRKQCSEKKAQAKGWRQWLTEEMQWSVPEGNFWDDEKLQR

350   RLASRLDRWVSLMRMHGGAQAEMIASAPEEIRDLFSKRIKLMAPLLKAWK

400   GALKAENAVDFSGLIHQAIVILEKGRFISPWKHILVDEFQDISPQRAALL

450   AALRKQNSQTTLFAVGDDWQAIYRFSGAQMSLTTAFHENFGEGERCDLDT
                                               *********
500   TYRFNSRIGEVANRFIQQNPGQLKKPLNSLTNGDKKAVTLLDESQLDALL
      ***********
550   DKLSGYAKPEERILILARYHHMRPASLEKAATRWPKLQIDFMTIHASKGQ

600   QADYVIIVGLQEGSDGFPAAARESIMEEALLPPVEDFPDAEERRLMYVAL

650   TRARHRVWALFNKENPSPFVEILKNLDVPVARKP
```

sp|P76116|YNCE_ECOLI Uncharacterized protein YncE OS=Escherichia coli (strain K12) OX=83333 GN=yncE PE=1 SV=1

```
0     MHLRHLFSSRLRGSLLLGSLLVVSSFSTQAAEEMLRKAVGKGAYEMAYSQ

50    QENALWLATSQSRKLDKGGVVYRLDPVTLEVTQAIHNDLKPFGATINNTT

100   QTLWFGNTVNSAVTAIDAKTGEVKGRLVLDDRKRTEEVRPLQPRELVADD

150   ATNTVYISGIGKESVIWVVDGGNIKLKTAIQNTGKMSTGLALDSEGKRLY
                *********************
200   TTNADGELITIDTADNKILSRKKLLDDGKEHFFINISLDTARQRAFITDS

250   KAAEVLVVDTRNGNILAKVAAPESLAVLFNPARNEAYVTHRQAGKVSVID

300   AKSYKVVKTFDTPTHPNSLALSADGKTLYVSVKQKSTKQQEATQPDDVIR

350   IAL
```

sp|P0AEK7|FDNI_ECOLI Formate dehydrogenase, nitrate-inducible, cytochrome b556(Fdn) subunit OS=Escherichia coli (strain K12) OX=83333 GN=fdnI PE=1 SV=1

```
0     MSKSKMIVRTKFIDRACHWTVVICFFLVALSGISFFFPTLQWLTQTFGTP

50    QMGRILHPFFGIAIFVALMFMFVRFVHHNIPDKKDIPWLLNIVEVLKGNE

100   HKVADVGKYNAGQKMMFWSIMSMIFVLLVTGVIIWRPYFAQYFPMQVVRY
                                                 *******
150   SLLIHAAAGIILIHAILIHMYMAFWVKGSIKGMIEGKVSRRWAKKHHPRW
      *****************
200   YREIEKAEAKKESEEGI
```

sp|P0A8V2|RPOB_ECOLI DNA-directed RNA polymerase subunit beta OS=Escherichia coli (strain K12) OX=83333 GN=rpoB PE=1 SV=1

```
0     MVYSYTEKKRIRKDFGKRPQVLDVPYLLSIQLDSFQKFIEQDPEGQYGLE

50    AAFRSVFPIQSYSGNSELQYVSYRLGEPVFDVQECQIRGVTYSAPLRVKL

100   RLVIYEREAPEGTVKDIKEQEVYMGEIPLMTDNGTFVINGTERVIVSQLH

150   RSPGVFFDSDKGKTHSSGKVLYNARIIPYRGSWLDFEFDPKDNLFVRIDR

200   RRKLPATIILRALNYTTEQILDLFFEKVIFEIRDNKLQMELVPERLRGET

250   ASFDIEANGKVYVEKGRRITARHIRQLEKDDVKLIEVPVEYIAGKVVAKD

300   YIDESTGELICAANMELSLDLLAKLSQSGHKRIETLFTNDLDHGPYISET

350   LRVDPTNDRLSALVEIYRMMRPGEPPTREAAESLFENLFFSEDRYDLSAV
             ***************************
400   GRMKFNRSLLREEIEGSGILSKDDIIDVMKKLIDIRNGKGEVDDIDHLGN
```

450   RRIRSVGEMAENQFRVGLVRVERAVKERLSLGDLDTLMPQDMINAKPISA

500   AVKEFFGSSQLSQFMDQNNPLSEITHKRRISALGPGGLTRERAGFEVRDV

550   HPTHYGRVCPIETPEGPNIGLINSLSVYAQTNEYGFLETPYRKVTDGVVT
                                   ****
600   DEIHYLSAIEEGNYVIAQANSNLDEEGHFVEDLVTCRSKGESSLFSRDQV
        ****
650   DYMDVSTQQVVSVGASLIPFLEHDDANRALMGANMQRQAVPTLRADKPLV

700   GTGMERAVAVDSGVTAVAKRGGVVQYVDASRIVIKVNEDEMYPGEAGIDI

750   YNLTKYTRSNQNTCINQMPCVSLGEPVERGDVLADGPSTDLGELALGQNM

800   RVAFMPWNGYNFEDSILVSERVVQEDRFTTIHIQELACVSRDTKLGPEEI
                                                      ****
850   TADIPNVGEAALSKLDESGIVYIGAEVTGGDILVGKVTPKGETQLTPEEK
        ******************   ************
900   LLRAIFGEKASDVKDSSLRVPNGVSGTVIDVQVFTRDGVEKDKRALEIEE
        ****
950   MQLKQAKKDLSEELQILEAGLFSRIRAVLVAGGVEAEKLDKLPRDRWLEL
                  ****
1000   GLTDEEKQNQLEQLAEQYDELKHEFEKKLEAKRRKITQGDDLAPGVLKIV

1050   KVYLAVKRRIQPGDKMAGRHGNKGVISKINPIEDMPYDENGTPVDIVLNP

1100   LGVPSRMNIGQILETHLGMAAKGIGDKINAMLKQQQEVAKLREFIQRAYD

1150   LGADVRQKVDLSTFSDEEVMRLAENLRKGMPIATPVFDGAKEAEIKELLK

1200   LGDLPTSGQIRLYDGRTGEQFERPVTVGYMYMLKLNHLVDDKMHARSTGS

1250   YSLVTQQPLGGKAQFGGQRFGEMEVWALEAYGAAYTLQEMLTVKSDDVNG

1300   RTKMYKNIVDGNHQMEPGMPESFNVLLKEIRSLGINIELEDE

sp|P15723|DGTP_ECOLI Deoxyguanosinetriphosphate triphosphohydrolase OS=Escherichia coli (strain K12) OX=83333 GN=dgt PE=1 SV=4

0   MAQIDFRKKINWHRRYRSPQGVKTEHEILRIFESDRGRIINSPAIRRLQQ

50   KTQVFPLERNAAVRTRLTHSMEVQQVGRYIAKEILSRLKELKLLEAYGLD

100   ELTGPFESIVEMSCLMHDIGNPPFGHFGEAAINDWFRQRLHPEDAESQPL

150   TDDRCSVAALRLRDGEEPLNELRRKIRQDLCHFEGNAQGIRLVHTLMRMN

200   LTWAQVGGILKYTRPAWWRGETPETHHYLMKKPGYYLSEEAYIARLRKEL

250   NLALYSRFPLTWIMEAADDISYCVADLEDAVEKRIFTVEQLYHHLHEAWG
                  ****
300   QHEKGSLFSLVVENAWEKSRSNSLSRSTEDQFFMYLRVNTLNKLVPYAAQ

350   RFIDNLPAIFAGTFNHALLEDASECSDLLKLYKNVAVKHVFSHPDVERLE

400   LQGYRVISGLLEIYRPLLSLSLSDFTELVEKERVKRFPIESRLFHKLSTR

450   HRLAYVEAVSKLPSDSPEFPLWEYYYRCRLLQDYISGMTDLYAWDEYRRL

500   MAVEQ

sp|P23827|ECOT_ECOLI Ecotin OS=Escherichia coli (strain K12) OX=83333 GN=eco PE=1 SV=1

0   MKTILPAVLFAAFATTSAWAAESVQPLEKIAPYPQAEKGMKRQVIQLTPQ
                                                  ******
50   EDESTLKVELLIGQTLEVDCNLHRLGGKLENKTLEGWGYDYYVFDKVSSP
      ****
100   VSTMMACPDGKKEKKFVTAYLGDAGMLRYNSKLPIVVYTPDNVDVKYRVW

150   KAEEKIDNAVVR

sp|P76016|DHAR_ECOLI PTS-dependent dihydroxyacetone kinase operon regulatory protein OS=Escherichia coli (strain K12) OX=83333 GN=dhaR PE=1 SV=2

0   MSGAFNNDGRGISPLIATSWERCNKLMKRETWNVPHQAQGVTFASIYRRK

50   KAMLTLGQAALEDAWEYMAPRECALFILDETACILSRNGDPQTLQQLSAL

100   GFNDGTYCAEGIIGTCALSLAAISGQAVKTMADQHFKQVLWNWAFCATPL

150   FDSKGRLTGTIALACPVEQTTAADLPLTLAIAREVGNLLLTDSLLAETNR

200   HLNQLNALLESMDDGVISWDEQGNLQFINAQAARVLRLDATASQGRAITE

250   LLTLPAVLQQAIKQAHPLKHVEATFESQHQFIDAVITLKPIIETQGTSFI

300   LLLHPVEQMRQLMTSQLGKVSHTFAHMPQDDPQTRRLIHFGRQAARSSFP

350   VLLCGEEGVGKALLSQAIHNESERAAGPYIAVNCELYGDAALAEEFIGGD

400   RTDNENGRLSRLELAHGGTLFLEKIEYLAVELQSALLQVIKQGVITRLDA

450   RRLIPIDVKVIATTTADLAMLVEQNRFSRQLYYALHAFEITIPPLRMRRG

500   SIPALVNNKLRSLEKRFSTRLKIDDDALARLVSCAWPGNDFELYSVIENL
                              ****
550   ALSSDNGRIRVSDLPEHLFTEQATDDVSATRLSTSLSFAEVEKEAIINAA

600   QVTGGRIQEMSALLGIGRTTLWRKMKQHGIDAGQFKRRV

sp|P77234|YBEQ_ECOLI Sel1-repeat-containing protein YbeQ OS=Escherichia coli (strain K12) OX=83333 GN=ybeQ PE=4 SV=2
          ****
0   MIFTSSCCDNLSIDEIIERAEKGDCEAQYIVGFYYNRDSAIDSPDDEKAF

50   YWLKLAAEQGHCEAQYSLGQKYTEDKSRHKDNEQAIFWLKKAALQGHTFA

100   SNALGWTLDRGEAPNYKEAVVWYQIAAESGMSYAQNNLGWMYRNGNGVAK

150   DYALAFFWYKQAALQGHSDAQNNLADLYEDGKGVAQNKTLAAFWYLKSAQ

200   QGNRHAQFQIAWDYNAGEGVDQDYKQAMYWYLKAAAQGSVGAYVNIGYMY

250   KHGQGVEKDYQAAFEWFTKAAECNDATAWYNLAIMYHYGEGRPVDLRQAL

300   DLYRKVQSSGTRDVSQEIRETEDLL

sp|P31667|RPNA_ECOLI Recombination-promoting nuclease RpnA OS=Escherichia coli (strain K12) OX=83333 GN=rpnA PE=1 SV=1

```
                                        **************
0     MSKKQSSTPHDALFKLFLRQPDTARDFLAFHLPAPIHALCDMKTLKLESS
      ***********************
50    SFIDDDLRESYSDVLWSVKTEQGPGYIYCLIEHQSTSNKLIAFRMMRYAI
```

100   AAMQNHLDAGYKTLPMVVPLLFYHGIESPYPYSLCWLDCFADPKLARQLY

150   ASAFPLIDVTVMPDDEIMQHRRMALLELIQKHIRQRDLMGLVEQMACLLS

200   SGYANDRQIKGLFNYILQTGDAVRFNDFIDGVAERSPKHKESLMTIAERL

250   RQEGEQSKALHIAKIMLESGVPLADIMRFTGLSEEELAAASQ

sp|P77802|ECPC_ECOLI Probable outer membrane usher protein EcpC OS=Escherichia coli (strain K12) OX=83333 GN=ecpC PE=1 SV=1

0   MPLRRFSPGLKAQFAFGMVFLFVQPDASAADISAQQIGGVIIPQAFSQAL

50   QDGMSVPLYIHLAGSQGRQDDQRIGSAFIWLDDGQLRIRKIQLEESEDNA

100   SVSEQTRQQLMALANAPFNEALTIPLTDNAQLDLSLRQLLLQLVVKREAL

150   GTVLRSRSEDIGQSSVNTLSSNLSYNLGVYNNQLRNGGSNTSSYLSLNNV

200   TALREHHVVLDGSLYGIGSGQQDSELYKAMYERDFAGHRFAGGMLDTWNL

250   QSLGPMTAISAGKIYGLSWGNQASSTIFDSSQSATPVIAFLPAAGEVHLT

300   RDGRLLSVQNFTMGNHEVDTRGLPYGIYDVEVEVIVNGRVISKRTQRVNK

350   LFSRGRGVGAPLAWQVWGGSFHMDRWSENGKKTRPAKESWLAGASTSGSL

400   STLSWAATGYGYDNQAVGETRLTLPLGGAINVNLQNMLASDSSWSSIGSI

450   SATLPGGFSSLWVNQEKTRIGNQLRRSDADNRAIGGTLNLNSLWSKLGTF

500   SISYNDDRRYNSHYYTADYYQNVYSGTFGSLGLRAGIQRYNNGDSNANTG

550   KYIALDLSLPLGNWFSAGMTHQNGYTMANLSARKQFDEGTIRTVGANLSR

600   AISGDTGDDKTLSGGAYAQFDARYASGTLNVNSAADGYVNTNLTANGSVG

650   WQGKNIAASGRTDGNAGVIFNTGLEDDGQISAKINGRIFPLNGKRNYLPL
            ****
700   SPYGRYEVELQNSKNSLDSYDIVSGRKSRLTLYPGNVAVIEPEVKQMVTV

750   SGRIRAEDGTLLANARINNHIGRTRTDENGEFVMDVDKKYPTIDFRYSGN

800   KTCEVALELNQARGAVWVGDVVCSGLSSWAAVTQTGEENES

sp|P17994|YFAA_ECOLI Uncharacterized protein YfaA OS=Escherichia coli (strain K12) OX=83333 GN=yfaA PE=4 SV=3

0   MSGEKKAKGWRFYGLVGFGAIALLSAGVWALQYAGSGPEKTLSPLVVHNN
    ****
50   LQIDLNEPDLFLDSDSLSQLPKDLLTIPFLHDVLSEDFVFYYQNHADRLG

100   IEGSIRRIVYEHDLTLKDKLFSSLLDQPAQAALWHDKQGHLSHYMVLIQR
             ****
150   SGLSKLLEPLLFAATSDSQLSKTEISSIKINSETVPVYQLRYNGNNALMF

200   ATYQDKMLVFSSTDMLFKDDQQDTEATAIAGDLLSGKKRWQASFGLEERT

250   AEKTPVRQRIVVSARWLGFGYQRLMPSFAGVRFEMGNDGWHSFVALNDES

300   ASVDASFDFTPVWNSMPAGASFCVAVPYSHGIAEEMLSHISQENDKLNGA

350   LDGAAGLCWYEDSKLQTPLFVGQFDGTAEQAQLPGKLFTQNIGAHESKAP

400   EGVLPVSQTQQGEAQIWRREVSSRYGQYPKAQAAQPDQLMSDYFFRVSLA

450   MQNKTLLFSLDDTLVNNALQTLNKTRPAMVDVIPTDGIVPLYINPQGIAK

500   LLRNETLTSLPKNLEPVFYNAAQTLLMPKLDALSQQPRYVMKLAQMEPGA

550   AWQWLPITWQPL

sp|P37679|SGBU_ECOLI Putative L-ribulose-5-phosphate 3-epimerase SgbU OS=Escherichia coli (strain K12) OX=83333 GN=sgbU PE=3 SV=2
                          ****
0   MRNHQLGIYEKALAKDLSWPERLVLAKSCGFDFVEMSVDETDERLSRLDW

50   SAAQRTSLVAAMIETGVGIPSMCLSAHRRFPFGSRDEAVRERAREIMSKA

100   IRLARDLGIRTIQLAGYDVYYEDHDEGTRQRFAEGLAWAVEQAAASQVML

150   AVEIMDTAFMNSISKWKKWDEMLASPWFTVYPDVGNLSAWGNDVPAELKL

200   GIDRIAAIHLKDTQPVTGQSPGQFRDVPFGEGCVDFVGIFKTLHKLNYRG

250   SFLIEMWTEKAKEPVLEIIQARRWIEARMQEAGFIC

sp|P39838|RCSD_ECOLI Phosphotransferase RcsD OS=Escherichia coli (strain K12) OX=83333 GN=rcsD PE=1 SV=3

```
0     MRQKETTATTRFSLLPGSITRFFLLLIIVLLVTMGVMVQSAVNAWLKDKS

50    YQIVDITHAIQKRVDNWRYVTWQIYDNIAATTSPSSGEGLQETRLKQDVY

100   YLEKPRRKTEALIFGSHDNSTLEMTQRMSTYLDTLWGAENVPWSMYYLNG
                 **********************
150   QDNSLVLISTLPLKDLTSGFKESTVSDIVDSRRAEMLQQANALDERESFS

200   NMRRLAWQNGHYFTLRTTFNQPGHLATVVAFDLPINDLIPPGMPLDSFRL
                ***********************
250   EPDATATGNNDNEKEGTDSVSIHFNSTKIEISSALNSTDMRLVWQVPYGT

300   LLLDTLQNILLPLLLNIGLLALALFGYTTFRHFSSRSTENVPSTAVNNEL

350   RILRAINEEIVSLLPLGLLVHDQESNRTVISNKIADHLLPHLNLQNITTM

400   AEQHQGIIQATINNELYEIRMFRSQVAPRTQIFIIRDQDREVLVNKKLKQ

450   AQRLYEKNQQGRMIFMKNIGDALKEPAQSLAESAAKLNAPESKQLANQAD
                     *********************
500   VLVRLVDEIQLANMLADDSWKSETVLFSVQDLIDEVVPSVLPAIKRKGLQ
                                      ******************
550   LLINNHLKAHDMRRGDRDALRRILLLLMQYAVTSTQLGKITLEVDQDESS
      ***
600   EDRLTFRILDTGEGVSIHEMDNLHFPFINQTQNDRYGKADPLAFWLSDQL
                             *********************
650   ARKLGGHLNIKTRDGLGTRYSVHIKMLAADPEVEEEEERLLDDVCVMVDV

700   TSAEIRNIVTRQLENWGATCITPDERLISQDYDIFLTDNPSNLTASGLLL

750   SDDESGVREIGPGQLCVNFNMSNAMQEAVLQLIEVQLAQEEVTESPLGGD

800   ENAQLHASGYYALFVDTVPDDVKRLYTEAATSDFAALAQTAHRLKGVFAM
                       **********************
850   LNLVPGKQLCETLEHLIREKDVPGIEKYISDIDSYVKSLL
```

sp|P35340|AHPF_ECOLI Alkyl hydroperoxide reductase subunit F
OS=Escherichia coli (strain K12) OX=83333 GN=ahpF PE=1 SV=2

```
                         *******************************
0     MLDTNMKTQLKAYLEKLTKPVELIATLDDSAKSAEIKELLAEIAELSDKV
      **************
50    TFKEDNSLPVRKPSFLITNPGSNQGPRFAGSPLGHEFTSLVLALLWTGGH

100   PSKEAQSLLEQIRHIDGDFEFETYYSLSCHNCPDVVQALNLMSVLNPRIK

150   HTAIDGGTFQNEITDRNVMGVPAVFVNGKEFGQGRMTLTEIVAKIDTGAE

200   KRAAEELNKRDAYDVLIVGSGPAGAAAAIYSARKGIRTGLMGERFGGQIL
                            *********************
250   DTVDIENYISVPKTEGQKLAGALKVHVDEYDVDVIDSQSASKLIPAAVEG

300   GLHQIETASGAVLKARSIIVATGAKWRNMNVPGEDQYRTKGVTYCPHCDG

350   PLFKGKRVAVIGGGNSGVEAAIDLAGIVEHVTLLEFAPEMKADQVLQDKL
```

400   RSLKNVDIILNAQTTEVKGDGSKVVGLEYRDRVSGDIHNIELAGIFVQIG

450   LLPNTNWLEGAVERNRMGEIIIDAKCETNVKGVFAAGDCTTVPYKQIIIA

500   TGEGAKASLSAFDYLIRTKTA

sp|P76160|YDFR_ECOLI Uncharacterized protein YdfR OS=Escherichia coli (strain K12) OX=83333 GN=ydfR PE=1 SV=1

```
0     MTQDYELVVKGVRNFENKVTVTVALQDKERFDGEIFDLDVAMDRVEGAAL
                         ********************
50    EFYEAAARRSVRQVFLEVAEKLSEKVESYLQHQYSFKIENPANKHERPHH

100   KYL
```

sp|P77607|YAGL_ECOLI Uncharacterized protein YagL OS=Escherichia coli (strain K12) OX=83333 GN=yagL PE=4 SV=1

```
0     MRGKILLYQLKYRWQSLSIFGCFLCKMTLFRYQKIIYDTGVHQMRSFFYT

50    ICSSEQQESITDHHSLAEICQKFNILPEHVVIEQVDIKEVVSEQRLLRQL

100   IHHEMNRQDTLVIPDLSCLGRTVEDLQNILFFCLQKEMFIYSYHPASRIE
                            *********************
150   PSAESCLSFLIARQDTIDIHNLKSTKSRYRHVKKKLGRKEGSKYRRDITI

200   LKKGGFTQAEIAKKLSISLSTVKRHWNNGIIG
```

sp|P0A8H8|YACG_ECOLI DNA gyrase inhibitor YacG OS=Escherichia coli (strain K12) OX=83333 GN=yacG PE=1 SV=1

```
                                         ***************
0     MSETITVNCPTCGKTVVWGEISPFRPFCSKRCQLIDLGEWAAEEKRIPSS
      ***************
50    GDLSESDDWSEEPKQ
```

sp|P0AFZ3|SSPB_ECOLI Stringent starvation protein B OS=Escherichia coli (strain K12) OX=83333 GN=sspB PE=1 SV=1

```
0     MDLSQLTPRRPYLLRAFYEWLLDNQLTPHLVVDVTLPGVQVPMEYARDGQ

50    IVLNIAPRAVGNLELANDEVRFNARFGGIPRQVSVPLAAVLAIYARENGA
                       *********************************
100   GTMFEPEAAYDEDTSIMNDEEASADNETVMSVIDGDKPDHDDDTHPDDEP
      ***
150   PQPPRGGRPALRVVK
```

sp|P09378|RHAR_ECOLI HTH-type transcriptional activator RhaR OS=Escherichia coli (strain K12) OX=83333 GN=rhaR PE=1 SV=2

```
0     MAHQLKLLKDDFFASDQQAVAVADRYPQDVFAEHTHDFCELVIVWRGNGL

50    HVLNDRPYRITRGDLFYIHADDKHSYASVNDLVLQNIIYCPERLKLNLDW
```

```
100   QGAIPGFNASAGQPHWRLGSMGMAQARQVIGQLEHESSQHVPFANEMAEL
                                            **************
150   LFGQLVMLLNRHRYTSDSLPPTSSETLLDKLITRLAASLKSPFALDKFCD
      *******
200   EASCSERVLRQQFRQQTGMTINQYLRQVRVCHAQYLLQHSRLLISDISTE

250   CGFEDSNYFSVVFTRETGMTPSQWRHLNSQKD
```

sp|P32680|YJAG_ECOLI Uncharacterized protein YjaG OS=Escherichia coli (strain K12) OX=83333 GN=yjaG PE=4 SV=1

```
0     MLQNPIHLRLERLESWQHVTFMACLCERMYPNYAMFCQQTGFGDGQIYRR
                 ***************************
50    ILDLIWETLTVKDAKVNFDSQLEKFEEAIPSADDFDLYGVYPAIDACVAL
                                           ***************
100   SELVHSRLSGETLEHAVEVSKTSITTVAMLEMTQAGREMSDEELKENPAV
      *********
150   EQEWDIQWEIFRLLAECEERDIELIKGLRADLREAGESNIGIIFQQ
```

sp|P06846|EBGR_ECOLI HTH-type transcriptional regulator EbgR OS=Escherichia coli (strain K12) OX=83333 GN=ebgR PE=4 SV=2

```
                                    ********************
0     MATLKDIAIEAGVSLATVSRVLNDDPTLNVKEETKHRILEIAEKLEYKTS
      *
50    SARKLQTGAVNQHHILAIYSYQQELEINDPYYLAIRHGIETQCEKLGIEL

100   TNCYEHSGLPDIKNVTGILIVGKPTPALRAAASALTDNICFIDFHEPGSG

150   YDAVDIDLARISKEIIDFYINQGVNRIGFIGGEDEPGKADIREVAFAEYG

200   RLKQVVREEDIWRGGFSSSSGYELAKQMLAREDYPKALFVASDSIAIGVL

250   RAIHERGLNIPQDISLISVNDIPTARFTFPPLSTVRIHSEMMGSQGVNLV

300   YEKARDGRALPLLVFVPSKLKLRGTTR
```

sp|P28635|METQ_ECOLI D-methionine-binding lipoprotein MetQ OS=Escherichia coli (strain K12) OX=83333 GN=metQ PE=1 SV=2

```
0     MAFKFKTFAAVGALIGSLALVGCGQDEKDPNHIKVGVIVGAEQQVAEVAQ

50    KVAKDKYGLDVELVTFNDYVLPNEALSKGDIDANAFQHKPYLDQQLKDRG

100   YKLVAVGNTFVYPIAGYSKKIKSLDELQDGSQVAVPNDPTNLGRSLLLLQ

150   KVGLIKLKDGVGLLPTVLDVVENPKNLKIVELEAPQLPRSLDDAQIALAV
                                        ****************
200   INTTYASQIGLTPAKDGIFVEDKESPYVNLIVTREDNKDAENVKKFVQAY
      ****
250   QSDEVYEAANKVFNGGAVKGW
```

sp|P41442|GSPG_ECOLI Putative type II secretion system protein G OS=Escherichia coli (strain K12) OX=83333 GN=gspG PE=2 SV=1

0   MRATDKQRGFTLLEIMVVIVIIGVLASLVVPNLMGNKEKADKQKAVSDIV

50   ALENALDMYKLDNHHYPTTNQGLESLVEAPTLPPLAANYNKEGYIKRLPA
                                ****
100   DPWGNDYVLVNPGEHGAYDLLSAGPDGEMGTEDDITNWGLSKKKK

sp|P33594|NIKE_ECOLI Nickel import ATP-binding protein NikE
OS=Escherichia coli (strain K12) OX=83333 GN=nikE PE=3 SV=2

0   MTLLNISGLSHHYAHGGFNGKHQHQAVLNNVSLTLKSGETVALLGRSGCG

50   KSTLARLLVGLESPAQGNISWRGEPLAKLNRAQRKAFRRDIQMVFQDSIS
                              ****
100   AVNPRKTVREILREPMRHLLSLKKSEQLARASEMLKAVDLDDSVLDKRPP

150   QLSGGQLQRVCLARALAVEPKLLILDEAVSNLDLVLQAGVIRLLKKLQQQ

200   FGTACLFITHDLRLVERFCQRVMVMDNGQIVETQVVGEKLTFSSDAGRVL

250   QNAVLPAFPVRRRTTEKV

sp|P67553|YNFC_ECOLI UPF0257 lipoprotein YnfC OS=Escherichia coli (strain
K12) OX=83333 GN=ynfC PE=3 SV=1

0   MKYKLLPCLLAIFLTGCDRTEVTLSFTPEMASFSNEFDFDPLRGPVKDFT

50   QTLMDEQGEVTKRVSGTLSEEGCFDSLELLDLENNTVVALVLDANYYRDA

100   ETLEKRVRLQGKCQLAELPSAGVSWETDDNGFVIKASSKQMQMEYRYDDQ
                ****
150   GYPLGKTTKSNDKTLSVSATPSTDPIKKLDYTAVTLLNNQRVGNVKQSCE

200   YDSHANPVDCQLIIVDEGVKPAVERVYTIKNTIDYY

sp|P0AAN9|IRAP_ECOLI Anti-adapter protein IraP OS=Escherichia coli
(strain K12) OX=83333 GN=iraP PE=1 SV=1
       ****
0   MKNLIAELLFKLAQKEEESKELCAQVEALEIIVTAMLRNMAQNDQQRLID
                 ****
50   QVEGALYEVKPDASIPDDDTELLRDYVKKLLKHPRQ

sp|P00962|SYQ_ECOLI Glutamine--tRNA ligase OS=Escherichia coli (strain
K12) OX=83333 GN=glnS PE=1 SV=3

0   MSEAEARPTNFIRQIIDEDLASGKHTTVHTRFPPEPNGYLHIGHAKSICL
                     ****
50   NFGIAQDYKGQCNLRFDDTNPVKEDIEYVESIKNDVEWLGFHWSGNVRYS

100   SDYFDQLHAYAIELINKGLAYVDELTPEQIREYRGTLTQPGKNSPYRDRS

150   VEENLALFEKMRAGGFEEGKACLRAKIDMASPFIVMRDPVLYRIKFAEHH

200   QTGNKWCIYPMYDFTHCISDALEGITHSLCTLEFQDNRRLYDWVLDNITI

```
250   PVHPRQYEFSRLNLEYTVMSKRKLNLLVTDKHVEGWDDPRMPTISGLRRR

300   GYTAASIREFCKRIGVTKQDNTIEMASLESCIREDLNENAPRAMAVIDPV

350   KLVIENYQGEGEMVTMPNHPNKPEMGSRQVPFSGEIWIDRADFREEANKQ

400   YKRLVLGKEVRLRNAYVIKAERVEKDAEGNITTIFCTYDADTLSKDPADG

450   RKVKGVIHWVSAAHALPVEIRLYDRLFSVPNPGAADDFLSVINPESLVIK

500   QGFAEPSLKDAVAGKAFQFEREGYFCLDSRHSTAEKPVFNRTVGLRDTWA

550   KVGE

sp|P0A6D7|AROK_ECOLI Shikimate kinase 1 OS=Escherichia coli (strain K12)
OX=83333 GN=aroK PE=1 SV=2

0     MAEKRNIFLVGPMGAGKSTIGRQLAQQLNMEFYDSDQEIEKRTGADVGWV
         ********************
50    FDLEGEEGFRDREEKVINELTEKQGIVLATGGGSVKSRETRNRLSARGVV

100   VYLETTIEKQLARTQRDKKRPLLHVETPPREVLEALANERNPLYEEIADV

150   TIRTDDQSAKVVANQIIHMLESN

sp|P0AEJ6|EUTB_ECOLI Ethanolamine ammonia-lyase heavy chain
OS=Escherichia coli (strain K12) OX=83333 GN=eutB PE=1 SV=1

0     MKLKTTLFGNVYQFKDVKEVLAKANELRSGDVLAGVAAASSQERVAAKQV
                                              ************
50    LSEMTVADIRNNPVIAYEDDCVTRLIQDDVNETAYNQIKNWSISELREYV
      ********
100   LSDETSVDDIAFTRKGLTSEVVAAVAKICSNADLIYGAKKMPVIKKANTT

150   IGIPGTFSARLQPNDTRDDVQSIAAQIYEGLSFGVGDAVIGVNPVTDDVE

200   NLSRVLDTIYGVIDKFNIPTQGCVLAHVTTQIEAIRRGAPGGLIFQSICG

250   SEKGLKEFGVELAMLDEARAVGAEFNRIAGENCLYFETGQGSALSAGANF

300   GADQVTMEARNYGLARHYDPFIVNTVVGFIGPEYLYNDRQIIRAGLEDHF

350   MGKLSGISMGCDCCYTNHADADQNLNENLMILLATAGCNYIMGMPLGDDI

400   MLNYQTTAFHDTATVRQLLNLRPSPEFERWLESMGIMANGRLTKRAGDPS

450   LFF

sp|P69425|TATB_ECOLI Sec-independent protein translocase protein TatB
OS=Escherichia coli (strain K12) OX=83333 GN=tatB PE=1 SV=1
                                                      **
0     MFDIGFSELLLVFIIGLVVLGPQRLPVAVKTVAGWIRALRSLATTVQNEL
      **********************               *************
50    TQELKLQEFQDSLKKVEKASLTNLTPELKASMDELRQAAESMKRSYVAND
```

```
     *********
100  PEKASDEAHTIHNPVVKDNEAAHEGVTPAAAQTQASSPEQKPETTPEPVV
     ********************
150  KPAADAEPKTAAPSPSSSDKP
```

sp|P60785|LEPA_ECOLI Elongation factor 4 OS=Escherichia coli (strain K12) OX=83333 GN=lepA PE=1 SV=1

```
0    MKNIRNFSIIAHIDHGKSTLSDRIIQICGGLSDREMEAQVLDSMDLERER

50   GITIKAQSVTLDYKASDGETYQLNFIDTPGHVDFSYEVSRSLAACEGALL

100  VVDAGQGVEAQTLANCYTAMEMDLEVVPVLNKIDLPAADPERVAEEIEDI

150  VGIDATDAVRCSAKTGVGVQDVLERLVRDIPPPEGDPEGPLQALIIDSWF

200  DNYLGVVSLIRIKNGTLRKGDKVKVMSTGQTYNADRLGIFTPKQVDRTEL

250  KCGEVGWLVCAIKDIHGAPVGDTLTLARNPAEKALPGFKKVKPQVYAGLF

300  PVSSDDYEAFRDALGKLSLNDASLFYEPESSSALGFGFRCGFLGLLHMEI

350  IQERLEREYDLDLITTAPTVVYEVETTSREVIYVDSPSKLPAVNNIYELR

400  EPIAECHMLLPQAYLGNVITLCVEKRGVQTNMVYHGNQVALTYEIPMAEV

450  VLDFFDRLKSTSRGYASLDYNFKRFQASDMVRVDVLINGERVDALALITH

500  RDNSQNRGRELVEKMKDLIPRQQFDIAIQAAIGTHIIARSTVKQLRKNVL
        *************************
550  AKCYGGDISRKKKLLQKQKEGKKRMKQIGNVELPQEAFLAILHVGKDNK
```

sp|P76621|GLAH_ECOLI Glutarate 2-hydroxylase OS=Escherichia coli (strain K12) OX=83333 GN=glaH PE=1 SV=2

```
0    MNALTAVQNNAVDSGQDYSGFTLTPSAQSPRLLELTFTEQTTKQFLEQVA

50   EWPVQALEYKSFLRFRVAKILDDLCANQLQPLLLKTLLNRAEGALLINAV

100  GVDDVKQADEMVKLATAVAHLIGRSNFDAMSGQYYARFVVKNVDNSDSYL

150  RQPHRVMELHNDGTYVEEITDYVLMMKIDEQNMQGGNSLLLHLDDWEHLD
                                                      *
200  NYFRHPLARRPMRFAAPPSKNVSKDVFHPVFDVDQQGRPVMRYIDQFVQP
     *********************
250  KDFEEGVWLSELSDAIETSKGILSVPVPVGKFLLINNLFWLHGRDRFTPH

300  PDLRRELMRQRGYFAYASNHYQTHQ
```

sp|P76134|YDEM_ECOLI Anaerobic sulfatase-maturating enzyme homolog YdeM OS=Escherichia coli (strain K12) OX=83333 GN=ydeM PE=3 SV=2

```
                         ************************
0    MHVTAKPSSFQCNLKCDYCFYLEKESQFTHEKWMDDSTLKEFIKQYIAAS
```

```
50    GNQVYFTWQGGEPTLAGLDFFRKVIHYQQRYAGQKRIFNALQTNGILLNN

100   EWCAFLKEHEFLVGISIDGPQELHDRYRRSNSGNGTFAKVIAAIERLKSY

150   QVEFNTLTVINNVNVHYPLEVYHFLKSIGSKHMQFIELLETGTPNIDFSG

200   HSENTFRIIDFSVPPTAYGKFMSTIFMQWVKNDVGEIFIRQFESFVSRFL

250   GNGHTSCIFQESCKDNLVVESNGDIYECDHFVYPQYKIGNINKSELKTMN

300   SVQLTAQKKRIPAKCQQCAYKPICNGGCPKHRITKVNNETVSYFCEGYKI

350   LFSTMVPYMNAMVELAKNRVPLYHIMDVAKQMENN
```

sp|P76505|YFDF_ECOLI Uncharacterized protein YfdF OS=Escherichia coli (strain K12) OX=83333 GN=yfdF PE=4 SV=1

```
0     MLPSISINNTSAAYPESINENNNDEVNGLVQEFKNLFNGKEGISTCIKHL

50    LELIKNAIRVNDDPYRFNINNSSVTYIDIDSNDTDHITIGIDNQEPIELP
                                                    ****
100   ANYKDKELVRTIINDNIVEKTHDINNKEMIFSALKEIYDGDPGFIFDKIS
      **********************************
150   HKLRHTVTEFDESGKSEPTDLFTWYGKDKKGDSLAIVIKNKNGNDYLSLG

200   YYDQDDYHIQRGIRINGDSLTQYCSENARSASAWFESSKAIMAESFATGS

250   DHQVVNELNGERLREPNDVFKRYGRAIRYDFQVDDAKYKCDHLKEIVSTL

300   VGNKINVGHSQKIYKHFKDLEGKIEERLQNRQAEYQNEINQPSAPGVNFD

350   DI
```

sp|P0AFF6|NUSA_ECOLI Transcription termination/antitermination protein NusA OS=Escherichia coli (strain K12) OX=83333 GN=nusA PE=1 SV=1

```
                                         ***************
0     MNKEILAVVEAVSNEKALPREKIFEALESALATATKKKYEQEIDVRVQID
      *****
50    RKSGDFDTFRRWLVVDEVTQPTKEITLEAARYEDESLNLGDYVEDQIESV

100   TFDRITTQTAKQVIVQKVREAERAMVVDQFREHEGEIITGVVKKVNRDNI

150   SLDLGNNAEAVILREDMLPRENFRPGDRVRGVLYSVRPEARGAQLFVTRS

200   KPEMLIELFRIEVPEIGEEVIEIKAAARDPGSRAKIAVKTNDKRIDPVGA

250   CVGMRGARVQAVSTELGGERIDIVLWDDNPAQFVINAMAPADVASIVVDE

300   DKHTMDIAVEAGNLAQAIGRNGQNVRLASQLSGWELNVMTVDDLQAKHQA

350   EAHAAIDTFTKYLDIDEDFATVLVEEGFSTLEELAYVPMKELLEIEGLDE

400   PTVEALRERAKNALATIAQAQEESLGDNKPADDLLNLEGVDRDLAFKLAA
```

```
450   RGVCTLEDLAEQGIDDLADIEGLTDEKAGALIMAARNICWFGDEA

sp|P0A9U6|PUUR_ECOLI HTH-type transcriptional regulator PuuR
OS=Escherichia coli (strain K12) OX=83333 GN=puuR PE=1 SV=1

0     MSDEGLAPGKRLSEIRQQQGLSQRRAAELSGLTHSAISTIEQDKVSPAIS
           *********************
50    TLQKLLKVYGLSLSEFFSEPEKPDEPQVVINQDDLIEMGSQGVSMKLVHN

100   GNPNRTLAMIFETYQPGTTTGERIKHQGEEIGTVLEGEIVLTINGQDYHL

150   VAGQSYAINTGIPHSFSNTSAGICRIISAHTPTTF

sp|P75862|ZAPC_ECOLI Cell division protein ZapC OS=Escherichia coli
(strain K12) OX=83333 GN=zapC PE=1 SV=2

0     MRIKPDDNWRWYYDEEHDRMMLDLANGMLFRSRFARKMLTPDAFSPAGFC
            **********************
50    VDDAALYFSFEEKCRDFNLSKEQKAELVLNALVAIRYLKPQMPKSWHFVS

100   HGEMWVPMPGDAACVWLSDTHEQVNLLVVESGENAALCLLAQPCVVIAGR

150   AMQLGDAIKIMNDRLKPQVNVDSFSLEQAV

sp|P76339|HPRS_ECOLI Sensor histidine kinase HprS OS=Escherichia coli
(strain K12) OX=83333 GN=hprS PE=1 SV=1

0     MKRLSITVRLTLLFILLLSVAGAGIVWTLYNGLASELKWRDDTTLINRTA

50    QIKQLLIDGVNPDTLPVYFNRMMDVSQDILIIHGDSINKIVNRTNVSDGM

100   LNNIPASETISAAGIYRSIINDTEIDALRINIDEVSPSLTVTVAKLASAR

150   HNMLEQYKINSIIICIVAIVLCSVLSPLLIRTGLREIKKLSGVTEALNYN

200   DSREPVEVSALPRELKPLGQALNKMHHALVKDFERLSQFADDLAHELRTP

250   INALLGQNQVTLSQTRSIAEYQKTIAGNIEELENISRLTENILFLARADK
                ***********************
300   NNVLVKLDSLSLNKEVENLLDYLEYLSDEKEICFKVECNQQIFADKILLQ

350   RMLSNLIVNAIRYSPEKSRIHITSFLDTNSYLNIDIASPGTKINEPEKLF

400   RRFWRGDNSRHSVGQGLGLSLVKAIAELHGGSATYHYLNKHNVFRITLPQ

450   RN

sp|P28248|DCD_ECOLI dCTP deaminase OS=Escherichia coli (strain K12)
OX=83333 GN=dcd PE=1 SV=1

0     MRLCDRDIEAWLDEGRLSINPRPPVERINGATVDVRLGNKFRTFRGHTAA
        *************************
50    FIDLSGPKDEVSAALDRVMSDEIVLDEGEAFYLHPGELALAVTLESVTLP
```

```
100   ADLVGWLDGRSSLARLGLMVHVTAHRIDPGWSGCIVLEFYNSGKLPLALR

150   PGMLIGALSFEPLSGPAVRPYNRREDAKYRNQQGAVASRIDKD
```

sp|P0A940|BAMA_ECOLI Outer membrane protein assembly factor BamA OS=Escherichia coli (strain K12) OX=83333 GN=bamA PE=1 SV=1

```
0     MAMKKLLIASLLFSSATVYGAEGFVVKDIHFEGLQRVAVGAALLSMPVRT

50    GDTVNDEDISNTIRALFATGNFEDVRVLRDGDTLLVQVKERPTIASITFS

100   GNKSVKDDMLKQNLEASGVRVGESLDRTTIADIEKGLEDFYYSVGKYSAS

150   VKAVVTPLPRNRVDLKLVFQEGVSAEIQQINIVGNHAFTTDELISHFQLR

200   DEVPWWNVVGDRKYQKQKLAGDLETLRSYYLDRGYARFNIDSTQVSLTPD

250   KKGIYVTVNITEGDQYKLSGVEVSGNLAGHSAEIEQLTKIEPGELYNGTK

300   VTKMEDDIKKLLGRYGYAYPRVQSMPEINDADKTVKLRVNVDAGNRFYVR

350   KIRFEGNDTSKDAVLRREMRQMEGAWLGSDLVDQGKERLNRLGFFETVDT

400   DTQRVPGSPDQVDVVYKVKERNTGSFNFGIGYGTESGVSFQAGVQQDNWL

450   GTGYAVGINGTKNDYQTYAELSVTNPYFTVDGVSLGGRLFYNDFQADDAD
                                                     *
500   LSDYTNKSYGTDVTLGFPINEYNSLRAGLGYVHNSLSNMQPQVAMWRYLY
      ********************
550   SMGEHPSTSDQDNSFKTDDFTFNYGWTYNKLDRGYFPTDGSRVNLTGKVT

600   IPGSDNEYYKVTLDTATYVPIDDDHKWVVLGRTRWGYGDGLGGKEMPFYE
                                        ****************
650   NFYAGGSSTVRGFQSNTIGPKAVYFPHQASNYDPDYDYECATQDGAKDLC
      ****
700   KSDDAVGGNAMAVASLEFITPTPFISDKYANSVRTSFFWDMGTVWDTNWD

750   SSQYSGYPDYSDPSNIRMSAGIALQWMSPLGPLVFSYAQPFKKYDGDKAE

800   QFQFNIGKTW
```

sp|P37353|MENE_ECOLI 2-succinylbenzoate--CoA ligase OS=Escherichia coli (strain K12) OX=83333 GN=menE PE=1 SV=2

```
0     MIFSDWPWRHWRQVRGETIALRLNDEQLNWRELCARVDELASGFAVQGVV

50    EGSGVMLRAWNTPQTLLAWLALLQCGARVLPVNPQLPQPLLEELLPNLTL

100   QFALVPDGENTFPALTSLHIQLVEGAHAATWQPTRLCSMTLTSGSTGLPK

150   AAVHTYQAHLASAQGVLSLIPFGDHDDWLLSLPLFHVSGQGIMWRWLYAG

200   ARMTVRDKQPLEQMLAGCTHASLVPTQLWRLLVNRSSVSLKAVLLGGAAI
```

250   PVELTEQAREQGIRCFCGYGLTEFASTVCAKEADGLADVGSPLPGREVKI

300   VNNEVWLRAASMAEGYWRNGQLVSLVNDEGWYATRDRGEMHNGKLTIVGR
                                                  ******
350   LDNLFFSGGEGIQPEEVERVIAAHPAVLQVFIVPVADKEFGHRPVAVMEY
      ****
400   DHESVDLSEWVKDKLARFQQPVRWLTLPPELKNGGIKISRQALKEWVQRQ

450   Q

sp|P0AES6|GYRB_ECOLI DNA gyrase subunit B OS=Escherichia coli (strain K12) OX=83333 GN=gyrB PE=1 SV=2
       ****
0   MSNSYDSSSIKVLKGLDAVRKRPGMYIGDTDDGTGLHHMVFEVVDNAIDE

50   ALAGHCKEIIVTIHADNSVSVQDDGRGIPTGIHPEEGVSAAEVIMTVLHA

100   GGKFDDNSYKVSGGLHGVGVSVVNALSQKLELVIQREGKIHRQIYEHGVP

150   QAPLAVTGETEKTGTMVRFWPSLETFTNVTEFEYEILAKRLRELSFLNSG

200   VSIRLRDKRDGKEDHFHYEGGIKAFVEYLNKNKTPIHPNIFYFSTEKDGI
                                                  ******
250   GVEVALQWNDGFQENIYCFTNNIPQRDGGTHLAGFRAAMTRTLNAYMDKE
      ******************   ****************************
300   GYSKKAKVSATGDDAREGLIAVVSVKVPDPKFSSQTKDKLVSSEVKSAVE
      ******
350   QQMNELLAEYLLENPTDAKIVVGKIIDAARAREAARRAREMTRRKGALDL

400   AGLPGKLADCQERDPALSELYLVEGDSAGGSAKQGRNRKNQAILPLKGKI

450   LNVEKARFDKMLSSQEVATLITALGCGIGRDEYNPDKLRYHSIIIMTDAD
                                       ****
500   VDGSHIRTLLLTFFYRQMPEIVERGHVYIAQPPLYKVKKGKQEQYIKDDE
      *****
550   AMDQYQISIALDGATLHTNASAPALAGEALEKLVSEYNATQKMINRMERR

600   YPKAMLKELIYQPTLTEADLSDEQTVTRWVNALVSELNDKEQHGSQWKFD

650   VHTNAEQNLFEPIVRVRTHGVDTDYPLDHEFITGGEYRRICTLGEKLRGL

700   LEEDAFIERGERRQPVASFEQALDWLVKESRRGLSIQRYKGLGEMNPEQL

750   WETTMDPESRRMLRVTVKDAIAADQLFTTLMGDAVEPRRAFIEENALKAA

800   NIDI

sp|P71297|YAGN_ECOLI Uncharacterized protein YagN OS=Escherichia coli (strain K12) OX=83333 GN=yagN PE=4 SV=1

0   MATPATVSIEPTLAAIRARWCINSSKTTQSFNDPASMEEVVEYLKGTYSA
                                        ****
50   LRKSVACAKLKILHLKQRMQNATNFLARLMSCKNQASRSHHSTAKSAKSA
      ****

```
100   LSSDSGDGSDPDPEPETFPSAFITTPTNSIMLKAFFANISITEVAK

sp|P37903|USPF_ECOLI Universal stress protein F OS=Escherichia coli
(strain K12) OX=83333 GN=uspF PE=1 SV=2
               ***********************
0     MNRTILVPIDISDSELTQRVISHVEEEAKIDDAEVHFLTVIPSLPYYASL
          ******************************
50    GLAYSAELPAMDDLKAEAKSQLEEIIKKFKLPTDRVHVHVEEGSPKDRIL

100   ELAKKIPAHMIIIASHRPDITTYLLGSNAAAVVRHAECSVLVVR

sp|P39286|RSGA_ECOLI Small ribosomal subunit biogenesis GTPase RsgA
OS=Escherichia coli (strain K12) OX=83333 GN=rsgA PE=1 SV=2
                          ************************
0     MSKNKLSKGQQRRVNANHQRRLKTSKEKPDYDDNLFGEPDEGIVISRFGM

50    HADVESADGDVHRCNIRRTIRSLVTGDRVVWRPGKPAAEGVNVKGIVEAV

100   HERTSVLTRPDFYDGVKPIAANIDQIVIVSAILPELSLNIIDRYLVACET

150   LQIEPIIVLNKIDLLDDEGMAFVNEQMDIYRNIGYRVLMVSSHTQDGLKP

200   LEEALTGRISIFAGQSGVGKSSLLNALLGLQKEILTNDISDNSGLGQHTT

250   TAARLYHFPHGGDVIDSPGVREFGLWHLEPEQITQGFVEFHDYLGLCKYR

300   DCKHDTDPGCAIREAVEEGKIAETRFENYHRILESMAQVKTRKNFSDTDD

350

sp|P0AEC5|BARA_ECOLI Signal transduction histidine-protein kinase BarA
OS=Escherichia coli (strain K12) OX=83333 GN=barA PE=1 SV=1

0     MTNYSLRARMMILILAPTVLIGLLLSIFFVVHRYNDLQRQLEDAGASIIE

50    PLAVSTEYGMSLQNRESIGQLISVLHRRHSDIVRAISVYDENNRLFVTSN
                                     *******************
100   FHLDPSSMQLGSNVPFPRQLTVTRDGDIMILRTPIISESYSPDESPSSDA
      ********
150   KNSQNMLGYIALELDLKSVRLQQYKEIFISSVMMLFCIGIALIFGWRLMR

200   DVTGPIRNMVNTVDRIRRGQLDSRVEGFMLGELDMLKNGINSMAMSLAAY

250   HEEMQHNIDQATSDLRETLEQMEIQNVELDLAKKRAQEAARIKSEFLANM

300   SHELRTPLNGVIGFTRLTLKTELTPTQRDHLNTIERSANNLLAIINDVLD

350   FSKLEAGKLILESIPFPLRSTLDEVVTLLAHSSHDKGLELTLNIKSDVPD

400   NVIGDPLRLQQIITNLVGNAIKFTENGNIDILVEKRALSNTKVQIEVQIR

450   DTGIGIPERDQSRLFQAFRQADASISRRHGGTGLGLVITQKLVNEMGGDI

500   SFHSQPNRGSTFWFHINLDLNPNIIIEGPSTQCLAGKRLAYVEPNSAAAQ
```

```
550   CTLDILSETPLEVVYSPTFSALPPAHYDMMLLGIAVTFREPLTMQHERLA

600   KAVSMTDFLMLALPCHAQVNAEKLKQDGIGACLLKPLTPTRLLPALTEFC

650   HHKQNTLLPVTDESKLAMTVMAVDDNPANLKLIGALLEDMVQHVELCDSG

700   HQAVERAKQMPFDLILMDIQMPDMDGIRACELIHQLPHQQQTPVIAVTAH

750   AMAGQKEKLLGAGMSDYLAKPIEEERLHNLLLRYKPGSGISSRVVTPEVN

800   EIVVNPNATLDWQLALRQAAGKTDLARDMLQMLLDFLPEVRNKVEEQLVG
                                                ***************
850   ENPEGLVDLIHKLHGSCGYSGVPRMKNLCQLIEQQLRSGTKEEDLEPELL
      **********
900   ELLDEMDNVAREASKILG
```

sp|P75748|YBGO_ECOLI Uncharacterized protein YbgO OS=Escherichia coli (strain K12) OX=83333 GN=ybgO PE=3 SV=2

```
0     MSAGKGLLLVICLLFLPLKSAMALNCYFGTSGGAVEKSEAIQPFAVPGNA

50    KPGDKIWESDDIKIPVYCDNNTNGNFESEHVYAWVNPYPGVQDRYYQLGV

100   TYNGVDYDASLGKSRIDTNQCIDSKNIDIYTPEQIIAMGWQNKICSGDPA

150   NIHMSRTFLARMRLYVKIREMPPHDYQSTLSDYIVVQFDGAGSVNEDPTA

200   QNLKYHITGLENIRVLDCSVNFSISPETQVIDFGKFNLLDIRRHTMSKTF
                  ************************
250   SIKTTKSQNDQCTDGFKVSSSFYTEETLVEEDKALLIGNGLKLRLLDENA

300   SPYTFNKYAEYADFTSDMLVYEKTYTAELSSIPGTPIEAGPFDTVVLFKI

350   NYN
```

sp|P0AE22|APHA_ECOLI Class B acid phosphatase OS=Escherichia coli (strain K12) OX=83333 GN=aphA PE=1 SV=1

```
0     MRKITQAISAVCLLFALNSSAVALASSPSPLNPGTNVARLAEQAPIHWVS
                                               ****************
50    VAQIENSLAGRPPMAVGFDIDDTVLFSSPGFWRGKKTFSPESEDYLKNPV
      ****
100   FWEKMNNGWDEFSIPKEVARQLIDMHVRRGDAIFFVTGRSPTKTETVSKT

150   LADNFHIPATNMNPVIFAGDKPGQNTKSQWLQDKNIRIFYGDSDNDITAA

200   RDVGARGIRILRASNSTYKPLPQAGAFGEEVIVNSEY
```

sp|P19768|INSJ_ECOLI Insertion element IS150 protein InsJ OS=Escherichia coli (strain K12) OX=83333 GN=insJ PE=3 SV=1

```
0     MSKPKYPFEKRLEVVNHYFTTDDGYRIISARFGVPRTQVRTWVALYEKHG
```

50   EKGLIPKPKGVSADPELRIKVVKAVIEQHMSLNQAAAHFMLAGSGSVARW
                                 ****

100   LKVYEERGEAGLRALKIGTKRNIAISVDPEKAASALELSKDRRIEDLERQ

150   VRFLETRLMYLKKLKALAHPTKK

sp|P09323|PTW3C_ECOLI PTS system N-acetylglucosamine-specific EIICBA component OS=Escherichia coli (strain K12) OX=83333 GN=nagE PE=1 SV=1

0   MNILGFFQRLGRALQLPIAVLPVAALLLRFGQPDLLNVAFIAQAGGAIFD

50   NLALIFAIGVASSWSKDSAGAAALAGAVGYFVLTKAMVTINPEINMGVLA

100   GIITGLVGGAAYNRWSDIKLPDFLSFFGGKRFVPIATGFFCLVLAAIFGY

150   VWPPVQHAIHAGGEWIVSAGALGSGIFGFINRLLIPTGLHQVLNTIAWFQ

200   IGEFTNAAGTVFHGDINRFYAGDGTAGMFMSGFFPIMMFGLPGAALAMYF

250   AAPKERRPMVGGMLLSVAVTAFLTGVTEPLEFLFMFLAPLLYLLHALLTG

300   ISLFVATLLGIHAGFSFSAGAIDYALMYNLPAASQNVWMLLVMGVIFFAI
                   ****

350   YFVVFSLVIRMFNLKTPGREDKEDEIVTEEANSNTEEGLTQLATNYIAAV

400   GGTDNLKAIDACITRLRLTVADSARVNDTMCKRLGASGVVKLNKQTIQVI

450   VGAKAESIGDAMKKVVARGPVAAASAEATPATAAPVAKPQAVPNAVSIAE

500   LVSPITGDVVALDQVPDEAFASKAVGDGVAVKPTDKIVVSPAAGTIVKIF

550   NTNHAFCLETEKGAEIVVHMGIDTVALEGKGFKRLVEEGAQVSAGQPILE

600   MDLDYLNANARSMISPVVCSNIDDFSGLIIKAQGHIVAGQTPLYEIKK

sp|P71298|INTF_ECOLI Prophage integrase IntF OS=Escherichia coli (strain K12) OX=83333 GN=intF PE=3 SV=1

0   MFIPSIYLHQQLHYCKTAILNWSRKMALSRQKFTFERLRRFTLPEGKKQT

50   FLWDADVTTLACRATSGAKAFVFQSVYAGKTLRMTIGNINDWKIDDARAE

100   ARRLQTLIDTGIDPRIAKAVKIAEAESLQAESRKTKVTFSVAWEDYLQEL

150   RTGISAKTKRPYSTRYIADHINLSSRGGESKKRGQGPTSAGPLASLLNLP

200   LSELTPDYIAAWLSTERQNRPTVTAHAYRLLRAFIKWSNYQKKYQGIIPG
                   ****

250   DLAQDYNVRKMVPVSASKADDCLQKEQLKSWFSAVRSLNNPIASAYLQVL
                 ****

300   LLTGARREEIASLRWSDVDFKWSSMRIKDKIEGERIIPLTPYVSELLNVL

350   AQSPNSDVNKEGWVFRSNSKSGKIIEPRSAHNRALVLAELPHISLHGLRR

400   SFGTLAEWVEVPTGIVAQIMGHKPSALAEKHYRRRPLDLLRKWHEKIETW

450   ILNEAGITIKNNVDMR

sp|P03014|PINE_ECOLI Serine recombinase PinE OS=Escherichia coli (strain K12) OX=83333 GN=pinE PE=3 SV=2
****
0   MLIGYVRVSTNDQNTDLQRNALNCAGCELIFEDKISGTKSERPGLKKLLR

50   TLSAGDTLVVWKLDRLGRSMRHLVVLVEELRERGINFRSLTDSIDTSTPM

100   GRFFFHVMGALAEMERELIVERTKAGLETARAQGRIGGRRPKLTPEQWAQ

150   AGRLIAAGTPRQKVAIIYDVGVSTLYKRFPAGDK

sp|P0AFA2|NARX_ECOLI Nitrate/nitrite sensor protein NarX OS=Escherichia coli (strain K12) OX=83333 GN=narX PE=1 SV=1

0   MLKRCLSPLTLVNQVALIVLLSTAIGLAGMAVSGWLVQGVQGSAHAINKA
****
50   GSLRMQSYRLLAAVPLSEKDKPLIKEMEQTAFSAELTRAAERDGQLAQLQ

100   GLQDYWRNELIPALMRAQNRETVSADVSQFVAGLDQLVSGFDRTTEMRIE

150   TVVLVHRVMAVFMALLLVFTIIWLRARLLQPWRQLLAMASAVSHRDFTQR

200   ANISGRNEMAMLGTALNNMSAELAESYAVLEQRVQEKTAGLEHKNQILSF

250   LWQANRRLHSRAPLCERLSPVLNGLQNLTLLRDIELRVYDTDDEENHQEF

300   TCQPDMTCDDKGCQLCPRGVLPVGDRGTTLKWRLADSHTQYGILLATLPQ

350   GRHLSHDQQQLVDTLVEQLTATLALDRHQERQQQLIVMEERATIARELHD

400   SIAQSLSCMKMQVSCLQMQGDALPESSRELLSQIRNELNASWAQLRELLT

450   TFRLQLTEPGLRPALEASCEEYSAKFGFPVKLDYQLPPRLVPSHQAIHLL

500   QIAREALSNALKHSQASEVVVTVAQNDNQVKLTVQDNGCGVPENAIRSNH

550   YGMIIMRDRAQSLRGDCRVRRRESGGTEVVVTFIPEKTFTDVQGDTHE

sp|P13035|GLPD_ECOLI Aerobic glycerol-3-phosphate dehydrogenase OS=Escherichia coli (strain K12) OX=83333 GN=glpD PE=1 SV=3

0   METKDLIVIGGGINGAGIAADAAGRGLSVLMLEAQDLACATSSASSKLIH

50   GGLRYLEHYEFRLVSEALAEREVLLKMAPHIAFPMRFRLPHRPHLRPAWM

100   IRIGLFMYDHLGKRTSLPGSTGLRFGANSVLKPEIKRGFEYSDCWVDDAR

150   LVLANAQMVVRKGGEVLTRTRATSARRENGLWIVEAEDIDTGKKYSWQAR

200   GLVNATGPWVKQFFDDGMHLPSPYGIRLIKGSHIVVPRVHTQKQAYILQN

****
250   EDKRIVFVIPWMDEFSIIGTTDVEYKGDPKAVKIEESEINYLLNVYNTHF

300   KKQLSRDDIVWTYSGVRPLCDDESDSPQAITRDYTLDIHDENGKAPLLSV

350   FGGKLTTYRKLAEHALEKLTPYYQGIGPAWTKESVLPGGAIEGDRDDYAA

400   RLRRRYPFLTESLARHYARTYGSNSELLLGNAGTVSDLGEDFGHEFYEAE

450   LKYLVDHEWVRRADDALWRRTKQGMWLNADQQSRVSQWLVEYTQQRLSLA

500   S

sp|P07004|PROA_ECOLI Gamma-glutamyl phosphate reductase OS=Escherichia coli (strain K12) OX=83333 GN=proA PE=1 SV=2

****
0   MLEQMGIAAKQASYKLAQLSSREKNRVLEKIADELEAQSEIILNANAQDV

50   ADARANGLSEAMLDRLALTPARLKGIADDVRQVCNLADPVGQVIDGGVLD

100   SGLRLERRRVPLGVIGVIYEARPNVTVDVASLCLKTGNAVILRGGKETCR

150   TNAATVAVIQDALKSCGLPAGAVQAIDNPDRALVSEMLRMDKYIDMLIPR

200   GGAGLHKLCREQSTIPVITGGIGVCHIYVDESVEIAEALKVIVNAKTQRP

250   STCNTVETLLVNKNIADSFLPALSKQMAESGVTLHADAAALAQLQAGPAK

****
300   VVAVKAEEYDDEFLSLDLNVKIVSDLDDAIAHIREHGTQHSDAILTRDMR

350   NAQRFVNEVDSSAVYVNASTRFTDGGQFGLGAEVAVSTQKLHARGPMGLE

400   ALTTYKWIGIGDYTIRA

sp|P76486|YFBP_ECOLI Uncharacterized protein YfbP OS=Escherichia coli (strain K12) OX=83333 GN=yfbP PE=4 SV=2

0   MKLIPRSSDISPGIDGICPGPFPPNGFTVLTDAAYGNGDCFGLYWPIGQE

****
50   HKLPIVCETYHDEWRIVPAFSSIKKFEEWLEVNDDDPHENGISIEDQDFA

100   ANLFRVARKCLSTGRLDDALPLLQRATEQLPEVSEYWLALAIQYRRCKKT

150   EAAAQAALNAYLGNWAFGVPDNKVIHLLSQAADVPNFQDDPVIQCIKEQG

200   LDLSFGGTKENNNYPLMQMCVDTYFAQRKPLQALTLLHNYAWIMSSETTA

250   FQERYDFNIDEWRAKFRQLCLEYFGDSRTQFT

sp|P27126|RFAS_ECOLI Lipopolysaccharide core biosynthesis protein RfaS OS=Escherichia coli (strain K12) OX=83333 GN=rfaS PE=4 SV=1

0   MTIYFINWVADYELKMIQYLKKKYKIKNITTPKKYNWINKKISKIGMDNA

```
50    WLGRLFIKHYLNDIKKDDIIIINDSVVNKGINKQILKNINCHKVLLLRNT

100   VGEDFILDNANYFDIIYDFEHRFIGNEKIKAIEQFFPIGMDEIRNYSLSD
                                         ***************
150   KNNSQPICFFLGRDKGRLQIINELAERLTTLGCKLDFNVVKDKTSSTTSK
      ************
200   YLIEKQISYEENIRRTLNANIIVDITKENQSGWTLRILEALFFNKKLITN
                          ******************************
250   NINVFGSEIYSESRFFIIGHDDWDKLEYFINSSVKPMDYDSLYKFSPDKM
      *******
300   MSTIVSDFIDK
```

sp|P39385|YJIN_ECOLI Uncharacterized protein YjiN OS=Escherichia coli (strain K12) OX=83333 GN=yjiN PE=4 SV=1

```
0     MNKLIELRRAKRLALSLLLIAAATFVVTLFLPPNFWVSGVKAIAEAAMVG

50    ALADWFAVVALFRRVPIPIISRHTAIIPRNKDRIGENLGQFVQEKFLDTQ

100   SLVALIRRHEPALLIGNWFSQPENARRVGQHLLQIMSGFLELTDDARIQR

150   LLKRAVHRAIDKVDLSGTSALMLESMTKNDRHQVLLDTLIAQLIALLQRD

200   KSRKFIAQQIVRWLESEHPLKAKILPTEWLGEHSAELVSDAVNSLLDDIS
                                                       *
250   RDRAHQIRHAFDRATFALIDKLKNDPEMAARADAVKSYLKEDEAFNRYLS
      ************************
300   ELWGDLREWLKVDINSEDSRVKERIARAGQWFGETLIADDALRASLNGHL

350   EQAAHRVAPEFSAFLTRHISDTVKSWDARDMSRQIELNIGKDLQFIRVNG

400   TLVGGCIGLILYLLSQLPALFPLGNF
```

sp|P0C037|PPNP_ECOLI Pyrimidine/purine nucleoside phosphorylase OS=Escherichia coli (strain K12) OX=83333 GN=ppnP PE=1 SV=1

```
       **********************
0     MLQSNEYFSGKVKSIGFSSSSTGRASVGVMVEGEYTFSTAEPEEMTVISG

50    ALNVLLPDATDWQVYEAGSVFNVPGHSEFHLQVAEPTSYLCRYL
```

sp|P39453|TORS_ECOLI Sensor protein TorS OS=Escherichia coli (strain K12) OX=83333 GN=torS PE=1 SV=4

```
0     MNLTLTRRLWMGFALMALLTLTSTLVGWYNLRFISQVEKDNTQALIPTMN

50    MARQLSEASAWELFAAQNLTSADNEKMWQAQGRMLTAQSLKINALLQALR

100   EQGFDTTAIEQQEQEISRSLRQQGELVGQRLQLRQQQQQLSQQIVAAADE

150   IARLAQGQANNATTSAGATQAGIYDLIEQDQRQAAESALDRLIDIDLEYV

200   NQMNELRLSALRVQQMVMNLGLEQIQKNAPTLEKQLNNAVKILQRRQIRI

250   EDPGVRAQVATTLTTVSQYSDLLALYQQDSEISNHLQTLAQNNIAQFAQF
```

300   SSEVSQLVDTIELRNQHGLAHLEKASARGQYSLLLLGMVSLCALILILWR

350   VVYRSVTRPLAEQTQALQRLLDGDIDSPFPETAGVRELDTIGRLMDAFRS

400   NVHALNRHREQLAAQVKARTAELQELVIEHRQARAEAEKASQAKSAFLAA

450   MSHEIRTPLYGILGTAQLLADNPALNAQRDDLRAITDSGESLLTILNDIL

500   DYSAIEAGGKNVSVSDEPFEPRPLLESTLQLMSGRVKGRPIRLATAIADD

550   MPCALMGDPRRIRQVITNLLSNALRFTDEGYIILRSRTDGEQWLVEVEDS

600   GCGIDPAKLAEIFQPFVQVSGKRGGTGLGLTISSRLAQAMGGELSATSTP

650   EVGSCFCLRLPLRVATAPVPKTVNQAVRLDGLRLLLIEDNPLTQRITIEM

700   LKTSGAQIVAVGNAAQALETLQNSEPFAAALVDFDLPDIDGITLARQLAQ

750   QYPSLVLIGFSAHVIDETLRQRTSSLFRGIIPKPVPREVLGQLLAHYLQL
***

800   QVNNDQSLDVSQLNEDAQLMGTEKIHEWLVLFTQHALPLLDEIDIARASQ
****

850   DSEKIKRAAHQLKSSCSSLGMHIASQLCAQLEQQPLSAPLPHEEITRSVA

900   ALEAWLHKKDLNAI

sp|P00582|DPO1_ECOLI DNA polymerase I OS=Escherichia coli (strain K12) OX=83333 GN=polA PE=1 SV=1

0   MVQIPQNPLILVDGSSYLYRAYHAFPPLTNSAGEPTGAMYGVLNMLRSLI

50   MQYKPTHAAVVFDAKGKTFRDELFEHYKSHRPPMPDDLRAQIEPLHAMVK

100   AMGLPLLAVSGVEADDVIGTLAREAEKAGRPVLISTGDKDMAQLVTPNIT

150   LINTMTNTILGPEEVVNKYGVPPELIIDFLALMGDSSDNIPGVPGVGEKT

200   AQALLQGLGGLDTLYAEPEKIAGLSFRGAKTMAAKLEQNKEVAYLSYQLA

250   TIKTDVELELTCEQLEVQQPAAEELLGLFKKYEFKRWTADVEAGKWLQAK

300   GAKPAAKPQETSVADEAPEVTATVISYDNYVTILDEETLKAWIAKLEKAP

350   VFAFDTETDSLDNISANLVGLSFAIEPGVAAYIPVAHDYLDAPDQISRER

400   ALELLKPLLEDEKALKVGQNLKYDRGILANYGIELRGIAFDTMLESYILN

450   SVAGRHDMDSLAERWLKHKTITFEEIAGKGKNQLTFNQIALEEAGRYAAE

500   DADVTLQLHLKMWPDLQKHKGPLNVFENIEMPLVPVLSRIERNGVKIDPK
****

550   VLHNHSEELTLRLAELEKKAHEIAGEEFNLSSTKQLQTILFEKQGIKPLK

600   KTPGGAPSTSEEVLEELALDYPLPKVILEYRGLAKLKSTYTDKLPLMINP

650   KTGRVHTSYHQAVTATGRLSSTDPNLQNIPVRNEEGRRIRQAFIAPEDYV

700   IVSADYSQIELRIMAHLSRDKGLLTAFAEGKDIHRATAAEVFGLPLETVT

750   SEQRRSAKAINFGLIYGMSAFGLARQLNIPRKEAQKYMDLYFERYPGVLE

800   YMERTRAQAKEQGYVETLDGRRLYLPDIKSSNGARRAAAERAAINAPMQG

850   TAADIIKRAMIAVDAWLQAEQPRVRMIMQVHDELVFEVHKDDVDAVAKQI

900   HQLMENCTRLDVPLLVEVGSGENWDQAH

sp|P43672|UUP_ECOLI ABC transporter ATP-binding protein uup OS=Escherichia coli (strain K12) OX=83333 GN=uup PE=1 SV=2

0   MSLISMHGAWLSFSDAPLLDNAELHIEDNERVCLVGRNGAGKSTLMKILN

50   REQGLDDGRIIYEQDLIVARLQQDPPRNVEGSVYDFVAEGIEEQAEYLKR

100   YHDISRLVMNDPSEKNLNELAKVQEQLDHHNLWQLENRINEVLAQLGLDP

150   NVALSSLSGGWLRKAALGRALVSNPRVLLLDEPTNHLDIETIDWLEGFLK

200   TFNGTIIFISHDRSFIRNMATRIVDLDRGKLVTYPGNYDQYLLEKEEALR

250   VEELQNAEFDRKLAQEEVWIRQGIKARRTRNEGRVRALKAMRRERGERRE

300   VMGTAKMQVEEASRSGKIVFEMEDVCYQVNGKQLVKDFSAQVLRGDKIAL

350   IGPNGCGKTTLLKLMLGQLQADSGRIHVGTKLEVAYFDQHRAELDPDKTV

400   MDNLAEGKQEVMVNGKPRHVLGYLQDFLFHPKRAMTPVRALSGGERNRLL

450   LARLFLKPSNLLILDEPTNDLDVETLELLEELIDSYQGTVLLVSHDRQFV
                                                ****
500   DNTVTECWIFEGGGKIGRYVGGYHDARGQQEQYVALKQPAVKKTEEAAAA
      ****
550   KAETVKRSSSKLSYKLQRELEQLPQLLEDLEAKLEALQTQVADASFFSQP

600   HEQTQKVLADMAAAEQELEQAFERWEYLEALKNGG

sp|Q47083|CBL_ECOLI HTH-type transcriptional regulator cbl OS=Escherichia coli (strain K12) OX=83333 GN=cbl PE=1 SV=2

0   MNFQQLKIIREAARQDYNLTEVANMLFTSQSGVSRHIRELEDELGIEIFV

50   RRGKRLLGMTEPGKALLVIAERILNEASNVRRLADLFTNDTSGVLTIATT

100   HTQARYSLPEVIKAFRELFPEVRLELIQGTPQEIATLLQNGEADIGIASE

150   RLSNDPQLVAFPWFRWHHSLLVPHDHPLTQISPLTLESIAKWPLITYRQG

```
200   ITGRSRIDDAFARKGLLADIVLSAQDSDVIKTYVALGLGIGLVAEQSSGE
                                                *********
250   QEEENLIRLDTRHLFDANTVWLGLKRGQLQRNYVWRFLELCNAGLSVEDI
      ****************
300   KRQVMESSEEEIDYQI
```

sp|P77338|MSCK_ECOLI Mechanosensitive channel MscK OS=Escherichia coli (strain K12) OX=83333 GN=mscK PE=1 SV=1

```
0     MTMFQYYKRSRHFVFSAFIAFVFVLLCQNTAFARASSNGDLPTKADLQAQ

50    LDSLNKQKDLSAQDKLVQQDLTDTLATLDKIDRIKEETVQLRQKVAEAPE

100   KMRQATAALTALSDVDNDEETRKILSTLSLRQLETRVAQALDDLQNAQND

150   LASYNSQLVSLQTQPERVQNAMYNASQQLQQIRSRLDGTDVGETALRPSQ

200   KVLMQAQQALLNAEIDQQRKSLEGNTVLQDTLQKQRDYVTANSARLEHQL

250   QLLQEAVNSKRLTLTEKTAQEAVSPDEAARIQANPLVKQELEINQQLSQR

300   LITATENGNQLMQQNIKVKNWLERALQSERNIKEQIAVLKGSLLLSRILY

350   QQQQTLPSADELENMTNRIADLRLEQFEVNQQRDALFQSDAFVNKLEEGH

400   TNEVNSEVHDALLQVVDMRRELLDQLNKQLGNQLMMAINLQINQQQLMSV
                           ********************
450   SKNLKSILTQQIFWVNSNRPMDWDWIKAFPQSLKDEFKSMKITVNWQKAW

500   PAVFIAFLAGLPLLLIAGLIHWRLGWLKAYQQKLASAVGSLRNDSQLNTP

550   KAILIDLIRALPVCLIIILAVGLILLTMQLNISELLWSFSKKLAIFWLVFG

600   LCWKVLEKNGVAVRHFGMPEQQTSHWRRQIVRISLALLPIHFWSVVAELS

650   PLHLMDDVLGQAMIFFNLLLIAFLVWPMCRESWRDKESHTMRLVTITVLS

700   IIPIALMVLTATGYFYTTLRLAGRWIETVYLVIIWNLLYQTVLRGLSVAA

750   RRIAWRRALARRQNLVKEGAEGAEPPEEPTIALEQVNQQTLRITMLLMFA

800   LFGVMFWAIWSDLITVFSYLDSITLWHYNGTEAGAAVVKNVTMGSLLFAI

850   IASMVAWALIRNLPGLLEVLVLSRLNMRQGASYAITTILNYIIIAVGAMT

900   VFGSLGVSWDKLQWLAAALSVGLGFGLQEIFGNFVSGLIIILFERPVRIGD

950   TVTIGSFSGTVSKIRIRATTITDFDRKEVIIPNKAFVTERLINWSLTDTT

1000  TRLVIRLGVAYGSDLEKVRKVLLKAATEHPRVMHEPMPEVFFTAFGASTL
                                                  ******
1050  DHELRLYVRELRDRSRTVDELNRTIDQLCRENDINIAFNQLEVHLHNEKG
      *****************
1100  DEVTEVKRDYKGDDPTPAVG
```

```
sp|P0A6V1|GLGC_ECOLI Glucose-1-phosphate adenylyltransferase
OS=Escherichia coli (strain K12) OX=83333 GN=glgC PE=1 SV=2

0     MVSLEKNDHLMLARQLPLKSVALILAGGRGTRLKDLTNKRAKPAVHFGGK

50    FRIIDFALSNCINSGIRRMGVITQYQSHTLVQHIQRGWSFFNEEMNEFVD

100   LLPAQQRMKGENWYRGTADAVTQNLDIIRRYKAEYVVILAGDHIYKQDYS

150   RMLIDHVEKGARCTVACMPVPIEEASAFGVMAVDENDKIIEFVEKPANPP
                             ****************************
200   SMPNDPSKSLASMGIYVFDADYLYELLEEDDRDENSSHDFGKDLIPKITE

250   AGLAYAHPFPLSCVQSDPDAEPYWRDVGTLEAYWKANLDLASVVPELDMY

300   DRNWPIRTYNESLPPAKFVQDRSGSHGMTLNSLVSGGCVISGSVVVQSVL

350   FSRVRVNSFCNIDSAVLLPEVWVGRSCRLRRCVIDRACVIPEGMVIGENA

400   EEDARRFYRSEEGIVLVTREMLRKLGHKQER

sp|P0A7G6|RECA_ECOLI Protein RecA OS=Escherichia coli (strain K12)
OX=83333 GN=recA PE=1 SV=2

0     MAIDENKQKALAAALGQIEKQFGKGSIMRLGEDRSMDVETISTGSLSLDI

50    ALGAGGLPMGRIVEIYGPESSGKTTLTLQVIAAAQREGKTCAFIDAEHAL

100   DPIYARKLGVDIDNLLCSQPDTGEQALEICDALARSGAVDVIVVDSVAAL

150   TPKAEIEGEIGDSHMGLAARMMSQAMRKLAGNLKQSNTLLIFINQIRMKI

200   GVMFGNPETTTGGNALKFYASVRLDIRRIGAVKEGENVVGSETRVKVVKN

250   KIAAPFKQAEFQILYGEGINFYGELVDLGVKEKLIEKAGAWYSYKGEKIG
                ********************
300   QGKANATAWLKDNPETAKEIEKKVRELLLSNPNSTPDFSVDDSEGVAETN

350   EDF

sp|P0AAJ5|FDOH_ECOLI Formate dehydrogenase-O iron-sulfur subunit
OS=Escherichia coli (strain K12) OX=83333 GN=fdoH PE=1 SV=1

0     MAYQSQDIIRRSATNGLTPAPQARDFQEEVAKLIDVTTCIGCKACQVACS

50    EWNDIRDTVGNNIGVYDNPNDLSAKSWTVMRFSEVEQNDKLEWLIRKDGC

100   MHCSDPGCLKACPAEGAIIQYANGIVDFQSEQCIGCGYCIAGCPFDIPRL

150   NPEDNRVYKCTLCVDRVVVGQEPACVKTCPTGAIHFGTKESMKTLASERV

200   AELKTRGYDNAGLYDPAGVGGTHVMYVLHHADKPNLYHGLPENPEISETV
                                  **********************
```

250   KFWKGIWKPLAAVGFAATFAASIFHYVGVGPNRADEEENNLHEEKDEERK

300

sp|P37639|GADX_ECOLI HTH-type transcriptional regulator GadX
OS=Escherichia coli (strain K12) OX=83333 GN=gadX PE=1 SV=1

0   MQSLHGNCLIAYARHKYILTMVNGEYRYFNGGDLVFADASQIRVDKCVEN

50   FVFVSRDTLSLFLPMLKEEALNLHAHKKVSSLLVHHCSRDIPVFQEVAQL

100   SQNKNLRYAEMLRKRALIFALLSVFLEDEHFIPLLLNVLQPNMRTRVCTV
                   ****
150   INNNIAHEWTLARIASELLMSPSLLKKKLREEETSYSQLLTECRMQRALQ

200   LIVIHGFSIKRVAVSCGYHSVSYFIYVFRNYYGMTPTEYQERSAQRLSNR

250   DSAASIVAQGNFYGTDRSAEGIRL

sp|P19636|EUTC_ECOLI Ethanolamine ammonia-lyase light chain
OS=Escherichia coli (strain K12) OX=83333 GN=eutC PE=1 SV=2

0   MDQKQIEEIVRSVMASMGQAAPAPSEAKCATTNCAAPVTSESCALDLGSA
                                                    ****
50   EAKAWIGVENPHRADVLTELRRSTVARVCTGRAGPRPRTQALLRFLADHS
      ****
100   RSKDTVLKEVPEEWVKAQGLLEVRSEISDKNLYLTRPDMGRRLCAEAVEA

150   LKAQCVANPDVQVVISDGLSTDAITVNYEEILPPLMAGLKQAGLKVGTPF

200   FVRYGRVKIEDQIGEILGAKVVILLVGERPGLGQSESLSCYAVYSPRMAT

250   TVEADRTCISNIHQGGTPPVEAAAVIVDLAKRMLEQKASGINMTR

sp|P26218|BGLH_ECOLI Cryptic outer membrane porin BglH OS=Escherichia
coli (strain K12) OX=83333 GN=bglH PE=1 SV=3
                                       ****
0   MFRRNLITSAILLMAPLAFSAQSLAESLTVEQRLELLEKALRETQSELKK
      *******************   ******************
50   YKDEEKKKYTPATVNRSVSTNDQGYAANPFPTSSAAKPDAVLVKNEEKNA
      ****
100   SETGSIYSSMTLKDFSKFVKDEIGFSYNGYYRSGWGTASHGSPKSWAIGS

150   LGRFGNEYSGWFDLQLKQRVYNENGKRVDAVVMMDGNVGQQYSTGWFGDN

200   AGGENYMQFSDMYVTTKGFLPFAPEADFWVGKHGAPKIEIQMLDWKTQRT

250   DAAAGVGLENWKVGPGKIDIALVREDVDDYDRSLQNKQQINTNTIDLRYK
                         ****
300   DIPLWDKATLMVSGRYVTANESASEKDNQDNNGYYDWKDTWMFGTSLTQK

350   FDKGGFNEFSFLVANNSIASNFGRYAGASPFTTFNGRYYGDHTGGTAVRL

400   TSQGEAYIGDHFIVANAIVYSFGNDIYSYETGAHSDFESIRAVVRPAYIW

```
450   DQYNQTGVELGYFTQQNKDANSNKFNESGYKTTLFHTFKVNTSMLTSRPE

500   IRFYATYIKALENELDGFTFEDNKDDQFAVGAQAEIWW
```

sp|P27550|ACSA_ECOLI Acetyl-coenzyme A synthetase OS=Escherichia coli (strain K12) OX=83333 GN=acs PE=1 SV=2

```
0     MSQIHKHTIPANIADRCLINPQQYEAMYQQSINVPDTFWGEQGKILDWIK
                                                  ****
50    PYQKVKNTSFAPGNVSIKWYEDGTLNLAANCLDRHLQENGDRTAIIWEGD
      *****************
100   DASQSKHISYKELHRDVCRFANTLLELGIKKGDVVAIYMPMVPEAAVAML

150   ACARIGAVHSVIFGGFSPEAVAGRIIDSNSRLVITSDEGVRAGRSIPLKK

200   NVDDALKNPNVTSVEHVVVLKRTGGKIDWQEGRDLWWHDLVEQASDQHQA

250   EEMNAEDPLFILYTSGSTGKPKGVLHTTGGYLVYAALTFKYVFDYHPGDI

300   YWCTADVGWVTGHSYLLYGPLACGATTLMFEGVPNWPTPARMAQVVDKHQ

350   VNILYTAPTAIRALMAEGDKAIEGTDRSSLRILGSVGEPINPEAWEWYWK

400   KIGNEKCPVVDTWWQTETGGFMITPLPGATELKAGSATRPFFGVQPALVD

450   NEGNPLEGATEGSLVITDSWPGQARTLFGDHERFEQTYFSTFKNMYFSGD

500   GARRDEDGYYWITGRVDDVLNVSGHRLGTAEIESALVAHPKIAEAAVVGI

550   PHNIKGQAIYAYVTLNHGEEPSPELYAEVRNWVRKEIGPLATPDVLHWTD

600   SLPKTRSGKIMRRILRKIAAGDTSNLGDTSTLADPGVVEKLLEEKQAIAM

650   PS
```

sp|P24232|HMP_ECOLI Flavohemoprotein OS=Escherichia coli (strain K12) OX=83333 GN=hmp PE=1 SV=1

```
0     MLDAQTIATVKATIPLLVETGPKLTAHFYDRMFTHNPELKEIFNMSNQRN

50    GDQREALFNAIAAYASNIENLPALLPAVEKIAQKHTSFQIKPEQYNIVGE

100   HLLATLDEMFSPGQEVLDAWGKAYGVLANVFINREAEIYNENASKAGGWE

150   GTRDFRIVAKTPRSALITSFELEPVDGGAVAEYRPGQYLGVWLKPEGFPH

200   QEIRQYSLTRKPDGKGYRIAVKREEGGQVSNWLHNHANVGDVVKLVAPAG

250   DFFMAVADDTPVTLISAGVGQTPMLAMLDTLAKAGHTAQVNWFHAAENGD
                                 ********************
300   VHAFADEVKELGQSLPRFTAHTWYRQPSEADRAKGQFDSEGLMDLSKLEG

350   AFSDPTMQFYLCGPVGFMQFTAKQLVDLGVKQENIHYECFGPHKVL
```

```
sp|Q46793|YGEN_ECOLI Putative uncharacterized protein YgeN OS=Escherichia
coli (strain K12) OX=83333 GN=ygeN PE=5 SV=3
                      **********************************
0     MRKKIEMSLIKSPANGVVIKRKISDGLKEIVSLKEKILLETTAKIQSIEE
      *****                                          ***
50    KREEKFIQGYYDGYTKGIIDEMDNFIPLISLLCSELEKKRINMINDLKSI
      *****************              *******************
100   LLKPSEEVDVFIKIFESWVTKLPSISGPVNLHIPTSFKDKSLEVESYFVD
      *******
150   KSIWNVHITFHDDKRFVFFTDQFIAEFSPQEFVDNCEQYLINNHCFSPDK

200   VNEICEQARHYLVEKMFETHSLDMNNSVLASPEDL

sp|P31434|XYLS_ECOLI Alpha-xylosidase OS=Escherichia coli (strain K12)
OX=83333 GN=yicI PE=1 SV=2

0     MKISDGNWLIQPGLNLIHPLQVFEVEQQDNEMVVYAAPRDVRERTWQLDT

50    PLFTLRFFSPQEGIVGVRIEHFQGALNNGPHYPLNILQDVKVTIENTERY

100   AEFKSGNLSARVSKGEFWSLDFLRNGERITGSQVKNNGYVQDTNNQRNYM

150   FERLDLGVGETVYGLGERFTALVRNGQTVETWNRDGGTSTEQAYKNIPFY
                    ***************************
200   MTNRGYGVLVNHPQCVSFEVGSEKVSKVQFSVESEYLEYFVIDGPTPKAV

250   LDRYTRFTGRPALPPAWSFGLWLTTSFTTNYDEATVNSFIDGMAERNLPL

300   HVFHFDCFWMKAFQWCDFEWDPLTFPDPEGMIRRLKAKGLKICVWINPYI

350   GQKSPVFKELQEKGYLLKRPDGSLWQWDKWQPGLAIYDFTNPDACKWYAD

400   KLKGLVAMGVDCFKTDFGERIPTDVQWFDGSDPQKMHNHYAYIYNELVWN

450   VLKDTVGEEEAVLFARSASVGAQKFPVHWGGDCYANYESMAESLRGGLSI

500   GLSGFGFWSHDIGGFENTAPAHVYKRWCAFGLLSSHSRLHGSKSYRVPWA

550   YDDESCDVVRFFTQLKCRMMPYLYREAARANARGTPMMRAMMMEFPDDPA

600   CDYLDRQYMLGDNVMVAPVFTEAGDVQFYLPEGRWTHLWHNDELDGSRWH

650   KQQHGFLSLPVYVRDNTLLALGNNDQRPDYVWHEGTAFHLFNLQDGHEAV

700   CEVPAADGSVIFTLKAARTGNTITVTGAGEAKNWTLCLRNVVKVNGLQDG

750   SQAESEQGLVVKPQGNALTITL

sp|P20083|PARE_ECOLI DNA topoisomerase 4 subunit B OS=Escherichia coli
(strain K12) OX=83333 GN=parE PE=1 SV=3

0     MTQTYNADAIEVLTGLEPVRRRPGMYTDTTRPNHLGQEVIDNSVDEALAG
```

50 HAKRVDVILHADQSLEVIDDGRGMPVDIHPEEGVPAVELILCRLHAGGKF

100 SNKNYQFSGGLHGVGISVVNALSKRVEVNVRRDGQVYNIAFENGEKVQDL

150 QVVGTCGKRNTGTSVHFWPDETFFDSPRFSVSRLTHVLKAKAVLCPGVEI

200 TFKDEINNTEQRWCYQDGLNDYLAEAVNGLPTLPEKPFIGNFAGDTEAVD

250 WALLWLPEGGELLTESYVNLIPTMQGGTHVNGLRQGLLDAMREFCEYRNI

300 LPRGVKLSAEDIWDRCAYVLSVKMQDPQFAGQTKERLSSRQCAAFVSGVV

350 KDAFILWLNQNVQAAELLAEMAISSAQRRMRAAKKVVRKKLTSGPALPGK

400 LADCTAQDLNRTELFLVEGDSAGGSAKQARDREYQAIMPLKGKILNTWEV

450 SSDEVLASQEVHDISVAIGIDPDSDDLSQLRYGKICILADADSDGLHIAT
****
500 LLCALFVKHFRALVKHGHVYVALPPLYRIDLGKEVYYALTEEEKEGVLEQ
****
550 LKRKKGKPNVQRFKGLGEMNPMQLRETTLDPNTRRLVQLTIDDEDDQRTD

600 AMMDMLLAKKRSEDRRNWLQEKGDMAEIEV

sp|P00926|SDHD_ECOLI D-serine dehydratase OS=Escherichia coli (strain K12) OX=83333 GN=dsdA PE=1 SV=3

0 MENAKMNSLIAQYPLVKDLVALKETTWFNPGTTSLAEGLPYVGLTEQDVQ

50 DAHARLSRFAPYLAKAFPETAATGGIIESELVAIPAMQKRLEKEYQQPIS
****
100 GQLLLKKDSHLPISGSIKARGGIYEVLAHAEKLALEAGLLTLDDDYSKLL
****
150 SPEFKQFFSQYSIAVGSTGNLGLSIGIMSARIGFKVTVHMSADARAWKKA

200 KLRSHGVTVVEYEQDYGVAVEEGRKAAQSDPNCFFIDDENSRTLFLGYSV

250 AGQRLKAQFAQQGRIVDADNPLFVYLPCGVGGGPGGVAFGLKLAFGDHVH

300 CFFAEPTHSPCMLLGVHTGLHDQISVQDIGIDNLTAADGLAVGRASGFVG

350 RAMERLLDGFYTLSDQTMYDMLGWLAQEEGIRLEPSALAGMAGPQRVCAS

400 VSYQQMHGFSAEQLRNTTHLVWATGGGMVPEEEMNQYLAKGR

sp|P02919|PBPB_ECOLI Penicillin-binding protein 1B OS=Escherichia coli (strain K12) OX=83333 GN=mrcB PE=1 SV=2
****
0 MAGNDREPIGRKGKPTRPVKQKVSRRRYEDDDDYDDYDDYEDEEPMPRKG
****
50 KGKGKGRKPRGKRGWLWLLLKLAIVFAVLIAIYGVYLDQKIRSRIDGKVW

100 QLPAAVYGRMVNLEPDMTISKNEMVKLLEATQYRQVSKMTRPGEFTVQAN

150   SIEMIRRPFDFPDSKEGQVRARLTFDGDHLATIVNMENNRQFGFFRLDPR

200   LITMISSPNGEQRLFVPRSGFPDLLVDTLLATEDRHFYEHDGISLYSIGR

250   AVLANLTAGRTVQGASTLTQQLVKNLFLSSERSYWRKANEAYMALIMDAR

300   YSKDRILELYMNEVYLGQSGDNEIRGFPLASLYYFGRPVEELSLDQQALL

350   VGMVKGASIYNPWRNPKLALERRNLVLRLLQQQQIIDQELYDMLSARPLG

400   VQPRGGVISPQPAFMQLVRQELQAKLGDKVKDLSGVKIFTTFDSVAQDAA

450   EKAAVEGIPALKKQRKLSDLETAIVVVDRFSGEVRAMVGGSEPQFAGYNR

500   AMQARRSIGSLAKPATYLTALSQPKIYRLNTWIADAPIALRQPNGQVWSP

550   QNDDRRYSESGRVMLVDALTRSMNVPTVNLGMALGLPAVTETWIKLGVPK

600   DQLHPVPAMLLGALNLTPIEVAQAFQTIASGGNRAPLSALRSVIAEDGKV

650   LYQSFPQAERAVPAQAAYLTLWTMQQVVQRGTGRQLGAKYPNLHLAGKTG

700   TTNNNVDTWFAGIDGSTVTITWVGRDNNQPTKLYGASGAMSIYQRYLANQ

750   TPTPLNLVPPEDIADMGVDYDGNFVCSGGMRILPVWTSDPQSLCQQSEMQ

800   QQPSGNPFDQSSQPQQQPQQQPAQQEQKDSDGVAGWIKDMFGSN

sp|P0ABI8|CYOB_ECOLI Cytochrome bo(3) ubiquinol oxidase subunit 1
OS=Escherichia coli (strain K12) OX=83333 GN=cyoB PE=1 SV=1

0   MFGKLSLDAVPFHEPIVMVTIAGIILGGLALVGLITYFGKWTYLWKEWLT

50   SVDHKRLGIMYIIVAIVMLLRGFADAIMMRSQQALASAGEAGFLPPHHYD

100   QIFTAHGVIMIFFVAMPFVIGLMNLVVPLQIGARDVAFPFLNNLSFWFTV

150   VGVILVNVSLGVGEFAQTGWLAYPPLSGIEYSPGVGVDYWIWSLQLSGIG

200   TTLTGINFFVTILKMRAPGMTMFKMPVFTWASLCANVLIIASFPILTVTV

250   ALLTLDRYLGTHFFTNDMGGNMMMYINLIWAWGHPEVYILILPVFGVFSE

300   IAATFSRKRLFGYTSLVWATVCITVLSFIVWLHHFFTMGAGANVNAFFGI

350   TTMIIAIPTGVKIFNWLFTMYQGRIVFHSAMLWTIGFIVTFSVGGMTGVL

400   LAVPGADFVLHNSLFLIAHFHNVIIGGVVFGCFAGMTYWWPKAFGFKLNE

450   TWGKRAFWFWIIGFFVAFMPLYALGFMGMTRRLSQQIDPQFHTMLMIAAS

500   GAVLIALGILCLVIQMYVSIRDRDQNRDLTGDPWGGRTLEWATSSPPPFY
              ****

550   NFAVVPHVHERDAFWEMKEKGEAYKKPDHYEEIHMPKNSGAGIVIAAFST

```
                                       ********************
600   IFGFAMIWHIWWLAIVGFAGMIITWIVKSFDEDVDYYVPVAEIEKLENQH

650   FDEITKAGLKNGN
```

sp|P10408|SECA_ECOLI Protein translocase subunit SecA OS=Escherichia coli (strain K12) OX=83333 GN=secA PE=1 SV=2

```
                                    ************************
0     MLIKLLTKVFGSRNDRTLRRMRKVVNIINAMEPEMEKLSDEELKGKTAEF
      ********
50    RARLEKGEVLENLIPEAFAVVREASKRVFGMRHFDVQLLGGMVLNERCIA

100   EMRTGEGKTLTATLPAYLNALTGKGVHVVTVNDYLAQRDAENNRPLFEFL

150   GLTVGINLPGMPAPAKREAYAADITYGTNNEYGFDYLRDNMAFSPEERVQ
                                                     ***
200   RKLHYALVDEVDSILIDEARTPLIISGPAEDSSEMYKRVNKIIPHLIRQE
      *********************
250   KEDSETFQGEGHFSVDEKSRQVNLTERGLVLIEELLVKEGIMDEGESLYS

300   PANIMLMHHVTAALRAHALFTRDVDYIVKDGEVIIVDEHTGRTMQGRRWS

350   DGLHQAVEAKEGVQIQNENQTLASITFQNYFRLYEKLAGMTGTADTEAFE

400   FSSIYKLDTVVVPTNRPMIRKDLPDLVYMTEAEKIQAIIEDIKERTAKGQ

450   PVLVGTISIEKSELVSNELTKAGIKHNVLNAKFHANEAAIVAQAGYPAAV

500   TIATNMAGRGTDIVLGGSWQAEVAALENPTAEQIEKIKADWQVRHDAVLE

550   AGGLHIIGTERHESRRIDNQLRGRSGRQGDAGSSRFYLSMEDALMRIFAS

600   DRVSGMMRKLGMKPGEAIEHPWVTKAIANAQRKVESRNFDIRKQLLEYDD

650   VANDQRRAIYSQRNELLDVSDVSETINSIREDVFKATIDAYIPPQSLEEM

700   WDIPGLQERLKNDFDLDLPIAEWLDKEPELHEETLRERILAQSIEVYQRK

750   EEVVGAEMMRHFEKGVMLQTLDSLWKEHLAAMDYLRQGIHLRGYAQKDPK

800   QEYKRESFSMFAAMLESLKYEVISTLSKVQVRMPEEVEELEQQRRMEAER

850   LAQMQQLSHQDDDSAAAAALAAQTGERKVGRNDPCPCGSGKKYKQCHGRL

900   Q
```

sp|P0ACN4|ALLR_ECOLI HTH-type transcriptional repressor AllR OS=Escherichia coli (strain K12) OX=83333 GN=allR PE=1 SV=1

```
                                     *******************
0     MTEVRRRGRPGQAEPVAQKGAQALERGIAILQYLEKSGGSSSVSDISLNL
      *****
50    DLPLSTTFRLLKVLQAADFVYQDSQLGWWHIGLGVFNVGAAYIHNRDVLS

100   VAGPFMRRLMLLSGETVNVAIRNGNEAVLIGQLECKSMVRMCAPLGSRLP
```

```
150   LHASGAGKALLYPLAEEELMSIILQTGLQQFTPTTLVDMPTLLKDLEQAR

200   ELGYTVDKEEHVVGLNCIASAIYDDVGSVVAAISISGPSSRLTEDRFVSQ

250   GELVRDTARDISTALGLKAHP

sp|P33230|RCBA_ECOLI Double-strand break reduction protein OS=Escherichia
coli (strain K12) OX=83333 GN=rcbA PE=1 SV=2
                      **********************************
0     MYKITATIEKEGGTPTNWTRYSKSKLTKSECEKMLSGKKEAGVSREQKVK
      *
50    LINFNCEKLQSSRIALYSN

sp|P76069|YDAY_ECOLI Protein YdaY OS=Escherichia coli (strain K12)
OX=83333 GN=ydaY PE=1 SV=1
                           *****************************
0     MSRSSDNDQYRSRNALIRRHIEKMDASLHVGTKEFDISKVSEVDSVDDLL
      *****
50    IDNAARYLLKDWKGVGELVNGVEVALEYTAERGIALLKQNPELYWQILAE

100   AASIAQGKEQQKQDTIKKP

sp|P0C0R7|RLME_ECOLI Ribosomal RNA large subunit methyltransferase E
OS=Escherichia coli (strain K12) OX=83333 GN=rlmE PE=1 SV=1
       **********************
0     MTGKKRSASSSRWLQEHFSDKYVQQAQKKGLRSRAWFKLDEIQQSDKLFK

50    PGMTVVDLGAAPGGWSQYVVTQIGGKGRIIACDLLPMDPIVGVDFLQGDF

100   RDELVMKALLERVGDSKVQVVMSDMAPNMSGTPAVDIPRAMYLVELALEM

150   CRDVLAPGGSFVVKVFQGEGFDEYLREIRSLFTKVKVRKPDSSRARSREV

200   YIVATGRKP

sp|P0AG67|RS1_ECOLI 30S ribosomal protein S1 OS=Escherichia coli (strain
K12) OX=83333 GN=rpsA PE=1 SV=1

0     MTESFAQLFEESLKEIETRPGSIVRGVVVAIDKDVVLVDAGLKSESAIPA

50    EQFKNAQGELEIQVGDEVDVALDAVEDGFGETLLSREKAKRHEAWITLEK

100   AYEDAETVTGVINGKVKGGFTVELNGIRAFLPGSLVDVRPVRDTLHLEGK

150   ELEFKVIKLDQKRNNVVVSRRAVIESENSAERDQLLENLQEGMEVKGIVK

200   NLTDYGAFVDLGGVDGLLHITDMAWKRVKHPSEIVNVGDEITVKVLKFDR

250   ERTRVSLGLKQLGEDPWVAIAKRYPEGTKLTGRVTNLTDYGCFVEIEEGV

300   EGLVHVSEMDWTNKNIHPSKVVNVGDVVEVMVLDIDEERRRISLGLKQCK

350   ANPWQQFAETHNKGDRVEGKIKSITDFGIFIGLDGGIDGLVHLSDISWNV
```

400   AGEEAVREYKKGDEIAAVVLQVDAERERISLGVKQLAEDPFNNWVALNKK

450   GAIVTGKVTAVDAKGATVELADGVEGYLRASEASRDRVEDATLVLSVGDE
              ****
500   VEAKFTGVDRKNRAISLSVRAKDEADEKDAIATVNKQEDANFSNNAMAEA

550   FKAAKGE

sp|P28916|YBFD_ECOLI H repeat-associated putative transposase YbfD OS=Escherichia coli (strain K12) OX=83333 GN=ybfD PE=3 SV=1

0   MELKKLMEHISIIPDYRQAWKVEHKLSDILLLTICAVISGAEGWEDIEDF
                                        ****
50   GETHPDFLKQYGDFENGIPVHDTIARVVSCICPAKFHESFINWMLDYHSS
      ****
100   DDKDVIAIDGKIHRHSYDKSRRKGAIHVISAFSTMHSLVIGQIKTDKKSN

150   EITAIPELLNMLDIKGKIIKTDAMGCQKDIAEKIQKQGGDYLFAVKGNQG

200   RLNKAFEEKFPLKELNNPKHDSYAISEKSHGREETRLHIVCDVPDELIDF

250   TFE

sp|P32721|ALSA_ECOLI D-allose import ATP-binding protein AlsA OS=Escherichia coli (strain K12) OX=83333 GN=alsA PE=3 SV=2

0   MATPYISMAGIGKSFGPVHALKSVNLTVYPGEIHALLGENGAGKSTLMKV

50   LSGIHEPTKGTITINNISYNKLDHKLAAQLGIGIIYQELSVIDELTVLEN

100   LYIGRHLTKKICGVNIIDWREMRVRAAMMLLRVGLKVDLDEKVANLSISH

150   KQMLEIAKTLMLDAKVIIMDEPTSSLTNKEVDYLFLIMNQLRKEGTAIVY
                      ****
200   ISHKLAEIRRICDRYTVMKDGSSVCSGIVSDVSNDDIVRLMVGRELQNRF

250   NAMKENVSNLAHETVFEVRNVTSRDRKKVRDISFSVCRGEILGFAGLVGS

300   GRTELMNCLFGVDKRAGGEIRLNGKDISPRSPLDAVKKGMAYITESRRDN

350   GFFPNFSIAQNMAISRSLKDGGYKGAMGLFHEVDEQRTAENQRELLALKC

400   HSVNQNITELSGGNQQKVLISKWLCCCPEVIIFDEPTRGIDVGAKAEIYK

450   VMRQLADDGKVILMVSSELPEIITVCDRIAVFCEGRLTQILTNRDDMSEE

500   EIMAWALPQE

sp|P28629|ADIA_ECOLI Biodegradative arginine decarboxylase OS=Escherichia coli (strain K12) OX=83333 GN=adiA PE=1 SV=1

0   MKVLIVESEFLHQDTWVGNAVERLADALSQQNVTVIKSTSFDDGFAILSS

```
50    NEAIDCLMFSYQMEHPDEHQNVRQLIGKLHERQQNVPVFLLGDREKALAA

100   MDRDLLELVDEFAWILEDTADFIAGRAVAAMTRYRQQLLPPLFSALMKYS

150   DIHEYSWAAPGHQGGVGFTKTPAGRFYHDYYGENLFRTDMGIERTSLGSL

200   LDHTGAFGESEKYAARVFGADRSWSVVVGTSGSNRTIMQACMTDNDVVVV
                                                ************
250   DRNCHKSIEQGLMLTGAKPVYMVPSRNRYGIIGPIYPQEMQPETLQKKIS
      ****************
300   ESPLTKDKAGQKPSYCVVTNCTYDGVCYNAKEAQDLLEKTSDRLHFDEAW

350   YGYARFNPIYADHYAMRGEPGDHNGPTVFATHSTHKLLNALSQASYIHVR

400   EGRGAINFSRFNQAYMMHATTSPLYAICASNDVAVSMMDGNSGLSLTQEV

450   IDEAVDFRQAMARLYKEFTADGSWFFKPWNKEVVTDPQTGKTYDFADAPT

500   KLLTTVQDCWVMHPGESWHGFKDIPDNWSMLDPIKVSILAPGMGEDGELE

550   ETGVPAALVTAWLGRHGIVPTRTTDFQIMFLFSMGVTRGKWGTLVNTLCS

600   FKRHYDANTPLAQVMPELVEQYPDTYANMGIHDLGDTMFAWLKENNPGAR

650   LNEAYSGLPVAEVTPREAYNAIVDNNVELVSIENLPGRIAANSVIPYPPG

700   IPMLLSGENFGDKNSPQVSYLRSLQSWDHHFPGFEHETEGTEIIDGIYHV

750   MCVKA
```

sp|P0A8K1|PSD_ECOLI Phosphatidylserine decarboxylase proenzyme OS=Escherichia coli (strain K12) OX=83333 GN=psd PE=1 SV=1

```
0     MLNSFKLSLQYILPKLWLTRLAGWGASKRAGWLTKLVIDLFVKYYKVDMK

50    EAQKPDTASYRTFNEFFVRPLRDEVRPIDTDPNVLVMPADGVISQLGKIE

100   EDKILQAKGHNYSLEALLAGNYLMADLFRNGTFVTTYLSPRDYHRVHMPC

150   NGILREMIYVPGDLFSVNHLTAQNVPNLFARNERVICLFDTEFGPMAQIL

200   VGATIVGSIETVWAGTITPPREGIIKRWTWPAGENDGSVALLKGQEMGRF
                                                         *
250   KLGSTVINLFAPGKVNLVEQLESLSVTKIGQPLAVSTETFVTPDAEPAPL
      **********************
300   PAEEIEAEHDASPLVDDKKDQV
```

sp|P0AEC3|ARCB_ECOLI Aerobic respiration control sensor protein ArcB OS=Escherichia coli (strain K12) OX=83333 GN=arcB PE=1 SV=1

```
0     MKQIRLLAQYYVDLMMKLGLVRFSMLLALALVVLAIVVQMAVTMVLHGQV

50    ESIDVIRSIFFGLLITPWAVYFLSVVVEQLEESRQRLSRLVQKLEEMRER
```

100   DLSLNVQLKDNIAQLNQEIAVREKAEAELQETFGQLKIEIKEREETQIQL

150   EQQSSFLRSFLDASPDLVFYRNEDKEFSGCNRAMELLTGKSEKQLVHLKP

200   ADVYSPEAAAKVIETDEKVFRHNVSLTYEQWLDYPDGRKACFEIRKVPYY

250   DRVGKRHGLMGFGRDITERKRYQDALERASRDKTTFISTISHELRTPLNG

300   IVGLSRILLDTELTAEQEKYLKTIHVSAVTLGNIFNDIIDMDKMERRKVQ

350   LDNQPVDFTSFLADLENLSALQAQQKGLRFNLEPTLPLPHQVITDGTRLR

400   QILWNLISNAVKFTQQGQVTVRVRYDEGDMLHFEVEDSGIGIPQDELDKI

450   FAMYYQVKDSHGGKPATGTGIGLAVSRRLAKNMGGDITVTSEQGKGSTFT

500   LTIHAPSVAEEVDDAFDEDDMPLPALNVLLVEDIELNVIVARSVLEKLGN

550   SVDVAMTGKAALEMFKPGEYDLVLLDIQLPDMTGLDISRELTKRYPREDL
                                                   ****
600   PPLVALTANVLKDKQEYLNAGMDDVLSKPLSVPALTAMIKKFWDTQDDEE
      ****
650   STVTTEENSKSEALLDIPMLEQYLELVGPKLITDGLAVFEKMMPGYVSVL
                                                          ****
700   ESNLTAQDKKGIVEEGHKIKGAAGSVGLRHLQQLGQQIQSPDLPAWEDNV
      ****
750   GEWIEEMKEEWRHDVEVLKAWVAKATKK

sp|P37759|RMLB1_ECOLI dTDP-glucose 4,6-dehydratase 1 OS=Escherichia coli (strain K12) OX=83333 GN=rfbB PE=3 SV=2

0   MKILVTGGAGFIGSAVVRHIINNTQDSVVNVDKLTYAGNRESLADVSDSE

50   RYVFEHADICDAPAMARIFAQHQPDAVMHLAAESHVDRSITGPAAFIETN
                     ****
100   IVGTYVLLEAARNYWSALDSDKKNSFRFHHISTDEVYGDLPHPDEVNNTE

150   ELPLFTETTAYAPSSPYSASKASSDHLVRAWKRTYGLPTIVTNCSNNYGP

200   YHFPEKLIPLVILNALEGKALPIYGKGDQIRDWLYVEDHARALYTVVTEG

250   KAGETYNIGGHNEKKNIDVVLTICDLLDEIVPKEKSYREQITYVADRPGH

300   DRRYAIDAEKIGRALGWKPQETFESGIRKTVEWYLSNTKWVDNVKSGAYQ

350   SWIEQNYEGRQ

sp|P0ABB4|ATPB_ECOLI ATP synthase subunit beta OS=Escherichia coli (strain K12) OX=83333 GN=atpD PE=1 SV=2

0   MATGKIVQVIGAVVDVEFPQDAVPRVYDALEVQNGNERLVLEVQQQLGGG

50   IVRTIAMGSSDGLRRGLDVKDLEHPIEVPVGKATLGRIMNVLGEPVDMKG

```
100   EIGEEERWAIHRAAPSYEELSNSQELLETGIKVIDLMCPFAKGGKVGLFG

150   GAGVGKTVNMMELIRNIAIEHSGYSVFAGVGERTREGNDFYHEMTDSNVI

200   DKVSLVYGQMNEPPGNRLRVALTGLTMAEKFRDEGRDVLLFVDNIYRYTL

250   AGTEVSALLGRMPSAVGYQPTLAEEMGVLQERITSTKTGSITSVQAVYVP

300   ADDLTDPSPATTFAHLDATVVLSRQIASLGIYPAVDPLDSTSRQLDPLVV
                        *********************
350   GQEHYDTARGVQSILQRYQELKDIIAILGMDELSEEDKLVVARARKIQRF

400   LSQPFFVAEVFTGSPGKYVSLKDTIRGFKGIMEGEYDHLPEQAFYMVGSI

450   EEAVEKAKKL
```

sp|P06149|DLD_ECOLI Quinone-dependent D-lactate dehydrogenase
OS=Escherichia coli (strain K12) OX=83333 GN=dld PE=1 SV=3

```
0     MSSMTTTDNKAFLNELARLVGSSHLLTDPAKTARYRKGFRSGQGDALAVV

50    FPGSLLELWRVLKACVTADKIILMQAANTGLTEGSTPNGNDYDRDVVIIS

100   TLRLDKLHVLGKGEQVLAYPGTTLYSLEKALKPLGREPHSVIGSSCIGAS
                                                 *******
150   VIGGICNNSGGSLVQRGPAYTEMSLFARINEDGKLTLVNHLGIDLGETPE
      ***********************
200   QILSKLDDDRIKDDDVRHDGRHAHDYDYVHRVRDIEADTPARYNADPDRL

250   FESSGCAGKLAVFAVRLDTFEAEKNQQVFYIGTNQPEVLTEIRRHILANF

300   ENLPVAGEYMHRDIYDIAEKYGKDTFLMIDKLGTDKMPFFFNLKGRTDAM

350   LEKVKFFRPHFTDRAMQKFGHLFPSHLPPRMKNWRDKYEHHLLLKMAGDG

400   VGEAKSWLVDYFKQAEGDFFVCTPEEGSKAFLHRFAAAGAAIRYQAVHSD

450   EVEDILALDIALRRNDTEWYEHLPPEIDSQLVHKLYYGHFMCYVFHQDYI

500   VKKGVDVHALKEQMLELLQQRGAQYPAEHNVGHLYKAPETLQKFYRENDP

550   TNSMNPGIGKTSKRKNWQEVE
```

sp|P37642|YHJD_ECOLI Inner membrane protein YhjD OS=Escherichia coli
(strain K12) OX=83333 GN=yhjD PE=1 SV=1

```
                ************************** 
0     MTQENEIKRPIQDLEHEPIKPLDNSEKGSKVSQALETVTTTAEKVQRQPV

50    IAHLIRATERFNDRLGNQFGAAITYFSFLSMIPILMVSFAAGGFVLASHP

100   MLLQDIFDKILQNISDPTLAATLKNTINTAVQQRTTVGLVGLAVALYSGI

150   NWMGNLREAIRAQSRDVWERSPQDQEKFWVKYLRDFISLIGLLIALIVTL
```

200   SITSVAGSAQQMIISALHLNSIEWLKPTWRLIGLAISIFANYLLFFWIFW

250   RLPRHRPRKKALIRGTFLAAIGFEVIKIVMTYTLPSLMKSPSGAAFGSVL

300   GLMAFFYFFARLTLFCAAWIATAEYKDDPRMPGKTQP

sp|P38394|YDAE_ECOLI Uncharacterized protein YdaE OS=Escherichia coli (strain K12) OX=83333 GN=ydaE PE=4 SV=1

```
        ***********************       ********************
0       MTKKIKCAYHLCKKDVEESKAIERMLHFMHGILSKDEPRKYCSEACAEKD
        ***
50      QMAHEL
```

sp|P37197|YHJA_ECOLI Probable cytochrome c peroxidase OS=Escherichia coli (strain K12) OX=83333 GN=yhjA PE=4 SV=1

```
0       MKMVSRITAIGLAGVAICYLGLSGYVWYHDNKRSKQADVQASAVSENNKV

50      LGFLREKGCDYCHTPSAELPAYYYIPGAKQLMDYDIKLGYKSFNLEAVRA

100     ALLADKPVSQSDLNKIEWVMQYETMPPTRYTALHWAGKVSDEERAEILAW

150     IAKQRAEYYASNDTAPEHRNEPVQPIPQKLPTDAQKVALGFALYHDPRLS

200     ADSTISCAHCHALNAGGVDGRKTSIGVGGAVGPINAPTVFNSVFNVEQFW
                        **************************
250     DGRAATLQDQAGGPPLNPIEMASKSWDEIIAKLEKDPQLKTQFLEVYPQG

300     FSGENITDAIAEFEKTLITPDSPFDKWLRGDENALTAQQKKGYQLFKDNK

350     CATCHGGIILGGRSFEPLGLKKDFNFGEITAADIGRMNVTKEERDKLRQK

400     VPGLRNVALTAPYFHRGDVPTLDGAVKLMLRYQVGKELPQEDVDDIVAFL

450     HSLNGVYTPYMQDKQ
```

sp|P77726|YAJR_ECOLI Inner membrane transport protein YajR OS=Escherichia coli (strain K12) OX=83333 GN=yajR PE=1 SV=2

```
0       MNDYKMTPGERRATWGLGTVFSLRMLGMFMVLPVLTTYGMALQGASEALI

50      GIAIGIYGLTQAVFQIPFGLLSDRIGRKPLIVGGLAVFAAGSVIAALSDS

100     IWGIILGRALQGSGAIAAAVMALLSDLTREQNRTKAMAFIGVSFGITFAI

150     AMVLGPIITHKLGLHALFWMIAILATTGIALTIWVVPNSSTHVLNRESGM

200     VKGSFSKVLAEPRLLKLNFGIMCLHILLMSTFVALPGQLADAGFPAAEHW

250     KVYLATMLIAFGSVVPFIIYAEVKRKMKQVFVFCVGLIVVAEIVLWNAQT

300     QFWQLVVGVQLFFVAFNLMEALLPSLISKESPAGYKGTAMGVYSTSQFLG

350     VAIGGSLGGWINGMFDGQGVFLAGAMLAAVWLTVASTMKEPPYVSSLRIE
```

```
                          *********************
400   IPANIAANEALKVRLLETEGIKEVLIAEEEHSAYVKIDSKVTNRFEIEQA

450   IRQA
```

sp|P77529|YDJN_ECOLI L-cystine transporter YdjN OS=Escherichia coli (strain K12) OX=83333 GN=ydjN PE=3 SV=1

```
0     MNFPLIANIVVFVVLLFALAQTRHKQWSLAKKVLVGLVMGVVFGLALHTI

50    YGSDSQVLKDSVQWFNIVGNGYVQLLQMIVMPLVFASILSAVARLHNASQ

100   LGKISFLTIGTLLFTTLIAALVGVLVTNLFGLTAEGLVQGGAETARLNAI

150   ESNYVGKVSDLSVPQLVLSFIPKNPFADLTGANPTSIISVVIFAAFLGVA

200   ALKLLKDDAPKGERVLAAIDTLQSWVMKLVRLVMQLTPYGVLALMTKVVA

250   GSNLQDIIKLGSFVVASYLGLLIMFAVHGILLGINGVSPLKYFRKVWPVL

300   TFAFTSRSSAASIPLNVEAQTRRLGVPESIASFAASFGATIGQNGCAGLY

350   PAMLAVMVAPTVGINPLDPMWIATLVGIVTVSSAGVAGVGGGATFAALIV
                                                 ***********
400   LPAMGLPVTLVALLISVEPLIDMGRTALNVSGSMTAGTLTSQWLKQTDKA
      **********
450   ILDSEDDAELAHH
```

sp|P77359|DJLC_ECOLI Uncharacterized J domain-containing protein DjlC OS=Escherichia coli (strain K12) OX=83333 GN=djlC PE=4 SV=1

```
                                                    ****
0     MKTCWQILEIESTTQIDIIRQAYLARLPLCHPETDPQGFKALRQAYEEAL
      ***************************
50    RLAVNPVEEADDEEKDAAAEHEILRAFRTLLDSESDRFQPSAWQKFIQQL

100   NTWNMEDVDQLRWPLCAIAIEARYLSLNCASLLAERLNWHSFNDSEGMDE

150   EEREAFLEAIQAGDCFDFLSLLEYPIALQNQTVEYYFALERCCRYHPDYV

200   TAFLAMEGPWLIPDDAKLHRKLLRWYSSVQTGMAELIPVAQQWQTEEPES

250   EDARYYLCAQRLYCGEGESLLADLCAYWESYPSTQADNLLLQWSKRHCPD

300   YFALLVMVIEARSMVDAQGQPLKYVPGESARTRLLWAEILHSGKLSPLGQ

350   SFIESLFFKRKAWAWWKSRVGSETEQDSPFLDLYRVAEQVVLEAFPKQEM
                             ***************************
400   LARLNTRLEGGDAHPLEAIVTRMLLTKVKLEPEDEDVDEPTPENHEEKND
      ****************
450   EGEKPQSITSIIKISLTVLVIGYALGKIAMLFS
```

sp|P21177|FADB_ECOLI Fatty acid oxidation complex subunit alpha OS=Escherichia coli (strain K12) OX=83333 GN=fadB PE=1 SV=2

```
0     MLYKGDTLYLDWLEDGIAELVFDAPGSVNKLDTATVASLGEAIGVLEQQS

50    DLKGLLLRSNKAAFIVGADITEFLSLFLVPEEQLSQWLHFANSVFNRLED

100   LPVPTIAAVNGYALGGGCECVLATDYRLATPDLRIGLPETKLGIMPGFGG

150   SVRMPRMLGADSALEIIAAGKDVGADQALKIGLVDGVVKAEKLVEGAKAV

200   LRQAINGDLDWKAKRQPKLEPLKLSKIEATMSFTIAKGMVAQTAGKHYPA

250   PITAVKTIEAAARFGREEALNLENKSFVPLAHTNEARALVGIFLNDQYVK

300   GKAKKLTKDVETPKQAAVLGAGIMGGGIAYQSAWKGVPVVMKDINDKSLT

350   LGMTEAAKLLNKQLERGKIDGLKLAGVISTIHPTLDYAGFDRVDIVVEAV

400   VENPKVKKAVLAETEQKVRQDTVLASNTSTIPISELANALERPENFCGMH
                      ********************
450   FFNPVHRMPLVEIIRGEKSSDETIAKVVAWASKMGKTPIVVNDCPGFFVN

500   RVLFPYFAGFSQLLRDGADFRKIDKVMEKQFGWPMGPAYLLDVVGIDTAH
                                       *****************
550   HAQAVMAAGFPQRMQKDYRDAIDALFDANRFGQKNGLGFWRYKEDSKGKP
      ****************************
600   KKEEDAAVEDLLAEVSQPKRDFSEEEIIARMMIPMVNEVVRCLEEGIIAT

650   PAEADMALVYGLGFPPFHGGAFRWLDTLGSAKYLDMAQQYQHLGPLYEVP

700   EGLRNKARHNEPYYPPVEPARPVGDLKTA
```

sp|P0AGF4|XYLE_ECOLI D-xylose-proton symporter OS=Escherichia coli (strain K12) OX=83333 GN=xylE PE=1 SV=1

```
0     MNTQYNSSYIFSITLVATLGGLLFGYDTAVISGTVESLNTVFVAPQNLSE

50    SAANSLLGFCVASALIGCIIGGALGGYCSNRFGRRDSLKIAAVLFFISGV

100   GSAWPELGFTSINPDNTVPVYLAGYVPEFVIYRIIGGIGVGLASMLSPMY

150   IAELAPAHIRGKLVSFNQFAIIFGQLLVYCVNYFIARSGDASWLNTDGWR

200   YMFASECIPALLFLMLLYTVPESPRWLMSRGKQEQAEGILRKIMGNTLAT

250   QAVQEIKHSLDHGRKTGGRLLMFGVGVIVIGVMLSIFQQFVGINVVLYYA

300   PEVFKTLGASTDIALLQTIIVGVINLTFTVLAIMTVDKFGRKPLQIIGAL

350   GMAIGMFSLGTAFYTQAPGIVALLSMLFYVAAFAMSWGPVCWVLLSEIFP

400   NAIRGKALAIAVAAQWLANYFVSWTFPMMDKNSWLVAHFHNGFSYWIYGC
                    ********************
450   MGVLAALFMWKFVPETKGKTLEELEALWEPETKKTQQTATL
```

sp|P07001|PNTA_ECOLI NAD(P) transhydrogenase subunit alpha OS=Escherichia coli (strain K12) OX=83333 GN=pntA PE=1 SV=2

```
0     MRIGIPRERLTNETRVAATPKTVEQLLKLGFTVAVESGAGQLASFDDKAF

50    VQAGAEIVEGNSVWQSEIILKVNAPLDDEIALLNPGTTLVSFIWPAQNPE

100   LMQKLAERNVTVMAMDSVPRISRAQSLDALSSMANIAGYRAIVEAAHEFG

150   RFFTGQITAAGKVPPAKVMVIGAGVAGLAAIGAANSLGAIVRAFDTRPEV
                  ********************
200   KEQVQSMGAEFLELDFKEEAGSGDGYAKVMSDAFIKAEMELFAAQAKEVD

250   IIVTTALIPGKPAPKLITREMVDSMKAGSVIVDLAAQNGGNCEYTVPGEI

300   FTTENGVKVIGYTDLPGRLPTQSSQLYGTNLVNLLKLLCKEKDGNITVDF

350   DDVVIRGVTVIRAGEITWPAPPIQVSAQPQAAQKAAPEVKTEEKCTCSPW

400   RKYALMALAIILFGWMASVAPKEFLGHFTVFALACVVGYYVVWNVSHALH

450   TPLMSVTNAISGIIVVGALLQIGQGGWVSFLSFIAVLIASINIFGGFTVT

500   QRMLKMFRKN
```

sp|P0A6L0|DEOC_ECOLI Deoxyribose-phosphate aldolase OS=Escherichia coli (strain K12) OX=83333 GN=deoC PE=1 SV=1

```
0     MTDLKASSLRALKLMDLTTLNDDDTDEKVIALCHQAKTPVGNTAAICIYP

50    RFIPIARKTLKEQGTPEIRIATVTNFPHGNDDIDIALAETRAAIAYGADE
                                              **********
100   VDVVFPYRALMAGNEQVGFDLVKACKEACAAANVLLKVIIETGELKDEAL
      **********
150   IRKASEISIKAGADFIKTSTGKVAVNATPESARIMMEVIRDMGVEKTVGF

200   KPAGGVRTAEDAQKYLAIADELFGADWADARHYRFGASSLLASLLKALGH

250   GDGKSASSY
```

sp|P77288|YFCV_ECOLI Uncharacterized fimbrial-like protein YfcV OS=Escherichia coli (strain K12) OX=83333 GN=yfcV PE=2 SV=1

```
                                            ************
0     MSKFVKTAIAAAMVMGAFTSTATIAAGNNGTARFYGTIEDSVCSIVPDDH
      ********
50    KLEVDMGDIGAEKLKNNGTTTPKNFQIRLQDCVFDTQETMTTTFTGTVSS

100   ANSGNYYTIFNTDTGAAFNNVSLAIGDSLGTSYKSGMGIDQKIVKDTATN

150   KGKAKQTLNFKAWLVGAADAPDLGNFEANTTFQITYL
```

sp|P09169|OMPT_ECOLI Protease 7 OS=Escherichia coli (strain K12) OX=83333 GN=ompT PE=1 SV=1

```
0     MRAKLLGIVLTTPIAISSFASTETLSFTPDNINADISLGTLSGKTKERVY

50    LAEEGGRKVSQLDWKFNNAAIIKGAINWDLMPQISIGAAGWTTLGSRGGN

100   MVDQDWMDSSNPGTWTDESRHPDTQLNYANEFDLNIKGWLLNEPNYRLGL

150   MAGYQESRYSFTARGGSYIYSSEEGFRDDIGSFPNGERAIGYKQRFKMPY
                       ***************************
200   IGLTGSYRYEDFELGGTFKYSGWVESSDNDEHYDPGKRITYRSKVKDQNY

250   YSVAVNAGYYVTPNAKVYVEGAWNRVTNKKGNTSLYDHNNNTSDYSKNGA

300   GIENYNFITTAGLKYTF
```

sp|P0ACG1|STPA_ECOLI DNA-binding protein StpA OS=Escherichia coli (strain K12) OX=83333 GN=stpA PE=1 SV=1

```
                               *************************
0     MSVMLQSLNNIRTLRAMAREFSIDVLEEMLEKFRVVTKERREEEEQQQRE
      *******
50    LAERQEKISTWLELMKADGINPEELLGNSSAAAPRAGKKRQPRPAKYKFT

100   DVNGETKTWTGQGRTPKPIAQALAEGKSLDDFLI
```

sp|P77499|SUFC_ECOLI Probable ATP-dependent transporter SufC OS=Escherichia coli (strain K12) OX=83333 GN=sufC PE=1 SV=1

```
0     MLSIKDLHVSVEDKAILRGLSLDVHPGEVHAIMGPNGSGKSTLSATLAGR

50    EDYEVTGGTVEFKGKDLLALSPEDRAGEGIFMAFQYPVEIPGVSNQFFLQ

100   TALNAVRSYRGQETLDRFDFQDLMEEKIALLKMPEDLLTRSVNVGFSGGE
                  ********************
150   KKRNDILQMAVLEPELCILDESDSGLDIDALKVVADGVNSLRDGKRSFII

200   VTHYQRILDYIKPDYVHVLYQGRIVKSGDFTLVKQLEEQGYGWLTEQQ
```

sp|P0AFM4|PSIF_ECOLI Phosphate starvation-inducible protein PsiF OS=Escherichia coli (strain K12) OX=83333 GN=psiF PE=1 SV=1

```
                                                     *
0     MKITLLVTLLFGLVFLTTVGAAERTLTPQQQRMTSCNQQATAQALKGDAR
      ********************
50    KTYMSDCLKNSKSAPGEKSLTPQQQKMRECNNQATQQSLKGDDRNKFMSA

100   CLKKAA
```

sp|P11557|DAMX_ECOLI Cell division protein DamX OS=Escherichia coli (strain K12) OX=83333 GN=damX PE=1 SV=2

```
      ******************************
0     MDEFKPEDELKPDPSDRRTGRSRQSSERSERTERGEPQINFDDIELDDTD
         *****************************************
50    DRRPTRAQKERNEEPEIEEEIDESEDETVDEERVERRPRKRKKAASKPAS
                           ***********************
100   RQYMMMGVGILVLLLLIIGIGSALKAPSTTSSDQTASGEKSIDLAGNATD
```

150   QANGVQPAPGTTSAENTQQDVSLPPISSTPTQGQTPVATDGQQRVEVQGD

200   LNNALTQPQNQQQLNNVAVNSTLPTEPATVAPVRNGNASRDTAKTQTAER

250   PSTTRPARQQAVIEPKKPQATVKTEPKPVAQTPKRTEPAAPVASTKAPAA

300   TSTPAPKETATTAPVQTASPAQTTATPAAGAKTAGNVGSLKSAPSSHYTL

350   QLSSSSNYDNLNGWAKKENLKNYVVYETTRNGQPWYVLVSGVYASKEEAK

400   KAVSTLPADVQAKNPWAKPLRQVQADLK

sp|P0A9L8|P5CR_ECOLI Pyrroline-5-carboxylate reductase OS=Escherichia coli (strain K12) OX=83333 GN=proC PE=1 SV=1

0   MEKKIGFIGCGNMGKAILGGLIASGQVLPGQIWVYTPSPDKVAALHDQFG

50   INAAESAQEVAQIADIIFAAVKPGIMIKVLSEITSSLNKDSLVVSIAAGV

100   TLDQLARALGHDRKIIRAMPNTPALVNAGMTSVTPNALVTPEDTADVLNI

150   FRCFGEAEVIAEPMIHPVVGVSGSSPAYVFMFIEAMADAAVLGGMPRAQA
                                                       *
200   YKFAAQAVMGSAKMVLETGEHPGALKDMVCSPGGTTIEAVRVLEEKGFRA
      ****
250   AVIEAMTKCMEKSEKLSKS

sp|P31058|YADC_ECOLI Uncharacterized fimbrial-like protein YadC OS=Escherichia coli (strain K12) OX=83333 GN=yadC PE=2 SV=2

0   MKTIFRYILFLALYSCCNTVSAYTSFIVGNNAGVDNYRGPSTAAQMTFNY

50   TSTASNLVFYKPTQLGPTGVKMYWSYLDTGTGGGILYCNTSGRANPGPIT

100   IENAMVYSGKDYGGHKLFNTSVPGLYYTMLISRVWSAYDTITDIQSPGIY
                 ****
150   IGDPSNQEFFFSVTDSDLQTKGCNKADDYDKFWAIGGIVHNITVEFYTDT

200   NFDPTLNQQVQLSSSSNYLYSFKAYSPGTKVVDHSNHIYVNFTLNNVKLT

250   LPTCFTSILTGPSVNGSTVRMGEYSSGTIKNGASPVPFDISLQNCIRVRN
                                                      **
300   IETKLVTGKVGTQNTQLLGNTLTGSTAAKGVGVLIEGLATSKNPLMTLKP
      ****
350   NDTNSVYIDYETEDDTSDGVYPNQGNGTSQPLHFQATLKQDGNIAIEPGE

400   FKATSTFQVTYP

sp|P65807|YGEY_ECOLI Uncharacterized protein YgeY OS=Escherichia coli (strain K12) OX=83333 GN=ygeY PE=3 SV=1
                                    ****
0   MAKNIPFKLILEKAKDYQADMTRFLRDMVAIPSESCDEKRVVHRIKEEME
      ******
50   KVGFDKVEIDPMGNVLGYIGHGPRLVAMDAHIDTVGIGNIKNWDFDPYEG

```
100   METDELIGGRGTSDQEGGMASMVYAGKIIKDLGLEDEYTLLVTGTVQEED

150   CDGLCWQYIIEQSGIRPEFVVSTEPTDCQVYRGQRGRMEIRIDVQGVSCH

200   GSAPERGDNAIFKMGPILGELQELSQRLGYDEFLGKGTLTVSEIFFTSPS

250   RCAVADSCAVSIDRRLTWGETWEGALDEIRALPAVQKANAVVSMYNYDRP

300   SWTGLVYPTECYFPTWKVEEDHFTVKALVNAYEGLFGKAPVVDKWTFSTN

350   GVSIMGRHGIPVIGFGPGKEPEAHAPNEKTWKSHLVTCAAMYAAIPLSWL

400   ATE
```

sp|P31469|CBRC_ECOLI UPF0167 protein CbrC OS=Escherichia coli (strain K12) OX=83333 GN=cbrC PE=2 SV=1

```
0     MTQNIRPLPQFKYHPKPLETGAFEQDKTVECDCCEQQTSVYYSGPFYCVD
                                ************************
50    EVEHLCPWCIADGSAAEKFAGSFQDDASIEGVEFEYDEEDEFAGIKNTYP
      ******
100   DEMLKELVERTPGYHGWQQEFWLAHCGDFCVFIGYVGWNDIKDRLDEFAN

150   LEEDCENFGIRNSDLAKCLQKGGHCQGYLFRCLHCGKLRLWGDFS
```

sp|P0AGD7|SRP54_ECOLI Signal recognition particle protein OS=Escherichia coli (strain K12) OX=83333 GN=ffh PE=1 SV=1

```
0     MFDNLTDRLSRTLRNISGRGRLTEDNVKDTLREVRMALLEADVALPVVRE

50    FINRVKEKAVGHEVNKSLTPGQEFVKIVRNELVAAMGEENQTLNLAAQPP

100   AVVLMAGLQGAGKTTSVGKLGKFLREKHKKKVLVVSADVYRPAAIKQLET

150   LAEQVGVDFFPSDVGQKPVDIVNAALKEAKLKFYDVLLVDTAGRLHVDEA

200   MMDEIKQVHASINPVETLFVVDAMTGQDAANTAKAFNEALPLTGVVLTKV
                                                      **
250   DGDARGGAALSIRHITGKPIKFLGVGEKTEALEPFHPDRIASRILGMGDV
      ************************************
300   LSLIEDIESKVDRAQAEKLASKLKKGDGFDLNDFLEQLRQMKNMGGMASL

350   MGKLPGMGQIPDNVKSQMDDKVLVRMEAIINSMTMKERAKPEIIKGSRKR

400   RIAAGCGMQVQDVNRLLKQFDDMQRMMKKMKKGGMAKMMRSMKGMMPPGF

450   PGR
```

sp|P0AE45|YTFL_ECOLI UPF0053 inner membrane protein YtfL OS=Escherichia coli (strain K12) OX=83333 GN=ytfL PE=1 SV=1

```
0     MLNSILVILCLIAVSAFFSMSEISLAASRKIKLKLLADEGNINAQRVLNM
```

50 QENPGMFFTVVQIGLNAVAILGGIVGDAAFSPAFHSLFSRYMSAELSEQL

100 SFILSFSLVTGMFILFADLTPKRIGMIAPEAVALRIINPMRFCLYVCTPL

150 VWFFNGLANIIFRIFKLPMVRKDDITSDDIYAVVEAGALAGVLRKQEHEL
****
200 IENVFELESRTVPSSMTPRENVIWFDLHEDEQSLKNKVAEHPHSKFLVCN

250 EDIDHIIGYVDSKDLLNRVLANQSLALNSGVQIRNTLIVPDTLTLSEALE

300 SFKTAGEDFAVIMNEYALVVGIITLNDVMTTLMGDLVGQGLEEQIVARDE

350 NSWLIDGGTPIDDVMRVLDIDEFPQSGNYETIGGFMMFMLRKIPKRTDSV
****
400 KFAGYKFEVVDIDNYRIDQLLVTRIDSKATALSPKLPDAKDKEESVA

sp|P0CF68|INSE3_ECOLI Transposase InsE for insertion sequence IS3C OS=Escherichia coli (strain K12) OX=83333 GN=insE3 PE=3 SV=1
****
0 MTKTVSTSKKPRKQHSPEFRSEALKLAERIGVTAAARELSLYESQLYNWR

50 SKQQNQQTSSERELEMSTEIARLKRQLAERDEELAILQKAATYFAKRLK

sp|P28917|YDCC_ECOLI H repeat-associated putative transposase YdcC OS=Escherichia coli (strain K12) OX=83333 GN=ydcC PE=3 SV=1

0 MELKKLMGHISIIPDYRQAWKMEHKLSDILLLTICAVISGAEGWEDIEDF
**
50 GETHPDFLKQYGDFENGIPVHDTIARVVSCISPAKFHECFINWMRDCHSS
****
100 DDKDVIAIDGKTLRHSYDKSRRRGAIHVISAFSTMHSLVIGQIKTDEKSN

150 EITAIPELLNMLDIKGKIITTDAMGCQKDIAEKIQKQGGDYLFAVKGNQG

200 RLNKAFEEKFPLKELNNPAHDSYAMSEKSHGREEIRLHIVCDVPDELIDF

250 TFEWKGLKKLCVAVSFRSIIAEQKKELEMTVRYYISSADLTAEKFATAIR

300 NHWHVENKLHWRLDVVMNEDDCKIRRGNAAELFSGIRHIAINILTNDKVF

350 KAGLRRKMRKAAMDRNYLASVLTGSGLS

sp|P77610|ANSP_ECOLI L-asparagine permease OS=Escherichia coli (strain K12) OX=83333 GN=ansP PE=3 SV=2

0 MSKHDTDTSDQHAAKRRWLNAHEEGYHKAMGNRQVQMIAIGGAIGTGLFL

50 GAGARLQMAGPALALVYLICGLFSFFILRALGELVLHRPSSGSFVSYARE

100 FLGEKAAYVAGWMYFINWAMTGIVDITAVALYMHYWGAFGGVPQWVFALA

150 ALTIVGTMNMIGVKWFAEMEFWFALIKVLAIVTFLVVGTVFLGSGQPLDG

200 NTTGFHLITDNGGFFPHGLLPALVLIQGVVFAFASIEMVGTAAGECKDPQ

```
250   TMVPKAINSVIWRIGLFYVGSVVLLVMLLPWSAYQAGQSPFVTFFSKLGV

300   PYIGSIMNIVVLTAALSSLNSGLYCTGRILRSMAMGGSAPSFMAKMSRQH

350   VPYAGILATLVVYVVGVFLNYLVPSRVFEIVLNFASLGIIASWAFIIVCQ

400   MRLRKAIKEGKAADVSFKLPGAPFTSWLTLLFLLSVLVLMAFDYPNGTYT
                                ***********************
450   IAALPIIGILLVIGWFGVRKRVAEIHSTAPVVEEDEEKQEIVFKPETAS
```

sp|P23890|CADC_ECOLI Transcriptional activator CadC OS=Escherichia coli (strain K12) OX=83333 GN=cadC PE=1 SV=1

```
0     MQQPVVRVGEWLVTPSINQISRNGRQLTLEPRLIDLLVFFAQHSGEVLSR
                     ***************************
50    DELIDNVWKRSIVTNHVVTQSISELRKSLKDNDEDSPVYIATVPKRGYKL

100   MVPVIWYSEEEGEEIMLSSPPPIPEAVPATDSPSHSLNIQNTATPPEQSP

150   VKSKRFTTFWVWFFFLLSLGICVALVAFSSLDTRLPMSKSRILLNPRDID

200   INMVNKSCNSWSSPYQLSYAIGVGDLVATSLNTFSTFMVHDKINYNIDEP

250   SSSGKTLSIAFVNQRQYRAQQCFMSIKLVDNADGSTMLDKRYVITNGNQL

300   AIQNDLLESLSKALNQPWPQRMQETLQKILPHRGALLTNFYQAHDYLLHG

350   DDKSLNRASELLGEIVQSSPEFTYARAEKALVDIVRHSQHPLDEKQLAAL

400   NTEIDNIVTLPELNNLSIIYQIKAVSALVKGKTDESYQAINTGIDLEMSW

450   LNYVLLGKVYEMKGMNREAADAYLTAFNLRPGANTLYWIENGIFQTSVPY

500   VVPYLDKFLASE
```

sp|P39393|YJIV_ECOLI Putative uncharacterized protein YjiV OS=Escherichia coli (strain K12) OX=83333 GN=yjiV PE=5 SV=3

```
                                                 *****
0     MYVNQRQNCDCGSPVYEVAFCNDCNEPHLLARDKKGKLVQWENKGGDEFS
      ***********************
50    LQDEVPVEHDATEEKVEKENSFQPPLIIAAGETSEAGYTLQRLDRQTRRI

100   GVINNDSIPLIINDIEQVCSASGCGYRGMSGKQPFRRALLGGPFYVTNIV

150   PTVLEYCQDFTSDEGKEGVGPDSLPGRGRRLITFTDSRQGTARMAVRMQQ

200   EAERSRLRGSVVEILSWHQRTQTSTAPNANADLEKLAARAKQAREQAEEY

250   RSWGMPDQAKLSQAQAEQLEQAYQAATGGKAATILVSRTWTEMVNELKER

300   ADIRGPVLQYNHYLKPEVFNENGGPLKLSEMLLFREFMRRPKRTNSLETQ

350   GLVQVGYQGLEKIHKSPLHWQEKGLTLDDWRDFLKVTLDHYVRESNFTQL
```

       ****
400   DDELKNWIGSRFSSKFVRNPESKDPEDNQNRRWPQIRNGNVSHRLAKLLM

450   LGAGFKTVNAATIDIINTWLKEAWAQLTGPLAVLKPDGNRFYLPKEHMTF

500   SLITDAWICPVTNKILDTAFKGLTPYLPTHISFEHLTLAQYDTFVAQKVT

550   MPEIWKLDRSQEDYAEGLAKARDWVSHDPLIAQLRSENVWTDINDRVVEG

600   GFYYRTAEHSAQQSSERLQSYEKMFKNGQLNVLNCSTTMEMGVDTDRVMT

650   LASRSQQATIPGPEWHLNDELVVRSLGYKTVELNEFILPAKATNAVERVK

700   DIQIHKQLNGPLSQFGQRFWDVLFNDHEEAQSLMNNTRITGVHYTDRYLQ

750   NPVALALLGSILRPLKTKLTDGAEVTLDTLFKDKDRPGNRPFHDWMSIAD

800   FQDFADQWFAAALGRPVELTVFDSPRDIPHHRKLTVTFEDGQVLKIRFDQ

850   GMGYWRINFSSQWHYFDFRDDVSFQLVKMAQACKEGNVANSEESWATDVL

900   VEVIAS

sp|P76228|YNJI_ECOLI Inner membrane protein YnjI OS=Escherichia coli (strain K12) OX=83333 GN=ynjI PE=1 SV=2

0   MKKVLLQNHPGSEKYSFNGWEIFNSNFERMIKENKAMLLCKWGFYLTCVV

50   AVMFVFAAITSNGLNERGLITAGCSFLYLLIMMGLIVRAGFKAKKEQLHY

100   YQAKGIEPLSIEKLQALQLIAPYRFYHKQWSETLEFWPRKPEPGKDTFQY
           ****
150   HVLPFDSIDIISKRRESLEDQWGIEDSESYCALMEHFLSGDHGANTFKAN
                       ****
200   MEEAPEQVIALLNKFAVFPSDYISDCANHSSGKSSAKLIWAAELSWMISI
                          ****
250   SSTAFQNGTIEEELAWHYIMLASRKAHELFESEEDYQKNSQMGFLYWHIC

300   CYRRKLTDAELEACYRYDKQFWEHYSKKCRWPIRNVPWGASSVKYS

sp|P0ADU5|YGIW_ECOLI Protein YgiW OS=Escherichia coli (strain K12) OX=83333 GN=ygiW PE=1 SV=1

0   MKKFAAVIAVMALCSAPVMAAEQGGFSGPSATQSQAGGFQGPNGSVTTVE

50   SAKSLRDDTWVTLRGNIVERISDDLYVFKDASGTINVDIDHKRWNGVTVT
       ****
100   PKDTVEIQGEVDKDWNSVEIDVKQIRKVNP

sp|P00968|CARB_ECOLI Carbamoyl-phosphate synthase large chain OS=Escherichia coli (strain K12) OX=83333 GN=carB PE=1 SV=2

0   MPKRTDIKSILILGAGPIVIGQACEFDYSGAQACKALREEGYRVILVNSN

```
50    PATIMTDPEMADATYIEPIHWEVVRKIIEKERPDAVLPTMGGQTALNCAL

100   ELERQGVLEEFGVTMIGATADAIDKAEDRRRFDVAMKKIGLETARSGIAH

150   TMEEALAVAADVGFPCIIRPSFTMGGSGGGIAYNREEFEEICARGLDLSP
            ********************
200   TKELLIDESLIGWKEYEMEVVRDKNDNCIIVCSIENFDAMGIHTGDSITV

250   APAQTLTDKEYQIMRNASMAVLREIGVETGGSNVQFAVNPKNGRLIVIEM

300   NPRVSRSSALASKATGFPIAKVAAKLAVGYTLDELMNDITGGRTPASFEP

350   SIDYVVTKIPRFNFEKFAGANDRLTTQMKSVGEVMAIGRTQQESLQKALR

400   GLEVGATGFDPKVSLDDPEALTKIRRELKDAGADRIWYIADAFRAGLSVD

450   GVFNLTNIDRWFLVQIEELVRLEEKVAEVGITGLNADFLRQLKRKGFADA

500   RLAKLAGVREAEIRKLRDQYDLHPVYKRVDTCAAEFATDTAYMYSTYEEE

550   CEANPSTDREKIMVLGGGPNRIGQGIEFDYCCVHASLALREDGYETIMVN

600   CNPETVSTDYDTSDRLYFEPVTLEDVLEIVRIEKPKGVIVQYGGQTPLKL

650   ARALEAAGVPVIGTSPDAIDRAEDRERFQHAVERLKLKQPANATVTAIEM

700   AVEKAKEIGYPLVVRPSYVLGGRAMEIVYDEADLRRYFQTAVSVSNDAPV

750   LLDHFLDDAVEVDVDAICDGEMVLIGGIMEHIEQAGVHSGDSACSLPAYT

800   LSQEIQDVMRQQVQKLAFELQVRGLMNVQFAVKNNEVYLIEVNPRAARTV

850   PFVSKATGVPLAKVAARVMAGKSLAEQGVTKEVIPPYYSVKEVVLPFNKF

900   PGVDPLLGPEMRSTGEVMGVGRTFAEAFAKAQLGSNSTMKKHGRALLSVR

950   EGDKERVVDLAAKLLKQGFELDATHGTAIVLGEAGINPRLVNKVHEGRPH

1000  IQDRIKNGEYTYIINTTSGRRAIEDSRVIRRSALQYKVHYDTTLNGGFAT

1050  AMALNADATEKVISVQEMHAQIK
```

sp|P76150|YNEK_ECOLI Uncharacterized protein YneK OS=Escherichia coli (strain K12) OX=83333 GN=yneK PE=4 SV=1

```
0     MVTPVSISNYISLPDDFPVRNIAPQVKEVLKDFIDALSTIICNEEWRTSL

50    NINSATKKIFNNLDNLSYIQRTSFRGNDTLYNEKVQFKLTYPARNGRHKE

100   NIEFQVVINLSPIYLDNFRHDGEINIFCAPNPKPVTMGRVFQTGVERVLF

150   LFLNDFIEQFPMINPGVPIKRAHTPHIEPLPSDHHTAADYLRQFDLLVLN
                                       *****************
200   FISRGNFVILPRLWNNSEVHRWFVNKDPNLITAILDITDSELKEDLLQSL
```

      ****
250   MDSLGSNKHVLPEVCICFLSLLAEQESPHFQNLFLFFANMLLHYHQFMNP
       ****
300   NESDLNDVLMPASLSDDKIIKHMARRTLKLFVKNETPPKVTHEDLVKNRP

350   RSPVRPPIPATAKTPDLPERH

sp|Q47208|FDRA_ECOLI Protein FdrA OS=Escherichia coli (strain K12) OX=83333 GN=fdrA PE=1 SV=1
                 ****
0   MIHAFIKKGCFQDSVSLMIISRKLSESENVDDVSVMMGTPANKALLDTTG

50   FWHDDFNNATPNDICVAIRSEAADAGIAQAIMQQLEEALKQLAQGSGSSQ

100   ALTQVRRWDSACQKLPDANLALISVAGEYAAELANQALDRNLNVMMFSDN

150   VTLEDEIQLKTRAREKGLLVMGPDCGTSMIAGTPLAFANVMPEGNIGVIG

200   ASGTGIQELCSQIALAGEGITHAIGLGGRDLSREVGGISALTALEMLSAD

250   EKSEVLAFVSKPPAEAVRLKIVNAMKATGKPTVALFLGYTPAVARDENVW

300   FASSLDEAARLACLLSRVTARRNAIAPVSSGFICGLYTGGTLAAEAAGLL

350   AGHLGVEADDTHQHGMMLDADSHQIIDLGDDFYTVGRPHPMIDPTLRNQL

400   IADLGAKPQVRVLLLDVVIGFGATADPAASLVSAWQKACAARLDNQPLYA

450   IATVTGTERDPQCRSQQIATLEDAGIAVVSSLPEATLLAAALIHPLSPAA

500   QQHTPSLLENVAVINIGLRSFALELQSASKPVVHYQWSPVAGGNKKLARL

550   LERLQ

sp|P77335|HLYE_ECOLI Hemolysin E, chromosomal OS=Escherichia coli (strain K12) OX=83333 GN=hlyE PE=1 SV=4

0   MTEIVADKTVEVVKNAIETADGALDLYNKYLDQVIPWQTFDETIKELSRF

50   KQEYSQAASVLVGDIKTLLMDSQDKYFEATQTVYEWCGVATQLLAAYILL
       *********************   ******
100   FDEYNEKKASAQKDILIKVLDDGITKLNEAQKSLLVSSQSFNNASGKLLA
      ****
150   LDSQLTNDFSEKSSYFQSQVDKIRKEAYAGAAAGVVAGPFGLIISYSIAA

200   GVVEGKLIPELKNKLKSVQNFFTTLSNTVKQANKDIDAAKLKLTTEIAAI

250   GEIKTETETTRFYVDYDDLMLSLLKEAAKKMINTCNEYQKRHGKKTLFEV

300   PEV

sp|P33920|NDPA_ECOLI Nucleoid-associated protein YejK OS=Escherichia coli (strain K12) OX=83333 GN=yejK PE=1 SV=3

```
0     MSLDINQIALHQLIKRDEQNLELVLRDSLLEPTETVVEMVAELHRVYSAK

50    NKAYGLFSEESELAQTLRLQRQGEEDFLAFSRAATGRLRDELAKYPFADG

100   GFVLFCHYRYLAVEYLLVAVLSNLSSMRVNENLDINPTHYLDINHADIVA

150   RIDLTEWETNPESTRYLTFLKGRVGRKVADFFMDFLGASEGLNAKAQNRG
                                        ****************
200   LLQAVDDFTAEAQLDKAERQNVRQQVYSYCNEQLQAGEEIELKSLSKELA
      **********************
250   GVSEVSFTEFAAEKGYELEESFPADRSTLRQLTKFAGSGGGLTINFDAML

300   LGERIFWDPATDTLTIKGTPPNLRDQLQRRTSGGN
```

sp|P37327|YFDC_ECOLI Inner membrane protein YfdC OS=Escherichia coli (strain K12) OX=83333 GN=yfdC PE=1 SV=1

```
      **************************************
0     MDNDKIDQHSDEIEVESEEKERGKKIEIDEDRLPSRAMAIHEHIRQDGEK

50    ELERDAMALLWSAIAAGLSMGASLLAKGIFQVELEGVPGSFLLENLGYTF

100   GFIIVIMARQQLFTENTVTAVLPVMQKPTMSNVGLLIRLWGVVLLGNILG

150   TGIAAWAFEYMPIFNEETRDAFVKIGMDVMKNTPSEMFANAIISGWLIAT

200   MVWMFPAAGAAKIVVIILMTWLIALGDTTHIVVGSVEILYLVFNGTLHWS

250   DFIWPFALPTLAGNICGGTFIFALMSHAQIRNDMSNKRKAEARQKAERAE

300   NIKKNYKNPA
```

sp|P12008|AROC_ECOLI Chorismate synthase OS=Escherichia coli (strain K12) OX=83333 GN=aroC PE=1 SV=4

```
0     MAGNTIGQLFRVTTFGESHGLALGCIVDGVPPGIPLTEADLQHDLDRRRP

50    GTSRYTTQRREPDQVKILSGVFEGVTTGTSIGLLIENTDQRSQDYSAIKD

100   VFRPGHADYTYEQKYGLRDYRGGGRSSARETAMRVAAGAIAKKYLAEKFG
                                     *******************
150   IEIRGCLTQMGDIPLDIKDWSQVEQNPFFCPDPDKIDALDELMRALKKEG
      ***
200   DSIGAKVTVVASGVPAGLGEPVFDRLDADIAHALMSINAVKGVEIGDGFD

250   VVALRGSQNRDEITKDGFQSNHAGGILGGISSGQQIIAHMALKPTSSITV

300   PGRTINRFGEEVEMITKGRHDPCVGIRAVPIAEAMLAIVLMDHLLRQRAQ

350   NADVKTDIPRW
```

sp|P31446|YIDI_ECOLI Inner membrane protein YidI OS=Escherichia coli (strain K12) OX=83333 GN=yidI PE=1 SV=1

```
0     MGIIAQNKISSLGMLFGAIALMMGIIHFSFGPFSAPPPTFESIVADKTAE
```

****
50 IKRGLLAGIKGEKITTVEKKEDVDVDKILNQSGIALAIAALLCAFIGGMR

100 KENRWGIRGALVFGGGTLAFHTLLFGIGIVCSILLIFLIFSFLTGGSLV

sp|P76045|OMPG_ECOLI Outer membrane protein G OS=Escherichia coli (strain K12) OX=83333 GN=ompG PE=1 SV=1

0 MKKLLPCTALVMCAGMACAQAEERNDWHFNIGAMYEIENVEGYGEDMDGL

50 AEPSVYFNAANGPWRIALAYYQEGPVDYSAGKRGTWFDRPELEVHYQFLE

100 NDDFSFGLTGGFRNYGYHYVDEPGKDTANMQRWKIAPDWDVKLTDDLRFN

150 GWLSMYKFANDLNTTGYADTRVETETGLQYTFNETVALRVNYYLERGFNM

200 DDSRNNGEFSTQEIRAYLPLTLGNHSVTPYTRIGLDRWSNWDWQDDIERE
****
250 GHDFNRVGLFYGYDFQNGLSVSLEYAFEWQDHDEGDSDKFHYAGVGVNYS

300 F

sp|P05704|MCP3_ECOLI Methyl-accepting chemotaxis protein III OS=Escherichia coli (strain K12) OX=83333 GN=trg PE=1 SV=3

0 MNTTPSQRLGFLHHIRLVPLFACILGGILVLFALSSALAGYFLWQADRDQ

50 RDVTAEIEIRTGLANSSDFLRSARINMIQAGAASRIAEMEAMKRNIAQAE

100 SEIKQSQQGYRAYQNRPVKTPADEALDTELNQRFQAYITGMQPMLKYAKN

150 GMFEAIINHESEQIRPLDNAYTDILNKAVKIRSTRANQLAELAHQRTRLG

200 GMFMIGAFVLALVMTLITFMVLRRIVIRPLQHAAQRIEKIASGDLTMNDE

250 PAGRNEIGRLSRHLQQMQHSLGMTVGTVRQGAEEIYRGTSEISAGNADLS

300 SRTEEQAAAIEQTAASMEQLTATVKQNADNAHHASKLAQEASIKASDGGQ

350 TVSGVVKTMGAISTSSKKISEITAVINSIAFQTNILALNAAVEAARAGEQ
****
400 GRGFAVVASEVRTLASRSAQAAKEIEGLISESVRLIDLGSDEVATAGKTM

450 STIVDAVASVTHIMQEIAAASDEQSRGITQVSQAISEMDKVTQQNASLVE

500 EASAAAVSLEEQAARLTEAVDVFRLHKHSVSAEPRGAGEPVSFATV

sp|P54746|MNGB_ECOLI Mannosylglycerate hydrolase OS=Escherichia coli (strain K12) OX=83333 GN=mngB PE=1 SV=2

0 MKAVSRVHITPHMHWDREWYFTTEESRILLVNNMEEILCRLEQDNEYKYY

50 VLDGQTAILEDYFAVKPENKDRVKKQVEAGKLIIGPWYTQTDTTIVSAES

100   IVRNLMYGMRDCLAFGEPMKIGYLPDSFGMSGQLPHIYNGFGITRTMFWR
        ****
150   GCSERHGTDKTEFLWQSSDGSEVTAQVLPLGYAIGKYLPADENGLRKRLD

200   SYFDVLEKASVTKEILLPNGHDQMPLQQNIFEVMDKLREIYPQRKFVMSR

250   FEEVFEKIEAQRDNLATLKGEFIDGKYMRVHRTIGSTRMDIKIAHARIEN

300   KIVNLLEPLATLAWTLGFEYHHGLLEKMWKEILKNHAHDSIGCCCSDKVH

350   REIVARFELAEDMADNLIRFYMRKIADNMPQSDADKLVLFNLMPWPREEV

400   INTTVRLRASQFNLRDDRGQPVPYFIRHAREIDPGLIDRQIVHYGNYDPF

450   MEFDIQINQIVPSMGYRTLYIEANQPGNVIAAKSDAEGILENAFWQIALN
             ****
500   EDGSLQLVDKDSGVRYDRVLQIEESSDDGDEYDYSPAKEEWVITAANAKP

550   QCDIIHEAWQSRAVIRYDMAVPLNLSERSARQSTGRVGVVLVVTLSHNSR

600   RIDVDINLDNQADDHRLRVLVPTPFNTDSVLADTQFGSLTRPVNDSAMNN

650   WQQEGWKEAPVPVWNMLNYVALQEGRNGMAVFSEGLREFEVIGEEKKTFA

700   ITLLRGVGLLGKEDLLLRPGRPSGIKMPVPDSQLRGLLSCRLSLLSYTGT

750   PTAAGVAQQARAWLTPVQCYNKIPWDVMKLNKAGFNVPESYSLLKMPPVG

800   CLISALKKAEDRQEVILRLFNPAESATCDATVAFSREVISCSETMMDEHI

850   TTEENQGSNLSGPFLPGQSRTFSYRLA

sp|P09155|RND_ECOLI Ribonuclease D OS=Escherichia coli (strain K12) OX=83333 GN=rnd PE=1 SV=1

0   MNYQMITTDDALASLCEAVRAFPAIALDTEFVRTRTYYPQLGLIQLFDGE

50   HLALIDPLGITDWSPLKAILRDPSITKFLHAGSEDLEVFLNVFGELPQPL
                        ****
100   IDTQILAAFCGRPMSWGFASMVEEYSGVTLDKSESRTDWLARPLTERQCE

150   YAAADVWYLLPITAKLMVETEASGWLPAALDECRLMQMRRQEVVAPEDAW

200   RDITNAWQLRTRQLACLQLLADWRLRKARERDLAVNFVVREEHLWSVARY

250   MPGSLGELDSLGLSGSEIRFHGKTLLALVEKAQTLPEDALPQPMLNLMDM

300   PGYRKAFKAIKSLITDVSETHKISAELLASRRQINQLLNWHWKLKPQNNL

350   PELISGWRGELMAEALHNLLQEYPQ

sp|P0ADV9|LPTC_ECOLI Lipopolysaccharide export system protein LptC OS=Escherichia coli (strain K12) OX=83333 GN=lptC PE=1 SV=1

```
0     MSKARRWVIIVLSLAVLVMIGINMAEKDDTAQVVVNNNDPTYKSEHTDTL
                                          **************
50    VYNPEGALSYRLIAQHVEYYSDQAVSWFTQPVLTTFDKDKIPTWSVKADK
      ******
100   AKLTNDRMLYLYGHVEVNALVPDSQLRRITTDNAQINLVTQDVTSEDLVT

150   LYGTTFNSSGLKMRGNLRSKNAELIEKVRTSYEIQNKQTQP
```

sp|P0AA93|YPDA_ECOLI Sensor histidine kinase YpdA OS=Escherichia coli (strain K12) OX=83333 GN=ypdA PE=1 SV=1

```
0     MHEIFNMLLAVFDRAALMLICLFFLIRIRLFRELLHKSAHSPKELLAVTA

50    IFSLFALFSTWSGVPVEGSLVNVRIIAVMSGGILFGPWVGIITGVIAGIH

100   RYLIDIGGVTAIPCFITSILAGCISGWINLKIPKAQRWRVGILGGMLCET

150   LTMILVIVWAPTTALGIDIVSKIGIPMILGSVCIGFIVLLVQSVEGEKEA

200   SAARQAKLALDIANKTLPLFRHVNSESLRKVCEIIRDDIHADAVAITNTD

250   HVLAYVGVGEHNYQNGDDFISPTTRQAMNYGKIIIKNNDEAHRTPEIHSM

300   LVIPLWEKGVVTGTLKIYYCHAHQITSSLQEMAVGLSQIISTQLEVSRAE

350   QLREMANKAELRALQSKINPHFLFNALNAISSSIRLNPDTARQLIFNLSR
          ************************
400   YLRYNIELKDDEQIDIKKELYQIKDYIAIEQARFGDKLTVIYDIDEEVNC

450   CIPSLLIQPLVENAIVHGIQPCKGKGVVTISVAECGNRVRIAVRDTGHGI

500   DPKVIERVEANEMPGNKIGLLNVHHRVKLLYGEGLHIRRLEPGTEIAFYI

550   PNQRTPVASQATLLL
```

sp|P76347|YEEJ_ECOLI Uncharacterized protein YeeJ OS=Escherichia coli (strain K12) OX=83333 GN=yeeJ PE=3 SV=3

```
0     MATKKRSGEEINDRQILCGMGIKLRRLTAGICLITQLAFPMAAAAQGVVN

50    AATQQPVPAQIAIANANTVPYTLGALESAQSVAERFGISVAELRKLNQFR

100   TFARGFDNVRQGDELDVPAQVSEKKLTPPPGNSSDNLEQQIASTSQQIGS

150   LLAEDMNSEQAANMARGWASSQASGAMTDWLSRFGTARITLGVDEDFSLK

200   NSQFDFLHPWYETPDNLFFSQHTLHRTDERTQINNGLGWRHFTPTWMSGI

250   NFFFDHDLSRYHSRAGIGAEYWRDYLKLSSNGYLRLTNWRSAPELDNDYE

300   ARPANGWDVRAESWLPAWPHLGGKLVYEQYYGDEVALFDKDDRQSNPHAI

350   TAGLNYTPFPLMTFSAEQRQGKQGENDTRFAVDFTWQPGSAMQKQLDPNE
```

400   VAARRSLAGSRYDLVDRNNNIVLEYRKKELVRLTLTDPVTGKSGEVKSLV

450   SSLQTKYALKGYNVEATALEAAGGKVVTTGKDILVTLPAYRFTSTPETDN
                                    ****
500   TWPIEVTAEDVKGNLSNREQSMVVVQAPTLSQKDSSVSLSTQTLNADSHS

550   TATLTFIAHDAAGNPVVGLVLSTRHEGVQDITLSDWKDNGDGSYTQILTT
                              ****
600   GAMSGTLTLMPQLNGVDAAKAPAVVNIISVSSSRTHSSIKIDKDRYLSGN

650   PIEVTVELRDENDKPVKEQKQQLNNAVSIDNVKPGVTTDWKETADGVYKA

700   TYTAYTKGSGLTAKLLMQNWNEDLHTAGFIIDANPQSAKIATLSASNNGV

750   LANENAANTVSVNVADEGSNPINDHTVTFAVLSGSATSFNNQNTAKTDVN

800   GLATFDLKSSKQEDNTVEVTLENGVKQTLIVSFVGDSSTAQVDLQKSKNE

850   VVADGNDSVTMTATVRDAKGNLLNDVMVTFNVNSAEAKLSQTEVNSHDGI

900   ATATLTSLKNGDYRVTASVSSGSQANQQVNFIGDQSTAALTLSVPSGDIT
                           ****
950   VTNTAPQYMTATLQDKNGNPLKDKEITFSVPNDVASKFSISNGGKGMTDS

1000 NGVAIASLTGTLAGTHMIMARLANSNVSDAQPMTFVADKDRAVVVLQTSK

1050 AEIIGNGVDETTLTATVKDPSNHPVAGITVNFTMPQDVAANFTLENNGIA

1100 ITQANGEAHVTLKGKKAGTHTVTATLGNNNTSDSQPVTFVADKASAQVVL

1150 QISKDEITGNGVDSATLTATVKDQFDNEVNNLPVTFSSASSGLTLTPGVS

1200 NTNESGIAQATLAGVAFGEKTVTASLANNGASDNKTVHFIGDTAAAKIIE

1250 LAPVPDSIIAGTPQNSSGSVITATVVDNNGFPVKGVTVNFTSNAATAEMT

1300 NGGQAVTNEQGKATVTYTNTRSSIESGARPDTVEASLENGSSTLSTSINV

1350 NADASTAHLTLLQALFDTVSAGETTSLYIEVKDNYGNGVPQQEVTLSVSP

1400 SEGVTPSNNAIYTTNHDGNFYASFTATKAGVYQLTATLENGDSMQQTVTY

1450 VPNVANAEITLAASKDPVIADNNDLTTLTATVADTEGNAIANTEVTFTLP

1500 EDVKANFTLSDGGKVITDAEGKAKVTLKGTKAGAHTVTASMTGGKSEQLV

1550 VNFIADTLTAQVNLNVTEDNFIANNVGMTRLQATVTDGNGNPLANEAVTF

1600 TLPADVSASFTLGQGGSAITDINGKAEVTLSGTKSGTYPVTVSVNNYGVS

1650 DTKQVTLIADAGTAKLASLTSVYSFVVSTTEGATMTASVTDANGNPVEGI

1700 KVNFRGTSVTLSSTSVETDDRGFAEILVTSTEVGLKTVSASLADKPTEVI

1750 SRLLNASADVNSATITSLEIPEGQVMVAQDVAVKAHVNDQFGNPVAHQPV

1800 TFSAEPSSQMIISQNTVSTNTQGVAEVTMTPERNGSYMVKASLPNGASLE

1850 KQLEAIDEKLTLTASSPLIGVYAPTGATLTATLTSANGTPVEGQVINFSV

1900 TPEGATLSGGKVRTNSSGQAPVVLTSNKVGTYTVTASFHNGVTIQTQTTV

1950 KVTGNSSTAHVASFIADPSTIAATNTDLSTLKATVEDGSGNLIEGLTVYF

2000 ALKSGSATLTSLTAVTDQNGIATTSVKGAMTGSVTVSAVTTAGGMQTVDI
             ****
2050 TLVAGPADTSQSVLKSNRSSLKGDYTDSAELRLVLHDISGNPIKVSEGME

2100 FVQSGTNVPYIKISAIDYSLNINGDYKATVTGGGEGIATLIPVLNGVHQA

2150 GLSTTIQFTRAEDKIMSGTVSVNGTDLPTTTFPSQGFTGAYYQLNNDNFA

2200 PGKTAADYEFSSSASWVDVDATGKVTFKNVGSNSERITATPKSGGPSYVY

2250 EIRVKSWWVNAGEAFMIYSLAENFCSSNGYTLPRANYLNHCSSRGIGSLY

2300 SEWGDMGHYTTDAGFQSNMYWSSSPANSSEQYVVSLATGDQSVFEKLGFA

2350 YATCYKNL

sp|P0ABB8|ATMA_ECOLI Magnesium-transporting ATPase, P-type 1 OS=Escherichia coli (strain K12) OX=83333 GN=mgtA PE=1 SV=1

0   MFKEIFTRLIRHLPSRLVHRDPLPGAQQTVNTVVPPSLSAHCLKMAVMPE

50   EELWKTFDTHPEGLNQAEVESAREQHGENKLPAQQPSPWWVHLWVCYRNP

100   FNILLTILGAISYATEDLFAAGVIALMVAISTLLNFIQEARSTKAADALK

150   AMVSNTATVLRVINDKGENGWLEIPIDQLVPGDIIKLAAGDMIPADLRIL

200   QARDLFVAQASLTGESLPVEKAATTRQPEHSNPLECDTLCFMGTTVVSGT

250   AQAMVIATGANTWFGQLAGRVSEQESEPNAFQQGISRVSMLLIRFMLVMA

300   PVVLLINGYTKGDWWEAALFALSVAVGLTPEMLPMIVTSTLARGAVKLSK

350   QKVIVKHLDAIQNFGAMDILCTDKTGTLTQDKIVLENHTDISGKTSERVL
                            ****
400   HSAWLNSHYQTGLKNLLDTAVLEGTDEESARSLASRWQKIDEIPFDFERR

450   RMSVVVAENTEHHQLVCKGALQEILNVCSQVRHNGEIVPLDDIMLRKIKR

500   VTDTLNRQGLRVVAVATKYLPAREGDYQRADESDLILEGYIAFLDPPKET

550   TAPALKALKASGITVKILTGDSELVAAKVCHEVGLDAGEVVIGSDIETLS

600   DDELANLAQRTTLFARLTPMHKERIVTLLKREGHVVGFMGDGINDAPALR

650   AADIGISVDGAVDIAREAADIILLEKSLMVLEEGVIEGRRTFANMLKYIK

700   MTASSNFGNVFSVLVASAFLPFLPMLPLHLLIQNLLYDVSQVAIPFDNVD

750   DEQIQKPQRWNPADLGRFMIFFGPISSIFDILTFCLMWWVFHANTPETQT

800   LFQSGWFVVGLLSQTLIVHMIRTRRVPFIQSCASWPLMIMTVIVMIVGIA

850   LPFSPLASYLQLQALPLSYFPWLVAILAGYMTLTQLVKGFYSRRYGWQ

sp|P0CF70|INSE5_ECOLI Transposase InsE for insertion sequence IS3E OS=Escherichia coli (strain K12) OX=83333 GN=insE5 PE=3 SV=1

****

0   MTKTVSTSKKPRKQHSPEFRSEALKLAERIGVTAAARELSLYESQLYNWR

50   SKQQNQQTSSERELEMSTEIARLKRQLAERDEELAILQKAATYFAKRLK

sp|P22523|MUKB_ECOLI Chromosome partition protein MukB OS=Escherichia coli (strain K12) OX=83333 GN=mukB PE=1 SV=2

0   MIERGKFRSLTLINWNGFFARTFDLDELVTTLSGGNGAGKSTTMAAFVTA

50   LIPDLTLLHFRNTTEAGATSGSRDKGLHGKLKAGVCYSMLDTINSRHQRV

100   VVGVRLQQVAGRDRKVDIKPFAIQGLPMSVQPTQLVTETLNERQARVLPL

150   NELKDKLEAMEGVQFKQFNSITDYHSLMFDLGIIARRLRSASDRSKFYRL

200   IEASLYGGISSAITRSLRDYLLPENSGVRKAFQDMEAALRENRMTLEAIR

250   VTQSDRDLFKHLISEATNYVAADYMRHANERRVHLDKALEFRRELHTSRQ

300   QLAAEQYKHVDMARELAEHNGAEGDLEADYQAASDHLNLVQTALRQQEKI

350   ERYEADLDELQIRLEEQNEVVAEAIERQQENEARAEAAELEVDELKSQLA

400   DYQQALDVQQTRAIQYNQAIAALNRAKELCHLPDLTADCAAEWLETFQAK

450   ELEATEKMLSLEQKMSMAQTAHSQFEQAYQLVVAINGPLARNEAWDVARE

500   LLREGVDQRHLAEQVQPLRMRLSELEQRLREQQEAERLLADFCKRQGKNF

550   DIDELEALHQELEARIASLSDSVSNAREERMALRQEQEQLQSRIQSLMQR

600   APVWLAAQNSLNQLSEQCGEEFTSSQDVTEYLQQLLEREREAIVERDEVG

650   ARKNAVDEEIERLSQPGGSEDQRLNALAERFGGVLLSEIYDDVSLEDAPY

****

700   FSALYGPSRHAIVVPDLSQVTEHLEGLTDCPEDLYLIEGDPQSFDDSVFS

****

750   VDELEKAVVVKIADRQWRYSRFPEVPLFGRAARESRIESLHAEREVLSER

800   FATLSFDVQKTQRLHQAFSRFIGSHLAVAFESDPEAEIRQLNSRRVELER

```
850   ALSNHENDNQQQRIQFEQAKEGVTALNRILPRLNLLADDSLADRVDEIRE

900   RLDEAQEAARFVQQFGNQLAKLEPIVSVLQSDPEQFEQLKEDYAYSQQMQ
                        ************************
950   RDARQQAFALTEVVQRRAHFSYSDSAEMLSGNSDLNEKLRERLEQAEAER
                           ***********************
1000  TRAREALRGHAAQLSQYNQVLASLKSSYDTKKELLNDLQRELQDIGVRAD

1050  SGAEERARIRRDELHAQLSNNRSRRNQLEKALTFCEAEMDNLTRKLRKLE

1100  RDYFEMREQVVTAKAGWCAVMRMVKDNGVERRLHRRELAYLSADDLRSMS

1150  DKALGALRLAVADNEHLRDVLRMSEDPKRPERKIQFFVAVYQHLRERIRQ

1200  DIIRTDDPVEAIEQMEIELSRLTEELTSREQKLAISSRSVANIIRKTIQR

1250  EQNRIRMLNQGLQNVSFGQVNSVRLNVNVRETHAMLLDVLSEQHEQHQDL

1300  FNSNRLTFSEALAKLYQRLNPQIDMGQRTPQTIGEELLDYRNYLEMEVEV

1350  NRGSDGWLRAESGALSTGEAIGTGMSILVMVVQSWEDESRRLRGKDISPC

1400  RLLFLDEAARLDARSIATLFELCERLQMQLIIAAPENISPEKGTTYKLVR

1450  KVFQNTEHVHVVGLRGFAPQLPETLPGTDEAPSQAS
```

sp|P0ABR1|DINI_ECOLI DNA damage-inducible protein I OS=Escherichia coli (strain K12) OX=83333 GN=dinI PE=1 SV=1

```
0     MRIEVTIAKTSPLPAGAIDALAGELSRRIQYAFPDNEGHVSVRYAAANNL
           **********************
50    SVIGATKEDKQRISEILQETWESADDWFVSE
```

sp|P0A8N0|MATP_ECOLI Macrodomain Ter protein OS=Escherichia coli (strain K12) OX=83333 GN=matP PE=1 SV=1

```
0     MKYQQLENLESGWKWKYLVKKHREGELITRYIEASAAQEAVDVLLSLENE

50    PVLVNGWIDKHMNPELVNRMKQTIRARRKRHFNAEHQHTRKKSIDLEFIV
                          ************************
100   WQRLAGLAQRRGKTLSETIVQLIEDAENKEKYANKMSSLKQDLQALLGKE

150
```

sp|P76035|YCIW_ECOLI Uncharacterized protein YciW OS=Escherichia coli (strain K12) OX=83333 GN=yciW PE=4 SV=2

```
0     MEQRHITGKSHWYHETQSSTTEYDVLPLVPEAAKVSDPFLLDVILEKETL

50    APFLSWLDPARVLAVDLFPDQLTVTRSQTFTAYERLSTALTVAQVCGVQR

100   LCNYYSARLTPLPGPDSTRESNHRLAQITQYARQLASSPSIIDNRSRQHL
```

150   NDVGLTAWDCVIISQIIGFIGFQARTIATFQAYLGHPVRWLPGLEIQNYA
      ****
200   DASLFADESLRWRSSYEVEKLPEEHTKSSTAELCQLAEILSLHPISLSLL
                                         ****
250   EKLLNSTRGNTQPDNQLAALLCARINGSPACFATCMDSSNEYKKISTLMR
      ******
300   KGENEINQWADRHSVERATVQAIQWLTRAPDRFSAAQFSPLLEHEKSSTQ

350   IINLLVWSGLCGWINRLKIALGETY

sp|P69348|YOEB_ECOLI Toxin YoeB OS=Escherichia coli (strain K12) OX=83333 GN=yoeB PE=1 SV=1
       ****
0   MKLIWSEESWDDYLYWQETDKRIVKKINELIKDTRRTPFEGKGKPEPLKH

50   NLSGFWSRRITEEHRLVYAVTDDSLLIAACRYHY

sp|P0A7U3|RS19_ECOLI 30S ribosomal protein S19 OS=Escherichia coli (strain K12) OX=83333 GN=rpsS PE=1 SV=2
               ****
0   MPRSLKKGPFIDLHLLKKVEKAVESGDKKPLRTWSRRSTIFPNMIGLTIA

50   VHNGRQHVPVFVTDEMVGHKLGEFAPTRTYRGHAADKKAKKK

sp|P42909|PTPB1_ECOLI N-acetylgalactosamine-specific phosphotransferase enzyme IIB component 1 OS=Escherichia coli (strain K12) OX=83333 GN=agaB PE=4 SV=1

0   MTSPNILLTRIDNRLVHGQVGVTWTSTIGANLLVVVDDVVANDDIQQKLM

50   GITAETYGFGIRFFTIEKTINVIGKAAPHQKIFLICRTPQTVRKLVEGGI
               ****
100   DLKDVNVGNMHFSEGKKQISSKVYVDDQDLTDLRFIKQRGVNVFIQDVPG

150   DQKEQIPD

sp|P33235|FLGK_ECOLI Flagellar hook-associated protein 1 OS=Escherichia coli (strain K12) OX=83333 GN=flgK PE=1 SV=3

0   MSSLINNAMSGLNAAQAALNTASNNISSYNVAGYTRQTTIMAQANSTLGA

50   GGWVGNGVYYVSGVQREYDAFITNQLRAAQTQSSGLTARYEQMSKIDNMLS

100   TSTSSLATQMQDFFTSLQTLVSNAEDPAARQALIGKSEGLVNQFKTTDQY

150   LRDQDKQVNIAIGASVDQINNYAKQIASLNDQISRLTGVGAGASPNNLLD

200   QRDQLVSELNQIVGVEVSVQDGGTYNITMANGYSLVQGSTARQLAAVPSS

250   ADPSRTTVAYVDGTAGNIEIPEKLLNTGSLGGILTFRSQDLDQTRNTLGQ

300   LALAFAEAFNTQHKAGFDANGDAGEDFFAIGKPAVLQNTKNKGDVAIGAT

350   VTDASAVLATDYKISFDNNQWQVTRLASNTTFTVTPDANGKVAFDGLELT

```
                               ***********************
400   FTGTPAVNDSFTLKPVSDAIVNMDVLITDEAKIAMASEEDAGDSDNRNGQ

450   ALLDLQSNSKTVGGAKSFNDAYASLVSDIGNKTATLKTSSATQGNVVTQL

500   SNQQQSISGVNLDEEYGNLQRFQQYYLANAQVLQTANAIFDALINIR
```

sp|P21499|RNR_ECOLI Ribonuclease R OS=Escherichia coli (strain K12) OX=83333 GN=rnr PE=1 SV=2

```
0     MSQDPFQEREAEKYANPIPSREFILEHLTKREKPASRDELAVELHIEGEE

50    QLEGLRRRLRAMERDGQLVFTRRQCYALPERLDLVKGTVIGHRDGYGFLR

100   VEGRKDDLYLSSEQMKTCIHGDQVLAQPLGADRKGRREARIVRVLVPKTS

150   QIVGRYFTEAGVGFVVPDDSRLSFDILIPPDQIMGARMGFVVVVELTQRP

200   TRRTKAVGKIVEVLGDNMGTGMAVDIALRTHEIPYIWPQAVEQQVAGLKE

250   EVPEEAKAGRVDLRDLPLVTIDGEDARDFDDAVYCEKKRGGGWRLWVAIA

300   DVSYYVRPSTPLDREARNRGTSVYFPSQVIPMLPEVLSNGLCSLNPQVDR

350   LCMVCEMTVSSKGRLTGYKFYEAVMSSHARLTYTKVWHILQGDQDLREQY
                   ************************
400   APLVKHLEELHNLYKVLDKAREERGGISFESEEAKFIFNAERRIERIEQT

450   QRNDAHKLIEECMILANISAARFVEKAKEPALFRIHDKPSTEAITSFRSV

500   LAELGLELPGGNKPEPRDYAELLESVADRPDAEMLQTMLLRSMKQAIYDP

550   ENRGHFGLALQSYAHFTSPIRRYPDLTLHRAIKYLLAKEQGHQGNTTETG

600   GYHYSMEEMLQLGQHCSMAERRADEATRDVADWLKCDFMLDQVGNVFKGV

650   ISSVTGFGFFVRLDDLFIDGLVHVSSLDNDYYRFDQVGQRLMGESSGQTY

700   RLGDRVEVRVEAVNMDERKIDFSLISSERAPRNVGKTAREKAKKGDAGKK
              ***************************************
750   GGKRRQVGKKVNFEPDSAFRGEKKTKPKAAKKDARKAKKPSAKTQKIAAA

800   TKAKRAAKKKVAE
```

sp|P0A705|IF2_ECOLI Translation initiation factor IF-2 OS=Escherichia coli (strain K12) OX=83333 GN=infB PE=1 SV=1

```
0     MTDVTIKTLAAERQTSVERLVQQFADAGIRKSADDSVSAQEKQTLIDHLN

50    QKNSGPDKLTLQRKTRSTLNIPGTGGKSKSVQIEVRKKRTFVKRDPQEAE
                        ********************************
100   RLAAEEQAQREAEEQARREAEESAKREAQQKAEREAAEQAKREAAEQAKR
      **************************************************
150   EAAEKDKVSNQQDDMTKNAQAEKARREQEAAELKRKAEEEARRKLEEEAR
```

      ***** ***********************
200   RVAEEARRMAEENKWTDNAEPTEDSSDYHVTTSQHARQAEDESDREVEGG
             ****
250   RGRGRNAKAARPKKGNKHAESKADREEARAAVRGGKGGKRKGSSLQQGFQ

300   KPAQAVNRDVVIGETITVGELANKMAVKGSQVIKAMMKLGAMATINQVID

350   QETAQLVAEEMGHKVILRRENELEEAVMSDRDTGAAAEPRAPVVTIMGHV

400   DHGKTSLLDYIRSTKVASGEAGGITQHIGAYHVETENGMITFLDTPGHAA

450   FTSMRARGAQATDIVVLVVAADDGVMPQTIEAIQHAKAAQVPVVVAVNKI

500   DKPEADPDRVKNELSQYGILPEEWGGESQFVHVSAKAGTGIDELLDAILL

550   QAEVLELKAVRKGMASGAVIESFLDKGRGPVATVLVREGTLHKGDIVLCG

600   FEYGRVRAMRNELGQEVLEAGPSIPVEILGLSGVPAAGDEVTVVRDEKKA
                                                 *****
650   REVALYRQGKFREVKLARQQKSKLENMFANMTEGEVHEVNIVLKADVQGS
      ****
700   VEAISDSLLKLSTDEVKVKIIGSGVGGITETDATLAAASNAILVGFNVRA

750   DASARKVIEAESLDLRYYSVIYNLIDEVKAAMSGMLSPELKQQIIGLAEV

800   RDVFKSPKFGAIAGCMVTEGVVKRHNPIRVLRDNVVIYEGELESLRRFKD

850   DVNEVRNGMECGIGVKNYNDVRTGDVIEVFEIIEIQRTIA

sp|P45756|GSPA_ECOLI Putative general secretion pathway protein A OS=Escherichia coli (strain K12) OX=83333 GN=gspA PE=2 SV=1

0   MSTRREVILSWLCEKRQTWRLCYLLGEAGSGKTWLAQQLQKDKHRRVITL

50   SLVVSWQGKAAWIVTDDNAAEQGCRDSAWTRDEMAGQLLHALHRTDSRCP

100   LIIIENAHLNHRRILDDLQRAISLIPDGQFLLIGRPDRKVERDFKKQGIE

150   LVSIGRLTEHELKASILEGQNIDQPDLLLTARVLKRIALLCRGDRRKLAL

200   AGETIRLLQQAEQTSVFTAKQWRMIYRILGDNRPRKMQLAVVMSGTIIAL

250   TCGWLLLSSFTATLPVPAWLIPVTPVVKQDMTKDIAHVVMRDSEALSVLY

300   GVWGYEVPADSAWCDQAVRAGLACKSGNASLQTLVDQNLPWIASLKVGDK
                                             ****
350   KLPVVVVRVGEASVDVLVGQQTWTLTHKWFESVWTGDYLLLWKMSPEGES
      ****
400   TITRDSSEEEILWLETMLNRALHISTEPSAEWRPLLVEKIKQFQKSHHLK

450   TDGVVGFSTLVHLWQVAGESAYLYRDEANISPETTVKGK

sp|P77699|TFAD_ECOLI Protein TfaD OS=Escherichia coli (strain K12) OX=83333 GN=tfaD PE=3 SV=2

0 MNAMGSDYIREVNVVKSARVGYSKMLLGVYAYFIEHKQRNTLIPAGFVAV

50 FNSDESSWHLVEDHRGKTVYDVASGDALFISELGPLPENVTWLSPEGEFQ
****

100 KWNGTAWVKDAEAEKLFRIREAEETKNSLMQVASEHIAPLQDAVDLEIAT

150 EEETSLLEAWKKYRVLLNRVDTSTAPDIEWPTNPVRE

sp|P10423|IAP_ECOLI Alkaline phosphatase isozyme conversion protein OS=Escherichia coli (strain K12) OX=83333 GN=iap PE=3 SV=1

0 MFSALRHRTAALALGVCFILPVHASSPKPGDFANTQARHIATFFPGRMTG

50 TPAEMLSADYIRQQFQQMGYRSDIRTFNSRYIYTARDNRKSWHNVTGSTV

100 IAAHEGKAPQQIIIMAHLDTYAPLSDADADANLGGLTLQGMDDNAAGLGV
****

150 MLELAERLKNTPTEYGIRFVATSGEEEGKLGAENLLKRMSDTEKKNTLLV

200 INLDNLIVGDKLYFNSGVKTPEAVRKLTRDRALAIARSHGIAATTNPGLN

250 KNYPKGTGCCNDAEIFDKAGIAVLSVEATNWNLGNKDGYQQRAKTPAFPA

300 GNSWHDVRLDNHQHIDKALPGRIERRCRDVMRIMLPLVKELAKAS

sp|P0A6P5|DER_ECOLI GTPase Der OS=Escherichia coli (strain K12) OX=83333 GN=der PE=1 SV=1

0 MVPVVALVGRPNVGKSTLFNRLTRTRDALVADFPGLTRDRKYGRAEIEGR

50 EFICIDTGGIDGTEDGVETRMAEQSLLAIEEADVVLFMVDARAGLMPADE

100 AIAKHLRSREKPTFLVANKTDGLDPDQAVVDFYSLGLGEIYPIAASHGRG
****

150 VLSLLEHVLLPWMEDLAPQEEVDEDAEYWAQFEAEENGEEEEEDDFDPQS
****

200 LPIKLAIVGRPNVGKSTLTNRILGEERVVVYDMPGTTRDSIYIPMERDGR

250 EYVLIDTAGVRKRGKITDAVEKFSVIKTLQAIEDANVVMLVIDAREGISD

300 QDLSLLGFILNSGRSLVIVVNKWDGLSQEVKEQVKETLDFRLGFIDFARV

350 HFISALHGSGVGNLFESVREAYDSSTRRVGTSMLTRIMTMAVEDHQPPLV

400 RGRRVKLKYAHAGGYNPPIVVIHGNQVKDLPDSYKRYLMNYFRKSLDVMG

450 SPIRIQFKEGENPYANKRNTLTPTQMRKRKRLMKHIKKNK

sp|P11875|SYR_ECOLI Arginine--tRNA ligase OS=Escherichia coli (strain K12) OX=83333 GN=argS PE=1 SV=1

0 MNIQALLSEKVRQAMIAAGAPADCEPQVRQSAKVQFGDYQANGMMAVAKK

50 LGMAPRQLAEQVLTHLDLNGIASKVEIAGPGFINIFLDPAFLAEHVQQAL

100 ASDRLGVATPEKQTIVVDYSAPNVAKEMHVGHLRSTIIGDAAVRTLEFLG
****
150 HKVIRANHVGDWGTQFGMLIAWLEKQQQENAGEMELADLEGFYRDAKKHY
****
200 DEDEEFAERARNYVVKLQSGDEYFREMWRKLVDITMTQNQITYDRLNVTL

250 TRDDVMGESLYNPMLPGIVADLKAKGLAVESEGATVVFLDEFKNKEGEPM

300 GVIIQKKDGGYLYTTTDIACAKYRYETLHADRVLYYIDSRQHQHLMQAWA

350 IVRKAGYVPESVPLEHHMFGMMLGKDGKPFKTRAGGTVKLADLLDEALER

400 ARRLVAEKNPDMPADELEKLANAVGIGAVKYADLSKNRTTDYIFDWDNML

450 AFEGNTAPYMQYAYTRVLSVFRKAEIDEEQLAAAPVIIREDREAQLAARL

500 LQFEETLTVVAREGTPHVMCAYLYDLAGLFSGFYEHCPILSAENEEVRNS

550 RLKLAQLTAKTLKLGLDTLGIETVERM

sp|P76484|YFBN_ECOLI Uncharacterized protein YfbN OS=Escherichia coli (strain K12) OX=83333 GN=yfbN PE=4 SV=1

0 MEWLSEIRKLRKNVPVGIQVARRLLERTGGDVDEAIKLFHIDQINILTAK

50 ADVTHQEAENVLLATNYDIAEALRRIDEQRYTLTELILRKNKDAGDALNN

100 IALAIEYEWDLKRKFWFGFADIQLLPPVLQTFMLVYEWHEYVGWEGMECG
****
150 IFFESDHTHQQLQALGLLELAQKMVTARIRYDELKDKAENFHEITEDDIF
**
200 KMLIIHCDQLAREVDSILLQFVKDNIDVFPCRHNRHEL

sp|P30015|LHR_ECOLI Probable ATP-dependent helicase lhr OS=Escherichia coli (strain K12) OX=83333 GN=lhr PE=3 SV=2

0 MADNPDPSSLLPDVFSPATRDWFLRAFKQPTAVQPQTWHVAARSEHALVI

50 APTGSGKTLAAFLYALDRLFREGGEDTREAHKRKTSRILYISPIKALGTD

100 VQRNLQIPLKGIADERRRRGETEVNLRVGIRTGDTPAQERSKLTRNPPDI

150 LITTPESLYLMLTSRARETLRGVETVIIDEVHAVAGSKRGAHLALSLERL

200 DALLHTSAQRIGLSATVRSASDVAAFLGGDRPVTVVNPPAMRHPQIRIVV

250 PVANMDDVSSVASGTGEDSHAGREGSIWPYIETGILDEVLRHRSTIVFTN

300 SRGLAEKLTARLNELYAARLQRSPSIAVDAAHFESTSGATSNRVQSSDVF

350 IARSHHGSVSKEQRAITEQALKSGELRCVVATSSLELGIDMGAVDLVIQV

400 ATPLSVASGLQRIGRAGHQVGGVSKGLFFPRTRRDLVDSAVIVECMFAGR

450 LENLTPPHNPLDVLAQQTVAAAAMDALQVDEWYSRVRRAAPWKDLPRRVF

500 DATLDMLSGRYPSGDFSAFRPKLVWNRETGILTARPGAQLLAVTSGGTIP
****
550 DRGMYSVLLPEGEEKAGSRRVGELDEEMVYESRVNDIITLGATSWRIQQI

600 TRDQVIVTPAPGRSARLPFWRGEGNGRPAELGEMIGDFLHLLADGAFFSG

650 TIPPWLAEENTIANIQGLIEEQRNATGIVPGSRHLVLERCRDEIGDWRII

700 LHSPYGRRVHEPWAVAIAGRIHALWGADASVVASDDGIVARIPDTDGKLP

750 DAAIFLFEPEKLLQIVREAVGSSALFAARFRECAARALLMPGRTPGHRTP

800 LWQQRLRASQLLEIAQGYPDFPVILETLRECLQDVYDLPALERLMRRLNG

850 GEIQISDVTTTTPSPFATSLLFGYVAEFMYQSDAPLAERRASVLSLDSEL

900 LRNLLGQVDPGELLDPQVIRQVEEELQRLAPGRRAKGEEGLFDLLRELGP

950 MTVEDLAQRHTGSSEEVASYLENLLAVKRIFPAMISGQERLACMDDAARL

1000 RDALGVRLPESLPEIYLHRVSYPLRDLFLRYLRAHALVTAEQLAHEFSLG

1050 IAIVEEQLQQLREQGLVMNLQQDIWVSDEVFRRLRLRSLQAAREATRPVA

1100 ATTYARLLLERQGVLPATDGSPALFASTSPGVYEGVDGVMRVIEQLAGVG

1150 LPASLWESQILPARVRDYSSEMLDELLATGAVIWSGQKKLGEDDGLVALH

1200 LQEYAAESFTPAEADQANRSALQQAIVAVLADGGAWFAQQISQRIRDKIG

1250 ESVDLSALQEALWALVWQGVITSDIWAPLRALTRSSSNARTSTRRSHRAR

1300 RGRPVYAQPVSPRVSYNTPNLAGRWSLLQVEPLNDTERMLALAENMLDRY

1350 GIISRQAVIAENIPGGFPSMQTLCRSMEDSGRIMRGRFVEGLGGAQFAER

1400 LTIDRLRDLATQATQTRHYTPVALSANDPANVWGNLLPWPAHPATLVPTR

1450 RAGALVVVSGGKLLLYLAQGGKKMLVWQEKEELLAPEVFHALTTALRREP

1500 RLRFTLTEVNDLPVRQTPMFTLLREAGFSSSPQGLDWG

sp|P64581|YQJD_ECOLI Uncharacterized protein YqjD OS=Escherichia coli (strain K12) OX=83333 GN=yqjD PE=1 SV=1
****
0 MSKEHTTEHLRAELKSLSDTLEEVLSSSGEKSKEELSKIRSKAEQALKQS
**
50 RYRLGETGDAIAKQTRVAAARADEYVRENPWTGVGIGAAIGVVLGVLLSR

100 R

```
sp|P0AF43|YJBB_ECOLI Uncharacterized protein YjbB OS=Escherichia coli
(strain K12) OX=83333 GN=yjbB PE=4 SV=1

0     MLTLLHLLSAVALLVWGTHIVRTGVMRVFGARLRTVLSRSVEKKPLAFCA

50    GIGVTALVQSSNATTMLVTSFVAQDLVALAPALVIVLGADVGTALMARIL

100   TFDLSWLSPLLIFIGVIFFLGRKQSRAGQLGRVGIGLGLILLALELIVQA

150   VTPITQANGVQVIFASLTGDILLDALIGAMFAIISYSSLAAVLLTATLTA

200   AGIISFPVALCLVIGANLGSGLLAMLNNSAANAAARRVALGSLLFKLVGS

250   LIILPFVHLLAETMGKLSLPKAELVIYFHVFYNLVRCLVMLPFVDPMARF

300   CKTIIRDEPELDTQLRPKHLDVSALDTPTLALANAARETLRIGDAMEQMM
                                                  ************
350   EGLNKVMHGEPRQEKELRKLADDINVLYTAIKLYLARMPKEELAEEESRR
      *********
400   WAEIIEMSLNLEQASDIVERMGSEIADKSLAARRAFSLDGLKELDALYEQ

450   LLSNLKLAMSVFFSGDVTSARRLRRSKHRFRILNRRYSHAHVDRLHQQNV

500   QSIETSSLHLGLLGDMQRLNSLFCSVAYSVLEQPDEDEGRDEY

sp|P0A6Y5|HSLO_ECOLI 33 kDa chaperonin OS=Escherichia coli (strain K12)
OX=83333 GN=hslO PE=1 SV=1

0     MPQHDQLHRYLFENFAVRGELVTVSETLQQILENHDYPQPVKNVLAELLV

50    ATSLLTATLKFDGDITVQLQGDGPMNLAVINGNNNQQMRGVARVQGEIPE

100   NADLKTLVGNGYVVITITPSEGERYQGVVGLEGDTLAACLEDYFMRSEQL

150   PTRLFIRTGDVDGKPAAGGMLLQVMPAQNAQQDDFDHLATLTETIKTEEL
                                                  ************
200   LTLPANEVLWRLYHEEEVTVYDPQDVEFKCTCSRERCADALKTLPDEEVD
      ***********
250   SILAEDGEIDMHCDYCGNHYLFNAMDIAEIRNNASPADPQVH

sp|P25519|HFLX_ECOLI GTPase HflX OS=Escherichia coli (strain K12)
OX=83333 GN=hflX PE=1 SV=3
                      *************************
0     MFDRYDAGEQAVLVHIYFTQDKDMEDLQEFESLVSSAGVEALQVITGSRK

50    APHPKYFVGEGKAVEIAEAVKATGASVVLFDHALSPAQERNLERLCECRV

100   IDRTGLILDIFAQRARTHEGKLQVELAQLRHLATRLVRGWTHLERQKGGI

150   GLRGPGETQLETDRRLLRNRIVQIQSRLERVEKQREQGRQSRIKADVPTV

200   SLVGYTNAGKSTLFNRITEARVYAADQLFATLDPTLRRIDVADVGETVLA
```

```
250   DTVGFIRHLPHDLVAAFKATLQETRQATLLLHVIDAADVRVQENIEAVNT
                      *********************
300   VLEEIDAHEIPTLLVMNKIDMLEDFEPRIDRDEENKPNRVWLSAQTGAGI

350   PQLFQALTERLSGEVAQHTLRLPPQEGRLRSRFYQLQAIEKEWMEEDGSV

400   SLQVRMPIVDWRRLCKQEPALIDYLI
```

sp|P23909|MUTS_ECOLI DNA mismatch repair protein MutS OS=Escherichia coli (strain K12) OX=83333 GN=mutS PE=1 SV=1

```
0     MSAIENFDAHTPMMQQYLRLKAQHPEILLFYRMGDFYELFYDDAKRASQL

50    LDISLTKRGASAGEPIPMAGIPYHAVENYLAKLVNQGESVAICEQIGDPA

100   TSKGPVERKVVRIVTPGTISDEALLQERQDNLLAAIWQDSKGFGYATLDI

150   SSGRFRLSEPADRETMAAELQRTNPAELLYAEDFAEMSLIEGRRGLRRRP

200   LWEFEIDTARQQLNLQFGTRDLVGFGVENAPRGLCAAGCLLQYAKDTQRT

250   TLPHIRSITMEREQDSIIMDAATRRNLEITQNLAGGAENTLASVLDCTVT

300   PMGSRMLKRWLHMPVRDTRVLLERQQTIGALQDFTAGLQPVLRQVGDLER

350   ILARLALRTARPRDLARMRHAFQQLPELRAQLETVDSAPVQALREKMGEF

400   AELRDLLERAIIDTPPVLVRDGGVIASGYNEELDEWRALADGATDYLERL

450   EVRERERTGLDTLKVGFNAVHGYYIQISRGQSHLAPINYMRRQTLKNAER
          **********************
500   YIIPELKEYEDKVLTSKGKALALEKQLYEELFDLLLPHLEALQQSASALA

550   ELDVLVNLAERAYTLNYTCPTFIDKPGIRITEGRHPVVEQVLNEPFIANP

600   LNLSPQRRMLIITGPNMGGKSTYMRQTALIALMAYIGSYVPAQKVEIGPI

650   DRIFTRVGAADDLASGRSTFMVEMTETANILHNATEYSLVLMDEIGRGTS

700   TYDGLSLAWACAENLANKIKALTLFATHYFELTQLPEKMEGVANVHLDAL

750   EHGDTIAFMHSVQDGAASKSYGLAVAALAGVPKEVIKRARQKLRELESIS

800   PNAAATQVDGTQMSLLSVPEETSPAVEALENLDPDSLTPRQALEWIYRLK

850   SLV
```

sp|P37590|PMRD_ECOLI Signal transduction protein PmrD OS=Escherichia coli (strain K12) OX=83333 GN=pmrD PE=1 SV=2

```
0     MEWLVKKSCCNKQDNRHVLMLCDAGGAIKMIAEVKSDFAVKVGDLLSPLQ
              ********************
50    NALYCINREKLHTVKVLSASSYSPDEWERQCKVAGKTQ
```

sp|P0AC33|FUMA_ECOLI Fumarate hydratase class I, aerobic OS=Escherichia coli (strain K12) OX=83333 GN=fumA PE=1 SV=2

```
                   **********************
0     MSNKPFHYQAPFPLKKDDTEYYLLTSEHVSVSEFEGQEILKVAPEALTLL

50    ARQAFHDASFMLRPAHQQQVADILRDPEASENDKYVALQFLRNSDIAAKG

100   VLPTCQDTGTAIIVGKKGQRVWTGGGDEAALARGVYNTYIEDNLRYSQNA

150   PLDMYKEVNTGTNLPAQIDLYAVDGDEYKFLCIAKGGGSANKTYLYQETK

200   ALLTPGKLKNYLVEKMRTLGTAACPPYHIAFVIGGTSAETNLKTVKLASA

250   KYYDELPTEGNEHGQAFRDVELEKELLIEAQNLGLGAQFGGKYFAHDIRV

300   IRLPRHGASCPVGMGVSCSADRNIKAKINRQGIWIEKLEHNPGKYIPEEL

350   RKAGEGEAVRVDLNRPMKEILAQLSQYPVSTRLSLNGTIIVGRDIAHAKL

400   KERMDNGEGLPQYIKDHPIYYAGPAKTPEGYASGSLGPTTAGRMDSYVDQ

450   LQAQGGSMIMLAKGNRSQQVTDACKKHGGFYLGSIGGPAAVLAQGSIKSL

500   ECVEYPELGMEAIWKIEVEDFPAFILVDDKGNDFFQQIQLTQCTRCVK
```

sp|P52005|TORY_ECOLI Cytochrome c-type protein TorY OS=Escherichia coli (strain K12) OX=83333 GN=torY PE=1 SV=2

```
0     MRGKKRIGLLFLLIAVVVGGGGLLLAQKVLHKTSDTAFCLSCHSMSKPFE
                                      ******************
50    EYQGTVHFSNQKGIRAECADCHIPKSGMDYLFAKLKASKDIYHEFVSGKI
      ********************                  ************
100   DSDDKFEAHRQEMAETVWKELKATDSATCRSCHSFDAMDIASQSESAQKM
      *************
150   HNKAQKDSETCIDCHKGIAHFPPEIKMDDNAAHELESQAATSVTNGAHIY

200   PFKTSHIGELATVNPGTDLTVVDASGKQPIVLLQGYQMQGSENTLYLAAG

250   QRLALATLSEEGIKALTVNGEWQADEYGNQWRQASLQGALTDPALADRKP

300   LWQYAEKLDDTYCAGCHAPIAADHYTVNAWPSIAKGMGARTSMSENELDI

350   LTRYFQYNAKDITEKQ
```

sp|P11349|NARH_ECOLI Respiratory nitrate reductase 1 beta chain OS=Escherichia coli (strain K12) OX=83333 GN=narH PE=1 SV=3

```
0     MKIRSQVGMVLNLDKCIGCHTCSVTCKNVWTSREGVEYAWFNNVETKPGQ

50    GFPTDWENQEKYKGGWIRKINGKLQPRMGNRAMLLGKIFANPHLPGIDDY
                                                  ************
100   YEPFDFDYQNLHTAPEGSKSQPIARPRSLITGERMAKIEKGPNWEDDLGG
      **************
150   EFDKLAKDKNFDNIQKAMYSQFENTFMMYLPRLCEHCLNPACVATCPSGA
```

200   IYKREEDGIVLIDQDKCRGWRMCITGCPYKKIYFNWKSGKSEKCIFCYPR

250   IEAGQPTVCSETCVGRIRYLGVLLYDADAIERAASTENEKDLYQRQLDVF

300   LDPNDPKVIEQAIKDGIPLSVIEAAQQSPVYKMAMEWKLALPLHPEYRTL

350   PMVWYVPPLSPIQSAADAGELGSNGILPDVESLRIPVQYLANLLTAGDTK

400   PVLRALKRMLAMRHYKRAETVDGKVDTRALEEVGLTEAQAQEMYRYLAIA

450   NYEDRFVVPSSHRELAREAFPEKNGCGFTFGDGCHGSDTKFNLFNSRRID

500   AIDVTSKTEPHP

sp|P75800|PDEI_ECOLI Probable cyclic di-GMP phosphodiesterase PdeI OS=Escherichia coli (strain K12) OX=83333 GN=pdeI PE=1 SV=1

0   MLSLYEKIKIRLIILFLLAALSFIGLFFIINYQLVSERAVKRADSRFELI

50   QKNVGYFFKDIERSALTLKDSLYLLKNTEEIQRAVILKMEMMPFLDSVGL

100   VLDDNKYYLFSRRANDKIVVYHQEQVNGPLVDESGRVIFADFNPSKRPWS

150   VASDDSNNSWNPAYNCFDRPGKKCISFTLHINGKDHDLLAVDKIHVDLNW

200   RYLNEYLDQISANDEVLFLKQGHEIIAKNQLAREKLIIYNSEGNYNIIDS

```
250   VDTEYIEKTSAVPNNALFEIYFYYPGGNLLNASDKLFYLPFAFIIIVLLV
                                                ************
300   VYLMTTRVFRRQFSEMTELVNTLAFLPDSTDQIEALKIREGDAKEIISIK
      ***************************
```

350   NSIAEMKDAEIERSNKLLSLISYDQESGFIKNMAIIESNNNQYLAVGIIK

400   LCGLEAVEAVFGVDERNKIVRKLCQRIAEKYAQCCDIVTFNADLYLLLCR

450   ENVQTFTRKIAMVNDFDSSFGYRNLRIHKSAICEPLQGENAWSYAEKLKL

500   AISSIRDHMFSEFIFCDDAKLNEIEENIWIARNIRHAMEIGELFLVYQPI

550   VDINTRAILGAEALCRWVSAERGIISPLKFITIAEDIGFINELGYQIIKT

600   AMGEFRHFSQRASLKDDFLLHINVSPWQLNEPHFHERFTTIMKENGLKAN

650   SLCVEITETVIERINEHFYLNIEQLRKQGVRISIDDFGTGLSNLKRFYEI

700   NPDSIKVDSQFTGDIFGTAGKIVRIIFDLARYNRIPVIAEGVESEDVARE

750   LIKLGCVQAQGYLYQKPMPFSAWDKSGKLVKE

sp|P0A6E6|ATPE_ECOLI ATP synthase epsilon chain OS=Escherichia coli (strain K12) OX=83333 GN=atpC PE=1 SV=2

0   MAMTYHLDVVSAEQQMFSGLVEKIQVTGSEGELGIYPGHAPLLTAIKPGM

```
                                            ************
50    IRIVKQHGHEEFIYLSGGILEVQPGNVTVLADTAIRGQDLDEARAMEAKR
      ****************
100   KAEEHISSSHGDVDYAQASAELAKAIAQLRVIELTKKAM
```

sp|P0ACD4|ISCU_ECOLI Iron-sulfur cluster assembly scaffold protein IscU OS=Escherichia coli (strain K12) OX=83333 GN=iscU PE=1 SV=1

```
0     MAYSEKVIDHYENPRNVGSFDNNDENVGSGMVGAPACGDVMKLQIKVNDE
                    ********************
50    GIIEDARFKTYGCGSAIASSSLVTEWVKGKSLDEAQAIKNTDIAEELELP

100   PVKIHCSILAEDAIKAAIADYKSKREAK
```

sp|P0A894|RAPZ_ECOLI RNase adapter protein RapZ OS=Escherichia coli (strain K12) OX=83333 GN=rapZ PE=1 SV=1

```
0     MVLMIVSGRSGSGKSVALRALEDMGFYCVDNLPVVLLPDLARTLADREIS

50    AAVSIDVRNMPESPEIFEQAMSNLPDAFSPQLLFLDADRNTLIRRYSDTR
      ********************************
100   RLHPLSSKNLSLESAIDKESDLLEPLRSRADLIVDTSEMSVHELAEMLRT

150   RLLGKRERELTMVFESFGFKHGIPIDADYVFDVRFLPNPHWDPKLRPMTG

200   LDKPVAAFLDRHTEVHNFIYQTRSYLELWLPMLETNNRSYLTVAIGCTGG

250   KHRSVYIAEQLADYFRSRGKNVQSRHRTLEKRKP
```

sp|P39361|SGCR_ECOLI Putative sgc region transcriptional regulator OS=Escherichia coli (strain K12) OX=83333 GN=sgcR PE=4 SV=1

```
0     MSQQRPDRIKQMLHYLWQHRHLSTQQAMELFGYAEATVRRDFQYIVNQYP
           **************************
50    GMIRGHGCLDFDDSTDDKEYVFDVKRTLQSVAKREIAALARTMIKDGDCF

100   FLDSGSTCLELAKCLADARVKVICNDIKIANELGCFPHVESYIIGGLIRP

150   GYFSVGESLALEMINAFSVERAFISCDALSLETGITNATMFEVGVKTRII

200   QRSREVILMADHSKFDAVEPHAVATLSCIKTIISDSGLPETIAQRYQRAG

250   CQLFLPHSIK
```

sp|P37773|MPL_ECOLI UDP-N-acetylmuramate--L-alanyl-gamma-D-glutamyl-meso-2,6-diaminoheptandioate ligase OS=Escherichia coli (strain K12) OX=83333 GN=mpl PE=1 SV=3

```
0     MRIHILGICGTFMGGLAMLARQLGHEVTGSDANVYPPMSTLLEKQGIELI

50    QGYDASQLEPQPDLVIIGNAMTRGNPCVEAVLEKNIPYMSGPQWLHDFVL

100   RDRWVLAVAGTHGKTTTAGMATWILEQCGYKPGFVIGGVPGNFEVSAHLG
```

```
150   ESDFFVIEADEYDCAFFDKRSKFVHYCPRTLILNNLEFDHADIFDDLKAI

200   QKQFHHLVRIVPGQGRIIWPENDINLKQTMAMGCWSEQELVGEQGHWQAK
          ********************
250   KLTTDASEWEVLLDGEKVGEVKWSLVGEHNMHNGLMAIAAARHVGVAPAD

300   AANALGSFINARRRLELRGEANGVTVYDDFAHHPTAILATLAALRGKVGG

350   TARIIAVLEPRSNTMKMGICKDDLAPSLGRADEVFLLQPAHIPWQVAEVA

400   EACVQPAHWSGDVDTLADMVVKTAQPGDHILVMSNGGFGGIHQKLLDGLA

450   KKAEAAQ
```

sp|P19934|TOLA_ECOLI Tol-Pal system protein TolA OS=Escherichia coli (strain K12) OX=83333 GN=tolA PE=1 SV=1

```
0     MSKATEQNDKLKRAIIISAVLHVILFAALIWSSFDENIEASAGGGGGSSI
               *****************************************
50    DAVMVDSGAVVEQYKRMQSQESSAKRSDEQRKMKEQQAAEELREKQAAEQ
      *********       ********************      ********
100   ERLKQLEKERLAAQEQKKQAEEAAKQAELKQKQAEEAAAKAAADAKAKAE
      *************************************
150   ADAKAAEEAAKKAAADAKKKAEAEAAKAAAEAQKKAEAAAAALKKKAEAA
           *********************************************
200   EAAAAEARKKAATEAAEKAKAEAEKKAAAEKAAADKKAAAEKAAADKKAA
      *************************
250   EKAAAEKAAADKKAAAEKAAADKKAAAAKAAAEKAAAAKAAAEADDIFGE

300   LSSGKNAPKTGGGAKGNNASPAGSGNTKNNGASGADINNYAGQIKSAIES

350   KFYDASSYAGKTCTLRIKLAPDGMLLDIKPEGGDPALCQAALAAAKLAKI

400   PKPPSQAVYEVFKNAPLDFKP
```

sp|P0A7W7|RS8_ECOLI 30S ribosomal protein S8 OS=Escherichia coli (strain K12) OX=83333 GN=rpsH PE=1 SV=2

```
                                            ************
0     MSMQDPIADMLTRIRNGQAANKAAVTMPSSKLKVAIANVLKEEGFIEDFK
      ***********
50    VEGDTKPELELTLKYFQGKAVVESIQRVSRPGLRIYKRKDELPKVMAGLG

100   IAVVSTSKGVMTDRAARQAGLGGEIICYVA
```

sp|P0AFW8|ROF_ECOLI Protein rof OS=Escherichia coli (strain K12) OX=83333 GN=rof PE=1 SV=1

```
                                 ***********************
0     MNDTYQPINCDDYDNLELACQHHLMLTLELKDGEKLQAKASDLVSRKNVE

50    YLVVEAAGETRELRLDKITSFSHPEIGTVVVSES
```

sp|P05458|PTRA_ECOLI Protease 3 OS=Escherichia coli (strain K12) OX=83333 GN=ptrA PE=1 SV=1

```
0     MPRSTWFKALLLLVALWAPLSQAETGWQPIQETIRKSDKDNRQYQAIRLD

50    NGMVVLLVSDPQAVKSLSALVVPVGSLEDPEAYQGLAHYLEHMSLMGSKK

100   YPQADSLAEYLKMHGGSHNASTAPYRTAFYLEVENDALPGAVDRLADAIA

150   EPLLDKKYAERERNAVNAELTMARTRDGMRMAQVSAETINPAHPGSKFSG

200   GNLETLSDKPGNPVQQALKDFHEKYYSANLMKAVIYSNKPLPELAKMAAD

250   TFGRVPNKESKKPEITVPVVTDAQKGIIIHYVPALPRKVLRVEFRIDNNS

300   AKFRSKTDELITYLIGNRSPGTLSDWLQKQGLVEGISANSDPIVNGNSGV

350   LAISASLTDKGLANRDQVVAAIFSYLNLLREKGIDKQYFDELANVLDIDF

400   RYPSITRDMDYVEWLADTMIRVPVEHTLDAVNIADRYDAKAVKERLAMMT

450   PQNARIWYISPKEPHNKTAYFVDAPYQVDKISAQTFADWQKKAADIALSL
       *********************************
500   PELNPYIPDDFSLIKSEKKYDHPELIVDESNLRVVYAPSRYFASEPKADV

550   SLILRNPKAMDSARNQVMFALNDYLAGLALDQLSNQASVGGISFSTNANN

600   GLMVNANGYTQRLPQLFQALLEGYFSYTATEDQLEQAKSWYNQMMDSAEK

650   GKAFEQAIMPAQMLSQVPYFSRDERRKILPSITLKEVLAYRDALKSGARP

700   EFMVIGNMTEAQATTLARDVQKQLGADGSEWCRNKDVVVDKKQSVIFEKA

750   GNSTDSALAAVFVPTGYDEYTSSAYSSLLGQIVQPWFYNQLRTEEQLGYA

800   VFAFPMSVGRQWGMGFLLQSNDKQPSFLWERYKAFFPTAEAKLRAMKPDE
                         ************************
850   FAQIQQAVITQMLQAPQTLGEEASKLSKDFDRGNMRFDSRDKIVAQIKLL

900   TPQKLADFFHQAVVEPQGMAILSQISGSQNGKAEYVHPEGWKVWENVSAL

950   QQTMPLMSEKNE
```

sp|P28638|YHDJ_ECOLI DNA adenine methyltransferase YhdJ OS=Escherichia coli (strain K12) OX=83333 GN=yhdJ PE=1 SV=3

```
0     MRTGCEPTRFGNEAKTIIHGDALAELKKIPAESVDLIFADPPYNIGKNFD

50    GLIEAWKEDLFIDWLFEVIAECHRVLKKQGSMYIMNSTENMPFIDLQCRK

100   LFTIKSRIVWSYDSSGVQAKKHYGSMYEPILMMVKDAKNYTFNGDAILVE

150   AKTGSQRALIDYRKNPPQPYNHQKVPGNVWDFPRVRYLMDEYENHPTQKP

200   EALLKRIILASSNPGDIVLDPFAGSFTTGAVAIASGRKFIGIEINSEYIK
                  ****************************
250   MGLRRLDVASHYSAEELAKVKKRKTGNLSKRSRLSEVDPDLITK
```

```
sp|P09127|HEMX_ECOLI Protein HemX OS=Escherichia coli (strain K12)
OX=83333 GN=hemX PE=1 SV=1

0     MTEQEKTSAVVEETREAVDTTSQPVATEKKSKNNTALILSAVAIAIALAA

50    GIGLYGWGKQQAVNQTATSDALANQLTALQKAQESQKAELEGIIKQQAAQ

100   LKQANRQQETLAKQLDEVQQKVATISGSDAKTWLLAQADFLVKLAGRKLW

150   SDQDVTTAALLKSADASLADMNDPSLITVRRAITDDIASLSAVSQVDYD
                                  **********************
200   GIILKLNQLSNQVDNLRLADNDSDGSPMDSDGEELSSSISEWRINLQKSW

250   QNFMDNFITIRRRDDTAVPLLAPNQDIYLRENIRSRLLVAAQAVPRHQEE

300   TYRQALENVSTWVRAYYDTDDATTKAFLDEVDQLSQQNISMDLPETLQSQ

350   AMLEKLMQTRVRNLLAQPAAGTTEAKPAPAPQADTPAAAPQGE

sp|P32053|INTA_ECOLI Prophage integrase IntA OS=Escherichia coli (strain
K12) OX=83333 GN=intA PE=1 SV=2

0     MARKTKPLTDTEIKAAKPKDADYQLYDGDGLTLLIKSSGSKLWQFRYYRP

50    LTKQRTKQSFGAYPAVSLSDARKLRAESKVLLAKDIDPQEHQKEQVRNSQ
                       ********************
100   EAKTNTFLLVAERWWNVKKTSVTEDYADDIWRSLERDIFPAIGDISITEI

150   KAHTLVKAVQPVQARGALETVRRLCQRINEVMIYAQNTGLIDAVPSVNIG

200   KAFEKPQKKNMPSIRPDQLPQLMHTMRTASISMSTRCLFMWQLLTITRPA

250   EAAEARWDEIDFNASEWKIPAARMKMNRDHTVPLSDGALAILEMMKPLSG

300   GREFIFPSRIKPNQPMNSQTVNAALKRAGLGGVLVSHGLRSIASTALNEE

350   GFPPDVIEAALAHVDKNEVRRAYNRSDYLEQRRPMMQWWADLVKAADSGS

400   IVLTHLSKIRLVG

sp|P27241|RFAZ_ECOLI Lipopolysaccharide core biosynthesis protein RfaZ
OS=Escherichia coli (strain K12) OX=83333 GN=rfaZ PE=4 SV=2
         ************************
0     MKNIRYIDKKDVENLIENKISDDVIIFLSGPTSQKTPLSVLRTKDIIAVN

50    GSAQYLLSNNIVPFIYVLTDVRFLHQRRDDFYKFSQRSRYTIVNVDVYEH

100   ASKEDKLYILQNCLVLRSFYRREKGGFIKKIKFNILRQIHKELLISVPLS

150   KKGRLVGFCKDISLGYCSCHTIAFAAIQIAYSLKYARIICSGLDLTGSCS
                                                    ****
200   RFYDENKNPMPSELSRDLFKILPFFRFMHDNVKDINIYNLSDDTAISYDV
      *******************************
```

250   IPFIKLQDISAEESKDMTRKKMQYRTSTDSYAN

sp|P76483|YFBM_ECOLI Protein YfbM OS=Escherichia coli (strain K12) OX=83333 GN=yfbM PE=1 SV=1

```
              ********************
0     MGMIGYFAEIDSEKINQLLESTEKPLMDNIHDTLSGLRRLDIDKRWDFLH

50    FGLTGTSAFDPAKNDPLSRAVLGEHSLEDGIDGFLGLTWNQELAATIDRL

100   ESLDRNELRKQFSIKRLNEMEIYPGVTFSEELEGQLFASIMLDMEKLISA

150   YRRMLRQGNHALTVIVG
```

sp|P38038|CYSJ_ECOLI Sulfite reductase [NADPH] flavoprotein alpha-component OS=Escherichia coli (strain K12) OX=83333 GN=cysJ PE=1 SV=4

```
0     MTTQVPPSALLPLNPEQLARLQAATTDLTPTQLAWVSGYFWGVLNQQPAA

50    LAATPAPAAEMPGITIISASQTGNARRVAEALRDDLLAAKLNVKLVNAGD

100   YKFKQIASEKLLIVVTSTQGEGEPPEEAVALHKFLFSKKAPKLENTAFAV
      *************************
150   FSLGDSSYEFFCQSGKDFDSKLAELGGERLLDRVDADVEYQAAASEWRAR

200   VVDALKSRAPVAAPSQSVATGAVNEIHTSPYSKDAPLVASLSVNQKITGR

250   NSEKDVRHIEIDLGDSGMRYQPGDALGVWYQNDPALVKELVELLWLKGDE

300   PVTVEGKTLPLNEALQWHFELTVNTANIVENYATLTRSETLLPLVGDKAK

350   LQHYAATTPIVDMVRFSPAQLDAEALINLLRPLTPRLYSIASSQAEVENE

400   VHVTVGVVRYDVEGRARAGGASSFLADRVEEEGEVRVFIEHNDNFRLPAN

450   PETPVIMIGPGTGIAPFRAFMQQRAADEAPGKNWLFFGNPHFTEDFLYQV

500   EWQRYVKDGVLTRIDLAWSRDQKEKVYVQDKLREQGAELWRWINDGAHIY

550   VCGDANRMAKDVEQALLEVIAEFGGMDTEAADEFLSELRVERRYQRDVY
```

sp|P76062|RACR_ECOLI Prophage repressor RacR OS=Escherichia coli (strain K12) OX=83333 GN=racR PE=4 SV=1

```
                                        ****************
0     MLSGKDLGRAIEQAINKKIASGSVKSKAEVARHFKVQPPSIYDWIKKGSI
      ***************
50    SKDKLPELWRFFSDVVGPEHWGLNEYPIPTPTNSDTKSELLDINNLYQAA

100   SDEIRAIVAFLLSGNATEPDWVDHDVRAYIAAMEMKVGKYLKALESERKS

150   QNITKTGT
```

sp|P0A8B5|YBAB_ECOLI Nucleoid-associated protein YbaB OS=Escherichia coli (strain K12) OX=83333 GN=ybaB PE=1 SV=1

```
0     MFGKGGLGNLMKQAQQMQEKMQKMQEEIAQLEVTGESGAGLVKVTINGAH
       ************************
50    NCRRVEIDPSLLEDDKEMLEDLVAAAFNDAARRIEETQKEKMASVSSGMQ

100   LPPGFKMPF
```

sp|P08194|GLPT_ECOLI Glycerol-3-phosphate transporter OS=Escherichia coli (strain K12) OX=83333 GN=glpT PE=1 SV=1

```
0     MLSIFKPAPHKARLPAAEIDPTYRRLRWQIFLGIFFGYAAYYLVRKNFAL

50    AMPYLVEQGFSRGDLGFALSGISIAYGFSKFIMGSVSDRSNPRVFLPAGL

100   ILAAAVMLFMGFVPWATSSIAVMFVLLFLCGWFQGMGWPPCGRTMVHWWS

150   QKERGGIVSVWNCAHNVGGGIPPLLFLLGMAWFNDWHAALYMPAFCAILV
                        **********************
200   ALFAFAMMRDTPQSCGLPPIEEYKNDYPDDYNEKAEQELTAKQIFMQYVL

250   PNKLLWYIAIANVFVYLLRYGILDWSPTYLKEVKHFALDKSSWAYFLYEY

300   AGIPGTLLCGWMSDKVFRGNRGATGVFFMTLVTIATIVYWMNPAGNPTVD

350   MICMIVIGFLIYGPVMLIGLHALELAPKKAAGTAAGFTGLFGYLGGSVAA

400   SAIVGYTVDFFGWDGGFMVMIGGSILAVILLIVVMIGEKRRHEQLLQERN

450   GG
```

sp|P76558|MAO2_ECOLI NADP-dependent malic enzyme OS=Escherichia coli (strain K12) OX=83333 GN=maeB PE=1 SV=1

```
0     MDDQLKQSALDFHEFPVPGKIQVSPTKPLATQRDLALAYSPGVAAPCLEI
                                                      **
50    EKDPLKAYKYTARGNLVAVISNGTAVLGLGNIGALAGKPVMEGKGVLFKK
      ******************
100   FAGIDVFDIEVDELDPDKFIEVVAALEPTFGGINLEDIKAPECFYIEQKL

150   RERMNIPVFHDDQHGTAIISTAAILNGLRVVEKNISDVRMVVSGAGAAAI

200   ACMNLLVALGLQKHNIVVCDSKGVIYQGREPNMAETKAAYAVVDDGKRTL

250   DDVIEGADIFLGCSGPKVLTQEMVKKMARAPMILALANPEPEILPPLAKE

300   VRPDAIICTGRSDYPNQVNNVLCFPFIFRGALDVGATAINEEMKLAAVRA

350   IAELAHAEQSEVVASAYGDQDLSFGPEYIIPKPFDPRLIVKIAPAVAKAA

400   MESGVATRPIADFDVYIDKLTEFVYKTNLFMKPIFSQARKAPKRVVLPEG

450   EEARVLHATQELVTLGLAKPILIGRPNVIEMRIQKLGLQIKAGVDFEIVN

500   NESDPRFKEYWTEYFQIMKRRGVTQEQAQRALISNPTVIGAIMVQRGEAD
```

```
550   AMICGTVGDYHEHFSVVKNVFGYRDGVHTAGAMNALLLPSGNTFIADTYV

600   NDEPDAEELAEITLMAAETVRRFGIEPRVALLSHSNFGSSDCPSSSKMRQ

650   ALELVRERAPELMIDGEMHGDAALVEAIRNDRMPDSSLKGSANILVMPNM

700   EAARISYNLLRVSSSEGVTVGPVLMGVAKPVHVLTPIASVRRIVNMVALA

750   VVEAQTQPL
```

sp|P0ABA0|ATPF_ECOLI ATP synthase subunit b OS=Escherichia coli (strain K12) OX=83333 GN=atpF PE=1 SV=1

```
0     MNLNATILGQAIAFVLFVLFCMKYVWPPLMAAIEKRQKEIADGLASAERA
       ********************
50    HKDLDLAKASATDQLKKAKAEAQVIIEQANKRRSQILDEAKAEAEQERTK
                                     *******************
100   IVAQAQAEIEAERKRAREELRKQVAILAVAGAEKIIERSVDEAANSDIVD
      **
150   KLVAEL
```

sp|P0AG24|SPOT_ECOLI Bifunctional (p)ppGpp synthase/hydrolase SpoT OS=Escherichia coli (strain K12) OX=83333 GN=spoT PE=1 SV=1

```
0     MYLFESLNQLIQTYLPEDQIKRLRQAYLVARDAHEGQTRSSGEPYITHPV
                                              **********
50    AVACILAEMKLDYETLMAALLHDVIEDTPATYQDMEQLFGKSVAELVEGV
      *********************
100   SKLDKLKFRDKKEAQAENFRKMIMAMVQDIRVILIKLADRTHNMRTLGSL

150   RPDKRRRIARETLEIYSPLAHRLGIHHIKTELEELGFEALYPNRYRVIKE

200   VVKAARGNRKEMIQKILSEIEGRLQEAGIPCRVSGREKHLYSIYCKMVLK

250   EQRFHSIMDIYAFRVIVNDSDTCYRVLGQMHSLYKPRPGRVKDYIAIPKA

300   NGYQSLHTSMIGPHGVPVEVQIRTEDMDQMAEMGVAAHWAYKEHGETSTT
                  ****************************
350   AQIRAQRWMQSLLELQQSAGSSFEFIESVKSDLFPDEIYVFTPEGRIVEL

400   PAGATPVDFAYAVHTDIGHACVGARVDRQPYPLSQPLTSGQTVEIITAPG

450   ARPNAAWLNFVVSSKARAKIRQLLKNLKRDDSVSLGRRLLNHALGGSRKL

500   NEIPQENIQRELDRMKLATLDDLLAEIGLGNAMSVVVAKNLQHGDASIPP

550   ATQSHGHLPIKGADGVLITFAKCCRPIPGDPIIAHVSPGKGLVIHHESCR

600   NIRGYQKEPEKFMAVEWDKETAQEFITEIKVEMFNHQGALANLTAAINTT

650   TSNIQSLNTEEKDGRVYSAFIRLTARDRVHLANIMRKIRVMPDVIKVTRN

700   RN
```

sp|P0DMC7|RCSB_ECOLI Transcriptional regulatory protein RcsB OS=Escherichia coli (strain K12) OX=83333 GN=rcsB PE=1 SV=1

```
0     MNNMNVIIADDHPIVLFGIRKSLEQIEWVNVVGEFEDSTALINNLPKLDA

50    HVLITDLSMPGDKYGDGITLIKYIKRHFPSLSIIVLTMNNNPAILSAVLD
                                                   *************
100   LDIEGIVLKQGAPTDLPKALAALQKGKKFTPESVSRLLEKISAGGYGDKR
      ********          ************************
150   LSPKESEVLRLFAEGFLVTEIAKKLNRSIKTISSQKKSAMMKLGVENDIA

200   LLNYLSSVTLSPADKD
```

sp|P0ABH9|CLPA_ECOLI ATP-dependent Clp protease ATP-binding subunit ClpA OS=Escherichia coli (strain K12) OX=83333 GN=clpA PE=1 SV=1

```
0     MLNQELELSLNMAFARAREHRHEFMTVEHLLLALLSNPSAREALEACSVD

50    LVALRQELEAFIEQTTPVLPASEEERDTQPTLSFQRVLQRAVFHVQSSGR
                                                       *********
100   NEVTGANVLVAIFSEQESQAAYLLRKHEVSRLDVVNFISHGTRKDEPTQS
      *************
150   SDPGSQPNSEEQAGGEERMENFTTNLNQLARVGGIDPLIGREKELERAIQ

200   VLCRRRKNNPLLVGESGVGKTAIAEGLAWRIVQGDVPEVMADCTIYSLDI

250   GSLLAGTKYRGDFEKRFKALLKQLEQDTNSILFIDEIHTIIGAGAASGGQ

300   VDAANLIKPLLSSGKIRVIGSTTYQEFSNIFEKDRALARRFQKIDITEPS

350   IEETVQIINGLKPKYEAHHDVRYTAKAVRAAVELAVKYINDRHLPDKAID
                                                   *************
400   VIDEAGARARLMPVSKRKKTVNVADIESVVARIARIPEKSVSQSDRDTLK
      *******
450   NLGDRLKMLVFGQDKAIEALTEAIKMARAGLGHEHKPVGSFLFAGPTGVG

500   KTEVTVQLSKALGIELLRFDMSEYMERHTVSRLIGAPPGYVGFDQGGLLT

550   DAVIKHPHAVLLLDEIEKAHPDVFNILLQVMDNGTLTDNNGRKADFRNVV

600   LVMTTNAGVRETERKSIGLIHQDNSTDAMEEIKKIFTPEFRNRLDNIIWF

650   DHLSTDVIHQVVDKFIVELQVQLDQKGVSLEVSQEARNWLAEKGYDRAMG

700   ARPMARVIQDNLKKPLANELLFGSLVDGGQVTVALDKEKNELTYGFQSAQ

750   KHKAEAAH
```

sp|P39317|YTFI_ECOLI Uncharacterized protein YtfI OS=Escherichia coli (strain K12) OX=83333 GN=ytfI PE=4 SV=2

```
0     MLPRIRHNNFIGAVELFVKSSYTKTHSNNFFNNIHHAFKKKDWISNYDSL

50    LTLREFFRCATQIDKSGYQVLSSKNETVHAMDKFLISFSLKDNGAEYTMT
```

```
100   LRGSGFEYEEIPITINEYNSFMDFKNREFPLEQNRRLYAWDILQKKQSDI

150   PKRIKGYIHQAIGDVSLGYALLEDIVSKLKRGKFELQIPGGGIKECDGWY

200   IYEKIIDDNFAIVIESLGFALKIYGGDERFRNGSSVVLEDEDYSLIYNFL
                        ********************************
250   VNAGCQQVELAEQVDAIVSANLAADSDITKEKICEKYKSTIEAFKKEQLA

300   LPVLVRRKNSET
```

sp|P19323|FHLA_ECOLI Formate hydrogenlyase transcriptional activator FhlA OS=Escherichia coli (strain K12) OX=83333 GN=fhlA PE=1 SV=1

```
0     MSYTPMSDLGQQGLFDITRTLLQQPDLASLCEALSQLVKRSALADNAAIV

50    LWQAQTQRASYYASREKDTPIKYEDETVLAHGPVRSILSRPDTLHCSYEE

100   FCETWPQLDAGGLYPKFGHYCLMPLAAEGHIFGGCEFIRYDDRPWSEKEF

150   NRLQTFTQIVSVVTEQIQSRVVNNVDYELLCRERDNFRILVAITNAVLSR

200   LDMDELVSEVAKEIHYYFDIDDISIVLRSHRKNKLNIYSTHYLDKQHPAH
      ********************
250   EQSEVDEAGTLTERVFKSKEMLLINLHERDDLAPYERMLFDTWGNQIQTL

300   CLLPLMSGDTMLGVLKLAQCEEKVFTTTNLNLLRQIAERVAIAVDNALAY

350   QEIHRLKERLVDENLALTEQLNNVDSEFGEIIGRSEAMYSVLKQVEMVAQ

400   SDSTVLILGETGTGKELIARAIHNLSGRNNRRMVKMNCAAMPAGLLESDL

450   FGHERGAFTGASAQRIGRFELADKSSLFLDEVGDMPLELQPKLLRVLQEQ

500   EFERLGSNKIIQTDVRLIAATNRDLKKMVADREFRSDLYYRLNVFPIHLP

550   PLRERPEDIPLLAKAFTFKIARRLGRNIDSIPAETLRTLSNMEWPGNVRE

600   LENVIERAVLLTRGNVLQLSLPDIVLPEPETPPAATVVALEGEDEYQLIV

650   RVLKETNGVVAGPKGAAQRLGLKRTTLLSRMKRLGIDKSALI
```

sp|P60240|RAPA_ECOLI RNA polymerase-associated protein RapA OS=Escherichia coli (strain K12) OX=83333 GN=rapA PE=1 SV=2

```
0     MPFTLGQRWISDTESELGLGTVVAVDARTVTLLFPSTGENRLYARSDSPV

50    TRVMFNPGDTITSHDGWQMQVEEVKEENGLLTYIGTRLDTEESGVALREV

100   FLDSKLVFSKPQDRLFAGQIDRMDRFALRYRARKYSSEQFRMPYSGLRGQ

150   RTSLIPHQLNIAHDVGRRHAPRVLLADEVGLGKTIEAGMILHQQLLSGAA

200   ERVLIIVPETLQHQWLVEMLRRFNLRFALFDDERYAEAQHDAYNPFDTEQ
```

250   LVICSLDFARRSKQRLEHLCEAEWDLLVVDEAHHLVWSEDAPSREYQAIE

300   QLAEHVPGVLLLTATPEQLGMESHFARLRLLDPNRFHDFAQFVEEQKNYR

350   PVADAVAMLLAGNKLSNDELNMLGEMIGEQDIEPLLQAANSDSEDAQSAR

400   QELVSMLMDRHGTSRVLFRNTRNGVKGFPKRELHTIKLPLPTQYQTAIKV

450   SGIMGARKSAEDRARDMLYPERIYQEFEGDNATWWNFDPRVEWLMGYLTS

500   HRSQKVLVICAKAATALQLEQVLREREGIRAAVFHEGMSIIERDRAAAWF

550   AEEDTGAQVLLCSEIGSEGRNFQFASHMVMFDLPFNPDLLEQRIGRLDRI

600   GQAHDIQIHVPYLEKTAQSVLVRWYHEGLDAFEHTCPTGRTIYDSVYNDL
****
650   INYLASPDQTEGFDDLIKNCREQHEALKAQLEQGRDRLLEIHSNGGEKAQ
****
700   ALAESIEEQDDDTNLIAFAMNLFDIIGINQDDRGDNMIVLTPSDHMLVPD

750   FPGLSEDGITITFDREVALAREDAQFITWEHPLIRNGLDLILSGDTGSST

800   ISLLKNKALPVGTLLVELIYVVEAQAPKQLQLNRFLPPTPVRMLLDKNGN

850   NLAAQVEFETFNRQLNAVNRHTGSKLVNAVQQDVHAILQLGEAQIEKSAR

900   ALIDAARNEADEKLSAELSRLEALRAVNPNIRDDELTAIESNRQQVMESL

950   DQAGWRLDALRLIVVTHQ

sp|P76206|YDIY_ECOLI Uncharacterized protein YdiY OS=Escherichia coli (strain K12) OX=83333 GN=ydiY PE=3 SV=1

0   MKLLKTVPAIVMLAGGMFASLNAAADDSVFTVMDDPASAKKPFEGNLNAG
****
50   YLAQSGNTKSSSLTADTTMTWYGHTTAWSLWGNASNTSSNDERSSEKYAA
****
100   GGRSRFNLTDYDYLFGQASWLTDRYNGYRERDVLTAGYGRQFLNGPVHSF

150   RFEFGPGVRYDKYTDNASETQPLGYASGAYAWQLTDNAKFTQGVSVFGAE

200   DTTLNSESALNVAINEHFGLKVAYNVTWNSEPPESAPEHTDRRTTLSLGY

250   SM

sp|P09394|GLPQ_ECOLI Glycerophosphodiester phosphodiesterase, periplasmic OS=Escherichia coli (strain K12) OX=83333 GN=glpQ PE=1 SV=2

0   MKLTLKNLSMAIMMSTIVMGSSAMAADSNEKIVIAHRGASGYLPEHTLPA

50   KAMAYAQGADYLEQDLVMTKDDNLVVLHDHYLDRVTDVADRFPDRARKDG
****
100   RYYAIDFTLDEIKSLKFTEGFDIENGKKVQTYPGRFPMGKSDFRVHTFEE

```
150   EIEFVQGLNHSTGKNIGIYPEIKAPWFHHQEGKDIAAKTLEVLKKYGYTG

200   KDDKVYLQCFDADELKRIKNELEPKMGMELNLVQLIAYTDWNETQQKQPD

250   GSWVNYNYDWMFKPGAMKQVAEYADGIGPDYHMLIEETSQPGNIKLTGMV

300   QDAQQNKLVVHPYTVRSDKLPEYTPDVNQLYDALYNKAGVNGLFTDFPDK

350   AVKFLNKE

sp|P0AEU7|SKP_ECOLI Chaperone protein Skp OS=Escherichia coli (strain
K12) OX=83333 GN=skp PE=1 SV=1

0     MKKWLLAAGLGLALATSAQAADKIAIVNMGSLFQQVAQKTGVSNTLENEF
                     ***********************
50    KGRASELQRMETDLQAKMKKLQSMKAGSDRTKLEKDVMAQRQTFAQKAQA

100   FEQDRARRSNEERGKLVTRIQTAVKSVANSQDIDLVVDANAVAYNSSDVK

150   DITADVLKQVK

sp|P31069|KCH_ECOLI Voltage-gated potassium channel Kch OS=Escherichia
coli (strain K12) OX=83333 GN=kch PE=1 SV=1

0     MSHWATFKQTATNLWVTLRHDILALAVFLNGLLIFKTIYGMSVNLLDIFH

50    IKAFSELDLSLLANAPLFMLGVFLVLNSIGLLFRAKLAWAISIILLLIAL

100   IYTLHFYPWLKFSIGFCIFTLVFLLILRKDFSHSSAAAGTIFAFISFTTL

150   LFYSTYGALYLSEGFNPRIESLMTAFYFSIETMSTVGYGDIVPVSESARL

200   FTISVIISGITVFATSMTSIFGPLIRGGFNKLVKGNNHTMHRKDHFIVCG

250   HSILAINTILQLNQRGQNVTVISNLPEDDIKQLEQRLGDNADVIPGDSND
                        ********************************
300   SSVLKKAGIDRCRAILALSDNDADNAFVVLSAKDMSSDVKTVLAVSDSKN
      *****                                     ********
350   LNKIKMVHPDIILSPQLFGSEILARVLNGEEINNDMLVSMLLNSGHGIFS
      *****************
400   DNDELETKADSKESAQK

sp|P51024|YAIL_ECOLI Uncharacterized protein YaiL OS=Escherichia coli
(strain K12) OX=83333 GN=yaiL PE=4 SV=2
                                               *********
0     MAKLTLQEQLLKAGLVTSKKAAKVERTAKKSRVQAREARAAVEENKKAQL
      ************
50    ERDKQLSEQQKQAALAKEYKAQVKQLIEMNRITIANGDIGFNFTDGNLIK

100   KIFVDKLTQAQLINGRLAIARLLVDNNSEGEYAIIPASVADKIAQRDASS
        ***************************
150   IVLHSALSAEEQDEDDPYADFKVPDDLMW
```

sp|P0DMC5|RCSC_ECOLI Sensor histidine kinase RcsC OS=Escherichia coli (strain K12) OX=83333 GN=rcsC PE=1 SV=1

0 MKYLASFRTTLKASRYMFRALALVLWLLIAFSSVFYIVNALHQRESEIRQ

50 EFNLSSDQAQRFIQRTSDVMKELKYIAENRLSAENGVLSPRGRETQADVP

100 AFEPLFADSDCSAMSNTWRGSLESLAWFMRYWRDNFSAAYDLNRVFLIGS

150 DNLCMANFGLRDMPVERDTALKALHERINKYRNAPQDDSGSNLYWISEGP

200 RPGVGYFYALTPVYLANRLQALLGVEQTIRMENFFLPGTLPMGVTILDEN

250 GHTLISLTGPESKIKGDPRWMQERSWFGYTEGFRELVLKKNLPPSSLSIV

300 YSVPVDKVLERIRMLILNAILLNVLAGAALFTLARMYERRIFIPAESDAL

350 RLEEHEQFNRKIVASAPVGICILRTADGVNILSNELAHTYLNMLTHEDRQ

400 RLTQIICGQQVNFVDVLTSNNTNLQISFVHSRYRNENVAICVLVDVSSRV

450 KMEESLQEMAQAAEQASQSKSMFLATVSHELRTPLYGIIGNLDLLQTKEL
****
500 PKGVDRLVTAMNNSSSLLLKIISDILDFSKIESEQLKIEPREFSPREVMN

550 HITANYLPLVVRKQLGLYCFIEPDVPVALNGDPMRLQQVISNLLSNAIKF

600 TDTGCIVLHVRADGDYLSIRVRDTGVGIPAKEVVRLFDPFFQVGTGVQRN

650 FQGTGLGLAICEKLISMMDGDISVDSEPGMGSQFTVRIPLYGAQYPQKKG

700 VEGLSGKRCWLAVRNASLCQFLETSLQRSGIVVTTYEGQEPTPEDVLITD

750 EVVSKKWQGRAVVTFCRRHIGIPLEKAPGEWVHSVAAPHELPALLARIYL
****
800 IEMESDDPANALPSTDKAVSDNDDMMILVVDDHPINRRLLADQLGSLGYQ

850 CKTANDGVDALNVLSKNHIDIVLSDVNMPNMDGYRLTQRIRQLGLTLPVI

900 GVTANALAEEKQRCLESGMDSCLSKPVTLDVIKQTLTLYAERVRKSRDS

sp|P21866|KDPE_ECOLI KDP operon transcriptional regulatory protein KdpE OS=Escherichia coli (strain K12) OX=83333 GN=kdpE PE=1 SV=3

0 MTNVLIVEDEQAIRRFLRTALEGDGMRVFEAETLQRGLLEAATRKPDLII
****
50 LDLGLPDGDGIEFIRDLRQWSAVPVIVLSARSEESDKIAALDAGADDYLS
**
100 KPFGIGELQARLRVALRRHSATTAPDPLVKFSDVTVDLAARVIHRGEEEV

150 HLTPIEFRLLAVLLNNAGKVLTQRQLLNQVWGPNAVEHSHYLRIYMGHLR

200 QKLEQDPARPRHFITETGIGYRFML

sp|P75839|YCAP_ECOLI UPF0702 transmembrane protein YcaP OS=Escherichia coli (strain K12) OX=83333 GN=ycaP PE=3 SV=1

```
0     MKAFDLHRMAFDKVPFDFLGEVALRSLYTFVLVFLFLKMTGRRGVRQMSL
                                                     ***
50    FEVLIILTLGSAAGDVAFYDDVPMVPVLIVFITLALLYRLVMWLMAHSEK
      *****************
100   LEDLLEGKPVVIIEDGELAWSKLNNSNMTEFEFFMELRLRGVEQLGQVRL

150   AILETNGQISVYFFEDDKVKPGLLILPSDCTQRYKVVPESADYACIRCSE

200   IIHMKAGEKQLCPRCANPEWTKASRAKRVT
```

sp|P13656|CHIA_ECOLI Probable bifunctional chitinase/lysozyme OS=Escherichia coli (strain K12) OX=83333 GN=chiA PE=1 SV=2

```
0     MKLNIFTKSMIGMGLVCSALPALAMEAWNNQQGGNKYQVIFDGKIYENAW
                                  **********************
50    WVSSTNCPGKAKANDATNPWRLKRTATAAEISQFGNTLSCEKSGSSSSSN
      **********
100   SNTPASNTPANGGSATPAQGTVPSNSSVVAWNKQQGGQTWYVVFNGAVYK

150   NAWWVASSNCPGDAKSNDASNPWRYVRAATATEISETSNPQSCTSAPQPS

200   PDVKPAPDVKPAPDVQPAPADKSNDNYAVVAWKGQEGSSTWYVIYNGGIY

250   KNAWWVGAANCPGDAKENDASNPWRYVRAATATEISQYGNPGSCSVKPDN

300   NGGAVTPVDPTPETPVTPTPDNSEPSTPADSVNDYSLQAWSGQEGSEIYH

350   VIFNGNVYKNAWWVGSKDCPRGTSAENSNNPWRLERTATAAELSQYGNPT

400   TCEIDNGGVIVADGFQASKAYSADSIVDYNDAHYKTSVDQDAWGFVPGGD

450   NPWKKYEPAKAWSASTVYVKGDRVVVDGQAYEALFWTQSDNPALVANQNA

500   TGSNSRPWKPLGKAQSYSNEELNNAPQFNPETLYASDTLIRFNGVNYISQ

550   SKVQKVSPSDSNPWRVFVDWTGTKERVGTPKKAWPKHVYAPYVDFTLNTI

600   PDLAALAKNHNVNHFTLAFVVSKDANTCLPTWGTAYGMQNYAQYSKIKAL

650   REAGGDVMLSIGGANNAPLAASCKNVDDLMQHYYDIVDNLNLKVLDFDIE
                ********************
700   GTWVADQASIERRNLAVKKVQDKWKSEGKDIAIWYTLPILPTGLTPEGMN

750   VLSDAKAKGVELAGVNVMTMDYGNAICQSANTEGQNIHGKCATSAIANLH

800   SQLKGLHPNKSDAEIDAMMGTTPMVGVNDVQGEVFYLSDARLVMQDAQKR

850   NLGMVGIWSIARDLPGGTNLSPEFHGLTKEQAPKYAFSEIFAPFTKQ
```

sp|P52127|YFJL_ECOLI Uncharacterized protein YfjL OS=Escherichia coli (strain K12) OX=83333 GN=yfjL PE=4 SV=1

```
0     MESNDSGGVAAKHGFLFQDCVAAYHVTRMLRDKTIRSVRCEVTDDIDIVS
                                 ********************
50    DGYIDFVQVKSTGKTRWNISDIVQNSKGADKKTIPCSSILHKSMQCESDL

100   SLGRRYSIVTEEKVNKTLEYLTISPNARLDKPGRQELIDDLNKRTDNFLT

150   DSGISVSDWIDAATWEVFSSLRELELLGIKNIRLASQDLHGVILSSETVA

200   EDIWCRILDTVTRKGEHSRRIHSADDKSYLRPDLLEWFKQRVEDDQSRSG

250   RKIYVKRDLPHILTPFRAPMASVCAKRKGQVLHQQYSLKKYRYKHIADNV
                            *********************
300   CQWLDEVFLRPKEMSDIHKLTFIEKRERLKNSVFKSLHDVSEFLGRVLLH

350   ATIRQHHESQPIPCMLYVEKAGAEKILENVHIVRRDPEGDQLWIGFSELV
                      **********************
400   TDINIAVRLPEIRDQLYEDISDCIDTARKKILDIKDDNYLLRHDIDEILD
                          ********************
450   GSQPFDAHLDRFTFVLFVGYDSNLLTEPETPGFEDDLEKETAVLFEKFAA

500   DLIEDSPFANLCIHVFIYPAPSLERLTQLVDEKVREVV
```

sp|P0C0V0|DEGP_ECOLI Periplasmic serine endoprotease DegP OS=Escherichia coli (strain K12) OX=83333 GN=degP PE=1 SV=1

```
0     MKKTTLALSALALSLGLALSPLSATAAETSSATTAQQMPSLAPMLEKVMP

50    SVVSINVEGSTTVNTPRMPRNFQQFFGDDSPFCQEGSPFQSSPFCQGGQG

100   GNGGGQQQKFMALGSGVIIDADKGYVVTNNHVVDNATVIKVQLSDGRKFD

150   AKMVGKDPRSDIALIQIQNPKNLTAIKMADSDALRVGDYTVAIGNPFGLG

200   ETVTSGIVSALGRSGLNAENYENFIQTDAAINRGNSGGALVNLNGELIGI

250   NTAILAPDGGNIGIGFAIPSNMVKNLTSQMVEYGQVKRGELGIMGTELNS

300   ELAKAMKVDAQRGAFVSQVLPNSSAAKAGIKAGDVITSLNGKPISSFAAL
                                              ************
350   RAQVGTMPVGSKLTLGLLRDGKQVNVNLELQQSSQNQVDSSSIFNGIEGA
      ********
400   EMSNKGKDQGVVVNNVKTGTPAAQIGLKKGDVIIGANQQAVKNIAELRKV

450   LDSKPSVLALNIQRGDSTIYLLMQ
```

sp|P32690|YJBI_ECOLI Putative protein YjbI OS=Escherichia coli (strain K12) OX=83333 GN=yjbI PE=5 SV=1

```
0     MKKIECACNFLMDKDAQGYIDLSDLDLTSCHFKGDVISKVSFLSSNLQHV

50    TFECKEIGDCNFTTAIVDNVIFRCRRLHNVIFIKASGECVDFSKNILDTV

100   DFSQSQLGHSNFRECQIRNSNFDNCYLYASHFTRAEFLSAKEISFIKSNL
```

```
150   TAVMFDYVRMSTGNFKDCITEQLELTIDYSDIFWNEDLDGYINNIIKMID
                                                  ******
200   TLPDNAMILKSVLAVKLVMQLKILNIVNKNFIENMKKIFSHCPYIKDPII
      ***************
250   RSYIHSDEDNKFDDFMRQHRFSEVNFDTQQMIDFINRFNTNKWLIDKNNN

300   FFIQLIDQALRSTDDMIKANVWHLYKEWIRSDDVSPIFIETEDNLRTFNT

350   NELTRNDNIFILFSSVDDGPVMVVSSQRLHDMLNPTKDTNWNSTYIYKSR

400   HEMLPVNLTQETLFSSKSHGKYALFPIFTASWRAHRIMNKGV
```

sp|P00370|DHE4_ECOLI NADP-specific glutamate dehydrogenase OS=Escherichia coli (strain K12) OX=83333 GN=gdhA PE=1 SV=1

```
0     MDQTYSLESFLNHVQKRDPNQTEFAQAVREVMTTLWPFLEQNPKYRQMSL

50    LERLVEPERVIQFRVVWVDDRNQIQVNRAWRVQFSSAIGPYKGGMRFHPS

100   VNLSILKFLGFEQTFKNALTTLPMGGGKGGSDFDPKGKSEGEVMRFCQAL

150   MTELYRHLGADTDVPAGDIGVGGREVGFMAGMMKKLSNNTACVFTGKGLS

200   FGGSLIRPEATGYGLVYFTEAMLKRHGMGFEGMRVSVSGSGNVAQYAIEK
              ***********************
250   AMEFGARVITASDSSGTVVDESGFTKEKLARLIEIKASRDGRVADYAKEF

300   GLVYLEGQQPWSLPVDIALPCATQNELDVDAAHQLIANGVKAVAEGANMP

350   TTIEATELFQQAGVLFAPGKAANAGGVATSGLEMAQNAARLGWKAEKVDA

400   RLHHIMLDIHHACVEHGGEGEQTNYVQGANIAGFVKVADAMLAQGVI
```

sp|P32704|YJCF_ECOLI Uncharacterized protein YjcF OS=Escherichia coli (strain K12) OX=83333 GN=yjcF PE=4 SV=1

```
                       *******************************
0     MRYNGLNNMFFPLCLINDNHSVTSPSHTKKTKSDNYSKHHKNTLIDNKAL

50    SLFKMDDHEKVIGLIQKMKRIYDSLPSGKITKETDRKIHKYFIDIASHAN
                                                   ***
100   NKCDDRITRRVYLNKDKEVSIKVVYFINNVTVHNNTIEIPQTVNGGYDFS
      *****************
150   HLSLKGIVIKDEDLSNSNFAGCRLQNAIFQDCNMYKTNFNFAIMEKILFD

200   NCILDDSNFAQIKMTDGTLNSCSAMHVQFYNATMNRANIKNTFLDYSNFY

250   MAYMAEVNLYKVIAPYINLFRADLSFSKLDLINFEHADLSRVNLNKATLQ

300   NINLIDSKLFFTRLTNTFLEMVICTDSNMANVNFNNANLSNCHFNCSVLT

350   KAWMFNIRLYRVNFDEASVQGMGITILRGEENISINSDILVTLQKFFEED

400   CATHTGMSQTEDNLHAVAMKITADIMQDAD
```

sp|P42616|YQJC_ECOLI Protein YqjC OS=Escherichia coli (strain K12) OX=83333 GN=yqjC PE=1 SV=3

```
0     MKYRIALAVSLFALSAGSYATTLCQEKEQNILKEISYAEKHQNQNRIDGL
                ****************** ***     **************
50    NKALSEVRANCSDSQLRADHQKKIAKQKDEVAERQQDLAEAKQKGDADKI
      *********************
100   AKRERKLAEAQEELKKLEARDY
```

sp|P76192|YDHV_ECOLI Uncharacterized oxidoreductase YdhV OS=Escherichia coli (strain K12) OX=83333 GN=ydhV PE=2 SV=1

```
0     MANGWTGNILRVNLTTGNITLEDSSKFKSFVGGMGFGYKIMYDEVPPGTK

50    PFDEANKLVFATGPLTGSGAPCSSRVNITSLSTFTKGNLVVDAHMGGFFA
                 ********************************
100   AQMKFAGYDVIIIEGKAKSPVWLKIKDDKVSLEKADFLWGKGTRATTEEI

150   CRLTSPETCVAAIGQAGENLVPLSGMLNSRNHSGGAGTGAIMGSKNLKAI

200   AVEGTKGVNIADRQEMKRLNDYMMTELIGANNNHVVPSTPQSWAEYSDPK

250   SRWTARKGLFWGAAEGGPIETGEIPPGNQNTVGFRTYKSVFDLGPAAEKY

300   TVKMSGCHSCPIRCMTQMNIPRVKEFGVPSTGGNTCVANFVHTTIFPNGP

350   KDFEDKDDGRVIGNLVGLNLFDDYGLWCNYGQLHRDFTYCYSKGVFKRVL

400   PAEEYAEIRWDQLEAGDVNFIKDFYYRLAHRVGELSHLADGSYAIAERWN

450   LGEEYWGYAKNKLWSPFGYPVHHANEASAQVGSIVNCMFNRDCMTHTHIN

500   FIGSGLPLKLQREVAKELFGSEDAYDETKNYTPINDAKIKYAKWSLLRVC

550   LHNAVTLCNWVWPMTVSPLKSRNYRGDLALEAKFFKAITGEEMTQEKLDL

600   AAERIFTLHRAYTVKLMQTKDMRNEHDLICSWVFDKDPQIPVFTEGTDKM

650   DRDDMHASLTMFYKEMGWDPQLGCPTRETLQRLGLEDIAADLAAHNLLPA

700
```

sp|Q47146|FADE_ECOLI Acyl-coenzyme A dehydrogenase OS=Escherichia coli (strain K12) OX=83333 GN=fadE PE=2 SV=2

```
0     MMILSILATVVLLGALFYHRVSLFISSLILLAWTAALGVAGLWSAWVLVP

50    LAIILVPFNFAPMRKSMISAPVFRGFRKVMPPMSRTEKEAIDAGTTWWEG

100   DLFQGKPDWKKLHNYPQPRLTAEEQAFLDGPVEEACRMANDFQITHELAD

150   LPPELWAYLKEHRFFAMIIKKEYGGLEFSAYAQSRVLQKLSGVSGILAIT
```

200   VGVPNSLGPGELLQHYGTDEQKDHYLPRLARGQEIPCFALTSPEAGSDAG

250   AIPDTGIVCMGEWQGQQVLGMRLTWNKRYITLAPIATVLGLAFKLSDPEK

300   LLGGAEDLGITCALIPTTTPGVEIGRRHFPLNVPFQNGPTRGKDVFVPID

350   YIIGGPKMAGQGWRMLVECLSVGRGITLPSNSTGGVKSVALATGAYAHIR

400   RQFKISIGKMEGIEEPLARIAGNAYVMDAAASLITYGIMLGEKPAVLSAI

450   VKYHCTHRGQQSIIDAMDITGGKGIMLGQSNFLARAYQGAPIAITVEGAN

500   ILTRSMMIFGQGAIRCHPYVLEEMEAAKNNDVNAFDKLLFKHIGHVGSNK

550   VRSFWLGLTRGLTSSTPTGDATKRYYQHLNRLSANLALLSDVSMAVLGGS

600   LKRRERISARLGDILSQLYLASAVLKRYDDEGRNEADLPLVHWGVQDALY

650   QAEQAMDDLLQNFPNRVVAGLLNVVIFPTGRHYLAPSDKLDHKVAKILQV

700   PNATRSRIGRGQYLTPSEHNPVGLLEEALVDVIAADPIHQRICKELGKNL
****

750   PFTRLDELAHNALVKGLIDKDEAAILVKAEESRLRSINVDDFDPEELATK

800   PVKLPEKVRKVEAA

sp|P0A8M0|SYN_ECOLI Asparagine--tRNA ligase OS=Escherichia coli (strain K12) OX=83333 GN=asnS PE=1 SV=2

0   MSVVPVADVLQGRVAVDSEVTVRGWVRTRRDSKAGISFLAVYDGSCFDPV

50   QAVINNSLPNYNEDVLRLTTGCSVIVTGKVVASPGQGQQFEIQASKVEVA

100   GWVEDPDTYPMAAKRHSIEYLREVAHLRPRTNLIGAVARVRHTLAQALHR

150   FFNEQGFFWVSTPLITASDTEGAGEMFRVSTLDLENLPRNDQGKVDFDKD

200   FFGKESFLTVSGQLNGETYACALSKIYTFGPTFRAENSNTSRHLAEFWML
****

250   EPEVAFANLNDIAGLAEAMLKYVFKAVLEERADDMKFFAERVDKDAVSRL

300   ERFIEADFAQVDYTDAVTILENCGRKFENPVYWGVDLSSEHERYLAEEHF

350   KAPVVVKNYPKDIKAFYMRLNEDGKTVAAMDVLAPGIGEIIGGSQREERL

400   DVLDERMLEMGLNKEDYWWYRDLRRYGTVPHSGFGLGFERLIAYVTGVQN

450   VRDVIPFPRTPRNASF

sp|P02929|TONB_ECOLI Protein TonB OS=Escherichia coli (strain K12) OX=83333 GN=tonB PE=1 SV=2

0   MTLDLPRRFPWPTLLSVCIHGAVVAGLLYTSVHQVIELPAPAQPISVTMV
****

50   TPADLEPPQAVQPPPEPVVEPEPEPEPIPEPPKEAPVVIEKPKPKPKPKP
      ****
100   KPVKKVQEQPKRDVKPVESRPASPFENTAPARLTSSTATAATSKPVTSVA

150   SGPRALSRNQPQYPARAQALRIEGQVKVKFDVTPDGRVDNVQILSAKPAN

200   MFEREVKNAMRRWRYEPGKPGSGIVVNILFKINGTTEIQ

sp|P15640|PUR2_ECOLI Phosphoribosylamine--glycine ligase OS=Escherichia coli (strain K12) OX=83333 GN=purD PE=1 SV=2

0   MKVLVIGNGGREHALAWKAAQSPLVETVFVAPGNAGTALEPALQNVAIGV

50   TDIPALLDFAQNEKIDLTIVGPEAPLVKGVVDTFRAAGLKIFGPTAGAAQ

100   LEGSKAFTKDFLARHKIPTAEYQNFTEVEPALAYLREKGAPIVIKADGLA

150   AGKGVIVAMTLEEAEAAVHDMLAGNAFGDAGHRIVIEEFLDGEEASFIVM

200   VDGEHVLPMATSQDHKRVGDKDTGPNTGGMGAYSPAPVVTDDVHQRTMER

250   IIWPTVKGMAAEGNTYTGFLYAGLMIDKQGNPKVIEFNCRFGDPETQPIM
        ****
300   LRMKSDLVELCLAACESKLDEKTSEWDERASLGVVMAAGGYPGDYRTGDV

350   IHGLPLEEVAGGKVFHAGTKLADDEQVVTNGGRVLCVTALGHTVAEAQKR

400   AYALMTDIHWDDCFCRKDIGWRAIEREQN

sp|P39358|YJHG_ECOLI D-xylonate dehydratase YjhG OS=Escherichia coli (strain K12) OX=83333 GN=yjhG PE=1 SV=2

0   MSVRNIFADESHDIYTVRTHADGPDGELPLTAEMLINRPSGDLFGMTMNA

50   GMGWSPDELDRDGILLLSTLGGLRGADGKPVALALHQGHYELDIQMKAAA

100   EVIKANHALPYAVYVSDPCDGRTQGTTGMFDSLPYRNDASMVMRRLIRSL

150   PDAKAVIGVASCDKGLPATMMALAAQHNIATVLVPGGATLPAKDGEDNGK

200   VQTIGARFANGELSLQDARRAGCKACASSGGGCQFLGTAGTSQVVAEGLG

250   LAIPHSALAPSGEPVWREIARASARAALNLSQKGITTREILTDKAIENAM

300   TVHAAFGGSTNLLLHIPAIAHQAGCHIPTVDDWIRINKRVPRLVSVLPNG

350   PVYHPTVNAFMAGGVPEVMLHLRSLGLLHEDVMTVTGSTLKENLDWWEHS

400   ERRQRFKQLLLDQEQINADEVIMSPQQAKARGLTSTITFPVGNIAPEGSV
                            ****
450   IKSTAIDPSMIDEQGIYYHKGVAKVYLSEKSAIYDIKHDKIKAGDILVII

500   GVGPSGTGMEETYQVTSALKHLSYGKHVSLITDARFSGVSTGACIGHVGP

```
550   EALAGGPIGKLRTGDLIEIKIDCRELHGEVNFLGTRSDEQLPSQEEATAI

600   LNARPSHQDLLPDPELPDDTRLWAMLQAVSGGTWTGCIYDVNKIGAALRD

650   FMNKN

sp|Q2M7X4|YICS_ECOLI Uncharacterized protein YicS OS=Escherichia coli
(strain K12) OX=83333 GN=yicS PE=4 SV=1
                                                     ***
0     MKPTTLLLIFTFFAMPGIVYAESPFSSLQSAKEKTTVLQDLRKICTPQAS
      *******************
50    LSDEAWEKLMLSDENNKQHIREAIVAMERNNQSNYWEALGKVECPDM

sp|P0ABU0|MENB_ECOLI 1,4-dihydroxy-2-naphthoyl-CoA synthase
OS=Escherichia coli (strain K12) OX=83333 GN=menB PE=1 SV=1
                  ************************
0     MIYPDEAMLYAPVEWHDCSEGFEDIRYEKSTDGIAKITINRPQVRNAFRP

50    LTVKEMIQALADARYDDNIGVIILTGAGDKAFCSGGDQKVRGDYGGYKDD

100   SGVHHLNVLDFQRQIRTCPKPVVAMVAGYSIGGGHVLHMMCDLTIAADNA

150   IFGQTGPKVGSFDGGWGASYMARIVGQKKAREIWFLCRQYDAKQALDMGL

200   VNTVVPLADLEKETVRWCREMLQNSPMALRCLKAALNADCDGQAGLQELA

250   GNATMLFYMTEEGQEGRNAFNQKRQPDFSKFKRNP

sp|P77804|YDGA_ECOLI Protein YdgA OS=Escherichia coli (strain K12)
OX=83333 GN=ydgA PE=1 SV=1

0     MNKSLVAVGVIVALGVVWTGGAWYTGKKIETHLEDMVAQANAQLKLTAPE

50    SNLEVSYQNYHRGVFSSQLQLLVKPIAGKENPWIKSGQSVIFNESVDHGP
                                              **********
100   FPLAQLKKLNLIPSMASIQTTLVNNEVSKPLFDMAKGETPFEINSRIGYS
      ************************
150   GDSSSDISLKPLNYEQKDEKVAFSGGEFQLNADRDGKAISLSGEAQSGRI
                                                  ******
200   DAVNEYNQKVQLTFNNLKTDGSSTLASFGERVGNQKLSLEKMTISVEGKE
      **************
250   LALLEGMEISGKSDLVNDGKTINSQLDYSLNSLKVQNQDLGSGKLTLKVG

300   QIDGEAWHQFSQQYNAQTQALLAQPEIANNPELYQEKVTEAFFSALPLML

350   KGDPVITIAPLSWKNSQGESALNLSLFLKDPATTKEAPQTLAQEVDRSVK

400   SLDAKLTIPVDMATEFMTQVAKLEGYQEDQAKKLAKQQVEGASAMGQMFR

450   LTTLQDNTITTSLQYANGQITLNGQKMSLEDFVGMFAMPALNVPAVPAIP

500   QQ
```

sp|P0AC53|G6PD_ECOLI Glucose-6-phosphate 1-dehydrogenase OS=Escherichia coli (strain K12) OX=83333 GN=zwf PE=1 SV=1

0   MAVTQTAQACDLVIFGAKGDLARRKLLPSLYQLEKAGQLNPDTRIIGVGR

50   ADWDKAAYTKVVREALETFMKETIDEGLWDTLSARLDFCNLDVNDTAAFS

100   RLGAMLDQKNRITINYFAMPPSTFGAICKGLGEAKLNAKPARVVMEKPLG

150   TSLATSQEINDQVGEYFEECQVYRIDHYLGKETVLNLLALRFANSLFVNN
****
200   WDNRTIDHVEITVAEEVGIEGRWGYFDKAGQMRDMIQNHLLQILCMIAMS
****
250   PPSDLSADSIRDEKVKVLKSLRRIDRSNVREKTVRGQYTAGFAQGKKVPG

300   YLEEEGANKSSNTETFVAIRVDIDNWRWAGVPFYLRTGKRLPTKCSEVVV

350   YFKTPELNLFKESWQDLPQNKLTIRLQPDEGVDIQVLNKVPGLDHKHNLQ

400   ITKLDLSYSETFNQTHLADAYERLLLETMRGIQALFVRRDEVEEAWKWVD

450   SITEAWAMDNDAPKPYQAGTWGPVASVAMITRDGRSWNEFE

sp|P37329|MODA_ECOLI Molybdate-binding protein ModA OS=Escherichia coli (strain K12) OX=83333 GN=modA PE=1 SV=1
******
0   MARKWLNLFAGAALSFAVAGNALADEGKITVFAAASLTNAMQDIATQFKK
****
50   EKGVDVVSSFASSSTLARQIEAGAPADLFISADQKWMDYAVDKKAIDTAT

100   RQTLLGNSLVVVAPKASVQKDFTIDSKTNWTSLLNGGRLAVGDPEHVPAG

150   IYAKEALQKLGAWDTLSPKLAPAEDVRGALALVERNEAPLGIVYGSDAVA

200   SKGVKVVATFPEDSHKKVEYPVAVVEGHNNATVKAFYDYLKGPQAAEIFK

250   RYGFTIK

sp|P42915|YRAJ_ECOLI Outer membrane usher protein YraJ OS=Escherichia coli (strain K12) OX=83333 GN=yraJ PE=2 SV=1

0   MPQRHHQGHKRTPKQLALIIKRCLPMVLTGSGMLCTTANAEEYYFDPIML

50   ETTKSGMQTTDLSRFSKKYAQLPGTYQVDIWLNKKKVSQKKITFTANAEQ
****
100   LLQPQFTVEQLRELGIKVDEIPALAEKDDDSVINSLEQIIPGTAAEFDFN

150   HQQLNLSIPQIALYRDARGYVSPSRWDDGIPTLFTNYSFTGSDNRYRQGN

200   RSQRQYLNMQNGANFGPWRLRNYSTWTRNDQTSSWNTISSYLQRDIKALK

250   SQLLLGESATSGSIFSSYTFTGVQLASDDNMLPNSQRGFAPTVRGIANSS

300   AIVTIRQNGYVIYQSNVSAGAFEINDLYPSSNSGDLEVTIEESDGTQRRF

350   IQPYSSLPMMQRPGHLKYSATAGRYRADANSDSKEPEFAEATAIYGLNNT

400   FTLYGGLLGSEDYYALGIGIGGTLGALGALSMDINRADTQFDNQHSFHGY

450   QWRTQYIKDIPETNTNIAVSYYRYTNDGYFSFNEANTRNWDYNSRQKSEI

500   QFNISQTIFDGVSLYASGSQQDYWGNNDKNRNISVGVSGQQWGVGYSLNY

550   QYSRYTDQNNDRALSLNLSIPLERWLPRSRVSYQMTSQKDRPTQHEMRLD

600   GSLLDDGRLSYSLEQSLDDDNNHNSSLNASYRSPYGTFSAGYSYGNDSSQ

650   YNYGVTGGVVIHPHGVTLSQYLGNAFALIDANGASGVRIQNYPGIATDPF

700   GYAVVPYLTTYQENRLSVDTTQLPDNVDLEQTTQFVVPNRGAMVAARFNA

750   NIGYRVLVTVSDRNGKPLPFGALASNDDTGQQSIVDEGGILYLSGISSKS

800   QSWTVRWGNQADQQCQFAFSTPDSEPTTSVLQGTAQCH

sp|P76272|YEBT_ECOLI Intermembrane transport protein YebT OS=Escherichia coli (strain K12) OX=83333 GN=yebT PE=1 SV=2

0   MSQETPASTTEAQIKNKRRISPFWLLPFIALMIASWLIWDSYQDRGNTVT
                                 ****
50   IDFMSADGIVPGRTPVRYQGVEVGTVQDISLSDDLRKIEVKVSIKSDMKD
      ****
100   ALREETQFWLVTPKASLAGVSGLDALVGGNYIGMMPGKGKEQDHFVALDT

150   QPKYRLDNGDLMIHLQAPDLGSLNSGSLVYFRKIPVGKVYDYAINPNKQG

200   VVIDVLIERRFTDLVKKGSRFWNVSGVDANVSISGAKVKLESLAALVNGA
        ****
250   IAFDSPEESKPAEAEDTFGLYEDLAHSQRGVIIKLELPSGAGLTADSTPL

300   MYQGLEVGQLTKLDLNPGGKVTGEMTVDPSVVTLLRENTRIELRNPKLSL

350   SDANLSALLTGKTFELVPGDGEPRKEFVVVPGEKALLHEPDVLTLTLTAP

400   ESYGIDAGQPLILHGVQVGQVIDRKLTSKGVTFTVAIEPQHRELVKGDSK

450   FVVNSRVDVKVGLDGVEFLGASASEWINGGIRILPGDKGEMKASYPLYAN

500   LEKALENSLSDLPTTTVSLSAETLPDVQAGSVVLYRKFEVGEVITVRPRA

550   NAFDIDLHIKPEYRNLLTSNSVFWAEGGAKVQLNGSGLTVQASPLSRALK

600   GAISFDNLSGASASQRKGDKRILYASETAARAVGGQITLHAFDAGKLAVG

650   MPIRYLGIDIGQIQTLDLITARNEVQAKAVLYPEYVQTFARGGTRFSVVT

700   PQISAAGVEHLDTILQPYINVEPGRGNPRRDFELQEATITDSRYLDGLSI

750 IVEAPEAGSLGIGTPVLFRGLEVGTVTGMTLGTLSDRVMIAMRISKRYQH

800 LVRNNSVFWLASGYSLDFGLTGGVVKTGTFNQFIRGGIAFATPPGTPLAP

850 KAQEGKHFLLQESEPKEWREWGTALPK

sp|P67430|NEMR_ECOLI HTH-type transcriptional repressor NemR
OS=Escherichia coli (strain K12) OX=83333 GN=nemR PE=1 SV=1

0 MNKHTEHDTREHLLATGEQLCLQRGFTGMGLSELLKTAEVPKGSFYHYFR

50 SKEAFGVAMLERHYAAYHQRLTELLQSGEGNYRDRILAYYQQTLNQFCQH
****
100 GTISGCLTVKLSAEVCDLSEDMRSAMDKGARGVIALLSQALENGRENHCL

150 TFCGEPLQQAQVLYALWLGANLQAKISRSFEPLENALAHVKNIIATPAV

sp|P17315|CIRA_ECOLI Colicin I receptor OS=Escherichia coli (strain K12)
OX=83333 GN=cirA PE=1 SV=2

0 MFRLNPFVRVGLCLSAISCAWPVLAVDDDGETMVVTASSVEQNLKDAPAS

50 ISVITQEDLQRKPVQNLKDVLKEVPGVQLTNEGDNRKGVSIRGLDSSYTL

100 ILVDGKRVNSRNAVFRHNDFDLNWIPVDSIERIEVVRGPMSSLYGSDALG

150 GVVNIITKKIGQKWSGTVTVDTTIQEHRDRGDTYNGQFFTSGPLIDGVLG

200 MKAYGSLAKREKDDPQNSTTTDTGETPRIEGFSSRDGNVEFAWTPNQNHD
****
250 FTAGYGFDRQDRDSDSLDKNRLERQNYSVSHNGRWDYGTSELKYYGEKVE

300 NKNPGNSSPITSESNTVDGKYTLPLTAINQFLTVGGEWRHDKLSDAVNLT

350 GGTSSKTSASQYALFVEDEWRIFEPLALTTGVRMDDHETYGEHWSPRAYL

400 VYNATDTVTVKGGWATAFKAPSLLQLSPDWTSNSCRGACKIVGSPDLKPE

450 TSESWELGLYYMGEEGWLEGVESSVTVFRNDVKDRISISRTSDVNAAPGY

500 QNFVGFETGANGRRIPVFSYYNVNKARIQGVETELKIPFNDEWKLSINYT

550 YNDGRDVSNGENKPLSDLPFHTANGTLDWKPLALEDWSFYVSGHYTGQKR

600 ADSATAKTPGGYTIWNTGAAWQVTKDVKLRAGVLNLGDKDLSRDDYSYNE

650 DGRRYFMAVDYRF

sp|P0AEF0|DNAC_ECOLI DNA replication protein DnaC OS=Escherichia coli
(strain K12) OX=83333 GN=dnaC PE=1 SV=1

0 MKNVGDLMQRLQKMMPAHIKPAFKTGEELLAWQKEQGAIRSAALERENRA

50 MKMQRTFNRSGIRPLHQNCSFENYRVECEGQMNALSKARQYVEEFDGNIA

100   SFIFSGKPGTGKNHLAAAICNELLLRGKSVLIITVADIMSAMKDTFRNSG
                                 ****
150   TSEEQLLNDLSNVDLLVIDEIGVQTESKYEKVIINQIVDRRSSSKRPTGM

200   LTNSNMEEMTKLLGERVMDRMRLGNSLWVIFNWDSYRSRVTGKEY

sp|P0ABN9|DCUB_ECOLI Anaerobic C4-dicarboxylate transporter DcuB OS=Escherichia coli (strain K12) OX=83333 GN=dcuB PE=1 SV=1

0   MLFTIQLIIILICLFYGARKGGIALGLLGGIGLVILVFVFHLQPGKPPVD

50   VMLVIIAVVAASATLQASGGLDVMLQIAEKLLRRNPKYVSIVAPFVTCTL

100   TILCGTGHVVYTILPIIYDVAIKNNIRPERPMAASSIGAQMGIIASPVSV
                                               ****
150   AVVSLVAMLGNVTFDGRHLEFLDLLAITIPSTLIGILAIGIFSWFRGKDL
      ****
200   DKDEEFQKFISVPENREYVYGDTATLLDKKLPKSNWLAMWIFLGAIAVVA

250   LLGADSDLRPSFGGKPLSMVLVIQMFMLLTGALIIILTKTNPASISKNEV

300   FRSGMIAIVAVYGIAWMAETMFGAHMSEIQGVLGEMVKEYPWAYAIVLLL

350   VSKFVNSQAAALAAIVPVALAIGVDPAYIVASAPACYGYYILPTYPSDLA

400   AIQFDRSGTTHIGRFVINHSFILPGLIGVSVSCVFGWIFAAMYGFL

sp|P77562|YAIW_ECOLI Uncharacterized protein YaiW OS=Escherichia coli (strain K12) OX=83333 GN=yaiW PE=4 SV=1

0   MSRVNPLSSLSLLAVLVLAGCSSQAPQPLKKGEKAIDVASVVRQKMPASV

50   KDRDAWAKDLATTFESQGLAPTLENVCSVLAVAQQESNYQADPAVPGLSK
                              ****
100   IAWQEIDRRAERMHIPAFLVHTALKIKSPNGKSYSERLDSVRTEKQLSAI

150   FDDLINMVPMGQTLFGSLNPVRTGGPMQVSIAFAEQHTKGYPWKMDGTVR

200   QEVFSRRGGLWFGTYHLLNYPASYSAPIYRFADFNAGWYASRNAAFQNAV
                                        ****
250   SKASGVKLALDGDLIRYDSKEPGKTELATRKLAAKLGMSDSEIRRQLEKG
      ****
300   DSFSFEETALYKKVYQLAETKTGKSLPREMLPGIQLESPKITRNLTTAWF

350   AKRVDERRARCMKQ

sp|P0A6I3|COAA_ECOLI Pantothenate kinase OS=Escherichia coli (strain K12) OX=83333 GN=coaA PE=1 SV=1
                                    ****
0   MSIKEQTLMTPYLQFDRNQWAALRDSVPMTLSEDEIARLKGINEDLSLEE
      *
50   VAEIYLPLSRLLNFYISSNLRRQAVLEQFLGTNGQRIPYIISIAGSVAVG

100   KSTTARVLQALLSRWPEHRRVELITTDGFLHPNQVLKERGLMKKKGFPES

150   YDMHRLVKFVSDLKSGVPNVTAPVYSHLIYDVIPDGDKTVVQPDILILEG

200   LNVLQSGMDYPHDPHHVFVSDFVDFSIYVDAPEDLLQTWYINRFLKFREG

250   AFTDPDSYFHNYAKLTKEEAIKTAMTLWKEINWLNLKQNILPTRERASLI

300   LTKSANHAVEEVRLRK

sp|P39293|YJFK_ECOLI Uncharacterized protein YjfK OS=Escherichia coli (strain K12) OX=83333 GN=yjfK PE=4 SV=1

0   MSGFFQRLFGKDNKPAIARGPLGLHLNSGFTLDTLAFRLLEDELLIALPG
                                        ****
50   EEFTVAAVSHIDLGGGSQIFRYYTSGDEFLQINTTGGEDIDDIDDIKLFV
      ****
100   YEESYGISKESHWREAINAKAMGAMTLNWQEKRWQRFFNSEEPGNIEPVY

150   MLEKVENQNHAKWEVHNFTMGYQRQVTEDTYEYLLLNGEESFNDLGEPEW

200   LFSRALGVDIPLTSLHIIG

sp|P00490|PHSM_ECOLI Maltodextrin phosphorylase OS=Escherichia coli (strain K12) OX=83333 GN=malP PE=1 SV=7

0   MSQPIFNDKQFQEALSRQWQRYGLNSAAEMTPRQWWLAVSEALAEMLRAQ

50   PFAKPVANQRHVNYISMEFLIGRLTGNNLLNLGWYQDVQDSLKAYDINLT

100   DLLEEEIDPALGNGGLGRLAACFLDSMATVGQSATGYGLNYQYGLFRQSF

150   VDGKQVEAPDDWHRSNYPWFRHNEALDVQVGIGGKVTKDGRWEPEFTITG

200   QAWDLPVVGYRNGVAQPLRLWQATHAHPFDLTKFNDGDFLRAEQQGINAE

250   KLTKVLYPNDNHTAGKKLRLMQQYFQCACSVADILRRHHLAGRKLHELAD

300   YEVIQLNDTHPTIAIPELLRVLIDEHQMSWDDAWAITSKTFAYTNHTLMP

350   EALERWDVKLVKGLLPRHMQIINEINTRFKTLVEKTWPGDEKVWAKLAVV

400   HDKQVHMANLCVVGGFAVNGVAALHSDLVVKDLFPEYHQLWPNKFHNVTN

450   GITPRRWIKQCNPALAALLDKSLQKEWANDLDQLINLEKFADDAKFRQQY

500   REIKQANKVRLAEFVKVRTGIEINPQAIFDIQIKRLHEYKRQHLNLLHIL

550   ALYKEIRENPQADRVPRVFLFGAKAAPGYYLAKNIIFAINKVADVINNDP

600   LVGDKLKVVFLPDYCVSAAEKLIPAADISEQISTAGKEASGTGNMKLALN
                                              ****
650   GALTVGTLDGANVEIAEKVGEENIFIFGHTVEQVKAILAKGYDPVKWRKK
      ****

700   DKVLDAVLKELESGKYSDGDKHAFDQMLHSIGKQGGDPYLVMADFAAYVE

750   AQKQVDVLYRDQEAWTRAAILNTARCGMFSSDRSIRDYQARIWQAKR

sp|P09373|PFLB_ECOLI Formate acetyltransferase 1 OS=Escherichia coli (strain K12) OX=83333 GN=pflB PE=1 SV=2

0   MSELNEKLATAWEGFTKGDWQNEVNVRDFIQKNYTPYEGDESFLAGATEA

50   TTTLWDKVMEGVKLENRTHAPVDFDTAVASTITSHDAGYINKQLEKIVGL

100   QTEAPLKRALIPFGGIKMIEGSCKAYNRELDPMIKKIFTEYRKTHNQGVF

150   DVYTPDILRCRKSGVLTGLPDAYGRGRIIGDYRRVALYGIDYLMKDKLAQ

200   FTSLQADLENGVNLEQTIRLREEIAEQHRALGQMKEMAAKYGYDISGPAT

250   NAQEAIQWTYFGYLAAVKSQNGAAMSFGRTSTFLDVYIERDLKAGKITEQ

300   EAQEMVDHLVMKLRMVRFLRTPEYDELFSGDPIWATESIGGMGLDGRTLV
****
350   TKNSFRFLNTLYTMGPSPEPNMTILWSEKLPLNFKKFAAKVSIDTSSLQY
****
400   ENDDLMRPDFNNDDYAIACCVSPMIVGKQMQFFGARANLAKTMLYAINGG

450   VDEKLKMQVGPKSEPIKGDVLNYDEVMERMDHFMDWLAKQYITALNIIHY

500   MHDKYSYEASLMALHDRDVIRTMACGIAGLSVAADSLSAIKYAKVKPIRD

550   EDGLAIDFEIEGEYPQFGNNDPRVDDLAVDLVERFMKKIQKLHTYRDAIP

600   TQSVLTITSNVVYGKKTGNTPDGRRAGAPFGPGANPMHGRDQKGAVASLT

650   SVAKLPFAYAKDGISYTFSIVPNALGKDDEVRKTNLAGLMDGYFHHEASI

700   EGGQHLNVNVMNREMLLDAMENPEKYPQLTIRVSGYAVRFNSLTKEQQQD

750   VITRTFTQSM

sp|Q47702|YFEK_ECOLI Uncharacterized protein YfeK OS=Escherichia coli (strain K12) OX=83333 GN=yfeK PE=3 SV=1

0   MKKIICLVITLLMTLPVYAKLTAHEEARINAMLEGLAQKKDLIFVRNGDE
****
50   HTCYEAVSHLRLKLGNTRNRIDTAEQFIDKVASSSSITGKPYIVKIPGKS
**
100   DENAQPFLHALIAQTDKTVPAEGN

sp|P76296|YECT_ECOLI Uncharacterized protein YecT OS=Escherichia coli (strain K12) OX=83333 GN=yecT PE=4 SV=2
****
0   MFKFLVLTLGIISCQAYAEDTVIVNDHDISAIKDCWQKNSDDDTDVNVIK
**
50   SCLRQEYNLVDAQLNKAYGEAYRYIEQVPRTGVKKPDTEQLNLLKKSQRA

****
100   WLDFRDKECELILSNEDVQDLSDPYSESEWLSCMIIQTNTRTRQLQLYRN

150   SEDFYPSPLTRG

sp|P0AB85|APBE_ECOLI FAD:protein FMN transferase OS=Escherichia coli (strain K12) OX=83333 GN=apbE PE=1 SV=1

0   MEISFTRVALLAAALFFVGCDQKPQPAKTHATEVTVLEGKTMGTFWRASI
****
50   PGIDAKRSAELKEKIQTQLDADDQLLSTYKKDSALMRFNDSQSLSPWPVS

100   EAMADIVTTSLRIGAKTDGAMDITVGPLVNLWGFGPEQQPVQIPSQEQID

150   AMKAKTGLQHLTVINQSHQQYLQKDLPDLYVDLSTVGEGYAADHLARLME

200   QEGISRYLVSVGGALNSRGMNGEGLPWRVAIQKPTDKENAVQAVVDINGH

250   GISTSGSYRNYYELDGKRLSHVIDPQTGRPIEHNLVSVTVIAPTALEADA

300   WDTGLMVLGPEKAKEVVRREGLAVYMITKEGDSFKTWMSPQFKSFLVSEK

350   N

sp|P0A7A7|PLSB_ECOLI Glycerol-3-phosphate acyltransferase OS=Escherichia coli (strain K12) OX=83333 GN=plsB PE=1 SV=2

0   MSGWPRIYYKLLNLPLSILVKSKSIPADPAPELGLDTSRPIMYVLPYNSK

50   ADLLTLRAQCLAHDLPDPLEPLEIDGTLLPRYVFIHGGPRVFTYYTPKEE

100   SIKLFHDYLDLHRSNPNLDVQMVPVSVMFGRAPGREKGEVNPPLRMLNGV

150   QKFFAVLWLGRDSFVRFSPSVSLRRMADEHGTDKTIAQKLARVARMHFAR
****
200   QRLAAVGPRLPARQDLFNKLLASRAIAKAVEDEARSKKISHEKAQQNAIA

250   LMEEIAANFSYEMIRLTDRILGFTWNRLYQGINVHNAERVRQLAHDGHEL

300   VYVPCHRSHMDYLLLSYVLYHQGLVPPHIAAGINLNFWPAGPIFRRLGAF

350   FIRRTFKGNKLYSTVFREYLGELFSRGYSVEYFVEGGRSRTGRLLDPKTG

400   TLSMTIQAMLRGGTRPITLIPIYIGYEHVMEVGTYAKELRGATKEKESLP

450   QMLRGLSKLRNLGQGYVNFGEPMPLMTYLNQHVPDWRESIDPIEAVRPAW

500   LTPTVNNIAADLMVRINNAGAANAMNLCCTALLASRQRSLTREQLTEQLN

550   CYLDLMRNVPYSTDSTVPSASASELIDHALQMNKFEVEKDTIGDIIILPR

600   EQAVLMTYYRNNIAHMLVLPSLMAAIVTQHRHISRDVLMEHVNVLYPMLK

650   AELFLRWDRDELPDVIDALANEMQRQGLITLQDDELHINPAHSRTLQLLA

700   AGARETLQRYAITFWLLSANPSINRGTLEKESRTVAQRLSVLHGINAPEF

750   FDKAVFSSLVLTLRDEGYISDSGDAEPAETMKVYQLLAELITSDVRLTIE

800   SATQGEG

sp|P0ACH5|MARA_ECOLI Multiple antibiotic resistance protein MarA OS=Escherichia coli (strain K12) OX=83333 GN=marA PE=1 SV=2

****

0   MSRRNTDAITIHSILDWIEDNLESPLSLEKVSERSGYSKWHLQRMFKKET

50   GHSLGQYIRSRKMTEIAQKLKESNEPILYLAERYGFESQQTLTRTFKNYF

100   DVPPHKYRMTNMQGESRFLHPLNHYNS

sp|P0A9M8|PTA_ECOLI Phosphate acetyltransferase OS=Escherichia coli (strain K12) OX=83333 GN=pta PE=1 SV=2

0   MSRIIMLIPTGTSVGLTSVSLGVIRAMERKGVRLSVFKPIAQPRTGGDAP

50   DQTTTIVRANSSTTTAAEPLKMSYVEGLLSSNQKDVLMEEIVANYHANTK

100   DAEVVLVEGLVPTRKHQFAQSLNYEIAKTLNAEIVFVMSQGTDTPEQLKE

****

150   RIELTRNSFGGAKNTNITGVIVNKLNAPVDEQGRTRPDLSEIFDDSSKAK

****

200   VNNVDPAKLQESSPLPVLGAVPWSFDLIATRAIDMARHLNATIINEGDIN

250   TRRVKSVTFCARSIPHMLEHFRAGSLLVTSADRPDVLVAACLAAMNGVEI

300   GALLLTGGYEMDARISKLCERAFATGLPVFMVNTNTWQTSLSLQSFNLEV

350   PVDDHERIEKVQEYVANYINADWIESLTATSERSRRLSPPAFRYQLTELA

400   RKAGKRIVLPEGDEPRTVKAAAICAERGIATCVLLGNPAEINRVAASQGV

450   ELGAGIEIVDPEVVRESYVGRLVELRKNKGMTETVAREQLEDNVVLGTLM

500   LEQDEVDGLVSGAVHTTANTIRPPLQLIKTAPGSSLVSSVFFMLLPEQVY

550   VYGDCAINPDPTAEQLAEIAIQSADSAAAFGIEPRVAMLSYSTGTSGAGS

600   DVEKVREATRLAQEKRPDLMIDGPLQYDAAVMADVAKSKAPNSPVAGRAT

650   VFIFPDLNTGNTTYKAVQRSADLISIGPMLQGMRKPVNDLSRGALVDDIV

700   YTIALTAIQSAQQQ

sp|P39346|IDND_ECOLI L-idonate 5-dehydrogenase (NAD(P)(+)) OS=Escherichia coli (strain K12) OX=83333 GN=idnD PE=1 SV=1

0   MQVKTQSCVVAGKKTVAVTEQTIDWNNNGTLVQITRGGICGSDLHYYQEG

```
50    KVGNFMIKAPMVLGHEVIGKVIHSDSSELHEGQTVAINPSKPCGHCKYCI

100   EHNENQCTDMRFFGSAMYFPHVDGGFTRYKMVETSQCVPYPAKADEKVMA

150   FAEPLAVAIHAAHQAGELQGKRVFISGVGPIGCLIVSAVKTLGAAEIVCA
                             ********************
200   DVSPRSLSLGKEMGADVLVNPQNDDMDHWKAEKGYFDVSFEVSGHPSSVN

250   TCLEVTRARGVMVQVGMGGAMAEFPMMTLIGKEISLRGSFRFTSEFNTAV

300   SWLANGVINPLPLLSAEYPFTDLEEALRFAGDKTQAAKVQLVF
```

sp|P03004|DNAA_ECOLI Chromosomal replication initiator protein DnaA OS=Escherichia coli (strain K12) OX=83333 GN=dnaA PE=1 SV=2

```
0     MSLSLWQQCLARLQDELPATEFSMWIRPLQAELSDNTLALYAPNRFVLDW

50    VRDKYLNNINGLLTSFCGADAPQLRFEVGTKPVTQTPQAAVTSNVAAPAQ

100   VAQTQPQRAAPSTRSGWDNVPAPAEPTYRSNVNVKHTFDNFVEGKSNQLA

150   RAAARQVADNPGGAYNPLFLYGGTGLGKTHLLHAVGNGIMARKPNAKVVY

200   MHSERFVQDMVKALQNNAIEEFKRYYRSVDALLIDDIQFFANKERSQEEF

250   FHTFNALLEGNQQIILTSDRYPKEINGVEDRLKSRFGWGLTVAIEPPELE

300   TRVAILMKKADENDIRLPGEVAFFIAKRLRSNVRELEGALNRVIANANFT

350   GRAITIDFVREALRDLLALQEKLVTIDNIQKTVAEYYKIKVADLLSKRRS
                                             ***********
400   RSVARPRQMAMALAKELTNHSLPEIGDAFGGRDHTTVLHACRKIEQLREE
      *****************
450   SHDIKEDFSNLIRTLSS
```

sp|P23524|GLXK1_ECOLI Glycerate 2-kinase OS=Escherichia coli (strain K12) OX=83333 GN=garK PE=1 SV=2

```
           **********************
0     MKIVIAPDSYKESLSASEVAQAIEKGFREIFPDAQYVSVPVADGGEGTVE

50    AMIAATQGAERHAWVTGPLGEKVNASWGISGDGKTAFIEMAAASGLELVP

100   AEKRDPLVTTSRGTGELILQALESGATNIIIGIGGSATNDGGAGMVQALG

150   AKLCDANGNEIGFGGGSLNTLNDIDISGLDPRLKDCVIRVACDVTNPLVG

200   DNGASRIFGPQKGASEAMIVELDNNLSHYAEVIKKALHVDVKDVPGAGAA

250   GGMGAALMAFLGAELKSGIEIVTTALNLEEHIHDCTLVITGEGRIDSQSI

300   HGKVPIGVANVAKKYHKPVIGIAGSLTDDVGVVHQHGIDAVFSVLTSIGT

350   LDEAFRGAYDNICRASRNIAATLAIGMRNAG
```

```
sp|P36548|AMIA_ECOLI N-acetylmuramoyl-L-alanine amidase AmiA
OS=Escherichia coli (strain K12) OX=83333 GN=amiA PE=1 SV=1
                                      ******************
0     MSTFKPLKTLTSRRQVLKAGLAALTLSGMSQAIAKDELLKTSNGHSKPKA
      ******
50    KKSGGKRVVVLDPGHGGIDTGAIGRNGSKEKHVVLAIAKNVRSILRNHGI

100   DARLTRSGDTFIPLYDRVEIAHKHGADLFMSIHADGFTNPKAAGASVFAL
                  *********************
150   SNRGASSAMAKYLSERENRADEVAGKKATDKDHLLQQVLFDLVQTDTIKN

200   SLTLGSHILKKIKPVHKLHSRNTEQAAFVVLKSPSVPSVLVETSFITNPE

250   EERLLGTAAFRQKIATAIAEGVISYFHWFDNQKAHSKKR

sp|P76544|YFFM_ECOLI Uncharacterized protein YffM OS=Escherichia coli
(strain K12) OX=83333 GN=yffM PE=4 SV=1
                                        ****************
0     MMVIRHECPSYCIAQKRVALREFSELVLGTLSLLLEQKTNGKCSASLYDC
      ******************
50    SEEEKLFVKRLKLIKADIHAQLKACDCDISE

sp|P76049|YCJY_ECOLI Uncharacterized protein YcjY OS=Escherichia coli
(strain K12) OX=83333 GN=ycjY PE=1 SV=2

0     MMNNKVSFTNSNNPTISLSAVIYFPPKFDETRQYQAIVLSHPGGGVKEQT

50    AGTYAKKLAEKGFVTIAYDASYQGESGGEPRQLENPYIRTEDISAVIDYL

100   TTLSYVDNTRIGAMGICAGAGYTANAAIQDRRIKAIGTVSAVNIGSIFRN

150   GWENNVKSIDALPYVEAGSNARTSDISSGEYAIMPLAPMKESDAPNEELR

200   QAWEYYHTPRAQYPTAPGYATLRSLNQIITYDAYHMAEVYLTQPTQIVAG
      ************************
250   SQAGSKWMSDDLYDRASSQDKRYHIVEGANHMDLYDGKAYVAEAISVLAP

300   FFEETL

sp|P69428|TATA_ECOLI Sec-independent protein translocase protein TatA
OS=Escherichia coli (strain K12) OX=83333 GN=tatA PE=1 SV=1
                                ************************
0     MGGISIWQLLIIAVIVVLLFGTKKLGSIGSDLGASIKGFKKAMSDDEPKQ
      **************
50    DKTSQDADFTAKTIADKQADTNQEQAKTEDAKRHDKEQV

sp|P0CE48|EFTU2_ECOLI Elongation factor Tu 2 OS=Escherichia coli (strain
K12) OX=83333 GN=tufB PE=1 SV=1

0     MSKEKFERTKPHVNVGTIGHVDHGKTTLTAAITTVLAKTYGGAARAFDQI

50    DNAPEEKARGITINTSHVEYDTPTRHYAHVDCPGHADYVKNMITGAAQMD
                                          **************
100   GAILVVAATDGPMPQTREHILLGRQVGVPYIIVFLNKCDMVDDEELLELV
```

******
150 EMEVRELLSQYDFPGDDTPIVRGSALKALEGDAEWEAKILELAGFLDSYI

200 PEPERAIDKPFLLPIEDVFSISGRGTVVTGRVERGIIKVGEEVEIVGIKE

250 TQKSTCTGVEMFRKLLDEGRAGENVGVLLRGIKREEIERGQVLAKPGTIK

300 PHTKFESEVYILSKDEGGRHTPFFKGYRPQFYFRTTDVTGTIELPEGVEM

350 VMPGDNIKMVVTLIHPIAMDDGLRFAIREGGRTVGAGVVAKVLS

sp|P27296|DING_ECOLI ATP-dependent DNA helicase DinG OS=Escherichia coli (strain K12) OX=83333 GN=dinG PE=1 SV=3

0 MALTAALKAQIAAWYKALQEQIPDFIPRAPQRQMIADVAKTLAGEEGRHL

50 AIEAPTGVGKTLSYLIPGIAIAREEQKTLVVSTANVALQDQIYSKDLPLL

100 KKIIPDLKFTAAFGRGRYVCPRNLTALASTEPTQQDLLAFLDDELTPNNQ

150 EEQKRCAKLKGDLDTYKWDGLRDHTDIAIDDDLWRRLSTDKASCLNRNCY

200 YYRECPFFVARREIQEAEVVVANHALVMAAMESEAVLPDPKNLLLVLDEG

250 HHLPDVARDALEMSAEITAPWYRLQLDLFTKLVATCMEQFRPKTIPPLAI

300 PERLNAHCEELYELIASLNNILNLYMPAGQEAEHRFAMGELPDEVLEICQ

350 RLAKLTEMLRGLAELFLNDLSEKTGSHDIVRLHRLILQMNRALGMFEAQS

400 KLWRLASLAQSSGAPVTKWATREEREGQLHLWFHCVGIRVSDQLERLLWR

450 SIPHIIVTSATLRSLNSFSRLQEMSGLKEKAGDRFVALDSPFNHCEQGKI

500 VIPRMRVEPSIDNEEQHIAEMAAFFRKQVESKKHLGMLVLFASGRAMQRF

550 LDYVTDLRLMLLVQGDQPRYRLVELHRKRVANGERSVLVGLQSFAEGLDL

600 KGDLLSQVHIHKIAFPPIDSPVVITEGEWLKSLNRYPFEVQSLPSASFNL

****

650 IQQVGRLIRSHGCWGEVVIYDKRLLTKNYGKRLLDALPVFPIEQPEVPEG

****

700 IVKKKEKTKSPRRRRR

sp|P0AEY1|MARC_ECOLI UPF0056 inner membrane protein MarC OS=Escherichia coli (strain K12) OX=83333 GN=marC PE=1 SV=1

0 MLDLFKAIGLGLVVLLPLANPLTTVALFLGLAGNMNSAERNRQSLMASVY

****

50 VFAIMMVAYYAGQLVMDTFGISIPGLRIAGGLIVAFIGFRMLFPQQKAID

****

100 SPEAKSKSEELEDEPSANIAFVPLAMPSTAGPGTIAMIISSASTVRQSST

150 FADWVLMVAPPLIFFLVAVILWGSLRSSGAIMRLVGKGGIEAISRLMGFL

200   LVCMGVQFIINGILEIIKTYH

sp|P0A8U6|METJ_ECOLI Met repressor OS=Escherichia coli (strain K12) OX=83333 GN=metJ PE=1 SV=2

          ****
0   MAEWSGEYISPYAEHGKKSEQVKKITVSIPLKVLKILTDERTRRQVNNLR
                          ****
50   HATNSELLCEAFLHAFTGQPLPDDADLRKERSDEIPEAAKEIMREMGINP

100   ETWEY

sp|P04825|AMPN_ECOLI Aminopeptidase N OS=Escherichia coli (strain K12) OX=83333 GN=pepN PE=1 SV=2

0   MTQQPQAKYRHDYRAPDYQITDIDLTFDLDAQKTVVTAVSQAVRHGASDA

50   PLRLNGEDLKLVSVHINDEPWTAWKEEEGALVISNLPERFTLKIINEISP

100   AANTALEGLYQSGDALCTQCEAEGFRHITYYLDRPDVLARFTTKIIADKI

150   KYPFLLSNGNRVAQGELENGRHWVQWQDPFPKPCYLFALVAGDFDVLRDT
                                  ****
200   FTTRSGREVALELYVDRGNLDRAPWAMTSLKNSMKWDEERFGLEYDLDIY

250   MIVAVDFFNMGAMENKGLNIFNSKYVLARTDTATDKDYLDIERVIGHEYF

300   HNWTGNRVTCRDWFQLSLKEGLTVFRDQEFSSDLGSRAVNRINNVRTMRG

350   LQFAEDASPMAHPIRPDMVIEMNNFYTLTVYEKGAEVIRMIHTLLGEENF

400   QKGMQLYFERHDGSAATCDDFVQAMEDASNVDLSHFRRWYSQSGTPIVTV

450   KDDYNPETEQYTLTISQRTPATPDQAEKQPLHIPFAIELYDNEGKVIPLQ

500   KGGHPVNSVLNVTQAEQTFVFDNVYFQPVPALLCEFSAPVKLEYKWSDQQ

550   LTFLMRHARNDFSRWDAAQSLLATYIKLNVARHQQGQPLSLPVHVADAFR

600   AVLLDEKIDPALAAEILTLPSVNEMAELFDIIDPIAIAEVREALTRTLAT

650   ELADELLAIYNANYQSEYRVEHEDIAKRTLRNACLRFLAFGETHLADVLV

700   SKQFHEANNMTDALAALSAAVAAQLPCRDALMQEYDDKWHQNGLVMDKWF

750   ILQATSPAANVLETVRGLLQHRSFTMSNPNRIRSLIGAFAGSNPAAFHAE

800   DGSGYLFLVEMLTDLNSRNPQVASRLIEPLIRLKRYDAKRQEKMRAALEQ

850   LKGLENLSGDLYEKITKALA

sp|P76548|YFFQ_ECOLI Uncharacterized protein YffQ OS=Escherichia coli (strain K12) OX=83333 GN=yffQ PE=4 SV=2

0   MKLLIAIILMVLTGVCFADVGDYRLNGEDNARIESVVTDNCEKTAVLVGG
                                             ****
50   DRLARVEIEYIATLCKPVALVIIYDRFDDIAAIPLKVTLKKVLKENSDEK
      ****
100   INLLNKMGDLAGRIVAEQYLGMSFE

sp|P08201|NIRB_ECOLI Nitrite reductase (NADH) large subunit OS=Escherichia coli (strain K12) OX=83333 GN=nirB PE=3 SV=4

0   MSKVRLAIIGNGMVGHRFIEDLLDKSDAANFDITVFCEEPRIAYDRVHLS

50   SYFSHHTAEELSLVREGFYEKHGIKVLVGERAITINRQEKVIHSSAGRTV

100   FYDKLIMATGSYPWIPPIKGSDTQDCFVYRTIEDLNAIESCARRSKRGAV

150   VGGGLLGLEAAGALKNLGIETHVIEFAPMLMAEQLDQMGGEQLRRKIESM

200   GVRVHTSKNTLEIVQEGVEARKTMRFADGSELEVDFIVFSTGIRPRDKLA

250   TQCGLDVAPRGGIVINDSCQTSDPDIYAIGECASWNNRVFGLVAPGYKMA
                                                  ******
300   QVAVDHILGSENAFEGADLSAKLKLLGVDVGGIGDAHGRTPGARSYVYLD
      ****
350   ESKEIYKRLIVSEDNKTLLGAVLVGDTSDYGNLLQLVLNAIELPENPDSL

400   ILPAHSGSGKPSIGVDKLPDSAQICSCFDVTKGDLIAAINKGCHTVAALK

450   AETKAGTGCGGCIPLVTQVLNAELAKQGIEVNNNLCEHFAYSRQELFHLI

500   RVEGIKTFEELLAKHGKGYGCEVCKPTVGSLLASCWNEYILKPEHTPLQD

550   SNDNFLANIQKDGTYSVIPRSPGGEITPEGLMAVGRIAREFNLYTKITGS

600   QRLAMFGAQKDDLPEIWRQLIEAGFETGHAYAKALRMAKTCVGSTWCRYG

650   VGDSVGLGVELENRYKGIRTPHKMKFGVSGCTRECSEAQGKDVGIIATEK

700   GWNLYVCGNGGMKPRHADLLAADIDRETLIKYLDRFMMFYIRTADKLTRT

750   APWLENLEGGIDYLKAVIIDDKLGLNAHLEEEMARLREAVLCEWTETVNT

800   PSAQTRFKHFINSDKRDPNVQMVPEREQHRPATPYERIPVTLVEDNA

sp|Q46786|YGEF_ECOLI Putative uncharacterized protein YgeF OS=Escherichia coli (strain K12) OX=83333 GN=ygeF PE=5 SV=1
                     ****
0   MKPRNINNSLPLQPLVPDQENKNKKNEEKSVNPVKITMGSGLNYIEQESL

50   GGKYLTHDLSIKIADISEEIIQQAILSAMSIYKFSITDDLMSMAVNELIK

100   LTKIENNVDLNKFTTICTDVLSPRVTRHNKEKNKRHSTLLKNPLFNFH

sp|P37747|GLF_ECOLI UDP-galactopyranose mutase OS=Escherichia coli (strain K12) OX=83333 GN=glf PE=1 SV=1

```
0     MYDYIIVGSGLFGAVCANELKKLNKKVLVIEKRNHIGGNAYTEDCEGIQI

50    HKYGAHIFHTNDKYIWDYVNDLVEFNRFTNSPLAIYKDKLFNLPFNMNTF

100   HQMWGVKDPQEAQNIINAQKKKYGDKVPENLEEQAISLVGEDLYQALIKG
                                                    **
150   YTEKQWGRSAKELPAFIIKRIPVRFTFDNNYFSDRYQGIPVGGYTKLIEK
      *************************
200   MLEGVDVKLGIDFLKDKDSLASKAHRIIYTGPIDQYFDYRFGALEYRSLK

250   FETERHEFPNFQGNAVINFTDANVPYTRIIEHKHFDYVETKHTVVTKEYP
                    ********************
300   LEWKVGDEPYYPVNDNKNMELFKKYRELASREDKVIFGGRLAEYKYYDMH

350   QVISAALYQVKNIMSTD
```

sp|P77485|CUSS_ECOLI Sensor histidine kinase CusS OS=Escherichia coli (strain K12) OX=83333 GN=cusS PE=1 SV=1

```
0     MVSKPFQRPFSLATRLTFFISLATIAAFFAFAWIMIHSVKVHFAEQDIND

50    LKEISATLERVLNHPDETQARRLMTLEDIVSGYSNVLISLADSQGKTVYH

100   SPGAPDIREFTRDAIPDKDAQGGEVYLLSGPTMMMPGHGHGHMEHSNWRM

150   INLPVGPLVDGKPIYTLYIALSIDFHLHYINDLMNKLIMTASVISILIVF

200   IVLLAVHKGHAPIRSVSRQIQNITSKDLDVRLDPQTVPIELEQLVLSFNH
                                             *************
250   MIERIEDVFTRQSNFSADIAHEIRTPITNLITQTEIALSQSRSQKELEDV
      ********
300   LYSNLEELTRMAKMVSDMLFLAQADNNQLIPEKKMLNLADEVGKVFDFFE

350   ALAEDRGVELRFVGDKCQVAGDPLMLRRALSNLLSNALRYTPTGETIVVR

400   CQTVDHLVQVIVENPGTPIAPEHLPRLFDRFYRVDPSRQRKGEGSGIGLA

450   IVKSIVVAHKGTVAVTSDARGTRFVITLPA
```

sp|P76042|YCJN_ECOLI Putative ABC transporter periplasmic-binding protein YcjN OS=Escherichia coli (strain K12) OX=83333 GN=ycjN PE=3 SV=1

```
                           ********************
0     MIKSKIVLLSALVSCALISGCKEENKTNVSIEFMHSSVEQERQAVISKLI

50    ARFEKENPGITVKQVPVEEDAYNTKVITLSRSGSLPEVIETSHDYAKVMD

100   KEQLIDRKAVATVISNVGEGAFYDGVLRIVRTEDGSAWTGVPVSAWIGGI

150   WYRKDVLAKAGLEEPKNWQQLLDVAQKLNDPANKKYGIALPTAESVLTEQ

200   SFSQFALSNQANVFNAEGKITLDTPEMMQALTYYRDLTANTMPGSNDIME

250   VKDAFMNGTAPMAIYSTYILPAVIKEGDPKNVGFVVPTEKNSAVYGMLTS
```

```
300   LTITAGQKTEETEAAEKFVTFMEQADNIADWVMMSPGAALPVNKAVVTTA

350   TWKDNDVIKALGELPNQLIGELPNIQVFGAVGDKNFTRMGDVTGSGVVSS

400   MVHNVTVGKADLSTTLQASQKKLDELIEQH
```

sp|P77228|YDFJ_ECOLI Putative transporter YdfJ OS=Escherichia coli (strain K12) OX=83333 GN=ydfJ PE=1 SV=1

```
0     MDFQLYSLGAALVFHEIFFPESSTAMALILAMGTYGAGYVARIVGAFIFG

50    KMGDRIGRKKVLFITITMMGICTTLIGVLPTYAQIGVFAPILLVTLRIIQ

100   GLGAGAEISGAGTMLAEYAPKGKRGIISSFVAMGTNCGTLSATAIWAFMF

150   FILSKEELLAWGWRIPFLASVVVMVFAIWLRMNLKESPVFEKVNDSNQPT

200   AKPAPAGSMFQSKSFWLATGLRFGQAGNSGLIQTFLAGYLVQTLLFNKAI

250   PTDALMISSILGFMTIPFLGWLSDKIGRRIPYIIMNTSAIVLAWPMLSII

300   VDKSYAPSTIMVALIVIHNCAVLGLFALENITMAEMFGCKNRFTRMAISK
                                                     *******
350   EIGGLIASGFGPILAGIFCTMTESWYPIAIMIMAYSVIGLISALKMPEVK
      **************
400   DRDLSALEDAAEDQPRVVRAAQPSRSL
```

sp|P60752|MSBA_ECOLI Lipid A export ATP-binding/permease protein MsbA OS=Escherichia coli (strain K12) OX=83333 GN=msbA PE=1 SV=1

```
0     MHNDKDLSTWQTFRRLWPTIAPFKAGLIVAGVALILNAASDTFMLSLLKP

50    LLDDGFGKTDRSVLVWMPLVVIGLMILRGITSYVSSYCISWVSGKVVMTM

100   RRRLFGHMMGMPVSFFDKQSTGTLLSRITYDSEQVASSSSGALITVVREG

150   ASIIGLFIMMFYYSWQLSIILIVLAPIVSIAIRVVSKRFRNISKNMQNTM

200   GQVTTSAEQMLKGHKEVLIFGGQEVETKRFDKVSNRMRLQGMKMVSASSI

250   SDPIIQLIASLALAFVLYAASFPSVMDSLTAGTITVVFSSMIALMRPLKS
                             **********************
300   LTNVNAQFQRGMAACQTLFTILDSEQEKDEGKRVIERATGDVEFRNVTFT

350   YPGRDVPALRNINLKIPAGKTVALVGRSGSGKSTIASLITRFYDIDEGEI

400   LMDGHDLREYTLASLRNQVALVSQNVHLFNDTVANNIAYARTEQYSREQI

450   EEAARMAYAMDFINKMDNGLDTVIGENGVLLSGGQRQRIAIARALLRDSP

500   ILILDEATSALDTESERAIQAALDELQKNRTSLVIAHRLSTIEKADEIVV

550   VEDGVIVERGTHNDLLEHRGVYAQLHKMQFGQ
```

sp|P32176|FDOG_ECOLI Formate dehydrogenase-O major subunit OS=Escherichia coli (strain K12) OX=83333 GN=fdoG PE=1 SV=5

0   MQVSRRQFFKICAGGMAGTTAAALGFAPSVALAETRQYKLLRTRETRNTC

50   TYCSVGCGLLMYSLGDGAKNAKASIFHIEGDPDHPVNRGALCPKGAGLVD

100   FIHSESRLKFPEYRAPGSDKWQQISWEEAFDRIAKLMKEDRDANYIAQNA

150   EGVTVNRWLSTGMLCASASSNETGYLTQKFSRALGMLAVDNQARVUHGPT

200   VASLAPTFGRGAMTNHWVDIKNANLVVVMGGNAAEAHPVGFRWAMEAKIH

250   NGAKLIVIDPRFTRTAAVADYYAPIRSGTDIAFLSGVLLYLLNNEKFNRE
                       ****
300   YTEAYTNASLIVREDYGFEDGLFTGYDAEKRKYDKSSWTYELDENGFAKR
      *
350   DTTLQHPRCVWNLLKQHVSRYTPDVVENICGTPKDAFLKVCEYIAETSAH

400   DKTASFLYALGWTQHSVGAQNIRTMAMIQLLLGNMGMAGGGVNALRGHSN

450   IQGLTDLGLLSQSLPGYMTLPSEKQTDLQTYLTANTPKPLLEGQVNYWGN

500   YPKFFVSMMKAFFGDKATAENSWGFDWLPKWDKGYDVLQYFEMMKEGKVN

550   GYICQGFNPVASFPNKNKVIGCLSKLKFLVTIDPLNTETSNFWQNHGELN

600   EVDSSKIQTEVFRLPSTCFAEENGSIVNSGRWLQWHWKGADAPGIALTDG

650   EILSGIFLRLRKMYAEQGGANPDQVLNMTWNYAIPHEPSSEEVAMESNGK

700   ALADITDPATGAVIVKKGQQLSSFAQLRDDGTTSCGCWIFAGSWTPEGNQ

750   MARRDNADPSGLGNTLGWAWAWPLNRRILYNRASADPQGNPWDPKRQLLK

800   WDGTKWTGWDIPDYSAAPPGSGVGPFIMQQEGMGRLFALDKMAEGPFPEH

850   YEPFETPLGTNPLHPNVISNPAARIFKDDAEALGKADKFPYVGTTYRLTE

900   HFHYWTKHALLNAILQPEQFVEIGESLANKLGIAQGDTVKVSSNRGYIKA

950   KAVVTKRIRTLKANGKDIDTIGIPIHWGYEGVAKKGFIANTLTPFVGDAN

1000 TQTPEFKSFLVNVEKV

sp|Q06067|ATOS_ECOLI Signal transduction histidine-protein kinase AtoS OS=Escherichia coli (strain K12) OX=83333 GN=atoS PE=1 SV=1
                                      ****
0   MHYMKWIYPRRLRNQMILMAILMVIVPTLTIGYIVETEGRSAVLSEKEKK
      *****
50   LSAVVNLLNQALGDRYDLYIDLPREERIRALNAELAPITENITHAFPGIG

100   AGYYNKMLDAIITYAPSALYQNNVGVTIAADHPGREVMRTNTPLVYSGRQ

150 VRGDILNSMLPIERNGEILGYIWANELTEDIRRQAWKMDVRIIIVLTAGL

200 LISLLLIVLFSRRLSANIDIITDGLSTLAQNIPTRLPQLPGEMGQISQSV

250 NNLAQALRETRTLNDLIIENAADGVIAIDRQGDVTTMNPAAEVITGYQRH

300 ELVGQPYSMLFDNTQFYSPVLDTLEHGTEHVALEISFPGRDRTIELSVTT

350 SRIHNTHGEMIGALVIFSDLTARKETQRRMAQAERLATLGELMAGVAHEV

400 RNPLTAIRGYVQILRQQTSDPIHQEYLSVVLKEIDSINKVIQQLLEFSRP

450 RHSQWQQVSLNALVEETLVLVQTAGVQARVDFISELDNELSPINADRELL

500 KQVLLNILINAVQAISARGKIRIQTWQYSDSQQAISIEDNGCGIDLSLQK

550 KIFDPFFTTKASGTGLGLALSQRIINAHQGDIRVASLPGYGATFTLILPI

600 NPQGNQTV

sp|P0A9Q5|ACCD_ECOLI Acetyl-coenzyme A carboxylase carboxyl transferase subunit beta OS=Escherichia coli (strain K12) OX=83333 GN=accD PE=1 SV=1

```
0     MSWIERIKSNITPTRKASIPEGVWTKCDSCGQVLYRAELERNLEVCPKCD
                 ***************************************
50    HHMRMTARNRLHSLLDEGSLVELGSELEPKDVLKFRDSKKYKDRLASAQK
      ********
100   ETGEKDALVVMKGTLYGMPVVAAAFEFAFMGGSMGSVVGARFVRAVEQAL

150   EDNCPLICFSASGGARMQEALMSLMQMAKTSAALAKMQERGLPYISVLTD

200   PTMGGVSASFAMLGDLNIAEPKALIGFAGPRVIEQTVREKLPPGFQRSEF

250   LIEKGAIDMIVRRPEMRLKLASILAKLMNLPAPNPEAPREGVVVPPVPDQ

300   EPEA
```

sp|P17169|GLMS_ECOLI Glutamine--fructose-6-phosphate aminotransferase [isomerizing] OS=Escherichia coli (strain K12) OX=83333 GN=glmS PE=1 SV=4

0 MCGIVGAIAQRDVAEILLEGLRRLEYRGYDSAGLAVVDAEGHMTRLRRLG

50 KVQMLAQAAEEHPLHGGTGIAHTRWATHGEPSEVNAHPHVSEHIVVVHNG

100 IIENHEPLREELKARGYTFVSETDTEVIAHLVNWELKQGGTLREAVLRAI

150 PQLRGAYGTVIMDSRHPDTLLAARSGSPLVIGLGMGENFIASDQLALLPV

200 TRRFIFLEEGDIAEITRRSVNIFDKTGAEVKRQDIESNLQYDAGDKGIYR

250 HYMQKEIYEQPNAIKNTLTGRISHGQVDLSELGPNADELLSKVEHIQILA

300 CGTSYNSGMVSRYWFESLAGIPCDVEIASEFRYRKSAVRRNSLMITLSQS

```
350   GETADTLAGLRLSKELGYLGSLAICNVPGSSLVRESDLALMTNAGTEIGV
                                                 *****
400   ASTKAFTTQLTVLLMLVAKLSRLKGLDASIEHDIVHGLQALPSRIEQMLS
      ***************
450   QDKRIEALAEDFSDKHHALFLGRGDQYPIALEGALKLKEISYIHAEAYAA

500   GELKHGPLALIDADMPVIVVAPNNELLEKLKSNIEEVRARGGQLYVFADQ

550   DAGFVSSDNMHIIEMPHVEEVIAPIFYTVPLQLLAYHVALIKGTDVDQPR

600   NLAKSVTVE
```

sp|P0A715|KDSA_ECOLI 2-dehydro-3-deoxyphosphooctonate aldolase OS=Escherichia coli (strain K12) OX=83333 GN=kdsA PE=1 SV=1

```
0     MKQKVVSIGDINVANDLPFVLFGGMNVLESRDLAMRICEHYVTVTQKLGI

50    PYVFKASFDKANRSSIHSYRGPGLEEGMKIFQELKQTFGVKIITDVHEPS

100   QAQPVADVVDVIQLPAFLARQTDLVEAMAKTGAVINVKKPQFVSPGQMGN

150   IVDKFKEGGNEKVILCDRGANFGYDNLVVDMLGFSIMKKVSGNSPVIFDV

200   THALQCRDPFGAASGGRRAQVAELARAGMAVGLAGLFIEAHPDPEHAKCD
                    ********************
250   GPSALPLAKLEPFLKQMKAIDDLVKGFEELDTSK
```

sp|Q46858|YQHG_ECOLI Uncharacterized protein YqhG OS=Escherichia coli (strain K12) OX=83333 GN=yqhG PE=3 SV=3

```
0     MKIILLFLAALASFTVHAQPPSQTVEQTVRHIYQNYKSDATAPYFGETGE

50    RAITSARIQQALTLNDNLTLPGNIGWLDYDPVCDCQDFGDLVLESVAITQ

100   TDADHADAVVRFRIFKDDKEKTTQTLKMVAENGRWVIDDIVSNHGSVLQA

150   VNSENEKTLAALASLQKEQPEAFVAELFEHIADYSWPWTWVVSDSYRQAV
                                       *****************
200   NAFYKTTFKTANNPDEDMQIERQFIYDNPICFGEESLFSRVDEIRVLEKT
      ***
250   ADSARIHVRFTLTNGNNEEQELVLQRREGKWEIADFIRPNSGSLLKQIEA

300   KTAARLKQ
```

sp|P76656|YQII_ECOLI Uncharacterized protein YqiI OS=Escherichia coli (strain K12) OX=83333 GN=yqiI PE=3 SV=1

```
                                             ***********
0     MRYLLIVITFFMGFSSLPAWAMDCYAEHEGGNTVVIGYVPRISIPSDGKK
      ************
50    GDKIWQSSEYFMNVFCNNALPGPSPGEEYPSAWANIMMLLASGQDFYNQN

100   SYTFGVTYNGVDYDSTSPLPIAAPVCIDIKGAGTFGNGYKKPAVCSGGPE
```

```
150   PQLSVTFPVRVQLYIKLAKNANKVNKKLVLPDEYIALEFKGMSGAGAIEV

200   DKNLTFRIRGLNNIHVLDCFVNVDLEPADGVVDFGKINSRTIKNTSVSET

250   FSVVMTKDPGAACTEQFNILGSFFTTDILSDYSHLDIGNGLLLKIFHNDG

300   TATEFNRFSQFASFSSSSAPSVTAPFRAELSANPAETVVEGPFSKDVILK

350   ITYN
```

sp|P77515|STFQ_ECOLI Prophage side tail fiber protein homolog StfQ OS=Escherichia coli (strain K12) OX=83333 GN=stfQ PE=3 SV=1

```
0     MNITALTDNTQGAAGLELYEVYNNGYPTAYGNIIHLKGMTAVGEGELLIG

50    WSGTSGAHAPAFIRSRRDTTDANWSPWAQLYTSAHPPAEFYPVGAPIPWP

100   SDTVPSGYALMQGQTFDKSAYPKLAVAYPSGVIPDMRGWTIKGKPASGRA
        ********************
150   VLSQEQDGIKSHTHSASASSTDLGTETTSSFDYGTKSTNNTGAHTHSISG

200   TANSAGAHQHKSSGAFGGTNTSIFPNGYTAISNLSAGIMSTTSGSGQTRN

250   AGKTSSDGAHTHSLSGTAASAGAHAHTVGIGAHTHSVAIGSHGHTITVNA

300   AGNAENTVKNIAFNYIVRLA
```

sp|P0AEY3|MAZG_ECOLI Nucleoside triphosphate pyrophosphohydrolase OS=Escherichia coli (strain K12) OX=83333 GN=mazG PE=1 SV=1

```
0     MNQIDRLLTIMQRLRDPENGCPWDKEQTFATIAPYTLEETYEVLDAIARE

50    DFDDLRGELGDLLFQVVFYAQMAQEEGRFDFNDICAAISDKLERRHPHVF
       *********************
100   ADSSAENSSEVLARWEQIKTEERAQKAQHSALDDIPRSLPALMRAQKIQK

150   RCANVGFDWTTLGPVVDKVYEEIDEVMYEARQAVVDQAKLEEEMGDLLFA

200   TVNLARHLGTKAEIALQKANEKFERRFREVERIVAARGLEMTGVDLETME

250   EVWQQVKRQEIDL
```

sp|P0A832|SSRP_ECOLI SsrA-binding protein OS=Escherichia coli (strain K12) OX=83333 GN=smpB PE=1 SV=2

```
0     MTKKKAHKPGSATIALNKRARHEYFIEEEFEAGLALQGWEVKSLRAGKAN

50    ISDSYVLLRDGEAFLFGANITPMAVASTHVVCDPTRTRKLLLNQRELDSL
                             ***************************
100   YGRVNREGYTVVALSLYWKNAWCKVKIGVAKGKKQHDKRSDIKEREWQVD
      ******
150   KARIMKNAHR
```

```
sp|P0CF67|INSE2_ECOLI Transposase InsE for insertion sequence IS3B
OS=Escherichia coli (strain K12) OX=83333 GN=insE2 PE=3 SV=1
        ***********************
0     MTKTVSTSKKPRKQHSPEFRSEALKLAERIGVTAAARELSLYESQLYNWR

50    SKQQNQQTSSERELEMSTEIARLKRQLAERDEELAILQKAATYFAKRLK

sp|P13024|FDHE_ECOLI Protein FdhE OS=Escherichia coli (strain K12)
OX=83333 GN=fdhE PE=1 SV=2

0     MSIRIIPQDELGSSEKRTADMIPPLLFPRLKNLYNRRAERLRELAENNPL

50    GDYLRFAALIAHAQEVVLYDHPLEMDLTARIKEASAQGKPPLDIHVLPRD
                                          **************
100   KHWQKLLMALIAELKPEMSGPALAVIENLEKASTQELEDMASALFASDFS
      ********
150   SVSSDKAPFIWAALSLYWAQMANLIPGKARAEYGEQRQYCPVCGSMPVSS
                                             ***********
200   MVQIGTTQGLRYLHCNLCETEWHVVRVKCSNCEQSGKLHYWSLDDEQAAI
      **********************
250   KAESCDDCDTYLKILYQEKDPKIEAVADDLASLVLDARMEQEGYARSSIN

300   PFLFPGEGE

sp|P0A843|TATE_ECOLI Sec-independent protein translocase protein TatE
OS=Escherichia coli (strain K12) OX=83333 GN=tatE PE=2 SV=1
                                                  ******
0     MGEISITKLLVVAALVVLLFGTKKLRTLGGDLGAAIKGFKKAMNDDDAAA
      **************
50    KKGADVDLQAEKLSHKE

sp|P06612|TOP1_ECOLI DNA topoisomerase 1 OS=Escherichia coli (strain K12)
OX=83333 GN=topA PE=1 SV=2
                                        ****************
0     MGKALVIVESPAKAKTINKYLGSDYVVKSSVGHIRDLPTSGSAAKKSADS
      ******************
50    TSTKTAKKPKKDERGALVNRMGVDPWHNWEAHYEVLPGKEKVVSELKQLA

100   EKADHIYLATDLDREGEAIAWHLREVIGGDDARYSRVVFNEITKNAIRQA

150   FNKPGELNIDRVNAQQARRFMDRVVGYMVSPLLWKKIARGLSAGRVQSVA

200   VRLVVEREREIKAFVPEEFWEVDASTTTPSGEALALQVTHQNDKPFRPVN

250   KEQTQAAVSLLEKARYSVLEREDKPTTSKPGAPFITSTLQQAASTRLGFG

300   VKKTMMMAQRLYEAGYITYMRTDSTNLSQDAVNMVRGYISDNFGKKYLPE

350   SPNQYASKENSQEAHEAIRPSDVNVMAESLKDMEADAQKLYQLIWRQFVA

400   CQMTPAKYDSTTLTVGAGDFRLKARGRILRFDGWTKVMPALRKGDEDRIL

450   PAVNKGDALTLVELTPAQHFTKPPARFSEASLVKELEKRGIGRPSTYASI
```

```
500   ISTIQDRGYVRVENRRFYAEKMGEIVTDRLEENFRELMNYDFTAQMENSL
                    ********************
550   DQVANHEAEWKAVLDHFFSDFTQQLDKAEKDPEEGGMRPNQMVLTSIDCP

600   TCGRKMGIRTASTGVFLGCSGYALPPKERCKTTINLVPENEVLNVLEGED

650   AETNALRAKRRCPKCGTAMDSYLIDPKRKLHVCGNNPTCDGYEIEEGEFR

700   IKGYDGPIVECEKCGSEMHLKMGRFGKYMACTNEECKNTRKILRNGEVAP

750   PKEDPVPLPELPCEKSDAYFVLRDGAAGVFLAANTFPKSRETRAPLVEEL

800   YRFRDRLPEKLRYLADAPQQDPEGNKTMVRFSRKTKQQYVSSEKDGKATG

850   WSAFYVDGKWVEGKK

sp|P69824|PTMA_ECOLI Mannitol-specific cryptic phosphotransferase enzyme
IIA component OS=Escherichia coli (strain K12) OX=83333 GN=cmtB PE=1 SV=1
         *************************
0     MRLSDYFPESSISVIHSAKDWQEAIDFSMVSLLDKNYISENYIQAIKDST

50    INNGPYYILAPGVAMPHARPECGALKTGMSLTLLEQGVYFPGNDEPIKLL
                        ********************
100   IGLSAADADSHIGAIQALSELLCEEEILEQLLTASSEKQLADIISRG

sp|P0A867|TALA_ECOLI Transaldolase A OS=Escherichia coli (strain K12)
OX=83333 GN=talA PE=3 SV=1

0     MNELDGIKQFTTVVADSGDIESIRHYHPQDATTNPSLLLKAAGLSQYEHL
                                             ***********
50    IDDAIAWGKKNGKTQEQQVVAACDKLAVNFGAEILKIVPGRVSTEVDARL
      ****************
100   SFDKEKSIEKARHLVDLYQQQGVEKSRILIKLASTWEGIRAAEELEKEGI

150   NCNLTLLFSFAQARACAEAGVFLISPFVGRIYDWYQARKPMDPYVVEEDP

200   GVKSVRNIYDYYKQHHYETIVMGASFRRTEQILALTGCDRLTIAPNLLKE

250   LQEKVSPVVRKLIPPSQTFPRPAPMSEAEFRWEHNQDAMAVEKLSEGIRL

300   FAVDQRKLEDLLAAKL

sp|P0A8H6|YIHI_ECOLI Der GTPase-activating protein YihI OS=Escherichia
coli (strain K12) OX=83333 GN=yihI PE=1 SV=1
         ********************
0     MKPSSSNSRSKGHAKARRKTREELDQEARDRKRQKKRRGHAPGSRAAGGN
                                          **************
50    TTSGSKGQNAPKDPRIGSKTPIPLGVTEKVTKQHKPKSEKPMLSPQAELE
      ******            ********************************
100   LLETDERLDALLERLEAGETLSAEEQSWVDAKLDRIDELMQKLGLSYDDD
      *******************
150   EEEEEDEKQEDMMRLLRGN
```

sp|P39353|YJHC_ECOLI Uncharacterized oxidoreductase YjhC OS=Escherichia coli (strain K12) OX=83333 GN=yjhC PE=1 SV=2

0 MINYGVVGVGYFGAELARFMNMHDNAKITCVYDPENGENIARELQCINMS

50 SLDALVSSKLVDCVIVATPNYLHKEPVIKAAKNKKHVFCEKPIALSYEDC

100 VDMVKACKEAGVTFMAGHIMNFFNGVQYARKLIKEGVIGEILSCHTKRNG

150 WENKQERLSWKKMKEQSGGHLYHHIHELDCVQHLLGEIPETVTMIGGNLA

200 HSGPGFGNEDDMLFMTLEFPSGKLATLEWGSAFNWPEHYVIINGTKGSIK
****

250 IDMQETAGSLRIGGQTKHFLVHETQEEDDDRRKGNMTSEMDGAIAYGHPG

300 KKTPLWLASLIRKETLFLHNILCGAKPEEDYIDLLNGEAAMSAIATADAA

350 TLSRSQDRKVKISEIIKHTSVM

sp|P75828|YBJD_ECOLI Uncharacterized protein YbjD OS=Escherichia coli (strain K12) OX=83333 GN=ybjD PE=4 SV=1

0 MILERVEIVGFRGINRLSLMLEQNNVLIGENAWGKSSLLDALTLLLSPES

50 DLYHFERDDFWFPPGDINGREHHLHIILTFRESLPGRHRVRRYRPLEACW
****

100 TPCTDGYHRIFYRLEGESAEDGSVMTLRSFLDKDGHPIDVEDINDQARHL

150 VRLMPVLRLRDARFMRRIRNGTVPNVPNVEVTARQLDFLARELSSHPQNL

200 SDGQIRQGLSAMVQLLEHYFSEQGAGQARYRLMRRRASNEQRSWRYLDII

250 NRMIDRPGGRSYRVILLGLFATLLQAKGTLRLDKDARPLLLIEDPETRLH

300 PIMLSVAWHLLNLLPLQRIATTNSGELLSLTPVEHVCRLVRESSRVAAWR

350 LGPSGLSTEDSRRISFHIRFNRPSSLFARCWLLVEGETETWVINELARQC

400 GHHFDAEGIKVIEFAQSGLKPLVKFARRMGIEWHVLVDGDEAGKKYAATV

450 RSLLNNDREAEREHLTALPALDMEHFMYRQGFSDVFHRMAQIPENVPMNL

500 RKIISKAIHRSSKPDLAIEVAMEAGRRGVDSVPTLLKKMFSRVLWLARGR

550 AD

sp|P09372|GRPE_ECOLI Protein GrpE OS=Escherichia coli (strain K12) OX=83333 GN=grpE PE=1 SV=1
****

0 MSSKEQKTPEGQAPEEIIMDQHEEIEAVEPEASAEQVDPRDEKVANLEAQ

50 LAEAQTRERDGILRVKAEMENLRRRTELDIEKAHKFALEKFINELLPVID

100 SLDRALEVADKANPDMSAMVEGIELTLKSMLDVVRKFGVEVIAETNVPLD

```
150   PNVHQAIAMVESDDVAPGNVLGIMQKGYTLNGRTIRAAMVTVAKAKA
```

sp|P0A8M6|YEEX_ECOLI UPF0265 protein YeeX OS=Escherichia coli (strain K12) OX=83333 GN=yeeX PE=1 SV=1

```
0     METTKPSFQDVLEFVRLFRRKNKLQREIQDVEKKIRDNQKRVLLLDNLSD
                        ********************************
50    YIKPGMSVEAIQGIIASMKGDYEDRVDDYIIKNAELSKERRDISKKLKAM
      *******
100   GEMKNGEAK
```

sp|P62623|ISPH_ECOLI 4-hydroxy-3-methylbut-2-enyl diphosphate reductase OS=Escherichia coli (strain K12) OX=83333 GN=ispH PE=1 SV=1

```
0     MQILLANPRGFCAGVDRAISIVENALAIYGAPIYVRHEVVHNRYVVDSLR

50    ERGAIFIEQISEVPDGAILIFSAHGVSQAVRNEAKSRDLTVFDATCPLVT
                                                ********
100   KVHMEVARASRRGEESILIGHAGHPEVEGTMGQYSNPEGGMYLVESPDDV
      **************
150   WKLTVKNEEKLSFMTQTTLSVDDTSDVIDALRKRFPKIVGPRKDDICYAT

200   TNRQEAVRALAEQAEVVLVVGSKNSSNSNRLAELAQRMGKRAFLIDDAKD

250   IQEEWVKEVKCVGVTAGASAPDILVQNVVARLQQLGGGEAIPLEGREENI

300   VFEVPKELRVDIREVD
```

sp|P0A799|PGK_ECOLI Phosphoglycerate kinase OS=Escherichia coli (strain K12) OX=83333 GN=pgk PE=1 SV=2

```
0     MSVIKMTDLDLAGKRVFIRADLNVPVKDGKVTSDARIRASLPTIELALKQ

50    GAKVMVTSHLGRPTEGEYNEEFSLLPVVNYLKDKLSNPVRLVKDYLDGVD
             ******************************
100   VAEGELVVLENVRFNKGEKKDDETLSKKYAALCDVFVMDAFGTAHRAQAS

150   THGIGKFADVACAGPLLAAELDALGKALKEPARPMVAIVGGSKVSTKLTV

200   LDSLSKIADQLIVGGGIANTFIAAQGHDVGKSLYEADLVDEAKRLLTTCN

250   IPVPSDVRVATEFSETAPATLKSVNDVKADEQILDIGDASAQELAEILKN

300   AKTILWNGPVGVFEFPNFRKGTEIVANAIADSEAFSIAGGGDTLAAIDLF

350   GIADKISYISTGGGAFLEFVEGKVLPAVAMLEERAKK
```

sp|P0AAT9|YBEL_ECOLI Uncharacterized protein YbeL OS=Escherichia coli (strain K12) OX=83333 GN=ybeL PE=4 SV=1

```
0     MNKVAQYYRELVASLSERLRNGERDIDALVEQARERVIKTGELTRTEVDE
         *************************************
50    LTRAVRRDLEEFAMSYEESLKEESDSVFMRVIKESLWQELADITDKTQLE
```

100   WREVFQDLNHHGVYHSGEVVGLGNLVCEKCHFHLPIYTPEVLTLCPKCGH

150   DQFQRRPFEP

sp|P77473|PDEB_ECOLI Probable cyclic di-GMP phosphodiesterase PdeB OS=Escherichia coli (strain K12) OX=83333 GN=pdeB PE=2 SV=2

0   MRTRHLVGLISGVLILSVLLPVGLSIWLAHQQVETSFIEELDTYSSRVAI

50   RANKVATQGKDALQELERWQGAACSEAHLMEMRRVSYSYRYIQEVAYIDN
           ****
100   NVPQCSSLEHESPPDTFPEPGKISKDGYRVWLTSHNDLGIIRYMVAMGTA

150   HYVVMIDPASFIDVIPYSSWQIDAAIIGNAHNVVITSSDEIAQGIITRLQ

200   KTPGEHIENNGIIYDILPLPEMNISIITWASTKMLQKGWHRQVFIWLPLG

250   LVIGLLAAMFVLRILRRIQSPHHRLQDAIENRDICVHYQPIVSLANGKIV

300   GAEALARWPQTDGSWLSPDSFIPLAQQTGLSEPLTLLIIRSVFEDMGDWL

350   RQHPQQHISINLESPVLTSEKIPQLLRDMINHYQVNPRQIALELTEREFA
                                 ****
400   DPKTSAPIISRYREAGHEIYLDDFGTGYSSLSYLQDLDVDILKIDKSFVD
      *****
450   ALEYKNVTPHIIEMAKTLKLKMVAEGIETSKQEEWLRQHGVHYGQGWLYS

500   KALPKEDFLRWAEQHL

sp|P05825|FEPA_ECOLI Ferrienterobactin receptor OS=Escherichia coli (strain K12) OX=83333 GN=fepA PE=1 SV=2

0   MNKKIHSLALLVNLGIYGVAQAQEPTDTPVSHDDTIVVTAAEQNLQAPGV

50   STITADEIRKNPVARDVSKIIRTMPGVNLTGNSTSGQRGNNRQIDIRGMG

100   PENTLILIDGKPVSSRNSVRQGWRGERDTRGDTSWVPPEMIERIEVLRGP

150   AAARYGNGAAGGVVNIITKKGSGEWHGSWDAYFNAPEHKEEGATKRTNFS

200   LTGPLGDEFSFRLYGNLDKTQADAWDINQGHQSARAGTYATTLPAGREGV

250   INKDINGVVRWDFAPLQSLELEAGYSRQGNLYAGDTQNTNSDSYTRSKYG

300   DETNRLYRQNYALTWNGGWDNGVTTSNWVQYEHTRNSRIPEGLAGGTEGK

350   FNEKATQDFVDIDLDDVMLHSEVNLPIDFLVNQTLTLGTEWNQQRMKDLS

400   SNTQALTGTNTGGAIDGVSTTDRSPYSKAEIFSLFAENNMELTDSTIVTP

450   GLRFDHHSIVGNNWSPALNISQGLGDDFTLKMGIARAYKAPSLYQTNPNY
                             ****
500   ILYSKGQGCYASAGGCYLQGNDDLKAETSINKEIGLEFKRDGWLAGVTWF

```
550   RNDYRNKIEAGYVAVGQNAVGTDLYQWDNVPKAVVEGLEGSLNVPVSETV

600   MWTNNITYMLKSENKTTGDRLSIIPEYTLNSTLSWQAREDLSMQTTFTWY

650   GKQQPKKYNYKGQPAVGPETKEISPYSIVGLSATWDVTKNVSLTGGVDNL

700   FDKRLWRAGNAQTTGDLAGANYIAGAGAYTYNEPGRTWYMSVNTHF
```

sp|P63284|CLPB_ECOLI Chaperone protein ClpB OS=Escherichia coli (strain K12) OX=83333 GN=clpB PE=1 SV=1

```
0     MRLDRLTNKFQLALADAQSLALGHDNQFIEPLHLMSALLNQEGGSVSPLL

50    TSAGINAGQLRTDINQALNRLPQVEGTGGDVQPSQDLVRVLNLCDKLAQK

100   RGDNFISSELFVLAALESRGTLADILKAAGATTANITQAIEQMRGGESVN

150   DQGAEDQRQALKKYTIDLTERAEQGKLDPVIGRDEEIRRTIQVLQRRTKN

200   NPVLIGEPGVGKTAIVEGLAQRIINGEVPEGLKGRRVLALDMGALVAGAK

250   YRGEFEERLKGVLNDLAKQEGNVILFIDELHTMVGAGKADGAMDAGNMLK

300   PALARGELHCVGATTLDEYRQYIEKDAALERRFQKVFVAEPSVEDTIAIL
                                                 *********
350   RGLKERYELHHHVQITDPAIVAAATLSHRYIADRQLPDKAIDLIDEAASS
      **************     *******************************
400   IRMQIDSKPEELDRLDRRIIQLKLEQQALMKESDEASKKRLDMLNEELSD
      ****************************
450   KERQYSELEEEWKAEKASLSGTQTIKAELEQAKIAIEQARRVGDLARMSE

500   LQYGKIPELEKQLEAATQLEGKTMRLLRNKVTDAEIAEVLARWTGIPVSR

550   MMESEREKLLRMEQELHHRVIGQNEAVDAVSNAIRRSRAGLADPNRPIGS
                            **********************
600   FLFLGPTGVGKTELCKALANFMFDSDEAMVRIDMSEFMEKHSVSRLVGAP

650   PGYVGYEEGGYLTEAVRRRPYSVILLDEVEKAHPDVFNILLQVLDDGRLT

700   DGQGRTVDFRNTVVIMTSNLGSDLIQERFGELDYAHMKELVLGVVSHNFR

750   PEFINRIDEVVVFHPLGEQHIASIAQIQLKRLYKRLEERGYEIHISDEAL

800   KLLSENGYDPVYGARPLKRAIQQQIENPLAQQILSGELVPGKVIRLEVNE

850   DRIVAVQ
```

sp|P09551|ARGT_ECOLI Lysine/arginine/ornithine-binding periplasmic protein OS=Escherichia coli (strain K12) OX=83333 GN=argT PE=1 SV=3

```
0     MKKSILALSLLVGLSTAASSYAALPETVRIGTDTTYAPFSSKDAKGDFVG
                                  *********************
50    FDIDLGNEMCKRMQVKCTWVASDFDALIPSLKAKKIDAIISSLSITDKRQ
```

100   QEIAFSDKLYAADSRLIAAKGSPIQPTLDSLKGKHVGVLQGSTQEAYANE

150   TWRSKGVDVVAYANQDLVYSDLAAGRLDAALQDEVAASEGFLKQPAGKDF

200   AFAGSSVKDKKYFGDGTGVGLRKDDAELTAAFNKALGELRQDGTYDKMAK

250   KYFDFNVYGD

sp|P31801|CHAA_ECOLI Sodium-potassium/proton antiporter ChaA OS=Escherichia coli (strain K12) OX=83333 GN=chaA PE=1 SV=1

0   MSNAQEAVKTRHKETSLIFPVLALVVLFLWGSSQTLPVVIAINLLALIGI

50   LSSAFSVVRHADVLAHRLGEPYGSLILSLSVVILEVSLISALMATGDAAP

100   TLMRDTLYSIIMIVTGGLVGFSLLLGGRKFATQYMNLFGIKQYLIALFPL
                                                  ****
150   AIIVLVFPMALPAANFSTGQALLVALISAAMYGVFLLIQTKTHQSLFVYE
      ****
200   HEDDSDDDDPHHGKPSAHSSLWHAIWLIIHLIAVIAVTKMNASSLETLLD

250   SMNAPVAFTGFLVALLILSPEGLGALKAVLNNQVQRAMNLFFGSVLATIS

300   LTVPVVTLIAFMTGNELQFALGAPEMVVMVASLVLCHISFSTGRTNVLNG

350   AAHLALFAAYLMTIFA

sp|P0AEH5|ELAB_ECOLI Protein ElaB OS=Escherichia coli (strain K12) OX=83333 GN=elaB PE=1 SV=1
                                            ****
0   MSNQFGDTRIDDDLTLLSETLEEVLRSSGDPADQKYVELKARAEKALDDV
      ****
50   KKRVSQASDSYYYRAKQAVYRADDYVHEKPWQGIGVGAAVGLVLGLLLAR

100   R

sp|P71311|YAIS_ECOLI Uncharacterized deacetylase YaiS OS=Escherichia coli (strain K12) OX=83333 GN=yaiS PE=3 SV=1

0   MDKVLDSALLSSANKRKGILAIGAHPDDIELGCGASLARLAQKGIYIAAV

50   VMTTGNSGTDGIIDRHEESRNALKILGCHQTIHLNFADTRAHLQLNDMIS

100   ALEDIIKNQIPSDVEIMRVYTMHDADRHQDHLAVYQASMVACRTIPQILG
                     ****
150   YETPSTWLSFMPQVFESVKEEYFTVKLAALKKHKS

sp|P45564|YFEN_ECOLI Uncharacterized protein YfeN OS=Escherichia coli (strain K12) OX=83333 GN=yfeN PE=3 SV=1
                                       ****
0   MKKHLLTLTLSSILAIPVVSHAEFKGGFADIGVHYLDWTSRTTEKSSTKS
      ****
50   HKDDFGYLEFEGGANFSWGEMYGFFDWENFYNGRHNKPGSEQRYTFKNTN

```
100   RIYLGDTGFNLYLHAYGTYGSANRVNFHDDMFLYGIGYNFTGSGWWFKPF

150   FAKRYTDQTYYTGDNGYVAGWVAGYNFMLGSEKFTLTNWNEYEFDRDATY

200   AAGNGGKEGLNGAVALWWNATSHITTGIQYRYADDKLGEDFYQDAIIYSI

250   KFNF
```

sp|P0A8T7|RPOC_ECOLI DNA-directed RNA polymerase subunit beta'
OS=Escherichia coli (strain K12) OX=83333 GN=rpoC PE=1 SV=1

```
0     MKDLLKFLKAQTKTEEFDAIKIALASPDMIRSWSFGEVKKPETINYRTFK

50    PERDGLFCARIFGPVKDYECLCGKYKRLKHRGVICEKCGVEVTQTKVRRE

100   RMGHIELASPTAHIWFLKSLPSRIGLLLDMPLRDIERVLYFESYVVIEGG
                                                  ****
150   MTNLERQQILTEEQYLDALEEFGDEFDAKMGAEAIQALLKSMDLEQECEQ
      **********************
200   LREELNETNSETKRKKLTKRIKLLEAFVQSGNKPEWMILTVLPVLPPDLR

250   PLVPLDGGRFATSDLNDLYRRVINRNNRLKRLLDLAAPDIIVRNEKRMLQ

300   EAVDALLDNGRRGRAITGSNKRPLKSLADMIKGKQGRFRQNLLGKRVDYS

350   GRSVITVGPYLRLHQCGLPKKMALELFKPFIYGKLELRGLATTIKAAKKM

400   VEREEAVVWDILDEVIREHPVLLNRAPTLHRLGIQAFEPVLIEGKAIQLH

450   PLVCAAYNADFDGDQMAVHVPLTLEAQLEARALMMSTNNILSPANGEPII

500   VPSQDVVLGLYYMTRDCVNAKGEGMVLTGPKEAERLYRSGLASLHARVKV

550   RITEYEKDANGELVAKTSLKDTTVGRAILWMIVPKGLPYSIVNQALGKKA
                                            **********
600   ISKMLNTCYRILGLKPTVIFADQIMYTGFAYAARSGASVGIDDMVIPEKK
      *****************
650   HEIISEAEAEVAEIQEQFQSGLVTAGERYNKVIDIWAAANDRVSKAMMDN

700   LQTETVINRDGQEEKQVSFNSIYMMADSGARGSAAQIRQLAGMRGLMAKP

750   DGSIIETPITANFREGLNVLQYFISTHGARKGLADTALKTANSGYLTRRL

800   VDVAQDLVVTEDDCGTHEGIMMTPVIEGGDVKEPLRDRVLGRVTAEDVLK

850   PGTADILVPRNTLLHEQWCDLLEENSVDAVKVRSVVSCDTDFGVCAHCYG
                                                 *****
900   RDLARGHIINKGEAIGVIAAQSIGEPGTQLTMRTFHIGGAASRAAAESSI
      **********************
950   QVKNKGSIKLSNVKSVVNSSGKLVITSRNTELKLIDEFGRTKESYKVPYG

1000  AVLAKGDGEQVAGGETVANWDPHTMPVITEVSGFVRFTDMIDGQTITRQT
```

1050 DELTGLSSLVVLDSAERTAGGKDLRPALKIVDAQGNDVLIPGTDMPAQYF

1100 LPGKAIVQLEDGVQISSGDTLARIPQESGGTKDITGGLPRVADLFEARRP

1150 KEPAILAEISGIVSFGKETKGKRRLVITPVDGSDPYEEMIPKWRQLNVFE

1200 GERVERGDVISDGPEAPHDILRLRGVHAVTRYIVNEVQDVYRLQGVKIND

1250 KHIEVIVRQMLRKATIVNAGSSDFLEGEQVEYSRVKIANRELEANGKVGA

1300 TYSRDLLGITKASLATESFISAASFQETTRVLTEAAVAGKRDELRGLKEN

1350 VIVGRLIPAGTGYAYHQDRMRRRAAGEAPAAPQVTAEDASASLAELLNAG

1400 LGGSDNE

sp|P31550|THIB_ECOLI Thiamine-binding periplasmic protein OS=Escherichia coli (strain K12) OX=83333 GN=thiB PE=1 SV=2

0   MLKKCLPLLLLCTAPVFAKPVLTVYTYDSFAADWGPGPVVKKAFEADCNC

50   ELKLVALEDGVSLLNRLRMEGKNSKADVVLGLDNNLLDAASKTGLFAKSG
                                  ****
100   VAADAVNVPGGWNNDTFVPFDYGYFAFVYDKNKLKNPPQSLKELVESDQN

150   WRVIYQDPRTSTPGLGLLLWMQKVYGDDAPQAWQKLAKKTVTVTKGWSEA

200   YGLFLKGESDLVLSYTTSPAYHILEEKKDNYAAANFSEGHYLQVEVAART

250   AASKQPELAQKFLQFMVSPAFQNAIPTGNWMYPVANVTLPAGFEKLTKPA

300   TTLEFTPAEVAAQRQAWISEWQRAVSR

sp|P24202|MRR_ECOLI Mrr restriction system protein OS=Escherichia coli (strain K12) OX=83333 GN=mrr PE=1 SV=1

0   MTVPTYDKFIEPVLRYLATKPEGAAARDVHEAAADALGLDDSQRAKVITS

50   GQLVYKNRAGWAHDRLKRAGLSQSLSRGKWCLTPAGFDWVASHPQPMTEQ
                 ****
100   ETNHLAFAFVNVKLKSRPDAVDLDPKADSPDHEELAKSSPDDRLDQALKE
      ***
150   LRDAVADEVLENLLQVSPSRFEVIVLDVLHRLGYGGHRDDLQRVGGTGDG

200   GIDGVISLDKLGLEKVYVQAKRWQNTVGRPELQAFYGALAGQKAKRGVFI

250   TTSGFTSQARDFAQSVEGMVLVDGERLVHLMIENEVGVSSRLLKVPKLDM

300   DYFE

sp|P10121|FTSY_ECOLI Signal recognition particle receptor FtsY OS=Escherichia coli (strain K12) OX=83333 GN=ftsY PE=1 SV=1

0   MAKEKKRGFFSWLGFGQKEQTPEKETEVQNEQPVVEEIVQAQEPVKASEQ

```
50    AVEEQPQAHTEAEAETFAADVVEVTEQVAESEKAQPEAEVVAQPEPVVEE
                                   *********************
100   TPEPVAIEREELPLPEDVNAEAVSPEEWQAEAETVEIVEAAEEEAAKEEI
      ****************       *********************
150   TDEELETALAAEAAEEAVMVVPPAEEEQPVEEIAQEQEKPTKEGFFARLK
                   ***************************
200   RSLLKTKENLGSGFISLFRGKKIDDDLFEELEEQLLIADVGVETTRKIIT

250   NLTEGASRKQLRDAEALYGLLKEEMGEILAKVDEPLNVEGKAPFVILMVG

300   VNGVGKTTTIGKLARQFEQQGKSVMLAAGDTFRAAAVEQLQVWGQRNNIP

350   VIAQHTGADSASVIFDAIQAAKARNIDVLIADTAGRLQNKSHLMEELKKI

400   VRVMKKLDVEAPHEVMLTIDASTGQNAVSQAKLFHEAVGLTGITLTKLDG

450   TAKGGVIFSVADQFGIPIRYIGVGERIEDLRPFKADDFIEALFARED
```

```
sp|P37769|KDUD_ECOLI 2-dehydro-3-deoxy-D-gluconate 5-dehydrogenase
OS=Escherichia coli (strain K12) OX=83333 GN=kduD PE=1 SV=2

0     MILSAFSLEGKVAVVTGCDTGLGQGMALGLAQAGCDIVGINIVEPTETIE
                                                      **
50    QVTALGRRFLSLTADLRKIDGIPALLDRAVAEFGHIDILVNNAGLIRRED
      *******************
100   ALEFSEKDWDDVMNLNIKSVFFMSQAAAKHFIAQGNGGKIINIASMLSFQ

150   GGIRVPSYTASKSGVMGVTRLMANEWAKHNINVNAIAPGYMATNNTQQLR

200   ADEQRSAEILDRIPAGRWGLPSDLMGPIVFLASSASDYVNGYTIAVDGGW

250   LAR
```

```
sp|P0ABD8|BCCP_ECOLI Biotin carboxyl carrier protein of acetyl-CoA
carboxylase OS=Escherichia coli (strain K12) OX=83333 GN=accB PE=1 SV=1
      ***************************
0     MDIRKIKKLIELVEESGISELEISEGEESVRISRAAPAASFPVMQQAYAA

50    PMMQQPAQSNAAAPATVPSMEAPAAAEISGHIVRSPMVGTFYRTPSPDAK

100   AFIEVGQKVNVGDTLCIVEAMKMMNQIEADKSGTVKAILVESGQPVEFDE

150   PLVVIE
```

```
sp|P0CE47|EFTU1_ECOLI Elongation factor Tu 1 OS=Escherichia coli (strain
K12) OX=83333 GN=tufA PE=1 SV=1

0     MSKEKFERTKPHVNVGTIGHVDHGKTTLTAAITTVLAKTYGGAARAFDQI

50    DNAPEEKARGITINTSHVEYDTPTRHYAHVDCPGHADYVKNMITGAAQMD
                                          **************
100   GAILVVAATDGPMPQTREHILLGRQVGVPYIIVFLNKCDMVDDEELLELV
      ******
```

```
150   EMEVRELLSQYDFPGDDTPIVRGSALKALEGDAEWEAKILELAGFLDSYI

200   PEPERAIDKPFLLPIEDVFSISGRGTVVTGRVERGIIKVGEEVEIVGIKE

250   TQKSTCTGVEMFRKLLDEGRAGENVGVLLRGIKREEIERGQVLAKPGTIK

300   PHTKFESEVYILSKDEGGRHTPFFKGYRPQFYFRTTDVTGTIELPEGVEM

350   VMPGDNIKMVVTLIHPIAMDDGLRFAIREGGRTVGAGVVAKVLG
```

sp|P0AG84|YGHA_ECOLI Uncharacterized oxidoreductase YghA OS=Escherichia coli (strain K12) OX=83333 GN=yghA PE=1 SV=1

```
0     MSHLKDPTTQYYTGEYPKQKQPTPGIQAKMTPVPDCGEKTYVGSGRLKDR
                                 ***********************
50    KALVTGGDSGIGRAAAIAYAREGADVAISYLPVEEEDAQDVKKIIEECGR
      ***
100   KAVLLPGDLSDEKFARSLVHEAHKALGGLDIMALVAGKQVAIPDIADLTS

150   EQFQKTFAINVFALFWLTQEAIPLLPKGASIITTSSIQAYQPSPHLLDYA

200   ATKAAILNYSRGLAKQVAEKGIRVNIVAPGPIWTALQISGGQTQDKIPQF

250   GQQTPMKRAGQPAELAPVYVYLASQESSYVTAEVHGVCGGEHLG
```

sp|P0AG14|SOHB_ECOLI Probable protease SohB OS=Escherichia coli (strain K12) OX=83333 GN=sohB PE=1 SV=1

```
0     MELLSEYGLFLAKIVTVVLAIAAIAAIIVNVAQRNKRQRGELRVNNLSEQ
                           ************************
50    YKEMKEELAAALMDSHQQKQWHKAQKKKHKQEAKAAKAKAKLGEVATDSK

100   PRVWVLDFKGSMDAHEVNSLREEITAVLAAFKPQDQVVLRLESPGGMVHG

150   YGLAASQLQRLRDKNIPLTVTVDKVAASGGYMMACVADKIVSAPFAIVGS

200   IGVVAQMPNFNRFLKSKDIDIELHTAGQYKRTLTLLGENTEEGREKFREE

250   LNETHQLFKDFVKRMRPSLDIEQVATGEHWYGQQAVEKGLVDEINTSDEV

300   ILSLMEGREVVNVRYMQRKRLIDRFTGSAAESADRLLLRWWQRGQKPLM
```

sp|P75838|YCAO_ECOLI Ribosomal protein S12 methylthiotransferase accessory factor YcaO OS=Escherichia coli (strain K12) OX=83333 GN=ycaO PE=1 SV=2

```
0     MTQTFIPGKDAALEDSIARFQQKLSDLGFQIEEASWLNPVPNVWSVHIRD

50    KECALCFTNGKGATKKAALASALGEYFERLSTNYFFADFWLGETIANGPF

100   VHYPNEKWFPLTENDDVPEGLLDDRLRAFYDPENELTGSMLIDLQSGNED

150   RGICGLPFTRQSDNQTVYIPMNIIGNLYVSNGMSAGNTRNEARVQGLSEV
```

```
200   FERYVKNRIIAESISLPEIPADVLARYPAVVEAIETLEAEGFPIFAYDGS

250   LGGQYPVICVVLFNPANGTCFASFGAHPDFGVALERTVTELLQGRGLKDL

300   DVFTPPTFDDEEVAEHTNLETHFIDSSGLISWDLFKQDADYPFVDWNFSG

350   TTEEEFATLMAIFNKEDKEVYIADYEHLGVYACRIIVPGMSDIYPAEDLW
                         *************************
400   LANNSMGSHLRETILSLPGSEWEKEDYLNLIEQLDEEGFDDFTRVRELLG

450   LATGSDNGWYTLRIGELKAMLALAGGDLEQALVWTEWTMEFNSSVFSPER

500   ANYYRCLQTLLLLAQEEDRQPLQYLNAFVRMYGADAVEAASAAMSGEAAF

550   YGLQPVDSDLHAFAAHQSLLKAYEKLQRAKAAFWAK
```

sp|P0A8N5|SYK2_ECOLI Lysine--tRNA ligase, heat inducible OS=Escherichia coli (strain K12) OX=83333 GN=lysU PE=1 SV=2

```
                                                ********
0     MSEQETRGANEAIDFNDELRNRREKLAALRQQGVAFPNDFRRDHTSDQLH
      ********************
50    EEFDAKDNQELESLNIEVSVAGRMMTRRIMGKASFVTLQDVGGRIQLYVA

100   RDSLPEGVYNDQFKKWDLGDIIGARGTLFKTQTGELSIHCTELRLLTKAL

150   RPLPDKFHGLQDQEVRYRQRYLDLIANDKSRQTFVVRSKILAAIRQFMVA

200   RGFMEVETPMMQVIPGGASARPFITHHNALDLDMYLRIAPELYLKRLVVG

250   GFERVFEINRNFRNEGISVRHNPEFTMMELYMAYADYHDLIELTESLFRT

300   LAQEVLGTTKVTYGEHVFDFGKPFEKLTMREAIKKYRPETDMADLDNFDA

350   AKALAESIGITVEKSWGLGRIVTEIFDEVAEAHLIQPTFITEYPAEVSPL

400   ARRNDVNPEITDRFEFFIGGREIGNGFSELNDAEDQAERFQEQVNAKAAG

450   DDEAMFYDEDYVTALEYGLPPTAGLGIGIDRMIMLFTNSHTIRDVILFPA

500   MRPQK
```

sp|P0A6F9|CH10_ECOLI 10 kDa chaperonin OS=Escherichia coli (strain K12) OX=83333 GN=groS PE=1 SV=1

```
0     MNIRPLHDRVIVKRKEVETKSAGGIVLTGSAAAKSTRGEVLAVGNGRILE
                        **************************
50    NGEVKPLDVKVGDIVIFNDGYGVKSEKIDNEEVLIMSESDILAIVEA
```

sp|P39336|YJGL_ECOLI Uncharacterized protein YjgL OS=Escherichia coli (strain K12) OX=83333 GN=yjgL PE=1 SV=2

```
0     MSKISDLNYSQHITLADNFKQKSEVLNTWRVGMNDFARIAGGQDNRRNIL
                                     *******************
50    SPGAFLEFLAKIFTLGYVDFSKRSNEAGRNMMAHIKSSSYSKDTNGNEKM
```

```
      *****                                    *********
100   KFYMNNPVGERADSPKVIIEISLSTITTMGTRQGHTAIIFPQPDGSTNRY
      ********************
150   EGKSFERKDESSLHLITNKVLACYQSEANKKIARLLNNNQELNNLQKLNN

200   LQKLNNLLKLNNIQGLNNPQELNNPQNLNDSQELNNSQELNSPQELNDPQ
                                                       *
250   ELNNSQDLNNSKVSCTVSVDSTITGLLKEPLNNALLAIRNEHLLLMPHVC
      ********************
300   DESISYLLGEKGILEEIDKLYALNDHGIDNDKVGNNEINDIKVNLSHILI

350   DSLDDAKVNLTPVIDSILETFSKSPYINDVRILDWCFNKSMQYFDDTKKI

400   KHACSVINHINLRSDQSKIAETLFFNLDKEPYKNSPELQGLIWNKLVVYV

450   NEFNLSNREKTNLIQRLFDNVESIFNEVPVSILVNDIFMNDFFMKNPEMI
                 ***************************************
500   NWYFPQLLKSYEGEKIYFDNLKYDLNDNDKESNKEILKNQPDNVIKEKLN

550   NEYKLRFRMMQTILQSRVNVLPYINEQRLNKLNPPENLRIAIEHFGWKNR

600   PITA
```

sp|P37766|YDIF_ECOLI Acetate CoA-transferase YdiF OS=Escherichia coli (strain K12) OX=83333 GN=ydiF PE=3 SV=3

```
0     MKPVKPPRINGRVPVLSAQEAVNYIPDEATLCVLGAGGGILEATTLITAL

50    ADKYKQTQTPRNLSIISPTGLGDRADRGISPLAQEGLVKWALCGHWGQSP

100   RISELAEQNKIIAYNYPQGVLTQTLRAAAAHQPGIISDIGIGTFVDPRQQ
        ********************
150   GGKLNEVTKEDLIKLVEFDNKEYLYYKAIAPDIAFIRATTCDSEGYATFE

200   DEVMYLDALVIAQAVHNNGGIVMMQVQKMVKKATLHPKSVRIPGYLVDIV

250   VVDPDQTQLYGGAPVNRFISGDFTLDDSTKLSLPLNQRKLVARRALFEMR

300   KGAVGNVGVGIADGIGLVAREEGCADDFILTVETGPIGGITSQGIAFGAN

350   VNTRAILDMTSQFDFYHGGGLDVCYLSFAEVDQHGNVGVHKFNGKIMGTG

400   GFIDISATSKKIIFCGTLTAGSLKTEITDGKLNIVQEGRVKKFIRELPEI

450   TFSGKIALERGLDVRYITERAVFTLKEDGLHLIEIAPGVDLQKDILDKMD

500   FTPVISPELKLMDERLFIDAAMGFVLPEAAH
```

sp|P76298|FLHA_ECOLI Flagellar biosynthesis protein FlhA OS=Escherichia coli (strain K12) OX=83333 GN=flhA PE=3 SV=1

```
0     MSNLAAMLRLPANLKSTQWQILAGPILILLILSMMVLPLPAFILDLLFTF

50    NIALSIMVLLVAMFTQRTLEFAAFPTILLFTTLLRLALNVASTRIILMEG
```

```
100   HTGAAAAGKVVEAFGHFLVGGNFAIGIVVFVILVIINFMVITKGAGRIAE
                                 ********************
150   VGARFVLDGMPGKQMAIDADLNAGLIGEDEAKKRRSEVTQEADFYGSMDG

200   ASKFVRGDAIAGILIMVINIVGGLLVGVLQHGMSMGHAAESYTLLTIGDG

250   LVAQIPALVISTAAGVIVTRVSTDQDVGEQMVNQLFSNPSVMLLSAAVLG

300   LLGLVPGMPNLVFLLFTAGLLGLAWWIRGREQKAPAEPKPVKMAENNTVV

350   EATWNDVQLEDSLGMEVGYRLIPMVDFQQDGELLGRIRSIRKKFAQEMGF

400   LPPVVHIRDNMDLQPARYRILMKGVEIGSGDAYPGRWLAINPGTAAGTLP

450   GEATVDPAFGLNAIWIESALKEQAQIQGYTVVEASTVVATHLNHLISQHA

500   AELFGRQEAQQLLDRVAQEMPKLTEDLVPGVVTLTTLHKVLQNLLDEKVP

550   IRDMRTILETLAEHAPIQSDPHELTAVVRVALGRAITQQWFPGKDEVHVI

600   GLDTPLERLLLQALQGGGGLEPGLADRLLAQTQEALSRQEMLGAPPVLLV

650   NHALRPLLSRFLRRSLPQLVVLSNLELSDNRHIRMTATIGGK
```

sp|P08395|SPPA_ECOLI Protease 4 OS=Escherichia coli (strain K12) OX=83333 GN=sppA PE=1 SV=2

```
0     MRTLWRFIAGFFKWTWRLLNFVREMVLNLFFIFLVLVGVGIWMQVSGGDS
                 ********************
50    KETASRGALLLDISGVIVDKPDSSQRFSKLSRQLLGASSDRLQENSLFDI

100   VNTIRQAKDDRNITGIVMDLKNFAGGDQPSMQYIGKALKEFRDSGKPVYA

150   VGENYSQGQYYLASFANKIWLSPQGVVDLHGFATNGLYYKSLLDKLKVST

200   HVFRVGTYKSAVEPFIRDDMSPAAREADSRWIGELWQNYLNTVAANRQIP
                                         ***************
250   AEQVFPGAQGLLEGLTKTGGDTAKYALENKLVDALASSAEIEKALTKEFG
      *******
300   WSKTDKNYRAISYYDYALKTPADTGDSIGVVFANGAIMDGEETQGNVGGD

350   TTAAQIRDARLDPKVKAIVLRVNSPGGSVTASEVIRAELAAARAAGKPVV

400   VSMGGMAASGGYWISTPANYIVANPSTLTGSIGIFGVITTVENSLDSIGV

450   HTDGVSTSPLADVSITRALPPEAQLMMQLSIENGYKRFITLVADARHSTP

500   EQIDKIAQGHVWTGQDAKANGLVDSLGDFDDAVAKAAELAKVKQWHLEYY

550   VDEPTFFDKVMDNMSGSVRAMLPDAFQAMLPAPLASVASTVKSESDKLAA

600   FNDPQNRYAFCLTCANMR
```

sp|P0A6Z3|HTPG_ECOLI Chaperone protein HtpG OS=Escherichia coli (strain K12) OX=83333 GN=htpG PE=1 SV=1

```
0     MKGQETRGFQSEVKQLLHLMIHSLYSNKEIFLRELISNASDAADKLRFRA
                                                  ****
50    LSNPDLYEGDGELRVRVSFDKDKRTLTISDNGVGMTRDEVIDHLGTIAKS
      *******************
100   GTKSFLESLGSDQAKDSQLIGQFGVGFYSAFIVADKVTVRTRAAGEKPEN
                                     *******************
150   GVFWESAGEGEYTVADITKEDRGTEITLHLREGEDEFLDDWRVRSIISKY
      *******************************     **************
200   SDHIALPVEIEKREEKDGETVISWEKINKAQALWTRNKSEITDEEYKEFY
      *******
250   KHIAHDFNDPLTWSHNRVEGKQEYTSLLYIPSQAPWDMWNRDHKHGLKLY

300   VQRVFIMDDAEQFMPNYLRFVRGLIDSSDLPLNVSREILQDSTVTRNLRN

350   ALTKRVLQMLEKLAKDDAEKYQTFWQQFGLVLKEGPAEDFANQEAIAKLL

400   RFASTHTDSSAQTVSLEDYVSRMKEGQEKIYYITADSYAAAKSSPHLELL
                           *****************************
450   RKKGIEVLLLSDRIDEWMMNYLTEFDGKPFQSVSKVDESLEKLADEVDES
      **************
500   AKEAEKALTPFIDRVKALLGERVKDVRLTHRLTDTPAIVSTDADEMSTQM

550   AKLFAAAGQKVPEVKYIFELNPDHVLVKRAADTEDEAKFSEWVELLLDQA

600   LLAERGTLEDPNLFIRRMNQLLVS
```

sp|P0DMC9|RCSA_ECOLI Transcriptional regulatory protein RcsA OS=Escherichia coli (strain K12) OX=83333 GN=rcsA PE=1 SV=1

```
0     MSTIIMDLCSYTRLGLTGYLLSRGVKKREINDIETVDDLAIACDSQRPSV
                                                 *****
50    VFINEDCFIHDASNSQRIKLIINQHPNTLFIVFMAIANVHFDEYLLVRKN
      **************************
100   LLISSKSIKPESLDDILGDILKKETTITSFLNMPTLSLSRTESSMLRMWM

150   AGQGTIQISDQMNIKAKTVSSHKGNIKRKIKTHNKQVIYHVVRLTDNVTN

200   GIFVNMR
```

sp|P06720|AGAL_ECOLI Alpha-galactosidase OS=Escherichia coli (strain K12) OX=83333 GN=melA PE=1 SV=1

```
0     MMSAPKITFIGAGSTIFVKNILGDVFHREALKTAHIALMDIDPTRLEESH

50    IVVRKLMDSAGASGKITCHTQQKEALEDADFVVVAFQIGGYEPCTVTDFE

100   VCKRHGLEQTIADTLGPGGIMRALRTIPHLWQICEDMTEVCPDATMLNYV

150   NPMAMNTWAMYARYPHIKQVGLCHSVQGTAEELARDLNIDPATLRYRCAG

200   INHMAFYLELERKTADGSYVNLYPELLAAYEAGQAPKPNIHGNTRCQNIV
```

250 RYEMFKKLGYFVTESSEHFAEYTPWFIKPGREDLIERYKVPLDEYPKRCV
****
300 EQLANWHKELEEYKKASRIDIKPSREYASTIMNAIWTGEPSVIYGNVRND

350 GLIDNLPQGCCVEVACLVDANGIQPTKVGTLPSHLAALMQTNINVQTLLT

400 EAILTENRDRVYHAAMMDPHTAAVLGIDEIYALVDDLIAAHGDWLPGWLH

450 R

sp|P76235|YEAH_ECOLI UPF0229 protein YeaH OS=Escherichia coli (strain K12) OX=83333 GN=yeaH PE=3 SV=1
****
0 MTWFIDRRLNGKNKSMVNRQRFLRRYKAQIKQSISEAINKRSVTDVDSGE
****
50 SVSIPTEDISEPMFHQGRGGLRHRVHPGNDHFVQNDRIERPQGGGGGSGS

100 GQGQASQDGEGQDEFVFQISKDEYLDLLFEDLALPNLKQNQQRQLTEYKT

150 HRAGYTANGVPANISVVRSLQNSLARRTAMTAGKRRELHALEENLAIISN

200 SEPAQLLEEERLRKEIAELRAKIERVPFIDTFDLRYKNYEKRPDPSSQAV

250 MFCLMDVSGSMDQSTKDMAKRFYILLYLFLSRTYKNVEVVYIRHHTQAKE

300 VDEHEFFYSQETGGTIVSSALKLMDEVVKERYNPAQWNIYAAQASDGDNW

350 ADDSPLCHEILAKKLLPVVRYYSYIEITRRAHQTLWREYEHLQSTFDNFA

400 MQHIRDQDDIYPVFRELFHKQNATAKG

sp|P75617|YAAW_ECOLI UPF0174 protein YaaW OS=Escherichia coli (strain K12) OX=83333 GN=yaaW PE=3 SV=1

0 MNVNYLNDSDLDFLQHCSEEQLANFARLLTHNEKGKTRLSSVLMRNELFK
****
50 SMEGHPEQHRRNWQLIAGELQHFGGDSIANKLRGHGKLYRAILLDVSKRL
****
100 KLKADKEMSTFEIEQQLLEQFLRNTWKKMDEEHKQEFLHAVDARVNELEE

150 LLPLLMKDKLLAKGVSHLLSSQLTRILRTHAAMSVLGHGLLRGAGLGGPV

200 GAALNGVKAVSGSAYRVTIPAVLQIACLRRMVSATQV

sp|P60584|CAIA_ECOLI Crotonobetainyl-CoA reductase OS=Escherichia coli (strain K12) OX=83333 GN=caiA PE=1 SV=1

0 MDFNLNDEQELFVAGIRELMASENWEAYFAECDRDSVYPERFVKALADMG

50 IDSLLIPEEHGGLDAGFVTLAAVWMELGRLGAPTYVLYQLPGGFNTFLRE

100 GTQEQIDKIMAFRGTGKQMWNSAITEPGAGSDVGSLKTTYTRRNGKIYLN

150   GSKCFITSSAYTPYIVVMARDGASPDKPVYTEWFVDMSKPGIKVTKLEKL
       ****
200   GLRMDSCCEITFDDVELDEKDMFGREGNGFNRVKEEFDHERFLVALTNYG

250   TAMCAFEDAARYANQRVQFGEAIGRFQLIQEKFAHMAIKLNSMKNMLYEA

300   AWKADNGTITSGDAAMCKYFCANAAFEVVDSAMQVLGGVGIAGNHRISRF

350   WRDLRVDRVSGGSDEMQILTLGRAVLKQYR

sp|P32694|YJBM_ECOLI Uncharacterized protein YjbM OS=Escherichia coli (strain K12) OX=83333 GN=yjbM PE=4 SV=2

0   MWVNKYIDDCTDEDLNDRDFIASVVDRAIFHFAINSICNPGDNKDAMPIE

50   QCTFDVETKNDLPSTVQLFYEESKDNEPLANIHFQAIGSGFLTFVNACQE

100   HDDNSLKLFASLLISLSYSSAYADLSETVYINENNESYLKAQFEKLSQRD

150   MKKYLGEMKRLADGGEMNFDGYLDKMSHLVNEGTLDPDILSKMRDAAPQL
      ****
200   ISFAKSFDPTSKEEIKILTDTSKLIYDLFGVKSEK

sp|P0A9M2|HPRT_ECOLI Hypoxanthine phosphoribosyltransferase OS=Escherichia coli (strain K12) OX=83333 GN=hpt PE=1 SV=1

0   MKHTVEVMIPEAEIKARIAELGRQITERYKDSGSDMVLVGLLRGSFMFMA
                              ****
50   DLCREVQVSHEVDFMTASSYGSGMSTTRDVKILKDLDEDIRGKDVLIVED
      ****
100   IIDSGNTLSKVREILSLREPKSLAICTLLDKPSRREVNVPVEFIGFSIPD

150   EFVVGYGIDYAQRYRHLPYIGKVILLDE

sp|P37147|FXSA_ECOLI UPF0716 protein FxsA OS=Escherichia coli (strain K12) OX=83333 GN=fxsA PE=1 SV=2

0   MRWLPFIAIFLYVYIEISIFIQVAHVLGVLLTLVLVIFTSVIGMSLVRNQ

50   GFKNFVLMQQKMAAGENPAAEMIKSVSLIIAGLLLLLPGFFTDFLGLLLL
                                        ****
100   LPPVQKHLTVKLMPHLRFSRMPGGGFSAGTGGGNTFDGEYQRKDDERDRL
      ****
150   DHKDDRQD

sp|P69797|PTNAB_ECOLI PTS system mannose-specific EIIAB component OS=Escherichia coli (strain K12) OX=83333 GN=manX PE=1 SV=2

0   MTIAIVIGTHGWAAEQLLKTAEMLLGEQENVGWIDFVPGENAETLIEKYN

50   AQLAKLDTTKGVLFLVDTWGGSPFNAASRIVVDKEHYEVIAGVNIPMLVE

100   TLMARDDDPSFDELVALAVETGREGVKALKAKPVEKAAPAPAAAAPKAAP

```
150   TPAKPMGPNDYMVIGLARIDDRLIHGQVATRWTKETNVSRIIVVSDEVAA

200   DTVRKTLLTQVAPPGVTAHVVDVAKMIRVYNNPKYAGERVMLLFTNPTDV

250   ERLVEGGVKITSVNVGGMAFRQGKTQVNNAVSVDEKDIEAFKKLNARGIE
         ********************
300   LEVRKVSTDPKLKMMDLISKIDK
```

```
sp|Q47013|ELAD_ECOLI Protease ElaD OS=Escherichia coli (strain K12)
OX=83333 GN=elaD PE=1 SV=3
                                ***********************
0     MMVTVVSNYCQLSQTQLSQTFAEKFTVTEELLQSLKKTALSGDEESIELL

50    HNIALGYDKFGKEAEDILYHIVRTPTNETLSIIRLIKNACLKLYNLAHIA
      **************************   *********************
100   TNSPLKSHDSDDLLFKKLFSPSKLMTIIGDEIPLISEKQSLSKVLLNDEN
      ******
150   NELSDGTNFWDKNRQLTTDEIACYLQKIAANAKNTQVNYPTGLYVPYSTR

200   THLEDALNENIKSDPSWPNEVQLFPINTGGHWILVSLQKIVNKKNNKLQI

250   KCVIFNSLRALGYDKENSLKRVINSFNSELMGEMSNNNIKVHLNEPEIIF

300   LHADLQQYLSQSCGAFVCMAAQEVIEQRESNSDSAPYTLLKNHADRFKKY

350   SAEEQYEIDFQHRLANRNCYLDKYGDANINHYYRNLEIKHSQPKNRASGK

400   RVS
```

```
sp|P00550|PTM3C_ECOLI PTS system mannitol-specific EIICBA component
OS=Escherichia coli (strain K12) OX=83333 GN=mtlA PE=1 SV=1

0     MSSDIKIKVQSFGRFLSNMVMPNIGAFIAWGIITALFIPTGWLPNETLAK

50    LVGPMITYLLPLLIGYTGGKLVGGERGGVVGAITTMGVIVGADMPMFLGS

100   MIAGPLGGWCIKHFDRWVDGKIKSGFEMLVNNFSAGIIGMILAILAFLGI

150   GPIVEALSKMLAAGVNFMVVHDMLPLASIFVEPAKILFLNNAINHGIFSP

200   LGIQQSHELGKSIFFLIEANPGPGMGVLLAYMFFGRGSAKQSAGGAAIIH

250   FLGGIHEIYFPYVLMNPRLILAVILGGMTGVFTLTILGGGLVSPASPGSI
                              **************************
300   LAVLAMTPKGAYFANIAGVCAAMAVSFVVSAILLKTSKVKEEDDIEAATR
      **********
350   RMQDMKAESKGASPLSAGDVTNDLSHVRKIIVACDAGMGSSAMGAGVLRK

400   KIQDAGLSQISVTNSAINNLPPDVDLVITHRDLTERAMRQVPQAQHISLT
                       *********************************
450   NFLDSGLYTSLTERLVAAQRHTANEEKVKDSLKDSFDDSSANLFKLGAEN

500   IFLGRKAATKEEAIRFAGEQLVKGGYVEPEYVQAMLDREKLTPTYLGESI
```

```
550   AVPHGTVEAKDRVLKTGVVFCQYPEGVRFGEEEDDIARLVIGIAARNNEH

600   IQVITSLTNALDDESVIERLAHTTSVDEVLELLAGRK
```

sp|P45522|KEFB_ECOLI Glutathione-regulated potassium-efflux system protein KefB OS=Escherichia coli (strain K12) OX=83333 GN=kefB PE=3 SV=1

```
0     MEGSDFLLAGVLFLFAAVAAVPLASRLGIGAVLGYLLAGIAIGPWGLGFI

50    SDVDEILHFSELGVVFLMFIIGLELNPSKLWQLRRSIFGVGAAQVLLSAA

100   LLAGLLMLTDFAWQAAVVGGIGLAMSSTAMALQLMREKGMNRSESGQLGF

150   SVLLFQDLAVIPALALVPLLAGSADEHFDWMKVGMKVLAFVGMLIGGRYL

200   LRPVFRFIAASGVREVFTAATLLLVLGSALFMDALGLSMALGTFIAGVLL

250   AESEYRHELETAIDPFKGLLLGLFFISVGMSLNLGVLYTHLLWVVISVVV

300   LVAVKILVLYLLARLYGVRSSERMQFAGVLSQGGEFAFVLFSTASSQRLF
                             ***************************
350   QGDQMALLLVTVTLSMMTTPLLMKLVDKWLSRQFNGPEEEDEKPWVNDDK

400   PQVIVVGFGRFGQVIGRLLMANKMRITVLERDISAVNLMRKYGYKVYYGD

450   ATQVDLLRSAGAEAAESIVITCNEPEDTMKLVEICQQHFPHLHILARARG

500   RVEAHELLQAGVTQFSRETFSSALELGRKTLVTLGMHPHQAQRAQLHFRR

550   LDMRMLRELIPMHADTVQISRAREARRELEEIFQREMQQERRQLDGWDEF

600   E
```

sp|P52124|YFJI_ECOLI Protein YfjI OS=Escherichia coli (strain K12) OX=83333 GN=yfjI PE=4 SV=1

```
0     MFNGRPFPVDAFPKIIRNAIYEVEQHTQAPQGLIAASALGVISLACQNRI

50    DVCRLNNLRGPVSLFLMTLAESGERKSTVDKLLMKPLYQLEEDLFEKYTH
            ****************************
100   DLTAWRNDEAIFNIEKKALMSKLKSDIRRNKDHLATNERLKELLTTNPKA

150   PVRFKFLFNDATPAAIKAHLCGHWRSVGIMSDEAGIIFNGYTLNELPFIN

200   KMWDGSIFTVERKNEPEKLIRDARITLSLMVQPNVFKGYIDRKGDMAKGI

250   GFFARCLMCQPASTQGNRKISNPIFSNEHLPVFHQRLMEIVNESIIKINE

300   NNRICLRFSAEAERHWIEFYNQVESEMRMIGLLYDFKDYASKMAENMARL
                                  **********************
350   AALLHYFSGDGGDISVTAVKAAVEIVAWYIEEYIRLFSKKEEFSLDVSEA
      **********
400   DELYCWIKDYCTQKFSSCIKKNIILQFGPNKFRNRDKANELIRILISQNK
```

```
450   IFISSWGKTKIININITHCVF
```

sp|P42914|YRAI_ECOLI Probable fimbrial chaperone YraI OS=Escherichia coli (strain K12) OX=83333 GN=yraI PE=2 SV=1

```
0     MSKRTFAVILTLLCSFCIGQALAGGIVLQRTRVIYDASRKEAALPVANKG
                                        ****************
50    AETPYLLQSWVDNIDGKSRAPFIITPPLFRLEAGDDSSLRIIKTADNLPE
      ****
100   NKESLFYINVRAIPAKKKSDDVNANELTLVFKTRIKMFYRPAHLKGRVND

150   AWKSLEFKRSDHSLNIYNPTEYYVVFAGLAVDKTDLTSKIEYIAPGEHKQ

200   LPLPASGGKNVKWAAINDYGGSSGTETRPLQ
```

sp|Q46899|CASC_ECOLI CRISPR system Cascade subunit CasC OS=Escherichia coli (strain K12) OX=83333 GN=casC PE=1 SV=1

```
0     MSNFINIHVLISHSPSCLNRDDMNMQKDAIFGGKRRVRISSQSLKRAMRK
                                         ***************
50    SGYYAQNIGESSLRTIHLAQLRDVLRQKLGERFDQKIIDKTLALLSGKSV
      *********                ************************
100   DEAEKISADAVTPWVVGEIAWFCEQVAKAEADNLDDKKLLKVLKEDIAAI

150   RVNLQQGVDIALSGRMATSGMMTELGKVDGAMSIAHAITTHQVDSDIDWF

200   TAVDDLQEQGSAHLGTQEFSSGVFYRYANINLAQLQENLGGASREQALEI

250   ATHVVHMLATEVPGAKQRTYAAFNPADMVMVNFSDMPLSMANAFEKAVKA

300   KDGFLQPSIQAFNQYWDRVANGYGLNGAAAQFSLSDVDPITAQVKQMPTL

350   EQLKSWVRNNGEA
```

sp|P0A7V0|RS2_ECOLI 30S ribosomal protein S2 OS=Escherichia coli (strain K12) OX=83333 GN=rpsB PE=1 SV=2

```
0     MATVSMRDMLKAGVHFGHQTRYWNPKMKPFIFGARNKVHIINLEKTVPMF

50    NEALAELNKIASRKGKILFVGTKRAASEAVKDAALSCDQFFVNHRWLGGM
                   *********************
100   LTNWKTVRQSIKRLKDLETQSQDGTFDKLTKKEALMRTRELEKLENSLGG

150   IKDMGGLPDALFVIDADHEHIAIKEANNLGIPVFAIVDTNSDPDGVDFVI

200   PGNDDAIRAVTLYLGAVAATVREGRSQDLASQAEESFVEAE
```

sp|P16682|PHND_ECOLI Phosphonates-binding periplasmic protein OS=Escherichia coli (strain K12) OX=83333 GN=phnD PE=3 SV=2

```
0     MNAKIIASLAFTSMFSLSTLLSPAHAEEQEKALNFGIISTESQQNLKPQW

50    TPFLQDMEKKLGVKVNAFFAPDYAGIIQGMRFNKVDIAWYGNLSAMEAVD
```

```
100   RANGQVFAQTVAADGSPGYWSVLIVNKDSPINNLNDLLAKRKDLTFGNGD

150   PNSTSGFLVPGYYVFAKNNISASDFKRTVNAGHETNALAVANKQVDVATN
      **************************
200   NTENLDKLKTSAPEKLKELKVIWKSPLIPGDPIVWRKNLSETTKDKIYDF

250   FMNYGKTPEEKAVLERLGWAPFRASSDLQLVPIRQLALFKEMQGVKSNKG

300   LNEQDKLAKTTEIQAQLDDLDRLNNALSAMSSVSKAVQ
```

sp|P0A7Z0|RPIA_ECOLI Ribose-5-phosphate isomerase A OS=Escherichia coli (strain K12) OX=83333 GN=rpiA PE=1 SV=1

```
                                               *********
0     MTQDELKKAVGWAALQYVQPGTIVGVGTGSTAAHFIDALGTMKGQIEGAV
      ********************
50    SSSDASTEKLKSLGIHVFDLNEVDSLGIYVDGADEINGHMQMIKGGGAAL

100   TREKIIASVAEKFICIADASKQVDILGKFPLPVEVIPMARSAVARQLVKL

150   GGRPEYRQGVVTDNGNVILDVHGMEILDPIAMENAINAIPGVVTVGLFAN

200   RGADVALIGTPDGVKTIVK
```

sp|P0AF26|NARJ_ECOLI Nitrate reductase molybdenum cofactor assembly chaperone NarJ OS=Escherichia coli (strain K12) OX=83333 GN=narJ PE=1 SV=1

```
0     MIELVIVSRLLEYPDAALWQHQQEMFEAIAASKNLPKEDAHALGIFLRDL

50    TTMDPLDAQAQYSELFDRGRATSLLLFEHVHGESRDRGQAMVDLLAQYEQ

100   HGLQLNSRELPDHLPLYLEYLAQLPQSEAVEGLKDIAPILALLSARLQQR
                     ************************
150   ESRYAVLFDLLLKLANTAIDSDKVAEKIADEARDDTPQALDAVWEEEQVK

200   FFADKGCGDSAITAHQRRFAGAVAPQYLNITTGGQH
```

sp|P0AEJ4|ENVZ_ECOLI Osmolarity sensor protein EnvZ OS=Escherichia coli (strain K12) OX=83333 GN=envZ PE=1 SV=1

```
0     MRRLRFSPRSSFARTLLLIVTLLFASLVTTYLVVLNFAILPSLQQFNKVL

50    AYEVRMLMTDKLQLEDGTQLVVPPAFRREIYRELGISLYSNEAAEEAGLR

100   WAQHYEFLSHQMAQQLGGPTEVRVEVNKSSPVVWLKTWLSPNIWVRVPLT

150   EIHQGDFSPLFRYTLAIMLLAIGGAWLFIRIQNRPLVDLEHAALQVGKGI

200   IPPPLREYGASEVRSVTRAFNHMAAGVKQLADDRTLLMAGVSHDLRTPLT
            ********************
250   RIRLATEMMSEQDGYLAESINKDIEECNAIIEQFIDYLRTGQEMPMEMAD

300   LNAVLGEVIAAESGYEREIETALYPGSIEVKMHPLSIKRAVANMVVNAAR
```

350   YGNGWIKVSSGTEPNRAWFQVEDDGPGIAPEQRKHLFQPFVRGDSARTIS

400   GTGLGLAIVQRIVDNHNGMLELGTSERGGLSIRAWLPVPVTRAQGTTKEG

450

sp|Q2EET2|YPFN_ECOLI UPF0370 protein YpfN OS=Escherichia coli (strain K12) OX=83333 GN=ypfN PE=3 SV=1
                                           ****
0   MDWLAKYWWILVIVFLVGVLLNVIKDLKRVDHKKFLANKPELPPHRDFND
      ****
50   KWDDDDDWPKKDQPKK

sp|P0A7H0|RECF_ECOLI DNA replication and repair protein RecF OS=Escherichia coli (strain K12) OX=83333 GN=recF PE=1 SV=2

0   MSLTRLLIRDFRNIETADLALSPGFNFLVGANGSGKTSVLEAIYTLGHGR

50   AFRSLQIGRVIRHEQEAFVLHGRLQGEERETAIGLTKDKQGDSKVRIDGT

100   DGHKVAELAHLMPMQLITPEGFTLLNGGPKYRRAFLDWGCFHNEPGFFTA

150   WSNLKRLLKQRNAALRQVTRYEQLRPWDKELIPLAEQISTWRAEYSAGIA

200   ADMADTCKQFLPEFSLTFSFQRGWEKETEYAEVLERNFERDRQLTYTAHG

250   PHKADLRIRADGAPVEDTLSRGQLKLLMCALRLAQGEFLTRESGRRCLYL
                                ****
300   IDDFASELDDERRGLLASRLKATQSQVFVSAISAEHVIDMSDENSKMFTV
      ****
350   EKGKITD

sp|P25534|UBIH_ECOLI 2-octaprenyl-6-methoxyphenol hydroxylase OS=Escherichia coli (strain K12) OX=83333 GN=ubiH PE=1 SV=2

0   MSVIIVGGGMAGATLALAISRLSHGALPVHLIEATAPESHAHPGFDGRAI

50   ALAAGTCQQLARIGVWQSLADCATAITTVHVSDRGHAGFVTLAAEDYQLA

100   ALGQVVELHNVGQRLFALLRKAPGVTLHCPDRVANVARTQSHVEVTLESG

150   ETLTGRVLVAADGTHSALATACGVDWQQEPYEQLAVIANVATSVAHEGRA
                             ****
200   FERFTQHGPLAMLPMSDGRCSLVWCHPLERREEVLSWSDEKFCRELQSAF

250   GWRLGKITHAGKRSAYPLALTHAARSITHRTVLVGNAAQTLHPIAGQGFN

300   LGMRDVMSLAETLTQAQERGEDMGDYGVLCRYQQRRQSDREATIGVTDSL

350   VHLFANRWAPLVVGRNIGLMTMELFTPARDVLAQRTLGWVAR

sp|P05847|TTDA_ECOLI L(+)-tartrate dehydratase subunit alpha OS=Escherichia coli (strain K12) OX=83333 GN=ttdA PE=1 SV=3
                             ****

0   MMSESNKQQAVNKLTEIVANFTAMISTRMPDDVVDKLKQLKDAETSSMGK
      *
50   IIYHTMFDNMQKAIDLNRPACQDTGEIMFFVKVGSRFPLLGELQSILKQA

100   VEEATVKAPLRHNAVEIFDEVNTGKNTGSGVPWVTWDIIPDNDDAEIEVY

150   MAGGGCTLPGRSKVLMPSEGYEGVVKFVFENISTLAVNACPPVLVGVGIA

200   TSVETAAVLSRKAILRPIGSRHPNPKAAELELRLEEGLNRLGIGPQGLTG

250   NSSVMGVHIESAARHPSTIGVAVSTGCWAHRRGTLLVHADLTFENLSHTR

300   SAL

sp|P77365|YAFY_ECOLI Lipoprotein YafY OS=Escherichia coli (strain K12) OX=83333 GN=yafY PE=1 SV=2
                      ****
0   MKRKTLPLLALVATTLFLIACDDRSDDLKAISKFKDLTPPRFSDVVSHQD

50   DVSEEWSQVDYLSGPTLQVLRTRQSPDGCEDGSYYYLVDMQEKTVQPLMN

100   ALCIADNIKLEYQEVTDPYTKEKYFEYAHDGKLMGQLLIPSNPDNQE

sp|P75717|EXOD_ECOLI Putative uncharacterized protein ExoD OS=Escherichia coli (strain K12) OX=83333 GN=exoD PE=5 SV=1

0   MKFRLGGFEAIKSAYMAQVQYSMWVTRKDAWYFANYDPRMKREGLHYVVI
         ****
50   ERNEKYMASFDEMVPEFIEKMDEALAEIGFVFGEQWR

sp|Q46890|OTNC_ECOLI 3-oxo-tetronate 4-phosphate decarboxylase OS=Escherichia coli (strain K12) OX=83333 GN=otnC PE=3 SV=1

0   MSDFAKVEQSLREEMTRIASSFFQRGYATGSAGNLSLLLPDGNLLATPTG
               ****
50   SCLGNLDPQRLSKVAADGEWLSGDKPSKEVLFHLALYRNNPRCKAVVHLH

100   STWSTALSCLQGLDSSNVIRPFTPYVVMRMGNVPLVPYYRPGDKRIAQDL

150   AELAADNQAFLLANHGPVVCGESLQEAANNMEELEETAKLIFILGDRPIR

200   YLTAGEIAELRS

sp|P69874|POTA_ECOLI Spermidine/putrescine import ATP-binding protein PotA OS=Escherichia coli (strain K12) OX=83333 GN=potA PE=1 SV=1

0   MGQSKKLNKQPSSLSPLVQLAGIRKCFDGKEVIPQLDLTINNGEFLTLLG

50   PSGCGKTTVLRLIAGLETVDSGRIMLDNEDITHVPAENRYVNTVFQSYAL

100   FPHMTVFENVAFGLRMQKTPAAEITPRVMEALRMVQLETFAQRKPHQLSG

150   GQQQRVAIARAVVNKPRLLLLDESLSALDYKLRKQMQNELKALQRKLGIT

```
200   FVFVTHDQEEALTMSDRIVVMRDGRIEQDGTPREIYEEPKNLFVAGFIGE

250   INMFNATVIERLDEQRVRANVEGRECNIYVNFAVEPGQKLHVLLRPEDLR
                                             ***********
300   VEEINDDNHAEGLIGYVRERNYKGMTLESVVELENGKMVMVSEFFNEDDP
      ***********
350   DFDHSLDQKMAINWVESWEVVLADEEHK

sp|P37338|GLAR_ECOLI HTH-type transcriptional repressor GlaR
OS=Escherichia coli (strain K12) OX=83333 GN=glaR PE=1 SV=2

0     MTITSLDGYRWLKNDIIRGNFQPDEKLRMSLLTSRYALGVGPLREALSQL

50    VAERLVTVVNQKGYRVASMSEQELLDIFDARANMEAMLVSLAIARGGDEW
            *************************
100   EADVLAKAHLLSKLEACDASEKMLDEWDLRHQAFHTAIVAGCGSHYLLQM

150   RERLFDLAARYRFIWLRRTVLSVEMLEDKHDQHQTLTAAVLARDTARASE

200   LMRQHLLTPIPIIQQAMAGN

sp|P64519|YODD_ECOLI Uncharacterized protein YodD OS=Escherichia coli
(strain K12) OX=83333 GN=yodD PE=4 SV=1
      *********************
0     MKTAKEYSDTAKREVSVDVDALLAAINEISESEVHRSQNDSEHVSVDGRE

50    YHTWRELADAFELDIHDFSVSEVNR

sp|P0AD35|YFDO_ECOLI Protein YfdO OS=Escherichia coli (strain K12)
OX=83333 GN=yfdO PE=3 SV=1

0     MTCAQWLWKKIIALYEQAAECDGEVVRPKEPNWTAWANEIRLMCVQDGRT
                           *****************************
50    HKQICEMYSRVSRDPFWCRNVLSPSKLREKWDELSLRLSPSVSTYTEKRE
      *
100   DPYFKASYDNVDYSQIPAGFRG

sp|P0AFH6|OPPC_ECOLI Oligopeptide transport system permease protein OppC
OS=Escherichia coli (strain K12) OX=83333 GN=oppC PE=1 SV=1
      *************************
0     MMLSKKNSETLENFSEKLEVEGRSLWQDARRRFMHNRAAVASLIVLVLIA

50    LFVILAPMLSQFAYDDTDWAMMSSAPDMESGHYFGTDSSGRDLLVRVAIG

100   GRISLMVGVAAALVAVVVGTLYGSLSGYLGGKVDSVMMRLLEILNSFPFM

150   FFVILLVTFFGQNILLIFVAIGMVSWLDMARIVRGQTLSLKRKEFIEAAQ

200   VGGVSTSGIVIRHIVPNVLGVVVVYASLLVPSMILFESFLSFLGLGTQEP

250   LSSWGALLSDGANSMEVSPWLLLFPAGFLVVTLFCFNFIGDGLRDALDPK

300   DR
```

```
sp|P33354|YEHR_ECOLI Uncharacterized lipoprotein YehR OS=Escherichia coli
(strain K12) OX=83333 GN=yehR PE=1 SV=2
                   ********************
0     MKAFNKLFSLVVASVLVFSLAGCGDKEESKKFSANLNGTEIAITYVYKGD

50    KVLKQSSETKIQFASIGATTKEDAAKTLEPLSAKYKNIAGVEEKLTYTDT

100   YAQENVTIDMEKVDFKALQGISGINVSAEDAKKGITMAQMELVMKAAGFK

150   EVK

sp|P0ACD8|MBHL_ECOLI Hydrogenase-1 large chain OS=Escherichia coli
(strain K12) OX=83333 GN=hyaB PE=1 SV=1

0     MSTQYETQGYTINNAGRRLVVDPITRIEGHMRCEVNINDQNVITNAVSCG

50    TMFRGLEIILQGRDPRDAWAFVERICGVCTGVHALASVYAIEDAIGIKVP
                                            ************
100   DNANIIRNIMLATLWCHDHLVHFYQLAGMDWIDVLDALKADPRKTSELAQ
      **********
150   SLSSWPKSSPGYFFDVQNRLKKFVEGGQLGIFRNGYWGHPQYKLPPEANL

200   MGFAHYLEALDFQREIVKIHAVFGGKNPHPNWIVGGMPCAINIDESGAVG

250   AVNMERLNLVQSIITRTADFINNVMIPDALAIGQFNKPWSEIGTGLSDKC

300   VLSYGAFPDIANDFGEKSLLMPGGAVINGDFNNVLPVDLVDPQQVQEFVD

350   HAWYRYPNDQVGRHPFDGITDPWYNPGDVKGSDTNIQQLNEQERYSWIKA

400   PRWRGNAMEVGPLARTLIAYHKGDAATVESVDRMMSALNLPLSGIQSTLG

450   RILCRAHEAQWAAGKLQYFFDKLMTNLKNGNLATASTEKWEPATWPTECR

500   GVGFTEAPRGALGHWAAIRDGKIDLYQCVVPTTWNASPRDPKGQIGAYEA

550   ALMNTKMAIPEQPLEILRTLHSFDPCLACSTHVLGDDGSELISVQVR

sp|P43676|PITB_ECOLI Probable low-affinity inorganic phosphate
transporter 2 OS=Escherichia coli (strain K12) OX=83333 GN=pitB PE=3 SV=1

0     MLNLFVGLDIYTGLLLLLALAFVLFYEAINGFHDTANAVAAVIYTRAMQP

50    QLAVVMAAFFNFFGVLLGGLSVAYAIVHMLPTDLLLNMGSTHGLAMVFSM

100   LLAAIIWNLGTWFFGLPASSSHTLIGAIIGIGLTNALLTGSSVMDALNLR
                                     *******************
150   EVTKIFSSLIVSPIVGLVIAGGLIFLLRRYWSGTKKRDRIHRIPEDRKKK
      *****
200   KGKRKPPFWTRIALIVSAAGVAFSHGANDGQKGIGLVMLVLVGIAPAGFV

250   VNMNASGYEITRTRDAVTNFEHYLQQHPELPQKLIAMEPPLPAASTDGTQ

300   VTEFHCHPANTFDAIARVKTMLPGNMESYEPLSVSQRSQLRRIMLCISDT
```

```
                ********************
350   SAKLAKLPGVSKEDQNLLKKLRSDMLSTIEYAPVWIIMAVALALGIGTMI

400   GWRRVAMTIGEKIGKRGMTYAQGMAAQMTAAVSIGLASYIGMPVSTTHVL

450   SSAVAGTMVVDGGGLQRKTVTSILMAWVFTLPAAIFLSGGLYWIALQLI
```

sp|P32128|YIHF_ECOLI Uncharacterized protein YihF OS=Escherichia coli (strain K12) OX=83333 GN=yihF PE=3 SV=3

```
                                     *******************
0     MIRKSATGVIVALAVIWGGGTWYTGTQIQPGVEKFIKDFNDAKKKGEHAY
      **
50    DMTLSYQNFDKGFFNSRFQMQMTFDNGAPDLNIKPGQKVVFDVDVEHGPL

100   PITMLMHGNVIPALAAAKVNLVNNELTQPLFIAAKNKSPVEATLRFAFGG

150   SFSTTLDVAPAEYGKFSFGEGQFTFNGDGSSLSNLDIEGKVEDIVLQLSP
               ***********************
200   MNKVTAKSFTIDSLARLEEKKFPVGESESKFNQINIINHGEDVAQIDAFV

250   AKTRLDRVKDKDYINVNLTYELDKLTKGNQQLGSGEWSLIAESIDPSAVR

300   QFIIQYNIAMQKQLAAHPELANDEVALQEVNAALFKEYLPLLQKSEPTIK
                   ********************************
350   QPVRWKNALGELNANLDISIADPAKSSSSTNKDIKSLNFDVKLPLNVVTE

400   TAKQLNLSEGMDAEKAQKQADKQISGMMTLGQMFQLITIDNNTASLQLRY

450   TPGKVVFNGQEMSEEEFMSRAGRFVH
```

sp|P0AGM5|SIRB1_ECOLI UPF0162 protein YchA OS=Escherichia coli (strain K12) OX=83333 GN=ychA PE=3 SV=1

```
                                    ********************
0     MRSLADFEFNKAPLCEGMILACEAIRRDFPSQDVYDELERLVSLAKEEIS

50    QLLPLEEQLEKLIALFYGDWGFKASRGVYRLSDALWLDQVLKNRQGSAVS

100   LGAVLLWVANRLDLPLLPVIFPTQLILRIECPDGEIWLINPFNGESLSEH

150   MLDVWLKGNISPSAELFYEDLDEADNIEVIRKLLDTLKASLMEENQMELA

200   LRTSEALLQFNPEDPYEIRDRGLIYAQLDCEHVALNDLSYFVEQCPEDPI

250   SEMIRAQINNIAHKHIVLH
```

sp|P28249|ASMA_ECOLI Protein AsmA OS=Escherichia coli (strain K12) OX=83333 GN=asmA PE=3 SV=2

```
0     MRRFLTTLMILLVVLVAGLSALVLLVNPNDFRDYMVKQVAARSGYQLQLD

50    GPLRWHVWPQLSILSGRMSLTAQGASQPLVRADNMRLDVALLPLLSHQLS
                                        ****************
100   VKQVMLKGAVIQLTPQTEAVRSEDAPVAPRDNTLPDLSDDRGWSFDISSL
      ***************
```

150   KVADSVLVFQHEDDEQVTIRNIRLQMEQDPQHRGSFEFSGRVNRDQRDLT

200   ISLNGTVDASDYPHDLTAAIEQINWQLQGADLPKQGIQGQGSFQAQWQES

250   HKRLSFNQISLTANDSTLSGQAQVTLTEKPEWQLRLQFPQLNLDNLIPLN

300   ETANGENGAAQQGQSQSTLPRPVISSRIDEPAYQGLQGFTADILLQASNV

350   RWRGMNFTDVATQMTNKSGLLEITQLQGKLNGGQVSLPGTLDATSINPRI

400   NFQPRLENVEIGTILKAFNYPISLTGKMSLAGDFSGADIDADAFRHNWQG

450   QAHVEMTDTRMEGMNFQQMIQQAVERNGGDVKAAENFDNVTRLDRFTTDL

500   TLKDGVVTLNDMQGQSPVLALTGEGMLNLADQTCDTQFDIRVVGGWNGES
*****
550   KLIDFLKETPVPLRVYGNWQQLNYSLQVDQLLRKHLQDEAKRRLNDWAER
****
600   NKDSRNGKDVKKLLEKM

sp|P30844|BASS_ECOLI Sensor protein BasS OS=Escherichia coli (strain K12) OX=83333 GN=basS PE=1 SV=1

0   MHFLRRPISLRQRLILTIGAILLVFELISVFWLWHESTEQIQLFEQALRD

50   NRNNDRHIMREIREAVASLIVPGVFMVSLTLFICYQAVRRITRPLAELQK

100   ELEARTADNLTPIAIHSATLEIEAVVSALNDLVSRLTSTLDNERLFTADV

150   AHELRTPLAGVRLHLELLAKTHHIDVAPLVARLDQMMESVSQLLQLARAG

200   QSFSSGNYQHVKLLEDVILPSYDELSTMLDQRQQTLLLPESAADITVQGD
****
250   ATLLRMLLRNLVENAHRYSPQGSNIMIKLQEDDGAVMAVEDEGPGIDESK
****
300   CGELSKAFVRMDSRYGGIGLGLSIVSRITQLHHGQFFLQNRQETSGTRAW

350   VRLKKDQYVANQI

sp|P39398|LGOT_ECOLI Probable L-galactonate transporter OS=Escherichia coli (strain K12) OX=83333 GN=lgoT PE=1 SV=2
****
0   MEKENITIDPRSSFTPSSSADIPVPPDGLVQRSTRIKRIQTTAMLLLFFA

50   AVINYLDRSSLSVANLTIREELGLSATEIGALLSVFSLAYGIAQLPCGPL

100   LDRKGPRLMLGLGMFFWSLFQAMSGMVHNFTQFVLVRIGMGIGEAPMNPC

150   GVKVINDWFNIKERGRPMGFFNAASTIGVAVSPPILAAMMLVMGWRGMFI

200   TIGVLGIFLAIGWYMLYRNREHVELTAVEQAYLNAGSVNARRDPLSFAEW

250   RSLFRNRTMWGMMLGFSGINYTAWLYLAWLPGYLQTAYNLDLKSTGLMAA

```
300   IPFLFGAAGMLVNGYVTDWLVKGGMAPIKSRKICIIAGMFCSAAFTLIVP

350   QATTSMTAVLLIGMALFCIHFAGTSCWGLIHVAVASRMTASVGSIQNFAS

400   FICASFAPIITGFIVDTTHSFRLALIICGCVTAAGALAYIFLVRQPINDP

450   RKD

sp|P39295|YJFM_ECOLI Uncharacterized protein YjfM OS=Escherichia coli
(strain K12) OX=83333 GN=yjfM PE=4 SV=1

0     MARKRKSRNNSKIGHGAISRIGRPNNPFEPCRNRYAQKYLTLALMGGAAF

50    FVLKGCSDSSDVDNDGDGTFYATVQDCIDDGNNADICARGWNNAKTAFYA

100   DVPKNMTQQNCQSKYENCYYDNVEQSWIPVVSGFLLSRVIRKDRDEPFVY
                 ************************************
150   NSGGSSFASRPVWRSTSGDYSWRSGSGKKESYSSGGFTTKKASTVSRGGY

200   GRSSSARGHWGG

sp|P0AB71|ALF_ECOLI Fructose-bisphosphate aldolase class 2 OS=Escherichia
coli (strain K12) OX=83333 GN=fbaA PE=1 SV=2

0     MSKIFDFVKPGVITGDDVQKVFQVAKENNFALPAVNCVGTDSINAVLETA

50    AKVKAPVIVQFSNGGASFIAGKGVKSDVPQGAAILGAISGAHHVHQMAEH
                                              **********
100   YGVPVILHTDHCAKKLLPWIDGLLDAGEKHFAATGKPLFSSHMIDLSEES
      *****************
150   LQENIEICSKYLERMSKIGMTLEIELGCTGGEEDGVDNSHMDASALYTQP

200   EDVDYAYTELSKISPRFTIAASFGNVHGVYKPGNVVLTPTILRDSQEYVS

250   KKHNLPHNSLNFVFHGGSGSTAQEIKDSVSYGVVKMNIDTDTQWATWEGV

300   LNYYKANEAYLQGQLGNPKGEDQPNKKYYDPRVWLRAGQTSMIARLEKAF

350   QELNAIDVL

sp|P64554|QUEE_ECOLI 7-carboxy-7-deazaguanine synthase OS=Escherichia
coli (strain K12) OX=83333 GN=queE PE=3 SV=1
                                                 *******
0     MQYPINEMFQTLQGEGYFTGVPAIFIRLQGCPVGCAWCDTKHTWEKLEDR
      *****************************
50    EVSLFSILAKTKESDKWGAASSEDLLAVIGRQGYTARHVVITGGEPCIHD

100   LLPLTDLLEKNGFSCQIETSGTHEVRCTPNTWVTVSPKLNMRGGYEVLSQ

150   ALERANEIKHPVGRVRDIEALDELLATLTDDKPRVIALQPISQKDDATRL

200   CIETCIARNWRLSMQTHKYLNIA
```

```
sp|P75882|GFCD_ECOLI Uncharacterized lipoprotein GfcD OS=Escherichia coli
(strain K12) OX=83333 GN=gfcD PE=3 SV=1

0     MKKNSYLLSCLAIAVSSACHAEVLTYPDPLGSSQSDFGGTGLLQMPNARI
                                            ************
50    APEGEFSVNYRDNDQYRFYSTSVALFPWLEGTIRYTDVRTRKYSQWEDFS
      *********************
100   GDQSYKDKSFDFKLRLWEEGYWLPQVAFGKRDIAGTGLFDGEYLVASKQA
                                 ********************
150   GPFDFTLGMAWGYAGNAGNITNPFCRVSDKYCHRAESHDAGDISFSDIFR

200   GPASIFGGIEYQTPWNPLRLKLEYDGNNYQNDFAGKLPQASHFNVGAVYR

250   AASWADLNLSYERGNTLMFGFTLRTNFNDLRPALRDTPKPAYQPAPESEG

300   LQYTTVANQLTALKYNAGFDAPEIQLRDKTLYMSGQQYKYRDSREAVDRA

350   NRILVNNLPQGVEKISVTQKREHMAMVTTETDVASLRKQLAGTAPGQSEP

400   LQQQRVEAEDLSAFGRGYRIREDRFSYSFNPTLSQSLGGPEDFYMFQLGL

450   MSSARYWFTDHLLLDGGIFTNIYNNYDKFKSSLLPADSTLPRVRTHIRDY

500   VRNDVYLNNLQANYFADLGNGFYGQVYGGYLETMYAGVGSELLYRPLDAC

550   WALGVDVNYVKQRDWDNMMRFTDYSTPTGFVTAYWNPPTLNGVLMKLSVG

600   QYLAKDKGATIDVAKRFDSGVAVGVWAAISNVSKDDYGEGGFSKGFYISI

650   PFDLMTIGPNRNRAVVSWTPLTRDGGQMLSRKYQLYPMTAEREVPVGQ

sp|P76162|YDFU_ECOLI Uncharacterized protein YdfU OS=Escherichia coli
(strain K12) OX=83333 GN=ydfU PE=4 SV=4

0     MRVLLRPVLVPELGLVVLKPGRESIQIFHNPRVLVEPEPKSMRNLPSGVV

50    PAVRQPLAEDKTLLPFFSNERVIRAAGGVGALSDWLLRHVTSCQWPNGDY

100   HHTETVIHRYGTGAMVLCWHCDNQLRDQTSESLELLAQQNLTAWVIDVIR

150   HAISGTQERELSLAELSWWAVCNQVVDALPEAVSRRSLGLPAEKICSVYR
                                      ******************
200   ESDIVPGELTATSILKQRTKNLAPLPYAHQQQKSPQEKTVVSITVDPESP
      **
250   ESFMKLPKRRRWVKEKYTRWVKTQPCACCGMPADDPHHLIGHGQGGMGTK

300   AHDLFVLPLCRKHHNELHTDTVAFEDKYGSQLELIFRFIDRALAIGVLA

sp|P45508|YFAL_ECOLI Probable autotransporter YfaL OS=Escherichia coli
(strain K12) OX=83333 GN=yfaL PE=1 SV=2

0     MRIIFLRKEYLSLLPSMIASLFSANGVAAVTDSCQGYDVKASCQASRQSL

50    SGITQDWSIADGQWLVFSDMTNNASGGAVFLQQGAEFSLLPENETGMTLF
```

100 ANNTVTGEYNNGGAIFAKENSTLNLTDVIFSGNVAGGYGGAIYSSGTNDT
**

150 GAVDLRVTNAMFRNNIANDGKGGAIYTINNDVYLSDVIFDNNQAYTSTSY
****

200 SDGDGGAIDVTDNNSDSKHPSGYTIVNNTAFTNNTAEGYGGAIYTNSVTA

250 PYLIDISVDDSYSQNGGVLVDENNSAAGYGDGPSSAAGGFMYLGLSEVTF

300 DIADGKTLVIGNTENDGAVDSIAGTGLITKTGSGDLVLNADNNDFTGEMQ

350 IENGEVTLGRSNSLMNVGDTHCQDDPQDCYGLTIGSIDQYQNQAELNVGS

400 TQQTFVHALTGFQNGTLNIDAGGNVTVNQGSFAGIIEGAGQLTIAQNGSY

450 VLAGAQSMALTGDIVVDDGAVLSLEGDAADLTALQDDPQSIVLNGGVLDL

500 SDFSTWQSGTSYNDGLEVSGSSGTVIGSQDVVDLAGGDNLHIGGDGKDGV
*

550 YVVVDASDGQVSLANNNSYLGTTQIASGTLMVSDNSQLGDTHYNRQVIFT
****

600 DKQQESVMEITSDVDTRSDAAGHGRDIEMRADGEVAVDAGVDTQWGALMA

650 DSSGQHQDEGSTLTKTGAGTLELTASGTTQSAVRVEEGTLKGDVADILPY

700 ASSLWVGDGATFVTGADQDIQSIDAISSGTIDISDGTVLRLTGQDTSVAL

750 NASLFNGDGTLVNATDGVTLTGELNTNLETDSLTYLSNVTVNGNLTNTSG

800 AVSLQNGVAGDTLTVNGDYTGGGTLLLDSELNGDDSVSDQLVMNGNTAGN

850 TTVVVNSITGIGEPTSTGIKVVDFAADPTQFQNNAQFSLAGSGYVNMGAY

900 DYTLVEDNNDWYLRSQEVTPPSPPDPDPTPDPDPTPDPDPTPDPEPTPAY

950 QPVLNAKVGGYLNNLRAANQAFMMERRDHAGGDGQTLNLRVIGGDYHYTA

1000 AGQLAQHEDTSTVQLSGDLFSGRWGTDGEWMLGIVGGYSDNQGDSRSNMT

1050 GTRADNQNHGYAVGLTSSWFQHGNQKQGAWLDSWLQYAWFSNDVSEQEDG

1100 TDHYHSSGIIASLEAGYQWLPGRGVVIEPQAQVIYQGVQQDDFTAANRAR
****

1150 VSQSQGDDIQTRLGLHSEWRTAVHVIPTLDLNYYHDPHSTEIEEDGSTIS
****

1200 DDAVKQRGEIKVGVTGNISQRVSLRGSVAWQKGSDDFAQTAGFLSMTVKW

1250

sp|P75685|RCLC_ECOLI Inner membrane protein RclC OS=Escherichia coli (strain K12) OX=83333 GN=rclC PE=1 SV=2

0 MEKYLHLLSRGDKIGLTLIRLSIAIVFMWIGLLKFVPYEADSITPFVANS

50 PLMSFFYEHPEDYKQYLTHEGEYKPEARAWQTANNTYGFSNGLGVVEVII

100 ALLVLANPVNRWLGLLGGLMAFTTPLVTLSFLITTPEAWVPALGDAHHGF
****

150 PYLSGAGRLVLKDTLMLAGAVMIMADSAREILKQRSNESSSTLKTEY

sp|P00864|CAPP_ECOLI Phosphoenolpyruvate carboxylase OS=Escherichia coli (strain K12) OX=83333 GN=ppc PE=1 SV=1

0 MNEQYSALRSNVSMLGKVLGETIKDALGEHILERVETIRKLSKSSRAGND

50 ANRQELLTTLQNLSNDELLPVARAFSQFLNLANTAEQYHSISPKGEAASN
****

100 PEVIARTLRKLKNQPELSEDTIKKAVESLSLELVLTAHPTEITRRTLIHK

150 MVEVNACLKQLDNKDIADYEHNQLMRRLRQLIAQSWHTDEIRKLRPSPVD

200 EAKWGFAVVENSLWQGVPNYLRELNEQLEENLGYKLPVEFVPVRFTSWMG

250 GDRDGNPNVTADITRHVLLLSRWKATDLFLKDIQVLVSELSMVEATPELL

300 ALVGEEGAAEPYRYLMKNLRSRLMATQAWLEARLKGEELPKPEGLLTQNE

350 ELWEPLYACYQSLQACGMGIIANGDLLDTLRRVKCFGVPLVRIDIRQEST

400 RHTEALGELTRYLGIGDYESWSEADKQAFLIRELNSKRPLLPRNWQPSAE

450 TREVLDTCQVIAEAPQGSIAAYVISMAKTPSDVLAVHLLLKEAGIGFAMP

500 VAPLFETLDDLNNANDVMTQLLNIDWYRGLIQGKQMVMIGYSDSAKDAGV

550 MAASWAQYQAQDALIKTCEKAGIELTLFHGRGGSIGRGGAPAHAALLSQP

600 PGSLKGGLRVTEQGEMIRFKYGLPEITVSSLSLYTGAILEANLLPPPEPK

650 ESWRRIMDELSVISCDVYRGYVRENKDFVPYFRSATPEQELGKLPLGSRP

700 AKRRPTGGVESLRAIPWIFAWTQNRLMLPAWLGAGTALQKVVEDGKQSEL

750 EAMCRDWPFFSTRLGMLEMVFAKADLWLAEYYDQRLVDKALWPLGKELRN

800 LQEEDIKVVLAIANDSHLMADLPWIAESIQLRNIYTDPLNVLQAELLHRS

850 RQAEKEGQEPDPRVEQALMVTIAGIAAGMRNTG

sp|P07012|RF2_ECOLI Peptide chain release factor RF2 OS=Escherichia coli (strain K12) OX=83333 GN=prfB PE=1 SV=3
****

0 MFEINPVNNRIQDLTERSDVLRGYLDYDAKKERLEEVNAELEQPDVWNEP
****

50 ERAQALGKERSSLEAVVDTLDQMKQGLEDVSGLLELAVEADDEETFNEAV
****

100 AELDALEEKLAQLEFRRMFSGEYDSADCYLDIQAGSGGTEAQDWASMLER
****

```
150   MYLRWAESRGFKTEIIEESEGEVAGIKSVTIKISGDYAYGWLRTETGVHR

200   LVRKSPFDSGGRRHTSFSSAFVYPEVDDDIDIEINPADLRIDVYRTSGAG
                                                   *****
250   GQHVNRTESAVRITHIPTGIVTQCQNDRSQHKNKDQAMKQMKAKLYELEM
      ********************
300   QKKNAEKQAMEDNKSDIGWGSQIRSYVLDDSRIKDLRTGVETRNTQAVLD

350   GSLDQFIEASLKAGL
```

sp|P69423|TATC_ECOLI Sec-independent protein translocase protein TatC OS=Escherichia coli (strain K12) OX=83333 GN=tatC PE=1 SV=2

```
0     MSVEDTQPLITHLIELRKRLLNCIIAVIVIFLCLVYFANDIYHLVSAPLI

50    KQLPQGSTMIATDVASPFFTPIKLTFMVSLILSAPVILYQVWAFIAPALY

100   KHERRLVVPLLVSSSLLFYIGMAFAYFVVFPLAFGFLANTAPEGVQVSTD

150   IASYLSFVMALFMAFGVSFEVPVAIVLLCWMGITSPEDLRKKRPYVLVGA
                                         ***************
200   FVVGMLLTPPDVFSQTLLAIPMYCLFEIGVFFSRFYVGKGRNREEENDAE
      ********
250   AESEKTEE
```

sp|P0AB96|ARSC_ECOLI Arsenate reductase OS=Escherichia coli (strain K12) OX=83333 GN=arsC PE=3 SV=1

```
0     MSNITIYHNPACGTSRNTLEMIRNSGTEPTIIHYLETPPTRDELVKLIAD

50    MGISVRALLRKNVEPYEELGLAEDKFTDDRLIDFMLQHPILINRPIVVTP
                     **************************
100   LGTRLCRPSEVVLEILPDAQKGAFSKEDGEKVVDEAGKRLK
```

sp|P0DPC5|YTID_ECOLI Protein YtiD OS=Escherichia coli (strain K12) OX=83333 GN=ytiD PE=1 SV=1

```
                  ********************
0     MADYAEINNFPPELSSSGDKYFHLRNYSEYSEYTSGFFLSLMIFIKS
```

sp|P17334|PTQC_ECOLI PTS system N,N'-diacetylchitobiose-specific EIIC component OS=Escherichia coli (strain K12) OX=83333 GN=chbC PE=1 SV=2

```
0     MSNVIASLEKVLLPFAVKIGKQPHVNAIKNGFIRLMPLTLAGAMFVLINN

50    VFLSFGEGSFFYSLGIRLDASTIETLNGLKGIGGNVYNGTLGIMSLMAPF

100   FIGMALAEERKVDALAAGLLSVAAFMTVTPYSVGEAYAVGANWLGGANII

150   SGIIIGLVVAEMFTFIVRRNWVIKLPDSVPASVSRSFSALIPGFIILSVM

200   GIIAWALNTWGTNFHQIIMDTISTPLASLGSVVGWAYVIFVPLLWFFGIH

250   GALALTALDNGIMTPWALENIATYQQYGSVEAALAAGKTFHIWAKPMLDS
```

```
300   FIFLGGSGATLGLILAIFIASRRADYRQVAKLALPSGIFQINEPILFGLP

350   IIMNPVMFIPFVLVQPILAAITLAAYYMGIIPPVTNIAPWTMPTGLGAFF
                                       *******************
400   NTNGSVAALLVALFNLGIATLIYLPFVVVANKAQNAIDKEESEEDIANAL
      *
450   KF
```

sp|P0AEX9|MALE_ECOLI Maltose/maltodextrin-binding periplasmic protein OS=Escherichia coli (strain K12) OX=83333 GN=malE PE=1 SV=1

```
0     MKIKTGARILALSALTTMMFSASALAKIEEGKLVIWINGDKGYNGLAEVG
      ***********************
50    KKFEKDTGIKVTVEHPDKLEEKFPQVAATGDGPDIIFWAHDRFGGYAQSG

100   LLAEITPDKAFQDKLYPFTWDAVRYNGKLIAYPIAVEALSLIYNKDLLPN
       *********************
150   PPKTWEEIPALDKELKAKGKSALMFNLQEPYFTWPLIAADGGYAFKYENG

200   KYDIKDVGVDNAGAKAGLTFLVDLIKNKHMNADTDYSIAEAAFNKGETAM

250   TINGPWAWSNIDTSKVNYGVTVLPTFKGQPSKPFVGVLSAGINAASPNKE
                          ********************
300   LAKEFLENYLLTDEGLEAVNKDKPLGAVALKSYEEELAKDPRIAATMENA

350   QKGEIMPNIPQMSAFWYAVRTAVINAASGRQTVDEALKDAQTRITK
```

sp|P00904|TRPGD_ECOLI Bifunctional protein TrpGD OS=Escherichia coli (strain K12) OX=83333 GN=trpGD PE=1 SV=3

```
0     MADILLLDNIDSFTYNLADQLRSNGHNVVIYRNHIPAQTLIERLATMSNP

50    VLMLSPGPGVPSEAGCMPELLTRLRGKLPIIGICLGHQAIVEAYGGYVGQ

100   AGEILHGKASSIEHDGQAMFAGLTNPLPVARYHSLVGSNIPAGLTINAHF

150   NGMVMAVRHDADRVCGFQFHPESILTTQGARLLEQTLAWAQQKLEPANTL

200   QPILEKLYQAQTLSQQESHQLFSAVVRGELKPEQLAAALVSMKIRGEHPN

250   EIAGAATALLENAAPFPRPDYLFADIVGTGGDGSNSINISTASAFVAAAC
      **********************
300   GLKVAKHGNRSVSSKSGSSDLLAAFGINLDMNADKSRQALDELGVCFLFA

350   PKYHTGFRHAMPVRQQLKTRTLFNVLGPLINPAHPPLALIGVYSPELVLP

400   IAETLRVLGYQRAAVVHSGGMDEVSLHAPTIVAELHDGEIKSYQLTAEDF

450   GLTPYHQEQLAGGTPEENRDILTRLLQGKGDAAHEAAVAANVAMLMRLHG

500   HEDLQANAQTVLEVLRSGSAYDRVTALAARG
```

sp|P0A7D7|PUR7_ECOLI Phosphoribosylaminoimidazole-succinocarboxamide synthase OS=Escherichia coli (strain K12) OX=83333 GN=purC PE=1 SV=1

```
0     MQKQAELYRGKAKTVYSTENPDLLVLEFRNDTSAGDGARIEQFDRKGMVN

50    NKFNYFIMSKLAEAGIPTQMERLLSDTECLVKKLDMVPVECVVRNRAAGS

100   LVKRLGIEEGIELNPPLFDLFLKNDAMHDPMVNESYCETFGWVSKENLAR
                                              ************
150   MKELTYKANDVLKKLFDDAGLILVDFKLEFGLYKGEVVLGDEFSPDGSRL
      *****************
200   WDKETLEKMDKDRFRQSLGGLIEAYEAVARRLGVQLD

sp|P0A8I3|YAAA_ECOLI Peroxide stress resistance protein YaaA
OS=Escherichia coli (strain K12) OX=83333 GN=yaaA PE=1 SV=1

0     MLILISPAKTLDYQSPLTTTRYTLPELLDNSQQLIHEARKLTPPQISTLM

50    RISDKLAGINAARFHDWQPDFTPANARQAILAFKGDVYTGLQAETFSEDD

100   FDFAQQHLRMLSGLYGVLRPLDLMQPYRLEMGIRLENARGKDLYQFWGDI
                          *********************
150   ITNKLNEALAAQGDNVVINLASDEYFKSVKPKKLNAEIIKPVFLDEKNGK
                                       *****************
200   FKIISFYAKKARGLMSRFIIENRLTKPEQLTGFNSEGYFFDEDSSSNGEL
      ****
250   VFKRYEQR

sp|P61316|LOLA_ECOLI Outer-membrane lipoprotein carrier protein
OS=Escherichia coli (strain K12) OX=83333 GN=lolA PE=1 SV=1
                     *****************************
0     MKKIAITCALLSSLVASSVWADAASDLKSRLDKVSSFHASFTQKVTDGSG

50    AAVQEGQGDLWVKRPNLFNWHMTQPDESILVSDGKTLWFYNPFVEQATAT

100   WLKDATGNTPFMLIARNQSSDWQQYNIKQNGDDFVLTPKASNGNLKQFTI

150   NVGRDGTIHQFSAVEQDDQRSSYQLKSQQNGAVDAAKFTFTPPQGVTVDD

200   QRK

sp|P76322|YEDM_ECOLI Uncharacterized protein YedM OS=Escherichia coli
(strain K12) OX=83333 GN=yedM PE=4 SV=1

0     MFPLNDLSLKTQPVQLNKVTSNTESTIKQHELVSDDAIINELSSELVSCL
             *********************
50    GNGKFTPISEDSKLFNMLSEFKLLHSEYFEWGDYSLWFQDFSIYNKIGFI

100   MIEKIRELVTHPFGIN

sp|P0A8A0|YEBC_ECOLI Probable transcriptional regulatory protein YebC
OS=Escherichia coli (strain K12) OX=83333 GN=yebC PE=1 SV=1

0     MAGHSKWANTRHRKAAQDAKRGKIFTKIIRELVTAAKLGGGDPDANPRLR

50    AAVDKALSNNMTRDTLNRAIARGVGGDDDANMETIIYEGYGPGGTAIMIE
```

```
                                  **********************
100   CLSDNRNRTVAEVRHAFSKCGGNLGTDGSVAYLFSKKGVISFEKGDEDTI
      ****                                     *********
150   MEAALEAGAEDVVTYDDGAIDVYTAWEEMGKVRDALEAAGLKADSAEVSM
      ***********
200   IPSTKADMDAETAPKLMRLIDMLEDCDDVQEVYHNGEISDEVAATL
```

sp|P76578|A2MG_ECOLI Alpha-2-macroglobulin OS=Escherichia coli (strain K12) OX=83333 GN=yfhM PE=1 SV=1

```
                                ************************
0     MKKLRVAACMLMLALAGCDNNDNAPTAVKKDAPSEVTKAASSENASSAKL
      *******
50    SVPERQKLAQQSAGKVLTLLDLSEVQLDGAATLVLTFSIPLDPDQDFSRV
        ***********************
100   IHVVDKKSGKVDGAWELSDNLKELRLRHLEPKRDLIVTIGKEVKALNNAT

150   FSKDYEKTITTRDIQPSVGFASRGSLLPGKVVEGLPVMALNVNNVDVNFF

200   RVKPESLPAFISQWEYRNSLANWQSDKLLQMADLVYTGRFDLNPARNTRE

250   KLLLPLGDIKPLQQAGVYLAVMNQAGRYDYSNPATLFTLSDIGVSAHRYH

300   NRLDIFTQSLENGAAQQGIEVSLLNEKGQTLTQATSDAQGHVQLENDKNA

350   ALLLARKDGQTTLLDLKLPALDLAEFNIAGAPGYSKQFFMFGPRDLYRPG

400   ETVILNGLLRDADGKALPNQPIKLDVIKPDGQVLRSVVSQPENGLYHFTW

450   PLDSNAATGMWHIRANTGDNQYRMWDFHVEDFMPERMALNLTGEKTPLTP

500   KDEVKFSVVGYYLYGAPANGNTLQGQLFLRPLREAVSALPGFEFGDIAAE
                  *********************
550   NLSRTLDEVQLTLDDKGRGEVSTESQWKETHSPLQVIFQGSLLESGGRPV

600   TRRAEQAIWPADALPGIRPQFASKSVYDYRTDSTVKQPIVDEGSNAAFDI
                                   *********************
650   VYSDAQGVKKAVSGLQVRLIRERRDYYWNWSEDEGWQSQFDQKDLIENEQ

700   TLDLKADETGKVSFPVEWGAYRLEVKAPNEAVSSVRFWAGYSWQDNSDGS

750   GAVRPDRVTLKLDKASYRPGDTIKLHIAAPTAGKGYAMVESSEGPLWWQE

800   IDVRAQGLDLTIPVDKTWNRHDLYLSTLVVRPGDKSRSATPKRAVGVLHL

850   PLGDENRRLDLALETPAKMRPNQPLTVKIKASTKNGEKPKQVNVLVSAVD

900   SGVLNITDYVTPDPWQAFFGQKRYGADIYDIYGQVIEGQGRLAALRFGGD

950   GDELKRGGKPPVNHVNIVVQQALPVTLNEQGEGSVTLPIGDFNGELRVMA

1000  QAWTADDFGSNESKVIVAAPVIAELNMPRFMASGDTSRLTLDITNLTDKP

1050  QKLNVALTASGLLELVSDSPAAVELAPGVRTTLFIPVRALPGYGDGEIQA
```

1100 TISGLALPGETVADQHKQWKIGVRPAFPAQTVNYGTALQPGETWAIPADG

1150 LQNFSPVTLEGQLLLSGKPPLNIARYIKELKAYPYGCLEQTASGLFPSLY

1200 TNAAQLQALGIKGDSDEKRRASVDIGISRLLQMQRDNGGFALWDKNGDEE

1250 YWLTAYVMDFLVRAGEQGYSVPTDAINRGNERLLRYLQDPGMMSIPYADN

1300 LKASKFAVQSYAALVLARQQKAPLGALREIWEHRADAASGLPLLQLGVAL

1350 KTMGDATRGEEAIALALKTPRNSDERIWLGDYGSSLRDNALMLSLLEENK

1400 LLPDEQYTLLNTLSQQAFGERWLSTQESNALFLAARTIQDLPGKWQAQTS

1450 FSAEQLTGEKAQNSNLNSDQLVTLQVSNSGDQPLWLRMDASGYPQSAPLP

1500 ANNVLQIERHILGTDGKSKSLDSLRSGDLVLVWLQVKASNSVPDALVVDL

1550 LPAGLELENQNLANGSASLEQSGGEVQNLLNQMQQASIKHIEFRDDRFVA

1600 AVAVDEYQPVTLVYLARAVTPGTYQVPQPMVESMYVPQWRATGAAEDLLI

1650 VRP

sp|P76190|MEPH_ECOLI Murein DD-endopeptidase MepH OS=Escherichia coli (strain K12) OX=83333 GN=mepH PE=1 SV=1

                                  ****
0   MARINRISITLCALLFTTLPLTPMAHASKQARESSATTHITKKADKKKST
      ****
50   ATTKKTQKTAKKAASKSTTKSKTASSVKKSSITASKNAKTRSKHAVNKTA

100   SASFTEKCTKRKGYKSHCVKVKNAASGTLADAHKAKVQKATKVAMNKLMQ

150   QIGKPYRWGGSSPRTGFDCSGLVYYAYKDLVKIRIPRTANEMYHLRDAAP

200   IERSELKNGDLVFFRTQGRGTADHVGVYVGNGKFIQSPRTGQEIQITSLS

250   EDYWQRHYVGARRVMTPKTLR

sp|P76128|DDPA_ECOLI Probable D,D-dipeptide-binding periplasmic protein DdpA OS=Escherichia coli (strain K12) OX=83333 GN=ddpA PE=2 SV=1

0   MKRSISFRPTLLALVLATNFPVAHAAVPKDMLVIGKAADPQTLDPAVTID
                     ****
50   NNDWTVTYPSYQRLVQYKTDGDKGSTDVEGDLASSWKASDDQKEWTFTLK
      ****
100   DNAKFADGTPVTAEAVKLSFERLLKIGQGPAEAFPKDLKIDAPDEHTVKF

150   TLSQPFAPFLYTLANDGASIINPAVLKEHAADDARGFLAQNTAGSGPFML

200   KSWQKGQQLVLVPNPHYPGNKPNFKRVSVKIIGESASRRLQLSRGDIDIA

250   DALPVDQLNALKQENKVNVAEYPSLRVTYLYLNNSKAPLNQADLRRAISW
                                           ****

300   STDYQGMVNGILSGNGKQMRGPIPEGMWGYDATAMQYNHDETKAKAEWDK
      ****
350   VTSKPTSLTFLYSDNDPNWEPIALATQSSLNKLGIIVKLEKLANATMRDR
                                  ****
400   VGKGDYDIAIGNWSPDFADPYMFMNYWFESDKKGLPGNRSFYENSEVDKL

450   LRNALATTDQTQRTRDYQQAQKIVIDDAAYVYLFQKNYQLAMNKEVKGFV

500   FNPMLEQVFNINTMSK

sp|P0ADY1|PPID_ECOLI Peptidyl-prolyl cis-trans isomerase D OS=Escherichia coli (strain K12) OX=83333 GN=ppiD PE=1 SV=1

0   MMDSLRTAANSLVLKIIFGIIIVSFILTGVSGYLIGGGNNYAAKVNDQEI

50   SRGQFENAFNSERNRMQQQLGDQYSELAANEGYMKTLRQQVLNRLIDEAL

100   LDQYARELKLGISDEQVKQAIFATPAFQVDGKFDNSRYNGILNQMGMTAD

150   QYAQALRNQLTTQQLINGVAGTDFMLKGETDELAALVAQQRVVREATIDV

200   NALAAKQPVTEQEIASYYEQNKNNFMTPEQFRVSYIKLDAATMQQPVSDA

250   DIQSYYDQHQDQFTQPQRTRYSIIQTKTEDEAKAVLDELNKGGDFAALAK

300   EKSADIISARNGGDMGWLEDATIPDELKNAGLKEKGQLSGVIKSSVGFLI
              ****
350   VRLDDIQPAKVKSLDEVRDDIAAKVKHEKALDAYYALQQKVSDAASNDTE

400   SLAGAEQAAGVKATQTGWFSKDNLPEELNFKPVADAIFNGGLVGENGAPG

450   INSDIITVDGDRAFVLRISEHKPEAVKPLADVQEQVKALVQHNKAEQQAK

500   VDAEKLLVDLKAGKGAEAMQAAGLKFGEPKTLSRSGRDPISQAAFALPLP

550   AKDKPSYGMATDMQGNVVLLALDEVKQGSMPEDQKKAMVQGITQNNAQIV

600   FEALMSNLRKEAKIKIGDALEQQ

sp|P76115|YNCD_ECOLI Probable TonB-dependent receptor YncD OS=Escherichia coli (strain K12) OX=83333 GN=yncD PE=3 SV=1

0   MKIFSVRQTVLPALLVLSPVVFAADEQTMIVSAAPQVVSELDTPAAVSVV

50   DGEEMRLATPRINLSESLTGVPGLQVQNRQNYAQDLQLSIRGFGSRSTYG

100   IRGIRLYVDGIPATMPDGQGQTSNIDLSSVQNVEVLRGPFSALYGNASGG

150   VMNVTTQTGQQPPTIEASSYYGSFGSWRYGLKATGATGDGTQPGDVDYTV
                                  ****
200   STTRFTTHGYRDHSGAQKNLANAKLGVRIDEASKLSLIFNSVDIKADDPG

250   GLTKAEWKANPQQAPRAEQYDTRKTIKQTQAGLRYERSLSSRDDMSVMMY

300   AGERETTQYQSIPMAPQLNPSHAGGVITLQRHYQGIDSRWTHRGELGVPV

350   TFTTGLNYENMSENRKGYNNFRLNSGMPEYGQKGELRRDERNLMWNIDPY

400   LQTQWQLSEKLSLDAGVRYSSVWFDSNDHYVTPGNGDDSGDASYHKWLPA

450   GSLKYAMTDAWNIYLAAGRGFETPTINELSYRADGQSGMNLGLKPSTNDT

500   IEIGSKTRIGDGLLSLALFQTDTDDEIVVDSSSGGRTTYKNAGKTRRQGA

550   ELAWDQRFAGDFRVNASWTWLDATYRSNVCNEQDCNGNRMPGIARNMGFA

600   SIGYVPEDGWYAGTEARYMGDIMADDENTAKAPSYTLVGLFTGYKYNYHN

650   LTVDLFGRVDNLFDKEYVGSVIVNESNGRYYEPSPGRNYGVGMNIAWRFE

700

sp|P0A9N4|PFLA_ECOLI Pyruvate formate-lyase 1-activating enzyme OS=Escherichia coli (strain K12) OX=83333 GN=pflA PE=1 SV=2

0   MSVIGRIHSFESCGTVDGPGIRFITFFQGCLMRCLYCHNRDTWDTHGGKE

50   VTVEDLMKEVVTYRHFMNASGGGVTASGGEAILQAEFVRDWFRACKKEGI

100   HTCLDTNGFVRRYDPVIDELLEVTDLVMLDLKQMNDEIHQNLVGVSNHRT

150   LEFAKYLANKNVKVWIRYVVVPGWSDDDDSAHRLGEFTRDMGNVEKIELL
                ****
200   PYHELGKHKWVAMGEEYKLDGVKPPKKETMERVKGILEQYGHKVMF

sp|P0AEU0|HISJ_ECOLI Histidine-binding periplasmic protein OS=Escherichia coli (strain K12) OX=83333 GN=hisJ PE=1 SV=1

0   MKKLVLSLSLVLAFSSATAAFAAIPQNIRIGTDPTYAPFESKNSQGELVG
                                 ****
50   FDIDLAKELCKRINTQCTFVENPLDALIPSLKAKKIDAIMSSLSITEKRQ

100   QEIAFTDKLYAADSRLVVAKNSDIQPTVESLKGKRVGVLQGTTQETFGNE

150   HWAPKGIEIVSYQGQDNIYSDLTAGRIDAAFQDEVAASEGFLKQPVGKDY

200   KFGGPSVKDEKLFGVGTGMGLRKEDNELREALNKAFAEMRADGTYEKLAK

250   KYFDFDVYGG

sp|P45763|GSPL_ECOLI Putative type II secretion system protein L OS=Escherichia coli (strain K12) OX=83333 GN=gspL PE=2 SV=2

0   MPESLMVIRSSSTLRKHWEWMTFSADSVSSVHTLTDDLPLESLADQPGAG

50   NVHLLIPPEGLLYRSLTLPNAKYKLTAQTLQWLAEETLPDNTQDWHWTVV

100   DKQNESVEVIGIQSEKLSRYLERLHTAGLNVTRVLPDGCYLPWEVDSWTL

```
150   VNQQTSWLIRSAAHAFNELDEHWLQHLAAQFPPENMLCYGVVPHGVAAAN

200   PLIQHPEIPSLSLYSADIAFQRYDMLHGIFRKQKTVSKSGKWLARLAVSC

250   LVLAILSFVGSRSIALWHTLKIEDQLQQQQQETWQRYFPQIKRTHNFRFY
                                      ******************
300   FKQQLAQQYPEAVPLLYHLQTLLLEHPELQLMEANYSQKQKSLTLKMSAK
      ******
350   SEANIDRFCELTQSWLPMEKTEKDPVSGVWTVRNSGK

sp|P36659|CBPA_ECOLI Curved DNA-binding protein OS=Escherichia coli
(strain K12) OX=83333 GN=cbpA PE=1 SV=2
                                        ****************
0     MELKDYYAIMGVKPTDDLKTIKTAYRRLARKYHPDVSKEPDAEARFKEVA
      ****
50    EAWEVLSDEQRRAEYDQMWQHRNDPQFNRQFHHGDGQSFNAEDFDDIFSS

100   IFGQHARQSRQRPATRGHDIEIEVAVFLEETLTEHKRTISYNLPVYNAFG

150   MIEQEIPKTLNVKIPAGVGNGQRIRLKGQGTPGENGGPNGDLWLVIHIAP

200   HPLFDIVGQDLEIVVPVSPWEAALGAKVTVPTLKESILLTIPPGSQAGQR

250   LRVKGKGLVSKKQTGDLYAVLKIVMPPKPDENTAALWQQLADAQSSFDPR

300   KDWGKA

sp|P0CF66|INSE1_ECOLI Transposase InsE for insertion sequence IS3A
OS=Escherichia coli (strain K12) OX=83333 GN=insE1 PE=3 SV=1
        ***********************
0     MTKTVSTSKKPRKQHSPEFRSEALKLAERIGVTAAARELSLYESQLYNWR

50    SKQQNQQTSSERELEMSTEIARLKRQLAERDEELAILQKAATYFAKRLK

sp|P76516|YFDT_ECOLI Uncharacterized protein YfdT OS=Escherichia coli
(strain K12) OX=83333 GN=yfdT PE=4 SV=1
          **************************
0     MTTFTNKELIKEIKERISSLEVRDDIERRAYEIALVSLEVEPDEREAYEL

50    FMEKRFGDLVDRRRAKNGDNEYMAWDMTLGWIIWQQRAGIHFSTMSQQEV

100   K

sp|P50457|PUUE_ECOLI 4-aminobutyrate aminotransferase PuuE OS=Escherichia
coli (strain K12) OX=83333 GN=puuE PE=1 SV=1

0     MSNNEFHQRRLSATPRGVGVMCNFFAQSAENATLKDVEGNEYIDFAAGIA

50    VLNTGHRHPDLVAAVEQQLQQFTHTAYQIVPYESYVTLAEKINALAPVSG

100   QAKTAFFTTGAEAVENAVKIARAHTGRPGVIAFSGGFHGRTYMTMALTGK
                                 ********************
150   VAPYKIGFGPFPGSVYHVPYPSDLHGISTQDSLDAIERLFKSDIEAKQVA
```

200 AIIFEPVQGEGGFNVAPKELVAAIRRLCDEHGIVMIADEVQSGFARTGKL

250 FAMDHYADKPDLMTMAKSLAGGMPLSGVVGNANIMDAPAPGGLGGTYAGN

300 PLAVAAAHAVLNIIDKESLCERANQLGQRLKNTLIDAKESVPAIAAVRGL

350 GSMIAVEFNDPQTGEPSAAIAQKIQQRALAQGLLLLTCGAYGNVIRFLYP

400 LTIPDAQFDAAMKILQDALSD

sp|P28912|YHHI_ECOLI H repeat-associated putative transposase YhhI OS=Escherichia coli (strain K12) OX=83333 GN=yhhI PE=3 SV=1

0 MELKKLMEHISIIPDYRQTWKVEHKLSDILLLTICAVISGAEGWEDIEDF
**

50 GETHLDFLKQYGDFENGIPVHDTIARVVSCISPAKFHECFINWMRDCHSS
****

100 DDKDVIAIDGKTLRHSYDKSRRRGAIHVISAFSTMHSLVIGQIKTDEKSN

150 EITAIPELLNMLDIKGKIITTDAMGCQKDIAEKIQKQGGDYLFAVKGTQG

200 RLNKAFEEKFPLKELNNPEHDSYAISEKSHGREEIRLHIVCDVPDELIDF

250 TFEWKGLKKLCVAVSFRSIIAEQKKEPEMTVRYYISSADLTAEKFATAIR

300 NHWHVENKLHWRLDVVMNEDDCKIRRGNAAELFSGIRHIAINILTNDKVF

350 KAGLRRKMRKAAMDRNYLASVLAGSGLS

sp|P77536|YKGF_ECOLI Uncharacterized electron transport protein YkgF OS=Escherichia coli (strain K12) OX=83333 GN=ykgF PE=3 SV=1

0 MSIKTSNTDFKTRIRQQIEDPIMRKAVANAQQRIGANRQKMVDELGHWEE

50 WRDRAAQIRDHVLSNLDAYLYQLSEKVTQNGGHVYFARTKEDATRYILQV

100 AQRKNARKVVKSKSMVTEEIGVNHVLQDAGIQVIETDLGEYILQLDQDPP

150 SHVVVPAIHKDRHQIRRVLHERLGYEGPETPEAMTLFIRQKIREDFLSAE

200 IGITGCNFAVAETGSVCLVTNEGNARMCTTLPKTHIAVMGMERIAPTFAE

250 VDVLITMLARSAVGARLTGYNTWLTGPREAGHVDGPEEFHLVIVDNGRSE

300 VLASEFRDVLRCIRCGACMNTCPAYRHIGGHGYGSIYPGPIGAVISPLLG

350 GYKDFKDLPYACSLCTACDNVCPVRIPLSKLILRHRRVMAEKGITAKAEQ

400 RAIKMFAYANSHPGLWKVGMMAGAHAASWFINGGKTPLKFGAISDWMEAR
****

450 DLPEADGESFRSWFKKHQAQEKKNG

sp|P32689|YJBH_ECOLI Uncharacterized lipoprotein YjbH OS=Escherichia coli (strain K12) OX=83333 GN=yjbH PE=3 SV=1

```
0     MKKRHLLSLLALGISTACYGETYPAPIGPSQSDFGGVGLLQTPTARMARE
                                                ********** 
50    GELSLNYRDNDQYRYYSASVQLFPWLETTLRYTDVRTRQYSSVEAFSGDQ
      **********
100   TYKDKAFDLKLRLWEESYWLPQVAVGARDIGGTGLFDAEYLVASKAWGPF

150   DFTLGLGWGYLGTSGNVKNPLCSASDKYCYRDNSYKQAGSIDGSQMFHGP

200   ASLFGGVEYQTPWQPLRLKLEYEGNNYQQDFAGKLEQKSKFNVGAIYRVT

250   DWADVNLSYERGNTFMFGVTLRTNFNDLRPSYNDNARPQYQPQPQDAILQ

300   HSVVANQLTLLKYNAGLADPQIQAKGDTLYVTGEQVKYRDSREGIIRANR

350   IVMNDLPDGIKTIRITENRLNMPQVTTETDVASLKNHLAGEPLGHETTLA

400   QKRVEPVVPQSTEQGWYIDKSRFDFHIDPVLNQSVGGPENFYMYQLGVMG

450   TADLWLTDHLLTTGSLFANLANNYDKFNYTNPPQDSHLPRVRTHVREYVQ

500   NDVYVNNLQANYFQHLGNGFYGQVYGGYLETMFGGAGAEVLYRPLDSNWA

550   FGLDANYVKQRDWRSAKDMMKFTDYSVKTGHLTAYWTPSFAQDVLVKASV

600   GQYLAGDKGGTLEIAKRFDSGVVVGGYATITNVSKEEYGEGDFTKGVYVS

650   VPLDLFSSGPTRSRAAIGWTPLTRDGGQQLGRKFQLYDMTSDRSVNFR
```

sp|P31120|GLMM_ECOLI Phosphoglucosamine mutase OS=Escherichia coli (strain K12) OX=83333 GN=glmM PE=1 SV=3

```
0     MSNRKYFGTDGIRGRVGDAPITPDFVLKLGWAAGKVLARHGSRKIIIGKD

50    TRISGYMLESALEAGLAAAGLSALFTGPMPTPAVAYLTRTFRAEAGIVIS
                      **********************************
100   ASHNPFYDNGIKFFSIDGTKLPDAVEEAIEAEMEKEISCVDSAELGKASR
      ***
150   IVDAAGRYIEFCKATFPNELSLSELKIVVDCANGATYHIAPNVLRELGAN

200   VIAIGCEPNGVNINAEVGATDVRALQARVLAEKADLGIAFDGDGDRVIMV

250   DHEGNKVDGDQIMYIIAREGLRQGQLRGGAVGTLMSNMGLELALKQLGIP

300   FARAKVGDRYVLEKMQEKGWRIGAENSGHVILLDKTTTGDGIVAGLQVLA

350   AMARNHMSLHDLCSGMKMFPQILVNVRYTAGSGDPLEHESVKAVTAEVEA

400   ALGNRGRVLLRKSGTEPLIRVMVEGEDEAQVTEFAHRIADAVKAV
```

sp|P76641|GUAD_ECOLI Guanine deaminase OS=Escherichia coli (strain K12) OX=83333 GN=guaD PE=1 SV=1

0   MMSGEHTLKAVRGSFIDVTRTIDNPEEIASALRFIEDGLLLIKQGKVEWF

50   GEWENGKHQIPDTIRVRDYRGKLIVPGFVDTHIHYPQSEMVGAYGEQLLE

100   WLNKHTFPTERRYEDLEYAREMSAFFIKQLLRNGTTTALVFGTVHPQSVD

150   ALFEAASHINMRMIAGKVMMDRNAPDYLLDTAESSYHQSKELIERWHKNG

200   RLLYAITPRFAPTSSPEQMAMAQRLKEEYPDTWVHTHLCENKDEIAWVKS
                                ****
250   LYPDHDGYLDVYHQYGLTGKNCVFAHCVHLEEKEWDRLSETKSSIAFCPT
      *
300   SNLYLGSGLFNLKKAWQKKVKVGMGTDIGAGTTFNMLQTLNEAYKVLQLQ

350   GYRLSAYEAFYLATLGGAKSLGLDDLIGNFLPGKEADFVVMEPTATPLQQ

400   LRYDNSVSLVDKLFVMMTLGDDRSIYRTYVDGRLVYERN

sp|P64423|ZNTB_ECOLI Zinc transport protein ZntB OS=Escherichia coli (strain K12) OX=83333 GN=zntB PE=1 SV=1

0   MEAIKGSDVNVPDAVFAWMLDGRGGVKPLENTDVIDEAHPCWLHLNYVHH

50   DSAQWLATTPLLPNNVRDALAGESTRPRVSRLGEGTLITLRCINGSTDER

100   PDQLVAMRVYMDGRLIVSTRQRKVLALDDVVSDLEEGTGPTDCGGWLVDV
          ****
150   CDALTDHSSEFIEQLHDKIIDLEDNLLDQQIPPRGFLALLRKQLIVMRRY

200   MAPQRDVYARLASERLPWMSDDQRRRMQDIADRLGRGLDEIDACIARTGV

250   MADEIAQVMQENLARRTYTMSLMAMVFLPSTFLTGLFGVNLGGIPGGGWQ

300   FGFSIFCILLVVLIGGVALWLHRSKWL

sp|P42911|PTPD_ECOLI N-acetylgalactosamine permease IID component OS=Escherichia coli (strain K12) OX=83333 GN=agaD PE=3 SV=1
                                                ****
0   MGSEISKKDITRLGFRSSLLQASFNYERMQAGGFTWAMLPILKKIYKDDK
      ****
50   PGLSAAMKDNLEFINTHPNLVGFLMGLLISMEEKGENRDTIKGLKVALFG

100   PIAGIGDAIFWFTLLPIMAGICSSFASQGNLLGPILFFAVYLLIFFLRVG

150   WTHVGYSVGVKAIDKVRENSQMIARSATILGITVIGGLIASYVHINVVTS

200   FAIDNTHSVALQQDFFDKVFPNILPMAYTLLMYYFLRVKKAHPVLLIGVT

250   FVLSIVCSAFGIL

sp|P09549|DEDD_ECOLI Cell division protein DedD OS=Escherichia coli (strain K12) OX=83333 GN=dedD PE=3 SV=2

```
0     MASKFQNRLVGTIVLVALGVIVLPGLLDGQKKHYQDEFAAIPLVPKAGDR

50    DEPDMMPAATQALPTQPPEGAAEEVRAGDAAAPSLDPATIAANNTEFEPE

100   PAPVAPPKPKPVEPPKPKVEAPPAPKPEPKPVVEEKAAPTGKAYVVQLGA
                                            ************
150   LKNADKVNEIVGKLRGAGYRVYTSPSTPVQGKITRILVGPDASKDKLKGS
      ************
200   LGELKQLSGLSGVVMGYTPN
```

sp|P0AAQ6|YBAA_ECOLI Uncharacterized protein YbaA OS=Escherichia coli (strain K12) OX=83333 GN=ybaA PE=4 SV=1

```
0     MKYVDGFVVAVPADKKDAYREMAAKAAPLFKEFGALRIVECWASDVPDGK
             ***********************
50    VTDFRMAVKAEENEEVVFSWIEYPSKEVRDAANQKMMSDPRMKEFGESMP

100   FDGKRMIYGGFESIIDE
```

sp|P0ABA6|ATPG_ECOLI ATP synthase gamma chain OS=Escherichia coli (strain K12) OX=83333 GN=atpG PE=1 SV=1

```
0     MAGAKEIRSKIASVQNTQKITKAMEMVAASKMRKSQDRMAASRPYAETMR

50    KVIGHLAHGNLEYKHPYLEDRDVKRVGYLVVSTDRGLCGGLNINLFKKLL

100   AEMKTWTDKGVQCDLAMIGSKGVSFFNSVGGNVVAQVTGMGDNPSLSELI
                                         ***************
150   GPVKVMLQAYDEGRLDKLYIVSNKFINTMSQVPTISQLLPLPASDDDDLK
      ***************
200   HKSWDYLYEPDPKALLDTLLRRYVESQVYQGVVENLASEQAARMVAMKAA

250   TDNGGSLIKELQLVYNKARQASITQELTEIVSGAAAV
```

sp|P77609|FLXA_ECOLI Protein FlxA OS=Escherichia coli (strain K12) OX=83333 GN=flxA PE=1 SV=1

```
                                         ***************
0     MSVTIQGNTSTVISNNSAPEGTSEIAKITRQIQVLTEKLGKISSEEGMTT
      ******                   *************************
50    QQKKEMAALVQKQIESLWAQLEQLLRQQAEKKNEDATVQPDKKEEKKDDT
      **********
100   NTAGTIDIYV
```

sp|P33913|YEJA_ECOLI Uncharacterized protein YejA OS=Escherichia coli (strain K12) OX=83333 GN=yejA PE=3 SV=3

```
0     MIVRILLLFIALFTFGVQAQAIKESYAFAVLGEPRYAFNFNHFDYVNPAA

50    PKGGQITLSALGTFDNFNRYALRGNPGARTEQLYDTLFTTSDDEPGSYYP

100   LIAESARYADDYSWVEVAINPRARFHDGSPITARDVEFTFQKFMTEGVPQ

150   FRLVYKGTTVKAIAPLTVRIELAKPGKEDMLSLFSLPVFPEKYWKDHKLS
```

200   DPLATPPLASGPYRVTSWKMGQNIVYSRVKDYWAANLPVNRGRWNFDTIR
                                       ****
250   YDYYLDDNVAFEAFKAGAFDLRMENDAKNWATRYTGKNFDKKYIIKDEQK
      ****

300   NESAQDTRWLAFNIQRPVFSDRRVREAITLAFDFEWMNKALFYNAWSRTN

350   SYFQNTEYAARNYPDAAELVLLAPMKKDLPSEVFTQIYQPPVSKGDGYDR

400   DNLLKADKLLNEAGWVLKGQQRVNATTGQPLSFELLLPASSNSQWVLPFQ

450   HSLQRLGINMDIRKVDNSQITNRMRSRDYDMMPRVWRAMPWPSSDLQISW

500   SSEYINSTYNAPGVQSPVIDSLINQIIAAQGNKEKLLPLGRALDRVLTWN

550   YYMLPMWYMAEDRLAWWDKFSQPAVRPIYSLGIDTWWYDVNKAAKLPSAS

600   KQGE

sp|P24216|FLID_ECOLI Flagellar hook-associated protein 2 OS=Escherichia coli (strain K12) OX=83333 GN=fliD PE=1 SV=5

0   MASISSLGVGSGLDLSSILDSLTAAQKATLTPISNQQSSFTAKLSAYGTL

50   KSALTTFQTANTALSKADLFSATSTTSSTTAFSATTAGNAIAGKYTISVT

100   HLAQAQTLTTRTTRDDTKTAIATSDSKLTIQQGGDKDPITIDISAANSSL

150   SGIRDAINNAKAGVSASIINVGNGEYRLSVTSNDTGLDNAMTLSVSGDDA

200   LQSFMGYDASASSNGMEVSVAAQNAQLTVNNVAIENSSNTISDALENITL

250   NLNDVTTGNQTLTITQDTSKAQTAIKDWVNAYNSLIDTFSSLTKYTAVDA
                                                      **
300   GADSQSSSNGALLGDSTLRTIQTQLKSMLSNTVSSSSYKTLAQIGITTDP
      ****

350   SDGKLELDADKLTAALKKDASGVGALIVGDGKKTGITTTIGSNLTSWLST

400   TGIIKAATDGVSKTLNKLTKDYNAASDRIDAQVARYKEQFTQLDVLMTSL

450   NSTSSYLTQQFENNSNSK

sp|P76170|YNFB_ECOLI UPF0482 protein YnfB OS=Escherichia coli (strain K12) OX=83333 GN=ynfB PE=1 SV=1

0   MKITLSKRIGLLAILLPCALALSTTVHAETNKLVIESGDSAQSRQHAAME
                    ****
50   KEQWNDTRNLRQKVNKRTEKEWDKADAAFDNRDKCEQSANINAYWEPNTL

100   RCLDRRTGRVITP

sp|P31827|YDDB_ECOLI Uncharacterized protein YddB OS=Escherichia coli (strain K12) OX=83333 GN=yddB PE=4 SV=2

0   MKRVLIPGVILCGADVAQAVDDKNMYMHFFEEMTVYAPVPVPVNGNTHYT

```
50    SESIERLPTGNGNISDLLRTNPAVRMDSTQSTSLNQGDIRPEKISIHGAS

100   PYQNAYLIDGISATNNLNPANESDASSATNISGMSQGYYLDVSLLDNVTL

150   YDSFVPVEFGRFNGGVIDAKIKRFNADDSKVKLGYRTTRSDWLTSHIDEN

200   NKSAFNQGSSGSTYYSPDFKKNFYTLSFNQELADNFGVTAGLSRRQSDIT

250   RADYVSNDGIVAGRAQYKNVIDTALSKFTWFASDRFTHDLTLKYTGSSRD

300   YNTSTFPQSDREMGNKSYGLAWDMDTQLAWAKLRTTVGWDHISDYTRHDH

350   DIWYTELSCTYGDITGRCTRGGLGHISQAVDNYTFKTRLDWQKFAVGNVS

400   HQPYFGAEYIYSDAWTERHNQSESYVINAAGKKTNHTIYHKGKGRLGIDN

450   YTLYMADRISWRNVSLMPGVRYDYDNYLSNHNISPRFMTEWDIFANQTSM

500   ITAGYNRYYGGNILDMGLRDIRNSWTESVSGNKTLTRYQDLKTPYNDELA

550   MGLQQKIGKNVIARANYVYREAHDQISKSSRTDSATKTTITEYNNDGKTK

600   THSFSLSFELAEPLHIRQVDINPQIVFSYIKSKGNLSLNNGYEESNTGDN

650   QVVYNGNLVSYDSVPVADFNNPLKISLNMDFTHQPSGLVWANTLAWQEAR
                ***************************
700   KARIILGKTNAQYISEYSDYKQYVDEKLDSSLTWDTRLSWTPQFLQQQNL

750   TISADILNVLDSKTAVDTTNTGVATYASGRTFWLDVSMKF
```

sp|P0AAG3|GLTL_ECOLI Glutamate/aspartate import ATP-binding protein GltL OS=Escherichia coli (strain K12) OX=83333 GN=gltL PE=3 SV=1

```
0     MITLKNVSKWYGHFQVLTDCSTEVKKGEVVVVCGPSGSGKSTLIKTVNGL

50    EPVQQGEITVDGIVVNDKKTDLAKLRSRVGMVFQHFELFPHLSIIENLTL

100   AQVKVLKRDKAPAREKALKLLERVGLSAHANKFPAQLSGGQQQRVAIARA

150   LCMDPIAMLFDEPTSALDPEMINEVLDVMVELANEGMTMMVVTHEMGFAR
            *********************************
200   KVANRVIFMDEGKIVEDSPKDAFFDDPKSDRAKDFLAKILH
```

sp|P0AGK8|ISCR_ECOLI HTH-type transcriptional regulator IscR OS=Escherichia coli (strain K12) OX=83333 GN=iscR PE=1 SV=1

```
0     MRLTSKGRYAVTAMLDVALNSEAGPVPLADISERQGISLSYLEQLFSRLR
                        ********************
50    KNGLVSSVRGPGGGYLLGKDASSIAVGEVISAVDESVDATRCQGKGGCQG

100   GDKCLTHALWRDLSDRLTGFLNNITLGELVNNQEVLDVSGRQHTHDAPRT

150   RTQDAIDVKLRA
```

sp|P32125|MOBB_ECOLI Molybdopterin-guanine dinucleotide biosynthesis adapter protein OS=Escherichia coli (strain K12) OX=83333 GN=mobB PE=1 SV=3

```
0     MAGKTMIPLLAFAAWSGTGKTTLLKKLIPALCARGIRPGLIKHTHHDMDV
                                       *****************
50    DKPGKDSYELRKAGAAQTIVASQQRWALMTETPDEEELDLQFLASRMDTS
      ***
100   KLDLILVEGFKHEEIAKIVLFRDGAGHRPEELVIDRHVIAVASDVPLNLD

150   VALLDINDVEGLADFVVEWMQKQNG
```

sp|P67662|AAER_ECOLI HTH-type transcriptional activator AaeR OS=Escherichia coli (strain K12) OX=83333 GN=aaeR PE=3 SV=1

```
                                 *********************
0     MERLKRMSVFAKVVEFGSFTAAARQLQMSVSSISQTVSKLEDELQVKLLN

50    RSTRSIGLTEAGRIYYQGCRRMLHEVQDVHEQLYAFNNTPIGTLRIGCSS

100   TMAQNVLAGLTAKMLKEYPGLSVNLVTGIPAPDLIADGLDVVIRVGALQD

150   SSLFSRRLGAMPMVVCAAKSYLTQYGIPEKPADLSSHSWLEYSVRPDNEF

200   ELIAPEGISTRLIPQGRFVTNDPMTLVRWLTAGAGIAYVPLMWVINEINR

250   GELEILLPRYQSDPRPVYALYTEKDKLPLKVQVVINSLTDYFVEVGKLFQ

300   EMHGRGKEK
```

sp|P77239|CUSB_ECOLI Cation efflux system protein CusB OS=Escherichia coli (strain K12) OX=83333 GN=cusB PE=1 SV=1

```
0     MKKIALIIGSMIAGGIISAAGFTWVAKAEPPAEKTSTAERKILFWYDPMY
              **********************
50    PNTRFDKPGKSPFMDMDLVPKYADEESSASGVRIDPTQTQNLGVKTATVT

100   RGPLTFAQSFPANVSYNEYQYAIVQARAAGFIDKVYPLTVGDKVQKGTPL

150   LDLTIPDWVEAQSEYLLLRETGGTATQTEGILERLRLAGMPEADIRRLIA

200   TQKIQTRFTLKAPIDGVITAFDLRAGMNIAKDNVVAKIQGMDPVWVTAAI

250   PESIAWLVKDASQFTLTVPARPDKTLTIRKWTLLPGVDAATRTLQLRLEV

300   DNADEALKPGMNAWLQLNTASEPMLLIPSQALIDTGSEQRVITVDADGRF

350   VPKRVAVFQASQGVTALRSGLAEGEKVVSSGLFLIDSEANISGALERMRS

400   ESATHAH
```

sp|P33129|HTRE_ECOLI Outer membrane usher protein HtrE OS=Escherichia coli (strain K12) OX=83333 GN=htrE PE=1 SV=3

```
0      MTIEYTKNYHHLTRIATFCALLYCNTAFSAELVEYDHTFLMGQNASNIDL

50     SRYSEGNPAIPGVYDVSVYVNDQPIINQSITFVAIEGKKNAQACITLKNL

100    LQFHINSPDINNEKAVLLARDETLGNCLNLTEIIPQASVRYDVNDQRLDI

150    DVPQAWVMKNYQNYVDPSLWENGINAAMLSYNLNGYHSETPGRKNESIYA

200    AFNGGMNLGAWRLRASGNYNWMTDSGSNYDFKNRYVQRDIASLRSQLILG

250    ESYTTGETFDSVSIRGIRLYSDSRMLPPTLASFAPIIHGVANTNAKVTIT
                            ********************
300    QGGYKIYETTVPPGAFVIDDLSPSGYGSDLIVTIEESDGSKRTFSQPFSS

350    VVQMLRPGVGRWDISGGQVLKDDIQDEPNLFQASYYYGLNNYLTGYTGIQ

400    ITDNNYTAGLLGLGLNTSVGAFSFDVTHSNVRIPDDKTYQGQSYRVSWNK

450    LFEETSTSLNIAAYRYSTQNYLGLNDALTLIDEVKHPEQDLEPKSMRNYS
                   ************************
500    RMKNQVTVSINQPLKFEKKDYGSFYLSGSWSDYWASGQNRSNYSIGYSNS

550    TSWGSYSVSAQRSWNEDGDTDDSVYLSFTIPIEKLLGTEQRTSGFQSIDT

600    QISSDFKGNNQLNVSSSGYSDNARVSYSVNTGYTMNKASKDLSYVGGYAS

650    YESPWGTLAGSISANSDNSRQVSLSTDGGFVLHSGGLTFSNDSFSDSDTL

700    AVVQAPGAQGARINYGNSTIDRWGYGVTSALSPYHENRIALDINDLENDV

750    ELKSTSAVAVPRQGSVVFADFETVQGQSAIMNITRSDGKNIPFAADIYDE

800    QGNVIGNVGQGGQAFVRGIEQQGNISIKWLEQSKPVSCLAHYQQSPEAEK

850    IAQSIILNGIRCQIQ
```

sp|P69826|PTMCB_ECOLI PTS system mannitol-specific cryptic EIICB component OS=Escherichia coli (strain K12) OX=83333 GN=cmtA PE=3 SV=1

```
0      MENKSARAKVQAFGGFLTAMVIPNIGAFIAWGFITALFIPTGWLPNEHFA

50     KIVGPMITYLLPVMIGSTGGHLVGGKRGAVMGGIGTIGVIVGAEIPMFLG

100    SMIMGPLGGLVIKYVDKALEKRIPAGFEMVINNFSLGIAGMLLCLLGFEV

150    IGPAVLIANTFVKECIEALVHAGYLPLLSVINEPAKVLFLNNAIDQGVYY

200    PLGMQQASVNGKSIFFMVASNPGPGLGLLLAFTLFGKGMSKRSAPGAMII

250    HFLGGIHELYFPYVLMKPLTIIAMIAGGMSGTWMFNLLDGGLVAGPSPGS
                                   **********************
300    IFAYLALTPKGSFLATIAGVTVGTLVSFAITSLILKMEKTVETESEDEFA
       *******
350    QSANAVKAMKQEGAFSLSRVKRIAFVCDAGMGSSAMGATTFRKRLEKAGL
```

400   AIEVKHYAIENVPADADIVVTHASLEGRVKRVTDKPLILINNYIGDPKLD

450   TLFNQLTAEHKH

sp|P0A6Z1|HSCA_ECOLI Chaperone protein HscA OS=Escherichia coli (strain K12) OX=83333 GN=hscA PE=1 SV=1

0   MALLQISEPGLSAAPHQRRLAAGIDLGTTNSLVATVRSGQAETLADHEGR

50   HLLPSVVHYQQQGHSVGYDARTNAALDTANTISSVKRLMGRSLADIQQRY

100   PHLPYQFQASENGLPMIETAAGLLNPVRVSADILKALAARATEALAGELD

150   GVVITVPAYFDDAQRQGTKDAARLAGLHVLRLLNEPTAAAIAYGLDSGQE

200   GVIAVYDLGGGTFDISILRLSRGVFEVLATGGDSALGGDDFDHLLADYIR

250   EQAGIPDRSDNRVQRELLDAAIAAKIALSDADSVTVNVAGWQGEISREQF

300   NELIAPLVKRTLLACRRALKDAGVEADEVLEVVMVGGSTRVPLVRERVGE

350   FFGRPPLTSIDPDKVVAIGAAIQADILVGNKPDSEMLLLDVIPLSLGLET

400   MGGLVEKVIPRNTTIPVARAQDFTTFKDGQTAMSIHVMQGERELVQDCRS
                                                        **
450   LARFALRGIPALPAGGAHIRVTFQVDADGLLSVTAMEKSTGVEASIQVKP
      ****
500   SYGLTDSEIASMIKDSMSYAEQDVKARMLAEQKVEAARVLESLHGALAAD

550   AALLSAAERQVIDDAAAHLSEVAQGDDVDAIEQAIKNVDKQTQDFAARRM

600   DQSVRRALKGHSVDEV

sp|P0AA25|THIO_ECOLI Thioredoxin 1 OS=Escherichia coli (strain K12) OX=83333 GN=trxA PE=1 SV=2
       ****
0   MSDKIIHLTDDSFDTDVLKADGAILVDFWAEWCGPCKMIAPILDEIADEY

50   QGKLTVAKLNIDQNPGTAPKYGIRGIPTLLLFKNGEVAATKVGALSKGQL

100   KEFLDANLA

sp|P0AG78|SUBI_ECOLI Sulfate-binding protein OS=Escherichia coli (strain K12) OX=83333 GN=sbp PE=1 SV=1

0   MNKWGVGLTFLLAATSVMAKDIQLLNVSYDPTRELYEQYNKAFSAHWKQQ

50   TGDNVVIRQSHGGSGKQATSVINGIEADVVTLALAYDVDAIAERGRIDKE

100   WIKRLPDNSAPYTSTIVFLVRKGNPKQIHDWNDLIKPGVSVITPNPKSSG

150   GARWNYLAAWGYALHHNNNDQAKAQDFVRALYKNVEVLDSGARGSTNTFV
                                     ****

```
200   ERGIGDVLIAWENEALLAANELGKDKFEIVTPSESILAEPTVSVVDKVVE
      **
250   KKGTKEVAEAYLKYLYSPEGQEIAAKNYYRPRDAEVAKKYENAFPKLKLF

300   TIDEEFGGWTKAQKEHFANGGTFDQISKR
```

sp|P0A6T9|GCSH_ECOLI Glycine cleavage system H protein OS=Escherichia coli (strain K12) OX=83333 GN=gcvH PE=1 SV=2

```
0     MSNVPAELKYSKEHEWLRKEADGTYTVGITEHAQELLGDMVFVDLPEVGA
                                                      **
50    TVSAGDDCAVAESVKAASDIYAPVSGEIVAVNDALSDSPELVNSEPYAGG
      *************************
100   WIFKIKASDESELESLLDATAYEALLEDE
```

sp|P52123|YFJH_ECOLI Uncharacterized protein YfjH OS=Escherichia coli (strain K12) OX=83333 GN=yfjH PE=4 SV=1

```
0     MGIFKAKNPCTKNTIFTTSNTLIYGGFMISLNDFYEQICRKRRDLAYHMS

50    ECEWAVDTDVLEEDHPEIRIELGRMREQFWSSEKIGTRVRLYSCDVPWET

100   RHHTVNGQLEIKEEYTELYDPAQECWKNLSSNLTKETFLPLVIEPFSIND

150   IFKAHLMFASISFFWGKSIMSENENVAFKAFHRAAELFDKCIGMTWFNIS
        ***********************
200   VCNQKKLSEVRRSAGKKGGKSKAEVYHIIQLKLVELINDSVPNDGWKNKV
                                                      **
250   VAVNELIEPLWDFIQMSEFEINNQNKKYRVATMSQDALVDTILNQWSLKN
      ******************
300   EDVKQAFDSAVRRKKRSK
```

sp|P0ACS9|ACRR_ECOLI HTH-type transcriptional regulator AcrR OS=Escherichia coli (strain K12) OX=83333 GN=acrR PE=1 SV=1

```
                                                     ***
0     MARKTKQEAQETRQHILDVALRLFSQQGVSSTSLGEIAKAAGVTRGAIYW
      ************************
50    HFKDKSDLFSEIWELSESNIGELELEYQAKFPGDPLSVLREILIHVLEST

100   VTEERRRLLMEIIFHKCEFVGEMAVVQQAQRNLCLESYDRIEQTLKHCIE

150   AKMLPADLMTRRAAIIMRGYISGLMENWLFAPQSFDLKKEARDYVAILLE

200   MYLLCPTLRNPATNE
```

sp|P45760|GSPI_ECOLI Putative type II secretion system protein I OS=Escherichia coli (strain K12) OX=83333 GN=gspI PE=2 SV=2

```
0     MNKQSGMTLLEVLLAMSIFTAVALTLMSSMQGQRNAIERMRNETLALWIA
      ***************************************
50    DNQLQSQDSFGEENTSSSGKELINGEEWNWRSDIHSSKDGTLLERTITVT

100   LPSGQTTSLTRYQSIDNKSGQAQDD
```

```
sp|P69346|YEFM_ECOLI Antitoxin YefM OS=Escherichia coli (strain K12)
OX=83333 GN=yefM PE=1 SV=1

0     MRTISYSEARQNLSATMMKAVEDHAPILITRQNGEACVLMSLEEYNSLEE
                ***********************
50    TAYLLRSPANARRLMDSIDSLKSGKGTEKDIIE

sp|P0AEG6|DSBC_ECOLI Thiol:disulfide interchange protein DsbC
OS=Escherichia coli (strain K12) OX=83333 GN=dsbC PE=1 SV=1

0     MKKGFMLFTLLAAFSGFAQADDAAIQQTLAKMGIKSSDIQPAPVAGMKTV

50    LTNSGVLYITDDGKHIIQGPMYDVSGTAPVNVTNKMLLKQLNALEKEMIV
                                                   *****
100   YKAPQEKHVITVFTDITCGYCHKLHEQMADYNALGITVRYLAFPRQGLDS
      **********************
150   DAEKEMKAIWCAKDKNKAFDDVMAGKSVAPASCDVDIADHYALGVQLGVS

200   GTPAVVLSNGTLVPGYQPPKEMKEFLDEHQKMTSGK

sp|P76344|ZINT_ECOLI Metal-binding protein ZinT OS=Escherichia coli
(strain K12) OX=83333 GN=zinT PE=1 SV=1

0     MAIRLYKLAVALGVFIVSAPAFSHGHHSHGKPLTEVEQKAANGVFDDANV
                                   ********************
50    QNRTLSDWDGVWQSVYPLLQSGKLDPVFQKKADADKTKTFAEIKDYYHKG
                                           *************
100   YATDIEMIGIEDGIVEFHRNNETTSCKYDYDGYKILTYKSGKKGVRYLFE
      ********
150   CKDPESKAPKYIQFSDHIIAPRKSSHFHIFMGNDSQQSLLNEMENWPTYY

200   PYQLSSEEVVEEMMSH

sp|P0AES9|HDEA_ECOLI Acid stress chaperone HdeA OS=Escherichia coli
(strain K12) OX=83333 GN=hdeA PE=1 SV=1

0     MKKVLGVILGGLLLLPVVSNAADAQKAADNKKPVNSWTCEDFLAVDESFQ
                                           *************
50    PTAVGFAEALNNKDKPEDAVLDVQGIATVTPAIVQACTQDKQANFKDKVK
      **********
100   GEWDKIKKDM

sp|P37629|YHIL_ECOLI Putative uncharacterized protein YhiL OS=Escherichia
coli (strain K12) OX=83333 GN=yhiL PE=5 SV=3
                 ***************************************
0     MKAIDNQIRNISSSHQDKHSDKVNSHQHHGKVDKTHRAKIVEFDKLDNDS

50    QIDNDFGLHIIYFLQHGHWKVNDRSHQMEKVWFYNSEPSIDIQEYNRFAD

100   NTTDTFIFTIIPDNNHVLKLSSPITVTVECKGGYYFINSSGDKSDIIYKV
                       *********************************
150   DGLSIIARNFFTLLSGNFKPDWRWDVSKETFTKEKFDSYVKSVFSKIDFY
      ***
200   KQCGVINPQNANTAYFGDTDGRVGAVLYALLVSGHIGIREKGWSLLCELL
```

```
250   KHEEMASSAYKHKNNKVLYDLLNTRDMILNELHQHVFLKDDAITPCIFLG

300   DHTGDRFSTIFGDKYILTLLNSMRNMEGNKDSRINKNVVVLAGNHEINFN

350   GNYTARLANHKLSAGDTYNLIKTLDVCNYDSERQVLTSHHGIIRDEEKKC

400   YCLGALQVPFNQMKNPTDPEELANIFNKKHKEHMDDPLFHLIRSNTLKPT

450   PVYANYFDNTTDFRPARERIFICGETLKGEDPSKYIRQKYGHHGPGVDHN

500   QQFDNGIMGLNSLKEARDKNNKIIYSSGLSCFQLH
```

sp|P0AFI2|PARC_ECOLI DNA topoisomerase 4 subunit A OS=Escherichia coli (strain K12) OX=83333 GN=parC PE=1 SV=1

```
0     MSDMAERLALHEFTENAYLNYSMYVIMDRALPFIGDGLKPVQRRIVYAMS

50    ELGLNASAKFKKSARTVGDVLGKYHPHGDSACYEAMVLMAQPFSYRYPLV

100   DGQGNWGAPDDPKSFAAMRYTESRLSKYSELLLSELGQGTADWVPNFDGT

150   LQEPKMLPARLPNILLNGTTGIAVGMATDIPPHNLREVAQAAIALIDQPK

200   TTLDQLLDIVQGPDYPTEAEIITSRAEIRKIYENGRGSVRMRAVWKKEDG

250   AVVISALPHQVSGARVLEQIAAQMRNKKLPMVDDLRDESDHENPTRLVIV

300   PRSNRVDMDQVMNHLFATTDLEKSYRINLNMIGLDGRPAVKNLLEILSEW

350   LVFRRDTVRRRLNYRLEKVLKRLHILEGLLVAFLNIDEVIEIIRNEDEPK
                             *************************
400   PALMSRFGLTETQAEAILELKLRHLAKLEEMKIRGEQSELEKERDQLQGI
                             ********************
450   LASERKMNNLLKKELQADAQAYGDDRRSPLQEREEAKAMSEHDMLPSEPV

500   TIVLSQMGWVRSAKGHDIDAPGLNYKAGDSFKAAVKGKSNQPVVFVDSTG

550   RSYAIDPITLPSARGQGEPLTGKLTLPPGATVDHMLMESDDQKLLMASDA

600   GYGFVCTFNDLVARNRAGKALITLPENAHVMPPVVIEDASDMLLAITQAG

650   RMLMFPVSDLPQLSKGKGNKIINIPSAEAARGEDGLAQLYVLPPQSTLTI

700   HVGKRKIKLRPEELQKVTGERGRRGTLMRGLQRIDRVEIDSPRRASSGDS

750   EE
```

sp|P77667|SUFA_ECOLI Protein SufA OS=Escherichia coli (strain K12) OX=83333 GN=sufA PE=1 SV=1

```
0     MDMHSGTFNPQDFAWQGLTLTPAAAIHIRELVAKQPGMVGVRLGVKQTGC
         *************************
50    AGFGYVLDSVSEPDKDDLLFEHDGAKLFVPLQAMPFIDGTEVDFVREGLN
```

```
100   QIFKFHNPKAQNECGCGESFGV
```

sp|P39401|OPGB_ECOLI Phosphoglycerol transferase I OS=Escherichia coli (strain K12) OX=83333 GN=mdoB PE=3 SV=2

```
0     MSELLSFALFLASVLIYAWKAGRNTWWFAATLTVLGLFVVLNITLFASDY

50    FTGDGINDAVLYTLTNSLTGAGVSKYILPGIGIVLGLTAVFGALGWILRR
                                         ***************
100   RRHHPHHFGYSLLALLLALGSVDASPAFRQITELVKSQSRDGDPDFAAYY
      *****
150   KEPSKTIPDPKLNLVYIYGESLERTYFDNEAFPDLTPELGALKNEGLDFS

200   HTQQLPGTDYTIAGMVASQCGIPLFAPFEGNASASVSSFFPQNICLGDIL

250   KNSGYQNYFVQGANLRFAGKDVFLKSHGFDHLYGSEELKSVVADPHYRND
        **********************
300   WGFYDDTVLDEAWKKFEELSRSGQRFSLFTLTVDTHHPDGFISRTCNRKK

350   YDFDGKPNQSFSAVSCSQENIATFINKIKASPWFKDTVIVVSSDHLAMNN

400   TAWKYLNKQDRNNLFFVIRGDKPQQETLAVKRNTMDNGATVLDILGGDNY

450   LGLGRSSLSGQSMSEIFLNIKEKTLAWKPDIIRLWKFPKEMKEFTIDQQK

500   NMIAFSGSHFRLPLLLRVSDKRVEPLPESEYSAPLRFQLADFAPRDNFVW

550   VDRCYKMAQLWAPELALSTDWCVSQGQLGGQQIVQHVDKTTWQGKTAFKD

600   TVIDMARYKGNVDTLKIVDNDIRYKADSFIFNVAGAPEEVKQFSGISRPE

650   SWGRWSNAQLGDEVKIEYKHPLPKKFDLVITAKAYGNNASRPIPVRVGNE

700   EQTLVLGNEVTTTTLHFDNPTDADTLVIVPPEPVSTNEGNILGHSPRKLG

750   IGMVEIKVVEREG
```

sp|P25772|LIGB_ECOLI DNA ligase B OS=Escherichia coli (strain K12) OX=83333 GN=ligB PE=1 SV=2

```
                                             *************
0     MKVWMAILIGILCWQSSVWAVCPAWSPARAQEEISRLQQQIKQWDDDYWK
      ****************
50    EGKSEVEDGVYDQLSARLTQWQRCFGSEPRDVMMPPLNGAVMHPVAHTGV

100   RKMVDKNALSLWMRERSDLWVQPKVDGVAVTLVYRDGKLNKAISRGNGLK

150   GEDWTQKVSLISAVPQTVSGPLANSTLQGEIFLQREGHIQQQMGGINARA

200   KVAGLMMRQDDSDTLNSLGVFVWAWPDGPQLMSDRLKELATAGFTLTQTY

250   TRAVKNADEVARVRNEWWKAELPFVTDGVVVRAAKEPESRHWLPGQAEWL

300   VAWKYQPVAQVAEVKAIQFAVGKSGKISVVASLAPVMLDDKKVQRVNIGS
```

```
350   VRRWQEWDIAPGDQILVSLAGQGIPRIDDVVWRGAERTKPTPPENRFNSL

400   TCYFASDVCQEQFISRLVWLGAKQVLGLDGIGEAGWRALHQTHRFEHIFS

450   WLLLTPEQLQNTPGIAKSKSAQLWHQFNLARKQPFTRWVMAMGIPLTRAA

500   LNASDERSWSQLLFSTEQFWQQLPGTGSGRARQVIEWKENAQIKKLGSWL

550   AAQQITGFEP

sp|P00895|TRPE_ECOLI Anthranilate synthase component 1 OS=Escherichia
coli (strain K12) OX=83333 GN=trpE PE=1 SV=2
                                    ********************
0     MQTQKPTLELLTCEGAYRDNPTALFHQLCGDRPATLLLESADIDSKDDLK
      ***********
50    SLLLVDSALRITALGDTVTIQALSGNGEALLALLDNALPAGVESEQSPNC

100   RVLRFPPVSPLLDEDARLCSLSVFDAFRLLQNLLNVPKEEREAMFFGGLF

150   SYDLVAGFEDLPQLSAENNCPDFCFYLAETLMVIDHQKKSTRIQASLFAP

200   NEEEKQRLTARLNELRQQLTEAAPPLPVVSVPHMRCECNQSDEEFGGVVR

250   LLQKAIRAGEIFQVVPSRRFSLPCPSPLAAYYVLKKSNPSPYMFFMQDND
                                            ************
300   FTLFGASPESSLKYDATSRQIEIYPIAGTRPRGRRADGSLDRDLDSRIEL
      ************
350   EMRTDHKELSEHLMLVDLARNDLARICTPGSRYVADLTKVDRYSYVMHLV

400   SRVVGELRHDLDALHAYRACMNMGTLSGAPKVRAMQLIAEAEGRRRGSYG

450   GAVGYFTAHGDLDTCIVIRSALVENGIATVQAGAGVVLDSVPQSEADETR

500   NKARAVLRAIATAHHAQETF

sp|P23485|FECR_ECOLI Protein FecR OS=Escherichia coli (strain K12)
OX=83333 GN=fecR PE=2 SV=1

0     MNPLLTDSRRQALRSASHWYAVLSGERVSPQQEARWQQWYEQDQDNQWAW

50    QQVENLRNQLGGVPGDVASRALHDTRLTRRHVMKGLLLLLGAGGGWQLWQ

100   SETGEGLRADYRTAKGTVSRQQLEDGSLLTLNTQSAADVRFDAHQRTVRL

150   WYGEIAITTAKDALQRPFRVLTRQGQLTALGTEFTVRQQDNFTQLDVQQH
                         *******************************
200   AVEVLLASAPAQKRIVNAGESLQFSASEFGAVKPLDDESTSWTKDILSFS
      *******
250   DKPLGEVIATLTRYRNGVLRCDPAVAGLRLSGTFPLKNTDAILNVIAQTL

300   PVKIQSITRYWINISPL
```

sp|P0A8X0|YJGA_ECOLI UPF0307 protein YjgA OS=Escherichia coli (strain K12) OX=83333 GN=yjgA PE=1 SV=1

```
      ************************************************
0     MTKQPEDWLDDVPGDDIEDEDDEIIWVSKSEIKRDAEELKRLGAEIVDLG

50    KNALDKIPLDADLRAAIELAQRIKMEGRRRQLQLIGKMLRQRDVEPIRQA

100   LDKLKNRHNQQVVLFHKLENLRDRLIDQGDDAIAEVLNLWPDADRQQLRT

150   LIRNAKKEKEGNKPPKSARQIFQYLRELAENEG
```

sp|P15032|RECE_ECOLI Exodeoxyribonuclease 8 OS=Escherichia coli (strain K12) OX=83333 GN=recE PE=1 SV=3

```
                ********************** 
0     MSTKPLFLLRKAKKSSGEPDVVLWASNDFESTCATLDYLIVKSGKKLSSY

50    FKAVATNFPVVNDLPAEGEIDFTWSERYQLSKDSMTWELKPGAAPDNAHY

100   QGNTNVNGEDMTEIEENMLLPISGQELPIRWLAQHGSEKPVTHVSRDGLQ

150   ALHIARAEELPAVTALAVSHKTSLLDPLEIRELHKLVRDTDKVFPNPGNS

200   NLGLITAFFEAYLNADYTDRGLLTKEWMKGNRVSHITRTASGANAGGGNL

250   TDRGEGFVHDLTSLARDVATGVLARSMDLDIYNLHPAHAKRIEEIIAENK

300   PPFSVFRDKFITMPGGLDYSRAIVVASVKEAPIGIEVIPAHVTEYLNKVL
                         ******************************
350   TETDHANPDPEIVDIACGRSSAPMPQRVTEEGKQDDEEKPQPSGTTAVEQ

400   GEAETMEPDATEHHQDTQPLDAQSQVNSVDAKYQELRAELHEARKNIPSK

450   NPVDDDKLLAASRGEFVDGISDPNDPKWVKGIQTRDCVYQNQPETEKTSP
                                            **************
500   DMNQPEPVVQQEPEIACNACGQTGGDNCPDCGAVMGDATYQETFDEESQV
      ****************
550   EAKENDPEEMEGAEHPHNENAGSDPHRDCSDETGEVADPVIVEDIEPGIY

600   YGISNENYHAGPGISKSQLDDIADTPALYLWRKNAPVDTTKTKTLDLGTA

650   FHCRVLEPEEFSNRFIVAPEFNRRTNAGKEEEKAFLMECASTGKTVITAE

700   EGRKIELMYQSVMALPLGQWLVESAGHAESSIYWEDPETGILCRCRPDKI

750   IPEFHWIMDVKTTADIQRFKTAYYDYRYHVQDAFYSDGYEAQFGVQPTFV

800   FLVASTTIECGRYPVEIFMMGEEAKLAGQQEYHRNLRTLSDCLNTDEWPA

850   IKTLSLPRWAKEYAND
```

sp|P39352|YJHB_ECOLI Putative metabolite transport protein YjhB OS=Escherichia coli (strain K12) OX=83333 GN=yjhB PE=1 SV=2

```
0     MATAWYKQVNPPQRKALFSAWLGYVFDGFDFMMIFYILHIIKADLGITDI
```

```
50    QATLIGTVAFIARPIGGGFFGAMADKYGRKPMMMWAIFIYSVGTGLSGIA

100   TNLYMLAVCRFIVGLGMSGEYACASTYAVESWPKNLQSKASAFLVSGFSV
                                      ******************
150   GNIIAAQIIPQFAEVYGWRNSFFIGLLPVLLVLWIRKSAPESQEWIEDKY
      ****************
200   KDKSTFLSVFRKPHLSISMIVFLVCFCLFGANWPINGLLPSYLADNGVNT

250   VVISTLMTIAGLGTLTGTIFFGFVGDKIGVKKAFVVGLITSFIFLCPLFF

300   ISVKNSSLIGLCLFGLMFTNLGIAGLVPKFIYDYFPTKLRGLGTGLIYNL

350   GATGGMAAPVLATYISGYYGLGVSLFIVTVAFSALLILLVGFDIPGKIYK

400   LSVAK
```

sp|P24218|INTD_ECOLI Prophage integrase IntD OS=Escherichia coli (strain K12) OX=83333 GN=intD PE=3 SV=1

```
                            ********************
0     MSLFRRNEIWYASYSLPGGKRIKESLGTKDKRQAQELHDKRKAELWRVEK
           **************************************
50    LGDLPDVTFEEACLRWLEEKADKKSLDSDKSRIEFWLEHFEGIRLKDISE

100   AKIYSAVSRMHNRKTKEIWKQKVQAAIRKGKELPVYEPKPVSTQTKAKHL
                                        ****************
150   AMIKAILRAAERDWKWLEKAPVIKIPAVRNKRVRWLEKEEAKRLIDECPE
      *******
200   PLKSVVKFALATGLRKSNIINLEWQQIDMQRRVAWVNPEESKSNRAIGVA

250   LNDTACKVLRDQIGKHHKWVFVHTKAAKRADGTSTPAVRKMRIDSKTSWL

300   SACRRAGIEDFRFHDLRHTWASWLIQSGVPLSVLQEMGGWESIEMVRRYA

350   HLAPNHLTEHARKIDDIFGDNVPNMSHSEIMEDIKKA
```

sp|P11072|LIT_ECOLI Cell death peptidase OS=Escherichia coli (strain K12) OX=83333 GN=lit PE=3 SV=2

```
                   ******************************
0     MRSPICHLFSAINSSPFKIAPEKEQDLKTIVDDKKIIISVVSEPGFNIRV
                                             ***********
50    RKNESNNSHEIVLTVASLEYIWAFSNFFWVFTQEYSKSQKNNDEHFDLTG
      ************           ********************
100   KNRLKKSDELLKWARKNLQTTGCESWPKKCPKPEAYLQGSEDSQVASEIF

150   LCAIAWILHHEISHVVLQHPLVTTAFSTQEEREADSHATKWILGNLYESA

200   PELKKRALGIATAVLCIQSLEVENYFCLQNTHPAAYERIYSNISCYPVGN

250   EELIEALCTVMLQYLFHGKNINVNLDGESFSSILGDLLCDISRLTSN
```

sp|Q46798|YGER_ECOLI Uncharacterized lipoprotein YgeR OS=Escherichia coli (strain K12) OX=83333 GN=ygeR PE=3 SV=2

```
0     MSAGRLNKKSLGIVMLLSVGLLLAGCSGSKSSDTGTYSGSVYTVKRGDTL
                                    ********************
50    YRISRTTGTSVKELARLNGISPPYTIEVGQKLKLGGAKSSSITRKSTAKS
      *******
100   TTKTASVTPSSAVPKSSWPPVGQRCWLWPTTGKVIMPYSTADGGNKGIDI

150   SAPRGTPIYAAGAGKVVYVGNQLRGYGNLIMIKHSEDYITAYAHNDTMLV

200   NNGQSVKAGQKIATMGSTDAASVRLHFQIRYRATAIDPLRYLPPQGSKPK

250   C
```

sp|P16431|HYCE_ECOLI Formate hydrogenlyase subunit 5 OS=Escherichia coli (strain K12) OX=83333 GN=hycE PE=1 SV=1

```
0     MSEEKLGQHYLAALNEAFPGVVLDHAWQTKDQLTVTVKVNYLPEVVEFLY

50    YKQGGWLSVLFGNDERKLNGHYAVYYVLSMEKGTKCWITVRVEVDANKPE

100   YPSVTPRVPAAVWGEREVRDMYGLIPVGLPDERRLVLPDDWPDELYPLRK

150   DSMDYRQRPAPTTDAETYEFINELGDKKNNVVPIGPLHVTSDEPGHFRLF

200   VDGENIIDADYRLFYVHRGMEKLAETRMGYNEVTFLSDRVCGICGFAHST

250   AYTTSVENAMGIQVPERAQMIRAILLEVERLHSHLLNLGLACHFTGFDSG

300   FMQFFRVRETSMKMAEILTGARKTYGLNLIGGIRRDLLKDDMIQTRQLAQ

350   QMRREVQELVDVLLSTPNMEQRTVGIGRLDPEIARDFSNVGPMVRASGHA

400   RDTRADHPFVGYGLLPMEVHSEQGCDVISRLKVRINEVYTALNMIDYGLD

450   NLPGGPLMVEGFTYIPHRFALGFAEAPRGDDIHWSMTGDNQKLYRWRCRA
                                                **********
500   ATYANWPTLRYMLRGNTVSDAPLIIGSLDPCYSCTDRMTVVDVRKKKSKV
      ****************
550   VPYKELERYSIERKNSPLK
```

sp|P77188|ECPB_ECOLI Probable fimbrial chaperone EcpB OS=Escherichia coli (strain K12) OX=83333 GN=ecpB PE=1 SV=1

```
                         *******************************
0     MKKHLLPLALLFSGISPAQALDVGDISSFMNSDSSTLSKTIKNSTDSGRL
      *    ********************
50    INIRLERLSSPLDDGQVISMDKPDELLLTPASLLLPAQASEVIRFFYKGP

100   ADEKERYYRIVWFDQALSDAQRDNANRSAVATASARIGTILVVAPRQANY

150   HFQYANGSLTNTGNATLRILAYGPCLKAANGKECKENYYLMPGKSRRFTR

200   VDTADNKGRVALWQGDKFIPVK
```

sp|P37651|GUN_ECOLI Endoglucanase OS=Escherichia coli (strain K12) OX=83333 GN=bcsZ PE=1 SV=1

```
0     MNVLRSGIVTMLLLAAFSVQAACTWPAWEQFKKDYISQEGRVIDPSDARK
                                                 *****
50    ITTSEGQSYGMFSALAANDRAAFDNILDWTQNNLAQGSLKERLPAWLWGK
      *********************
100   KENSKWEVLDSNSASDGDVWMAWSLLEAGRLWKEQRYTDIGSALLKRIAR

150   EEVVTVPGLGSMLLPGKVGFAEDNSWRFNPSYLPPTLAQYFTRFGAPWTT
                             *********************
200   LRETNQRLLLETAPKGFSPDWVRYEKDKGWQLKAEKTLISSYDAIRVYMW

250   VGMMPDSDPQKARMLNRFKPMATFTEKNGYPPEKVDVATGKAQGKGPVGF

300   SAAMLPFLQNRDAQAVQRQRVADNFPGSDAYYNYVLTLFGQGWDQHRFRF

350   STKGELLPDWGQECANSH
```

sp|P76613|YPJC_ECOLI Protein YpjC OS=Escherichia coli (strain K12) OX=83333 GN=ypjC PE=4 SV=1

```
0     MLVSKSNGFNASAVLGSGSYNENKSSKHMELLAHSILKLICKEAASETYR

50    GALETLQKMMSECIYQEGNAFVIMGAGEQLKRIKYEVGENNLKVFNVHFN
                                ********************
100   NNHELVSSGEPDVICLSKQVWENLLIKLKLENNENVFSETKKLSNKNNAD

150   QFFECAKRNE
```

sp|P37018|YADM_ECOLI Uncharacterized fimbrial-like protein YadM OS=Escherichia coli (strain K12) OX=83333 GN=yadM PE=2 SV=1

```
0     MIKTTPHKIVILMGILLSPSVFATDINVEFTATVKATTCNITLTGNNVTN
                                                      **
50    DGNNNYTLRIPKMGLDKIANKTTESQADFKLVASGCSSGISWIDTTLTGN
      **********************
100   ASSSSPKLIIPQSGDSSSTTSNIGMGFKKRTTDDATFLKPNSAEKIRWST

150   DEMQPDKGLEMTVALRETDAGQGVPGNFRALATFNFIYQ
```

sp|P42641|OBG_ECOLI GTPase ObgE/CgtA OS=Escherichia coli (strain K12) OX=83333 GN=obgE PE=1 SV=1

```
0     MKFVDEASILVVAGDGGNGCVSFRREKYIPKGGPDGGDGGDGGDVWMEAD

50    ENLNTLIDYRFEKSFRAERGQNGASRDCTGKRGKDVTIKVPVGTRVIDQG

100   TGETMGDMTKHGQRLLVAKGGWHGLGNTRFKSSVNRTPRQKTNGTPGDKR

150   ELLLELMLLADVGMLGMPNAGKSTFIRAVSAAKPKVADYPFTTLVPSLGV

200   VRMDNEKSFVVADIPGLIEGAAEGAGLGIRFLKHLERCRVLLHLIDIDPI
                                      ******************
250   DGTDPVENARIIISELEKYSQDLATKPRWLVFNKIDLLLDKVEAEEKAKAI
      *********
```

```
300   AEALGWEDKYYLISAASGLGVKDLCWDVMTFIIENPVVQAEEAKQPEKVE
                          *********************
350   FMWDDYHRQQLEEIAEEDDEDWDDDWDEDDEEGVEFIYKR
```

sp|P76464|A2MGH_ECOLI Putative alpha-2-macroglobulin homolog OS=Escherichia coli (strain K12) OX=83333 GN=yfaS PE=5 SV=3

```
0     MDTQRFQSQFHWHLSFKFSGAIAACLSLSLVGTGLANADDSLPSSNYAPP
          **************************
50    AGGTFFLLADSSFSSSEEAKVRLEAPGRDYRRYQMEEYGGVDVRLYRIPD

100   PMAFLRQQKNLHRIVVQPQYLGDGLNNTLTWLWDNWYGKSRRVMQRTFSS

150   QSRQNVTQALPELQLGNAIIKPSRYVQNNQFSPLKKYPLVKQFRYPLWQA

200   KPFEPQQGVKLEGASSNFISPQPGNIYIPLGQQEPGLYLVEAMVGGYRAT

250   TVVFVSDTVALSKVSGKELLVWTAGKKQGEAKPGSEILWTDGLGVMTRGV

300   TDDSGTLQLQHISPERSYILGKDAEGGVFVSENFFYESEIYNTRLYIFTD

350   RPLYRAGDRVDVKVIGREFHDPLHSSPIVSAPAKLSVLDANGSLLQTVNV

400   TLDARNGGQGSFRLPENAVAGGYELRLAYRNQVYSSSFRVANYIKPHFEI

450   GLALAKKEFKTGEAVSGKLQLLYPDGEPVKNARVQLSLRAQQLSMVGNDL

500   RYAGRFPVSLEGSETVSDASGHVALNLPAADKPSRYLLTVSASDGAAYRV

550   TTTKEILIERGLAHYSLSTAAQYSNSGESVVFRYAALESSKQVPVTYEWL

600   RLEDRTSHSGELPSGGKSFTVNFAKPGNYNLTLRDKDGLILAGLSHAVSG

650   KGSTAHTGTVDIVADKTLYQPGETAKMLITFPEPIDEALLTLERDRVEQQ

700   SLLSHPANWLTLQRLNDTQYEARVPVSNSFAPNITFSVLYTRNGQYSFQN

750   AGIKVAVPQLDIRVKTDKTHYQPGELVNVELTSSLKGKPVSAQLTVGVVD

800   EMIYALQPEIAPNIGKFFYPLGRNNVRTSSSLSFISYDQALSSEPVAPGA

850   TNRSERRVKMLERPRREEVDTAAWMPSLTTDKQGKAYFTFLMPDSLTRWR

900   ITARGMNGDGLVGQGRAYLRSEKNLYMKWSMPTVYRVGDKPAAGLFIFSQ

950   QDNEPVALVTKFAGAEMRQTLTLHKGANYISLTQNIQQSGLLSAELQQNG

1000  QVQDSISTKLSFVDNSWPVEQQKNVMLGGGDNALMLPEQASNIRLQSSET

1050  PQEIFRNNLDALVDEPWGGVINTGSRLIPLSLAWRSLADHQSAAANDIRQ

1100  MIQDNRLRLMQLAGPGARFTWWGEDGNGDAFLTAWAWYADWQASQAIGVT

1150  QQPEYWQHMLDSYAEQADNMPLLHRALVLAWAQEMNLPCKTLLKGLDEAI
```

```
       ************************
1200  ARRGTKTEDFSEEDTRDINDSLILDTPESPLADAVANVLTMTLLKKAQLK

1250  STVMPQVQQYAWDKAANSNQPLAHTVVLLNSGGDATQTAAILSGLTAEQS

1300  TIERALAMNWLAKYMATMPPVVLPAPAGAWAKHKLTGGGEDWRWVGQGVP

1350  DILSFGDELSPQNVQVR
```

sp|P77376|YDGJ_ECOLI Uncharacterized oxidoreductase YdgJ OS=Escherichia coli (strain K12) OX=83333 GN=ydgJ PE=1 SV=2

```
                                         ***************
0     MSDNIRVGLIGYGYASKTFHAPLIAGTPGQELAVISSSDETKVKADWPTV
      *****
50    TVVSEPKHLFNDPNIDLIVIPTPNDTHFPLAKAALEAGKHVVVDKPFTVT

100   LSQARELDALAKSLGRVLSVFHNRRWDSDFLTLKGLLAEGVLGEVAYFES

150   HFDRFRPQVRDRWREQGGPGSGIWYDLAPHLLDQAITLFGLPVSMTVDLA

200   QLRPGAQSTDYFHAILSYPQRRVILHGTMLAAAESARYIVHGSRGSYVKY

250   GLDPQEERLKNGERLPQEDWGYDMRDGVLTRVEGEERVEETLLTVPGNYP

300   AYYAAIRDALNGDGENPVPASQAIQVMELIELGIESAKHRATLCLA
```

sp|P29009|YDFB_ECOLI Uncharacterized protein YdfB OS=Escherichia coli (strain K12) OX=83333 GN=ydfB PE=1 SV=1

```
                 *******************************
0     MDFDTIMEKAYEEYFEGLAEGEEALSFSEFKQALSSSAKSNG
```

sp|P0A6P9|ENO_ECOLI Enolase OS=Escherichia coli (strain K12) OX=83333 GN=eno PE=1 SV=2

```
0     MSKIVKIIGREIIDSRGNPTVEAEVHLEGGFVGMAAAPSGASTGSREALE

50    LRDGDKSRFLGKGVTKAVAAVNGPIAQALIGKDAKDQAGIDKIMIDLDGT

100   ENKSKFGANAILAVSLANAKAAAAAKGMPLYEHIAELNGTPGKYSMPVPM

150   MNIINGGEHADNNVDIQEFMIQPVGAKTVKEAIRMGSEVFHHLAKVLKAK

200   GMNTAVGDEGGYAPNLGSNAEALAVIAEAVKAAGYELGKDITLAMDCAAS
                                 ********************
250   EFYKDGKYVLAGEGNKAFTSEEFTHFLEELTKQYPIVSIEDGLDESDWDG

300   FAYQTKVLGDKIQLVGDDLFVTNTKILKEGIEKGIANSILIKFNQIGSLT

350   ETLAAIKMAKDAGYTAVISHRSGETEDATIADLAVGTAAGQIKTGSMSRS

400   DRVAKYNQLIRIEEALGEKAPYNGRKEIKGQA
```

sp|P76481|YFBK_ECOLI Uncharacterized protein YfbK OS=Escherichia coli (strain K12) OX=83333 GN=yfbK PE=4 SV=1

```
0     MRNKNIIMLLMSSLILSGCGPQPENKESQQQQPSTPTEQQVLAAQQAAIK

50    EAEQSAAAAKALAQQEVQQYSDKQALQGRLQEAPTFARAAKAKATHIANP

100   GTARYQQFDDNPVKQVAQNPLATFSLDVDTGSYANVRRFLNQGLLPPPDA

150   VRVEEIVNYFPSDWDIKDKQSIPASKPIPFAMRYELAPAPWNEQRTLLKV

200   DILAKDRKSEELPASNLVFLIDTSGSMISDERLPLIQSSLKLLVKELREQ

250   DNIAIVTYAGDSRIALPSISGSHKAEINAAIDSLDAEGSTNGGAGLELAY
                         *****************************
300   QQATKGFIKGGINRILLATDGDFNVGIDDPKSIESMVKKQRESGVTLSTF

350   GVGNSNYNEAMMVRIADVGNGNYSYIDTLSEAQKVLNSEMRQMLITVAKD

400   VKAQIEFNPAWVTEYRQIGYEKRQLRVEHFNNDNVDAGDIGAGKHITLLF
             ************************
450   ELTLNGQKASIDKLRYAPDNKLAKSDKTKELAWLKIRWKYPQGKESQLVE

500   FPLGPTINAPSEDMRFRAAVAAYGQKLRGSEYLNNTSWQQIKQWAQQAKG

550   EDPQGYRAEFIRLIELADGVTDISQ
```

sp|P31473|RAVA_ECOLI ATPase RavA OS=Escherichia coli (strain K12) OX=83333 GN=ravA PE=1 SV=2

```
0     MAHPHLLAERISRLSSSLEKGLYERSHAIRLCLLAALSGESVFLLGPPGI

50    AKSLIARRLKFAFQNARAFEYLMTRFSTPEEVFGPLSIQALKDEGRYERL

100   TSGYLPEAEIVFLDEIWKAGPAILNTLLTAINERQFRNGAHVEKIPMRLL

150   VAASNELPEADSSLEALYDRMLIRLWLDKVQDKANFRSMLTSQQDENDNP

200   VPDALQVTDEEYERWQKEIGEITLPDHVFELIFMLRQQLDKLPDAPYVSD

250   RRWKKAIRLLQASAFFSGRSAVAPVDLILLKDCLWYDAQSLNLIQQQIDV

300   LMTGHAWQQQGMLTRLGAIVQRHLQLQQQSDKTALTVIRLGGIFSRRQQ

350   YQLPVNVTASTLTLLLQKPLKLHDMEVVHISFERSALEQWLSKGGEIRGK
                                            **************
400   LNGIGFAQKLNLEVDSAQHLVVRDVSLQGSTLALPGSSAEGLPGEIKQQL
      ******
450   EELESDWRKQHALFSEQQKCLFIPGDWLGRIEASLQDVGAQIRQAQQC
```

sp|P0CK95|ACFD_ECOLI Putative lipoprotein AcfD homolog OS=Escherichia coli (strain K12) OX=83333 GN=yghJ PE=3 SV=1

```
0     MNKKFKYKKSLLAAILSATLLAGCDGGGSGSSSDTPPVDSGTGSLPEVKP

50    DPTPNPEPTPEPTPDPEPTPEPIPDPEPTPEPEPEPVPTKTGYLTLGGSQ
```

```
                                                        ****
100   RVTGATCNGESSDGFTFKPGEDVTCVAGNTTIATFNTQSEAARSLRAVEK
      *********************************             ****
150   VSFSLEDAQELAGSDDKKSNAVSLVTSSNSCPANTEQVCLTFSSVIESKR
      *****************
200   FDSLYKQIDLAPEEFKKLVNEEVENNAATDKAPSTHTSPVVPVTTPGTKP

250   DLNASFVSANAEQFYQYQPTEIILSEGRLVDSQGYGVAGVNYYTNSGRGV

300   TGENGEFSFSWGETISFGIDTFELGSVRGNKSTIALTELGDEVRGANIDQ

350   LIHRYSTTGQNNTRVVPDDVRKVFAEYPNVINEIINLSLSNGATLGEGEQ

400   VVNLPNEFIEQFNTGQAKEIDTAICAKTDGCNEARWFSLTTRNVNDGQIQ

450   GVINKLWGVDTNYKSVSKFHVFHDSTNFYGSTGNARGQAVVNISNAAFPI

500   LMARNDKNYWLAFGEKRAWDKNELAYITEAPSLVEPENVTRDTATFNLPF

550   ISLGQVGEGKLMVIGNPHYNSILRCPNGYSWNGGVNKDGQCTLNSDPDDM

600   KNFMENVLRYLSDDKWKPDAKASMTVGTNLDTVYFKRHGQVTGNSAAFDF

650   HPDFAGISVEHLSSYGDLDPQEMPLLILNGFEYVTQVGNDPYAIPLRADT

700   SKPKLTQQDVTDLIAYLNKGGSVLIMENVMSNLKEESASGFVRLLDAAGL
                                                      ******
750   SMALNKSVVNNDPQGYPNRVRQQRATGIWVYERYPAVDGALPYTIDSKTG
      ********************************* ****************
800   EVKWKYQVENKPDDKPKLEVASWLEDVDGKQETRYAFIDEADHKTEDSLK
      *********
850   AAKEKIFAAFPGLKECTNPAYHYEVNCLEYRPGTGVPVTGGMYVPQYTQL

900   SLNADTAKAMVQAADLGTNIQRLYQHELYFRTNGRKGERLSSVDLERLYQ

950   NMSVWLWNDTSYRYEEGKNDELGFKTFTEFLNCYANDAYAGGTKCSADLK

1000  KSLVDNNMIYGDGSSKAGMMNPSYPLNYMEKPLTRLMLGRSWWDLNIKVD

1050  VEKYPGAVSEEGQNVTETISLYSNPTKWFAGNMQSTGLWAPAQKEVTIKS

1100  NANVPVTVTVALADDLTGREKHEVALNRPPRVTKTYSLDASGTVKFKVPY

1150  GGLIYIKGNSSTNESASFTFTGVVKAPFYKDGAWKNDLNSPAPLGELESD

1200  AFVYTTPKKNLNASNYTGGLEQFANDLDTFASSMNDFYGRDSEDGKHRMF

1250  TYKNLPGHKHRFTNDVQISIGDAHSGYPVMNSSFSPNSTTLPTTPLNDWL

1300  IWHEVGHNAAETPLTVPGATEVANNVLALYMQDRYLGKMNRVADDITVAP

1350  EYLEESNNQAWARGGAGDRLLMYAQLKEWAEKNFDIKKWYPDGTPLPEFY

1400  SEREGMKGWNLFQLMHRKARGDEVSNDKFGGKNYCAESNGNAADTLMLCA
```

1450 SWVAQTDLSEFFKKWNPGANAYQLPGASEMSFEGGVSQSAYNTLASLDLP

1500 KPEQGPETINQVTEHKMSAE

sp|P52125|YFJJ_ECOLI Uncharacterized protein YfjJ OS=Escherichia coli (strain K12) OX=83333 GN=yfjJ PE=4 SV=1

0 MTYDSEFGSHVSLYRDRIKQVIDDSLNEHLNSMILRVDLHDPIDTENMDN
****
50 PFFQPRVDSGAISRFTSALKAKLKHDKHIKTQRKDWPDSRHSTLRYAWVR

100 EYTKNRKRHYHLILCFNQDAYYHLGDYDLNRNTLRTMITTAWYSALGIPI

150 DSSGKLVNYPPNGKYLLNRKRDNFEQTYSDLMNRVDYMTKVRTKIVGDGD

200 RNFGCSRG

sp|P0ADQ7|YGAM_ECOLI Uncharacterized protein YgaM OS=Escherichia coli (strain K12) OX=83333 GN=ygaM PE=1 SV=2
****
0 MFNRPNRNDVDDGVQDIQNDVNQLADSLESVLKSWGSDAKGEAEAARSKA

50 QALLKETRARMHGRTRVQQAARDAVGCADSFVRERPWCSVGTAAAVGIFI

100 GALLSMRKS

sp|P0A8N3|SYK1_ECOLI Lysine--tRNA ligase OS=Escherichia coli (strain K12) OX=83333 GN=lysS PE=1 SV=2
****
0 MSEQHAQGADAVVDLNNELKTRREKLANLREQGIAFPNDFRRDHTSDQLH
****
50 AEFDGKENEELEALNIEVAVAGRMMTRRIMGKASFVTLQDVGGRIQLYVA

100 RDDLPEGVYNEQFKKWDLGDILGAKGKLFKTKTGELSIHCTELRLLTKAL

150 RPLPDKFHGLQDQEARYRQRYLDLISNDESRNTFKVRSQILSGIRQFMVN

200 RGFMEVETPMMQVIPGGAAARPFITHHNALDLDMYLRIAPELYLKRLVVG

250 GFERVFEINRNFRNEGISVRHNPEFTMMELYMAYADYKDLIELTESLFRT

300 LAQDILGKTEVTYGDVTLDFGKPFEKLTMREAIKKYRPETDMADLDNFDS

350 AKAIAESIGIHVEKSWGLGRIVTEIFEEVAEAHLIQPTFITEYPAEVSPL

400 ARRNDVNPEITDRFEFFIGGREIGNGFSELNDAEDQAQRFLDQVAAKDAG

450 DDEAMFYDEDYVTALEHGLPPTAGLGIGIDRMVMLFTNSHTIRDVILFPA

500 MRPVK

sp|P77718|THII_ECOLI tRNA sulfurtransferase OS=Escherichia coli (strain K12) OX=83333 GN=thiI PE=1 SV=1

```
0     MKFIIKLFPEITIKSQSVRLRFIKILTGNIRNVLKHYDETLAVVRHWDNI

50    EVRAKDENQRLAIRDALTRIPGIHHILEVEDVPFTDMHDIFEKALVQYRD

100   QLEGKTFCVRVKRRGKHDFSSIDVERYVGGGLNQHIESARVKLTNPDVTV

150   HLEVEDDRLLLIKGRYEGIGGFPIGTQEDVLSLISGGFDSGVSSYMLMRR

200   GCRVHYCFFNLGGAAHEIGVRQVAHYLWNRFGSSHRVRFVAINFEPVVGE

250   ILEKIDDGQMGVILKRMMVRAASKVAERYGVQALVTGEALGQVSSQTLTN
                                                     ******
300   LRLIDNVSDTLILRPLISYDKEHIINLARQIGTEDFARTMPEYCGVISKS
      *********************************
350   PTVKAVKSKIEAEEEKFDFSILDKVVEEANNVDIREIAQQTEQEVVEVET
             ***************************
400   VNGFGPNDVILDIRSIDEQEDKPLKVEGIDVVSLPFYKLSTKFGDLDQNK

450   TWLLWCERGVMSRLQALYLREQGFNNVKVYRP
```

sp|P0ACE0|MBHM_ECOLI Hydrogenase-2 large chain OS=Escherichia coli (strain K12) OX=83333 GN=hybC PE=1 SV=2

```
0     MSQRITIDPVTRIEGHLRIDCEIENGVVSKAWASGTMWRGMEEIVKNRDP

50    RDAWMIVQRICGVCTTTHALSSVRAAESALNIDVPVNAQYIRNIILAAHT

100   THDHIVHFYQLSALDWVDITSALQADPTKASEMLKGVSTWHLNSPEEFTK

150   VQNKIKDLVASGQLGIFANGYWGHPAMKLPPEVNLIAVAHYLQALECQRD

200   ANRVVALLGGKTPHIQNLAVGGVANPINLDGLGVLNLERLMYIKSFIDKL

250   SDFVEQVYKVDTAVIAAFYPEWLTRGKGAVNYLSVPEFPTDSKNGSFLFP
                        *********************
300   GGYIENADLSSYRPITSHSDEYLIKGIQESAKHSWYKDEAPQAPWEGTTI

350   PAYDGWSDDGKYSWVKSPTFYGKTVEVGPLANMLVKLAAGRESTQNKLNE

400   IVAIYQKLTGNTLEVAQLHSTLGRIIGRTVHCCELQDILQNQYSALITNI

450   GKGDHTTFVKPNIPATGEFKGVGFLEAPRGMLSHWMVIKDGIISNYQAVV

500   PSTWNSGPRNFNDDVGPYEQSLVGTPVADPNKPLEVVRTIHSFDPCMACA

550   VHVVDADGNEVVSVKVL
```

sp|P37683|YIAV_ECOLI Inner membrane protein YiaV OS=Escherichia coli (strain K12) OX=83333 GN=yiaV PE=1 SV=1

```
0     MDLLIILTYVAFAWAMFKIFKIPVNKWTIPTAALGGIFIVSGLILLMNYN

50    HPYTFKAQKAVISIPVVPQVTGVVIEVTDKKNTLIKKGEVLFRLDPTRYQ
```

100   ARVDRLMADIVTAEHKQRALGAELDEMAANTQQAKATRDKFAKEYQRYAR
                              ****

150   GSQAKVNPFSERDIDVARQNYLAQEASVKSSAAEQKQIQSQLDSLVLGEH

200   SQIASLKAQLAEAKYNLEQTIVRAPSDGYVTQVLIRPGTYAASLPLRPVM

250   VFIPDQKRQIVAQFRQNSLLRLAPGDDAEVVFNALPGKVFSGKLAAISPA

300   VPGGAYQSTGTLQTLNTAPGSDGVIATIELDEHTDLSALPDGIYAQVAVY

350   SDHFSHVSVMRKVLLRMTSWVHYLYLDH

sp|P77245|MURR_ECOLI HTH-type transcriptional regulator MurR OS=Escherichia coli (strain K12) OX=83333 GN=murR PE=1 SV=1

0   MLYLTKISNAGSEFTENEQKIADFLQANVSELQSVSSRQMAKQLGISQSS
                                             ****

50   IVKFAQKLGAQGFTELRMALIGEYSASREKTNATALHLHSSITSDDSLEV
      ****

100   IARKLNREKELALEQTCALLDYARLQKIIEVISKAPFIQITGLGGSALVG

150   RDLSFKLMKIGYRVACEADTHVQATVSQALKKGDVQIAISYSGSKKEIVL

200   CAEAARKQGATVIAITSLTDSPLRRLAHFTLDTVSGETEWRSSSMSTRTA

250   QNSVTDLLFVGLVQLNDVESLKMIQRSSELTQRLK

sp|P0A8M3|SYT_ECOLI Threonine--tRNA ligase OS=Escherichia coli (strain K12) OX=83333 GN=thrS PE=1 SV=1

0   MPVITLPDGSQRHYDHAVSPMDVALDIGPGLAKACIAGRVNGELVDACDL

50   IENDAQLSIITAKDEEGLEIIRHSCAHLLGHAIKQLWPHTKMAIGPVIDN
                          ****

100   GFYYDVDLDRTLTQEDVEALEKRMHELAEKNYDVIKKKVSWHEARETFAN

150   RGESYKVSILDENIAHDDKPGLYFHEEYVDMCRGPHVPNMRFCHHFKLMK

200   TAGAYWRGDSNNKMLQRIYGTAWADKKALNAYLQRLEEAAKRDHRKIGKQ

250   LDLYHMQEEAPGMVFWHNDGWTIFRELEVFVRSKLKEYQYQEVKGPFMMD

300   RVLWEKTGHWDNYKDAMFTTSSENREYCIKPMNCPGHVQIFNQGLKSYRD

350   LPLRMAEFGSCHRNEPSGSLHGLMRVRGFTQDDAHIFCTEEQIRDEVNGC

400   IRLVYDMYSTFGFEKIVVKLSTRPEKRIGSDEMWDRAEADLAVALEENNI

450   PFEYQLGEGAFYGPKIEFTLYDCLDRAWQCGTVQLDFSLPSRLSASYVGE

500   DNERKVPVMIHRAILGSMERFIGILTEEFAGFFPTWLAPVQVVIMNITDS

550   QSEYVNELTQKLSNAGIRVKADLRNEKIGFKIREHTLRRVPYMLVCGDKE

600   VESGKVAVRTRRGKDLGSMDVNEVIEKLQQEIRSRSLKQLEE

sp|P21362|YCIF_ECOLI Protein YciF OS=Escherichia coli (strain K12) OX=83333 GN=yciF PE=1 SV=1

0   MNMKTIEDVFIHLLSDTYSAEKQLTRALAKLARATSNEKLSQAFHAHLEE

50   THGQIERIDQVVESESNLKIKRMKCVAMEGLIEEANEVIESTEKNEVRDA
                                   ****
100   ALIAAAQKVEHYEIASYGTLATLAEQLGYRKAAKLLKETLEEEKATDIKL
      ***
150   TDLAINNVNKKAENKA

sp|Q59385|COPA_ECOLI Copper-exporting P-type ATPase OS=Escherichia coli (strain K12) OX=83333 GN=copA PE=1 SV=4

0   MSQTIDLTLDGLSCGHCVKRVKESLEQRPDVEQADVSITEAHVTGTASAE
                 ****
50   QLIETIKQAGYDASVSHPKAKPLAESSIPSEALTAVSEALPAATADDDDS

100   QQLLLSGMSCASCVTRVQNALQSVPGVTQARVNLAERTALVMGSASPQDL

150   VQAVEKAGYGAEAIEDDAKRRERQQETAVATMKRFRWQAIVALAVGIPVM

200   VWGMIGDNMMVTADNRSLWLVIGLITLAVMVFAGGHFYRSAWKSLLNGAA

250   TMDTLVALGTGVAWLYSMSVNLWPQWFPMEARHLYYEASAMIIGLINLGH

300   MLEARARQRSSKALEKLLDLTPPTARLVTDEGEKSVPLAEVQPGMLLRLT

350   TGDRVPVDGEITQGEAWLDEAMLTGEPIPQQKGEGDSVHAGTVVQDGSVL

400   FRASAVGSHTTLSRIIRMVRQAQSSKPEIGQLADKISAVFVPVVVVIALV

450   SAAIWYFFGPAPQIVYTLVIATTVLIIACPCALGLATPMSIISGVGRAAE

500   FGVLVRDADALQRASTLDTVVFDKTGTLTEGKPQVVAVKTFADVDEAQAL

550   RLAAALEQGSSHPLARAILDKAGDMQLPQVNGFRTLRGLGVSGEAEGHAL

600   LLGNQALLNEQQVGTKAIEAEITAQASQGATPVLLAVDGKAVALLAVRDP

650   LRSDSVAALQRLHKAGYRLVMLTGDNPTTANAIAKEAGIDEVIAGVLPDG

700   KAEAIKHLQSEGRQVAMVGDGINDAPALAQADVGIAMGGGSDVAIETAAI

750   TLMRHSLMGVADALAISRATLHNMKQNLLGAFIYNSIGIPVAAGILWPFT

800   GTLLNPVVAGAAMALSSITVVSNANRLLRFKPKE

sp|P77206|YAFZ_ECOLI UPF0380 protein YafZ OS=Escherichia coli (strain K12) OX=83333 GN=yafZ PE=3 SV=2
                            ****

0   MTRLASRFGAANLIRRDRPLTREELFRVVPSVFSEDKHESRSERYTYIPT

50   ISLLDSLQREGFQPFFACQTRVRDPGRREHTKHMLRLRREGQITGKQVPE

100   IILLNSHDGTSSYQMLPGLFRAVCQNGLVCGESFGEVRVPHKGDVVSQVI

150   EGAYEVLGIFDRVEEKRDAMQSLLLPPPAQQALAKAALTYRFGEDHQPVT

200   ESQILSPRRWQDESNDLWTTYQRIQENLIKGGLSGRNAKGGRSHTRAVRG

250   IDGDVKLNRALWVMAEALLTQLQ

sp|P0AB46|YMGD_ECOLI Uncharacterized protein YmgD OS=Escherichia coli (strain K12) OX=83333 GN=ymgD PE=1 SV=2

```
0     MKKFALLAGLFVFAPMTWAQDYNIKNGLPSETYITCAEANEMAKTDSAQV
                  *********************
50    AEIVAVMGNASVASRDLKIEQSPELSAKVVEKLNQVCAKDPQMLLITAID

100   DTMRAIGKK
```

sp|P75694|YAHO_ECOLI Uncharacterized protein YahO OS=Escherichia coli (strain K12) OX=83333 GN=yahO PE=3 SV=1

```
                            ****************************
0     MKIISKMLVGALALAVTNVYAAELMTKAEFEKVESQYEKIGDISTSNEMS
      ************************
50    TADAKEDLIKKADEKGADVLVLTSGQTDNKIHGTANIYKKK
```

sp|P37146|RIR4_ECOLI Ribonucleoside-diphosphate reductase 2 subunit beta OS=Escherichia coli (strain K12) OX=83333 GN=nrdF PE=1 SV=2

```
       ********************
0     MKLSRISAINWNKISDDKDLEVWNRLTSNFWLPEKVPLSNDIPAWQTLTV

50    VEQQLTMRVFTGLTLLDTLQNVIGAPSLMPDALTPHEEAVLSNISFMEAV

100   HARSYSSIFSTLCQTKDVDAAYAWSEENAPLQRKAQIIQQHYRGDDPLKK

150   KIASVFLESFLFYSGFWLPMYFSSRGKLTNTADLIRLIIRDEAVHGYYIG

200   YKYQKNMEKISLGQREELKSFAFDLLLELYDNELQYTDELYAETPWADDV
                                                    ****
250   KAFLCYNANKALMNLGYEPLFPAEMAEVNPAILAALSPNADENHDFFSGS
      *****************
300   GSSYVMGKAVETEDEDWNF
```

sp|P52138|YFJW_ECOLI Uncharacterized protein YfjW OS=Escherichia coli (strain K12) OX=83333 GN=yfjW PE=4 SV=2

```
0     MAFVSEREIVRKIFSKKIDFTILAFFYISSIFFLLCSGVLFQYFTAAFTK

50    GNCYECSMKLDYIKQFYFSLETAWYLISAVAVFIASVFIQHRIKAYLTLL

100   AITWIVLTITDVALIHALDNIAMNNILLNILYNLFGAILLSLFMCLSNSL
                                                   *****
```

```
150   LFHLNKIKHIPMILSAMIPLVSAIIIAILITAVIYLLFARQAVEIEMDIS
      *****************
200   EGSDIAYVGVKDNEESFGFLNDKKTDTPTYLDVIKNGSLIYNDTQGLSGA
                                                  ****
250   DIYIVSGCYALPDLLRNVPLDAKKSFLNVKKLEITQKLPMMGFIQGESAD
      **********************************
300   VMPKAASRLSLSKQDDKFMLASSVTDSQIKFKSNNAQLMVAFAFMPITTN

350   GILHDYTYDIIINDKKYKIENHVAPLSRLDKNKKMKCEYQQISDLTNTYN
                                          **************
400   INANYLTGFLLVLKPDDIINYNNSPSVLLKTDFAFYKKTYQKLDKIYDDI
      *******                    ********************
450   SNGKLSSLRATGISQFSINGKHLSLRPESEIIISEGSLYGLVNKSKKIKI

500   YGTADLVFVDNKIMNLRKITYLQSKLEIFGSSIMDILKYIFGLGLLAISI

550   KFIHSYFKNDVNENLFL
```

sp|P77398|ARNA_ECOLI Bifunctional polymyxin resistance protein ArnA OS=Escherichia coli (strain K12) OX=83333 GN=arnA PE=1 SV=1

```
0     MKTVVFAYHDMGCLGIEALLAAGYEISAIFTHTDNPGEKAFYGSVARLAA

50    ERGIPVYAPDNVNHPLWVERIAQLSPDVIFSFYYRHLIYDEILQLAPAGA

100   FNLHGSLLPKYRGRAPLNWVLVNGETETGVTLHRMVKRADAGAIVAQLRI

150   AIAPDDIAITLHHKLCHAARQLLEQTLPAIKHGNILEIAQRENEATCFGR

200   RTPDDSFLEWHKPASVLHNMVRAVADPWPGAFSYVGNQKFTVWSSRVHPH

250   ASKAQPGSVISVAPLLIACGDGALEIVTGQAGDGITMQGSQLAQTLGLVQ

300   GSRLNSQPACTARRRTRVLILGVNGFIGNHLTERLLREDHYEVYGLDIGS

350   DAISRFLNHPHFHFVEGDISIHSEWIEYHVKKCDVVLPLVAIATPIEYTR
                                   *********************
400   NPLRVFELDFEENLRIIRYCVKYRKRIIFPSTSEVYGMCSDKYFDEDHSN

450   LIVGPVNKPRWIYSVSKQLLDRVIWAYGEKEGLQFTLFRPFNWMGPRLDN

500   LNAARIGSSRAITQLILNLVEGSPIKLIDGGKQKRCFTDIRDGIEALYRI

550   IENAGNRCDGEIINIGNPENEASIEELGEMLLASFEKHPLRHHFPPFAGF

600   RVVESSSYYGKGYQDVEHRKPSIRNAHRCLDWEPKIDMQETIDETLDFFL

650   RTVDLTDKPS
```

sp|P39325|YTFQ_ECOLI ABC transporter periplasmic-binding protein YtfQ OS=Escherichia coli (strain K12) OX=83333 GN=ytfQ PE=1 SV=1

```
0     MWKRLLIVSAVSAAMSSMALAAPLTVGFSQVGSESGWRAAETNVAKSEAE
```

50   KRGITLKIADGQQKQENQIKAVRSFVAQGVDAIFIAPVVATGWEPVLKEA
      ****
100   KDAEIPVFLLDRSIDVKDKSLYMTTVTADNILEGKLIGDWLVKEVNGKPC
                                         ****
150   NVVELQGTVGASVAIDRKKGFAEAIKNAPNIKIIRSQSGDFTRSKGKEVM
      ****
200   ESFIKAENNGKNICMVYAHNDDMVIGAIQAIKEAGLKPGKDILTGSIDGV
                                                  ******
250   PDIYKAMMDGEANASVELTPNMAGPAFDALEKYKKDGTMPEKLTLTKSTL
      ****
300   YLPDTAKEELEKKKNMGY

sp|P42592|YGJK_ECOLI Glucosidase YgjK OS=Escherichia coli (strain K12)
OX=83333 GN=ygjK PE=1 SV=1

0   MKIKTILTPVTCALLISFSAHAANADNYKNVINRTGAPQYMKDYDYDDHQ

50   RFNPFFDLGAWHGHLLPDGPNTMGGFPGVALLTEEYINFMASNFDRLTVW

100   QDGKKVDFTLEAYSIPGALVQKLTAKDVQVEMTLRFATPRTSLLETKITS
            ****
150   NKPLDLVWDGELLEKLEAKEGKPLSDKTIAGEYPDYQRKISATRDGLKVT

200   FGKVRATWDLLTSGESEYQVHKSLPVQTEINGNRFTSKAHINGSTTLYTT

250   YSHLLTAQEVSKEQMQIRDILARPAFYLTASQQRWEEYLKKGLTNPDATP

300   EQTRVAVKAIETLNGNWRSPGGAVKFNTVTPSVTGRWFSGNQTWPWDTWK

350   QAFAMAHFNPDIAKENIRAVFSWQIQPGDSVRPQDVGFVPDLIAWNLSPE

400   RGGDGGNWNERNTKPSLAAWSVMEVYNVTQDKTWVAEMYPKLVAYHDWWL
                            ****
450   RNRDHNGNGVPEYGATRDKAHNTESGEMLFTVKKGDKEETQSGLNNYARV

500   VEKGQYDSLEIPAQVAASWESGRDDAAVFGFIDKEQLDKYVANGGKRSDW

550   TVKFAENRSQDGTLLGYSLLQESVDQASYMYSDNHYLAEMATILGKPEEA

600   KRYRQLAQQLADYINTCMFDPTTQFYYDVRIEDKPLANGCAGKPIVERGK

650   GPEGWSPLFNGAATQANADAVVKVMLDPKEFNTFVPLGTAALTNPAFGAD

700   IYWRGRVWVDQFWFGLKGMERYGYRDDALKLADTFFRHAKGLTADGPIQE

750   NYNPLTGAQQGAPNFSWSAAHLYMLYNDFFRKQ

sp|Q79E92|YKGN_ECOLI Putative transposase YkgN OS=Escherichia coli
(strain K12) OX=83333 GN=ykgN PE=5 SV=1
                                                ****
0   MSGKRYPEEFKTEAVKQVVDLGYSVASVATRLDITTHSLYAWIKKYGPDS
      *************   ********************
50   STNKEQSDAQAEIRRLQKELKRVTDERDILKKAAVDSICQCNTPFNYLFR

```
100   CFELQCLSRSVV

sp|P0ACI3|XYLR_ECOLI Xylose operon regulatory protein OS=Escherichia coli
(strain K12) OX=83333 GN=xylR PE=1 SV=1
                                  ****************
0     MFTKRHRITLLFNANKAYDRQVVEGVGEYLQASQSEWDIFIEEDFRARID
      ****
50    KIKDWLGDGVIADFDDKQIEQALADVDVPIVGVGGSYHLAESYPPVHYIA

100   TDNYALVESAFLHLKEKGVNRFAFYGLPESSGKRWATEREYAFRQLVAEE

150   KYRGVVYQGLETAPENWQHAQNRLADWLQTLPPQTGIIAVTDARARHILQ

200   VCEHLHIPVPEKLCVIGIDNEELTRYLSRVALSSVAQGARQMGYQAAKLL

250   HRLLDKEEMPLQRILVPPVRVIERRSTDYRSLTDPAVIQAMHYIRNHACK

300   GIKVDQVLDAVGISRSNLEKRFKEEVGETIHAMIHAEKLEKARSLLISTT

350   LSINEISQMCGYPSLQYFYSVFKKAYDTTPKEYRDVNSEVML

sp|P76007|NHAP2_ECOLI K(+)/H(+) antiporter NhaP2 OS=Escherichia coli
(strain K12) OX=83333 GN=cvrA PE=1 SV=2

0     MDATTIISLFILGSILVTSSILLSSFSSRLGIPILVIFLAIGMLAGVDGV

50    GGIPFDNYPFAYMVSNLALAIILLDGGMRTQASSFRVALGPALSLATLGV

100   LITSGLTGMMAAWLFNLDLIEGLLIGAIVGSTDAAAVFSLLGGKGLNERV

150   GSTLEIESGSNDPMAVFLTITLIAMIQHHESNISWMFIVDILQQFGLGIV

200   IGLGGGYLLLQMINRIALPAGLYPLLALSGGILIFSLTTALEGSGILAVY

250   LCGFLLGNRPIRNRYGILQNFDGLAWLAQIAMFLVLGLLVNPSDLLPIAI

300   PALILSAWMIFFARPLSVFAGLLPFRGFNLRERVFISWVGLRGAVPIILA

350   VFPMMAGLENARLFFNVAFFVVLVSLLLQGTSLSWAAKKAKVVVPPVGRP

400   VSRVGLDIHPENPWEQFVYQLSADKWCVGAALRDLHMPKETRIAALFRDN

450   QLLHPTGSTRLREGDVLCVIGRERDLPALGKLFSQSPPVALDQRFFGDFI

500   LEASAKYADVALIYGLEDGREYRDKQQTLGEIVQQLLGAAPVVGDQVEFA
             *********************
550   GMIWTVAEKEDNEVLKIGVRVAEEEAES

sp|P52106|CSGD_ECOLI CsgBAC operon transcriptional regulatory protein
OS=Escherichia coli (strain K12) OX=83333 GN=csgD PE=1 SV=1
                                                 *****
0     MFNEVHSIHGHTLLLITKSSLQATALLQHLKQSLAITGKLHNIQRSLDDI
      *****************
50    SSGSIILLDMMEADKKLIHYWQDTLSRKNNNIKILLLNTPEDYPYRDIEN
```

```
100   WPHINGVFYSMEDQERVVNGLQGVLRGECYFTQKLASYLITHSGNYRYNS

150   TESALLTHREKEILNKLRIGASNNEIARSLFISENTVKTHLYNLFKKIAV

200   KNRTQAVSWANDNLRR

sp|P61887|RMLA2_ECOLI Glucose-1-phosphate thymidylyltransferase 2
OS=Escherichia coli (strain K12) OX=83333 GN=rffH PE=1 SV=1

0     MKGIILAGGSGTRLHPITRGVSKQLLPIYDKPMIYYPLSVLMLAGIREIL

50    IITTPEDKGYFQRLLGDGSEFGIQLEYAEQPSPDGLAQAFIIGETFLNGE
                                                  ****
100   PSCLVLGDNIFFGQGFSPKLRHVAARTEGATVFGYQVMDPERFGVVEFDD
      ****************          ********************
150   NFRAISLEEKPKQPKSNWAVTGLYFYDSKVVEYAKQVKPSERGELEITSI

200   NQMYLEAGNLTVELLGRGFAWLDTGTHDSLIEASTFVQTVEKRQGFKIAC

250   LEEIAWRNGWLDDEGVKRAASSLAKTGYGQYLLELLRARPRQY

sp|P62601|TREF_ECOLI Cytoplasmic trehalase OS=Escherichia coli (strain
K12) OX=83333 GN=treF PE=1 SV=1
                            ********************
0     MLNQKIQNPNPDELMIEVDLCYELDPYELKLDEMIEAEPEPEMIEGLPAS

50    DALTPADRYLELFEHVQSAKIFPDSKTFPDCAPKMDPLDILIRYRKVRRH
                      ********************
100   RDFDLRKFVENHFWLPEVYSSEYVSDPQNSLKEHIDQLWPVLTREPQDHI

150   PWSSLLALPQSYIVPGGRFSETYYWDSYFTMLGLAESGREDLLKCMADNF

200   AWMIENYGHIPNGNRTYYLSRSQPPVFALMVELFEEDGVRGARRYLDHLK
                                          ****************
250   MEYAFWMDGAESLIPNQAYRHVVRMPDGSLLNRYWDDRDTPRDESWLEDV
      ******
300   ETAKHSGRPPNEVYRDLRAGAASGWDYSSRWLRDTGRLASIRTTQFIPID

350   LNAFLFKLESAIANISALKGEKETEALFRQKASARRDAVNRYLWDDENGI

400   YRDYDWRREQLALFSAAAIVPLYVGMANHEQADRLANAVRSRLLTPGGIL

450   ASEYETGEQWDKPNGWAPLQWMAIQGFKMYGDDLLGDEIARSWLKTVNQF

500   YLEQHKLIEKYHIADGVPREGGGGEYPLQDGFGWTNGVVRRLIGLYGEP

sp|P27129|RFAJ_ECOLI Lipopolysaccharide 1,2-glucosyltransferase
OS=Escherichia coli (strain K12) OX=83333 GN=rfaJ PE=3 SV=2

0     MDSFPAIEIDKVKAWDFRLANINTSECLNVAYGVDANYLDGVGVSITSIV

50    LNNRHINLDFYIIADVYNDGFFQKIAKLAEQNQLRITLYRINTDKLQCLP
```

```
100   CTQVWSRAMYFRLFAFQLLGLTLDRLLYLDADVVCKGDISQLLHLGLNGA
                                                    ************
150   VAAVVKDVEPMQEKAVSRLSDPELLGQYFNSGVVYLDLKKWADAKLTEKA
      ********                                 ***********
200   LSILMSKDNVYKYPDQDVMNVLLKGMTLFLPREYNTIYTIKSELKDKTHQ
      **********                       *****************
250   NYKKLITESTLLIHYTGATKPWHKWAIYPSVKYYKIALENSPWKDDSPRD
      **************
300   AKSIIEFKKRYKHLLVQHHYISGIIAGVCYLCRKYYRK
```

sp|P45766|YHDW_ECOLI Putative amino-acid ABC transporter-binding protein YhdW OS=Escherichia coli (strain K12) OX=83333 GN=yhdW PE=1 SV=2

```
0     MKKMMIATLAAASVLLAVANQAXAGATLDAVQKKGFVQCGISDGLPGFSY

50    ADADGKFSGIDVDICRGVAAAVFGDDTKVKYTPLTAKERFTALQSGEVDL

100   LSRNTTWTSSRDAGMGMAFTGVTYYDGIGFLTHDKAGLKSAKELDGATVC
                                 ***********************
150   IQAGTDTELNVADYFKANNMKYTPVTFDRSDESAKALESGRCDTLASDQS

200   QLYALRIKLSNPAEWIVLPEVISKEPLGPVVRRGDDEWFSIVRWTLFAML

250   NAEEMGINSQNVDEKAANPATPDMAHLLGKEGDYGKDLKLDNKWAYNIIK

300   QVGNYSEIFERNVGSESPLKIKRGQNNLWNNGGIQYAPPVR
```

sp|P76507|YFDI_ECOLI Uncharacterized protein YfdI OS=Escherichia coli (strain K12) OX=83333 GN=yfdI PE=4 SV=1

```
0     MNKAIKVSLYISFVLIICALSKNIMMLNTSDFGRAIKPLIEDIPAFTYDL
         *********************
50    PLLYKLKGHIDSIDSYEYISSYSYILYTYVLFISIFTEYLDARVLSLFLK

100   VIYIYSLYAIFTSYIKTERYVTLFTFFILAFLMCSSSTLSMFASFYQEQI

150   VIIFLPFLVYSLTCKNNKSMLLLFFSLLIISTAKNQFILTPLIVYSYYIF

200   FDRHKLIIKSVICVVCLLASIFAISYSKGVVELNKYHATYFGSYLYMKNN

250   GYKMPSYVDDKCVGLDAWGNKFDISFGATPTEVGTECFESHKDETFSNAL

300   FLLVSKPSTIFKLPFDDGVMSQYKENYFHVYKKLHVIYGESNILTTITNI

350   KDNIFKNIRFISLLLFFIASIFIRNNKIKASLFVVSLFGISQFYVSFFGE

400   GYRDLSKHLFGMYFSFDLCLYITVVFLIYKIIQRNQDNSDVKH
```

sp|P24215|UXUA_ECOLI Mannonate dehydratase OS=Escherichia coli (strain K12) OX=83333 GN=uxuA PE=1 SV=2

```
0     MEQTWRWYGPNDPVSLADVRQAGATGVVTALHHIPNGEVWSVEEILKRKA

50    IIEDAGLVWSVVESVPIHEDIKTHTGNYEQWIANYQQTLRNLAQCGIRTV
```

100 CYNFMPVLDWTRTDLEYVLPDGSKALRFDQIEFAAFEMHILKRPGAEADY

150 TEEEIAQAAERFATMSDEDKARLTRNIIAGLPGAEEGYTLDQFRKHLELY

200 KDIDKAKLRENFAVFLKAIIPVAEEVGVRMAVHPDDPPRPILGLPRIVST

250 IEDMQWMVDTVNSMANGFTMCTGSYGVRADNDLVDMIKQFGPRIYFTHLR
****

300 STMREDNPKTFHEAAHLNGDVDMYEVVKAIVEEEHRRKAEGKEDLIPMRP

350 DHGHQMLDDLKKKTNPGYSAIGRLKGLAEVRGVELAIQRAFFSR

sp|P0ABU7|EXBB_ECOLI Biopolymer transport protein ExbB OS=Escherichia coli (strain K12) OX=83333 GN=exbB PE=1 SV=1

0 MGNNLMQTDLSVWGMYQHADIVVKCVMIGLILASVVTWAIFFSKSVEFFN
****

50 QKRRLKREQQLLAEARSLNQANDIAADFGSKSLSLHLLNEAQNELELSEG
****

100 SDDNEGIKERTSFRLERRVAAVGRQMGRGNGYLATIGAISPFVGLFGTVW

150 GIMNSFIGIAQTQTTNLAVVAPGIAEALLATAIGLVAAIPAVVIYNVFAR

200 QIGGFKAMLGDVAAQVLLLQSRDLDLEASAAAHPVRVAQKLRAG

sp|Q46829|BGLA_ECOLI 6-phospho-beta-glucosidase BglA OS=Escherichia coli (strain K12) OX=83333 GN=bglA PE=1 SV=2

0 MIVKKLTLPKDFLWGGAVAAHQVEGGWNKGGKGPSICDVLTGGAHGVPRE

50 ITKEVLPGKYYPNHEAVDFYGHYKEDIKLFAEMGFKCFRTSIAWTRIFPK

100 GDEAQPNEEGLKFYDDMFDELLKYNIEPVITLSHFEMPLHLVQQYGSWTN

150 RKVVDFFVRFAEVVFERYKHKVKYWMTFNEINNQRNWRAPLFGYCCSGVV

200 YTEHENPEETMYQVLHHQFVASALAVKAARRINPEMKVGCMLAMVPLYPY

250 SCNPDDVMFAQESMRERYVFTDVQLRGYYPSYVLNEWERRGFNIKMEDGD

300 LDVLREGTCDYLGFSYYMTNAVKAEGGTGDAISGFEGSVPNPYVKASDWG

350 WQIDPVGLRYALCELYERYQRPLFIVENGFGAYDKVEEDGSINDDYRIDY

400 LRAHIEEMKKAVTYDGVDLMGYTPWGCIDCVSFTTGQYSKRYGFIYVNKH
****

450 DDGTGDMSRSRKKSFNWYKEVIASNGEKL

sp|P0AB24|EFEO_ECOLI Iron uptake system component EfeO OS=Escherichia coli (strain K12) OX=83333 GN=efeO PE=1 SV=1

0 MTINFRRNALQLSVAALFSSAFMANAADVPQVKVTVTDKQCEPMTITVNA

```
50    GKTQFIIQNHSQKALEWEILKGVMVVEERENIAPGFSQKMTANLQPGEYD

100   MTCGLLTNPKGKLIVKGEATADAAQSDALLSLGGAITAYKAYVMAETTQL
                                                 *************
150   VTDTKAFTDAIKAGDIEKAKALYAPTRQHYERIEPIAELFSDLDGSIDAR
      *************
200   EDDYEQKAADPKFTGFHRLEKALFGDNTTKGMDQYAEQLYTDVVDLQKRI
                       *********************
250   SELAFPPSKVVGGAAGLIEEVAASKISGEEDRYSHTDLWDFQANVEGSQK

300   IVDLLRPQLQKANPELLAKVDANFKKVDTILAKYRTKDGFETYDKLTDAD

350   RNALKGPITALAEDLAQLRGVLGLD
```

sp|P0AGJ9|SYY_ECOLI Tyrosine--tRNA ligase OS=Escherichia coli (strain K12) OX=83333 GN=tyrS PE=1 SV=2

```
0     MASSNLIKQLQERGLVAQVTDEEALAERLAQGPIALYCGFDPTADSLHLG

50    HLVPLLCLKRFQQAGHKPVALVGGATGLIGDPSFKAAERKLNTEETVQEW

100   VDKIRKQVAPFLDFDCGENSAIAANNYDWFGNMNVLTFLRDIGKHFSVNQ

150   MINKEAVKQRLNREDQGISFTEFSYNLLQGYDFACLNKQYGVVLQIGGSD

200   QWGNITSGIDLTRRLHQNQVFGLTVPLITKADGTKFGKTEGGAVWLDPKK
                               ***********************
250   TSPYKFYQFWINTADADVYRFLKFFTFMSIEEINALEEEDKNSGKAPRAQ

300   YVLAEQVTRLVHGEEGLQAAKRITECLFSGSLSALSEADFEQLAQDGVPM

350   VEMEKGADLMQALVDSELQPSRGQARKTIASNAITINGEKQSDPEYFFKE

400   EDRLFGRFTLLRRGKKNYCLICWK
```

sp|P0AFA7|NHAB_ECOLI Na(+)/H(+) antiporter NhaB OS=Escherichia coli (strain K12) OX=83333 GN=nhaB PE=1 SV=1

```
0     MEISWGRALWRNFLGQSPDWYKLALIIFLIVNPLIFLISPFVAGWLLVAE

50    FIFTLAMALKCYPLLPGGLLAIEAVFIGMTSAEHVREEVAANLEVLLLLM

100   FMVAGIYFMKQLLLFIFTRLLLSIRSKMLLSLSFCVAAAFLSAFLDALTV
                      ***************************
150   VAVVISVAVGFYGIYHRVASSRTEDTDLQDDSHIDKHYKVVLEQFRGFLR

200   SLMMHAGVGTALGGVMTMVGEPQNLIIAKAAGWHFGDFFLRMSPVTVPVL

250   ICGLLTCLLVEKLRWFGYGETLPEKVREVLQQFDDQSRHQRTRQDKIRLI

300   VQAIIGVWLVTALALHLAEVGLIGLSVIILATSLTGVTDEHAIGKAFTES

350   LPFTALLTVFFSVVAVIIDQQLFSPIIQFVLQASEHAQLSLFYIFNGLLS
```

```
400   SISDNVFVGTIYINEAKAAMESGAITLKQYELLAVAINTGTNLPSVATPN

450   GQAAFLFLLTSALAPLIRLSYGRMVWMALPYTLVLTLVGLLCVEFTLAPV

500   TEWFMQMGWIATL
```

sp|P77174|YBDM_ECOLI Uncharacterized protein YbdM OS=Escherichia coli (strain K12) OX=83333 GN=ybdM PE=1 SV=1

```
0     MGDTMQQRLTQDLTQFLASLPEDDRIKAINEIRMAIHQVSPFREEPVDCV

50    LWVKNSQLMPNDYNPNNVAPPEKKLLQKSIEIDGFTQPIVVTHTDKNAME

100   IVDGFHRHEIGKGSSSLKLRLKGYLPVTCLEGTRNQRIAATIRHNRARGR
                   *************************
150   HQITAMSEIVRELSQLGWDDNKIGKELGMDSDEVLRLKQINGLQELFADR

200   QYSRAWTVK
```

sp|P08622|DNAJ_ECOLI Chaperone protein DnaJ OS=Escherichia coli (strain K12) OX=83333 GN=dnaJ PE=1 SV=3

```
                                      ******************
0     MAKQDYYEILGVSKTAEEREIRKAYKRLAMKYHPDRNQGDKEAEAKFKEI
      *************
50    KEAYEVLTDSQKRAAYDQYGHAAFEQGGMGGGGFGGGADFSDIFGDVFGD

100   IFGGGRGRQRAARGADLRYNMELTLEEAVRGVTKEIRIPTLEECDVCHGS

150   GAKPGTQPQTCPTCHGSGQVQMRQGFFAVQQTCPHCQGRGTLIKDPCNKC

200   HGHGRVERSKTLSVKIPAGVDTGDRIRLAGEGEAGEHGAPAGDLYVQVQV

250   KQHPIFEREGNNLYCEVPINFAMAALGGEIEVPTLDGRVKLKVPGETQTG

300   KLFRMRGKGVKSVRGGAQGDLLCRVVVETPVGLNERQKQLLQELQESFGG
         ********************
350   PTGEHNSPRSKSFFDGVKKFFDDLTR
```

sp|P25718|AMY1_ECOLI Periplasmic alpha-amylase OS=Escherichia coli (strain K12) OX=83333 GN=malS PE=1 SV=1

```
0     MKLAACFLTLLPGFAVAASWTSPGFPAFSEQGTGTFVSHAQLPKGTRPLT

50    LNFDQQCWQPADAIKLNQMLSLQPCSNTPPQWRLFRDGEYTLQIDTRSGT

100   PTLMISIQNAAEPVASLVRECPKWDGLPLTVDVSATFPEGAAVRDYYSQQ

150   IAIVKNGQIMLQPAATSNGLLLLERAETDTSAPFDWHNATVYFVLTDRFE

200   NGDPSNDQSYGRHKDGMAEIGTFHGGDLRGLTNKLDYLQQLGVNALWISA

250   PFEQIHGWVGGGTKGDFPHYAYHGYYTQDWTNLDANMGNEADLRTLVDSA
                                     *******************
300   HQRGIRILFDVVMNHTGYATLADMQEYQFGALYLSGDEVKKSLGERWSDW
```

      ****
350   KPAAGQTWHSFNDYINFSDKTGWDKWWGKNWIRTDIGDYDNPGFDDLTMS

400   LAFLPDIKTESTTASGLPVFYKNKMDTHAKAIDGYTPRDYLTHWLSQWVR

450   DYGIDGFRVDTAKHVELPAWQQLKTEASAALREWKKANPDKALDDKPFWM

500   TGEAWGHGVMQSDYYRHGFDAMINFDYQEQAAKAVDCLAQMDTTWQQMAE

550   KLQGFNVLSYLSSHDTRLFREGGDKAAELLLLAPGAVQIFYGDESSRPFG

600   PTGSDPLQGTRSDMNWQDVSGKSAASVAHWQKISQFRARHPAIGAGKQTT

650   LLLKQGYGFVREHGDDKVLVVWAGQQ

sp|P0A9W3|ETTA_ECOLI Energy-dependent translational throttle protein EttA OS=Escherichia coli (strain K12) OX=83333 GN=ettA PE=1 SV=2

0   MAQFVYTMHRVGKVVPPKRHILKNISLSFFPGAKIGVLGLNGAGKSTLLR

50   IMAGIDKDIEGEARPQPDIKIGYLPQEPQLNPEHTVRESIEEAVSEVVNA

100   LKRLDEVYALYADPDADFDKLAAEQGRLEEIIQAHDGHNLNVQLERAADA

150   LRLPDWDAKIANLSGGERRRVALCRLLLEKPDMLLLDEPTNHLDAESVAW

200   LERFLHDFEGTVVAITHDRYFLDNVAGWILELDRGEGIPWEGNYSSWLEQ

250   KDQRLAQEASQEAARRKSIEKELEWVRQGTKGRQSKGKARLARFEELNST

300   EYQKRNETNELFIPPGPRLGDKVLEVSNLRKSYGDRLLIDDLSFSIPKGA
                                                 ****
350   IVGIIGPNGAGKSTLFRMISGQEQPDSGTITLGETVKLASVDQFRDSMDN
      ****
400   SKTVWEEVSGGLDIMKIGNTEMPSRAYVGRFNFKGVDQGKRVGELSGGER

450   GRLHLAKLLQVGGNMLLLDEPTNDLDIETLRALENALLEFPGCAMVISHD
                     ****
500   RWFLDRIATHILDYQDEGKVEFFEGNFTEYEEYKKRTLGADALEPKRIKY

550   KRIAK

sp|P25549|ASLA_ECOLI Putative sulfatase AslA OS=Escherichia coli (strain K12) OX=83333 GN=aslA PE=3 SV=2

0   MEFSFSPKRLVVAVAAALPLMASAADTPSTATARKGFAGYDHPNQYLVKP
                     ****
50   ATTIADNMMPVMQHPAQDKETQQKLAELEKKTGKKPNVVVFLLDDVGWMD

100   VGFNGGGVAVGNPTPDIDAVASQGLILTSAYSQPSSSPTRATILTGQYSI

150   HHGILMPPMYGQPGGLQGLTTLPQLLHDQGYVTQAIGKWHMGENKESQPQ

200   NVGFDDFRGFNSVSDMYTEWRDVHVNPEVALSPDRSEYIKQLPFSKDDVH

250   AVRGGEQQAIADITPKYMEDLDQRWMDYGVKFLDKMAKSDKPFFLYYGTR

300   GCHFDNYPNAKYAGSSPARTSYGDCMVEMNDVFANLYKTLEKNGQLDNTL

350   IVFTSDNGPEAEVPPHGRTPFRGAKGSTWEGGVRVPTFVYWKGMIQPRKS

400   DGIVDLADLFPTALDLAGHPGAKVANLVPKTTFIDGVDQTSFFLGTNGQS

450   NRKAEHYFLNGKLAAVRMDEFKYHVLIQQPYAYTQSGYQGGFTGTVMQTA

500   GSSVFNLYTDPQESDSIGVRHIPMGVPLQTEMHAYMEILKKYPPRAQIKS

550   D

sp|P39360|YJHI_ECOLI Uncharacterized HTH-type transcriptional regulator YjhI OS=Escherichia coli (strain K12) OX=83333 GN=yjhI PE=4 SV=1

```
0     MVRKGCNSLVRAEKILTHIAWVGMASYMELLNKFQYPKSSLLNLLNVMVD

50    CGFLIKNKNGYYSLGIKNYELGCQALHRQNIFEVTKRPMQELSLKSGLVC
           *******************************
100   HLGAMESISAIYLDKIESPDSVPTSKSWIGKKLELHITALGKALLAWKTR

150   EELDYFLEALTLTPHTRNTFTDKKLFLEELQKTRLRGWAIDNEESTYGAV

200   CLSMPVFNMYNRVNYAISLSGDPVVYSGNKIDSYLELLRKCAEQISYGLG

250   YRNENEHLRKGN
```

sp|P0ACI6|ASNC_ECOLI Regulatory protein AsnC OS=Escherichia coli (strain K12) OX=83333 GN=asnC PE=1 SV=1

```
0     MENYLIDNLDRGILEALMGNARTAYAELAKQFGVSPGTIHVRVEKMKQAG
                              ***********************
50    IITGARIDVSPKQLGYDVGCFIGIILKSAKDYPSALAKLESLDEVTEAYY

100   TTGHYSIFIKVMCRSIDALQHVLINKIQTIDEIQSTETLIVLQNPIMRTI

150   KP
```

sp|P29013|YCGB_ECOLI Uncharacterized protein YcgB OS=Escherichia coli (strain K12) OX=83333 GN=ycgB PE=4 SV=2

```
0     MATIDSMNKDTTRLSDGPDWTFDLLDVYLAEIDRVAKLYRLDTYPHQIEV

50    ITSEQMMDAYSSVGMPINYPHWSFGKKFIETERLYKHGQQGLAYEIVINS

100   NPCIAYLMEENTITMQALVMAHACYGHNSFFKNNYLFRSWTDASSIVDYL
                                           *************
150   IFARKYITECEERYGVDEVERLLDSCHALMNYGVDRYKRPQKISLQEEKA
      ***********
200   RQKSREEYLQSQVNMLWRTLPKREEEKTVAEARRYPSEPQENLLYFMEKN
```

```
250   APLLESWQREILRIVRKVSQYFYPQKQTQVMNEGWATFWHYTILNHLYDE

300   GKVTERFMLEFLHSHTNVVFQPPYNSPWYSGINPYALGFAMFQDIKRICQ

350   SPTEEDKYWFPDIAGSDWLETLHFAMRDFKDESFISQFLSPKVMRDFRFF

400   TVLDDDRHNYLEISAIHNEEGYREIRNRLSSQYNLSNLEPNIQIWNVDLR

450   GDRSLTLRYIPHNRAPLDRGRKEVLKHVHRLWGFDVMLEQQNEDGSIELL

500   ERCPPRMGNL
```

sp|P0A805|RRF_ECOLI Ribosome-recycling factor OS=Escherichia coli (strain K12) OX=83333 GN=frr PE=1 SV=1

```
0     MISDIRKDAEVRMDKCVEAFKTQISKIRTGRASPSLLDGIVVEYYGTPTP

50    LRQLASVTVEDSRTLKINVFDRSMSPAVEKAIMASDLGLNPNSAGSDIRV
                                    ********************
100   PLPPLTEERRKDLTKIVRGEAEQARVAVRNVRRDANDKVKALLKDKEISE
      ********************
150   DDDRRSQDDVQKLTDAAIKKIEAALADKEAELMQF
```

sp|P09152|NARG_ECOLI Respiratory nitrate reductase 1 alpha chain OS=Escherichia coli (strain K12) OX=83333 GN=narG PE=1 SV=4

```
0     MSKFLDRFRYFKQKGETFADGHGQLLNTNRDWEDGYRQRWQHDKIVRSTH

50    GVNCTGSCSWKIYVKNGLVTWETQQTDYPRTRPDLPNHEPRGCPRGASYS
                                  **********************
100   WYLYSANRLKYPMMRKRLMKMWREAKALHSDPVEAWASIIEDADKAKSFK

150   QARGRGGFVRSSWQEVNELIAASNVYTIKNYGPDRVAGFSPIPAMSMVSY

200   ASGARYLSLIGGTCLSFYDWYCDLPPASPQTWGEQTDVPESADWYNSSYI

250   IAWGSNVPQTRTPDAHFFTEVRYKGTKTVAVTPDYAEIAKLCDLWLAPKQ

300   GTDAAMALAMGHVMLREFHLDNPSQYFTDYVRRYTDMPMLVMLEERDGYY

350   AAGRMLRAADLVDALGQENNPEWKTVAFNTNGEMVAPNGSIGFRWGEKGK

400   WNLEQRDGKTGEETELQLSLLGSQDEIAEVGFPYFGGDGTEHFNKVELEN

450   VLLHKLPVKRLQLADGSTALVTTVYDLTLANYGLERGLNDVNCATSYDDV

500   KAYTPAWAEQITGVSRSQIIRIAREFADNADKTHGRSMIIVGAGLNHWYH

550   LDMNYRGLINMLIFCGCVGQSGGGWAHYVGQEKLRPQTGWQPLAFALDWQ

600   RPARHMNSTSYFYNHSSQWRYETVTAEELLSPMADKSRYTGHLIDFNVRA

650   ERMGWLPSAPQLGTNPLTIAGEAEKAGMNPVDYTVKSLKEGSIRFAAEQP
```

```
700   ENGKNHPRNLFIWRSNLLGSSGKGHEFMLKYLLGTEHGIQGKDLGQQGGV

750   KPEEVDWQDNGLEGKLDLVVTLDFRLSSTCLYSDIILPTATWYEKDDMNT
                      **********************
800   SDMHPFIHPLSAAVDPAWEAKSDWEIYKAIAKKFSEVCVGHLGKETDIVT

850   LPIQHDSAAELAQPLDVKDWKKGECDLIPGKTAPHIMVVERDYPATYERF

900   TSIGPLMEKIGNGGKGIAWNTQSEMDLLRKLNYTKAEGPAKGQPMLNTAI

950   DAAEMILTLAPETNGQVAVKAWAALSEFTGRDHTHLALNKEDEKIRFRDI

1000  QAQPRKIISSPTWSGLEDEHVSYNAGYTNVHELIPWRTLSGRQQLYQDHQ

1050  WMRDFGESLLVYRPPIDTRSVKEVIGQKSNGNQEKALNFLTPHQKWGIHS

1100  TYSDNLLMLTLGRGGPVVWLSEADAKDLGIADNDWIEVFNSNGALTARAV

1150  VSQRVPAGMTMMYHAQERIVNLPGSEITQQRGGIHNSVTRITPKPTHMIG

1200  GYAHLAYGFNYYGTVGSNRDEFVVVRKMKNIDWLDGEGNDQVQESVK

sp|P0AG80|UGPB_ECOLI sn-glycerol-3-phosphate-binding periplasmic protein
UgpB OS=Escherichia coli (strain K12) OX=83333 GN=ugpB PE=1 SV=1

0     MKPLHYTASALALGLALMGNAQAVTTIPFWHSMEGELGKEVDSLAQRFNA

50    ENPDYKIVPTYKGNYEQNLSAGIAAFRTGNAPAILQVYEVGTATMMASKA

100   IKPVYDVFKEAGIQFDESQFVPTVSGYYSDSKTGHLLSQPFNSSTPVLYY

150   NKDAFKKAGLDPEQPPKTWQDLADYAAKLKASGMKCGYASGWQGWIQLEN
                                         ***************
200   FSAWNGLPFASKNNGFDGTDAVLEFNKPEQVKHIAMLEEMNKKGDFSYVG
      ***********
250   RKDESTEKFYNGDCAMTTASSGSLANIREYAKFNYGVGMMPYDADAKDAP

300   QNAIIGGASLWVMQGKDKETYTGVAKFLDFLAKPENAAEWHQKTGYLPIT

350   KAAYDLTREQGFYEKNPGADTATRQMLNKPPLPFTKGLRLGNMPQIRVIV

400   DEELESVWTGKKTPQQALDTAVERGNQLLRRFEKSTKS

sp|P0A7G2|RBFA_ECOLI 30S ribosome-binding factor OS=Escherichia coli
(strain K12) OX=83333 GN=rbfA PE=1 SV=2

0     MAKEFGRPQRVAQEMQKEIALILQREIKDPRLGMMTTVSGVEMSRDLAYA

50    KVYVTFLNDKDEDAVKAGIKALQEASGFIRSLLGKAMRLRIVPELTFFYD
                  *********************
100   NSLVEGMRMSNLVTSVVKHDEERRVNPDDSKED

sp|Q46942|YQEI_ECOLI Uncharacterized protein YqeI OS=Escherichia coli
(strain K12) OX=83333 GN=yqeI PE=4 SV=1
```

```
0     MYWIINDNIEFWPEHRKLISVHNADLNVVLTTPASRCLSLLLEAFPDVVA

50    QQDFFTRVWEEEGMRVPTNTLYQNISIIRRGFRAVGDTTHSLIATVPRRG

100   FKIHNDINIQNHVINSSTDAHTHNAPPAIKVNAGYKESIGGAKNFNNKIL

150   KHIKSHLIMLSAFVIGAYSAYWLWNNNQPKPFFKDYKTVAEINGCHFNVT
      ********************
200   EDTIDGLKEFDKYKTRILDSGINCKKHPWLYFPLAKSSPGMIVMACNKNY

250   NQHEVANCLTLSYREVNRD
```

sp|P33234|ADIY_ECOLI HTH-type transcriptional regulator AdiY OS=Escherichia coli (strain K12) OX=83333 GN=adiY PE=4 SV=1

```
0     MRICSDQPCIVLLTEKDVWIRVNGKEPISLKANHMALLNCENNIIDVSSL

50    NNTLVAHISHDIIKDYLRFLNKDLSQIPVWQRSATPILTLPCLTPDVFRV

100   AAQHSMMPAETESEKERTRALLFTVLSRFLDSKKFVSLMMYMLRNCVSDS
                         *************************
150   VYQIIESDIHKDWNLSMVASCLCLSPSLLKKKLKSENTSYSQIITTCRMR

200   YAVNELMMDGKNISQVSQSCGYNSTSYFISVFKDFYGMTPLHYVSQHRER

250   TVA
```

sp|P23367|MUTL_ECOLI DNA mismatch repair protein MutL OS=Escherichia coli (strain K12) OX=83333 GN=mutL PE=1 SV=2

```
0     MPIQVLPPQLANQIAAGEVVERPASVVKELVENSLDAGATRIDIDIERGG

50    AKLIRIRDNGCGIKKDELALALARHATSKIASLDDLEAIISLGFRGEALA

100   SISSVSRLTLTSRTAEQQEAWQAYAEGRDMNVTVKPAAHPVGTTLEVLDL

150   FYNTPARRKFLRTEKTEFNHIDEIIRRIALARFDVTINLSHNGKIVRQYR

200   AVPEGGQKERRLGAICGTAFLEQALAIEWQHGDLTLRGWVADPNHTTPAL

250   AEIQYCYVNGRMMRDRLINHAIRQACEDKLGADQQPAFVLYLEIDPHQVD

300   VNVHPAKHEVRFHQSRLVHDFIYQGVLSVLQQQLETPLPLDDEPQPAPRS

350   IPENRVAAGRNHFAEPAAREPVAPRYTPAPASGSRPAAPWPNAQPGYQKQ

400   QGEVYRQLLQTPAPMQKLKAPEPQEPALAANSQSFGRVLTIVHSDCALLE
                                             ***********
450   RDGNISLLSLPVAERWLRQAQLTPGEAPVCAQPLLIPLRLKVSAEEKSAL
      **********
500   EKAQSALAELGIDFQSDAQHVTIRAVPLPLRQQNLQILIPELIGYLAKQS

550   VFEPGNIAQWIARNLMSEHAQWSMAQAITLLADVERLCPQLVKTPPGGLL
```

600 QSVDLHPAIKALKDE

sp|P39172|ZNUA_ECOLI High-affinity zinc uptake system protein ZnuA OS=Escherichia coli (strain K12) OX=83333 GN=znuA PE=1 SV=4

0 MLHKKTLLFAALSAALWGGATQAADAAVVASLKPVGFIASAIADGVTETE

50 VLLPDGASEHDYSLRPSDVKRLQNADLVVWVGPEMEAFMQKPVSKLPGAK
      ****
100 QVTIAQLEDVKPLLMKSIHGDDDDHDHAEKSDEDHHHGDFNMHLWLSPEI

150 ARATAVAIHGKLVELMPQSRAKLDANLKDFEAQLASTETQVGNELAPLKG

200 KGYFVFHDAYGYFEKQFGLTPLGHFTVNPEIQPGAQRLHEIRTQLVEQKA

250 TCVFAEPQFRPAVVESVARGTSVRMGTLDPLGTNIKLGKTSYSEFLSQLA

300 NQYASCLKGD

sp|P39830|YBAL_ECOLI Putative cation/proton antiporter YbaL OS=Escherichia coli (strain K12) OX=83333 GN=ybaL PE=1 SV=2

0 MHHATPLITTIVGGLVLAFILGMLANKLRISPLVGYLLAGVLAGPFTPGF

50 VADTKLAPELAELGVILLMFGVGLHFSLKDLMAVKAIAIPGAIAQIAVAT

100 LLGMALSAVLGWSLMTGIVFGLCLSTASTVVLLRALEERQLIDSQRGQIA

150 IGWLIVEDLVMVLTLVLLPAVAGMMEQGDVGFATLAVDMGITIGKVIAFI

200 AIMMLVGRRLVPWIMARSAATGSRELFTLSVLALALGVAFGAVELFDVSF

250 ALGAFFAGMVLNESELSHRAAHDTLPLRDAFAVLFFVSVGMLFDPLILIQ

300 QPLAVLATLAIILFGKSLAAFFLVRLFGHSQRTALTIAASLAQIGEFAFI
                                        ****
350 LAGLGMALNLLPQAGQNLVLAGAILSIMLNPVLFALLEKYLAKTETLEEQ
****
400 TLEEAIEEEKQIPVDICNHALLVGYGRVGSLLGEKLLASDIPLVVIETSR

450 TRVDELRERGVRAVLGNAANEEIMQLAHLECAKWLILTIPNGYEAGEIVA

500 SARAKNPDIEIIARAHYDDEVAYITERGANQVVMGEREIARTMLELLETP

550 PAGEVVTG

sp|P21363|YCIE_ECOLI Protein YciE OS=Escherichia coli (strain K12) OX=83333 GN=yciE PE=1 SV=1

0 MNRIEHYHDWLRDAHAMEKQAESMLESMASRIDNYPELRARIEQHLSETK
      ****
50 NQIVQLETILDRNDISRSVIKDSMSKMAALGQSIGGIFPSDEIVKGSISG

100   YVFEQFEIACYTSLLAAAKNAGDTASIPTIEAILNEEKQMADWLIQNIPQ

150   TTEKFLIRSETDGVEAKK

sp|P0A6P1|EFTS_ECOLI Elongation factor Ts OS=Escherichia coli (strain K12) OX=83333 GN=tsf PE=1 SV=2

0   MAEITASLVKELRERTGAGMMDCKKALTEANGDIELAIENMRKSGAIKAA

50   KKAGNVAADGVIKTKIDGNYGIILEVNCQTDFVAKDAGFQAFADKVLDAA

100   VAGKITDVEVLKAQFEEERVALVAKIGENINIRRVAALEGDVLGSYQHGA
****
150   RIGVLVAAKGADEELVKHIAMHVAASKPEFIKPEDVSAEVVEKEYQVQLD

200   IAMQSGKPKEIAEKMVEGRMKKFTGEVSLTGQPFVMEPSKTVGQLLKEHN

250   AEVTGFIRFEVGEGIEKVETDFAAEVAAMSKQS

sp|P25666|HTRL_ECOLI Protein HtrL OS=Escherichia coli (strain K12) OX=83333 GN=htrL PE=2 SV=2

0   MKSSTTIITAYFDIGRGDWTANKGFREKLARSVDVYFSYFERLAALENEM
****
50   IIFTSPDLKPRVEAIRNGKPTTVIVIDIKKKFRYIRSRIEKIQKDESFTN

100   RLEPRQLKNPEYWSPEYVLVCNLKAYFVNKAINMGLVKTPLVAWIDFGYC

150   HKPNVTRGLKIWDFPFDESKMHLFTIKKGLTVTSQQQVFDFMIGNHVYII

200   GGAIVGSQHKWKEFYKLVLESQKITLNNNIVDDDQGIFVMCYYKRPDLFN

250   LNYLGRGKWFDLFRCFRSNTLGAKMQALRIFLSRK

sp|P25714|YIDC_ECOLI Membrane protein insertase YidC OS=Escherichia coli (strain K12) OX=83333 GN=yidC PE=1 SV=2

0   MDSQRNLLVIALLFVSFMIWQAWEQDKNPQPQAQQTTQTTTTAAGSAADQ

50   GVPASGQGKLISVKTDVLDLTINTRGGDVEQALLPAYPKELNSTQPFQLL

100   ETSPQFIYQAQSGLTGRDGPDNPANGPRPLYNVEKDAYVLAEGQNELQVP

150   MTYTDAAGNTFTKTFVLKRGDYAVNVNYNVQNAGEKPLEISSFGQLKQSI
****
200   TLPPHLDTGSSNFALHTFRGAAYSTPDEKYEKYKFDTIADNENLNISSKG

250   GWVAMLQQYFATAWIPHNDGTNNFYTANLGNGIAAIGYKSQPVLVQPGQT

300   GAMNSTLWVGPEIQDKMAAVAPHLDLTVDYGWLWFISQPLFKLLKWIHSF

350   VGNWGFSIIIIITFIVRGIMYPLTKAQYTSMAKMRMLQPKIQAMRERLGDD

400   KQRISQEMMALYKAEKVNPLGGCFPLLIQMPIFLALYYMLMGSVELRQAP

450   FALWIHDLSAQDPYYILPILMGVTMFFIQKMSPTTVTDPMQQKIMTFMPV

500   IFTVFFLWFPSGLVLYYIVSNLVTIIQQQLIYRGLEKRGLHSREKKKS

sp|P77169|YAGJ_ECOLI Protein YagJ OS=Escherichia coli (strain K12) OX=83333 GN=yagJ PE=4 SV=1

                                              ****
0   MEARVTVAGMGLVMEVQDYFDGEADRLAKAWLAEYTPQIKSLKDERKEAY
      ****
50   RQIVEMSTEPQDVDLVRPANKFEMTRVREGEKEADLPVWKHHLLCDESGN

100   YPALLNHWETKVFEIETKREGFAFWYRNPQYTGQSSLGIAYVEAEQYKIV

150   RPDFLFFAEQDGKMVVDLVDPHSLHLADALPKLEGLALYAEHHSDAYRRI

200   ESVAEVKGKLRVLDLKRQDVQDAVATAENAETLFSSGLADDYQ

sp|P0AFJ7|PITA_ECOLI Low-affinity inorganic phosphate transporter 1 OS=Escherichia coli (strain K12) OX=83333 GN=pitA PE=1 SV=1

0   MLHLFAGLDLHTGLLLLLALAFVLFYEAINGFHDTANAVATVIYTRAMRS

50   QLAVVMAAVFNFLGVLLGGLSVAYAIVHMLPTDLLLNMGSSHGLAMVFSM

100   LLAAIIWNLGTWYFGLPASSSHTLIGAIIGIGLTNALMTGTSVVDALNIP

150   KVLSIFGSLIVSPIVGLVFAGGLIFLLRRYWSGTKKRARIHLTPAEREKK

200   DGKKKPPFWTRIALILSAIGVAFSHGANDGQKGIGLVMLVLIGVAPAGFV

250   VNMNATGYEITRTRDAINNVEAYFEQHPALLKQATGADQLVPAPEAGATQ

300   PAEFHCHPSNTINALNRLKGMLTTDVESYDKLSLDQRSQMRRIMLCVSDT
                ****
350   IDKVVKMPGVSADDQRLLKKLKSDMLSTIEYAPVWIIMAVALALGIGTMI

400   GWRRVATTIGEKIGKKGMTYAQGMSAQMTAAVSIGLASYTGMPVSTTHVL

450   SSSVAGTMVVDGGGLQRKTVTSILMAWVFTLPAAVLLSGGLYWLSLQFL

sp|P32674|PFLD_ECOLI Formate acetyltransferase 2 OS=Escherichia coli (strain K12) OX=83333 GN=pflD PE=3 SV=1

0   MTNRISRLKTALFANTREISLERALLYTASHRQTEGEPVILRRAKATAYI

50   LEHVEISIRDEELIAGNRTVKPRAGIMSPEMDPYWLLKELDQFPTRPQDR
         ****
100   FAISEEDKRIYREELFPYWEKRSMKDFINGQMTDEVKAATNTQIFSINQT

150   DKGQGHIIIDYPRLLNHGLGELVAQMQQHCQQQPENHFYQAALLLLEASQ

200   KHILRYAELAETMAANCTDAQRREELLTIAEISRHNAQHKPQTFWQACQL

250   FWYMNIILQYESNASSLSLGRFDQYMLPFYQTSLTQGEDAAFLKELLESL

300   WVKCNDIVLLRSTSSARYFAGFPTGYTALLGGLTENGRSAVNVLSFLCLD

350   AYQSVQLPQPNLGVRTNALIDTPFLMKTAETIRFGTGIPQIFNDEVVVPA

400   FLNRGVSLEDARDYSVVGCVELSIPGRTYGLHDIAMFNLLKVMEICLHEN

450   EGNAALTYEGLLEQIRAKISHYITLMVEGSNICDIGHRDWAPVPLLSSFI

500   SDCLEKGRDITDGGARYNFSGVQGIGIANLSDSLHALKGMVFEQQRLSFD

550   ELLSVLKANFATPEGEKVRARLINRFEKYGNDIDEVDNISAELLRHYCKE

600   VEKYQNPRGGYFTPGSYTVSAHVPLGSVVGATPDGRFAGEQLADGGLSPM

650   LGQDAQGPTAVLKSVSKLDNTLLSNGTLLNVKFTPATLEGEAGLRKLADF

700   LRAFTQLKLQHIQFNVVNADTLREAQQRPQDYAGLVVRVAGYSAFFVELS

750   KEIQDDIIRRTAHQL

sp|Q46834|YGHF_ECOLI Putative type II secretion system C-type protein YghF OS=Escherichia coli (strain K12) OX=83333 GN=yghF PE=3 SV=2

0   MARVVFRDARIYLIQWLTKIRHTLNQRQSLNTDKEHLRKIARGMFWLMLL

50   IISAKMAHSLWRYISFSAEYTAVSQPVNKPSRVDAKTFDKNDVQLISQQN

100   WFGKYQPVAAQVKQPEPVPVAETRLNVVLRGIAFGARPGAVIEEGGKQQV
                                    ****
150   YLQGETLGSHNAVIEEINRDHVMLRYQGKIERLSLAEEERSTVAVTNKKA
      ******
200   VSDEAKQAVAEPAVSVPVEIPAAVRQALAKDPQKIFNYIQLTPVRKEGIV

250   GYAAKPGADRSLFDASGFKEGDIAIALNQQDFTDPRAMIALMRQLPSMDS

300   IQLTVLRKGARHDISIALRYRLFCPPYWKKS

sp|P32684|RLUF_ECOLI 23S rRNA pseudouridine(2604) synthase OS=Escherichia coli (strain K12) OX=83333 GN=rluF PE=1 SV=1

0   MLPDSSVRLNKYISESGICSRREADRYIEQGNVFLNGKRATIGDQVKPGD

50   VVKVNGQLIEPREAEDLVLIALNKPVGIVSTTEDGERDNIVDFVNHSKRV

100   FPIGRLDKDSQGLIFLTNHGDLVNKILRAGNDHEKEYLVTVDKPITEEFI

150   RGMSAGVPILGTVTKKCKVKKEAPFVFRITLVQGLNRQIRRMCEHFGYEV
                        ****
200   KKLERTRIMNVSLSGIPLGEWRDLTDDELIDLFKLIENSSSEVKPKAKAK
      ****
250   PKTAGIKRPVVKMEKTAEKGGRPASNGKRFTSPGRKKKGR

sp|P32106|YIBG_ECOLI Uncharacterized protein YibG OS=Escherichia coli (strain K12) OX=83333 GN=yibG PE=3 SV=1

```
                                      ******************
0     MKACLLLFFYFSFICQLHGADVKIKQNESMMGSTAMTYDLSEEKLMKLKY
      ************
50    KSQHGDSEASFRLYQYYCFTKNNIYKQLRFLERSASQGNVTAQFNYGVFL
                                  **********************
100   SDTNPTLSEYYNLNRAIYWMEFAVNNGNIDAKSKLQELKKLKRMDRRKNK
      **
150   ENP
```

sp|P61949|FLAV_ECOLI Flavodoxin 1 OS=Escherichia coli (strain K12) OX=83333 GN=fldA PE=1 SV=2

```
                            ****************************
0     MAITGIFFGSDTGNTENIAKMIQKQLGKDVADVHDIAKSSKEDLEAYDIL
      *
50    LLGIPTWYYGEAQCDWDDFFPTLEEIDFNGKLVALFGCGDQEDYAEYFCD

100   ALGTIRDIIEPRGATIVGHWPTAGYHFEASKGLADDDHFVGLAIDEDRQP

150   ELTAERVEKWVKQISEELHLDEILNA
```

sp|P76586|YPHH_ECOLI Uncharacterized protein YphH OS=Escherichia coli (strain K12) OX=83333 GN=yphH PE=3 SV=2

```
                                                  ******
0     MRACINNQQIRHHNKCVILELLYRQKRANKSTLARLAQISIPAVSNILQE
      *********************
50    LESEKRVVNIDDESQTRGHSSGTWLIAPEGDWTLCLNVTPTSIECQVANA

100   CLSPKGEFEYLQIDAPTPQALLSEIEKCWHRHRKLWPDHTINLALAIHGQ

150   VDPVTGVSQTMPQAPWTTPVEVKYLLEEKLGIRVMVDNDCVMLALAEKWQ

200   NNSQERDFCVINVDYGIGSSFVINEQIYRGSLYGSGQIGHTIVNPDGVVC

250   DCGRYGCLETVASLSALKKQARVWLKSQPVSTQLDPEKLTTAQLIAAWQS

300   GEPWITSWVDRSANAIGLSLYNFLNILNINQIWLYGRSCAFGENWLNTII

350   RQTGFNPFDRDEGPSVKATQIGFGQLSRAQQVLGIGYLYVEAQLRQI
```

sp|P21179|CATE_ECOLI Catalase HPII OS=Escherichia coli (strain K12) OX=83333 GN=katE PE=1 SV=1

```
                   ************************
0     MSQHNEKNPHQHQSPLHDSSEAKPGMDSLAPEDGSHRPAAEPTPPGAQPT

50    APGSLKAPDTRNEKLNSLEDVRKGSENYALTTNQGVRIADDQNSLRAGSR

100   GPTLLEDFILREKITHFDHERIPERIVHARGSAAHGYFQPYKSLSDITKA

150   DFLSDPNKITPVFVRFSTVQGGAGSADTVRDIRGFATKFYTEEGIFDLVG

200   NNTPIFFIQDAHKFPDFVHAVKPEPHWAIPQGQSAHDTFWDYVSLQPETL
```

250 HNVMWAMSDRGIPRSYRTMEGFGIHTFRLINAEGKATFVRFHWKPLAGKA
****

300 SLVWDEAQKLTGRDPDFHRRELWEAIEAGDFPEYELGFQLIPEEDEFKFD
****

350 FDLLDPTKLIPEELVPVQRVGKMVLNRNPDNFFAENEQAAFHPGHIVPGL

400 DFTNDPLLQGRLFSYTDTQISRLGGPNFHEIPINRPTCPYHNFQRDGMHR

450 MGIDTNPANYEPNSINDNWPRETPPGPKRGGFESYQERVEGNKVRERSPS

500 FGEYYSHPRLFWLSQTPFEQRHIVDGFSFELSKVVRPYIRERVVDQLAHI

550 DLTLAQAVAKNLGIELTDDQLNITPPPDVNGLKKDPSLSLYAIPDGDVKG

600 RVVAILLNDEVRSADLLAILKALKAKGVHAKLLYSRMGEVTADDGTVLPI

650 AATFAGAPSLTVDAVIVPCGNIADIADNGDANYYLMEAYKHLKPIALAGD

700 ARKFKATIKIADQGEEGIVEADSADGSFMDELLTLMAAHRVWSRIPKIDK

750 IPA

sp|P32140|SQUS_ECOLI Sulfoquinovose isomerase OS=Escherichia coli (strain K12) OX=83333 GN=yihS PE=1 SV=2

0 MKWFNTLSHNRWLEQETDRIFDFGKNSVVPTGFGWLGNKGQIKEEMGTHL

50 WITARMLHVYSVAAAMGRPGAYSLVDHGIKAMNGALRDKKYGGWYACVND
****

100 EGVVDASKQGYQHFFALLGAASAVTTGHPEARKLLDYTIEIIEKYFWSEE
****

150 EQMCLESWDEAFSKTEEYRGGNANMHAVEAFLIVYDVTHDKKWLDRAIRV

200 ASVIIHDVARNNHYRVNEHFDTQWNPLPDYNKDNPAHRFRAFGGTPGHWI

250 EWGRLMLHIHAALEARCEQPPAWLLEDAKGLFNATVRDAWAPDGADGIVY

300 TVDWEGKPVVRERVRWPIVEAMGTAYALYTVTGDRQYETWYQTWWEYCIK

350 YLMDYENGSWWQELDADNKVTTKVWDGKQDIYHLLHCLVIPRIPLAPGMA

400 PAVAAGLLDINAK

sp|P39315|QOR2_ECOLI Quinone oxidoreductase 2 OS=Escherichia coli (strain K12) OX=83333 GN=qorB PE=1 SV=1

0 MIAITGATGQLGHYVIESLMKTVPASQIVAIVRNPAKAQALAAQGITVRQ

50 ADYGDEAALTSALQGVEKLLLISSSEVGQRAPQHRNVINAAKAAGVKFIA

100 YTSLLHADTSPLGLADEHIETEKMLADSGIVYTLLRNGWYSENYLASAPA

150 ALEHGVFIGAAGDGKIASATRADYAAAARVISEAGHEGKVYELAGDSAW
****

200   TLTQLAAELTKQSGKQVTYQNLSEADFAAALKSVGLPDGLADMLADSDVG
      ****
250   ASKGGLFDDSKTLSKLIGHPTTTLAESVSHLFNVNN

sp|P77783|YNFF_ECOLI Probable dimethyl sulfoxide reductase chain YnfF OS=Escherichia coli (strain K12) OX=83333 GN=ynfF PE=1 SV=4

0   MKIHTTEALMKAEISRRSLMKTSALGSLALASSAFTLPFSQMVRAAEAPV

50   EEKAVWSSCTVNCGSRCLLRLHVKDDTVYWVESDTTGDDVYGNHQVRACL

100   RGRSIRRRMNHPDRLKYPMKRVGKRGEGKFERISWDEALDTISDNLRRIL

150   KDYGNEAVHVLYGTGVDGGNITNSNVPYRLMNSCGGFLSRYGSYSTAQIS

200   AAMSYMFGANDGNSPDDIANTKLVVMFGNNPAETRMSGGGVTYYVEQARE

250   RSNARMIVIDPRYNDTAAGREDEWLPIRPGTDGALACAIAWVLITENMVD

300   QPFLDKYCVGYDEKTLPANAPRNAHYKAYILGEGPDGIAKTPEWAAKITS

350   IPAEKIIQLAREIGSAKPAYICQGWGPQRHSNGEQTSRAIAMLSVLTGNV

400   GINGGNSGVREGSWDLGVEWFPMLENPVKTQISVFTWTDAIDHGTEMTAT

450   RDGVRGKEKLDVPIKFLWCYASNTLINQHGDINHTHEVLQDDSKCEMIVG

500   IDHFMTASAKYCDILLPDLMPTEQEDLISHESAGNMGYVILAQPATSAKF

550   ERKPIYWMLSEVAKRLGPDVYQTFTEGRSQHEWIKYLHAKTKERNPEMPD
                                                   ****
600   YEEMKTTGIFKKKCPEEHYVAFRAFREDPQANPLKTPSGKIEIYSERLAK
      ****
650   IADTWELKKDEIIHPLPAYTPGFDGWDDPLRKTYPLQLTGFHYKARTHSS

700   YGNIDVLQQACPQEVWINPIDAQARGIRHGDTVRVFNNNGEMLIAAKVTP

750   RILPGVTAIGQGAWLKADMFGDRVDHGGSINILTSHRPSPLAKGNPSHSN

800   LVQIEKV

sp|P78067|YNJE_ECOLI Thiosulfate sulfurtransferase YnjE OS=Escherichia coli (strain K12) OX=83333 GN=ynjE PE=1 SV=2

0   MKRVSQMTALAMALGLACASSWAAELAKPLTLDQLQQQNGKAIDTRPSAF

50   YNGWPQTLNGPSGHELAALNLSASWLDKMSTEQLNAWIKQHNLKTDAPVA

100   LYGNDKDVDAVKTRLQKAGLTHISILSDALSEPSRLQKLPHFEQLVYPQW
                                                 ****
150   LHDLQQGKEVTAKPAGDWKVIEAAWGAPKLYLISHIPGADYIDTNEVESE
      ****
200   PLWNKVSDEQLKAMLAKHGIRHDTTVILYGRDVYAAARVAQIMLYAGVKD

```
250   VRLLDGGWQTWSDAGLPVERGTPPKVKAEPDFGVKIPAQPQLMLDMEQAR

300   GLLHRQDASLVSIRSWPEFIGTTSGYSYIKPKGEIAGARWGHAGSDSTHM

350   EDFHNPDGTMRSADDITAMWKAWNIKPEQQVSFYCGTGWRASETFMYARA

400   MGWKNVSVYDGGWYEWSSDPKNPVATGERGPDSSK
```

sp|P30863|DKGB_ECOLI 2,5-diketo-D-gluconic acid reductase B OS=Escherichia coli (strain K12) OX=83333 GN=dkgB PE=1 SV=2

```
0     MAIPAFGLGTFRLKDDVVISSVITALELGYRAIDTAQIYDNEAAVGQAIA
                  ****************************
50    ESGVPRHELYITTKIWIENLSKDKLIPSLKESLQKLRTDYVDLTLIHWPS

100   PNDEVSVEEFMQALLEAKKQGLTREIGISNFTIPLMEKAIAAVGAENIAT

150   NQIELSPYLQNRKVVAWAKQHGIHITSYMTLAYGKALKDEVIARIAAKHN

200   ATPAQVILAWAMGEGYSVIPSSTKRKNLESNLKAQNLQLDAEDKKAIAAL

250   DCNDRLVSPEGLAPEWD
```

sp|P0A6Y8|DNAK_ECOLI Chaperone protein DnaK OS=Escherichia coli (strain K12) OX=83333 GN=dnaK PE=1 SV=2

```
0     MGKIIGIDLGTTNSCVAIMDGTTPRVLENAEGDRTTPSIIAYTQDGETLV

50    GQPAKRQAVTNPQNTLFAIKRLIGRRFQDEEVQRDVSIMPFKIIAADNGD

100   AWVEVKGQKMAPPQISAEVLKKMKKTAEDYLGEPVTEAVITVPAYFNDAQ

150   RQATKDAGRIAGLEVKRIINEPTAAALAYGLDKGTGNRTIAVYDLGGGTF

200   DISIIEIDEVDGEKTFEVLATNGDTHLGGEDFDSRLINYLVEEFKKDQGI

250   DLRNDPLAMQRLKEAAEKAKIELSSAQQTDVNLPYITADATGPKHMNIKV

300   TRAKLESLVEDLVNRSIEPLKVALQDAGLSVSDIDDVILVGGQTRMPMVQ

350   KKVAEFFGKEPRKDVNPDEAVAIGAAVQGGVLTGDVKDVLLLDVTPLSLG

400   IETMGGVMTTLIAKNTTIPTKHSQVFSTAEDNQSAVTIHVLQGERKRAAD
                                ************************
450   NKSLGQFNLDGINPAPRGMPQIEVTFDIDADGILHVSAKDKNSGKEQKIT
      **************                                 ***
500   IKASSGLNEDEIQKMVRDAEANAEADRKFEELVQTRNQGDHLLHSTRKQV
      *****************
550   EEAGDKLPADDKTAIESALTALETALKGEDKAAIEAKMQELAQVSQKLME
                   *************************
600   IAQQQHAQQQTAGADASANNAKDDDVVDAEFEEVKDKK
```

sp|P0ABT2|DPS_ECOLI DNA protection during starvation protein OS=Escherichia coli (strain K12) OX=83333 GN=dps PE=1 SV=2

```
             ********************
0     MSTAKLVKSKATNLLYTRNDVSDSEKKATVELLNRQVIQFIDLSLITKQA

50    HWNMRGANFIAVHEMLDGFRTALIDHLDTMAERAVQLGGVALGTTQVINS
                                             *************
100   KTPLKSYPLDIHNVQDHLKELADRYAIVANDVRKAIGEAKDDDTADILTA
      *******
150   ASRDLDKFLWFIESNIE
```

sp|P0A6S0|FLGH_ECOLI Flagellar L-ring protein OS=Escherichia coli (strain K12) OX=83333 GN=flgH PE=3 SV=1

```
0     MQKNAAHTYAISSLLVLSLTGCAWIPSTPLVQGATSAQPVPGPTPVANGS
                                     *******************
50    IFQSAQPINYGYQPLFEDRRPRNIGDTLTIVLQENVSASKSSSANASRDG
      ***
100   KTNFGFDTVPRYLQGLFGNARADVEASGGNTFNGKGGANASNTFSGTLTV

150   TVDQVLVNGNLHVVGEKQIAINQGTEFIRFSGVVNPRTISGSNTVPSTQV

200   ADARIEYVGNGYINEAQNMGWLQRFFLNLSPM
```

sp|P75962|YMFA_ECOLI Inner membrane protein YmfA OS=Escherichia coli (strain K12) OX=83333 GN=ymfA PE=1 SV=2

```
0     MSQDSKVFFRIFLGIGLVLILISVVVFYNQFTYSKDAIHTEGVIVDTVWH
                               *************************
50    SSHSHRTGKDGSWYPVVAFRPTPDYTLIFNSSIGSDFYEDSEGDKVNVYY
      *
100   SPGHPEKAEINNPWVNFFKWGFIGIMGVIFIAVGLLISMPSSKKSRRKRK

150   SRP
```

sp|P0ADZ7|YAJC_ECOLI Sec translocon accessory complex subunit YajC OS=Escherichia coli (strain K12) OX=83333 GN=yajC PE=1 SV=1

```
                                               *********
0     MSFFISDAVAATGAPAQGSPMSLILMLVVFGLIFYFMILRPQQKRTKEHK
      *************
50    KLMDSIAKGDEVLTNGGLVGRVTKVAENGYIAIALNDTTEVVIKRDFVAA

100   VLPKGTMKAL
```

sp|P0DP21|YJIP_ECOLI Putative inactive recombination-promoting nuclease-like protein YjiP OS=Escherichia coli (strain K12) OX=83333 GN=yjiP PE=5 SV=1

```
                                     *******************
0     MTNFTTSTPHDALFKTFLTHPDTARDFMEIHLPKDLRELCDLDSLKLESA
      *************
50    SFVDEKLRALHSDILWSVKTREGDGYIYVVIEHQSREDIHMAFRLMRYSM

100   AVM
```

sp|P27896|NARQ_ECOLI Nitrate/nitrite sensor protein NarQ OS=Escherichia coli (strain K12) OX=83333 GN=narQ PE=1 SV=1

0 MIVKRPVSASLARAFFYIVLLSILSTGIALLTLASSLRDAEAINIAGSLR

50 MQSYRLGYDLQSGSPQLNAHRQLFQQALHSPVLTNLNVWYVPEAVKTRYA

100 HLNANWLEMNNRLSKGDLPWYQANINNYVNQIDLFVLALQHYAERKMLLV

150 VAISLAGGIGIFTLVFFTLRRIRHQVVAPLNQLVTASQRIEHGQFDSPPL
****
200 DTNLPNELGLLAKTFNQMSSELHKLYRSLEASVEEKTRDLHEAKRRLEVL

250 YQCSQALNTSQIDVHCFRHILQIVRDNEAAEYLELNVGENWRISEGQPNP

300 ELPMQILPVTMQETVYGELHWQNSHVSSSEPLLNSVSSMLGRGLYFNQAQ

350 KHFQQLLLMEERATIARELHDSLAQVLSYLRIQLTLLKRSIPEDNATAQS

400 IMADFSQALNDAYRQLRELLTTFRLTLQQADLPSALREMLDTLQNQTSAK

450 LTLDCRLPTLALDAQMQVHLLQIIREAVLNAMKHANASEIAVSCVTAPDG

500 NHTVYIRDNGIGIGEPKEPEGHYGLNIMRERAERLGGTLTFSQPSGGGTL

550 VSISFRSAEGEESQLM

sp|P00350|6PGD_ECOLI 6-phosphogluconate dehydrogenase, decarboxylating OS=Escherichia coli (strain K12) OX=83333 GN=gnd PE=1 SV=2

0 MSKQQIGVVGMAVMGRNLALNIESRGYTVSIFNRSREKTEEVIAENPGKK

50 LVPYYTVKEFVESLETPRRILLMVKAGAGTDAAIDSLKPYLDKGDIIIDG

100 GNTFFQDTIRRNRELSAEGFNFIGTGVSGGEEGALKGPSIMPGGQKEAYE

150 LVAPILTKIAAVAEDGEPCVTYIGADGAGHYVKMVHNGIEYGDMQLIAEA
****
200 YSLLKGGLNLTNEELAQTFTEWNNGELSSYLIDITKDIFTKKDEDGNYLV

250 DVILDEAANKGTGKWTSQSALDLGEPLSLITESVFARYISSLKDQRVAAS

300 KVLSGPQAQPAGDKAEFIEKVRRALYLGKIVSYAQGFSQLRAASEEYNWD

350 LNYGEIAKIFRAGCIIRAQFLQKITDAYAENPQIANLLLAPYFKQIADDY

400 QQALRDVVAYAVQNGIPVPTFSAAVAYYDSYRAAVLPANLIQAQRDYFGA

450 HTYKRIDKEGVFHTEWLD

sp|P39371|NANM_ECOLI N-acetylneuraminate epimerase OS=Escherichia coli (strain K12) OX=83333 GN=nanM PE=1 SV=2

0 MNKTITALAIMMASFAANASVLPETPVPFKSGTGAIDNDTVYIGLGSAGT

50 AWYKLDTQAKDKKWTALAAFPGGPRDQATSAFIDGNLYVFGGIGKNSEGL

100   TQVFNDVHKYNPKTNSWVKLMSHAPMGMAGHVTFVHNGKAYVTGGVNQNI
                  ****

150   FNGYFEDLNEAGKDSTAIDKINAHYFDKKAEDYFFNKFLLSFDPSTQQWS

200   YAGESPWYGTAGAAVVNKGDKTWLINGEAKPGLRTDAVFELDFTGNNLKW

250   NKLAPVSSPDGVAGGFAGISNDSLIFAGGAGFKGSRENYQNGKNYAHEGL

300   KKSYSTDIHLWHNGKWDKSGELSQGRAYGVSLPWNNSLLIIGGETAGGKA

350   VTDSVLITVKDNKVTVQN

sp|P11447|ARLY_ECOLI Argininosuccinate lyase OS=Escherichia coli (strain K12) OX=83333 GN=argH PE=1 SV=3

0   MALWGGRFTQAADQRFKQFNDSLRFDYRLAEQDIVGSVAWSKALVTVGVL
                                 ****

50   TAEEQAQLEEALNVLLEDVRARPQQILESDAEDIHSWVEGKLIDKVGQLG

100   KKLHTGRSRNDQVATDLKLWCKDTVSELLTANRQLQSALVETAQNNQDAV

150   MPGYTHLQRAQPVTFAHWCLAYVEMLARDESRLQDALKRLDVSPLGCGAL

200   AGTAYEIDREQLAGWLGFASATRNSLDSVSDRDHVLELLSAAAIGMVHLS

250   RFAEDLIFFNTGEAGFVELSDRVTSGSSLMPQKKNPDALELIRGKCGRVQ

300   GALTGMMMTLKGLPLAYNKDMQEDKEGLFDALDTWLDCLHMAALVLDGIQ

350   VKRPRCQEAAQQGYANATELADYLVAKGVPFREAHHIVGEAVVEAIRQGK

400   PLEDLPLSELQKFSQVIDEDVYPILSLQSCLDKRAAKGGVSPQQVAQAIA

450   FAQARLG

sp|P0AES0|GSP_ECOLI Bifunctional glutathionylspermidine synthetase/amidase OS=Escherichia coli (strain K12) OX=83333 GN=gss PE=1 SV=1

0   MSKGTTSQDAPFGTLLGYAPGGVAIYSSDYSSLDPQEYEDDAVFRSYIDD

50   EYMGHKWQCVEFARRFLFLNYGVVFTDVGMAWEIFSLRFLREVVNDNILP

100   LQAFPNGSPRAPVAGALLIWDKGGEFKDTGHVAIITQLHGNKVRIAEQNV

150   IHSPLPQGQQWTRELEMVVENGCYTLKDTFDDTTILGWMIQTEDTEYSLP

200   QPEIAGELLKISGARLENKGQFDGKWLDEKDPLQNAYVQANGQVINQDPY

250   HYYTITESAEQELIKATNELHLMYLHATDKVLKDDNLLALFDIPKILWPR

300   LRLSWQRRRHHMITGRMDFCMDERGLKVYEYNADSASCHTEAGLILERWA

```
350   EQGYKGNGFNPAEGLINELAGAWKHSRARPFVHIMQDKDIEENYHAQFME

400   QALHQAGFETRILRGLDELGWDAAGQLIDGEGRLVNCVWKTWAWETAFDQ

450   IREVSDREFAAVPIRTGHPQNEVRLIDVLLRPEVLVFEPLWTVIPGNKAI

500   LPILWSLFPHHRYLLDTDFTVNDELVKTGYAVKPIAGRCGSNIDLVSHHE
                                                        **
550   EVLDKTSGKFAEQKNIYQQLWCLPKVDGKYIQVCTFTVGGNYGGTCLRGD
      *******************
600   ESLVIKKESDIEPLIVVKK
```

sp|P00954|SYW_ECOLI Tryptophan--tRNA ligase OS=Escherichia coli (strain K12) OX=83333 GN=trpS PE=1 SV=3

```
0     MTKPIVFSGAQPSGELTIGNYMGALRQWVNMQDDYHCIYCIVDQHAITVR

50    QDAQKLRKATLDTLALYLACGIDPEKSTIFVQSHVPEHAQLGWALNCYTY

100   FGELSRMTQFKDKSARYAENINAGLFDYPVLMAADILLYQTNLVPVGEDQ
                                    ********************
150   KQHLELSRDIAQRFNALYGEIFKVPEPFIPKSGARVMSLLEPTKKMSKSD
      *       ***********************
200   DNRNNVIGLLEDPKSVVKKIKRAVTDSDEPPVVRYDVQNKAGVSNLLDIL

250   SAVTGQSIPELEKQFEGKMYGHLKGEVADAVSGMLTELQERYHRFRNDEA

300   FLQQVMKDGAEKASAHASRTLKAVYEAIGFVAKP
```

sp|P06993|MALT_ECOLI HTH-type transcriptional regulator MalT OS=Escherichia coli (strain K12) OX=83333 GN=malT PE=1 SV=2

```
0     MLIPSKLSRPVRLDHTVVRERLLAKLSGANNFRLALITSPAGYGKTTLIS

50    QWAAGKNDIGWYSLDEGDNQQERFASYLIAAVQQATNGHCAICETMAQKR

100   QYASLTSLFAQLFIELAEWHSPLYLVIDDYHLITNPVIHESMRFFIRHQP

150   ENLTLVVLSRNLPQLGIANLRVRDQLLEIGSQQLAFTHQEAKQFFDCRLS

200   SPIEAAESSRICDDVSGWATALQLIALSARQNTHSAHKSARRLAGINASH

250   LSDYLVDEVLDNVDLATRHFLLKSAILRSMNDALITRVTGEENGQMRLEE

300   IERQGLFLQRMDDTGEWFCYHPLFGNFLRQRCQWELAAELPEIHRAAAES
                                             *************
350   WMAQGFPSEAIHHALAAGDALMLRDILLNHAWSLFNHSELSLLEESLKAL
      *******
400   PWDSLLENPQLVLLQAWLMQSQHRYGEVNTLLARAEHEIKDIREDTMHAE

450   FNALRAQVAINDGNPDEAERLAKLALEELPPGWFYSRIVATSVLGEVLHC

500   KGELTRSLALMQQTEQMARQHDVWHYALWSLIQQSEILFAQGFLQTAWET
```

550 QEKAFQLINEQHLEQLPMHEFLVRIRAQLLWAWARLDEAEASARSGIEVL

600 SSYQPQQQLQCLAMLIQCSLARGDLDNARSQLNRLENLLGNGKYHSDWIS

650 NANKVRVIYWQMTGDKAAAANWLRHTAKPEFANNHFLQGQWRNIARAQIL

700 LGEFEPAEIVLEELNENARSLRLMSDLNRNLLLLNQLYWQAGRKSDAQRV

750 LLDALKLANRTGFISHFVIEGEAMAQQLRQLIQLNTLPELEQHRAQRILR

800 EINQHHRHKFAHFDENFVERLLNHPEVPELIRTSPLTQREWQVLGLIYSG

850 YSNEQIAGELEVAATTIKTHIRNLYQKLGVAHRQDAVQHAQQLLKMMGYG

900 V

sp|P26616|MAO1_ECOLI NAD-dependent malic enzyme OS=Escherichia coli (strain K12) OX=83333 GN=maeA PE=1 SV=4

0 MEPKTKKQRSLYIPYAGPVLLEFPLLNKGSAFSMEERRNFNLLGLLPEVV

50 ETIEEQAERAWIQYQGFKTEIDKHIYLRNIQDTNETLFYRLVNNHLDEMM

100 PVIYTPTVGAACERFSEIYRRSRGVFISYQNRHNMDDILQNVPNHNIKVI

150 VVTDGERILGLGDQGIGGMGIPIGKLSLYTACGGISPAYTLPVVLDVGTN

200 NQQLLNDPLYMGWRNPRITDDEYYEFVDEFIQAVKQRWPDVLLQFEDFAQ

250 KNAMPLLNRYRNEICSFNDDIQGTAAVTVGTLIAASRAAGGQLSEKKIVF

300 LGAGSAGCGIAEMIISQTQREGLSEEAARQKVFMVDRFGLLTDKMPNLLP
****
350 FQTKLVQKRENLSDWDTDSDVLSLLDVVRNVKPDILIGVSGQTGLFTEEI

400 IREMHKHCPRPIVMPLSNPTSRVEATPQDIIAWTEGNALVATGSPFNPVV

450 WKDKIYPIAQCNNAFIFPGIGLGVIASGASRITDEMLMSASETLAQYSPL

500 VLNGEGMVLPELKDIQKVSRAIAFAVGKMAQQQGVAVKTSAEALQQAIDD

550 NFWQAEYRDYRRTSI

sp|Q46790|PBL_ECOLI Putative peptidoglycan-binding-like protein OS=Escherichia coli (strain K12) OX=83333 GN=pbl PE=5 SV=2
*
0 MRSPKVKFLTIFTFCIFITKMSFASNSCSNEAGTMFRIEPNLIKAIALVE
****
50 SNLKKDSIGKNRDKNNNIKSLDYWLMQINQMHIPLLKKRGIIKDERDLLD

100 NPCLNIKIGTEILYNHFSRCGVTWQCLGTYNAGFAMDNQKKRQQYAPKYI

150 LYIPGLMN

```
sp|P37056|YAEF_ECOLI Probable lipoprotein peptidase YaeF OS=Escherichia
coli (strain K12) OX=83333 GN=yaeF PE=3 SV=3

0     MDKPKAYCRLFLPSFLLLSACTVDISQPDPSATAVDAEAKTWAVKFQHQS

50    SFTEQSIKEITAPDLKPGDLLFSSSLGVTSFGIRVFSTSSVSHVAIFLGD

100   NNVAEATGAGVQIVSLKKAMKHSDKLFVLRVPDLTPQQATDITAFANKIK
                                             ***********
150   DSGYNYRGIVEFIPFMVTRQMCSLNPFSEDFRQQCVSGLAKAQLSSVGEG
      **************
200   DKKSWFCSEFVTDAFAKAGHPLTLAQSGWISPADLMHMRIGDVSAFKPET

250   QLQYVGHLKPGIYIKAGRFVGLTR

sp|P02942|MCP1_ECOLI Methyl-accepting chemotaxis protein I OS=Escherichia
coli (strain K12) OX=83333 GN=tsr PE=1 SV=2

0     MLKRIKIVTSLLLVLAVFGLLQLTSGGLFFNALKNDKENFTVLQTIRQQQ

50    STLNGSWVALLQTRNTLNRAGIRYMMDQNNIGSGSTVAELMESASISLKQ

100   AEKNWADYEALPRDPRQSTAAAAEIKRNYDIYHNALAELIQLLGAGKINE

150   FFDQPTQGYQDGFEKQYVAYMEQNDRLHDIAVSDNNASYSQAMWILVGVM

200   IVVLAVIFAVWFGIKASLVAPMNRLIDSIRHIAGGDLVKPIEVDGSNEMG

250   QLAESLRHMQGELMRTVGDVRNGANAIYSGASEIATGNNDLSSRTEQQAA

300   SLEETAASMEQLTATVKQNAENARQASHLALSASETAQRGGKVVDNVVQT

350   MRDISTSSQKIADIISVIDGIAFQTNILALNAAVEAARAGEQGRGFAVVA
                       ********************
400   GEVRNLAQRSAQAAREIKSLIEDSVGKVDVGSTLVESAGETMAEIVSAVT

450   RVTDIMGEIASASDEQSRGIDQVGLAVAEMDRVTQQNAALVEESAAAAAA

500   LEEQASRLTEAVAVFRIQQQQRETSAVVKTVTPAAPRKMAVADSEENWET

550   F

sp|P10443|DPO3A_ECOLI DNA polymerase III subunit alpha OS=Escherichia
coli (strain K12) OX=83333 GN=dnaE PE=1 SV=1

0     MSEPRFVHLRVHSDYSMIDGLAKTAPLVKKAAALGMPALAITDFTNLCGL

50    VKFYGAGHGAGIKPIVGADFNVQCDLLGDELTHLTVLAANNTGYQNLTLL

100   ISKAYQRGYGAAGPIIDRDWLIELNEGLILLSGGRMGDVGRSLLRGNSAL

150   VDECVAFYEEHFPDRYFLELIRTGRPDEESYLHAAVELAEARGLPVVATN

200   DVRFIDSSDFDAHEIRVAIHDGFTLDDPKRPRNYSPQQYMRSEEEMCELF
```

250   ADIPEALANTVEIAKRCNVTVRLGEYFLPQFPTGDMSTEDYLVKRAKEGL
        ****

300   EERLAFLFPDEEERLKRRPEYDERLETELQVINQMGFPGYFLIVMEFIQW

350   SKDNGVPVGPGRGSGAGSLVAYALKITDLDPLEFDLLFERFLNPERVSMP

400   DFDVDFCMEKRDQVIEHVADMYGRDAVSQIITFGTMAAKAVIRDVGRVLG

450   HPYGFVDRISKLIPPDPGMTLAKAFEAEPQLPEIYEADEEVKALIDMARK

500   LEGVTRNAGKHAGGVVIAPTKITDFAPLYCDEEGKHPVTQFDKSDVEYAG

550   LVKFDFLGLRTLTIINWALEMINKRRAKNGEPPLDIAAIPLDDKKSFDML

600   QRSETTAVFQLESRGMKDLIKRLQPDCFEDMIALVALFRPGPLQSGMVDN

650   FIDRKHGREEISYPDVQWQHESLKPVLEPTYGIILYQEQVMQIAQVLSGY
             ****

700   TLGGADMLRRAMGKKKPEEMAKQRSVFAEGAEKNGINAELAMKIFDLVEK

750   FAGYGFNKSHSAAYALVSYQTLWLKAHYPAEFMAAVMTADMDNTEKVVGL

800   VDECWRMGLKILPPDINSGLYHFHVNDDGEIVYGIGAIKGVGEGPIEAII

850   EARNKGGYFRELFDLCARTDTKKLNRRVLEKLIMSGAFDRLGPHRAALMN

900   SLGDALKAADQHAKAEAIGQADMFGVLAEEPEQIEQSYASCQPWPEQVVL

950   DGERETLGLYLTGHPINQYLKEIERYVGGVRLKDMHPTERGKVITAAGLV

1000 VAARVMVTKRGNRIGICTLDDRSGRLEVMLFTDALDKYQQLLEKDRILIV

1050 SGQVSFDDFSGGLKMTAREVMDIDEAREKYARGLAISLTDRQIDDQLLNR

1100 LRQSLEPHRSGTIPVHLYYQRADARARLRFGATWRVSPSDRLLNDLRGLI

1150 GSEQVELEFD

sp|P19317|NARW_ECOLI Probable nitrate reductase molybdenum cofactor assembly chaperone NarW OS=Escherichia coli (strain K12) OX=83333 GN=narW PE=1 SV=1

0   MQILKVIGLLMEYPDELLWECKEDALALIRRDAPMLTDFTHNLLNAPLLD

50   KQAEWCEVFDRGRTTSLLLFEHVHAESRDRGQAMVDLLAEYEKVGLQLDC

100   RELPDYLPLYLEYLSVLPDDQAKEGLLNVAPILALLGGRLKQREAPWYAL
      ****

150   FDALLQLAGSSLSSDSVTKQVNSEERDDTRQALDAVWEEEQVKFIEDNAT

200   ACDSSPLNQYQRRFSQDVAPQYVDISAGGGK

sp|Q46796|YGEP_ECOLI Putative protein YgeP OS=Escherichia coli (strain K12) OX=83333 GN=ygeP PE=5 SV=1

```
        ********************************
0     MTSGLERLSNLLSKKDSVFVSDLLREAKVNELDETLSTTRLNHLIDKGYE

50    RITLQLDLGGESPGYLEKDKHYREADAALLNVIYPTNLSKINTRRKEQV
```

sp|P76612|YPJB_ECOLI Protein YpjB OS=Escherichia coli (strain K12) OX=83333 GN=ypjB PE=4 SV=1

```
      ***************************************
0     MESRNSYENKIDEISSLSESKEHPIDIQEKKDAFVNEFKGVLFDKNTRSS

50    ELLFNFYECCYKFLPRAQPQDKIDSYNSALQAFSIFCSSTLTHNNIGFDF

100   KLFPEVKLSGEHLETVFKYKNGDDVREIAKINITLQKEEGGLYNLRGLDF

150   KGCFFSGQNFSNYDIQYVNWGTSLFDVDTPCIFNAPAYNKSNEKSLKPVS
                                             ***********
200   ENGLSGVLTDRNNKIKLITGVAPFDDILFMDDDFDDSSSEDDPVENSPVV
      ***********
250   TSPVVSSSKSSFQ
```

sp|P33666|YDBA_ECOLI Exported protein YdbA OS=Escherichia coli (strain K12) OX=83333 GN=ydbA PE=3 SV=3

```
0     MQRKTLLSACIALALSGQGWAADITEVETTTGEKKNTNVTCPADPGKLSP

50    EELKRLPSECSPLVEQNLMPWLSTGAAALITALAVVELNDDDDHHHRNNS

100   PLPPTPPDDESDDTPVPPTPGGDEIIPDDPDDTPTPPKPVSFNNDVILDK

150   TEKTLTIRDSVFTYTENADGTISLQDSNGRKATINLWQIDEANNTVALEG

200   VSADGATKWQYNHNGELVITGDNATVNNNGKTTVDGKDSTGTEINGNNGK

250   VIQDGDLDVSGGGHGIDITGDSATVDNKGTMTVTDPESMGIQIDGDKAIV

300   NNEGESTITNGGTGTQINGDDATANNNGKTTVDGKDSTGTEINGNNGKVI

350   QDGDLDVSGGGHGIDITGDSATVDNKGTMTVTDPESIGIQVDGDQAVVNN

400   EGESAITNGGTGTQINGDDATANNNGKTTVDGKDSTGTEIAGNNGKVIQD

450   GDLDVSGGGHGIDITGDSATVDNKGTMTVTDPESIGIQIDGDQAIVNNEG

500   ESTITNGGTGTQINGNDATANNSGKTTVDGKDSTGTKIAGNIGIVNLDGS

550   LTVTGGAHGVENIGDNGTVNNKGDIVVSDTGSIGVLINGEGATVSNTGDV

600   NVSNEATGFSITTNSGKVSLAGSMQVGDFSTGVDLNGNNNSVTLAAKDLK

650   VVGQKATGINVSGDANTVNITGNVLVDKDKTADNAAEYFFDPSVGINVYG
                   **********************
700   SDNNVTLDGKLTVVSDSEVTSRQSNLFDGSAEKTSGLVVIGDGNTVNMNG
```

```
750   GLELIGEKNALADGSQVTSLRTGYSYTSVIVVSGESSVYLNGDTTISGEF

800   PLGFAGVIRVQDKALLEIGSGATLTMQDIDSFEHHGTRTLDLPLYFQTSV

850   IT
```

sp|P21507|SRMB_ECOLI ATP-dependent RNA helicase SrmB OS=Escherichia coli (strain K12) OX=83333 GN=srmB PE=1 SV=1

```
0     MTVTTFSELELDESLLEALQDKGFTRPTAIQAAAIPPALDGRDVLGSAPT

50    GTGKTAAYLLPALQHLLDFPRKKSGPPRILILTPTRELAMQVSDHARELA

100   KHTHLDIATITGGVAYMNHAEVFSENQDIVVATTGRLLQYIKEENFDCRA

150   VETLILDEADRMLDMGFAQDIEHIAGETRWRKQTLLFSATLEGDAIQDFA

200   ERLLEDPVEVSANPSTRERKKIHQWYYRADDLEHKTALLVHLLKQPEATR

250   SIVFVRKRERVHELANWLREAGINNCYLEGEMVQGKRNEAIKRLTEGRVN

300   VLVATDVAARGIDIPDVSHVFNFDMPRSGDTYLHRIGRTARAGRKGTAIS
                                         ***************
350   LVEAHDHLLLGKVGRYIEEPIKARVIDELRPKTRAPSEKQTGKPSKKVLA
      ****************************
400   KRAEKKKAKEKEKPRVKKRHRDTKNIGKRRKPSGTGVPPQTTEE
```

sp|P0A7A9|IPYR_ECOLI Inorganic pyrophosphatase OS=Escherichia coli (strain K12) OX=83333 GN=ppa PE=1 SV=2

```
0     MSLLNVPAGKDLPEDIYVVIEIPANADPIKYEIDKESGALFVDRFMSTAM
                                                     ***
50    FYPCNYGYINHTLSLDGDPVDVLVPTPYPLQPGSVIRCRPVGVLKMTDEA
      ********************************
100   GEDAKLVAVPHSKLSKEYDHIKDVNDLPELLKAQIAHFFEHYKDLEKGKW

150   VKVEGWENAEAAKAEIVASFERAKNK
```

sp|P75733|CHIP_ECOLI Chitoporin OS=Escherichia coli (strain K12) OX=83333 GN=chiP PE=1 SV=1

```
0     MRTFSGKRSTLALAIAGVTAMSGFMAMPEARAEGFIDDSTLTGGIYYWQR
      ********************
50    ERDRKDVTDGDKYKTNLSHSTWNANLDFQSGYAADMFGLDIAAFTAIEMA
        ***************************************
100   ENGDSSHPNEIAFSKSNKAYDEDWSGDKSGISLYKAAAKFKYGPVWARAG

150   YIQPTGQTLLAPHWSFMPGTYQGAEAGANFDYGDAGALSFSYMWTNEYKA

200   PWHLEMDEFYQNDKTTKVDYLHSFGAKYDFKNNFVLEAAFGQAEGYIDQY

250   FAKASYKFDIAGSPLTTSYQFYGTRDKVDDRSVNDLYDGTAWLQALTFGY
```

```
300   RAADVVDLRLEGTWVKADGQQGYFLQRMTPTYASSNGRLDIWWDNRSDFN

350   ANGEKAVFFGAMYDLKNWNLPGFAIGASYVYAWDAKPATWQSNPDAYYDK

400   NRTIEESAYSLDAVYTIQDGRAKGTMFKLHFTEYDNHSDIPSWGGGYGNI

450   FQDERDVKFMVIAPFTIF

sp|P52643|LDHD_ECOLI D-lactate dehydrogenase OS=Escherichia coli (strain
K12) OX=83333 GN=ldhA PE=1 SV=1

0     MKLAVYSTKQYDKKYLQQVNESFGFELEFFDFLLTEKTAKTANGCEAVCI

50    FVNDDGSRPVLEELKKHGVKYIALRCAGFNNVDLDAAKELGLKVVRVPAY

100   DPEAVAEHAIGMMMTLNRRIHRAYQRTRDANFSLEGLTGFTMYGKTAGVI

150   GTGKIGVAMLRILKGFGMRLLAFDPYPSAAALELGVEYVDLPTLFSESDV

200   ISLHCPLTPENYHLLNEAAFEQMKNGVMIVNTSRGALIDSQAAIEALKNQ
               *********************
250   KIGSLGMDVYENERDLFFEDKSNDVIQDDVFRRLSACHNVLFTGHQAFLT

300   AEALTSISQTTLQNLSNLEKGETCPNELV

sp|P39384|YJIM_ECOLI Putative dehydratase subunit YjiM OS=Escherichia
coli (strain K12) OX=83333 GN=yjiM PE=3 SV=2

0     MSLVTDLPAIFDQFSEARQTGFLTVMDLKERGIPLVGTYCTFMPQEIPMA
        ************************
50    AGAVVVSLCSTSDETIEEAEKDLPRNLCPLIKSSYGFGKTDKCPYFYFSD

100   LVVGETTCDGKKKMYEYMAEFKPVHVMQLPNSVKDDASRALWKAEMLRLQ

150   KTVEERFGHEISEDALRDAIALKNRERRALANFYHLGQLNPPALSGSDIL

200   KVVYGATFRFDKEALINELDAMTARVRQQWEEGQRLDPRPRILITGCPIG

250   GAAEKVVRAIEENGGWVVGYENCTGAKATEQCVAETGDVYDALADKYLAI

300   GCSCVSPNDQRLKMLSQMVEEYQVDGVVDVILQACHTYAVESLAIKRHVR

350   QQHNIPYIAIETDYSTSDVGQLSTRVAAFIEML

sp|P0A817|METK_ECOLI S-adenosylmethionine synthase OS=Escherichia coli
(strain K12) OX=83333 GN=metK PE=1 SV=2
           **********************
0     MAKHLFTSESVSEGHPDKIADQISDAVLDAILEQDPKARVACETYVKTGM

50    VLVGGEITTSAWVDIEEITRNTVREIGYVHSDMGFDANSCAVLSAIGKQS

100   PDINQGVDRADPLEQGAGDQGLMFGYATNETDVLMPAPITYAHRLVQRQA
                                          **************
150   EVRKNGTLPWLRPDAKSQVTFQYDDGKIVGIDAVVLSTQHSEEIDQKSLQ
```

```
      **********
200   EAVMEEIIKPILPAEWLTSATKFFINPTGRFVIGGPMGDCGLTGRKIIVD

250   TYGGMARHGGGAFSGKDPSKVDRSAAYAARYVAKNIVAAGLADRCEIQVS

300   YAIGVAEPTSIMVETFGTEKVPSEQLTLLVREFFDLRPYGLIQMLDLLHP

350   IYKETAAYGHFGREHFPWEKTDKAQLLRDAAGLK
```

sp|P0AB43|YCGL_ECOLI Protein YcgL OS=Escherichia coli (strain K12) OX=83333 GN=ycgL PE=1 SV=3

```
                      **********************
0     MPKPGILKSKSMFCVIYRSSKRDQTYLYVEKKDDFSRVPEELMKGFGQPQ

50    LAMILPLDGRKKLVNADIEKVKQALTEQGYYLQLPPPPEDLLKQHLSVMG

100   QKTDDTNK
```

sp|P77510|DPIB_ECOLI Sensor histidine kinase DpiB OS=Escherichia coli (strain K12) OX=83333 GN=dpiB PE=1 SV=1

```
0     MLQLNENKQFAFFQRLAFPLRIFLLILVFSIFVIAALAQYFTASFEDYLT

50    LHVRDMAMNQAKIIASNDSVISAVKTRDYKRLATIANKLQRDTDFDYVVI

100   GDRHSIRLYHPNPEKIGYPMQFTKQGALEKGESYFITGKGSMGMAMRAKT

150   PIFDDDGKVIGVVSIGYLVSKIDSWRAEFLLPMAGVFVVLLGILMLLSWF

200   LAAHIRRQMMGMEPKQIARVVRQQEALFSSVYEGLIAVDPHGYITAINRN

250   ARKMLGLSSPGRQWLGKPIVEVVRPADFFTEQIDEKRQDVVANFNGLSVI

300   ANREAIRSGDDLLGAIISFRSKDEISTLNAQLTQIKQYVESLRTLRHEHL

350   NWMSTLNGLLQMKEYDRVLAMVQGESQAQQQLIDSLREAFADRQVAGLLF
                                                 *****
400   GKVQRARELGLKMIIVPGSQLSQLPPGLDSTEFAAIVGNLLDNAFEASLR
      ********************
450   SDEGNKIVELFLSDEGDDVVIEVADQGCGVPESLRDKIFEQGVSTRADEP

500   GEHGIGLYLIASYVTRCGGVITLEDNDPCGTLFSIYIPKVKPNDSSINPI

550   DR
```

sp|P0A6M8|EFG_ECOLI Elongation factor G OS=Escherichia coli (strain K12) OX=83333 GN=fusA PE=1 SV=2

```
0     MARTTPIARYRNIGISAHIDAGKTTTTERILFYTGVNHKIGEVHDGAATM

50    DWMEQEQERGITITSAATTAFWSGMAKQYEPHRINIIDTPGHVDFTIEVE

100   RSMRVLDGAVMVYCAVGGVQPQSETVWRQANKYKVPRIAFVNKMDRMGAN
```

```
150   FLKVVNQIKTRLGANPVPLQLAIGAEEHFTGVVDLVKMKAINWNDADQGV
                            ***************************
200   TFEYEDIPADMVELANEWHQNLIESAAEASEELMEKYLGGEELTEAEIKG
                                                      **
250   ALRQRVLNNEIILVTCGSAFKNKGVQAMLDAVIDYLPSPVDVPAINGILD
      *******************
300   DGKDTPAERHASDDEPFSALAFKIATDPFVGNLTFFRVYSGVVNSGDTVL

350   NSVKAARERFGRIVQMHANKREEIKEVRAGDIAAAIGLKDVTTGDTLCDP

400   DAPIILERMEFPEPVISIAVEPKTKADQEKMGLALGRLAKEDPSFRVWTD

450   EESNQTIIAGMGELHLDIIVDRMKREFNVEANVGKPQVAYRETIRQKVTD

500   VEGKHAKQSGGRGQYGHVVIDMYPLEPGSNPKGYEFINDIKGGVIPGEYI

550   PAVDKGIQEQLKAGPLAGYPVVDMGIRLHFGSYHDVDSSELAFKLAASIA

600   FKEGFKKAKPVLLEPIMKVEVETPEENTGDVIGDLSRRRGMLKGQESEVT

650   GVKIHAEVPLSEMFGYATQLRSLTKGRASYTMEFLKYDEAPSNVAQAVIE

700   ARGK
```

sp|P39265|ALSB_ECOLI D-allose-binding periplasmic protein OS=Escherichia coli (strain K12) OX=83333 GN=alsB PE=1 SV=1

```
0     MNKYLKYFSGTLVGLMLSTSAFAAAEYAVVLKTLSNPFWVDMKKGIEDEA

50    KTLGVSVDIFASPSEGDFQSQLQLFEDLSNKNYKGIAFAPLSSVNLVMPV

100   ARAWKKGIYLVNLDEKIDMDNLKKAGGNVEAFVTTDNVAVGAKGASFIID

150   KLGAEGGEVAIIEGKAGNASGEARRNGATEAFKKASQIKLVASQPADWDR

200   IKALDVATNVLQRNPNIKAIYCANDTMAMGVAQAVANAGKTGKVLVVGTD
                                                  **************
250   GIPEARKMVEAGQMTATVAQNPADIGATGLKLMVDAEKSGKVIPLDKAPE
      ******
300   FKLVDSILVTQ
```

sp|P28722|YHCA_ECOLI Uncharacterized fimbrial chaperone YhcA OS=Escherichia coli (strain K12) OX=83333 GN=yhcA PE=3 SV=2

```
0     MLRHITFTVFITTSMNTLATGMVPETSVLLVDEKRGEASINIKNTDDHPS
                                                   *****
50    LLYTTIVDLPESNKSIRLIPTQPVIRVEAGQVQQVRFLLQATVPLQSEEL
      *****************
100   KRVTFEGIPPKDDKSSRVTVSIRQDLPVLIHPASLPEERETWKFLEWRKN

150   GDQIEISNPSNYVVRMTLQFKTLPSGKTGAINKTYFLPHTSTTTALTNAT

200   DTKVEFYPASRYGYRGNKYVTDLK
```

sp|P76513|YFDQ_ECOLI Uncharacterized protein YfdQ OS=Escherichia coli (strain K12) OX=83333 GN=yfdQ PE=4 SV=1

```
0     MSQNLDATAINQIHALISAQGVNEIISKIGADAVALPENFRIHDLEKFNL

50    NRFRFRGALSTASIDDFTRYSKDLADEGTRCFIDADNMRAVSVLNLGTID

100   EPGHADNTATLKLKKTAPFSALLSVNGERNSQKSLAEWIEDWADYLVGFD
                              **************************
150   ANGDAIQATKAAAAIRKITIEANQTADFEDNDFSGKRSLMESVEAKTKDI
      *                                           ******
200   MPVAFEFKCVPFEGLKERPFKLRLSIITGDRPVLVLRIIQLEAVQEDMAN
      ****************
250   EFRDLLVEKFKDSKVETFIGTFTA
```

sp|P75797|GSIB_ECOLI Glutathione-binding protein GsiB OS=Escherichia coli (strain K12) OX=83333 GN=gsiB PE=1 SV=1

```
0     MARAVHRSGLVALGIATALMASCAFAAKDVVVAVGSNFTTLDPYDANDTL
                    *********************
50    SQAVAKSFYQGLFGLDKEMKLKNVLAESYTVSDDGITYTVKLREGIKFQD

100   GTDFNAAAVKANLDRASDPANHLKRYNLYKNIAKTEAIDPTTVKITLKQP

150   FSAFINILAHPATAMISPAALEKYGKEIGFYPVGTGPYELDTWNQTDFVK

200   VKKFAGYWQPGLPKLDSITWRPVADNNTRAAMLQTGEAQFAFPIPYEQAT

250   LLEKNKNIELMASPSIMQRYISMNVTQKPFDNPKVREALNYAINRPALVK

300   VAFAGYATPATGVVPPSIAYAQSYKPWPYDPVKARELLKEAGYPNGFSTT

350   LWSSHNHSTAQKVLQFTQQQLAQVGIKAQVTAMDAGQRAAEVEGKGQKES

400   GVRMFYTGWSASTGEADWALSPLFASQNWPPTLFNTAFYSNKQVDDFLAQ

450   ALKTNDPAEKTRLYKAAQDIIWQESPWIPLVVEKLVSAHSKNLTGFWIMP

500   DTGFSFEDADLQ
```

sp|P37650|BCSC_ECOLI Cellulose synthase operon protein C OS=Escherichia coli (strain K12) OX=83333 GN=bcsC PE=1 SV=3

```
0     MRKFTLNIFTLSLGLAVMPMVEAAPTAQQQLLEQVRLGEATHREDLVQQS

50    LYRLELIDPNNPDVVAARFRSLLRQGDIDGAQKQLDRLSQLAPSSNAYKS

100   SRTTMLLSTPDGRQALQQARLQATTGHAEEAVASYNKLFNGAPPEGDIAV

150   EYWSTVAKIPARRGEAINQLKRINADAPGNTGLQNNLALLLFSSDRRDEG
                                   *********************
200   FAVLEQMAKSNAGREGASKIWYGQIKDMPVSDASVSALKKYLSIFSDGDS
      *
250   VAAAQSQLAEQQKQLADPAFRARAQGLAAVDSGMAGKAIPELQQAVRANP
```

```
300   KDSEALGALGQAYSQKGDRANAVANLEKALALDPHSSNNDKWNSLLKVNR

350   YWLAIQQGDAALKANNPDRAERLFQQARNVDNTDSYAVLGLGDVAMARKD

400   YPAAERYYQQTLRMDSGNTNAVRGLANIYRQQSPEKAEAFIASLSASQRR

450   SIDDIERSLQNDRLAQQAEALENQGKWAQAAALQRQRLALDPGSVWITYR

500   LSQDLWQAGQRSQADTLMRNLAQQKSNDPEQVYAYGLYLSGHDQDRAALA

550   HINSLPRAQWNSNIQELVNRLQSDQVLETANRLRESGKEAEAEAMLRQQP

600   PSTRIDLTLADWAQQRRDYTAARAAYQNVLTREPANADAILGLTEVDIAA

650   GDKAAARSQLAKLPATDNASLNTQRRVALAQAQLGDTAAAQRTFNKLIPQ

700   AKSQPPSMESAMVLRDGAKFEAQAGDPTQALETYKDAMVASGVTTTRPQD

750   NDTFTRLTRNDEKDDWLKRGVRSDAADLYRQQDLNVTLEHDYWGSSGTGG

800   YSDLKAHTTMLQVDAPYSDGRMFFRSDFVNMNVGSFSTNADGKWDDNWGT

850   CTLQDCSGNRSQSDSGASVAVGWRNDVWSWDIGTTPMGFNVVDVVGGISY

900   SDDIGPLGYTVNAHRRPISSSLLAFGGQKDSPSNTGKKWGGVRADGVGLS

950   LSYDKGEANGVWASLSGDQLTGKNVEDNWRVRWMTGYYYKVINQNNRRVT

1000  IGLNNMIWHYDKDLSGYSLGQGGYYSPQEYLSFAIPVMWRERTENWSWEL

1050  GASGSWSHSRTKTMPRYPLMNLIPTDWQEEAARQSNDGGSSQGFGYTARA

1100  LLERRVTSNWFVGTAIDIQQAKDYAPSHFLLYVRYSAAGWQGDMDLPPQP

1150  LIPYADW
```

sp|P36767|RDGC_ECOLI Recombination-associated protein RdgC OS=Escherichia coli (strain K12) OX=83333 GN=rdgC PE=1 SV=1

```
0     MLWFKNLMVYRLSREISLRAEEMEKQLASMAFTPCGSQDMAKMGWVPPMG
                                        ****************
50    SHSDALTHVANGQIVICARKEEKILPSPVIKQALEAKIAKLEAEQARKLK
      *******************
100   KTEKDSLKDEVLHSLLPRAFSRFSQTMMWIDTVNGLIMVDCASAKKAEDT

150   LALLRKSLGSLPVVPLSMENPIELTLTEWVRSGSAAQGFQLLDEAELKSL
                                                   *****
200   LEDGGVIRAKKQDLTSEEITNHIEAGKVVTKLALDWQQRIQFVMCDDGSL
      ********************
250   KRLKFCDELRDQNEDIDREDFAQRFDADFILMTGELAALIQNLIEGLGGE

300   AQR
```

sp|P0A9H3|LDCI_ECOLI Inducible lysine decarboxylase OS=Escherichia coli (strain K12) OX=83333 GN=cadA PE=1 SV=1

0 MNVIAILNHMGVYFKEEPIRELHRALERLNFQIVYPNDRDDLLKLIENNA

50 RLCGVIFDWDKYNLELCEEISKMNENLPLYAFANTYSTLDVSLNDLRLQI

100 SFFEYALGAAEDIANKIKQTTDEYINTILPPLTKALFKYVREGKYTFCTP
****

150 GHMGGTAFQKSPVGSLFYDFFGPNTMKSDISISVSELGSLLDHSGPHKEA

200 EQYIARVFNADRSYMVTNGTSTANKIVGMYSAPAGSTILIDRNCHKSLTH

250 LMMMSDVTPIYFRPTRNAYGILGGIPQSEFQHATIAKRVKETPNATWPVH

300 AVITNSTYDGLLYNTDFIKKTLDVKSIHFDSAWVPYTNFSPIYEGKCGMS

350 GGRVEGKVIYETQSTHKLLAAFSQASMIHVKGDVNEETFNEAYMMHTTTS

400 PHYGIVASTETAAAMMKGNAGKRLINGSIERAIKFRKEIKRLRTESDGWF

450 FDVWQPDHIDTTECWPLRSDSTWHGFKNIDNEHMYLDPIKVTLLTPGMEK

500 DGTMSDFGIPASIVAKYLDEHGIVVEKTGPYNLLFLFSIGIDKTKALSLL

550 RALTDFKRAFDLNLRVKNMLPSLYREDPEFYENMRIQELAQNIHKLIVHH

600 NLPDLMYRAFEVLPTMVMTPYAAFQKELHGMTEEVYLDEMVGRINANMIL

650 PYPPGVPLVMPGEMITEESRPVLEFLQMLCEIGAHYPGFETDIHGAYRQA

700 DGRYTVKVLKEESKK

sp|P11988|BGLB_ECOLI 6-phospho-beta-glucosidase BglB OS=Escherichia coli (strain K12) OX=83333 GN=bglB PE=1 SV=2

0 MKAFPETFLWGGATAANQVEGAWQEDGKGISTSDLQPHGVMGKMEPRILG

50 KENIKDVAIDFYHRYPEDIALFAEMGFTCLRISIAWARIFPQGDEVEPNE

100 AGLAFYDRLFDEMAQAGIKPLVTLSHYEMPYGLVKNYGGWANRAVIDHFE

150 HYARTVFTRYQHKVALWLTFNEINMSLHAPFTGVGLAEESGEAEVYQAIH

200 HQLVASARAVKACHSLLPEAKIGNMLLGGLVYPLTCQPQDMLQAMEENRR
****

250 WMFFGDVQARGQYPGYMQRFFRDHNITIEMTESDAEDLKHTVDFISFSYY

300 MTGCVSHDESINKNAQGNILNMIPNPHLKSSEWGWQIDPVGLRVLLNTLW

350 DRYQKPLFIVENGLGAKDSVEADGSIQDDYRIAYLNDHLVQVNEAIADGV

400 DIMGYTSWGPIDLVSASHSQMSKRYGFIYVDRDDNGEGSLTRTRKKSFGW

450   YAEVIKTRGLSLKKITIKAP

sp|P0A998|FTNA_ECOLI Bacterial non-heme ferritin OS=Escherichia coli (strain K12) OX=83333 GN=ftnA PE=1 SV=1

0   MLKPEMIEKLNEQMNLELYSSLLYQQMSAWCSYHTFEGAAAFLRRHAQEE

50   MTHMQRLFDYLTDTGNLPRINTVESPFAEYSSLDELFQETYKHEQLITQK
                          ****
100   INELAHAAMTNQDYPTFNFLQWYVSEQHEEEKLFKSIIDKLSLAGKSGEG

150   LYFIDKELSTLDTQN

sp|P06974|FLIM_ECOLI Flagellar motor switch protein FliM OS=Escherichia coli (strain K12) OX=83333 GN=fliM PE=1 SV=1
                   ****
0   MGDSILSQAEIDALLNGDSEVKDEPTASVSGESDIRPYDPNTQRRVVRER

50   LQALEIINERFARHFRMGLFNLLRRSPDITVGAIRIQPYHEFARNLPVPT

100   NLNLIHLKPLRGTGLVVFSPSLVFIAVDNLFGGDGRFPTKVEGREFTHTE

150   QRVINRMLKLALEGYSDAWKAINPLEVEYVRSEMQVKFTNITTSPNDIVV

200   NTPFHVEIGNLTGEFNICLPFSMIEPLRELLVNPPLENSRNEDQNWRDNL

250   VRQVQHSQLELVANFADISLRLSQILKLNPGDVLPIEKPDRIIAHVDGVP

300   VLTSQYGTLNGQYALRIEHLINPILNSLNEEQPK

sp|P64530|RCNR_ECOLI Transcriptional repressor RcnR OS=Escherichia coli (strain K12) OX=83333 GN=rcnR PE=1 SV=1

0   MSHTIRDKQKLKARASKIQGQVVALKKMLDEPHECAAVLQQIAAIRGAVN
                       ****
50   GLMREVIKGHLTEHIVHQGDELKREEDLDVVLKVLDSYIK

sp|P45523|FKBA_ECOLI FKBP-type peptidyl-prolyl cis-trans isomerase FkpA OS=Escherichia coli (strain K12) OX=83333 GN=fkpA PE=1 SV=1

0   MKSLFKVTLLATTMAVALHAPITFAAEAAKPATAADSKAAFKNDDQKSAY
             ****
50   ALGASLGRYMENSLKEQEKLGIKLDKDQLIAGVQDAFADKSKLSDQEIEQ
           ****
100   TLQAFEARVKSSAQAKMEKDAADNEAKGKEYREKFAKEKGVKTSSTGLVY

150   QVVEAGKGEAPKDSDTVVVNYKGTLIDGKEFDNSYTRGEPLSFRLDGVIP

200   GWTEGLKNIKKGGKIKLVIPPELAYGKAGVPGIPPNSTLVFDVELLDVKP
      ****
250   APKADAKPEADAKAADSAKK

sp|P04335|FRSA_ECOLI Esterase FrsA OS=Escherichia coli (strain K12) OX=83333 GN=frsA PE=3 SV=2

```
0     MTQANLSETLFKPRFKHPETSTLVRRFNHGAQPPVQSALDGKTIPHWYRM

50    INRLMWIWRGIDPREILDVQARIVMSDAERTDDDLYDTVIGYRGGNWIYE

100   WATQAMVWQQKACAEDDPQLSGRHWLHAATLYNIAAYPHLKGDDLAEQAQ

150   ALSNRAYEEAAQRLPGTMRQMEFTVPGGAPITGFLHMPKGDGPFPTVLMC

200   GGLDAMQTDYYSLYERYFAPRGIAMLTIDMPSVGFSSKWKLTQDSSLLHQ

250   HVLKALPNVPWVDHTRVAAFGFRFGANVAVRLAYLESPRLKAVACLGPVV

300   HTLLSDFKCQQQVPEMYLDVLASRLGMHDASDEALRVELNRYSLKVQGLL
                 *****************************
350   GRRCPTPMLSGYWKNDPFSPEEDSRLITSSSADGKLLEIPFNPVYRNFDK

400   GLQEITDWIEKRLC
```

sp|P67127|YGDQ_ECOLI UPF0053 inner membrane protein YgdQ OS=Escherichia coli (strain K12) OX=83333 GN=ygdQ PE=1 SV=1

```
0     MLFAWITDPNAWLALGTLTLLEIVLGIDNIIFLSLVVAKLPTAQRAHARR
                                                  ****
50    LGLAGAMVMRLALLASIAWVTRLTNPLFTIFSQEISARDLILLLGGLFLI
      *************************
100   WKASKEIHESIEGEEEGLKTRVSSFLGAIVQIMLLDIIFSLDSVITAVGL

150   SDHLFIMMAAVVIAVGVMMFAARSIGDFVERHPSVKMLALSFLILVGFTL

200   ILESFDIHVPKGYIYFAMFFSIAVESLNLIRNKKNPL
```

sp|P24181|ACRF_ECOLI Multidrug export protein AcrF OS=Escherichia coli (strain K12) OX=83333 GN=acrF PE=1 SV=2

```
0     MANFFIRRPIFAWVLAIILMMAGALAILQLPVAQYPTIAPPAVSVSANYP

50    GADAQTVQDTVTQVIEQNMNGIDNLMYMSSTSDSAGSVTITLTFQSGTDP

100   DIAQVQVQNKLQLATPLLPQEVQQQGISVEKSSSSYLMVAGFVSDNPGTT

150   QDDISDYVASNVKDTLSRLNGVGDVQLFGAQYAMRIWLDADLLNKYKLTP

200   VDVINQLKVQNDQIAAGQLGGTPALPGQQLNASIIAQTRFKNPEEFGKVT

250   LRVNSDGSVVRLKDVARVELGGENYNVIARINGKPAAGLGIKLATGANAL

300   DTAKAIKAKLAELQPFFPQGMKVLYPYDTTPFVQLSIHEVVKTLFEAIML

350   VFLVMYLFLQNMRATLIPTIAVPVVLLGTFAILAAFGYSINTLTMFGMVL
                      ********************
400   AIGLLVDDAIVVVENVERVMMEDKLPPKEATEKSMSQIQGALVGIAMVLS

450   AVFIPMAFFGGSTGAIYRQFSITIVSAMALSVLVALILTPALCATLLKPV
```

```
500   SAEHHENKGGFFGWFNTTFDHSVNHYTNSVGKILGSTGRYLLIYALIVAG

550   MVVLFLRLPSSFLPEEDQGVFLTMIQLPAGATQERTQKVLDQVTDYYLKN

600   EKANVESVFTVNGFSFSGQAQNAGMAFVSLKPWEERNGDENSAEAVIHRA

650   KMELGKIRDGFVIPFNMPAIVELGTATGFDFELIDQAGLGHDALTQARNQ

700   LLGMAAQHPASLVSVRPNGLEDTAQFKLEVDQEKAQALGVSLSDINQTIS

750   TALGGTYVNDFIDRGRVKKLYVQADAKFRMLPEDVDKLYVRSANGEMVPF

800   SAFTTSHWVYGSPRLERYNGLPSMEIQGEAAPGTSSGDAMALMENLASKL

850   PAGIGYDWTGMSYQERLSGNQAPALVAISFVVVFLCLAALYESWSIPVSV

900   MLVVPLGIVGVLLAATLFNQKNDVYFMVGLLTTIGLSAKNAILIVEFAKD

950   LMEKEGKGVVEATLMAVRMRLRPILMTSLAFILGVLPLAISNGAGSGAQN

1000  AVGIGVMGGMVSATLLAIFFVPVFFVVIRRCFKG
```

sp|P0AAF1|POTE_ECOLI Putrescine transporter PotE OS=Escherichia coli (strain K12) OX=83333 GN=potE PE=1 SV=1

```
0     MSQAKSNKMGVVQLTILTMVNMMGSGIIMLPTKLAEVGTISIISWLVTAV

50    GSMALAWAFAKCGMFSRKSGGMGGYAEYAFGKSGNFMANYTYGVSLLIAN

100   VAIAISAVGYGTELLGASLSPVQIGLATIGVLWICTVANFGGARITGQIS

150   SITVWGVIIPVVGLCIIGWFWFSPTLYVDSWNPHHAPFFSAVGSSIAMTL

200   WAFLGLESACANTDVVENPERNVPIAVLGGTLGAAVIYIVSTNVIAGIVP

250   NMELANSTAPFGLAFAQMFTPEVGKVIMALMVMSCCGSLLGWQFTIAQVF
      ********************
300   KSSSDEGYFPKIFSRVTKVDAPVQGMLTIVIIQSGLALMTISPSLNSQFN

350   VLVNLAVVTNIIPYILSMAALVIIQKVANVPPSKAKVANFVAFVGAMYSF

400   YALYSSGEEAMLYGSIVTFLGWTLYGLVSPRFELKNKHG
```

sp|P0AB14|YCCJ_ECOLI Uncharacterized protein YccJ OS=Escherichia coli (strain K12) OX=83333 GN=yccJ PE=1 SV=1

```
                                  **********************
0     MPTQEAKAHHVGEWASLRNTSPEIAEAIFEVAGYDEKMAEKIWEEGSDEV
      **************
50    LVKAFAKTDKDSLFWGEQTIERKNV
```

sp|P0AEP1|GALP_ECOLI Galactose-proton symporter OS=Escherichia coli (strain K12) OX=83333 GN=galP PE=1 SV=1

```
0     MPDAKKQGRSNKAMTFFVCFLAALAGLLFGLDIGVIAGALPFIADEFQIT

50    SHTQEWVVSSMMFGAAVGAVGSGWLSFKLGRKKSLMIGAILFVAGSLFSA

100   AAPNVEVLILSRVLLGLAVGVASYTAPLYLSEIAPEKIRGSMISMYQLMI

150   TIGILGAYLSDTAFSYTGAWRWMLGVIIIPAILLLIGVFFLPDSPRWFAA
                     **********************
200   KRRFVDAERVLLRLRDTSAEAKRELDEIRESLQVKQSGWALFKENSNFRR

250   AVFLGVLLQVMQQFTGMNVIMYYAPKIFELAGYTNTTEQMWGTVIVGLTN

300   VLATFIAIGLVDRWGRKPTLTLGFLVMAAGMGVLGTMMHIGIHSPSAQYF

350   AIAMLLMFIVGFAMSAGPLIWVLCSEIQPLKGRDFGITCSTATNWIANMI

400   VGATFLTMLNTLGNANTFWVYAALNVLFILLTLWLVPETKHVSLEHIERN

450   LMKGRKLREIGAHD
```

sp|P0AA89|DOSC_ECOLI Diguanylate cyclase DosC OS=Escherichia coli (strain K12) OX=83333 GN=dosC PE=1 SV=1

```
0     MEMYFKRMKDEWTGLVEQADPPIRAKAAEIAVAHAHYLSIEFYRIVRIDP

50    HAEEFLSNEQVERQLKSAMERWIINVLSAQVDDVERLIQIQHTVAEVHAR
                                                 ***
100   IGIPVEIVEMGFRVLKKILYPVIFSSDYSAAEKLQVYHFSINSIDIAMEV
      *********************************************
150   MTRAFTFSDSSASKEDENYRIFSLLENAEEEKERQIASILSWEIDIIYKI

200   LLDSDLGSSLPLSQADFGLWFNHKGRHYFSGIAEVGHISRLIQDFDGIFN

250   QTMRNTRNLNNRSLRVKFLLQIRNTVSQIITLLRELFEEVSRHEVGMDVL

300   TKLLNRRFLPTIFKREIAHANRTGTPLSVLIIDVDKFKEINDTWGHNTGD

350   EILRKVSQAFYDNVRSSDYVFRYGGDEFIIVLTEASENETLRTAERIRSR

400   VEKTKLKAANGEDIALSLSIGAAMFNGHPDYERLIQIADEALYIAKRRGR

450   NRVELWKASL
```

sp|P0ACY3|YEAG_ECOLI Uncharacterized protein YeaG OS=Escherichia coli (strain K12) OX=83333 GN=yeaG PE=3 SV=1

```
0     MNIFDHYRQRYEAAKDEEFTLQEFLTTCRQDRSAYANAAERLLMAIGEPV

50    MVDTAQEPRLSRLFSNRVIARYPAFEEFYGMEDAIEQIVSYLKHAAQGLE

100   EKKQILYLLGPVGGGKSSLAERLKSLMQLVPIYVLSANGERSPVNDHPFC

150   LFNPQEDAQILEKEYGIPRRYLGTIMSPWAAKRLHEFGGDITKFRVVKVW
```

```
200   PSILQQIAIAKTEPGDENNQDISALVGKVDIRKLEHYAQNDPDAYGYSGA

250   LCRANQGIMEFVEMFKAPIKVLHPLLTATQEGNYNGTEGISALPFNGIIL

300   AHSNESEWVTFRNNKNNEAFLDRVYIVKVPYCLRISEEIKIYEKLLNHSE
                    ************************************
350   LTHAPCAPGTLETLSRFSILSRLKEPENSSIYSKMRVYDGESLKDTDPKA
      **********
400   KSYQEYRDYAGVDEGMNGLSTRFAFKILSRVFNFDHVEVAANPVHLFYVL

450   EQQIEREQFPQEQAERYLEFLKGYLIPKYAEFIGKEIQTAYLESYSEYGQ

500   NIFDRYVTYADFWIQDQEYRDPDTGQLFDRESLNAELEKIEKPAGISNPK
                                                      **
550   DFRNEIVNFVLRARANNSGRNPNWTSYEKLRTVIEKKMFSNTEELLPVIS
      *************************
600   FNAKTSTDEQKKHDDFVDRMMEKGYTRKQVRLLCEWYLRVRKSS
```

sp|P39099|DEGQ_ECOLI Periplasmic pH-dependent serine endoprotease DegQ OS=Escherichia coli (strain K12) OX=83333 GN=degQ PE=1 SV=1

```
0     MKKQTQLLSALALSVGLTLSASFQAVASIPGQVADQAPLPSLAPMLEKVL

50    PAVVSVRVEGTASQGQKIPEEFKKFFGDDLPDQPAQPFEGLGSGVIINAS

100   KGYVLTNNHVINQAQKISIQLNDGREFDAKLIGSDDQSDIALLQIQNPSK

150   LTQIAIADSDKLRVGDFAVAVGNPFGLGQTATSGIVSALGRSGLNLEGLE

200   NFIQTDASINRGNSGGALLNLNGELIGINTAILAPGGGSVGIGFAIPSNM

250   ARTLAQQLIDFGEIKRGLLGIKGTEMSADIAKAFNLDVQRGAFVSEVLPG
                                                       *
300   SGSAKAGVKAGDIITSLNGKPLNSFAELRSRIATTEPGTKVKLGLLRNGK
      *******************
350   PLEVEVTLDTSTSSSASAEMITPALEGATLSDGQLKDGGKGIKIDEVVKG

400   SPAAQAGLQKDDVIIGVNRDRVNSIAEMRKVLAAKPAIIALQIVRGNESI

450   YLLMR
```

sp|P75692|YAHM_ECOLI Uncharacterized protein YahM OS=Escherichia coli (strain K12) OX=83333 GN=yahM PE=4 SV=2

```
                                        ****************
0     MAVQLFKTLLNQIPLLSSLQSGTLPLFGYSGWGRPMKKAHTGESGLKWEA
      *************
50    KDSSKLIGNKDHALRGLSSPMAVIRQIRLIT
```

sp|P76065|YDAU_ECOLI Uncharacterized protein YdaU OS=Escherichia coli (strain K12) OX=83333 GN=ydaU PE=4 SV=1

```
0     MLFVLILSHRAASYGAIMAALPYMQLYIADYLADTMHLSAEEHGAYLLLM
                                                      **
50    FNYWQTGKPIPKNRLAKIARLTNERWADVEPSLQEFFCDNGEEWVHLRIE
```

```
      ********************
100   EDLASVREKLTKKSAAGKASVQARRSRKEADVQTKQERNLTGVQTDVEVV
       ********************
150   FEHDVNTKATNKDTDKDLKTDPPLNPPRGNRGVKKFDPLDITLPNWISVS

200   LWREWVEFRQALRKPIRTEQGANGAIRELEKFRQQGFSPEQVIRHSIANE

250   YQGLFAPKGVRPETLLRQVNTVSLPDSAIPPGFRG
```

sp|P0AAX6|MCBA_ECOLI Uncharacterized protein McbA OS=Escherichia coli (strain K12) OX=83333 GN=mcbA PE=2 SV=2

```
                                                 *****
0     MKKCLTLLIATVLSGISLTAYAAQPMSNLDSGQLRPAGTVSATGASNLSD
      ***************
50    LEDKLAEKAREQGAKGYVINSAGGNDQMFGTATIYK
```

sp|P31665|RPNC_ECOLI Recombination-promoting nuclease RpnC OS=Escherichia coli (strain K12) OX=83333 GN=rpnC PE=3 SV=2

```
                                                  ****
0     MDAPSTTPHDAVFKQFLMHAETARDFLEIHLPVELRELCDLNTLHLESGS
      ****************
50    FIEESLKGHSTDVLYSVQMQGNPGYLHVVIEHQSKPDKKMAFRMMRYSIA

100   AMHRHLEADHDKLPLVVPILFYQGEATPYPLSMCWFDMFYSPELARRVYN

150   SPFPLVDITITPDDEIMQHRRIAILELLQKHIRQRDLMLLLEQLVTLIDE

200   GYTSGSQLVAMQNYMLQRGHTEQADLFYGVLRDRETGGESMMTLAQWFEE

250   KGIEKGIQQGRQEVSQEFAQRLLSKGMSREDVAEMANLPLAEIDKVINLI

300
```

sp|P37751|WBBK_ECOLI Putative glycosyltransferase WbbK OS=Escherichia coli (strain K12) OX=83333 GN=wbbK PE=4 SV=1

```
0     MGKSIVVVSAVNFTTGGPFTILKKFLAATNNKENVSFIALVHSAKELKES

50    YPWVKFIEFPEVKGSWLKRLHFEYVVCKKLSKELNATHWICLHDITANVV

100   TKKRYVYCHNPAPFYKGILFREILMEPSFFLFKMLYGLIYKINIKKNTAV
                         *******************************
150   FVQQFWMKEKFIKKYSINNIIVSRPEIKLSDKSQLTDDDSQFKNNPSELT

200   IFYPAVPRVFKNYELIISAARKLKEQSNIKFLLTISGTENAYAKYIISLA

250   EGLDNVHFLGYLDKEKIDHCYNISDIVCFPSRLETWGLPLSEAKERGKWV

300   LASDFPFTRETLGSYEKKAFFDSNNDDMLVKLIIDFKKGNLKKDISDANF

350   IYRNENVLVGFDELVNFITEEH
```

sp|P76418|YEGU_ECOLI Uncharacterized protein YegU OS=Escherichia coli (strain K12) OX=83333 GN=yegU PE=3 SV=1

```
0     MKTERILGALYGQALGDAMGMPSELWPRSRVKAHFGWIDRFLPGPKENNA

50    ACYFNRAEFTDDTSMALCLADALLEREGKIDPDLIGRNILDWALRFDAFN
                                                     ***
100   KNVLGPTSKIALNAIRDGKPVAELENNGVTNGAAMRVSPLGCLLPARDVD
      *****************
150   SFIDDVALASSPTHKSDLAVAGAVVIAWAISRAIDGESWSAIVDSLPSIA

200   RHAQQKRITTFSASLAARLEIALKIVRNADGTESASEQLYQVVGAGTSTI

250   ESVPCAIALVELAQTDPNRCAVLCANLGGDTDTIGAMATAICGALHGVNA

300   IDPALKAELDAVNQLDFNRYATALAKYRQQREAV
```

sp|P0AFG6|ODO2_ECOLI Dihydrolipoyllysine-residue succinyltransferase component of 2-oxoglutarate dehydrogenase complex OS=Escherichia coli (strain K12) OX=83333 GN=sucB PE=1 SV=2

```
0     MSSVDILVPDLPESVADATVATWHKKPGDAVVRDEVLVEIETDKVVLEVP
                              **************************
50    ASADGILDAVLEDEGTTVTSRQILGRLREGNSAGKETSAKSEEKASTPAQ
      *
100   RQQASLEEQNNDALSPAIRRLLAEHNLDASAIKGTGVGGRLTREDVEKHL

150   AKAPAKESAPAAAAPAAQPALAARSEKRVPMTRLRKRVAERLLEAKNSTA

200   MLTTFNEVNMKPIMDLRKQYGEAFEKRHGIRLGFMSFYVKAVVEALKRYP
                                                   *****
250   EVNASIDGDDVVYHNYFDVSMAVSTPRGLVTPVLRDVDTLGMADIEKKIK
      ***************
300   ELAVKGRDGKLTVEDLTGGNFTITNGGVFGSLMSTPIINPPQSAILGMHA

350   IKDRPMAVNGQVEILPMMYLALSYDHRLIDGRESVGFLVTIKELLEDPTR

400   LLLDV
```

sp|P75764|YBHJ_ECOLI Uncharacterized protein YbhJ OS=Escherichia coli (strain K12) OX=83333 GN=ybhJ PE=3 SV=2

```
                    *******************************
0     MIKLSEKGVFLASNNEIIAEEHFTGEIKKEEAKKGTIAWSILSSHNTSGN

50    MDKLKIKFDSLASHDITFVGIVQTAKASGMERFPLPYVLTNCHNSLCAVG

100   GTINGDDHVFGLSAAQRYGGIFVPPHIAVIHQYMREMMAGGGKMILGSDS

150   HTRYGALGTMAVGEGGGELVKQLLNDTWDIDYPGVVAVHLTGKPAPYVGP

200   QDVALAIIGAVFKNGYVKNKVMEFVGPGVSALSTDFRNSVDVMTTETTCL

250   SSVWQTDEEVHNWLALHGRGQDYCQLNPQPMAYYDGCISVDLSAIKPMIA

300   LPFHPSNVYEIDTLNQNLTDILREIEIESERVAHGKAKLSLLDKVENGRL
```

350   KVQQGIIAGCSGGNYENVIAAANALRGQSCGNDTFSLAVYPSSQPVFMDL

400   AKKGVVADLIGAGAIIRTAFCGPCFGAGDTPINNGLSIRHTTRNFPNREG

450   SKPANGQMSAVALMDARSIAATAANGGYLTSASELDCWDNVPEYAFDVTP

500   YKNRVYQGFVKGATQQPLIYGPNIKDWPELGALTDNIVLKVCSKILDEVT

550   TTDELIPSGETSSYRSNPIGLAEFTLSRRDPGYVSRSKATAELENQRLAG

600   NVSELTEVFARIKQIAGQEHIDPLQTEIGSMVYAVKPGDGSAREQAASCQ

650   RVIGGLANIAEEYATKRYRSNVINWGMLPLQMAEVPTFEVGDYIYIPGIK

700   AALDNPGTTFKGYVIHEDAPVTEITLYMESLTAEEREIIKAGSLINFNKN

750   RQM

sp|P0A6D0|ARGR_ECOLI Arginine repressor OS=Escherichia coli (strain K12) OX=83333 GN=argR PE=1 SV=1
       ****
0   MRSSAKQEELVKAFKALLKEEKFSSQGEIVAALQEQGFDNINQSKVSRML

50   TKFGAVRTRNAKMEMVYCLPAELGVPTTSSPLKNLVLDIDYNDAVVVIHT

100   SPGAAQLIARLLDSLGKAEGILGTIAGDDTIFTTPANGFTVKDLYEAILE

150   LFDQEL

sp|P0ABU2|YCHF_ECOLI Ribosome-binding ATPase YchF OS=Escherichia coli (strain K12) OX=83333 GN=ychF PE=1 SV=2

0   MGFKCGIVGLPNVGKSTLFNALTKAGIEAANFPFCTIEPNTGVVPMPDPR

50   LDQLAEIVKPQRTLPTTMEFVDIAGLVKGASKGEGLGNQFLTNIRETEAI
                                                     ***
100   GHVVRCFENDNIIHVSGKVNPADDIEVINTELALADLDTCERAIHRVQKK
      ****
150   AKGGDKDAKAELAVLEKCLPQLENAGMLRALDLSAEEKAAIRYLSFLTLK

200   PTMYIANVNEDGFENNPYLDQVREIAAKEGSVVVPVCAAVEADIAELDDE

250   ERDEFMQELGLEEPGLNRVIRAGYKLLNLQTYFTAGVKEVRAWTIPVGAT

300   APQAAGKIHTDFEKGFIRAQTISFEDFITYKGEQGAKEAGKMRAEGKDYI

350   VKDGDVMNFLFNV

sp|P0ACG8|HSLR_ECOLI Heat shock protein 15 OS=Escherichia coli (strain K12) OX=83333 GN=hslR PE=1 SV=1

0   MKEKPAVEVRLDKWLWAARFYKTRALAREMIEGGKVHYNGQRSKPSKIVE
                                 ****
50   LNATLTLRQGNDERTVIVKAITEQRRPASEAALLYEETAESVEKREKMAL

```
100   ARKLNALTMPHPDRRPDKKERRDLLRFKHGDSE
```

sp|Q46791|YGEK_ECOLI Uncharacterized response regulatory protein YgeK OS=Escherichia coli (strain K12) OX=83333 GN=ygeK PE=1 SV=3

```
0     MGAELVKWVKSHKIDAHIITFVAKMPYIDSIKLLEAGAKGCVWKTSHPAK
                  ******************************
50    LNRAIDSISNGYTYFDSVHMDCEKISSRYSSDNQLTNRESEILQLIADGK

100   TNKEIANFLQLSRKTVETHRLNIMKKLDVHSGIELIKTALRMGVCTI
```

sp|P0A9A2|FTNB_ECOLI Bacterial non-heme ferritin-like protein OS=Escherichia coli (strain K12) OX=83333 GN=ftnB PE=3 SV=1

```
0     MATAGMLLKLNSQMNREFYASNLYLHLSNWCSEQSLNGTATFLRAQAQSN

50    VTQMMRMFNFMKSVGATPIVKAIDVPGEKLNSLEELFQKTMEEYEQRSST
           *********************
100   LAQLADEAKELNDDSTVNFLRDLEKEQQHDGLLLQTILDEVRSAKLAGMC

150   PVQTDQHVLNVVSHQLH
```

sp|O32528|YPDI_ECOLI Uncharacterized lipoprotein YpdI OS=Escherichia coli (strain K12) OX=83333 GN=ypdI PE=3 SV=1

```
                            **********************
0     MKVNLILFSLFLLVSIMACNVFAFSISGGGSERSYKETEKTSAMTTTHST

50    KLQPSQAILFKMREDAPPLNLTEEMPPPFPTKANYLIHPVR
```

sp|P0AFM6|PSPA_ECOLI Phage shock protein A OS=Escherichia coli (strain K12) OX=83333 GN=pspA PE=1 SV=2

```
0     MGIFSRFADIVNANINALLEKAEDPQKLVRLMIQEMEDTLVEVRSTSARA

50    LAEKKQLTRRIEQASAREVEWQEKAELALLKEREDLARAALIEKQKLTDL
                 ********************
100   IKSLEHEVTLVDDTLARMKKEIGELENKLSETRARQQALMLRHQAANSSR
                              **************************
150   DVRRQLDSGKLDEAMARFESFERRIDQMEAEAESHSFGKQKSLDDQFAEL
      **********
200   KADDAISEQLAQLKAKMKQDNQ
```

sp|P40710|NLPE_ECOLI Lipoprotein NlpE OS=Escherichia coli (strain K12) OX=83333 GN=nlpE PE=1 SV=1

```
0     MVKKAIVTAMAVISLFTLMGCNNRAEVDTLSPAQAAELKPMPQSWRGVLP
                                                   *****
50    CADCEGIETSLFLEKDGTWVMNERYLGAREEPSSFASYGTWARTADKLVL
      *********************
100   TDSKGEKSYYRAKGDALEMLDREGNPIESQFNYTLEAAQSSLPMTPMTLR

150   GMYFYMADAATFTDCATGKRFMVANNAELERSYLAARGHSEKPVLLSVEG
```

```
200   HFTLEGNPDTGAPTKVLAPDTAGKFYPNQDCSSLGQ
```

sp|P0A6W5|GREA_ECOLI Transcription elongation factor GreA OS=Escherichia coli (strain K12) OX=83333 GN=greA PE=1 SV=1

```
0     MQAIPMTLRGAEKLREELDFLKSVRRPEIIAAIAEAREHGDLKENAEYHA
                                                  ****
50    AREQQGFCEGRIKDIEAKLSNAQVIDVTKMPNNGRVIFGATVTVLNLDSD
      *****************
100   EEQTYRIVGDDEADFKQNLISVNSPIARGLIGKEEDDVVVIKTPGGEVEF

150   EVIKVEYL
```

sp|P0A7I0|RF1_ECOLI Peptide chain release factor RF1 OS=Escherichia coli (strain K12) OX=83333 GN=prfA PE=1 SV=1

```
0     MKPSIVAKLEALHERHEEVQALLGDAQTIADQERFRALSREYAQLSDVSR
                        ***************************
50    CFTDWQQVQEDIETAQMMLDDPEMREMAQDELREAKEKSEQLEQQLQVLL

100   LPKDPDDERNAFLEVRAGTGGDEAALFAGDLFRMYSRYAEARRWRVEIMS

150   ASEGEHGGYKEIIAKISGDGVYGRLKFESGGHRVQRVPATESQGRIHTSA

200   CTVAVMPELPDAELPDINPADLRIDTFRSSGAGGQHVNTTDSAIRITHLP

250   TGIVVECQDERSQHKNKAKALSVLGARIHAAEMAKRQQAEASTRRNLLGS

300   GDRSDRNRTYNFPQGRVTDHRINLTLYRLDEVMEGKLDMLIEPIIQEHQA

350   DQLAALSEQE
```

sp|P39366|SGCX_ECOLI Putative aminopeptidase SgcX OS=Escherichia coli (strain K12) OX=83333 GN=sgcX PE=3 SV=3

```
0     MSFSVQETLFSLLQHNAISGHENAVADVMLCEFRRQAKEVWRDRLGNVVA

50    RYGSDKPDALRLMIFAHMDEVGFMVRKIEPSGFLRFERVGGPAQVTMAGS
                                  **********************
100   IVTLTGDKGPVMGCIGIKSYHFAKGDERTQSPSVDKLWIDIGAKDKDDAI

150   RMGIQVGTPVTLYNPPQLLANDLVCSKALDDRLGCTALLGVADAISTMEL

200   DIAVYLVASVQEEFNIRGIVPVLRRVKPDLAIGIDITPSCDTPDLHDYSE

250   VRINQGVGITCLNYHGRGTLAGLITPPRLIRMLEQTALEHNIPVQREVAP

300   GVITETGYIQVEQDGIPCASLSIPCRYTHSPAEVASLRDLTDCIRLLTAL

350   AGMSAAHFPVEPDSGTTQEAHPL
```

sp|P77601|YKGA_ECOLI Putative HTH-type transcriptional regulator YkgA OS=Escherichia coli (strain K12) OX=83333 GN=ykgA PE=5 SV=1

0 MRYDKELTENEMIRQKILQQLLEWIECNLEHPISIEDIAQKSGYSRRNIQ

50 LLFRNFMHVPLGEYIRKRRLCRAAILVRLTAKSMLDIALSLHFDSQQSFS
**

100 REFKKLFGCSPREYRHRDYWDLANIFPSFLIRQQQKTECRLINFPETPIF
****

150 GNSFKYDIEVSNKSPDEEVKLRRHHLARCMKNFKTDIYFVSTFEPSTKSV

200 DLLTVETFAGTVCEYADMPKEWTTTRGLYDPTHVIWTQA

sp|P00579|RPOD_ECOLI RNA polymerase sigma factor RpoD OS=Escherichia coli (strain K12) OX=83333 GN=rpoD PE=1 SV=2

0 MEQNPQSQLKLLVTRGKEQGYLTYAEVNDHLPEDIVDSDQIEDIIQMIND

50 MGIQVMEEAPDADDLMLAENTADEDAAEAAAQVLSSVESEIGRTTDPVRM

100 YMREMGTVELLTREGEIDIAKRIEDGINQVQCSVAEYPEAITYLLEQYDR

150 VEAEEARLSDLITGFVDPNAEEDLAPTATHVGSELSQEDLDDDEDEDEED
****

200 GDDDSADDDNSIDPELAREKFAELRAQYVVTRDTIKAKGRSHATAQEEIL

250 KLSEVFKQFRLVPKQFDYLVNSMRVMMDRVRTQERLIMKLCVEQCKMPKK

300 NFITLFTGNETSDTWFNAAIAMNKPWSEKLHDVSEEVHRALQKLQQIEEE

350 TGLTIEQVKDINRRMSIGEAKARRAKKEMVEANLRLVISIAKKYTNRGLQ

400 FLDLIQEGNIGLMKAVDKFEYRRGYKFSTYATWWIRQAITRSIADQARTI
***

450 RIPVHMIETINKLNRISRQMLQEMGREPTPEELAERMLMPEDKIRKVLKI
****

500 AKEPISMETPIGDDEDSHLGDFIEDTTLELPLDSATTESLRAATHDVLAG

550 LTAREAKVLRMRFGIDMNTDYTLEEVGKQFDVTRERIRQIEAKALRKLRH

600 PSRSEVLRSFLDD

sp|P76168|INTQ_ECOLI Putative defective protein IntQ OS=Escherichia coli (strain K12) OX=83333 GN=intQ PE=1 SV=3

0 MITDVWKYRGKSTGELRSSVCYAIKTGVFDYAKQFPSSRNLEKFGEARQD
****

50 LTIKELAEKFLALKETEVAKTSLNTYRAVIKNILSIIGEKNLASSINKEK
****

100 LLEVRKELLTGYQIPKSNYIVTQPGRSAVTVNNYMTNLNAVFQFGVDNGY

150 LADNPFKGISPLKESRTIPDPLSREEFIRLIDACRNQQAKNLWCVSVYTG

200 VRPGELCALGWEDIDLKNGTMMIRRNLAKDRFTVPKTQAGTNRVIHLIKP

250 AIDALRSQMTLTRLSKEHIIDVHFREYGRTEKQKCTFVFQPEVSARVKNY

300   GDHFTVDSIRQMWDAAIKRAGLRHRKSYQSRHTYACWSLTAGANPAFIAN

350   QMGHADAQMVFQVYGKWMSENNNAQVALLNTQLSEFAPTMPHNEAMKN

sp|P55135|RLMD_ECOLI 23S rRNA (uracil(1939)-C(5))-methyltransferase RlmD OS=Escherichia coli (strain K12) OX=83333 GN=rlmD PE=1 SV=3

0   MAQFYSAKRRTTTRQIITVSVNDLDSFGQGVARHNGKTLFIPGLLPQENA

50   EVTVTEDKKQYARAKVVRRLSDSPERETPRCPHFGVCGGCQQQHASVDLQ

100   QRSKSAALARLMKHDVSEVIADVPWGYRRRARLSLNYLPKTQQLQMGFRK

150   AGSSDIVDVKQCPILAPQLEALLPKVRACLGSLQAMRHLGHVELVQATSG
               ****
200   TLMILRHTAPLSSADREKLERFSHSEGLDLYLAPDSEILETVSGEMPWYD

250   SNGLRLTFSPRDFIQVNAGVNQKMVARALEWLDVQPEDRVLDLFCGMGNF

300   TLPLATQAASVVGVEGVPALVEKGQQNARLNGLQNVTFYHENLEEDVTKQ

350   PWAKNGFDKVLLDPARAGAAGVMQQIIKLEPIRIVYVSCNPATLARDSEA

400   LLKAGYTIARLAMLDMFPHTGHLESMVLFSRVK

sp|P0AE82|CPXA_ECOLI Sensor histidine kinase CpxA OS=Escherichia coli (strain K12) OX=83333 GN=cpxA PE=1 SV=1

0   MIGSLTARIFAIFWLTLALVLMLVLMLPKLDSRQMTELLDSEQRQGLMIE

50   QHVEAELANDPPNDLMWWRRLFRAIDKWAPPGQRLLLVTTEGRVIGAERS

100   EMQIIRNFIGQADNADHPQKKKYGRVELVGPFSVRDGEDNYQLYLIRPAS

150   SSQSDFINLLFDRPLLLLIVTMLVSTPLLLWLAWSLAKPARKLKNAADEV

200   AQGNLRQHPELEAGPQEFLAAGASFNQMVTALERMMTSQQRLLSDISHEL
                      ****
250   RTPLTRLQLGTALLRRRSGESKELERIETEAQRLDSMINDLLVMSRNQQK

300   NALVSETIKANQLWSEVLDNAAFEAEQMGKSLTVNFPPGPWPLYGNPNAL

350   ESALENIVRNALRYSHTKIEVGFAVDKDGITITVDDDGPGVSPEDREQIF

400   RPFYRTDEARDRESGGTGLGLAIVETAIQQHRGWVKAEDSPLGGLRLVIW

450   LPLYKRS

sp|P52128|YFJM_ECOLI Uncharacterized protein YfjM OS=Escherichia coli (strain K12) OX=83333 GN=yfjM PE=4 SV=1
                  ****
0   MGDDVHRFGNLFLISPSSNSILSNYSPADKKKFYVETERAESPKQAIMMS

50   YKEWGPDGQGINNIESHEHAMLTLLKEHRDMTLPTRK

sp|P77695|GNSB_ECOLI Protein GnsB OS=Escherichia coli (strain K12) OX=83333 GN=gnsB PE=3 SV=2

```
            *****************************
0     MNIENLKTKAEADISEYITKKIIELKKKTGKEVTSIQFTAREKMTGLESY

50    DVKINLI
```

sp|P0A6L9|HSCB_ECOLI Co-chaperone protein HscB OS=Escherichia coli (strain K12) OX=83333 GN=hscB PE=1 SV=1

```
0     MDYFTLFGLPARYQLDTQALSLRFQDLQRQYHPDKFASGSQAEQLAAVQQ
                                               *********
50    SATINQAWQTLRHPLMRAEYLLSLHGFDLASEQHTVRDTAFLMEQLELRE
      ***********************
100   ELDEIEQAKDEARLESFIKRVKKMFDTRHQLMVEQLDNETWDAAADTVRK

150   LRFLDKLRSSAEQLEEKLLDF
```

sp|P37749|WBBI_ECOLI Beta-1,6-galactofuranosyltransferase WbbI OS=Escherichia coli (strain K12) OX=83333 GN=wbbI PE=1 SV=1

```
0     MYFLNDLNFSRRDAGFKARKDALDIASDYENISVVNIPLWGGVVQRIISS

50    VKLSTFLCGLENKDVLIFNFPMAKPFWHILSFFHRLLKFRIVPLIHDIDE
                                     *************************
100   LRGGGGSDSVRLATCDMVISHNPQMTKYLSKYMSQDKIKDIKIFDYLVSS
      ******
150   DVEHRDVTDKQRGVIYAGNLSRHKCSFIYTEGCDFTLFGVNYENKDNPKY

200   LGSFDAQSPEKINLPGMQFGLIWDGDSVETCSGAFGDYLKFNNPHKTSLY

250   LSMELPVFIWDKAALADFIVDNRIGYAVGSIKEMQEIVDSMTIETYKQIS

300   ENTKIISQKIRTGSYFRDVLEEVIDDLKTR
```

sp|P10442|RNH2_ECOLI Ribonuclease HII OS=Escherichia coli (strain K12) OX=83333 GN=rnhB PE=1 SV=2

```
                                                ********
0     MIEFVYPHTQLVAGVDEVGRGPLVGAVVTAAVILDPARPIAGLNDSKKLS
      *******************
50    EKRRLALYEEIKEKALSWSLGRAEPHEIDELNILHATMLAMQRAVAGLHI

100   APEYVLIDGNRCPKLPMPAMAVVKGDSRVPEISAASILAKVTRDAEMAAL

150   DIVFPQYGFAQHKGYPTAFHLEKLAEHGATEHHRRSFGPVKRALGLAS
```

sp|P33919|RADD_ECOLI Putative DNA repair helicase RadD OS=Escherichia coli (strain K12) OX=83333 GN=radD PE=1 SV=2

```
0     MIFTLRPYQQEAVDATLNHFRRHKTPAVIVLPTGAGKSLVIAELARLARG

50    RVLVLAHVKELVAQNHAKYQALGLEADIFAAGLKRKESHGKVVFGSVQSV
```

100 ARNLDAFQGEFSLLIVDECHRIGDDEESQYQQILTHLTKVNPHLRLLGLT

150 ATPFRLGKGWIYQFHYHGMVRGDEKALFRDCIYELPLRYMIKHGYLTPPE

200 RLDMPVVQYDFSRLQAQSNGLFSEADLNRELKKQQRITPHIISQIMEFAA

250 TRKGVMIFAATVEHAKEIVGLLPAEDAALITGDTPGAERDVLIENFKAQR

300 FRYLVNVAVLTTGFDAPHVDLIAILRPTESVSLYQQIVGRGLRLAPGKTD

350 CLILDYAGNPHDLYAPEVGTPKGKSDNVPVQVFCPACGFANTFWGKTTAD

400 GTLIEHFGRRCQGWFEDDDGHREQCDFRFRFKNCPQCNAENDIAARRCRE
****

450 CDTVLVDPDDMLKAALRLKDALVLRCSGMSLQHGHDEKGEWLKITYYDED
******

500 GADVSERFRLQTPAQRTAFEQLFIRPHTRTPGIPLRWITAADILAQQALL

550 RHPDFVVARMKGQYWQVREKVFDYEGRFRLAHELRG

sp|P0A9U3|YBIT_ECOLI Uncharacterized ABC transporter ATP-binding protein YbiT OS=Escherichia coli (strain K12) OX=83333 GN=ybiT PE=1 SV=1

0 MLVSSNVTMQFGSKPLFENISVKFGGGNRYGLIGANGSGKSTFMKILGGD

50 LEPTLGNVSLDPNERIGKLRQDQFAFEEFTVLDTVIMGHKELWEVKQERD
****

100 RIYALPEMSEEDGYKVADLEVKYGEMDGYSAEARAGELLLGVGIPVEQHY

150 GPMSEVAPGWKLRVLLAQALFADPDILLLDEPTNNLDIDTIRWLEQVLNE

200 RDSTMIIISHDRHFLNMVCTHMADLDYGELRVYPGNYDEYMTAATQARER

250 LLADNAKKKAQIAELQSFVSRFSANASKSRQATSRARQIDKIKLEEVKAS

300 SRQNPFIRFEQDKKLFRNALEVEGLTKGFDNGPLFKNLNLLLEVGEKLAV

350 LGTNGVGKSTLLKTLVGDLQPDSGTVKWSENARIGYYAQDHEYEFENDLT

400 VFEWMSQWKQEGDDEQAVRSILGRLLFSQDDIKKPAKVLSGGEKGRMLFG

450 KLMMQKPNILIMDEPTNHLDMESIESLNMALELYQGTLIFVSHDREFVSS

500 LATRILEITPERVIDFSGNYEDYLRSKGIE

sp|P77148|YDHS_ECOLI Uncharacterized protein YdhS OS=Escherichia coli (strain K12) OX=83333 GN=ydhS PE=4 SV=1

0 MKKIAIVGAGPTGIYTLFSLLQQQTPLSISIFEQADEAGVGMPYSDEENS

50 KMMLANIASIEIPPIYCTYLEWLQKQEDSHLQRYGVKKETLHDRQFLPRI

100 LLGEYFRDQFLRLVDQARQQKFAVAVYESCQVTDLQITNAGVMLATNQDL

150 PSETFDLAVIATGHVWPDEEEATRTYFPSPWSGLMEAKVDACNVGIMGTS

200 LSGLDAAMAVAIQHGSFIEDDKQHVVFHRDNASEKLNITLLSRTGILPEA

250 DFYCPIPYEPLHIVTDQALNAEIQKGEEGLLDRVFRLIVEEIKFADPDWS

300 QRIALESLNVDSFAQAWFAERKQRDPFDWAEKNLQEVERNKREKHTVPWR

350 YVILRLHEAVQEIVPHLNEHDHKRFSKGLARVFIDNYAAIPSESIRRLLA

****

400 LREAGIIHILALGEDYKMEINESRTVLKTEDNSYSFDVFIDARGQRPLKV

450 KDIPFPGLREQLQKTGDEIPDVGEDYTLQQPEDIRGRVAFGALPWLMHDQ

500 PFVQGLTACAEIGEAMARAVVKPASRARRRLSFD

sp|P58094|YCIX_ECOLI Uncharacterized protein YciX OS=Escherichia coli (strain K12) OX=83333 GN=yciX PE=4 SV=2

****

0 MVGQEQLESSPLCQHSDNETETKRECSVVIPDDWQLTSQQQAFIELFAED

50 DQPKQ

sp|P33924|YEJO_ECOLI Putative uncharacterized outer membrane protein YejO OS=Escherichia coli (strain K12) OX=83333 GN=yejO PE=5 SV=1

0 MHQSGSVSLCRSAISVLVATALYSPIALASTVEYGETVDGVVLEKDIQLV

50 YGTANNTKINPGGEQHIKEFGVSNNTEINGGYQYIEMNGAAEYSVLNDGY

100 QIVQMGGAANQTTLNNGVLQVYGAANDTTIKGGRLIVEKDGGAVFVAIEK

150 GGLLEVKEGGFAFAVDQKAGGAIKTTTRAMEVFGTNRLGQFDIKNGIANN

200 MLLENGGSLRVEENDFAYNTTVDSGGLLEVMDGGTVTGVDKKAGGKLIVS

****

250 TNALEVSGPNSKGQFSIKDGVSKNYELDDGSGLIVMEDTQAIDTILDKHA

300 TMQSLGKDTGTKVQANAVYDLGRSYQNGSITYSSKAISENMVINNGRANV

350 WAGTMVNVSVRGNDGILEVMKPQINYAPAMLVGKVVVSEGASFRTHGAVD

400 TSKADVSLENSVWTIIADITTTNQNTLLNLANLAMSDANVIMMDEPVTRS

450 SVTASAENFITLTTNTLSGNGNFYMRTDMANHQSDQLNVTGQATGDFKIF

500 VTDTGASPAAGDSLTLVTTGGGDAAFTLGNAGGVVDIGTYEYTLLDNGNH

550 SWSLAENRAQITPSTTDVLNMAAAQPLVFDAELDTVRERLGSVKGVSYDT

600 AMWSSAINTRNNVTTDAGAGFEQTLTGLTLGIDSRFSREESSTIRGLIFG

650 YSHSDIGFDRGGKGNIDSYTLGAYAGWEHQNGAYVDGVVKVDRFANTIHG

700   KMSNGATAFGDYNSNGAGAHVESGFRWVDGLWSVRPYLAFTGFTTDGQDY

750   TLSNGMRADVGNTRILRAEAGTAVSYHMDLQNGTTLEPWLKAAVRQEYAD

800   SNQVKVNDDGKFNNDVAGTSGVYQAGIRSSFTPTLSGHLSVSYGNGAGVE

850   SPWNTQAGVVWTF

sp|P45800|IGAA_ECOLI Putative membrane protein IgaA homolog
OS=Escherichia coli (strain K12) OX=83333 GN=yrfF PE=1 SV=1

0   MSTIVIFLAALLACSLLAGWLIKVRSRRRQLPWTNAFADAQTRKLTPEER

50   SAVENYLESLTQVLQVPGPTGASAAPISLALNAESNNVMMLTHAITRYGI

100   STDDPNKWRYYLDSVEVHLPPFWEQYINDENTVELIHTDSLPLVISLNGH

150   TLQEYMQETRSYALQPVPSTQASIRGEESEQIELLNIRKETHEEYALSRP

200   RGLREALLIVASFLMFFFCLITPDVFVPWLAGGALLLLGAGLWGLFAPPA

250   KSSLREIHCLRGTPRRWGLFGENDQEQINNISLGIIDLVYPAHWQPYIAQ

300   DLGQQTDIDIYLDRHVVRQGRYLSLHDEVKNFPLQHWLRSTIIAAGSLLV

350   LFMLLFWIPLDMPLKFTLSWMKGAQTIEATSVKQLADAGVRVGDTLRISG

400   TGMCNIRTSGTWSAKTNSPFLPFDCSQIIWNDARSLPLPESELVNKATAL
         ****
450   TEAVNRQLHPKPEDESRVSASLRSAIQKSGMVLLDDFGDIVLKTADLCSA

500   KDDCVRLKNALVNLGNSKDWDALVKRANAGKLDGVNVLLRPVSAESLDNL

550   VATSTAPFITHETARAAQSLNSPAPGGFLIVSDEGSDFVDQPWPSASLYD

600   YPPQEQWNAFQKLAQMLMHTPFNAEGIVTKIFTDANGTQHIGLHPIPDRS

650   GLWRYLSTTLLLLTMLGSAIYNGVQAWRRYQRHRTRMMEIQAYYESCLNP

700   QLITPSESLIE

sp|P46118|HEXR_ECOLI HTH-type transcriptional regulator HexR
OS=Escherichia coli (strain K12) OX=83333 GN=hexR PE=3 SV=2
         ****
0   MNMLEKIQSQLEHLSKSERKVAEVILASPDNAIHSSIAAMALEANVSEPT

50   VNRFCRSMDTRGFPDFKLHLAQSLANGTPYVNRNVNEDDSVESYTGKIFE

100   SAMATLDHVRHSLDKSAINRAVDLLTQAKKIAFFGLGSSAAVAHDAMNKF

150   FRFNVPVVYSDDIVLQRMSCMNCSDGDVVVLISHTGRTKNLVELAQLARE

200   NDAMVIALTSAGTPLAREATLAITLDVPEDTDIYMPMVSRLAQLTVIDVL
         ****

```
250   ATGFTLRRGAKFRDNLKRVKEALKESRFDKQLLNLSDDR

sp|P08839|PT1_ECOLI Phosphoenolpyruvate-protein phosphotransferase
OS=Escherichia coli (strain K12) OX=83333 GN=ptsI PE=1 SV=1
                    ***************************
0     MISGILASPGIAFGKALLLKEDEIVIDRKKISADQVDQEVERFLSGRAKA
                      ********************
50    SAQLETIKTKAGETFGEEKEAIFEGHIMLLEDEELEQEIIALIKDKHMTA
           ******************************
100   DAAAHEVIEGQASALEELDDEYLKERAADVRDIGKRLLRNILGLKIIDLS

150   AIQDEVILVAADLTPSETAQLNLKKVLGFITDAGGRTSHTSIMARSLELP

200   AIVGTGSVTSQVKNDDYLILDAVNNQVYVNPTNEVIDKMRAVQEQVASEK

250   AELAKLKDLPAITLDGHQVEVCANIGTVRDVEGAERNGAEGVGLYRTEFL

300   FMDRDALPTEEEQFAAYKAVAEACGSQAVIVRTMDIGGDKELPYMNFPKE

350   ENPFLGWRAIRIAMDRREILRDQLRAILRASAFGKLRIMFPMIISVEEVR
        ************************
400   ALRKEIEIYKQELRDEGKAFDESIEIGVMVETPAAATIARHLAKEVDFFS

450   IGTNDLTQYTLAVDRGNDMISHLYQPMSPSVLNLIKQVIDASHAEGKWTG

500   MCGELAGDERATLLLLGMGLDEFSMSAISIPRIKKIIRNTNFEDAKVLAE

550   QALAQPTTDELMTLVNKFIEEKTIC

sp|P0AGC0|UHPT_ECOLI Hexose-6-phosphate:phosphate antiporter
OS=Escherichia coli (strain K12) OX=83333 GN=uhpT PE=1 SV=1

0     MLAFLNQVRKPTLDLPLEVRRKMWFKPFMQSYLVVFIGYLTMYLIRKNFN

50    IAQNDMISTYGLSMTQLGMIGLGFSITYGVGKTLVSYYADGKNTKQFLPF

100   MLILSAICMLGFSASMGSGSVSLFLMIAFYALSGFFQSTGGSCSYSTITK

150   WTPRRKRGTFLGFWNISHNLGGAGAAGVALFGANYLFDGHVIGMFIFPSI
                  **************************************
200   IALIVGFIGLRYGSDSPESYGLGKAEELFGEEISEEDKETESTDMTKWQI
      ****
250   FVEYVLKNKVIWLLCFANIFLYVVRIGIDQWSTVYAFQELKLSKAVAIQG

300   FTLFEAGALVGTLLWGWLSDLANGRRGLVACIALALIIATLGVYQHASNE

350   YIYLASLFALGFLVFGPQLLIGVAAVGFVPKKAIGAADGIKGTFAYLIGD

400   SFAKLGLGMIADGTPVFGLTGWAGTFAALDIAAIGCICLMAIVAVMEERK

450   IRREKKIQQLTVA

sp|P76249|LEUE_ECOLI Leucine efflux protein OS=Escherichia coli (strain
K12) OX=83333 GN=leuE PE=1 SV=1
```

```
0     MFAEYGVLNYWTYLVGAIFIVLVPGPNTLFVLKNSVSSGMKGGYLAACGV
                                                ********** 
50    FIGDAVLMFLAWAGVATLIKTTPILFNIVRYLGAFYLLYLGSKILYATLK
      ***********
100   GKNSEAKSDEPQYGAIFKRALILSLTNPKAILFYVSFFVQFIDVNAPHTG

150   ISFFILAATLELVSFCYLSFLIISGAFVTQYIRTKKKLAKVGNSLIGLMF

200   VGFAARLATLQS
```

sp|P38036|CAS3_ECOLI CRISPR-associated endonuclease/helicase Cas3 OS=Escherichia coli (strain K12) OX=83333 GN=ygcB PE=1 SV=2

```
0     MEPFKYICHYWGKSSKSLTKGNDIHLLIYHCLDVAAVADCWWDQSVVLQN

50    TFCRNEMLSKQRVKAWLLFFIALHDIGKFDIRFQYKSAESWLKLNPATPS

100   LNGPSTQMCRKFNHGAAGLYWFNQDSLSEQSLGDFFSFFDAAPHPYESWF

150   PWVEAVTGHHGFILHSQDQDKSRWEMPASLASYAAQDKQAREEWISVLEA

200   LFLTPAGLSINDIPPDCSSLLAGFCSLADWLGSWTTTNTFLFNEDAPSDI

250   NALRTYFQDRQQDASRVLELSGLVSNKRCYEGVHALLDNGYQPRQLQVLV

300   DALPVAPGLTVIEAPTGSGKTETALAYAWKLIDQQIADSVIFALPTQATA

350   NAMLTRMEASASHLFSSPNLILAHGNSRFNHLFQSIKSRAITEQGQEEAW

400   VQCCQWLSQSNKKVFLGQIGVCTIDQVLISVLPVKHRFIRGLGIGRSVLI

450   VDEVHAYDTYMNGLLEAVLKAQADVGGSVILLSATLPMKQKQKLLDTYGL

500   HTDPVENNSAYPLINWRGVNGAQRFDLLAHPEQLPPRFSIQPEPICLADM

550   LPDLTMLERMIAAANAGAQVCLICNLVDVAQVCYQRLKELNNTQVDIDLF

600   HARFTLNDRREKENRVISNFGKNGKRNVGRILVATQVVEQSLDVDFDWLI

650   TQHCPADLLFQRLGRLHRHHRKYRPAGFEIPVATILLPDGEGYGRHEHIY
                                           ***************
700   SNVRVMWRTQQHIEELNGASLFFPDAYRQWLDSIYDDAEMDEPEWVGNGM
      **********
750   DKFESAECEKRFKARKVLQWAEEYSLQDNDETILAVTRDGEMSLPLLPYV

800   QTSSGKQLLDGQVYEDLSHEQQYEALALNRVNVPFTWKRSFSEVVDEDGL

850   LWLEGKQNLDGWVWQGNSIVITYTGDEGMTRVIPANPK
```

sp|P37691|YIBQ_ECOLI Uncharacterized protein YibQ OS=Escherichia coli (strain K12) OX=83333 GN=yibQ PE=3 SV=2

```
0     MFPFRRNVLAFAALLALSSPVLAGKLAIVIDDFGYRPHNENQVLAMPSAI
```

****
50 SVAVLPDSPHAREMATKAHNSGHEVLIHLPMAPLSKQPLEKNTLRPEMSS
****
100 DEIERIIRSAVNNVPYAVGINNHMGSKMTSNLFGMQKVMQALERYNLYFL

150 DSVTIGNTQAMRAAQGTGVKVIKRKVFLDDSQNEADIRVQFNRAIDLARR

200 NGSTIAIGHPHPATVRVLQQMVYNLPPDITLVKASSLLNEPQVDTSTPPK

250 NAVPDAPRNPFRGVKLCKPKKPIEPVYANRFFEVLSESISQSTLIVYFQH

300 QWQGWGKQPEAAKFNASAN

sp|P76119|YNCI_ECOLI Putative transposase YncI OS=Escherichia coli (strain K12) OX=83333 GN=yncI PE=3 SV=3

0 MSIQSLLDYISVTPDIRQQGKVKHKLSAILFLTVCAVIAGADEWQEIEDF

50 GHERLEWLKKYGDFDNGIPVDDTIARVVSNIDSLAFEKMFIEWMQECHEI

100 TDGEIIAIDGKTIRGSFDKGKRKGAIHMVSAFSNENGVVLGQVKTEAKSN
****
150 EITAIPELLNLLYLKKNLITIDAMGCQKDIASKIKDKKADYLLAVKGNQG

200 KLHHAFEEKFPVNVFSNYKGDSFSTQEISHGRKETRLHIVSNVTPELL

sp|P02358|RS6_ECOLI 30S ribosomal protein S6 OS=Escherichia coli (strain K12) OX=83333 GN=rpsF PE=1 SV=1

0 MRHYEIVFMVHPDQSEQVPGMIERYTAAITGAEGKIHRLEDWGRRQLAYP

50 INKLHKAHYVLMNVEAPQEVIDELETTFRFNDAVIRSMVMRTKHAVTEAS
****
100 PMVKAKDERRERRDDFANETADDAEAGDSEEEEE

sp|P0ABC3|HFLC_ECOLI Modulator of FtsH protease HflC OS=Escherichia coli (strain K12) OX=83333 GN=hflC PE=1 SV=1

0 MRKSVIAIIIIVLVVLYMSVFVVKEGERGITLRFGKVLRDDDNKPLVYEP
****
50 GLHFKIPFIETVKMLDARIQTMDNQADRFVTKEKKDLIVDSYIKWRISDF
**
100 SRYYLATGGGDISQAEVLLKRKFSDRLRSEIGRLDVKDIVTDSRGRLTLE

150 VRDALNSGSAGTEDEVTTPAADNAIAEAAERVTAETKGKVPVINPNSMAA

200 LGIEVVDVRIKQINLPTEVSEAIYNRMRAEREAVARRHRSQGQEEAEKLR

250 ATADYEVTRTLAEAERQGRIMRGEGDAEAAKLFADAFSKDPDFYAFIRSL

300 RAYENSFSGNQDVMVMSPDSDFFRYMKTPTSATR

sp|P04994|EX7L_ECOLI Exodeoxyribonuclease 7 large subunit OS=Escherichia coli (strain K12) OX=83333 GN=xseA PE=1 SV=2

```
0     MLPSQSPAIFTVSRLNQTVRLLLEHEMGQVWISGEISNFTQPASGHWYFT

50    LKDDTAQVRCAMFRNSNRRVTFRPQHGQQVLVRANITLYEPRGDYQIIVE

100   SMQPAGEGLLQQKYEQLKAKLQAEGLFDQQYKKPLPSPAHCVGVITSKTG

150   AALHDILHVLKRRDPSLPVIIYPAAVQGDDAPGQIVRAIELANQRNECDV

200   LIVGRGGGSLEDLWSFNDERVARAIFTSRIPVVSAVGHETDVTIADFVAD

250   LRAPTPSAAAEVVSRNQQELLRQVQSTRQRLEMAMDYYLANRTRRFTQIH

300   HRLQQQHPQLRLARQQTMLERLQKRMSFALENQLKRTGQQQQRLTQRLNQ

350   QNPQPKIHRAQTRIQQLEYRLAETLRAQLSATRERFGNAVTHLEAVSPLS
                                       *****************
400   TLARGYSVTTATDGNVLKKVKQVKAGEMLTTRLEDGWIESEVKNIQPVKK
      ****
450   SRKKVH
```

sp|P0AAD8|TDCC_ECOLI Threonine/serine transporter TdcC OS=Escherichia coli (strain K12) OX=83333 GN=tdcC PE=1 SV=1

```
      ************************
0     MSTSDSIVSSQTKQSSWRKSDTTWTLGLFGTAIGAGVLFFPIRAGFGGLI

50    PILLMLVLAYPIAFYCHRALARLCLSGSNPSGNITETVEEHFGKTGGVVI

100   TFLYFFAICPLLWIYGVTITNTFMTFWENQLGFAPLNRGFVALFLLLLMA

150   FVIWFGKDLMVKVMSYLVWPFIASLVLISLSLIPYWNSAVIDQVDLGSLS
                            ****************************
200   LTGHDGILITVWLGISIMVFSFNFSPIVSSFVVSKREEYEKDFGRDFTER
      ****
250   KCSQIISRASMLMVAVVMFFAFSCLFTLSPANMAEAKAQNIPVLSYLANH

300   FASMTGTKTTFAITLEYAASIIALVAIFKSFFGHYLGTLEGLNGLVLKFG

350   YKGDKTKVSLGKLNTISMIFIMGSTWVVAYANPNILDLIEAMGAPIIASL

400   LCLLPMYAIRKAPSLAKYRGRLDNVFVTVIGLLTILNIVYKLF
```

sp|P0AEE5|DGAL_ECOLI D-galactose-binding periplasmic protein OS=Escherichia coli (strain K12) OX=83333 GN=mglB PE=1 SV=1

```
0     MNKKVLTLSAVMASMLFGAAAHAADTRIGVTIYKYDDNFMSVVRKAIEQD

50    AKAAPDVQLLMNDSQNDQSKQNDQIDVLLAKGVKALAINLVDPAAAGTVI
                       **********************
100   EKARGQNVPVVFFNKEPSRKALDSYDKAYYVGTDSKESGIIQGDLIAKHW

150   AANQGWDLNKDGQIQFVLLKGEPGHPDAEARTTYVIKELNDKGIKTEQLQ

200   LDTAMWDTAQAKDKMDAWLSGPNANKIEVVIANNDAMAMGAVEALKAHNK
```

250 SSIPVFGVDALPEALALVKSGALAGTVLNDANNQAKATFDLAKNLADGKG

300 AADGTNWKIDNKVVRVPYVGVDKDNLAEFSKK

sp|P24183|FDNG_ECOLI Formate dehydrogenase, nitrate-inducible, major subunit OS=Escherichia coli (strain K12) OX=83333 GN=fdnG PE=1 SV=3

0 MDVSRRQFFKICAGGMAGTTVAALGFAPKQALAQARNYKLLRAKEIRNTC

50 TYCSVGCGLLMYSLGDGAKNAREAIYHIEGDPDHPVSRGALCPKGAGLLD

100 YVNSENRLRYPEYRAPGSDKWQRISWEEAFSRIAKLMKADRDANFIEKNE

150 QGVTVNRWLSTGMLCASGASNETGMLTQKFARSLGMLAVDNQARVUHGPT

200 VASLAPTFGRGAMTNHWVDIKNANVVMVMGGNAAEAHPVGFRWAMEAKNN

250 NDATLIVVDPRFTRTASVADIYAPIRSGTDITFLSGVLRYLIENNKINAE

****

300 YVKHYTNASLLVRDDFAFEDGLFSGYDAEKRQYDKSSWNYQLDENGYAKR

350 DETLTHPRCVWNLLKEHVSRYTPDVVENICGTPKADFLKVCEVLASTSAP

400 DRTTTFLYALGWTQHTVGAQNIRTMAMIQLLLGNMGMAGGGVNALRGHSN

450 IQGLTDLGLLSTSLPGYLTLPSEKQVDLQSYLEANTPKATLADQVNYWSN

500 YPKFFVSLMKSFYGDAAQKENNWGYDWLPKWDQTYDVIKYFNMMDEGKVT

550 GYFCQGFNPVASFPDKNKVVSCLSKLKYMVVIDPLVTETSTFWQNHGESN

600 DVDPASIQTEVFRLPSTCFAEEDGSIANSGRWLQWHWKGQDAPGEARNDG

650 EILAGIYHHLRELYQSEGGKGVEPLMKMSWNYKQPHEPQSDEVAKENNGY

700 ALEDLYDANGVLIAKKGQLLSSFAHLRDDGTTASSCWIYTGSWTEQGNQM

750 ANRDNSDPSGLGNTLGWAWAWPLNRRVLYNRASADINGKPWDPKRMLIQW

800 NGSKWTGNDIPDFGNAAPGTPTGPFIMQPEGMGRLFAINKMAEGPFPEHY

850 EPIETPLGTNPLHPNVVSNPVVRLYEQDALRMGKKEQFPYVGTTYRLTEH

900 FHTWTKHALLNAIAQPEQFVEISETLAAAKGINNGDRVTVSSKRGFIRAV

950 AVVTRRLKPLNVNGQQVETVGIPIHWGFEGVARKGYIANTLTPNVGDANS

1000 QTPEYKAFLVNIEKA

sp|P0ADW3|YHCB_ECOLI Inner membrane protein YhcB OS=Escherichia coli (strain K12) OX=83333 GN=yhcB PE=1 SV=2

****

0 MTWEYALIGLVVGIIIGAVAMRFGNRKLRQQQALQYELEKNKAELDEYRE

```
      ******
50    ELVSHFARSAELLDTMAHDYRQLYQHMAKSSSSLLPELSAEANPFRNRLA

100   ESEASNDQAPVQMPRDYSEGASGLLRTGAKRD
```

sp|P21420|NMPC_ECOLI Putative outer membrane porin protein NmpC OS=Escherichia coli (strain K12) OX=83333 GN=nmpC PE=5 SV=2

```
0     MKKLTVAISAVAASVLMAMSAQAAEIYNKDSNKLDLYGKVNAKHYFSSND
                                 **********************
50    ADDGDTTYARLGFKGETQINDQLTGFGQWEYEFKGNRAESQGSSKDKTRL

100   AFAGLKFGDYGSIDYGRNYGVAYDIGAWTDVLPEFGGDTWTQTDVFMTQR

150   ATGVATYRNNDFFGLVDGLNFAAQYQGKNDRSDFDNYTEGNGDGFGFSAT

200   YEYEGFGIGATYAKSDRTDTQVNAGKVLPEVFASGKNAEVWAAGLKYDAN

250   NIYLATTYSETQNMTVFADHFVANKAQNFEAVAQYQFDFGLRPSVAYLQS

300   KGKDLGVWGDQDLVKYVDVGATYYFNKNMSTFVDYKINLLDKNDFTKALG

350   VSTDDIVAVGLVYQF
```

sp|P36943|EAEH_ECOLI Putative attaching and effacing protein homolog OS=Escherichia coli (strain K12) OX=83333 GN=eaeH PE=5 SV=2

```
0     MSHYKTGHKQPRFRYSVLARCVAWANISVQVLFPLAVTFTPVMAARAQHA

50    VQPRLSMGNTTVTADNNVEKNVASFAANAGTFLSSQPDSDATRNFITGMA
                      ****************************
100   TAKANQEIQEWLGKYGTARVKLNVDKDFSLKDSSLEMLYPIYDTPTNMLF

150   TQGAIHRTDDRTQSNIGFGWRHFSGNDWMAGVNTFIDHDLSRSHTRIGVG

200   AEYWRDYLKLSANGYIRASGWKKSPDIEDYQERPANGWDIRAEGYLPAWP

250   QLGASLMYEQYYGDEVGLFGKDKRQKDPHAISAEVTYTPVPLTQQ
```

sp|P16919|RHSD_ECOLI Protein RhsD OS=Escherichia coli (strain K12) OX=83333 GN=rhsD PE=2 SV=3

```
0     MSGKPAARQGDMTQYGGPIVQGSAGVRIGAPTGVACSVCPGGMTSGNPVN

50    PLLGAKVLPGETDLALPGPLPFILSRTYSSYRTKTPAPVGVFGPGWKAPS

100   DIRLQLRDDGLILNDNGGRSIHFEPLLPGEAVYSRSESMWLVRGGKAAQP

150   DGHTLARLWGALPPDIRLSPHLYLATNSAQGPWWILGWSERVPGAEDVLP

200   APLPPYRVLTGMADRFGRTLTYRREAAGDLAGEITGVTDGAGREFRLVLT

250   TQAQRAEEARTSSLSSSDSSRPLSASAFPDTLPGTEYGPDRGIRLSAVWL
```

300 MHDPAYPESLPAAPLVRYTYTEAGELLAVYDRSNTQVRAFTYDAQHPGRM

350 VAHRYAGRPEMRYRYDDTGRVVEQLNPAGLSYRYLYEQDRITVTDSLNRR

400 EVLHTEGGAGLKRVVKKELADGSVTRSGYDAAGRLTAQTDAAGRRTEYGL

450 NVVSGDITDITTPDGRETKFYYNDGNQLTAVVSPDGLESRREYDEPGRLV

500 SETSRSGETVRYRYDDAHSELPATTTDATGSTRQMTWSRYGQLLAFTDCS

550 GYQTRYEYDRFGQMTAVHREEGISLYRRYDNRGRLTSVKDAQGRETRYEY

600 NAAGDLTAVITPDGNRSETQYDAWGKAVSTTQGGLTRSMEYDAAGRVISL

650 TNENGSHSVFSYDALDRLVQQGGFDGRTQRYHYDLTGKLTQSEDEGLVIL

700 WYYDESDRITHRTVNGEPAEQWQYDGHGWLTDISHLSEGHRVAVHYGYDD

750 KGRLTGECQTVENPETGELLWQHETKHAYNEQGLANRVTPDSLPPVEWLT

800 YGSGYLAGMKLGGTPLVEYTRDRLHRETVRSFGSMAGSNAAYELTSTYTP

850 AGQLQSQHLNSLVYDRDYGWSDNGDLVRISGPRQTREYGYSATGRLESVR

900 TLAPDLDIRIPYATDPAGNRLPDPELHPDSTLTVWPDNRIAEDAHYVYRH

950 DEYGRLTEKTDRIPAGVIRTDDERTHHYHYDSQHRLVFYTRIQHGEPLVE

1000 SRYLYDPLGRRMAKRVWRRERDLTGWMSLSRKPEVTWYGWDGDRLTTVQT

1050 DTTRIQTVYEPGSFTPLIRVETENGEREKAQRRSLAETLQQEGSENGHGV

1100 VFPAELVRLLDRLEEEIRADRVSSESRAWLAQCGLTVEQLARQVEPEYTP

1150 ARKAHLYHCDHRGLPLALISEDGNTAWSAEYDEWGNQLNEENPHHVYQPY

1200 RLPGQQHDEESGLYYNRHRYYDPLQGRYITQDPMGLKGGWNLYQYPLNPL

1250 QQIDPMGLLQTWDDARSGACTGGVCGVLSRIIGPSKFDSTADAALDALKE

1300 TQNRSLCNDMEYSGIVCKDTNGKYFASKAETDNLRKESYPLKRKCPTGTD
****

1350 RVAAYHTHGADSHGDYVDEFFSSSDKNLVRSKDNNLEAFYLATPDGRFEA

1400 LNNKGEYIFIRNSVPGLSSVCIPYHD

sp|P11864|YHAC_ECOLI Uncharacterized protein YhaC OS=Escherichia coli (strain K12) OX=83333 GN=yhaC PE=4 SV=3

0 MFPVSSIGNDISSDLVRRKMNDLPESPTGNNLEALAPGIEKLKQTSIEMV

50 TLLNTLQPGGKCIITGDFQKELAYLQNVILYNVSSLRLDFLGYNAQIIQR

100 SDNTCELTINEPLKNQEISTGNININCPLKDIYNEIRRLNVIFSCGTGDI

150   VDLSSLDLRNVDLDYYDFTDKHMANTILNPFKLNSTNFTNANMFQVNFVS
                      ****
200   STQNATISWDYLLKITPVLISISDMYSEEKIKFVESCLNEPGDITEEQLK
                  ************************* ***********
250   IMRFAIIKSIPRATLTDKLENELTKEIYKSSSKIINCLNRIKLTEMKEFS
      ********************   *****************
300   SEKIYDYIDIIIEDYENTKENAYLVVPQINYTMDLNIEDSSSEELLSDNT
      ****
350   LEKDENSPDNGFEVGEYNTYEAYNSEKQYFTREDYTYDYDLLNAI

sp|P0ADP9|YIHD_ECOLI Protein YihD OS=Escherichia coli (strain K12) OX=83333 GN=yihD PE=1 SV=1

0   MKCKRLNEVIELLQPAWQKEPDLNLLQFLQKLAKESGFDGELADLTDDIL
             ****
50   IYHLKMRDSAKDAVIPGLQKDYEEDFKTALLRARGVIKE

sp|P77698|YBCK_ECOLI Uncharacterized protein YbcK OS=Escherichia coli (strain K12) OX=83333 GN=ybcK PE=4 SV=1

0   MKKAIAYMRFSSPGQMSGDSLNRQRRLIAEWLKVNSDYYLDTITYEDLGL

50   SAFKGKHAQSGAFSEFLDAIEHGYILPGTTLLVESLDRLSREKVGEAIER
                                                 ****
100   LKLILNHGIDVITLCDNTVYNIDSLNEPYSLIKAILIAQRANEESEIKSS
      ****
150   RVKLSWKKKRQDALESGTIMTASCPRWLSLDDKRTAFVPDPDRVKTIELI

200   FKLRMERRSLNAIAKYLNDHAVKNFSGKESAWGPSVIEKLLANKALIGIC

250   VPSYRARGKGISEIAGYYPRVISDDLFYAVQEIRLAPFGISNSSKNPMLI

300   NLLRTVMKCEACGNTMIVHAVSGSLHGYYVCPMRRLHRCDRPSIKRDLVD

350   YNIINELLFNCSKIQPVENKKDANETLELKIIELQMKINNLIVALSVAPE

400   VTAIAEKIRLLDKELRRASVSLKTLKSKGVNSFSDFYAIDLTSKNGRELC

450   RTLAYKTFEKIIINTDNKTCDIYFMNGIVFKHYPLMKVISAQQAISALKY

500   MVDGEIYF

sp|P76164|YDFW_ECOLI Protein YdfW OS=Escherichia coli (strain K12) OX=83333 GN=ydfW PE=4 SV=3
                                  ****
0   MDNARIDLRSKYYVKPKADHPWLTRRTQSHQQVKPPKLPKKKPDPDKKD

sp|P07464|THGA_ECOLI Galactoside O-acetyltransferase OS=Escherichia coli (strain K12) OX=83333 GN=lacA PE=1 SV=1
                                       ****
0   MNMPMTERIRAGKLFTDMCEGLPEKRLRGKTLMYEFNHSHPSEVEKRESL
      ****
50   IKEMFATVGENAWVEPPVYFSYGSNIHIGRNFYANFNLTIVDDYTVTIGD

```
100   NVLIAPNVTLSVTGHPVHHELRKNGEMYSFPITIGNNVWIGSHVVINPGV
                                       *****************
150   TIGDNSVIGAGSIVTKDIPPNVVAAGVPCRVIREINDRDKHYYFKDYKVE
      ***
200   SSV
```

sp|P0A8A2|YEEN_ECOLI Probable transcriptional regulatory protein YeeN OS=Escherichia coli (strain K12) OX=83333 GN=yeeN PE=1 SV=1

```
0     MGRKWANIVAKKTAKDGATSKIYAKFGVEIYAAAKQGEPDPELNTSLKFV

50    IERAKQAQVPKHVIDKAIDKAKGGGDETFVQGRYEGFGPNGSMIIAETLT

100   SNVNRTIANVRTIFNKKGGNIGAAGSVSYMFDNTGVIVFKGTDPDHIFEI

150   LLEAEVDVRDVTEEEGNIVIYTEPTDLHKGIAALKAAGITEFSTTELEMI
                 ********************
200   AQSEVELSPEDLEIFEGLVDALEDDDDVQKVYHNVANL
```

sp|P75785|OPGE_ECOLI Phosphoethanolamine transferase OpgE OS=Escherichia coli (strain K12) OX=83333 GN=opgE PE=1 SV=1

```
0     MNLTLKESLVTRSRVFSPWTAFYFLQSLLINLGLGYPFSLLYTAAFTAIL

50    LLLWRTLPRVQKVLVGVSSLVAACYFPFAQAYGAPNFNTLLALHSTNMEE

100   STEILTIFPWYSYLVGLFIFALGVIAIRRKKENEKARWNTFDSLCLVFSV

150   ATFFVAPVQNLAWGGVFKLKDTGYPVFRFAKDVIVNNNEVIEEQERMAKL

200   SGMKDTWTVTAVKPKYQTYVVVIGESARRDALGAFGGHWDNTPFASSVNG

250   LIFADYIAASGSTQKSLGLTLNRVVDGKPQFQDNFVTLANRAGFQTWWFS
                            ********************
300   NQGQIGEYDTAIASIAKRADEVYFLKEGNFEADKNTKDEALLDMTAQVLA

350   QEHSQPQLIVLHLMGSHPQACDRTQGKYETFVQSKETSCYLYTMTQTDDL

400   LRKLYDQLRNSGSSFSLVYFSDHGLAFKERGKDVQYLAHDDKYQQNFQVP

450   FMVISSDDKAHRVIKARRSANDFLGFFSQWTGIKAKEINIKYPFISEKKA

500   GPIYITNFQLQKVDYNHLGTDIFDPKP
```

sp|P62768|YAEH_ECOLI UPF0325 protein YaeH OS=Escherichia coli (strain K12) OX=83333 GN=yaeH PE=3 SV=1

```
0     MYDNLKSLGITNPEEIDRYSLRQEANNDILKIYFQKDKGEFFAKSVKFKY
                                              ***********
50    PRQRKTVVADGVGQGYKEVQEISPNLRYIIDELDQICQRDRSEVDLKRKI
      *********
100   LDDLRHLESVVTNKISEIEADLEKLTRK
```

sp|P37627|YHIJ_ECOLI Uncharacterized protein YhiJ OS=Escherichia coli (strain K12) OX=83333 GN=yhiJ PE=4 SV=1

```
0     MKIGTVAGTNDSTTTIATNDMVQEHVTNFTKELFGYIANGIGDDISSIAR
                               **********************
50    TMLGEVVEKIDDWQIERFQQSIQDDKISFTIQTDHSEKYSMLSGMRAHIL

100   RRNNNYQFIVTINSKNYGCSLDNTDINWCSIVYLLNNMTVNDNANDVAVT

150   ESYKPIWNWKISQYNVSDIKFETMIKPQFADRIYFSNCLPVDPTSTRPTY

200   FGDTDGSVGAVLFALFATGHLGIMAEGENFLSQLLNIEDEVLNVLLRENF

250   NEQLNTNVNTIISILNRRDIILESLQPYLVINKDAVTPCTFLGDQTGDRF

300   SNICGDQFIIDLLKRIMSINENVHVLAGNHETNCNGNYMQNFTRMKPLDE

350   DTYSGIKDYPVCFYDPKYKIMANHHGITFDDQRKRYIIGPITVSIDEMTN

400   ALDPVELAAIINKKHHAIINGKKFKTSRAISCRSFNRYFSVSTDYRPKLE

450   ALLACSQMLGINQVVAHNGNGGRERIGETGTVLGLNARDSKHAGRMFSMH

500   NCQINPGAGPEITTPWKSYQHEKNRNGLMPLIRRRTMLQL
```

sp|P0ACV0|LPXL_ECOLI Lipid A biosynthesis lauroyltransferase OS=Escherichia coli (strain K12) OX=83333 GN=lpxL PE=1 SV=1

```
0     MTNLPKFSTALLHPRYWLTWLGIGVLWLVVQLPYPVIYRLGCGLGKLALR

50    FMKRRAKIVHRNLELCFPEMSEQERRKMVVKNFESVGMGLMETGMAWFWP

100   DRRIARWTEVIGMEHIRDVQAQKRGILLVGIHFLTLELGARQFGMQEPGI
                                **********************
150   GVYRPNDNPLIDWLQTWGRLRSNKSMLDRKDLKGMIKALKKGEVVWYAPD

200   HDYGPRSSVFVPLFAVEQAATTTGTWMLARMSGACLVPFVPRRKPDGKGY

250   QLIMLPPECSPPLDDAETTAAWMNKVVEKCIMMAPEQYMWLHRRFKTRPE

300   GVPSRY
```

sp|P09546|PUTA_ECOLI Bifunctional protein PutA OS=Escherichia coli (strain K12) OX=83333 GN=putA PE=1 SV=3

```
0     MGTTTMGVKLDDATRERIKSAATRIDRTPHWLIKQAIFSYLEQLENSDTL

50    PELPALLSGAANESDEAPTPAEEPHQPFLDFAEQILPQSVSRAAITAAYR

100   RPETEAVSMLLEQARLPQPVAEQAHKLAYQLADKLRNQKNASGRAGMVQG

150   LLQEFSLSSQEGVALMCLAEALLRIPDKATRDALIRDKISNGNWQSHIGR

200   SPSLFVNAATWGLLFTGKLVSTHNEASLSRSLNRIIGKSGEPLIRKGVDM
```

250 AMRLMGEQFVTGETIAEALANARKLEEKGFRYSYDMLGEAALTAADAQAY

300 MVSYQQAIHAIGKASNGRGIYEGPGISIKLSALHPRYSRAQYDRVMEELY
****
350 PRLKSLTLLARQYDIGINIDAEESDRLEISLDLLEKLCFEPELAGWNGIG

400 FVIQAYQKRCPLVIDYLIDLATRSRRRLMIRLVKGAYWDSEIKRAQMDGL

450 EGYPVYTRKVYTDVSYLACAKKLLAVPNLIYPQFATHNAHTLAAIYQLAG

500 QNYYPGQYEFQCLHGMGEPLYEQVTGKVADGKLNRPCRIYAPVGTHETLL

550 AYLVRRLLENGANTSFVNRIADTSLPLDELVADPVTAVEKLAQQEGQTGL

600 PHPKIPLPRDLYGHGRDNSAGLDLANEHRLASLSSALLNSALQKWQALPM

650 LEQPVAAGEMSPVINPAEPKDIVGYVREATPREVEQALESAVNNAPIWFA

700 TPPAERAAILHRAAVLMESQMQQLIGILVREAGKTFSNAIAEVREAVDFL

750 HYYAGQVRDDFANETHRPLGPVVCISPWNFPLAIFTGQIAAALAAGNSVL

800 AKPAEQTPLIAAQGIAILLEAGVPPGVVQLLPGRGETVGAQLTGDDRVRG

850 VMFTGSTEVATLLQRNIASRLDAQGRPIPLIAETGGMNAMIVDSSALTEQ

900 VVVDVLASAFDSAGQRCSALRVLCLQDEIADHTLKMLRGAMAECRMGNPG

950 RLTTDIGPVIDSEAKANIERHIQTMRSKGRPVFQAVRENSEDAREWQSGT

1000 FVAPTLIELDDFAELQKEVFGPVLHVVRYNRNQLPELIEQINASGYGLTL

1050 GVHTRIDETIAQVTGSAHVGNLYVNRNMVGAVVGVQPFGGEGLSGTGPKA

1100 GGPLYLYRLLANRPESALAVTLARQDAKYPVDAQLKAALTQPLNALREWA

1150 ANRPELQALCTQYGELAQAGTQRLLPGPTGERNTWTLLPRERVLCIADDE

1200 QDALTQLAAVLAVGSQVLWPDDALHRQLVKALPSAVSERIQLAKAENITA

1250 QPFDAVIFHGDSDQLRALCEAVAARDGTIVSVQGFARGESNILLERLYIE

1300 RSLSVNTAAAGGNASLMTIG

sp|P04391|OTC1_ECOLI Ornithine carbamoyltransferase subunit I OS=Escherichia coli (strain K12) OX=83333 GN=argI PE=1 SV=3
****
0 MSGFYHKHFLKLLDFTPAELNSLLQLAAKLKADKKSGKEEAKLTGKNIAL

50 IFEKDSTRTRCSFEVAAYDQGARVTYLGPSGSQIGHKESIKDTARVLGRM

100 YDGIQYRGYGQEIVETLAEYASVPVWNGLTNEFHPTQLLADLLTMQEHLP

```
150   GKAFNEMTLVYAGDARNNMGNSMLEAAALTGLDLRLVAPQACWPEAALVT

200   ECRALAQQNGGNITLTEDVAKGVEGADFIYTDVWVSMGEAKEKWAERIAL

250   LREYQVNSKMMQLTGNPEVKFLHCLPAFHDDQTTLGKKMAEEFGLHGGME

300   VTDEVFESAASIVFDQAENRMHTIKAVMVATLSK
```

sp|P25798|FLIF_ECOLI Flagellar M-ring protein OS=Escherichia coli (strain K12) OX=83333 GN=fliF PE=1 SV=3

```
0     MNATAAQTKSLEWLNRLRANPKIPLIVAGSAAVAVMVALILWAKAPDYRT

50    LFSNLSDQDGGAIVSQLTQMNIPYRFSEASGAIEVPADKVHELRLRLAQQ

100   GLPKGGAVGFELLDQEKFGISQFSEQVNYQRALEGELSRTIETIGPVKGA

150   RVHLAMPKPSLFVREQKSPSASVTVNLLPGRALDEGQISAIVHLVSSAVA

200   GLPPGNVTLVDQGGHLLTQSNTSGRDLNDAQLKYASDVEGRIQRRIEAIL

250   SPIVGNGNIHAQVTAQLDFASKEQTEEQYRPNGDESHAALRSRQLNESEQ

300   SGSGYPGGVPGALSNQPAPANNAPISTPPANQNNRQQQASTTSNSGPRST

350   QRNETSNYEVDRTIRHTKMNVGDVQRLSVAVVVNYKTLPDGKPLPLSNEQ
                    *********************
400   MKQIEDLTREAMGFSEKRGDSLNVVNSPFNSSDESGGELPFWQQQAFIDQ

450   LLAAGRWLLVLLVAWLLWRKAVRPQLTRRAEAMKAVQQQAQAREEVEDAV

500   EVRLSKDEQLQQRRANQRLGAEVMSQRIREMSDNDPRVVALVIRQWINND

550   HE
```

sp|P0AEC8|DCUS_ECOLI Sensor histidine kinase DcuS OS=Escherichia coli (strain K12) OX=83333 GN=dcuS PE=1 SV=1

```
0     MRHSLPYRMLRKRPMKLSTTVILMVSAVLFSVLLVVHLIYFSQISDMTRD

50    GLANKALAVARTLADSPEIRQGLQKKPQESGIQAIAEAVRKRNDLLFIVV

100   TDMQSLRYSHPEAQRIGQPFKGDDILKALNGEENVAINRGFLAQALRVFT

150   PIYDENHKQIGVVAIGLELSRVTQQINDSRWSIIWSVLFGMLVGLIGTCI

200   LVKVLKKILFGLEPYEISTLFEQRQAMLQSIKEGVVAVDDRGEVTLINDA
        *******************************
250   AQELLNYRKSQDDEKLSTLSHSWSQVVDVSEVLRDGTPRRDEEITIKDRL

300   LLINTVPVRSNGVIIGAISTFRDKTEVRKLMQRLDGLVNYADALRERSHE

350   FMNKLHVILGLLHLKSYKQLEDYILKTANNYQEEIGSLLGKIKSPVIAGF
```

```
400   LISKINRATDLGHTLILNSESQLPDSGSEDQVATLITTLGNLIENALEAL

450   GPEPGGEISVTLHYRHGWLHCEVNDDGPGIAPDKIDHIFDKGVSTKGSER

500   GVGLALVKQQVENLGGSIAVESEPGIFTQFFVQIPWDGERSNR
```

sp|P56580|PTHB_ECOLI PTS system glucitol/sorbitol-specific EIIB component OS=Escherichia coli (strain K12) OX=83333 GN=srlE PE=1 SV=1

```
0     MTHIRIEKGTGGWGGPLELKATPGKKIVYITAGTRPAIVDKLAQLTGWQA

50    IDGFKEGEPAEAEIGVAVIDCGGTLRCGIYPKRRIPTINIHSTGKSGPLA
                                 **********************
100   QYIVEDIYVSGVKEENITVVGDATPQPSSVGRDYDTSKKITEQSDGLLAK

150   VGMGMGSTVAVLFQSGRDTIDTVLKTILPFMAFVSALIGIIMASGLGDWI

200   AHGLAPLASHPLGLVMLALICSFPLLSPFLGPGAVIAQVIGVLIGVQIGL

250   GNIPPHLALPALFAINAQAACDFIPVGLSLAEARQDTVRVGVPSVLVSRF

300   LTGAPTVLIAWFVSGFIYQ
```

sp|P0A707|IF3_ECOLI Translation initiation factor IF-3 OS=Escherichia coli (strain K12) OX=83333 GN=infC PE=1 SV=1

```
0     MKGGKRVQTARPNRINGEIRAQEVRLTGLEGEQLGIVSLREALEKAEEAG
                   *************************************
50    VDLVEISPNAEPPVCRIMDYGKFLYEKSKSSKEQKKKQKVIQVKEIKFRP

100   GTDEGDYQVKLRSLIRFLEEGDKAKITLRFRGREMAHQQIGMEVLNRVKD

150   DLQELAVVESFPTKIEGRQMIMVLAPKKKQ
```

sp|P0A9K7|PHOU_ECOLI Phosphate-specific transport system accessory protein PhoU OS=Escherichia coli (strain K12) OX=83333 GN=phoU PE=1 SV=1

```
0     MDSLNLNKHISGQFNAELESIRTQVMTMGGMVEQQLSDAITAMHNQDSDL

50    AKRVIEGDKNVNMMEVAIDEACVRIIAKRQPTASDLRLVMVISKTIAELE
                                                     *
100   RIGDVADKICRTALEKFSQQHQPLLVSLESLGRHTIQMLHDVLDAFARMD
      *******************
150   IDEAVRIYREDKKVDQEYEGIVRQLMTYMMEDSRTIPSVLTALFCARSIE

200   RIGDRCQNICEFIFYYVKGQDFRHVGGDELDKLLAGKDSDK
```

sp|P00936|CYAA_ECOLI Adenylate cyclase OS=Escherichia coli (strain K12) OX=83333 GN=cyaA PE=1 SV=5

```
0     MYLYIETLKQRLDAINQLRVDRALAAMGPAFQQVYSLLPTLLHYHHPLMP

50    GYLDGNVPKGICLYTPDETQRHYLNELELYRGMSVQDPPKGELPITGVYT
```

```
100   MGSTSSVGQSCSSDLDIWVCHQSWLDSEERQLLQRKCSLLENWAASLGVE

150   VSFFLIDENRFRHNESGSLGGEDCGSTQHILLLDEFYRTAVRLAGKRILW

200   NMVPCDEEEHYDDYVMTLYAQGVLTPNEWLDLGGLSSLSAEEYFGASLWQ

250   LYKSIDSPYKAVLKTLLLEAYSWEYPNPRLLAKDIKQRLHDGEIVSFGLD

300   PYCMMLERVTEYLTAIEDFTRLDLVRRCFYLKVCEKLSRERACVGWRRAV

350   LSQLVSEWGWDEARLAMLDNRANWKIDQVREAHNELLDAMMQSYRNLIRF

400   ARRNNLSVSASPQDIGVLTRKLYAAFEALPGKVTLVNPQISPDLSEPNLT

450   FIYVPPGRANRSGWYLYNRAPNIESIISHQPLEYNRYLNKLVAWAWFNGL

500   LTSRTRLYIKGNGIVDLPKLQEMVADVSHHFPLRLPAPTPKALYSPCEIR

550   HLAIIVNLEYDPTAAFRNQVVHFDFRKLDVFSFGENQNCLVGSVDLLYRN

600   SWNEVRTLHFNGEQSMIEALKTILGKMHQDAAPPDSVEVFCYSQHLRGLI

650   RTRVQQLVSECIELRLSSTRQETGRFKALRVSGQTWGLFFERLNVSVQKL

700   ENAIEFYGAISHNKLHGLSVQVETNHVKLPAVVDGFASEGIIQFFFEETQ
                          ************************
750   DENGFNIYILDESNRVEVYHHCEGSKEELVRDVSRFYSSSHDRFTYGSSF

800   INFNLPQFYQIVKVDGREQVIPFRTKSIGNMPPANQDHDTPLLQQYFS
```

sp|P17846|CYSI_ECOLI Sulfite reductase [NADPH] hemoprotein beta-component OS=Escherichia coli (strain K12) OX=83333 GN=cysI PE=1 SV=4

```
0     MSEKHPGPLVVEGKLTDAERMKHESNYLRGTIAEDLNDGLTGGFKGDNFL

50    LIRFHGMYQQDDRDIRAERAEQKLEPRHAMLLRCRLPGGVITTKQWQAID

100   KFAGENTIYGSIRLTNRQTFQFHGILKKNVKPVHQMLHSVGLDALATAND

150   MNRNVLCTSNPYESQLHAEAYEWAKKISEHLLPRTRAYAEIWLDQEKVAT

200   TDEEPILGQTYLPRKFKTTVVIPPQNDIDLHANDMNFVAIAENGKLVGFN

250   LLVGGGLSIEHGNKKTYARTASEFGYLPLEHTLAVAEAVVTTQRDWGNRT

300   DRKNAKTKYTLERVGVETFKAEVERRAGIKFEPIRPYEFTGRGDRIGWVK
                                                        **
350   GIDDNWHLTLFIENGRILDYPARPLKTGLLEIAKIHKGDFRITANQNLII
      ************************
400   AGVPESEKAKIEKIAKESGLMNAVTPQRENSMACVSFPTCPLAMAEAERF

450   LPSFIDNIDNLMAKHGVSDEHIVMRVTGCPNGCGRAMLAEVGLVGKAPGR

500   YNLHLGGNRIGTRIPRMYKENITEPEILASLDELIGRWAKEREAGEGFGD
```

550   FTVRAGIIRPVLDPARDLWD

sp|P45423|YHCG_ECOLI Putative nuclease YhcG OS=Escherichia coli (strain K12) OX=83333 GN=yhcG PE=1 SV=1

0   MESLSEGTTAGYQQIHDGIIHLVDSARTETVRSVNALMTATYQEIGRRIV

50   EFEQGGEARAAYGAQLIKRLSKDLCLRYKRGFSAKNLRQMRLFYLFFQHV

100   EIHQTMSGELTPLGIPQTPSAEFPSAKIWQTLSAKSFPLPRSTYVRLLSV

150   KNADARSFYEKETLRCGWSVRQLERQIATQFYERTLLSHDKSAMLQQHAP
                ****
200   AETHILPQQAIRDPFVLEFLELKDEYSESDFEEALINHLMDFMLELGDDF

250   AFVGRQRRLRIDDNWFRVDLLFFHRRLRCLLIVDLKVGKFSYSDAGQMNM

300   YLNYAKEHWTLPDENPPIGLVLCAEKGAGEAHYALAGLPNTVLASEYKMQ

350   LPDEKRLADELVRTQAVLEEGYRRR

sp|P0ADS9|YGGN_ECOLI Uncharacterized protein YggN OS=Escherichia coli (strain K12) OX=83333 GN=yggN PE=4 SV=1

0   MMRKMLLAAALSVTAMTAHADYQCSVTPRDDVIVSPQTVQVKGENGNLVI

50   TPDGNVMYNGKQYSLNAAQREQAKDYQAELRSTLPWIDEGAKSRVEKARI

100   ALDKIIVQEMGESSKMRSRLTKLDAQLKEQMNRIIETRSDGLTFHYKAID
                                                       *
150   QVRAEGQQLVNQAMGGILQDSINEMGAKAVLKSGGNPLQNVLGSLGGLQS
      ****
200   SIQTEWKKQEKDFQQFGKDVCSRVVTLEDSRKALVGNLK

sp|P37680|SGBE_ECOLI L-ribulose-5-phosphate 4-epimerase SgbE OS=Escherichia coli (strain K12) OX=83333 GN=sgbE PE=1 SV=1

0   MLEQLKADVLAANLALPAHHLVTFTWGNVSAVDETRQWMVIKPSGVEYDV
            ****
50   MTADDMVVVEIASGKVVEGSKKPSSDTPTHLALYRRYAEIGGIVHTHSRH

100   ATIWSQAGLDLPAWGTTHADYFYGAIPCTRQMTAEEINGEYEYQTGEVII

150   ETFEERGRSPAQIPAVLVHSHGPFAWGKNAADAVHNAVVLEECAYMGLFS

200   RQLAPQLPAMQNELLDKHYLRKHGANAYYGQ

sp|P46853|YHHX_ECOLI Uncharacterized oxidoreductase YhhX OS=Escherichia coli (strain K12) OX=83333 GN=yhhX PE=1 SV=1

0   MVINCAFIGFGKSTTRYHLPYVLNRKDSWHVAHIFRRHAKPEEQAPIYSH

50   IHFTSDLDEVLNDPDVKLVVVCTHADSHFEYAKRALEAGKNVLVEKPFTP

****
100 TLAQAKELFALAKSKGLTVTPYQNRRFDSCFLTAKKAIESGKLGEIVEVE
****
150 SHFDYYRPVAETKPGLPQDGAFYGLGVHTMDQIISLFGRPDHVAYDIRSL

200 RNKANPDDTFEAQLFYGDLKAIVKTSHLVKIDYPKFIVHGKKGSFIKYGI

250 DQQETSLKANIMPGEPGFAADDSVGVLEYVNDEGVTVREEMKPEMGDYGR

300 VYDALYQTITHGAPNYVKESEVLTNLEILERGFEQASPSTVTLAK

sp|P15877|DHG_ECOLI Quinoprotein glucose dehydrogenase OS=Escherichia coli (strain K12) OX=83333 GN=gcd PE=1 SV=3

0 MAINNTGSRRLLVTLTALFAALCGLYLLIGGGWLVAIGGSWYYPIAGLVM

50 LGVAWMLWRSKRAALWLYAALLLGTMIWGVWEVGFDFWALTPRSDILVFF

100 GIWLILPFVWRRLVIPASGAVAALVVALLISGGILTWAGFNDPQEINGTL

150 SADATPAEAISPVADQDWPAYGRNQEGQRFSPLKQINADNVHNLKEAWVF
****
200 RTGDVKQPNDPGEITNEVTPIKVGDTLYLCTAHQRLFALDAASGKEKWHY
****
250 DPELKTNESFQHVTCRGVSYHEAKAETASPEVMADCPRRIILPVNDGRLI

300 AINAENGKLCETFANKGVLNLQSNMPDTKPGLYEPTSPPIITDKTIVMAG

350 SVTDNFSTRETSGVIRGFDVNTGELLWAFDPGAKDPNAIPSDEHTFTFNS

400 PNSWAPAAYDAKLDLVYLPMGVTTPDIWGGNRTPEQERYASSILALNATT

450 GKLAWSYQTVHHDLWDMDLPAQPTLADITVNGQKVPVIYAPAKTGNIFVL

500 DRRNGELVVPAPEKPVPQGAAKGDYVTPTQPFSELSFRPTKDLSGADMWG

550 ATMFDQLVCRVMFHQMRYEGIFTPPSEQGTLVFPGNLGMFEWGGISVDPN

600 REVAIANPMALPFVSKLIPRGPGNPMEQPKDAKGTGTESGIQPQYGVPYG

650 VTLNPFLSPFGLPCKQPAWGYISALDLKTNEVVWKKRIGTPQDSMPFPMP

700 VPVPFNMGMPMLGGPISTAGNVLFIAATADNYLRAYNMSNGEKLWQGRLP

750 AGGQATPMTYEVNGKQYVVISAGGHGSFGTKMGDYIVAYALPDDVK

sp|P14294|TOP3_ECOLI DNA topoisomerase 3 OS=Escherichia coli (strain K12) OX=83333 GN=topB PE=1 SV=1

0 MRLFIAEKPSLARAIADVLPKPHRKGDGFIECGNGQVVTWCIGHLLEQAQ

50 PDAYDSRYARWNLADLPIVPEKWQLQPRPSVTKQLNVIKRFLHEASEIVH

100 AGDPDREGQLLVDEVLDYLQLAPEKRQQVQRCLINDLNPQAVERAIDRLR

150 SNSEFVPLCVSALARARADWLYGINMTRAYTILGRNAGYQGVLSVGRVQT

200 PVLGLVVRRDEEIENFVAKDFFEVKAHIVTPADERFTAIWQPSEACEPYQ

250 DEEGRLLHRPLAEHVVNRISGQPAIVTSYNDKRESESAPLPFSLSALQIE

300 AAKRFGLSAQNVLDICQKLYETHKLITYPRSDCRYLPEEHFAGRHAVMNA

350 ISVHAPDLLPQPVVDPDIRNRCWDDKKVDAHHAIIPTARSSAINLTENEA

400 KVYNLIARQYLMQFCPDAVFRKCVIELDIAKGKFVAKARFLAEAGWRTLL

450 GSKERDEENDGTPLPVVAKGDELLCEKGEVVERQTQPPRHFTDATLLSAM

500 TGIARFVQDKDLKKILRATDGLGTEATRAGIIELLFKRGFLTKKGRYIHS

550 TDAGKALFHSLPEMATRPDMTAHWESVLTQISEKQCRYQDFMQPLVGTLY
****
600 QLIDQAKRTPVRQFRGIVAPGSGGSADKKKAAPRKRSAKKSPPADEVGSG

650 AIA

sp|P64559|SDHE_ECOLI FAD assembly factor SdhE OS=Escherichia coli (strain K12) OX=83333 GN=sdhE PE=1 SV=1
****
0 MDINNKARIHWACRRGMRELDISIMPFFEHEYDSLSDDEKRIFIRLLECD
***
50 DPDLFNWLMNHGKPADAELEMMVRLIQTRNRERGPVAI

sp|P77759|YLBH_ECOLI Putative uncharacterized protein YlbH OS=Escherichia coli (strain K12) OX=83333 GN=ylbH PE=5 SV=1

0 MLALMDADGNIAWSGEYDEWGNQLNEENPHHLHQPYRLPGQQYDKESGLY

50 YNRNRYYDPLQGRYITQDPIGLEGGWSLYAYPLNPVNGIDPLGLSPADVA

100 LIRRKDQLNHQRAWDILSDTYEDMKRLNLGGTDQFFHCMAFCRVSKLNDA
****
150 GVSRSAKGLGYEKEIRDYGLNLFGMYGRKVKLSHSEMIEDNKKDLAVNDH

200 GLTCPSTTDCSDRCSDYINPEHKKTIKALQDAGYLK

sp|P0A6L2|DAPA_ECOLI 4-hydroxy-tetrahydrodipicolinate synthase OS=Escherichia coli (strain K12) OX=83333 GN=dapA PE=1 SV=1

0 MFTGSIVAIVTPMDEKGNVCRASLKKLIDYHVASGTSAIVSVGTTGESAT

50 LNHDEHADVVMMTLDLADGRIPVIAGTGANATAEAISLTQRFNDSGIVGC

100 LTVTPYYNRPSQEGLYQHFKAIAEHTDLPQILYNVPSRTGCDLLPETVGR
****
150 LAKVKNIIGIKEATGNLTRVNQIKELVSDDFVLLSGDDASALDFMQLGGH

200   GVISVTANVAARDMAQMCKLAAEGHFAEARVINQRLMPLHNKLFVEPNPI

250   PVKWACKELGLVATDTLRLPMTPITDSGRETVRAALKHAGLL

sp|P76491|5DNU_ECOLI 5'-deoxynucleotidase YfbR OS=Escherichia coli (strain K12) OX=83333 GN=yfbR PE=1 SV=1

0   MKQSHFFAHLSRLKLINRWPLMRNVRTENVSEHSLQVAMVAHALAAIKNR

50   KFGGNVNAERIALLAMYHDASEVLTGDLPTPVKYFNSQIAQEYKAIEKIA
                       ****
100   QQKLVDMVPEELRDIFAPLIDEHAYSDEEKSLVKQADALCAYLKCLEELA

150   AGNNEFLLAKTRLEATLEARRSQEMDYFMEIFVPSFHLSLDEISQDSPL

sp|P77136|YQEK_ECOLI Uncharacterized protein YqeK OS=Escherichia coli (strain K12) OX=83333 GN=yqeK PE=4 SV=3

0   MDIEFSQIHEMVYMHDIVNSDSKKKPRIPLKKFLNAENVLTQTTSWTLNS
               ****
50   RYVNVNSVNKVNVKSKVKNSYISRSVNDEFSLTDDEINSFKETLVLSSID
      *****
100   SLSKLVLNNPLSVLFTSTVRRNNNRAKMNVEFDSWICTRCC

sp|P08178|PUR5_ECOLI Phosphoribosylformylglycinamidine cyclo-ligase OS=Escherichia coli (strain K12) OX=83333 GN=purM PE=1 SV=3

0   MTDKTSLSYKDAGVDIDAGNALVGRIKGVVKKTRRPEVMGGLGGFGALCA

50   LPQKYREPVLVSGTDGVGTKLRLAMDLKRHDTIGIDLVAMCVNDLVVQGA

100   EPLFFLDYYATGKLDVDTASAVISGIAEGCLQSGCSLVGGETAEMPGMYH
               ****
150   GEDYDVAGFCVGVVEKSEIIDGSKVSDGDVLIALGSSGPHSNGYSLVRKI

200   LEVSGCDPQTTELDGKPLADHLLAPTRIYVKSVLELIEKVDVHAIAHLTG

250   GGFWENIPRVLPDNTQAVIDESSWQWPEVFNWLQTAGNVEHHEMYRTFNC

300   GVGMIIALPAPEVDKALALLNANGENAWKIGIIKASDSEQRVVIE

sp|P0A850|TIG_ECOLI Trigger factor OS=Escherichia coli (strain K12) OX=83333 GN=tig PE=1 SV=1

0   MQVSVETTQGLGRRVTITIAADSIETAVKSELVNVAKKVRIDGFRKGKVP

50   MNIVAQRYGASVRQDVLGDLMSRNFIDAIIKEKINPAGAPTYVPGEYKLG

100   EDFTYSVEFEVYPEVELQGLEAIEVEKPIVEVTDADVDGMLDTLRKQQAT

150   WKEKDGAVEAEDRVTIDFTGSVDGEEFEGGKASDFVLAMGQGRMIPGFED

200   GIKGHKAGEEFTIDVTFPEEYHAENLKGKAAKFAINLKKVEERELPELTA

```
250   EFIKRFGVEDGSVEGLRAEVRKNMERELKSAIRNRVKSQAIEGLVKANDI

300   DVPAALIDSEIDVLRRQAAQRFGGNEKQALELPRELFEEQAKRRVVVGLL
                ************************************** 
350   LGEVIRTNELKADEERVKGLIEEMASAYEDPKEVIEFYSKNKELMDNMRN

400   VALEEQAVEAVLAKAKVTEKETTFNELMNQQA
```

sp|P76655|YQIG_ECOLI Putative outer membrane usher protein YqiG OS=Escherichia coli (strain K12) OX=83333 GN=yqiG PE=5 SV=3

```
0     MSGNIGANPVIIIGCASAYAVEFNKDLIEAEDRENVNLSQFETDGQLPVG

50    KYSLSTLINNKRTPIHLDLQWVLIDNQTAVCVTPEQLTLLGFTDEFIEKT

100   QQNLIDGCYPIEKEKQITTYLDKGKMQLSISAPQAWLKYKDANWTPPELW

150   NHGIAGAFLDYNLYASHYAPHQGDNSQNISSYGQAGVNLGAWRLRTDYQY
                                          **************
200   DQSFNNGKSQATNLDFPRIYLFRPIPAMNAKLTIGQYDTESSIFDSFHFS
      **********
250   GISLKSDENMLPPDLRGYAPQITGVAQTNAKVTVSQNNRIIYQENVPPGP

300   FAITNLFNTLQGQLDVKVEEEDGRVTQWQVASNSIPYLTRKGQIRYTTAM

350   GKPTSVGGDSLQQPFFWTGEFSWGWLNNVSLYGGSVLTNRDYQSLAAGVG

400   FNLNSLGSLSFDVTRSDAQLHNQDKETGYSYRANYSKRFESTGSQLTFAG

450   YRFSDKNFVTMNEYINDTNHYTNYQNEKESYIVTFNQYLESLRLNTYVSL

500   ARNTYWDASSNVNYSLSLSRDFDIGPLKNVSTSLTFSRINWEEDNQDQLY
                                       *****************
550   LNISIPWGTSRTLSYGMQRNQDNEISHTASWYDSSDRNNSWSVSASGDND
      *************
600   EFKDMKASLRASYQHNTENGRLYLSGTSQRDSYYSLNASWNGSFTATRHG

650   AAFHDYSGSADSRFMIDADGTEDIPLNNKRAVTNRYGIGVIPSVSSYITT

700   SLSVDTRNLPENVDIENSVITTTLTEGAIGYAKLDTRKGYQIIGVIRLAD

750   GSHPPLGISVKDETSHKELGLVADGGFVYLNGIQDDNKLALRWGDKSCFI

800   QPPNSSNLTTGTAILPCISQN
```

sp|P55140|YGCG_ECOLI UPF0603 protein YgcG OS=Escherichia coli (strain K12) OX=83333 GN=ygcG PE=3 SV=2

```
                                   *********************
0     MRYFILMFTFVCSFVAAQPTIVPQLQQQVTDLTSSLNSQEKKELTHKLES
      ***
50    IFNNTQVQIAVLIVPTTKDETIEQYATRVFDNWRLGDAKRNDGILIVVAW

100   SDRTVRIQVGYGLEEKVTDALAGDIIRSNMIPAFKQQKLAKGLELAINAL
```

150   NNQLTSQHQYPTNPSESESASSSDHYYFAIFWVFAVMFFPFWFFHQGSNF

200   CRACKSGVCISAIYLLDLFLFSDKIFSIAVFSFFFTFTIFMVFTCLCVLQ

250   KRASGRSYHSDNSGSAGGSDSGGFSGGGGSSGGGGASGRW

sp|P00961|SYGB_ECOLI Glycine--tRNA ligase beta subunit OS=Escherichia coli (strain K12) OX=83333 GN=glyS PE=1 SV=4

0   MSEKTFLVEIGTEELPPKALRSLAESFAANFTAELDNAGLAHGTVQWFAA

50   PRRLALKVANLAEAQPDREIEKRGPAIAQAFDAEGKPSKAAEGWARGCGI

100   TVDQAERLTTDKGEWLLYRAHVKGESTEALLPNMVATSLAKLPIPKLMRW

150   GASDVHFVRPVHTVTLLLGDKVIPATILGIQSDRVIRGHRFMGEPEFTID
                  ****
200   NADQYPEILRERGKVIADYEERKAKIKADAEEAARKIGGNADLSESLLEE

250   VASLVEWPVVLTAKFEEKFLAVPAEALVYTMKGDQKYFPVYANDGKLLPN

300   FIFVANIESKDPQQIISGNEKVVRPRLADAEFFFNTDRKKRLEDNLPRLQ

350   TVLFQQQLGTLRDKTDRIQALAGWIAEQIGADVNHATRAGLLSKCDLMTN

400   MVFEFTDTQGVMGMHYARHDGEAEDVAVALNEQYQPRFAGDDLPSNPVAC

450   ALAIADKMDTLAGIFGIGQHPKGDKDPFALRRAALGVLRIIVEKNLNLDL

500   QTLTEEAVRLYGDKLTNANVVDDVIDFMLGRFRAWYQDEGYTVDTIQAVL
                                                ****
550   ARRPTRPADFDARMKAVSHFRTLDAAAALAAANKRVSNILAKSDEVLSDR
      ****
600   VNASTLKEPEEIKLAMQVVVLRDKLEPYFTEGRYQDALVELAELREPVDA

650   FFDKVMVMVDDKELRINRLTMLEKLRELFLRVADISLLQ

sp|P36938|PGM_ECOLI Phosphoglucomutase OS=Escherichia coli (strain K12) OX=83333 GN=pgm PE=1 SV=1

0   MAIHNRAGQPAQQSDLINVAQLTAQYYVLKPEAGNAEHAVKFGTSGHRGS

50   AARHSFNEPHILAIAQAIAEERAKNGITGPCYVGKDTHALSEPAFISVLE

100   VLAANGVDVIVQENNGFTPTPAVSNAILVHNKKGGPLADGIVITPSHNPP

150   EDGGIKYNPPNGGPADTNVTKVVEDRANALLADGLKGVKRISLDEAMASG

200   HVKEQDLVQPFVEGLADIVDMAAIQKAGLTLGVDPLGGSGIEYWKRIGEY

250   YNLNLTIVNDQVDQTFRFMHLDKDGAIRMDCSSECAMAGLLALRDKFDLA

300   FANDPDYDRHGIVTPAGLMNPNHYLAVAINYLFQHRPQWGKDVAVGKTLV

350   SSAMIDRVVNDLGRKLVEVPVGFKWFVDGLFDGSFGFGGEESAGASFLRF

400   DGTPWSTDKDGIIMCLLAAEITAVTGKNPQEHYNELAKRFGAPSYNRLQA

450   AATSAQKAALSKLSPEMVSASTLAGDPITARLTAAPGNGASIGGLKVMTD
                                ****
500   NGWFAARPSGTEDAYKIYCESFLGEEHRKQIEKEAVEIVSEVLKNA

sp|P62707|GPMA_ECOLI 2,3-bisphosphoglycerate-dependent phosphoglycerate mutase OS=Escherichia coli (strain K12) OX=83333 GN=gpmA PE=1 SV=2
                                  ****
0   MAVTKLVLVRHGESQWNKENRFTGWYDVDLSEKGVSEAKAAGKLLKEEGY
      *
50   SFDFAYTSVLKRAIHTLWNVLDELDQAWLPVEKSWKLNERHYGALQGLNK
                                   ****
100   AETAEKYGDEQVKQWRRGFAVTPPELTKDDERYPGHDPRYAKLSEKELPL

150   TESLALTIDRVIPYWNETILPRMKSGERVIIAAHGNSLRALVKYLDNMSE

200   EEILELNIPTGVPLVYEFDENFKPLKRYYLGNADEIAAKAAAVANQGKAK

250

sp|P32685|YJBD_ECOLI Uncharacterized protein YjbD OS=Escherichia coli (strain K12) OX=83333 GN=yjbD PE=4 SV=1
                                           ****
0   MALPRITQKEMTEREQRELKTLLDRARIAHGRVLTNSETNSIKKDYIDKL
      ****
50   MVEREAEAKKARQLKKKQAYKPDPEASFSWSANTSTRGRR

sp|P76569|YFGD_ECOLI Uncharacterized protein YfgD OS=Escherichia coli (strain K12) OX=83333 GN=yfgD PE=1 SV=1

0   MTKQVKIYHNPRCSKSRETLNLLKENGVEPEVVLYLETPADAATLRDLLK
                ****
50   ILGMNSARELMRQKEDLYKELNLADSSLSEEALIQAMVDNPKLMERPIVV

100   ANGKARIGRPPEQVLEIVG

sp|P52614|FLIK_ECOLI Flagellar hook-length control protein OS=Escherichia coli (strain K12) OX=83333 GN=fliK PE=3 SV=1

0   MIRLAPLITADVDTTTLPGGKASDAAQDFLALLSEALAGETTTDKAAPQL

50   LVATDKPTTKGEPLISDIVSDAQQANLLIPVDETPPVINDEQSTSTPLTT
            ****
100   AQTMALAAVADKNTTKDEKADDLNEDVTASLSALFAMLPGFDNTPKVTDA

150   PSTVLPTEKPTLFTKLTSEQLTTAQPDDAPGTPAQPLTPLVAEAQSKAEV

200   ISTPSPVTAAASPLITPHQTQPLPTVAAPVLSAPLGSHEWQQSLSQHISL

250   FTRQGQQSAELRLHPQDLGEVQISLKVDDNQAQIQMVSPHQHVRAALEAA

300   LPVLRTQLAESGIQLGQSNISGESFSGQQQAASQQQQSQRTANHEPLAGE

350   DDDTLPVPVSLQGRVTGNSGVDIFA

sp|P0ACW6|YDCH_ECOLI Uncharacterized protein YdcH OS=Escherichia coli (strain K12) OX=83333 GN=ydcH PE=4 SV=2
                          ****
0   MFPEYRDLISRLKNENPRFMSLFDKHNKLDHEIARKEGSDGRGYNAEVVR

50   MKKQKLQLKDEMLKILQQESVKEV

sp|P64448|YNBE_ECOLI Uncharacterized protein YnbE OS=Escherichia coli (strain K12) OX=83333 GN=ynbE PE=4 SV=1
                                         ****
0   MKILLAALTSSFMLVGCTPRIEVAAPKEPITINMNVKIEHEIIIKADKDV
      ****
50   EELLETRSDLF

sp|P77735|YAJO_ECOLI 1-deoxyxylulose-5-phosphate synthase YajO OS=Escherichia coli (strain K12) OX=83333 GN=yajO PE=1 SV=2

0   MQYNPLGKTDLRVSRLCLGCMTFGEPDRGNHAWTLPEESSRPIIKRALEG

50   GINFFDTANSYSDGSSEEIVGRALRDFARREDVVVATKVFHRVGDLPEGL

100   SRAQILRSIDDSLRRLGMDYVDILQIHRWDYNTPIEETLEALNDVVKAGK

150   ARYIGASSMHASQFAQALELQKQHGWAQFVSMQDHYNLIYREEEREMLPL
                                 ****
200   CYQEGVAVIPWSPLARGRLTRPWGETTARLVSDEVGKNLYKESDENDAQI
      ****
250   AERLTGVSEELGATRAQVALAWLLSKPGIAAPIIGTSREEQLDELLNAVD

300   ITLKPEQIAELETPYKPHPVVGFK

sp|P33940|MQO_ECOLI Malate:quinone oxidoreductase OS=Escherichia coli (strain K12) OX=83333 GN=mqo PE=1 SV=2

0   MKKVTAMLFSMAVGLNAVSMAAKAKASEEQETDVLLIGGGIMSATLGTYL

50   RELEPEWSMTMVERLEGVAQESSNGWNNAGTGHSALMELNYTPQNADGSI

100   SIEKAVAINEAFQISRQFWAHQVERGVLRTPRSFINTVPHMSFVWGEDNV

150   NFLRARYAALQQSSLFRGMRYSEDHAQIKEWAPLVMEGRDPQQKVAATRT

200   EIGTDVNYGEITRQLIASLQKKSNFSLQLSSEVRALKRNDDNTWTVTVAD

250   LKNGTAQNIRAKFVFIGAGGAALKLLQESGIPEAKDYAGFPVGGQFLVSE

300   NPDVVNHHLAKVYGKASVGAPPMSVPHIDTRVLDGKRVVLFGPFATFSTK
                                               ****
350   FLKNGSLWDLMSSTTTSNVMPMMHVGLDNFDLVKYLVSQVMLSEEDRFEA
      ****

400   LKEYYPQAKKEDWRLWQAGQRVQIIKRDAEKGGVLRLGTEVVSDQQGTIA

450   ALLGASPGASTAAPIMLNLLEKVFGDRVSSPQWQATLKAIVPSYGRKLNG

500   DVAATERELQYTSEVLGLNYDKPQAADSTPKPQLKPQPVQKEVADIAL

sp|P0ADE4|TAMA_ECOLI Translocation and assembly module subunit TamA OS=Escherichia coli (strain K12) OX=83333 GN=tamA PE=1 SV=1

0   MRYIRQLCCVSLLCLSGSAVAANVRLQVEGLSGQLEKNVRAQLSTIESDE

50   VTPDRRFRARVDDAIREGLKALGYYQPTIEFDLRPPPKKGRQVLIAKVTP

100   GVPVLIGGTDVVLRGGARTDKDYLKLLDTRPAIGTVLNQGDYENFKKSLT

150   SIALRKGYFDSEFTKAQLGIALGLHKAFWDIDYNSGERYRFGHVTFEGSQ
        ****
200   IRDEYLQNLVPFKEGDEYESKDLAELNRRLSATGWFNSVVVAPQFDKARE

250   TKVLPLTGVVSPRTENTIETGVGYSTDVGPRVKATWKKPWMNSYGHSLTT

300   STSISAPEQTLDFSYKMPLLKNPLEQYYLVQGGFKRTDLNDTESDSTTLV

350   ASRYWDLSSGWQRAINLRWSLDHFTQGEITNTTMLFYPGVMISRTRSRGG

400   LMPTWGDSQRYSIDYSNTAWGSDVDFSVFQAQNVWIRTLYDRHRFVTRGT

450   LGWIETGDFDKVPPDLRFFAGGDRSIRGYKYKSIAPKYANGDLKGASKLI

500   TGSLEYQYNVTGKWWGAVFVDSGEAVSDIRRSDFKTGTGVGVRWESPVGP

550   IKLDFAVPVADKDEHGLQFYIGLGPEL

sp|P76083|PAAH_ECOLI 3-hydroxyadipyl-CoA dehydrogenase OS=Escherichia coli (strain K12) OX=83333 GN=paaH PE=1 SV=1

0   MMINVQTVAVIGSGTMGAGIAEVAASHGHQVLLYDISAEALTRAIDGIHA

50   RLNSRVTRGKLTAETCERTLKRLIPVTDIHALAAADLVIEAASERLEVKK

100   ALFAQLAEVCPPQTLLTTNTSSISITAIAAEIKNPERVAGLHFFNPAPVM

150   KLVEVVSGLATAAEVVEQLCELTLSWGKQPVRCHSTPGFIVNRVARPYYS

200   EAWRALEEQVAAPEVIDAALRDGAGFPMGPLELTDLIGQDVNFAVTCSVF
                                               ****
250   NAFWQERRFLPSLVQQELVIGGRLGKKSGLGVYDWRAEREAVVGLEAVSD
      ****
300   SFSPMKVEKKSDGVTEIDDVLLIETQGETAQALAIRLARPVVVIDKMAGK

350   VVTIAAAAVNPDSATRKAIYYLQQQGKTVLQIADYPGMLIWRTVAMIINE

400   ALDALQKGVASEQDIDTAMRLGVNYPYGPLAWGAQLGWQRILRLLENLQH

450   HYGEERYRPCSLLRQRALLESGYES

sp|P0ACY9|YEBG_ECOLI Uncharacterized protein YebG OS=Escherichia coli (strain K12) OX=83333 GN=yebG PE=4 SV=1

```
         *************************************
0     MAVEVKYVVIREGEEKMSFTSKKEADAYDKMLDTADLLDTWLTNSPVQME

50    DEQREALSLWLAEQKDVLSTILKTGKLPSPQVVGAESEEEDASHAA
```

sp|P11289|YFIL_ECOLI Uncharacterized protein YfiL OS=Escherichia coli (strain K12) OX=83333 GN=yfiL PE=4 SV=3

```
                                     *******************
0     MMKKFIAPLLALLVSGCQIDPYTHAPTLTSTDWYDVGMEDAISGSAIKDD
      ************
50    DAFSDSQADRGLYLKGYAEGQKKTCQTDFTYARGLSGKSFPASCNNVENA

100   SQLHEVWQKGADENASTIRLN
```

sp|P37744|RMLA1_ECOLI Glucose-1-phosphate thymidylyltransferase 1 OS=Escherichia coli (strain K12) OX=83333 GN=rfbA PE=1 SV=2

```
0     MKMRKGIILAGGSGTRLYPVTMAVSKQLLPIYDKPMIYYPLSTLMLAGIR

50    DILIISTPQDTPRFQQLLGDGSQWGLNLQYKVQPSPDGLAQAFIIGEEFI
                                                       *
100   GGDDCALVLGDNIFYGHDLPKLMEAAVNKESGATVFAYHVNDPERYGVVE
      *******************
150   FDKNGTAISLEEKPLEPKSNYAVTGLYFYDNDVVQMAKNLKPSARGELEI

200   TDINRIYLEQGRLSVAMMGRGYAWLDTGTHQSLIEASNFIATIEERQGLK

250   VSCPEEIAFRKGFIDVEQVRKLAVPLIKNNYGQYLYKMTKDSN
```

sp|P0AG07|RPE_ECOLI Ribulose-phosphate 3-epimerase OS=Escherichia coli (strain K12) OX=83333 GN=rpe PE=1 SV=1

```
0     MKQYLIAPSILSADFARLGEDTAKALAAGADVVHFDVMDNHYVPNLTIGP

50    MVLKSLRNYGITAPIDVHLMVKPVDRIVPDFAAAGASIITFHPEASEHVD

100   RTLQLIKENGCKAGLVFNPATPLSYLDYVMDKLDVILLMSVNPGFGGQSF

150   IPQTLDKLREVRRRIDESGFDIRLEVDGGVKVNNIGEIAAAGADMFVAGS
         **********************
200   AIFDQPDYKKVIDEMRSELAKVSHE
```

sp|P76616|YGAQ_ECOLI Putative uncharacterized protein YgaQ OS=Escherichia coli (strain K12) OX=83333 GN=ygaQ PE=5 SV=2

```
0     MFSIKPGPRNLPIDNPTLLSWNITDGDLNSKLNTLEYLNCITNIINSCGV

50    YPQGLKDREIISTFHAEKVINDLLKNDYKISLSPDTTYRELNKAAQRSIT

100   APDRIGERKTWVYQRDTMIERGDNSGVYQYGRAEHFTHIISDKPSPKDKY
```

```
150   VAYAINIPDYELAADVYNINVTSPSGQQETFKILINLEHLRQTLERKSLT

200   AVQKSQCEIITPKKPGEAILHAFNATYQQIRENMSEFARCHYGYIQIPPV

250   TTFRADGPETPEEEKGYWFHAYQPEDLCTIHNPMGDLQDFIALVKDAKKF
                       **********************
300   GIDIIPDYTFNFMGIGGSGKNDLDYPSADIRAKISKDIEGGIPGYWQGQV
                       *********************************
350   LIPFIKDPVTKERKQIHPEDIHLTAKDFEASKDNISKDEWENLHALKEKR
      *
400   LNGMPKTTPKSDQVIMLQNQYVREMRKYGVRGLRYDAAKHSKHEQIERSI

450   TPPLKNYNERLHNTNLFNPKYHKKAVMNYMEYLVTCQLDEQQMSSLLYER

500   DDLSAIDFSLLMKTIKAFSFGGDLQTLASKPGSTISSIPSERRILININH

550   DFPNNGNLFNDFLFNHQQDEQLAMAYIAALPFSRPLVYWDGQVLKSTTEI

600   KNYDGSTRVGGEAWLNKGCSTYQQLYNEFHALYIDKAGIWSAFEGVSATK

650   NVLAFSRGDSVNINHSPHDGLVIINKGNEEVEGTWPNKLQPGIYKNMGSN

700   SVNIIINNTRKIIPPGKVFTLRGGTLNINIPGRSALLLGKTGEPPNYLYL

750

sp|P0A8X4|YCCT_ECOLI UPF0319 protein YccT OS=Escherichia coli (strain
K12) OX=83333 GN=yccT PE=3 SV=1
                                       *****************
0     MKTGIVTTLIALCLPVSVFATTLRLSTDVDLLVLDGKKVSSSLLRGADSI
      ****
50    ELDNGPHQLVFRVEKTIHLSNSEERLYISPPLVVSFNTQLINQVNFRLPR

100   LENEREANHFDAAPRLELLDGDATPIPVKLDILAITSTAKTIDYEVEVER

150   YNKSAKRASLPQFATMMADDSTLLSGVSELDAIPPQSQVLTEQRLKYWFK

200   LADPQTRNTFLQWAEKQPSS

sp|P0A6A8|ACP_ECOLI Acyl carrier protein OS=Escherichia coli (strain K12)
OX=83333 GN=acpP PE=1 SV=2
                                                ********
0     MSTIEERVKKIIGEQLGVKQEEVTNNASFVEDLGADSLDTVELVMALEEE
      *****************
50    FDTEIPDEEAEKITTVQAAIDYINGHQA

sp|P03825|GSPB_ECOLI Putative general secretion pathway protein B
OS=Escherichia coli (strain K12) OX=83333 GN=gspB PE=2 SV=2

0     MFEFYIAAREQKETGHPGIFSRQKHSTIIYVICLLLICLWFAGMVLVGGY

50    ARQLWVLWIVKAEVTVEAETPAFKQSTQHYFFKKQPLPVVESVEEEDDPG
         *****************************
```

```
100   VAVENAPSSSEDEENTVEESEEKAGLRERVKNALNELER

sp|P78271|YFES_ECOLI Uncharacterized protein YfeS OS=Escherichia coli
(strain K12) OX=83333 GN=yfeS PE=4 SV=1
                                         ***************
0     MKKRFIYHDEKSNKFWWIDYEGDSLAVNYGKVGSIGKFQTKEFDNEEQCL
      ********************
50    KEASKLIAAKMKKGYQEDPKFNFMDRYYFDDEEIGLHVKTSHPNFQCHFT

100   DPLYMCCWDEESPFGSDEGADALNVLENSLRKEPDLDCADFPQMLIETMW

150   GMKYIAMDSILEEDVRAQLLVDEMSTIQSNMITYATAFGQIKVMGKISHK

200   LKKMGLNALARHQLTAKILQWGDGQDSPILQKMIDDLTAFPHEN

sp|P0AE24|ARAE_ECOLI Arabinose-proton symporter OS=Escherichia coli
(strain K12) OX=83333 GN=araE PE=1 SV=1

0     MVTINTESALTPRSLRDTRRMNMFVSVAAAVAGLLFGLDIGVIAGALPFI

50    TDHFVLTSRLQEWVVSSMMLGAAIGALFNGWLSFRLGRKYSLMAGAILFV

100   LGSIGSAFATSVEMLIAARVVLGIAVGIASYTAPLYLSEMASENVRGKMI

150   SMYQLMVTLGIVLAFLSDTAFSYSGNWRAMLGVLALPAVLLIILVVFLPN
                          ************************
200   SPRWLAEKGRHIEAEEVLRMLRDTSEKAREELNEIRESLKLKQGGWALFK

250   INRNVRRAVFLGMLLQAMQQFTGMNIIMYYAPRIFKMAGFTTTEQQMIAT

300   LVVGLTFMFATFIAVFTVDKAGRKPALKIGFSVMALGTLVLGYCLMQFDN

350   GTASSGLSWLSVGMTMMCIAGYAMSAAPVVWILCSEIQPLKCRDFGITCS

400   TTTNWVSNMIIGATFLTLLDSIGAAGTFWLYTALNIAFVGITFWLIPETK

450   NVTLEHIERKLMAGEKLRNIGV

sp|P15082|SRLR_ECOLI Glucitol operon repressor OS=Escherichia coli
(strain K12) OX=83333 GN=srlR PE=1 SV=1

0     MKPRQRQAAILEYLQKQGKCSVEELAQYFDTTGTTIRKDLVILEHAGTVI
               ********************
50    RTYGGVVLNKEESDPPIDHKTLINTHKKELIAEAAVSFIHDGDSIILDAG

100   STVLQMVPLLSRFNNITVMTNSLHIVNALSELDNEQTILMPGGTFRKKSA

150   SFHGQLAENAFEHFTFDKLFMGTDGIDLNAGVTTFNEVYTVSKAMCNAAR
            *********************
200   EVILMADSSKFGRKSPNVVCSLESVDKLITDAGIDPAFRQALEEKGIDVI

250   ITGESNE
```

sp|P23865|PRC_ECOLI Tail-specific protease OS=Escherichia coli (strain K12) OX=83333 GN=prc PE=1 SV=2

```
0     MNMFFRLTALAGLLAIAGQTFAVEDITRADQIPVLKEETQHATVSERVTS
                                                ***************
50    RFTRSHYRQFDLDQAFSAKIFDRYLNLLDYSHNVLLASDVEQFAKKKTEL
      **************
100   GDELRSGKLDVFYDLYNLAQKRRFERYQYALSVLEKPMDFTGNDTYNLDR
            **********************************
150   SKAPWPKNEAELNALWDSKVKFDELSLKLTGKTDKEIRETLTRRYKFAIR

200   RLAQTNSEDVFSLAMTAFAREIDPHTNYLSPRNTEQFNTEMSLSLEGIGA

250   VLQMDDDYTVINSMVAGGPAAKSKAISVGDKIVGVGQTGKPMVDVIGWRL

300   DDVVALIKGPKGSKVRLEILPAGKGTKTRTVTLTRERIRLEDRAVKMSVK

350   TVGKEKVGVLDIPGFYVGLTDDVKVQLQKLEKQNVSSVIIDLRSNGGGAL

400   TEAVSLSGLFIPAGPIVQVRDNNGKVREDSDTDGQVFYKGPLVVLVDRFS

450   ASASEIFAAAMQDYGRALVVGEPTFGKGTVQQYRSLNRIYDQMLRPEWPA
                                        ****************
500   LGSVQYTIQKFYRVNGGSTQRKGVTPDIIMPTGNEETETGEKFEDNALPW
      ****
550   DSIDAATYVKSGDLTAFEPELLKEHNARIAKDPEFQNIMKDIARFNAMKD
                                 ***********************
600   KRNIVSLNYAVREKENNEDDATRLARLNERFKREGKPELKKLDDLPKDYQ
      *********
650   EPDPYLDETVNIALDLAKLEKARPAEQPAPVK
```

sp|P76299|FLHB_ECOLI Flagellar biosynthetic protein FlhB OS=Escherichia coli (strain K12) OX=83333 GN=flhB PE=1 SV=1

```
      ***********************
0     MSDESDDKTEAPTPHRLEKAREEGQIPRSRELTSLLILLVGVSVIWFGGV

50    SLARRLSGMLSAGLHFDHSIINDPNLILGQIILLIREAMLALLPLISGVV

100   LVALISPVMLGGLVFSGKSLQPKFSKLNPLPGIKRMFSAQTGAELLKAIL

150   KTILVGSVTGFFLWHHWPQMMRLMAESPITAMGNAMDLVGLCALLVVLGV
                      ********************
200   IPMVGFDVFFQIFSHLKKLRMSRQDIRDEFKQSEGDPHVKGRIRQMQRAA

250   ARRRMMADVPKADVIVNNPTHYSVALQYDENKMSAPKVVAKGAGLVALRI

300   REIGAENNVPTLEAPPLARALYRHAEIGQQIPGQLYAAVAEVLAWVWQLK

350   RWRLAGGQRPVQPTHLPVPEALDFINEKPTHE
```

sp|P0ADC3|LOLC_ECOLI Lipoprotein-releasing system transmembrane protein LolC OS=Escherichia coli (strain K12) OX=83333 GN=lolC PE=1 SV=1

```
0     MYQPVALFIGLRYMRGRAADRFGRFVSWLSTIGITLGVMALVTVLSVMNG
```

```
50    FERELQNNILGLMPQAILSSEHGSLNPQQLPETAVKLDGVNRVAPITTGD

100   VVLQSARSVAVGVMLGIDPAQKDPLTPYLVNVKQTDLEPGKYNVILGEQL

150   ASQLGVNRGDQIRVMVPSASQFTPMGRIPSQRLFNVIGTFAANSEVDGYE
                                 *********************
200   MLVNIEDASRLMRYPAGNITGWRLWLDEPLKVDSLSQQKLPEGSKWQDWR

250   DRKGELFQAVRMEKNMMGLLLSLIVAVAAFNIITSLGLMVMEKQGEVAIL

300   QTQGLTPRQIMMVFMVQGASAGIIGAILGAALGALLASQLNNLMPIIGVL

350   LDGAALPVAIEPLQVIVIALVAMAIALLSTLYPSWRAAATQPAEALRYE
```

sp|P00957|SYA_ECOLI Alanine--tRNA ligase OS=Escherichia coli (strain K12) OX=83333 GN=alaS PE=1 SV=2

```
0     MSKSTAEIRQAFLDFFHSKGHQVVASSSLVPHNDPTLLFTNAGMNQFKDV

50    FLGLDKRNYSRATTSQRCVRAGGKHNDLENVGYTARHHTFFEMLGNFSFG
                                        ******************
100   DYFKHDAIQFAWELLTSEKWFALPKERLWVTVYESDDEAYEIWEKEVGIP
      ***
150   RERIIRIGDNKGAPYASDNFWQMGDTGPCGPCTEIFYDHGDHIWGGPPGS

200   PEEDGDRYIEIWNIVFMQFNRQADGTMEPLPKPSVDTGMGLERIAAVLQH

250   VNSNYDIDLFRTLIQAVAKVTGATDLSNKSLRVIADHIRSCAFLIADGVM

300   PSNENRGYVLRRIIRRAVRHGNMLGAKETFFYKLVGPLIDVMGSAGEDLK

350   RQQAQVEQVLKTEEEQFARTLERGLALLDEELAKLSGDTLDGETAFRLYD

400   TYGFPVDLTADVCRERNIKVDEAGFEAAMEEQRRRAREASGFGADYNAMI

450   RVDSASEFKGYDHLELNGKVTALFVDGKAVDAINAGQEAVVVLDQTPFYA

500   ESGGQVGDKGELKGANFSFAVEDTQKYGQAIGHIGKLAAGSLKVGDAVQA

550   DVDEARRARIRLNHSATHLMHAALRQVLGTHVSQKGSLVNDKVLRFDFSH

600   NEAMKPEEIRAVEDLVNTQIRRNLPIETNIMDLEAAKAKGAMALFGEKYD

650   ERVRVLSMGDFSTELCGGTHASRTGDIGLFRIISESGTAAGVRRIEAVTG

700   EGAIATVHADSDRLSEVAHLLKGDSNNLADKVRSVLERTRQLEKELQQLK

750   EQAAAQESANLSSKAIDVNGVKLLVSELSGVEPKMLRTMVDDLKNQLGST

800   IIVLATVVEGKVSLIAGVSKDVTDRVKAGELIGMVAQQVGGKGGGRPDMA

850   QAGGTDAAALPAALASVKGWVSAKLQ
```

sp|P13029|KATG_ECOLI Catalase-peroxidase OS=Escherichia coli (strain K12) OX=83333 GN=katG PE=1 SV=2

```
0     MSTSDDIHNTTATGKCPFHQGGHDQSAGAGTTTRDWWPNQLRVDLLNQHS
            ************************
50    NRSNPLGEDFDYRKEFSKLDYYGLKKDLKALLTESQPWWPADWGSYAGLF

100   IRMAWHGAGTYRSIDGRGGAGRGQQRFAPLNSWPDNVSLDKARRLLWPIK

150   QKYGQKISWADLFILAGNVALENSGFRTFGFGAGREDVWEPDLDVNWGDE

200   KAWLTHRHPEALAKAPLGATEMGLIYVNPEGPDHSGEPLSAAAAIRATFG

250   NMGMNDEETVALIAGGHTLGKTHGAGPTSNVGPDPEAAPIEEQGLGWAST

300   YGSGVGADAITSGLEVVWTQTPTQWSNYFFENLFKYEWVQTRSPAGAIQF

350   EAVDAPEIIPDPFDPSKKRKPTMLVTDLTLRFDPEFEKISRRFLNDPQAF

400   NEAFARAWFKLTHRDMGPKSRYIGPEVPKEDLIWQDPLPQPIYNPTEQDI

450   IDLKFAIADSGLSVSELVSVAWASASTFRGGDKRGGANGARLALMPQRDW

500   DVNAAAVRALPVLEKIQKESGKASLADIIVLAGVVGVEKAASAAGLSIHV

550   PFAPGRVDARQDQTDIEMFELLEPIADGFRNYRARLDVSTTESLLIDKAQ
                                                     ***
600   QLTLTAPEMTALVGGMRVLGANFDGSKNGVFTDRVGVLSNDFFVNLLDMR
      **************************
650   YEWKATDESKELFEGRDRETGEVKFTASRADLVFGSNSVLRAVAEVYASS

700   DAHEKFVKDFVAAWVKVMNLDRFDLL
```

sp|P33634|YFIE_ECOLI Uncharacterized HTH-type transcriptional regulator YfiE OS=Escherichia coli (strain K12) OX=83333 GN=yfiE PE=3 SV=2

```
0     MDLRRFITLKTVVEEGSFLRASQKLCCTQSTVTFHIQQLEQEFSVQLFEK
                                  **********************
50    IGRRMCLTREGKKLLPHIYELTRVMDTLREAAKKESDPDGELRVVSGETL
      **
100   LSYRMPQVLQRFRQRAPKVRLSLQSLNCYVIRDALLNDEADVGVFYRVGN

150   DDALNRRELGEQSLVLVASPQIADVDFTEPGRHNACSFIINEPQCVFRQI

200   FESTLRQRRITVENTIELISIESIKRCVAANIGVSYLPRFAVAKELECGE

250   LIELPFGEQSQTITAMCAHHAGKAVSPAMHTFIQCVEESFVAG
```

sp|P0AFR4|YCIO_ECOLI Uncharacterized protein YciO OS=Escherichia coli (strain K12) OX=83333 GN=yciO PE=1 SV=1

```
0     MSQFFYIHPDNPQQRLINQAVEIVRKGGVIVYPTDSGYALGCKIEDKNAM

50    ERICRIRQLPDGHNFTLMCRDLSELSTYSFVDNVAFRLMKNNTPGNYTFI
```

****
100   LKGTKEVPRRLLQEKRKTIGMRVPSNPIAQALLEALGEPMLSTSLMLPGS
      ****
150   EFTESDPEEIKDRLEKQVDLIIHGGYLGQKPTTVIDLTDDTPVVVREGVG

200   DVKPFL

sp|P68644|FIXC_ECOLI Protein FixC OS=Escherichia coli (strain K12) OX=83333 GN=fixC PE=3 SV=1

0   MSEDIFDAIIVGAGLAGSVAALVLAREGAQVLVIERGNSAGAKNVTGGRL

50   YAHSLEHIIPGFADSAPVERLITHEKLAFMTEKSAMTMDYCNGDETSPSQ

100   RSYSVLRSKFDAWLMEQAEEAGAQLITGIRVDNLVQRDGKVVGVEADGDV

150   IEAKTVILADGVNSILAEKLGMAKRVKPTDVAVGVKELIELPKSVIEDRF

200   QLQGNQGAACLFAGSPTDGLMGGGFLYTNENTLSLGLVCGLHHLHDAKKS

250   VPQMLEDFKQHPAVAPLIAGGKLVEYSAHVVPEAGINMLPELVGDGVLIA
      ****
300   GDAAGMCMNLGFTIRGMDLAIAAGEAAAKTVLSAMKSDDFSKQKLAEYRQ
      *****
350   HLESGPLRDMRMYQKLPAFLDNPRMFSGYPELAVGVARDLFTIDGSAPEL

400   MRKKILRHGKKVGFINLIKDGMKGVTVL

sp|P0A7S9|RS13_ECOLI 30S ribosomal protein S13 OS=Escherichia coli (strain K12) OX=83333 GN=rpsM PE=1 SV=2
      ****
0   MARIAGINIPDHKHAVIALTSIYGVGKTRSKAILAAAGIAEDVKISELSE
      ****
50   GQIDTLRDEVAKFVVEGDLRREISMSIKRLMDLGCYRGLRHRRGLPVRGQ

100   RTKTNARTRKGPRKPIKK

sp|P0AGC5|MLTF_ECOLI Membrane-bound lytic murein transglycosylase F OS=Escherichia coli (strain K12) OX=83333 GN=mltF PE=1 SV=2

0   MKKLKINYLFIGILALLLAVALWPSIPWFGKADNRIAAIQARGELRVSTI

50   HTPLTYNEINGKPFGLDYELAKQFADYLGVKLKVTVRQNISQLFDDLDNG

100   NADLLAAGLVYNSERVKNYQPGPTYYSVSQQLVYKVGQYRPRTLGNLTAE
      ****
150   QLTVAPGHVVVNDLQTLKETKFPELSWKVDDKKGSAELMEDVIEGKLDYT

200   IADSVAISLFQRVHPELAVALDITDEQPVTWFSPLDGDNTLSAALLDFFN

250   EMNEDGTLARIEEKYLGHGDDFDYVDTRTFLRAVDAVLPQLKPLFEKYAE

300   EIDWRLLAAIAYQESHWDAQATSPTGVRGMMMLTKNTAQSLGITDRTDAE

```
350   QSISGGVRYLQDMMSKVPESVPENERIWFALAAYNMGYAHMLDARALTAK

400   TKGNPDSWADVKQRLPLLSQKPYYSKLTYGYARGHEAYAYVENIRKYQIS

450   LVGYLQEKEKQATEAAMQLAQDYPAVSPTELGKEKFPFLSFLSQSSSNYL

500   THSPSLLFSRKGSEEKQN
```

sp|P0AFE0|NUOJ_ECOLI NADH-quinone oxidoreductase subunit J OS=Escherichia coli (strain K12) OX=83333 GN=nuoJ PE=1 SV=1

```
0     MEFAFYICGLIAILATLRVITHTNPVHALLYLIISLLAISGVFFSLGAYF

50    AGALEIIVYAGAIMVLFVFVVMMLNLGGSEIEQERQWLKPQVWIGPAILS

100   AIMLVVIVYAILGVNDQGIDGTPISAKAVGITLFGPYVLAVELASMLLLA
               *************************
150   GLVVAFHVGREERAGEVLSNRKDDSAKRKTEEHA
```

sp|P39389|YJIR_ECOLI Uncharacterized HTH-type transcriptional regulator YjiR OS=Escherichia coli (strain K12) OX=83333 GN=yjiR PE=3 SV=1

```
0     MTRYQHLATLLAERIEQGLYRHGEKLPSVRSLSQEHGVSISTVQQAYQTL
                                                 *****
50    ETMKLITPQPRSGYFVAQRKAQPPVPPMTRPVQRPVEITQWDQVLDMLEA
      **********************
100   HSDSSIVPLSKSTPDVEAPSLKPLWRELSRVVQHNLQTVLGYDLLAGQRV

150   LREQIARLMLDSGSVVTADDIIITSGCHNSMSLALMAVCKPGDIVAVESP

200   CYYGSMQMLRGMGVKVIEIPTDPETGISVEALELALEQWPIKGIILVPNC

250   NNPLGFIMPDARKRAVLSLAQRHDIVIFEDDVYGELATEYPRPRTIHSWD

300   IDGRVLLCSSFSKSIAPGLRVGWVAPGRYHDKLMHMKYAISSFNVPSTQM

350   AAATFVLEGHYHRHIRRMRQIYQRNLALYTCWIREYFPCEICITRPKGGF

400   LLWIELPEQVDMVCVARQLCRMKIQVAAGSIFSASGKYRNCLRINCALPL

450   SETYREALKQIGEAVYRAME
```

sp|P0A9L3|FKBB_ECOLI FKBP-type 22 kDa peptidyl-prolyl cis-trans isomerase OS=Escherichia coli (strain K12) OX=83333 GN=fklB PE=1 SV=2

```
0     MTTPTFDTIEAQASYGIGLQVGQQLSESGLEGLLPEALVAGIADALEGKH
                                      ******************
50    PAVPVDVVHRALREIHERADAVRRQRFQAMAAEGVKYLEENAKKEGVNST
      **
100   ESGLQFRVINQGEGAIPARTDRVRVHYTGKLIDGTVFDSSVARGEPAEFP

150   VNGVIPGWIEALTLMPVGSKWELTIPQELAYGERGAGASIPPFSTLVFEV

200   ELLEIL
```

sp|P13669|MNGR_ECOLI Mannosyl-D-glycerate transport/metabolism system repressor MngR OS=Escherichia coli (strain K12) OX=83333 GN=mngR PE=1 SV=1

```
0     MGHKPLYRQIADRIREQIARGELKPGDALPTESALQTEFGVSRVTVRQAL

50    RQLVEQQILESIQGSGTYVKEERVNYDIFQLTSFDEKLSDRHVDTHSEVL

100   IFEVIPADDFLQQQLQITPQDRVWHVKRVRYRKQKPMALEETWMPLALFP
             **********************
150   DLTWQVMENSKYHFIEEVKKMVIDRSEQEIIPLMPTEEMSRLLNISQTKP

200   ILEKVSRGYLVDGRVFEYSRNAFNTDDYKFTLIAQRKSSR
```

sp|P0A9M0|LON_ECOLI Lon protease OS=Escherichia coli (strain K12) OX=83333 GN=lon PE=1 SV=1

```
0     MNPERSERIEIPVLPLRDVVVYPHMVIPLFVGREKSIRCLEAAMDHDKKI

50    MLVAQKEASTDEPGVNDLFTVGTVASILQMLKLPDGTVKVLVEGLQRARI

100   SALSDNGEHFSAKAEYLESPTIDEREQEVLVRTAISQFEGYIKLNKKIPP

150   EVLTSLNSIDDPARLADTIAAHMPLKLADKQSVLEMSDVNERLEYLMAMM
                                             *************
200   ESEIDLLQVEKRIRNRVKKQMEKSQREYYLNEQMKAIQKELGEMDDAPDE
      *************************************
250   NEALKRKIDAAKMPKEAKEKAEAELQKLKMMSPMSAEATVVRGYIDWMVQ

300   VPWNARSKVKKDLRQAQEILDTDHYGLERVKDRILEYLAVQSRVNKIKGP

350   ILCLVGPPGVGKTSLGQSIAKATGRKYVRMALGGVRDEAEIRGHRRTYIG
                            ********************
400   SMPGKLIQKMAKVGVKNPLFLLDEIDKMSSDMRGDPASALLEVLDPEQNV

450   AFSDHYLEVDYDLSDVMFVATSNSMNIPAPLLDRMEVIRLSGYTEDEKLN

500   IAKRHLLPKQIERNALKKGELTVDDSAIIGIIRYYTREAGVRGLEREISK

550   LCRKAVKQLLLDKSLKHIEINGDNLHDYLGVQRFDYGRADNENRVGQVTG

600   LAWTEVGGDLLTIETACVPGKGKLTYTGSLGEVMQESIQAALTVVRARAE

650   KLGINPDFYEKRDIHVHVPEGATPKDGPSAGIAMCTALVSCLTGNPVRAD

700   VAMTGEITLRGQVLPIGGLKEKLLAAHRGGIKTVLIPFENKRDLEEIPDN

750   VIADLDIHPVKRIEEVLTLALQNEPSGMQVVTAK
```

sp|P07102|PPA_ECOLI Periplasmic AppA protein OS=Escherichia coli (strain K12) OX=83333 GN=appA PE=1 SV=2

```
0     MKAILIPFLSLLIPLTPQSAFAQSEPELKLESVVIVSRHGVRAPTKATQL
```

```
50    MQDVTPDAWPTWPVKLGWLTPRGGELIAYLGHYQRQRLVADGLLAKKGCP

100   QSGQVAIIADVDERTRKTGEAFAAGLAPDCAITVHTQADTSSPDPLFNPL

150   KTGVCQLDNANVTDAILSRAGGSIADFTGHRQTAFRELERVLNFPQSNLC
       **********************
200   LKREKQDESCSLTQALPSELKVSADNVSLTGAVSLASMLTEIFLLQQAQG

250   MPEPGWGRITDSHQWNTLLSLHNAQFYLLQRTPEVARSRATPLLDLIKTA

300   LTPHPPQKQAYGVTLPTSVLFIAGHDTNLANLGGALELNWTLPGQPDNTP

350   PGGELVFERWRRLSDNSQWIQVSLVFQTLQQMRDKTPLSLNTPPGEVKLT

400   LAGCEERNAQGMCSLAGFTQIVNEARIPACSL

sp|P0AB74|KBAY_ECOLI D-tagatose-1,6-bisphosphate aldolase subunit KbaY
OS=Escherichia coli (strain K12) OX=83333 GN=kbaY PE=1 SV=1

0     MSIISTKYLLQDAQANGYAVPAFNIHNAETIQAILEVCSEMRSPVILAGT

50    PGTFKHIALEEIYALCSAYSTTYNMPLALHLDHHESLDDIRRKVHAGVRS
                                                 *********
100   AMIDGSHFPFAENVKLVKSVVDFCHSQDCSVEAELGRLGGVEDDMSVDAE
      ***********
150   SAFLTDPQEAKRFVELTGVDSLAVAIGTAHGLYSKTPKIDFQRLAEIREV

200   VDVPLVLHGASDVPDEFVRRTIELGVTKVNVATELKIAFAGAVKAWFAEN

250   PQGNDPRYYMRVGMDAMKEVVRNKINVCGSANRISA

sp|P76243|YEAO_ECOLI Uncharacterized protein YeaO OS=Escherichia coli
(strain K12) OX=83333 GN=yeaO PE=4 SV=2
                                   *********************
0     MNIQCKRVYDPAEQSDGYRILVDRLWPRGIKKTDLALDEWDKEITPSTEL

50    RKAFHGEVVDYATFREQYLAELAQHEQEGKRLADIAKKQPLTLLYSAKNT

100   TQNHALVLADWLRSL

sp|P62517|OPGH_ECOLI Glucans biosynthesis glucosyltransferase H
OS=Escherichia coli (strain K12) OX=83333 GN=mdoH PE=1 SV=1

0     MNKTTEYIDAMPIAASEKAALPKTDIRAVHQALDAEHRTWAREDDSPQGS
                        *********************
50    VKARLEQAWPDSLADGQLIKDDEGRDQLKAMPEAKRSSMFPDPWRTNPVG

100   RFWDRLRGRDVTPRYLARLTKEEQESEQKWRTVGTIRRYILLILTLAQTV

150   VATWYMKTILPYQGWALINPMDMVGQDLWVSFMQLLPYMLQTGILILFAV

200   LFCWVSAGFWTALMGFLQLLIGRDKYSISASTVGDEPLNPEHRTALIMPI
```

```
250   CNEDVNRVFAGLRATWESVKATGNAKHFDVYILSDSYNPDICVAEQKAWM

300   ELIAEVGGEGQIFYRRRRRRVKRKSGNIDDFCRRWGSQYSYMVVLDADSV

350   MTGDCLCGLVRLMEANPNAGIIQSSPKASGMDTLYARCQQFATRVYGPLF

400   TAGLHFWQLGESHYWGHNAIIRVKPFIEHCALAPLPGEGSFAGSILSHDF

450   VEAALMRRAGWGVWIAYDLPGSYEELPPNLLDELKRDRRWCHGNLMNFRL

500   FLVKGMHPVHRAVFLTGVMSYLSAPLWFMFLALSTALQVVHALTEPQYFL

550   QPRQLFPVWPQWRPELAIALFASTMVLLFLPKLLSILLIWCKGTKEYGGF

600   WRVTLSLLLEVLFSVLLAPVRMLFHTVFVVSAFLGWEVVWNSPQRDDDST

650   SWGEAFKRHGSQLLLGLVWAVGMAWLDLRFLFWLAPIVFSLILSPFVSVI

700   SSRATVGLRTKRWKLFLIPEEYSPPQVLVDTDRFLEMNRQRSLDDGFMHA

750   VFNPSFNALATAMATARHRASKVLEIARDRHVEQALNETPEKLNRDRRLV

800   LLSDPVTMARLHFRVWNSPERYSSWVSYYEGIKLNPLALRKPDAASQ

sp|P0A8L5|YCGN_ECOLI UPF0260 protein YcgN OS=Escherichia coli (strain
K12) OX=83333 GN=ycgN PE=3 SV=1
            ***************************
0     MAEHLMSDVPFWQSKTLDEMSDAEWESLCDGCGQCCLHKLMDEDTDEIYF

50    TNVACRQLNIKTCQCRNYERRFEFEPDCIKLTRENLPTFEWLPMTCAYRL

100   LAEGKDLPAWHPLLTGSKAAMHGERISVRHIAVKESEVIDWQDHILNKPD

150   WAQ

sp|P75925|C56I_ECOLI Cytochrome b561 homolog 2 OS=Escherichia coli
(strain K12) OX=83333 GN=yceJ PE=1 SV=1

0     MSFTNTPERYGVISAAFHWLSAIIVYGMFALGLWMVTLSYYDGWYHKAPE

50    LHKSIGILLMMGLVIRVLWRVISPPPGPLPSYSPMTRLAARAGHLALYLL

100   LFAIGISGYLISTADGKPISVFGWFDVPATLADAGAQADFAGALHFWLAW
                    ************************
150   SVVVLSVMHGFMALKHHFIDKDDTLKRMLGKSSSDYGV

sp|Q47537|TAUA_ECOLI Taurine-binding periplasmic protein OS=Escherichia
coli (strain K12) OX=83333 GN=tauA PE=1 SV=1

0     MAISSRNTLLAALAFIAFQAQAVNVTVAYQTSAEPAKVAQADNTFAKESG
                                                       *
50    ATVDWRKFDSGASIVRALASGDVQIGNLGSSPLAVAASQQVPIEVFLLAS
      *******************
100   KLGNSEALVVKKTISKPEDLIGKRIAVPFISTTHYSLLAALKHWGIKPGQ
```

```
150   VEIVNLQPPAIIAAWQRGDIDGAYVWAPAVNALEKDGKVLTDSEQVGQWG

200   APTLDVWVVRKDFAEKHPEVVKAFAKSAIDAQQPYIANPDVWLKQPENIS

250   KLARLSGVPEGDVPGLVKGNTYLTPQQQTAELTGPVNKAIIDTAQFLKEQ

300   GKVPAVANDYSQYVTSRFVQ
```

sp|P09831|GLTB_ECOLI Glutamate synthase [NADPH] large chain OS=Escherichia coli (strain K12) OX=83333 GN=gltB PE=1 SV=3

```
0     MLYDKSLERDNCGFGLIAHIEGEPSHKVVRTAIHALARMQHRGAILADGK

50    TGDGCGLLLQKPDRFFRIVAQERGWRLAKNYAVGMLFLNKDPELAAAARR

100   IVEEELQRETLSIVGWRDVPTNEGVLGEIALSSLPRIEQIFVNAPAGWRP

150   RDMERRLFIARRRIEKRLEADKDFYVCSLSNLVNIYKGLCMPTDLPRFYL

200   DLADLRLESAICLFHQRFSTNTVPRWPLAQPFRYLAHNGEINTITGNRQW

250   ARARTYKFQTPLIPDLHDAAPFVNETGSDSSSMDNMLELLLAGGMDIIRA

300   MRLLVPPAWQNNPDMDPELRAFFDFNSMHMEPWDGPAGIVMSDGRFAACN

350   LDRNGLRPARYVITKDKLITCASEVGIWDYQPDEVVEKGRVGPGELMVID
             ***********************         ***********
400   TRSGRILHSAETDDDLKSRHPYKEWMEKNVRRLVPFEDLPDEEVGSRELD
      **********
450   DDTLASYQKQFNYSAEELDSVIRVLGENGQEAVGSMGDDTPFAVLSSQPR

500   IIYDYFRQQFAQVTNPPIDPLREAHVMSLATSIGREMNVFCEAEGQAHRL

550   SFKSPILLYSDFKQLTTMKEEHYRADTLDITFDVTKTTLEATVKELCDKA

600   EKMVRSGTVLLVLSDRNIAKDRLPVPAPMAVGAIQTRLVDQSLRCDANII

650   VETASARDPHHFAVLLGFGATAIYPYLAYETLGRLVDTHAIAKDYRTVML

700   NYRNGINKGLYKIMSKMGISTIASYRCSKLFEAVGLHDDVVGLCFQGAVS

750   RIGGASFEDFQQDLLNLSKRAWLARKPISQGGLLKYVHGGEYHAYNPDVV

800   RTLQQAVQSGEYSDYQEYAKLVNERPATTLRDLLAITPGENAVNIADVEP

850   ASELFKRFDTAAMSIGALSPEAHEALAEAMNSIGGNSNSGEGGEDPARYG

900   TNKVSRIKQVASGRFGVTPAYLVNADVIQIKVAQGAKPGEGGQLPGDKVT

950   PYIAKLRYSVPGVTLISPPPHHDIYSIEDLAQLIFDLKQVNPKAMISVKL

1000  VSEPGVGTIATGVAKAYADLITIAGYDGGTGASPLSSVKYAGCPWELGLV
```

1050 ETQQALVANGLRHKIRLQVDGGLKTGVDIIKAAILGAESFGFGTGPMVAL

1100 GCKYLRICHLNNCATGVATQDDKLRKNHYHGLPFKVTNYFEFIARETREL

1150 MAQLGVTRLVDLIGRTDLLKELDGFTAKQQKLALSKLLETAEPHPGKALY

1200 CTENNPPFDNGLLNAQLLQQAKPFVDERQSKTFWFDIRNTDRSVGASLSG

1250 YIAQTHGDQGLAADPIKAYFNGTAGQSFGVWNAGGVELYLTGDANDYVGK

1300 GMAGGLIAIRPPVGSAFRSHEASIIGNTCLYGATGGRLYAAGRAGERFGV

1350 RNSGAITVVEGIGDNGCEYMTGGIVCILGKTGVNFGAGMTGGFAYVLDES

1400 GDFRKRVNPELVEVLSVDALAIHEEHLRGLITEHVQHTGSQRGEEILANW

1450 STFATKFALVKPKSSDVKALLGHRSRSAAELRVQAQ

sp|P75857|ELFC_ECOLI Probable outer membrane usher protein ElfC
OS=Escherichia coli (strain K12) OX=83333 GN=elfC PE=2 SV=1

0 MYRTHRQHSLLSSGGVPSFIGGLVVFVSAAFNAQAETWFDPAFFKDDPSM

50 VADLSRFEKGQKITPGVYRVDIVLNQTIVDTRNVNFVEITPEKGIAACLT

100 TESLDAMGVNTDAFPAFKQLDKQACVPLAEIIPDASVTFNVNKLRLEISV

150 PQIAIKSNARGYVPPERWDEGINALLLGYSFSGANSIHSSADSDSGDSYF

200 LNLNSGVNLGPWRLRNNSTWSRSSGQTAEWKNLSSYLQRAVIPLKGELTV

250 GDDYTAGDFFDSVSFRGVQLASDDNMLPDSLKGFAPVVRGIAKSNAQITI
****
300 KQNGYTIYQTYVSPGAFEISDLYSTSSSGDLLVEIKEADGSVNSYSVPFS

350 SVPLLQRQGRIKYAVTLAKYRTNSNEQQESKFAQATLQWGGPWGTTWYGG

400 GQYAEYYRAAMFGLGFNLGDFGAISFDATQAKSTLADQSEHKGQSYRFLY
****
450 AKTLNHLGTNFQLMGYRYSTSGFYTLSDTMYKHMDGYEFNDGDDEDTPMW

500 SRYYNLFYTKRGKLQVNISQQLGEYGSFYLSGSQQTYWHTDQQDRLLQFG

550 YNTQIKDLSLGISWNYSKSRGQPDADQVFALNFSLPLNLLLPRSNDSYTR

600 KKNYAWMTSNTSIDNEGHTTQNLGLTETLLDDGNLSYSVQQGYNSEGKTA

650 NGSASMDYKGAFADARVGYNYSDNGSQQQLNYALSGSLVAHSQGITLGQS

700 LGETNVLIAAPGAENTRVANSTGLKTDWRGYTVVPYATSYRENRIALDAA

750 SLKRNVDLENAVVNVVPTKGALVLAEFNAHAGARVLMKTSKQGIPLRFGA

800 IATLDGVQANSGIIDDDGSLYMAGLPAKGTISVRWGEAPDQICHINYELT

850   EQQINSAITRMDAICR

sp|P76156|YDFO_ECOLI Uncharacterized protein YdfO OS=Escherichia coli (strain K12) OX=83333 GN=ydfO PE=1 SV=2

0   MDQVVIFKQIFDKVRNDLNYQWFYSELKRHNVSHYIYYLATENVHIVLKN
              ****
50   DNTVLLKGLKNIVSVKFSKDRHLIETTSNKLKSREITFQEYRRNLAKAGV

100   FRWVTNIHEQKRYYYTFDNSLLFTESIQKTTQILPR

sp|P0AD44|YFHG_ECOLI Uncharacterized protein YfhG OS=Escherichia coli (strain K12) OX=83333 GN=yfhG PE=3 SV=1

0   MRHIFQRLLPRRLWLAGLPCLALLGCVQNHNKPAIDTPAEEKIPVYQLAD

50   YLSTECSDIWALQGKSTETNPLYWLRAMDCADRLMPAQSRQQARQYDDGS

100   WQNTFKQGILLADAKITPYERRQLVARIEALSTEIPAQVRPLYQLWRDGQ
      ****
150   ALQLQLAEERQRYSKLQQSSDSELDTLRQQHHVLQQQLELTTRKLENLTD

200   IERQLSTRKPAGNFSPDTPHESEKPAPSTHEVTPDEP

sp|P33218|YEBE_ECOLI Inner membrane protein YebE OS=Escherichia coli (strain K12) OX=83333 GN=yebE PE=1 SV=2
         ****
0   MANWLNQLQSLLGQSSSSTSSSADQGLVKLLVPGALGGLAGLLVANKSAR

50   KLLTKYGTNALLVGGGAVAGTVLWNKYKDKIRAAHQDEPQFGAQSTPLDE

100   RTARLILALVFAAKSDGHIDAKERAAIDQQLRGAGVEEQGRVLIEQAIEQ

150   PLDPQRLATGVRNEEEALEIYFLSCAAIDIDHFMERSYLNALGDALKIPQ

200   DVRDGIERDLEQQKRTLAE

sp|P08957|T1MK_ECOLI Type I restriction enzyme EcoKI M protein OS=Escherichia coli (strain K12) OX=83333 GN=hsdM PE=1 SV=1

0   MNNNDLVAKLWKLCDNLRDGGVSYQNYVNELASLLFLKMCKETGQEAEYL

50   PEGYRWDDLKSRIGQEQLQFYRKMLVHLGEDDKKLVQAVFHNVSTTITEP

100   KQITALVSNMDSLDWYNGAHGKSRDDFGDMYEGLLQKNANETKSGAGQYF

150   TPRPLIKTIIHLLKPQPREVVQDPAAGTAGFLIEADRYVKSQTNDLDDLD

200   GDTQDFQIHRAFIGLELVPGTRRLALMNCLLHDIEGNLDHGGAIRLGNTL

250   GSDGENLPKAHIVATNPPFGSAAGTNITRTFVHPTSNKQLCFMQHIIETL

300   HPGGRAAVVVPDNVLFEGGKGTDIRRDLMDKCHLHTILRLPTGIFYAQGV

350   KTNVLFFTKGTVANPNQDKNCTDDVWVYDLRTNMPSFGKRTPFTDEHLQP
                      ****
400   FERVYGEDPHGLSPRTEGEWSFNAEETEVADSEENKNTDQHLATSRWRKF
      ****
450   SREWIRTAKSDSLDISWLKDKDSIDADSLPEPDVLAAEAMGELVQALSEL

500   DALMRELGASDEADLQRQLLEEAFGGVKE

sp|P76402|YEGP_ECOLI UPF0339 protein YegP OS=Escherichia coli (strain K12) OX=83333 GN=yegP PE=1 SV=2
                                        ****
0   MAGWFELSKSSDNQFRFVLKAGNGETILTSELYTSKTSAEKGIASVRSNS
      ****
50   PQEERYEKKTASNGKFYFNLKAANHQIIGSSQMYATAQSRETGIASVKAN

100   GTSQTVKDNT

sp|P76520|YFDX_ECOLI Protein YfdX OS=Escherichia coli (strain K12) OX=83333 GN=yfdX PE=1 SV=1

0   MKRLIMATMVTAILASSTVWAADNAPVAAQQQTQQVQQTQKTAAAAERIS
                         ****
50   EQGLYAMRDVQVARLALFHGDPEKAKELTNEASALLSDDSTEWAKFAKPG
      **   *********************
100   KKTNLNDDQYIVINASVGISESYVATPEKEAAIKIANEKMAKGDKKGAME

150   ELRLAGVGVMENQYLMPLKQTRNALADAQKLLDKKQYYEANLALKGAEDG

200   IIVDSEALFVN

sp|P0AGC7|SMP_ECOLI Probable inner membrane protein Smp OS=Escherichia coli (strain K12) OX=83333 GN=ytjB PE=2 SV=1

0   MARTKLKFRLHRAVIVLFCLALLVALMQGASWFSQNHQRQRNPQLEELAR

50   TLARQVTLNVAPLMRTDSPDEKRIQAILDQLTDESRILDAGVYDEQGDLI

100   ARSGESVEVRDRLALDGKKAGGYFNQQIVEPIAGKNGPLGYLRLTLDTHT
                                                ****
150   LATEAQQVDNTTNILRLMLLLSLAIGVVLTRTLLQGKRTRWQQSPFLLTA
      ****
200   SKPVPEEEESEKKE

sp|P0CF69|INSE4_ECOLI Transposase InsE for insertion sequence IS3D OS=Escherichia coli (strain K12) OX=83333 GN=insE4 PE=3 SV=1
        ****
0   MTKTVSTSKKPRKQHSPEFRSEALKLAERIGVTAAARELSLYESQLYNWR

50   SKQQNQQTSSERELEMSTEIARLKRQLAERDEELAILQKAATYFAKRLK

sp|P45758|GSPD_ECOLI Putative secretin GspD OS=Escherichia coli (strain K12) OX=83333 GN=gspD PE=1 SV=2

0   MKGLNKITCCLLAALLMPCAGHAENEQYGANFNNADIRQFVEIVGQHLGK

50   TILIDPSVQGTISVRSNDTFSQQEYYQFFLSILDLYGYSVITLDNGFLKV

100   VRSANVKTSPGMIADSSRPGVGDELVTRIVPLENVPARDLAPLLRQMMDA

150   GSVGNVVHYEPSNVLILTGRASTINKLIEVIKRVDVIGTEKQQIIHLEYA
                                                  ******
200   SAEDLAEILNQLISESHGKSQMPALLSAKIVADKRTNSLIISGPEKARQR
      ***************   ****************
250   ITSLLKSLDVEESEEGNTRVYYLKYAKATNLVEVLTGVSEKLKDEKGNAR
      ****
300   KPSSSGAMDNVAITADEQTNSLVITADQSVQEKLATVIARLDIRRAQVLV

350   EAIIVEVQDGNGLNLGVQWANKNVGAQQFTNTGLPIFNAAQGVADYKKNG

400   GITSANPAWDMFSAYNGMAAGFFNGDWGVLLTALASNNKNDILATPSIVT

450   LDNKLASFNVGQDVPVLSGSQTTSGDNVFNTVERKTVGTKLKVTPQVNEG

500   DAVLLEIEQEVSSVDSSSNSTLGPTFNTRTIQNAVLVKTGETVVLGGLLD

550   DFSKEQVSKVPLLGDIPLVGQLFRYTSTERAKRNLMVFIRPTIIRDDDVY

600   RSLSKEKYTRYRQEQQQRIDGKSKALVGSEDLPVLDENTFNSHAPAPSSR

650

sp|P50456|MLC_ECOLI Protein mlc OS=Escherichia coli (strain K12) OX=83333 GN=mlc PE=1 SV=2

0   MVAENQPGHIDQIKQTNAGAVYRLIDQLGPVSRIDLSRLAQLAPASITKI

50   VREMLEAHLVQELEIKEAGNRGRPAVGLVVETEAWHYLSLRISRGEIFLA
      ****
100   LRDLSSKLVVEESQELALKDDLPLLDRIISHIDQFFIRHQKKLERLTSIA

150   ITLPGIIDTENGIVHRMPFYEDVKEMPLGEALEQHTGVPVYIQHDISAWT

200   MAEALFGASRGARDVIQVVIDHNVGAGVITDGHLLHAGSSSLVEIGHTQV

250   DPYGKRCYCGNHGCLETIASVDSILELAQLRLNQSMSSMLHGQPLTVDSL

300   CQAALRGDLLAKDIITGVGAHVGRILAIMVNLFNPQKILIGSPLSKAADI

350   LFPVISDSIRQQALPAYSQHISVESTQFSNQGTMAGAALVKDAMYNGSLL

400   IRLLQG

sp|P0AA41|TRUC_ECOLI tRNA pseudouridine synthase C OS=Escherichia coli (strain K12) OX=83333 GN=truC PE=1 SV=1

0   MLEILYQDEWLVAVNKPSGWLVHRSWLDRDEKVVVMQTVRDQIGQHVFTA

```
50    HRLDRPTSGVLLMGLSSEAGRLLAQQFEQHQIQKRYHAIVRGWLMEEAVL
      ********************
100   DYPLVEELDKIADKFAREDKGPQPAVTHYRGLATVEMPVATGRYPTTRYG

150   LVELEPKTGRKHQLRRHLAHLRHPIIGDSKHGDLRQNRSGAEHFGLQRLM

200   LHASQLSLTHPFTGEPLTIHAGLDDTWMQALSQFGWRGLLPENERVEFSA

250   PSGQDGEISS

sp|P0AGJ5|YFIF_ECOLI Uncharacterized tRNA/rRNA methyltransferase YfiF
OS=Escherichia coli (strain K12) OX=83333 GN=yfiF PE=1 SV=1
       ***************************
0     MNDEMKGKSGKVKVMYVRSDDDSDKRTHNPRTGKGGGRPGKSRADGGRRP

50    ARDDKQSQPRDRKWEDSPWRTVSRAPGDETPEKADHGGISGKSFIDPEVL

100   RRQRAEETRVYGENACQALFQSRPEAIVRAWFIQSVTPRFKEALRWMAAN

150   RKAYHVVDEAELTKASGTEHHGGVCFLIKKRNGTTVQQWVSQAGAQDCVL

200   ALENESNPHNLGGMMRSCAHFGVKGVVVQDAALLESGAAIRTAEGGAEHV

250   QPITGDNIVNVLDDFRQAGYTVVTTSSEQGKPLFKTSLPAKMVLVLGQEY

300   EGLPDAARDPNDLRVKIDGTGNVAGLNISVATGVLLGEWWRQNKA

sp|P0AFJ1|YJDM_ECOLI Protein YjdM OS=Escherichia coli (strain K12)
OX=83333 GN=yjdM PE=3 SV=1

0     MSLPHCPKCNSEYTYEDNGMYICPECAYEWNDAEPAQESDELIVKDANGN
       ******************************
50    LLADGDSVTIIKDLKVKGSSSMLKIGTKVKNIRLVEGDHNIDCKIDGFGP

100   MKLKSEFVKKN

sp|P13445|RPOS_ECOLI RNA polymerase sigma factor RpoS OS=Escherichia coli
(strain K12) OX=83333 GN=rpoS PE=1 SV=3
              ***************************************
0     MSQNTLKVHDLNEDAEFDENGVEVFDEKALVEQEPSDNDLAEEELLSQGA

50    TQRVLDATQLYLGEIGYSPLLTAEEEVYFARRALRGDVASRRRMIESNLR

100   LVVKIARRYGNRGLALLDLIEEGNLGLIRAVEKFDPERGFRFSTYATWWI
                                       *****************
150   RQTIERAIMNQTRTIRLPIHIVKELNVYLRTARELSHKLDHEPSAEEIAE
      ***********                ***********************
200   QLDKPVDDVSRMLRLNERITSVDTPLGGDSEKALLDILADEKENGPEDTT
      ***********
250   QDDDMKQSIVKWLFELNAKQREVLARRFGLLGYEAATLEDVGREIGLTRE

300   RVRQIQVEGLRRLREILQTQGLNIEALFRE
```

sp|P32151|YIIG_ECOLI Uncharacterized protein YiiG OS=Escherichia coli (strain K12) OX=83333 GN=yiiG PE=4 SV=1

0   MKRNLLSSAIIVAIMSLGLTGCDDKKAETETLPPANSQPAAPAPEAKPTE

50   APVAKAEAKPETPAQPVVDEQAVFDEKMDVYIKCYNKLQIPVQRSLARYA

100   DWLKDFKQGPTGEERTVYGIYGISESNLAECEKGVKSAVALTPALQPIDG

150   VAVSYIDAAVALGNTINEMDKYYTQENYKDDAFAKGKTLHQTFLKNLEAF

200   EPVAESYHAAIQEINDKRQLAELKNIEEREGKTFHYYSLAVMISAKQINN
      ****
250   LISQDKFDAEAAMKKVSELETLVAQAKEADKGGMNFSFINSAGQYQLEAK
            ****
300   KYVRRIRDKVPYSDWDKEQLQDANSSWMVEDSFPRALREYNEMVDDYNSL

350   R

sp|P05719|T1SK_ECOLI Type-1 restriction enzyme EcoKI specificity protein OS=Escherichia coli (strain K12) OX=83333 GN=hsdS PE=1 SV=1

0   MSAGKLPEGWVIAPVSTVTTLIRGVTYKKEQAINYLKDDYLPLIRANNIQ
                      ****
50   NGKFDTTDLVFVPKNLVKESQKISPEDIVIAMSSGSKSVVGKSAHQHLPF

100   ECSFGAFCGVLRPEKLIFSGFIAHFTKSSLYRNKISSLSAGANINNIKPA

150   SFDLINIPIPPLAEQKIIAEKLDTLLAQVDSTKARFEQIPQILKRFRQAV

200   LGGAVNGKLTEKWRNFEPQHSVFKKLNFESILTELRNGLSSKPNESGVGH

250   PILRISSVRAGHVDQNDIRFLECSESELNRHKLQDGDLLFTRYNGSLEFV

300   GVCGLLKKLQHQNLLYPDKLIRARLTKDALPEYIEIFFSSPSARNAMMNC

350   VKTTSGQKGISGKDIKSQVVLLPPVKEQAEIVRRVEQLFAYADTIEKQVN
                                                 ****
400   NALARVNNLTQSILAKAFRGELTAQWRAENPDLISGENSAAALLEKIKAE
      ****
450   RAASGGKKASRKKS

sp|P77732|RHMR_ECOLI Uncharacterized HTH-type transcriptional regulator RhmR OS=Escherichia coli (strain K12) OX=83333 GN=rhmR PE=1 SV=1

0   MLESSKVPALTRAIDILNLIARIGPCSAATIIDTLGIPKSTAYLLLNELR

50   RQRFLSLDHQENFCLWTRLVELSGHALSKMDLRELARPRLTQLMDTTGLL
                    ****
100   CHLGIIDNGSAYYILKVESSATISVRSHEGKSLSLYRSGIGKCLLAWQPA

150   AVQQSIIEGLVWEQATPTTITHPQQLHEELARIRRQGWSYDNGEDYADVR

200   CVAAPVFNANNELTAAISVVGTRLQINEEYRDYLAGKAIACARDISRLLG

```
250   WKSPFDLQAS

sp|P0A6I6|COAD_ECOLI Phosphopantetheine adenylyltransferase
OS=Escherichia coli (strain K12) OX=83333 GN=coaD PE=1 SV=1

0     MQKRAIYPGTFDPITNGHIDIVTRATQMFDHVILAIAASPSKKPMFTLEE

50    RVALAQQATAHLGNVEVVGFSDLMANFARNQHATVLIRGLRAVADFEYEM
                ************************
100   QLAHMNRHLMPELESVFLMPSKEWSFISSSLVKEVARHQGDVTHFLPENV

150   HQALMAKLA

sp|P04949|FLIC_ECOLI Flagellin OS=Escherichia coli (strain K12) OX=83333
GN=fliC PE=1 SV=2
                              *********************
0     MAQVINTNSLSLITQNNINKNQSALSSSIERLSSGLRINSAKDDAAGQAI
                                                      **
50    ANRFTSNIKGLTQAARNANDGISVAQTTEGALSEINNNLQRVRELTVQAT
      *****************************
100   TGTNSESDLSSIQDEIKSRLDEIDRVSGQTQFNGVNVLAKNGSMKIQVGA

150   NDNQTITIDLKQIDAKTLGLDGFSVKNNDTVTTSAPVTAFGATTTNNIKL

200   TGITLSTEAATDTGGTNPASIEGVYTDNGNDYYAKITGGDNDGKYYAVTV

250   ANDGTVTMATGATANATVTDANTTKATTITSGGTPVQIDNTAGSATANLG

300   AVSLVKLQDSKGNDTDTYALKDTNGNLYAADVNETTGAVSVKTITYTDSS
                  *********************
350   GAASSPTAVKLGGDDGKTEVVDIDGKTYDSADLNGGNLQTGLTAGGEALT

400   AVANGKTTDPLKALDDAIASVDKFRSSLGAVQNRLDSAVTNLNNTTTNLS

450   EAQSRIQDADYATEVSNMSKAQIIQQAGNSVLAKANQVPQQVLSLLQG

sp|Q46787|YGEG_ECOLI Uncharacterized protein YgeG OS=Escherichia coli
(strain K12) OX=83333 GN=ygeG PE=3 SV=1

0     MSTETIEIFNNSDEWANQLKHALSKGENLALLHGLTPDILDRIYAYAFDY

50    HEKGNITDAEIYYKFLCIYAFENHEYLKDFASVCQPKKKYQQAYDLYKLS
                                                 ***********
100   YNYFPYDDYSVIYRMGQCQIGAKNIDNAMQCFYHIINNCEDDSVKSKAQA
      ***********
150   YIELLNDNSEDNG

sp|P76594|LYSAC_ECOLI Peptidyl-lysine N-acetyltransferase PatZ
OS=Escherichia coli (strain K12) OX=83333 GN=patZ PE=1 SV=1

0     MSQRGLEALLRPKSIAVIGASMKPNRAGYLMMRNLLAGGFNGPVLPVTPA

50    WKAVLGVLAWPDIASLPFTPDLAVLCTNASRNLALLEELGEKGCKTCIIL
```

100 SAPASQHEDLRACALRHNMRLLGPNSLGLLAPWQGLNASFSPVPIKRGKL
****

150 AFISQSAAVSNTILDWAQQRKMGFSYFIALGDSLDIDVDELLDYLARDSK
**

200 TSAILLYLEQLSDARRFVSAARSASRNKPILVIKSGRSPAAQRLLNTTAG

250 MDPAWDAAIQRAGLLRVQDTHELFSAVETLSHMRPLRGDRLMIISNGAAP

300 AALALDALWSRNGKLATLSEETCQKLRDALPEHVAISNPLDLRDDASSEH

350 YIKTLDILLHSQDFDALMVIHSPSAAAPATESAQVLIEAVKHHPRSKYVS

400 LLTNWCGEHSSQEARRLFSEAGLPTYRTPEGTITAFMHMVEYRRNQKQLR

450 ETPALPSNLTSNTAEAHLLLQQAIAEGATSLDTHEVQPILQAYGMNTLPT

500 WIASDSTEAVHIAEQIGYPVALKLRSPDIPHKSEVQGVMLYLRTANEVQQ

550 AANAIFDRVKMAWPQARVHGLLVQSMANRAGAQELRVVVEHDPVFGPLIM

600 LGEGGVEWRPEDQAVVALPPLNMNLARYLVIQGIKSKKIRARSALRPLDV

650 AGLSQLLVQVSNLIVDCPEIQRLDIHPLLASGSEFTALDVTLDISPFEGD

700 NESRLAVRPYPHQLEEWVELKNGERCLFRPILPEDEPQLQQFISRVTKED

750 LYYRYFSEINEFTHEDLANMTQIDYDREMAFVAVRRIDQTEEILGVTRAI

800 SDPDNIDAEFAVLVRSDLKGLGLGRRLMEKLITYTRDHGLQRLNGITMPN

850 NRGMVALARKLGFNVDIQLEEGIVGLTLNLAQREES

sp|P07813|SYL_ECOLI Leucine--tRNA ligase OS=Escherichia coli (strain K12) OX=83333 GN=leuS PE=1 SV=2
****

0 MQEQYRPEEIESKVQLHWDEKRTFEVTEDESKEKYYCLSMLPYPSGRLHM

50 GHVRNYTIGDVIARYQRMLGKNVLQPIGWDAFGLPAEGAAVKNNTAPAPW

100 TYDNIAYMKNQLKMLGFGYDWSRELATCTPEYYRWEQKFFTELYKKGLVY

150 KKTSAVNWCPNDQTVLANEQVIDGCCWRCDTKVERKEIPQWFIKITAYAD

200 ELLNDLDKLDHWPDTVKTMQRNWIGRSEGVEITFNVNDYDNTLTVYTTRP

250 DTFMGCTYLAVAAGHPLAQKAAENNPELAAFIDECRNTKVAEAEMATMEK

300 KGVDTGFKAVHPLTGEEIPVWAANFVLMEYGTGAVMAVPGHDQRDYEFAS

350 KYGLNIKPVILAADGSEPDLSQQALTEKGVLFNSGEFNGLDHEAAFNAIA

400 DKLTAMGVGERKVNYRLRDWGVSRQRYWGAPIPMVTLEDGTVMPTPDDQL

450   PVILPEDVVMDGITSPIKADPEWAKTTVNGMPALRETDTFDTFMESSWYY

500   ARYTCPQYKEGMLDSEAANYWLPVDIYIGGIEHAIMHLLYFRFFHKLMRD

550   AGMVNSDEPAKQLLCQGMVLADAFYYVGENGERNWVSPVDAIVERDEKGR

600   IVKAKDAAGHELVYTGMSKMSKSKNNGIDPQVMVERYGADTVRLFMMFAS

650   PADMTLEWQESGVEGANRFLKRVWKLVYEHTAKGDVAALNVDALTENQKA

700   LRRDVHKTIAKVTDDIGRRQTFNTAIAAIMELMNKLAKAPTDGEQDRALM

750   QEALLAVVRMLNPFTPHICFTLWQELKGEGDIDNAPWPVADEKAMVEDST

800   LVVVQVNGKVRAKITVPVDATEEQVRERAGQEHLVAKYLDGVTVRKVIYV

850   PGKLLNLVVG

sp|P07118|SYV_ECOLI Valine--tRNA ligase OS=Escherichia coli (strain K12) OX=83333 GN=valS PE=1 SV=2

0   MEKTYNPQDIEQPLYEHWEKQGYFKPNGDESQESFCIMIPPPNVTGSLHM

50   GHAFQQTIMDTMIRYQRMQGKNTLWQVGTDHAGIATQMVVERKIAAEEGK

100   TRHDYGREAFIDKIWEWKAESGGTITRQMRRLGNSVDWERERFTMDEGLS

150   NAVKEVFVRLYKEDLIYRGKRLVNWDPKLRTAISDLEVENRESKGSMWHI

200   RYPLADGAKTADGKDYLVVATTRPETLLGDTGVAVNPEDPRYKDLIGKYV

250   ILPLVNRRIPIVGDEHADMEKGTGCVKITPAHDFNDYEVGKRHALPMINI
             ****
300   LTFDGDIRESAQVFDTKGNESDVYSSEIPAEFQKLERFAARKAVVAAVDA

350   LGLLEEIKPHDLTVPYGDRGGVVIEPMLTDQWYVRADVLAKPAVEAVENG

400   DIQFVPKQYENMYFSWMRDIQDWCISRQLWWGHRIPAWYDEAGNVYVGRN

450   EDEVRKENNLGADVVLRQDEDVLDTWFSSALWTFSTLGWPENTDALRQFH
                                                     ***
500   PTSVMVSGFDIIFFWIARMIMMTMHFIKDENGKPQVPFHTVYMTGLIRDD
      ****
550   EGQKMSKSKGNVIDPLDMVDGISLPELLEKRTGNMMQPQLADKIRKRTEK

600   QFPNGIEPHGTDALRFTLAALASTGRDINWDMKRLEGYRNFCNKLWNASR

650   FVLMNTEGQDCGFNGGEMTLSLADRWILAEFNQTIKAYREALDSFRFDIA

700   AGILYEFTWNQFCDWYLELTKPVMNGGTEAELRGTRHTLVTVLEGLLRLA

750   HPIIPFITETIWQRVKVLCGITADTIMLQPFPQYDASQVDEAALADTEWL

800   KQAIVAVRNIRAEMNIAPGKPLELLLRGCSADAERRVNENRGFLQTLARL

```
                                        ****************
850   ESITVLPADDKGPVSVTKIIDGAELLIPMAGLINKEDELARLAKEVAKIE
      ****
900   GEISRIENKLANEGFVARAPEAVIAKEREKLEGYAEAKAKLIEQQAVIAA

950   L
```

sp|P16433|HYCG_ECOLI Formate hydrogenlyase subunit 7 OS=Escherichia coli (strain K12) OX=83333 GN=hycG PE=1 SV=2

```
                       **********************
0     MSNLLGPRDANGIPVPMTVDESIASMKASLLKKIKRSAYVYRVDCGGCNG

50    CEIEIFATLSPLFDAERFGIKVVPSPRHADILLFTGAVTRAMRSPALRAW

100   QSAPDPKICISYGACGNSGGIFHDLYCVWGGTDKIVPVDVYIPGCPPTPA

150   ATLYGFAMALGLLEQKIHARGPGELDEQPAEILHGDMVQPLRVKVDREAR

200   RLAGYRYGRQIADDYLTQLGQGEEQVARWLEAENDPRLNEIVSHLNHVVE

250   EARIR
```

sp|P46142|YGGM_ECOLI Uncharacterized protein YggM OS=Escherichia coli (strain K12) OX=83333 GN=yggM PE=4 SV=2

```
0     MKKQWIVGTALLMLMTGNAWADGEPPTENILKDQFKKQYHGILKLDAITL
         ********************
50    KNLDAKGNQATWSAEGDVSSSDDLYTWVGQLADYELLEQTWTKDKPVKFS

100   AMLTSKGTPASGWSVNFYSFQAAASDRGRVVDDIKTNNKYLIVNSEDFNY

150   RFSQLESALNTQKNSIPALEKEVKALDKQMVAAQKAADAYWGKDANGKQM
                  *********************
200   TREDAFKKIHQQRDEFNKQNDSEAFAVKYDKEVYQPAIAACHKQSEECYE

250   VPIQQKRDFDINEQRRQTFLQSQKLSRKLQDDWVTLEKGQYPLTMKVSEI

300   NSKKVAILMKIDDINQANERWKKDTEQLRRNGVIK
```

sp|P76135|YDEO_ECOLI HTH-type transcriptional regulator YdeO OS=Escherichia coli (strain K12) OX=83333 GN=ydeO PE=2 SV=1

```
0     MSLVCSVIFIHHAFNANILDKDYAFSDGEILMVDNAVRTHFEPYERHFKE

50    IGFTENTIKKYLQCTNIQTVTVPVPAKFLRASNVPTGLLNEMIAYLNSEE

100   RNHHNFSELLLFSCLSIFAACKGFITLLTNGVLSVSGKVRNIVNMKPAHP
      ************************
150   WKLKDICDCLYISESLLKKKLKQEQTTFSQILLDARMQHAKNLIRVEGSV

200   NKIAEQCGYASTSYFIYAFRKHFGNSPKRVSKEYRCQSHTGMNTGNTMNA

250   LAI
```

sp|P68767|AMPA_ECOLI Cytosol aminopeptidase OS=Escherichia coli (strain K12) OX=83333 GN=pepA PE=1 SV=1

```
0     MEFSVKSGSPEKQRSACIVVGVFEPRRLSPIAEQLDKISDGYISALLRRG

50    ELEGKPGQTLLLHHVPNVLSERILLIGCGKERELDERQYKQVIQKTINTL

100   NDTGSMEAVCFLTELHVKGRNNYWKVRQAVETAKETLYSFDQLKTNKSEP

150   RRPLRKMVFNVPTRRELTSGERAIQHGLAIAAGIKAAKDLGNMPPNICNA

200   AYLASQARQLADSYSKNVITRVIGEQQMKELGMHSYLAVGQGSQNESLMS
                             *********************
250   VIEYKGNASEDARPIVLVGKGLTFDSGGISIKPSEGMDEMKYDMCGAAAV

300   YGVMRMVAELQLPINVIGVLAGCENMPGGRAYRPGDVLTTMSGQTVEVLN

350   TDAEGRLVLCDVLTYVERFEPEAVIDVATLTGACVIALGHHITGLMANHN

400   PLAHELIAASEQSGDRAWRLPLGDEYQEQLESNFADMANIGGRPGGAITA

450   GCFLSRFTRKYNWAHLDIAGTAWRSGKAKGATGRPVALLAQFLLNRAGFN

500   GEE
```

sp|P69783|PTGA_ECOLI PTS system glucose-specific EIIA component OS=Escherichia coli (strain K12) OX=83333 GN=crr PE=1 SV=2

```
      *************************
0     MGLFDKLKSLVSDDKKDTGTIEIIAPLSGEIVNIEDVPDVVFAEKIVGDG

50    IAIKPTGNKMVAPVDGTIGKIFETNHAFSIESDSGVELFVHFGIDTVELK

100   GEGFKRIAEEGQRVKVGDTVIEFDLPLLEEKAKSTLTPVVISNMDEIKEL

150   IKLSGSVTVGETPVIRIKK
```

sp|P77199|YAIT_ECOLI Putative uncharacterized protein YaiT OS=Escherichia coli (strain K12) OX=83333 GN=yaiT PE=5 SV=2

```
0     MHSWKKKLVVSQLALACTLAITSQANAANYDTWTYIDNPVTALDWDHMDK

50    AGTVDGNYVNYSGFVYYNNTNGDFDQSFNGDTVNGTISTYYLNHDYADST

100   ANQLDISNSVIHGSITSMLPGGYYDRFDADGNNLGGYDFYTDAVVDTHWR
                    ******************************
150   DGDVFTLNIANTTIDDDYEALYFTDSYKDGDVTKHTNETFDTSEGVAVNL

200   DVESNINISNNSRVAGIALSQGNTYNETYTTESHTWDNNISVKDSTVTSG

250   SNYILDSNTYGKTGHFGNSDEPSDYAGPGDVAMSFTASGSDYAMKNNVFL

300   SNSTLMGDVAFTSTWNSNFDPNGHDSNGDGVKDTNGGWTDDSLNVDELNL

350   TLDNGSKWVGQAIYNVAETSAMYDVATNSLTPDATYENNDWKRVVDDKVF
```

```
400   QSGVFNVALNNGSEWDTTGRSIVDTLTVNNGSQVNVSESKLTSDTIDLTN

450   GSSLNIGEDGYVDTDHLTINSYSTVALTESTGWGADYNLYANTITVTNGG

500   VLDVNVDQFDTEAFRTDKLELTSGNIADHNGNVVSGVFDIHSSDYVLNAD

550   LVNDRTWDTSKSNYGYGIVAMNSDGHLTINGNGDVDNGTELDNSSVDNVV

600   AATGNYKVRIDNATGAGAIADYKDKEIIYVNDVNSNATFSAANKADLGAY

650   TYQAEQRGNTVVLQQMELTDYANMALSIPSANTNIWNLEQDTVGTRLTNS

700   RHGLADNGGAWVSYFGGNFNGDNGTINYDQDVNGIMVGVDTKIDGNNAKW

750   IVGAAAGFAKGDMNDRSGQVDQDSQTAYIYSSAHFANNVFVDGSLSYSHF

800   NNDLSATMSNGTYVDGSTNSDAWGFGLKAGYDFKLGDAGYVTPYGSVSGL
        ********************
850   FQSGDDYQLSNDMKVDGQSYDSMRYELGVDAGYTFTYSEDQALTPYFKLA

900   YVYDDSNNDNDVNGDSIDNGTEGSAVRVGLGTQFSFTKNFSAYTDANYLG

950   GGDVDQDWSANVGVKYTW
```

sp|P75987|IRAM_ECOLI Anti-adapter protein IraM OS=Escherichia coli (strain K12) OX=83333 GN=iraM PE=1 SV=1

```
0     MKWIVIDTVIQPTCGISFSAIWGNMKMIIWYQSTIFLPPGSIFTPVKSGI
                       *********************
50    ILKDKEYPITIYHIAPFNKDLWSLLKSSQECPPGESKITNKCLHNSCIIK

100   ICPYGLK
```

sp|P0AED5|UVRY_ECOLI Response regulator UvrY OS=Escherichia coli (strain K12) OX=83333 GN=uvrY PE=1 SV=1

```
0     MINVLLVDDHELVRAGIRRILEDIKGIKVVGEASCGEDAVKWCRTNAVDV

50    VLMDMSMPGIGGLEATRKIARSTADVKIIMLTVHTENPLPAKVMQAGAAG
                                        ****************
100   YLSKGAAPQEVVSAIRSVYSGQRYIASDIAQQMALSQIEPEKTESPFASL
      *****
150   SERELQIMLMITKGQKVNEISEQLNLSPKTVNSYRYRMFSKLNIHGDVEL

200   THLAIRHGLCNAETLSSQ
```

sp|P08200|IDH_ECOLI Isocitrate dehydrogenase [NADP] OS=Escherichia coli (strain K12) OX=83333 GN=icd PE=1 SV=1

```
0     MESKVVVPAQGKKITLQNGKLNVPENPIIPYIEGDGIGVDVTPAMLKVVD

50    AAVEKAYKGERKISWMEIYTGEKSTQVYGQDVWLPAETLDLIREYRVAIK
```

```
100   GPLTTPVGGGIRSLNVALRQELDLYICLRPVRYYQGTPSPVKHPELTDMV
         **********************************
150   IFRENSEDIYAGIEWKADSADAEKVIKFLREEMGVKKIRFPEHCGIGIKP

200   CSEEGTKRLVRAAIEYAIANDRDSVTLVHKGNIMKFTEGAFKDWGYQLAR

250   EEFGGELIDGGPWLKVKNPNTGKEIVIKDVIADAFLQQILLRPAEYDVIA

300   CMNLNGDYISDALAAQVGGIGIAPGANIGDECALFEATHGTAPKYAGQDK

350   VNPGSIILSAEMMLRHMGWTEAADLIVKGMEGAINAKTVTYDFERLMDGA

400   KLLKCSEFGDAIIENM
```

sp|O52982|YFJS_ECOLI Lipoprotein YfjS OS=Escherichia coli (strain K12) OX=83333 GN=yfjS PE=1 SV=2

```
                   *******************************
0     MKRKTLPLLALVATSLFLSACDDRSDDLKAISKFKDLTPPRFSDVVSRQD

50    DVSEEWSQVGFSSGLTLQVLRTRESPDGCEGGSYYYLVDMEEKTVQPLMN

100   ALCIADNIKLEYHEVTDPYTKEKYFEYSHDGKLMGRLLIPSNPDNRE
```

sp|P76393|YEGI_ECOLI Protein kinase YegI OS=Escherichia coli (strain K12) OX=83333 GN=yegI PE=1 SV=1

```
0     MKTNIKVFTSTGELTTLGRELGKGGEGAVYDIEEFVDSVAKIYHTPPPAL

50    KQDKLAFMAATADAQLLNYVAWPQATLHGGRGGKVIGFMMPKVSGKEPIH

100   MIYSPAHRRQSYPHCAWDFLLYVARNIASSFATVHEHGHVVGDVNQNSFM

150   VGRDSKVVLIDSDSFQINANGTLHLCEVGVSHFTPPELQTLPSFVGFERT

200   ENHDNFGLALLIFHVLFGGRHPYSGVPLISDAGNALETDITHFRYAYASD

250   NQRRGLKPPPRSIPLSMLPSDVEAMFQQAFTESGVATGRPTAKAWVAALD

300   SLRQQLKKCIVSAMHVYPAHLTDCPWCALDNQGVIYFIDLGEEVITTGGN

350   FVLAKVWAMVMASVAPPALQLPLPDHFQPTGRPLPLGLLRREYIILLEIA

400   LSALSLLLCGLQAEPRYIILVPVLAAIWIIGSLTSKAYKAEVQQRREAFN
                              ********************
450   RAKMDYDHLVRQIQQVGGLEGFIAKRTMLEKMKDEILGLPEEEKRALAAL

500   HDTARERQKQKFLEGFFIDVASIPGVGPARKAALRSFGIETAADVTRRGV

550   KQVKGFGDHLTQAVIDWKASCERRFVFRPNEAITPADRQAVMAKMTAKRH

600   RLESALTVGATELQRFRLHAPARTMPLMEPLRQAAEKLAQAQADLSRC
```

sp|P52132|YFJQ_ECOLI UPF0380 protein YfjQ OS=Escherichia coli (strain K12) OX=83333 GN=yfjQ PE=3 SV=1

****
0 MTRLASRFGAANLIRRDRPLTREELFRVVPSVFSEDKHESRSERYTYIPT

50 ISLLDSLQREGFQPFFACQTRVRDPRRREHTKHMLRLRREGQITGKQVPE

100 IILLNSHDGTSSYQMLPGMFRAVCQNGLVCGESFGEVRVPHKGDVVSQVI

150 EGAYEVLGIFERVEEKRDAMQSLLLPPPVQQALAKAALTYRFGEDHQPVT

200 ESQILSPRRWQDESNDLWTTYQRIQENLIKGGLSGRNAKGGRTHTRAVRG

250 IDGDVKLNRALWVMAETLLTQLQ

sp|P32135|YIHN_ECOLI Inner membrane protein YihN OS=Escherichia coli (strain K12) OX=83333 GN=yihN PE=1 SV=1

0 MLTKKKWALFSLLTLCGGTIYKLPSLKDAFYIPMQEYFHLTNGQIGNAMS

50 VNSFVTTVGFFLSIYFADKLPRRYTMSFSLIATGLLGVYLTTMPGYWGIL

100 FVWALFGVTCDMMNWPVLLKSVSRLGNSEQQGRLFGFFETGRGIVDTVVA
****
150 FSALAVFTWFGSGLLGFKAGIWFYSLIVIAVGIIIFFVLNDKEEAPSVEV
****
200 KKEDGASKNTSMTSVLKDKTIWLIAFNVFFVYAVYCGLTFFIPFLKNIYL

250 LPVALVGAYGIINQYCLKMIGGPIGGMISDKILKSPSKYLCYTFIISTAA

300 LVLLIMLPHESMPVYLGMACTLGFGAIVFTQRAVFFAPIGEAKIAENKTG

350 AAMALGSFIGYAPAMFCFSLYGYILDLNPGIIGYKIVFGIMACFAFSGAV

400 VSVMLVKRISQRKKEMLAAEA

sp|P45470|YHBO_ECOLI Protein/nucleic acid deglycase 2 OS=Escherichia coli (strain K12) OX=83333 GN=yhbO PE=1 SV=2
******************************* ***********
0 MSKKIAVLITDEFEDSEFTSPADEFRKAGHEVITIEKQAGKTVKGKKGEA
****
50 SVTIDKSIDEVTPAEFDALLLPGGHSPDYLRGDNRFVTFTRDFVNSGKPV

100 FAICHGPQLLISADVIRGRKLTAVKPIIIDVKNAGAEFYDQEVVVDKDQL

150 VTSRTPDDLPAFNREALRLLGA

sp|P0AGB3|RPOH_ECOLI RNA polymerase sigma factor RpoH OS=Escherichia coli (strain K12) OX=83333 GN=rpoH PE=1 SV=1

0 MTDKMQSLALAPVGNLDSYIRAANAWPMLSADEERALAEKLHYHGDLEAA

50 KTLILSHLRFVVHIARNYAGYGLPQADLIQEGNIGLMKAVRRFNPEVGVR

100 LVSFAVHWIKAEIHEYVLRNWRIVKVATTKAQRKLFFNLRKTKQRLGWFN
****

150 QDEVEMVARELGVTSKDVREMESRMAAQDMTFDLSSDDDSDSQPMAPVLY
****
200 LQDKSSNFADGIEDDNWEEQAANRLTDAMQGLDERSQDIIRARWLDEDNK

250 STLQELADRYGVSAERVRQLEKNAMKKLRAAIEA

sp|P75749|YBGP_ECOLI Uncharacterized fimbrial chaperone YbgP OS=Escherichia coli (strain K12) OX=83333 GN=ybgP PE=3 SV=1
*****
0 MTFIKGLPLMLLTISLGCNAAVQPDRTRIVFNANDKATSLRIENQSDKLP
****
50 YLAYSWIENEKGEKSDALLVALPPIQRLEPKATSQVRVVKQASTTQLPGD

100 RETLFFYNMREIPPAPDKSSDHAILQVAIQSRIKLFWRPAALRKKAGEKV

150 ELQLQVSQQGNQLTLKNPTAYYLTIAYLGRNEKGVLPGFKTVMVAPFSTV

200 NTNTGSYSGSQFYLGYMDDYGALRMTTLNCSGQCRLQAVEAK

sp|P76335|YEDS_ECOLI Putative outer membrane protein YedS OS=Escherichia coli (strain K12) OX=83333 GN=yedS PE=5 SV=4
******
0 MKRKVLAMLVPALLVAGAANAAEVYNKDGNKLDLYGKVVGLHYFSDDSGS
****
50 DGDMSYARIGFKGETQIADQFTGYGQWEFNIGANGPESDKGNTATRLAFA

100 GLGFGQNGTFDYGRNYGVVYDVEAWTDMLPEFGGDTYAGADNFMNGRANG

150 VATYRNNGFFG

sp|Q2EES0|YNFO_ECOLI Uncharacterized protein YnfO OS=Escherichia coli (strain K12) OX=83333 GN=ynfO PE=4 SV=1
******
0 MSTKNRTRRTTTRNIRFPNQMIEQINIALEQKGSGNFSAWVIEACRRRLC
****
50 SEKRVSSEANKEKSDITELLRKQVRPD

sp|P37645|YHJG_ECOLI AsmA family protein YhjG OS=Escherichia coli (strain K12) OX=83333 GN=yhjG PE=3 SV=3

0 MSKAGKITAAISGAFLLLIVVAIILIATFDWNRLKPTINQKVSAELNRPF

50 AIRGDLGVVWERQKQETGWRSWVPWPHVHAEDIILGNPPDIPEVTMVHLP

100 RVEATLAPLALLTKTVWLPWIKLEKPDARLIRLSEKNNNWTFNLANDDNK
****
150 DANAKPSAWSFRLDNILFDQGRIAIDDKVSKADLEIFVDPLGKPLPFSEV
****
200 TGSKGKADKEKVGDYVFGLKAQGRYNGEPLTGTGKIGGMLALRGEGTPFP

250 VQADFRSGNTRVAFDGVVNDPMKMGGVDLRLKFSGDSLGDLYELTGVLLP

300 DTPPFETDGRLVAKIDTEKSSVFDYRGFNGRIGDSDIHGSLVYTTGKPRP
****

350 KLEGDVESRQLRLADLGPLIGVDSGKGAEKSKRSEQKKGEKSVQPAGKVL

400 PYDRFETDKWDVMDADVRFKGRRIEHGSSLPISDLSTHIILKNADLRLQP

450 LKFGMAGGSIAANIHLEGDKKPMQGRADIQARRLKLKELMPDVELMQKTL

500 GEMNGDAELRGSGNSVAALLGNSNGNLKLLMNDGLVSRNLMEIVGLNVGN

550 YIVGAIFGDDEVRVNCAAANLNIANGVARPQIFAFDTENALINVTGTASF

600 ASEQLDLTIDPESKGIRIITLRSPLYVRGTFKNPQAGVKAGPLIARGAVA

650 AALATLVTPAAALLALISPSEGEANQCRTILSQMKK

sp|P25522|MNME_ECOLI tRNA modification GTPase MnmE OS=Escherichia coli (strain K12) OX=83333 GN=mnmE PE=1 SV=3

0 MSDNDTIVAQATPPGRGGVGILRISGFKAREVAETVLGKLPKPRYADYLP

50 FKDADGSVLDQGIALWFPGPNSFTGEDVLELQGHGGPVILDLLLKRILTI

100 PGLRIARPGEFSERAFLNDKLDLAQAEAIADLIDASSEQAARSALNSLQG
****
150 AFSARVNHLVEALTHLRIYVEAAIDFPDEEIDFLSDGKIEAQLNDVIADL

200 DAVRAEARQGSLLREGMKVVIAGRPNAGKSSLLNALAGREAAIVTDIAGT

250 TRDVLREHIHIDGMPLHIIDTAGLREASDEVERIGIERAWQEIEQADRVL

300 FMVDGTTTDAVDPAEIWPEFIARLPAKLPITVVRNKADITGETLGMSEVN

350 GHALIRLSARTGEGVDVLRNHLKQSMGFDTNMEGGFLARRRHLQALEQAA

400 EHLQQGKAQLLGAWAGELLAEELRLAQQNLSEITGEFTSDDLLGRIFSSF

450 CIGK

sp|P76545|YFFN_ECOLI Uncharacterized protein YffN OS=Escherichia coli (strain K12) OX=83333 GN=yffN PE=4 SV=2
****
0 MKHVFKYLDFAEDREHAESVATKELKLDHVEKFAIRDLANDIKERGCVEL

50 VQPGGFDELVQIYEAGGDGIEPLNCGIESRKVAIAALLRVMREPDFQCLE

100 MVHEIIRIARDLEAPVDAPLDC

sp|P0A9R4|FER_ECOLI 2Fe-2S ferredoxin OS=Escherichia coli (strain K12) OX=83333 GN=fdx PE=1 SV=2

0 MPKIVILPHQDLCPDGAVLEANSGETILDAALRNGIEIEHACEKSCACTT
****
50 CHCIVREGFDSLPESSEQEDDMLDKAWGLEPESRLSCQARVTDEDLVVEI

100 PRYTINHAREH

sp|P75967|YMFD_ECOLI Uncharacterized protein YmfD OS=Escherichia coli (strain K12) OX=83333 GN=ymfD PE=4 SV=1
                                                       *
0   MVLALNYNMHGVNIRSENAAKPHTMPSRYLCEYIRSIEKNGHALDFGCGK
      ****
50   LRYSDELISKFDEVTFLDSKRQLEREQIIRGIKTKIIDYVPRYYKNANTV

100   AFEDVDKIIGGYDFILCSNVLSAVPCRDTIDKIVLSIKRLLKSGGETLIV
                                               ****
150   NQYKSSYFKKYETGRKHLYGYIYKNSKSVSYYGLLDELAVQEICSSHGLE
      ****
200   ILKSWSKAGSSYVTVGSCNAI

sp|P33937|NAPA_ECOLI Periplasmic nitrate reductase OS=Escherichia coli (strain K12) OX=83333 GN=napA PE=1 SV=3

0   MKLSRRSFMKANAVAAAAAAAGLSVPGVARAVVGQQEAIKWDKAPCRFCG

50   TGCGVLVGTQQGRVVACQGDPDAPVNRGLNCIKGYFLPKIMYGKDRLTQP
                            ****
100   LLRMKNGKYDKEGEFTPITWDQAFDVMEEKFKTALKEKGPESIGMFGSGQ

150   WTIWEGYAASKLFKAGFRSNNIDPNARHCMASAVVGFMRTFGMDEPMGCY

200   DDIEQADAFVLWGANMAEMHPILWSRITNRRLSNQNVTVAVLSTYQHRSF

250   ELADNGIIFTPQSDLVILNYIANYIIQNNAINQDFFSKHVNLRKGATDIG
              ****
300   YGLRPTHPLEKAAKNPGSDASEPMSFEDYKAFVAEYTLEKTAEMTGVPKD

350   QLEQLAQLYADPNKKVISYWTMGFNQHTRGVWANNLVYNLHLLTGKISQP

400   GCGPFSLTGQPSACGTAREVGTFAHRLPADMVVTNEKHRDICEKKWNIPS

450   GTIPAKIGLHAVAQDRALKDGKLNVYWTMCTNNMQAGPNINEERMPGWRD

500   PRNFIIVSDPYPTVSALAADLILPTAMWVEKEGAYGNAERRTQFWRQQVQ
                                                      **
550   APGEAKSDLWQLVQFSRRFKTEEVWPEDLLAKKPELRGKTLYEVLYATPE
      ****
600   VSKFPVSELAEDQLNDESRELGFYLQKGLFEEYAWFGRGHGHDLAPFDDY

650   HKARGLRWPVVNGKETQWRYSEGNDPYVKAGEGYKFYGKPDGKAVIFALP

700   FEPAAEAPDEEYDLWLSTGRVLEHWHTGSMTRRVPELHRAFPEAVLFIHP

750   LDAKARDLRRGDKVKVVSRRGEVISIVETRGRNRPPQGLVYMPFFDAAQL
            ****
800   VNKLTLDATDPLSKETDFKKCAVKLEKV

sp|P43671|PQIB_ECOLI Intermembrane transport protein PqiB OS=Escherichia coli (strain K12) OX=83333 GN=pqiB PE=1 SV=2

```
0     MESNNGEAKIQKVKNWSPVWIFPIVTALIGAWVLFYHYSHQGPEVTLITA

50    NAEGIEGGKTTIKSRSVDVGVVESATLADDLTHVEIKARLNSGMEKLLHK

100   DTVFWVVKPQIGREGISGLGTLLSGVYIELQPGAKGSKMDKYDLLDSPPL

150   APPDAKGIRVILDSKKAGQLSPGDPVLFRGYRVGSVETSTFDTQKRNISY

200   QLFINAPYDRLVTNNVRFWKDSGIAVDLTSAGMRVEMGSLTTLLSGGVSF

250   DVPEGLDLGQPVAPKTAFVLYDDQKSIQDSLYTDHIDYLMFFKDSVRGLQ

300   PGAPVEFRGIRLGTVSKVPFFAPNMRQTFNDDYRIPVLIRIEPERLKMQL

350   GENADVVEHLGELLKRGLRGSLKTGNLVTGALYVDLDFYPNTPAITGIRE

400   FNGYQIIPTVSGGLAQIQQRLMEALDKINKLPLNPMIEQATSTLSESQRT

450   MKNLQTTLDSMNKILASQSMQQLPTDMQSTLRELNRSMQGFQPGSAAYNK
                        ***************************
500   MVADMQRLDQVLRELQPVLKTLNEKSNALVFEAKDKKDPEPKRAKQ
```

sp|P30130|FIMD_ECOLI Outer membrane usher protein FimD OS=Escherichia coli (strain K12) OX=83333 GN=fimD PE=1 SV=2

```
0     MSYLNLRLYQRNTQCLHIRKHRLAGFFVRLVVACAFAAQAPLSSADLYFN

50    PRFLADDPQAVADLSRFENGQELPPGTYRVDIYLNNGYMATRDVTFNTGD

100   SEQGIVPCLTRAQLASMGLNTASVAGMNLLADDACVPLTTMVQDATAHLD

150   VGQQRLNLTIPQAFMSNRARGYIPPELWDPGINAGLLNYNFSGNSVQNRI
                            *********************
200   GGNSHYAYLNLQSGLNIGAWRLRDNTTWSYNSSDRSSGSKNKWQHINTWL

250   ERDIIPLRSRLTLGDGYTQGDIFDGINFRGAQLASDDNMLPDSQRGFAPV

300   IHGIARGTAQVTIKQNGYDIYNSTVPPGPFTINDIYAAGNSGDLQVTIKE

350   ADGSTQIFTVPYSSVPLLQREGHTRYSITAGEYRSGNAQQEKTRFFQSTL

400   LHGLPAGWTIYGGTQLADRYRAFNFGIGKNMGALGALSVDMTQANSTLPD

450   DSQHDGQSVRFLYNKSLNESGTNIQLVGYRYSTSGYFNFADTTYSRMNGY

500   NIETQDGVIQVKPKFTDYYNLAYNKRGKLQLTVTQQLGRTSTLYLSGSHQ

550   TYWGTSNVDEQFQAGLNTAFEDINWTLSYSLTKNAWQKGRDQMLALNVNI
            *********************
600   PFSHWLRSDSKSQWRHASASYSMSHDLNGRMTNLAGVYGTLLEDNNLSYS

650   VQTGYAGGGDGNSGSTGYATLNYRGGYGNANIGYSHSDDIKQLYYGVSGG

700   VLAHANGVTLGQPLNDTVVLVKAPGAKDAKVENQTGVRTDWRGYAVLPYA
```

750   TEYRENRVALDTNTLADNVDLDNAVANVVPTRGAIVRAEFKARVGIKLLM

800   TLTHNNKPLPFGAMVTSESSQSSGIVADNGQVYLSGMPLAGKVQVKWGEE

850   ENAHCVANYQLPPESQQQLLTQLSAECR

sp|P16917|RHSB_ECOLI Protein RhsB OS=Escherichia coli (strain K12) OX=83333 GN=rhsB PE=3 SV=4

0   MSGKPAARQGDMTQYGGSIVQGSAGVRIGAPTGVACSVCPGGVTSGHPVN

50   PLLGAKVLPGETDIALPGPLPFILSRTYSSYRTKTPAPVGSLGPGWKMPA

100   DIRLQLRDNTLILSDNGGRSLYFEHLFPGEDGYSRSESLWLVRGGVAKLD

150   EGHRLAALWQALPEELRLSPHRYLATNSPQGPWWLLGWCERVPEADEVLP

200   APLPPYRVLTGLVDRFGRTQTFHREAAGEFSGEITGVTDGAWRHFRLVLT

250   TQAQRAEEARQQAISGGTEPSAFPDTLPGYTEYGRDNGIRLSAVWLTHDP

300   EYPENLPAAPLVRYGWTPRGELAVVYDRSGKQVRSFTYDDKYRGRMVAHR

350   HTGRPEIRYRYDSDGRVTEQLNPAGLSYTYQYEKDRITITDSLDRREVLH

400   TQGEAGLKRVVKKEHADGSVTQSQFDAVGRLRAQTDAAGRTTEYSPDVVT

450   GLITRITTPDGRASAFYYNHHNQLTSATGPDGLELRREYDELGRLIQETA

500   PDGDITRYRYDNPHSDLPCATEDATGSRKTMTWSRYGQLLSFTDCSGYVT

550   RYDHDRFGQMTAVHREEGLSQYRAYDSRGQLIAVKDTQGHETRYEYNIAG

600   DLTAVIAPDGSRNGTQYDAWGKAVRTTQGGLTRSMEYDAAGRVIRLTSEN

650   GSHTTFRYDVLDRLIQETGFDGRTQRYHHDLTGKLIRSEDEGLVTHWHYD

700   EADRLTHRTVKGETAERWQYDERGWLTDISHISEGHRVAVHYRYDEKGRL

750   TGERQTVHHPQTEALLWQHETRHAYNAQGLANRCIPDSLPAVEWLTYGSG

800   YLAGMKLGDTPLVEYTRDRLHRETLRSFGRYELTTAYTPAGQLQSQHLNS

850   LLSDRDYTWNDNGELIRISSPRQTRSYSYSTTGRLTGVHTTAANLDIRIP

900   YATDPAGNRLPDPELHPDSTLSMWPDNRIARDAHYLYRYDRHGRLTEKTD

950   LIPEGVIRTDDERTHRYHYDSQHRLVHYTRTQYEEPLVESRYLYDPLGRR

1000 VAKRVWRRERDLTGWMSLSRKPQVTWYGWDGDRLTTIQNDRSRIQTIYQP

1050 GSFTPLIRVETATGELAKTQRRSLADALQQSGGEDGGSVVFPPVLVQMLD

1100 RLESEILADRVSEESRRWLASCGLTVEQMQNQMDPVYTPARKIHLYHCDH

1150 RGLPLALISTEGATAWCAEYDEWGNLLNEENPHQLQQLIRLPGQQYDEES

1200 GLYYNRHRYYDPLQGRYITQDPIGLKGGWNLYGYQLNPISDIDPLGLSMW

1250 EDAKSGACTNGLCGTLSAMIGPDKFDSIDSTAYDALNKINSQSICEDKEF
                                            ******
1300 AGLICKDNSGRYFSTAPNRGERKGSYPFNSPCPNGTEKVSAYHTHGADSH
****
1350 GEYWDEIFSGKDEKIVKSKDNNIKSFYLGTPSGNFKAIDNHGKEITNRKG

1400 LPNVCRVHGNM

sp|P0AF56|YJCO_ECOLI Sel1-repeat-containing protein YjcO OS=Escherichia coli (strain K12) OX=83333 GN=yjcO PE=3 SV=1
                                              ****
0 MKKIIALMLFLTFFAHANDSEPGSQYLKAAEAGDRRAQYFLADSWFSSGD
****
50 LSKAEYWAQKAADSGDADACALLAQIKITNPVSLDYPQAKVLAEKAAQAG
                    ****
100 SKEGEVTLAHILVNTQAGKPDYPKAISLLENASEDLENDSAVDAQMLLGL

150 IYANGVGIKADDDKATWYFKRSSAISRTGYSEYWAGMMFLNGEEGFIEKN

200 KQKALHWLNLSCMEGFDTGCEEFEKLTNG

sp|P33595|SGRR_ECOLI HTH-type transcriptional regulator SgrR OS=Escherichia coli (strain K12) OX=83333 GN=sgrR PE=1 SV=2

0 MPSARLQQQFIRLWQCCEGKSQDTTLNELAALLSCSRRHMRTLLNTMQDR

50 GWLTWEAEVGRGKRSRLTFLYTGLALQQQRAEDLLEQDRIDQLVQLVGDK

100 ATVRQMLVSHLGRSFRQGRHILRVLYYRPLRNLLPGSALRRSETHIARQI

150 FSSLTRINEENGELEADIAHHWQQISPLHWRFFLRPGVHFHHGRELEMDD

200 VIASLKRINTLPLYSHIADIVSPTPWTLDIHLTQPDRWLPLLLGQVPAMI

250 LPREWETLSNFASHPIGTGPYAVIRNSTNQLKIQAFDDFFGYRALIDEVN
                      ****
300 VWVLPEIADEPAGGLMLKGPQGEEKEIESRLEEGCYYLLFDSRTHRGANQ

350 QVRDWVSYVLSPTNLVYFAEEQYQQLWFPAYGLLPRWHHARTIKSEKPAG

400 LESLTLTFYQDHSEHRVIAGIMQQILASHQVTLKIKEIDYDQWHTGEIES

450 DIWLNSANFTLPLDFSVFAHLCEVPLLQHCIPIDWQADAARWRNGEMNLA

500 NWCQQLVASKAMVPLLHHWLIIQGQRSMRGLRMNTLGWFDFKSAWFAPPD

550 P

sp|P77774|BAMB_ECOLI Outer membrane protein assembly factor BamB OS=Escherichia coli (strain K12) OX=83333 GN=bamB PE=1 SV=1

```
0     MQLRKLLLPGLLSVTLLSGCSLFNSEEDVVKMSPLPTVENQFTPTTAWST
                                     *******************
50    SVGSGIGNFYSNLHPALADNVVYAADRAGLVKALNADDGKEIWSVSLAEK
      *********
100   DGWFSKEPALLSGGVTVSGGHVYIGSEKAQVYALNTSDGTVAWQTKVAGE

150   ALSRPVVSDGLVLIHTSNGQLQALNEADGAVKWTVNLDMPSLSLRGESAP

200   TTAFGAAVVGGDNGRVSAVLMEQGQMIWQQRISQATGSTEIDRLSDVDTT

250   PVVVNGVVFALAYNGNLTALDLRSGQIMWKRELGSVNDFIVDGNRIYLVD

300   QNDRVMALTIDGGVTLWTQSDLLHRLLTSPVLYNGNLVVGDSEGYLHWIN

350   VEDGRFVAQQKVDSSGFQTEPVAADGKLLIQAKDGTVYSITR
```

sp|P46889|FTSK_ECOLI DNA translocase FtsK OS=Escherichia coli (strain K12) OX=83333 GN=ftsK PE=1 SV=2

```
0     MSQEYIEDKEVTLTKLSSGRRLLEALLILIVLFAVWLMAALLSFNPSDPS

50    WSQTAWHEPIHNLGGMPGAWLADTLFFIFGVMAYTIPVIIVGGCWFAWRH

100   QSSDEYIDYFAVSLRIIGVLALILTSCGLAAINADDIWYFASGGVIGSLL

150   STTLQPLLHSSGGTIALLCVWAAGLTLFTGWSWVTIAEKLGGWILNILTF
                ****************************
200   ASNRTRRDDTWVDEDEYEDDEEYEDENHGKQHESRRARILRGALARRKRL

250   AEKFINPMGRQTDAALFSGKRMDDDEEITYTARGVAADPDDVLFSGNRAT

300   QPEYDEYDPLLNGAPITEPVAVAAAATTATQSWAAPVEPVTQTPPVASVD

350   VPPAQPTVAWQPVPGPQTGEPVIAPAPEGYPQQSQYAQPAVQYNEPLQQP

400   VQPQQPYYAPAAEQPAQQPYYAPAPEQPVAGNAWQAEEQQSTFAPQSTYQ

450   TEQTYQQPAAQEPLYQQPQPVEQQPVVEPEPVVEETKPARPPLYYFEEVE

500   EKRAREREQLAAWYQPIPEPVKEPEPIKSSLKAPSVAAVPPVEAAAAVSP

550   LASGVKKATLATGAAATVAAPVFSLANSGGPRPQVKEGIGPQLPRPKRIR

600   VPTRRELASYGIKLPSQRAAEEKAREAQRNQYDSGDQYNDDEIDAMQQDE

650   LARQFAQTQQQRYGEQYQHDVPVNAEDADAAAEAELARQFAQTQQQRYSG

700   EQPAGANPFSLDDFEFSPMKALLDDGPHEPLFTPIVEPVQQPQQPVAPQQ

750   QYQQPQQPVPPQPQYQQPQQPVAPQPQYQQPQQPVAPQQQYQQPQQPVAP
```

```
800   QQQYQQPQQPVAPQPQDTLLHPLLMRNGDSRPLHKPTTPLPSLDLLTPPP

850   SEVEPVDTFALEQMARLVEARLADFRIKADVVNYSPGPVITRFELNLAPG

900   VKAARISNLSRDLARSLSTVAVRVVEVIPGKPYVGLELPNKKRQTVYLRE

950   VLDNAKFRDNPSPLTVVLGKDIAGEPVVADLAKMPHLLVAGTTGSGKSVG

1000  VNAMILSMLYKAQPEDVRFIMIDPKMLELSVYEGIPHLLTEVVTDMKDAA

1050  NALRWCVNEMERRYKLMSALGVRNLAGYNEKIAEADRMMRPIPDPYWKPG

1100  DSMDAQHPVLKKEPYIVVLVDEFADLMMTVGKKVEELIARLAQKARAAGI

1150  HLVLATQRPSVDVITGLIKANIPTRIAFTVSSKIDSRTILDQAGAESLLG

1200  MGDMLYSGPNSTLPVRVHGAFVRDQEVHAVVQDWKARGRPQYVDGITSDS

1250  ESEGGAGGFDGAEELDPLFDQAVQFVTEKRKASISGVQRQFRIGYNRAAR

1300  IIEQMEAQGIVSEQGHNGNREVLAPPPFD
```

sp|P0A8E7|YAJQ_ECOLI UPF0234 protein YajQ OS=Escherichia coli (strain K12) OX=83333 GN=yajQ PE=1 SV=2

```
                                        ****************
0     MPSFDIVSEVDLQEARNAVDNASREVESRFDFRNVEASFELNDASKTIKV
      *******                                         **
50    LSESDFQVNQLLDILRAKLLKRGIEGSSLDVPENIVHSGKTWFVEAKLKQ
      **********************
100   GIESATQKKIVKMIKDSKLKVQAQIQGDEIRVTGKSRDDLQAVMAMVRGG

150   DLGQPFQFKNFRD
```

sp|P25894|LOIP_ECOLI Metalloprotease LoiP OS=Escherichia coli (strain K12) OX=83333 GN=loiP PE=1 SV=2

```
0     MKIRALLVAMSVATVLTGCQNMDSNGLLSSGAEAFQAYSLSDAQVKTLSD

50    QACQEMDSKATIAPANSEYAKRLTTIANALGNNINGQPVNYKVYMAKDVN

100   AFAMANGCIRVYSGLMDMMTDNEVEAVIGHEMGHVALGHVKKGMQVALGT

150   NAVRVAAASAGGIVGSLSQSQLGNLGEKLVNSQFSQRQEAEADDYSYDLL
                 *********************
200   RQRGISPAGLATSFEKLAKLEEGRQSSMFDDHPASAERAQHIRDRMSADG

250   IK
```

sp|P76547|YFFP_ECOLI Uncharacterized protein YffP OS=Escherichia coli (strain K12) OX=83333 GN=yffP PE=4 SV=1

```
0     MSLIRTETRDTKRAADPLHDLRSKPFSEWGEDEIRRFNLIDALLEFVYTD

50    TSSPFGIGMTFDYTECWEIGVRDDCLVMTRVKPVHPEYAKHWNMKGVMND
```

```
100   KTRFHADKWVGYSKVLAWVSLSHKDTFTGAKRFQYFQTMYDMERQINANL
                         **********************
150   PVGGLPNVDTERTGKLFQRDDFSEDSHANDPKLVGDDYVPQAPEQIN
```

sp|P0ACB0|DNAB_ECOLI Replicative DNA helicase OS=Escherichia coli (strain K12) OX=83333 GN=dnaB PE=1 SV=1

```
0     MAGNKPFNKQQAEPRERDPQVAGLKVPPHSIEAEQSVLGGLMLDNERWDD

50    VAERVVADDFYTRPHRHIFTEMARLQESGSPIDLITLAESLERQGQLDSV

100   GGFAYLAELSKNTPSAANISAYADIVRERAVVREMISVANEIAEAGFDPQ

150   GRTSEDLLDLAESRVFKIAESRANKDEGPKNIADVLDATVARIEQLFQQP

200   HDGVTGVNTGYDDLNKKTAGLQPSDLIIVAARPSMGKTTFAMNLVENAAM

250   LQDKPVLIFSLEMPSEQIMMRSLASLSRVDQTKIRTGQLDDEDWARISGT

300   MGILLEKRNIYIDDSSGLTPTEVRSRARRIAREHGGIGLIMIDYLQLMRV
                                                 *******
350   PALSDNRTLEIAEISRSLKALAKELNVPVVALSQLNRSLEQRADKRPVNS
      *************
400   DLRESGSIEQDADLIMFIYRDEVYHENSDLKGIAEIIIGKQRNGPIGTVR

450   LTFNGQWSRFDNYAGPQYDDE
```

sp|P77757|ARNC_ECOLI Undecaprenyl-phosphate 4-deoxy-4-formamido-L-arabinose transferase OS=Escherichia coli (strain K12) OX=83333 GN=arnC PE=1 SV=1

```
                                      ******************
0     MFEIHPVKKVSVVIPVYNEQESLPELIRRTTTACESLGKEYEILLIDDGS
      *****
50    SDNSAHMLVEASQAENSHIVSILLNRNYGQHSAIMAGFSHVTGDLIITLD

100   ADLQNPPEEIPRLVAKADEGYDVVGTVRQNRQDSWFRKTASKMINRLIQR

150   TTGKAMGDYGCMLRAYRRHIVDAMLHCHERSTFIPILANIFARRAIEIPV

200   HHAEREFGESKYSFMRLINLMYDLVTCLTTTPLRMLSLLGSIIAIGGFSI

250   AVLLVILRLTFGPQWAAEGVFMLFAVLFTFIGAQFIGMGLLGEYIGRIYT

300   DVRARPRYFVQQVIRPSSKENE
```

sp|P71237|WCAC_ECOLI Putative colanic acid biosynthesis glycosyl transferase WcaC OS=Escherichia coli (strain K12) OX=83333 GN=wcaC PE=4 SV=2

```
0     MNILQFNVRLAEGGAAGVALDLHQRALQQGLASHFVYGYGKGGKESVSHQ

50    NYPQVIKHTPRMTAMANIALFRLFNRDLFGNFNELYRTITRTAGPVVLHF
```

```
100   HVLHSYWLNLKSVVRFCEKVKNHKPDVTLVWTLHDHWSVTGRCAFTDGCE

150   GWKTGCQKCPTLNNYPPVKIDRAHQLVAGKRQLFREMLALGCQFISPSQH

200   VADAFNSLYGPGRCRIINNGIDMATEAILADLPPVRETQGKPKIAVVAHD

250   LRYDGKTNQQLVREMMALGDKIELHTFGKFSPFTAGNVVNHGFETDKRKL
                                         *************
300   MSALNQMDALVFSSRVDNYPLILCEALSIGVPVIATHSDAAREVLQKSGG
      *******
350   KTVSEEEVLQLVQLSKPEIAQAIFGTTLAEFSQRSRAAYSGQQMLEEYVN

400   FYQNL
```

sp|P52007|YECM_ECOLI Protein YecM OS=Escherichia coli (strain K12) OX=83333 GN=yecM PE=1 SV=3

```
0     MANWQSIDELQDIASDLPRFIHALDELSRRLGLNITPLTADHISLRCHQN

50    ATAERWRRGFEQCGELLSENMINGRPICLFKLHEPVQVAHWQFSIVELPW
                                       *****************
100   PGEKRYPHEGWEHIEIVLPGDPETLNARALALLSDEGLSLPGISVKTSSP
      ***
150   KGEHERLPNPTLAVTDGKTTIKFHPWSIEEIVASEQSA
```

sp|P76543|YFFL_ECOLI Uncharacterized protein YffL OS=Escherichia coli (strain K12) OX=83333 GN=yffL PE=4 SV=1

```
0     MFATKDPEFENRINTNKSPRNAATCRGRYEKQAKGEFLMSDMLAVEQETN
                     *************************
50    NDVRQFLNKINELRNKAPKNEETKHEEHTPDNHEETDHHEAKQQEQAWRG

100   NLRYLDTLNRLDEVLPRKLYERWEKEHTVNDEAVLRALCYFAGTGKNSQL

150   GWCRVGRGTIDKRARLSKNTVKKCLDRLVNHFKLVERTEGYIPGSAEREC

200   NEYQLLFKPYNMK
```

sp|P0A6H5|HSLU_ECOLI ATP-dependent protease ATPase subunit HslU OS=Escherichia coli (strain K12) OX=83333 GN=hslU PE=1 SV=1

```
0     MSEMTPREIVSELDKHIIGQDNAKRSVAIALRNRWRRMQLNEELRHEVTP

50    KNILMIGPTGVGKTEIARRLAKLANAPFIKVEATKFTEVGYVGKEVDSII

100   RDLTDAAVKMVRVQAIEKNRYRAEELAEERILDVLIPPAKNNWGQTEQQQ

150   EPSAARQAFRKKLREGQLDDKEIEIDLAAAPMGVEIMAPPGMEEMTSQLQ
               ********************
200   SMFQNLGGQKQKARKLKIKDAMKLLIEEEAAKLVNPEELKQDAIDAVEQH
         ***********************
250   GIVFIDEIDKICKRGESSGPDVSREGVQRDLLPLVEGCTVSTKHGMVKTD

300   HILFIASGAFQIAKPSDLIPELQGRLPIRVELQALTTSDFERILTEPNAS
```

350   ITVQYKALMATEGVNIEFTDSGIKRIAEAAWQVNESTENIGARRLHTVLE

400   RLMEEISYDASDLSGQNITIDADYVSKHLDALVADEDLSRFIL

sp|P0ADM4|YIDQ_ECOLI Uncharacterized protein YidQ OS=Escherichia coli (strain K12) OX=83333 GN=yidQ PE=3 SV=1

0   MIRNVLLAFMICSGMTLLGGCSSVMSHTGGKEGTYPGTRASATMIGDDET
                            ****
50   NWGTKSLAILDMPFTAVMDTLLLPWDVFRKDSSVRSRVEKSEANAQATNA

100   VIPPARMPDN

sp|P0AE52|BCP_ECOLI Peroxiredoxin Bcp OS=Escherichia coli (strain K12) OX=83333 GN=bcp PE=1 SV=1

0   MNPLKAGDIAPKFSLPDQDGEQVNLTDFQGQRVLVYFYPKAMTPGCTVQA
             ****
50   CGLRDNMDELKKAGVDVLGISTDKPEKLSRFAEKELLNFTLLSDEDHQVC

100   EQFGVWGEKSFMGKTYDGIHRISFLIDADGKIEHVFDDFKTSNHHDVVLN

150   WLKEHA

sp|P69441|KAD_ECOLI Adenylate kinase OS=Escherichia coli (strain K12) OX=83333 GN=adk PE=1 SV=1

0   MRIILLGAPGAGKGTQAQFIMEKYGIPQISTGDMLRAAVKSGSELGKQAK

50   DIMDAGKLVTDELVIALVKERIAQEDCRNGFLLDGFPRTIPQADAMKEAG
                                             ****
100   INVDYVLEFDVPDELIVDRIVGRRVHAPSGRVYHVKFNPPKVEGKDDVTG
      ****
150   EELTTRKDDQEETVRKRLVEYHQMTAPLIGYYSKEAEAGNTKYAKVDGTK

200   PVAEVRADLEKILG

sp|P11868|TDCD_ECOLI Propionate kinase OS=Escherichia coli (strain K12) OX=83333 GN=tdcD PE=1 SV=3
                  ****
0   MNEFPVVLVINCGSSSIKFSVLDASDCEVLMSGIADGINSENAFLSVNGG

50   EPAPLAHHSYEGALKAIAFELEKRNLNDSVALIGHRIAHGGSIFTESAII

100   TDEVIDNIRRVSPLAPLHNYANLSGIESAQQLFPGVTQVAVFDTSFHQTM

150   APEAYLYGLPWKYYEELGVRRYGFHGTSHRYVSQRAHSLLNLAEDDSGLV

200   VAHLGNGASICAVRNGQSVDTSMGMTPLEGLMMGTRSGDVDFGAMSWVAS

250   QTNQSLGDLERVVNKESGLLGISGLSSDLRVLEKAWHEGHERAQLAIKTF

300   VHRIARHIAGHAASLRRLDGIIFTGGIGENSSLIRRLVMEHLAVLGLEID

```
350   TEMNNRSNSCGERIVSSENARVICAVIPTNEEKMIALDAIHLGKVNAPAE

400   FA
```

sp|P00452|RIR1_ECOLI Ribonucleoside-diphosphate reductase 1 subunit alpha OS=Escherichia coli (strain K12) OX=83333 GN=nrdA PE=1 SV=2

```
0     MNQNLLVTKRDGSTERINLDKIHRVLDWAAEGLHNVSISQVELRSHIQFY

50    DGIKTSDIHETIIKAAADLISRDAPDYQYLAARLAIFHLRKKAYGQFEPP
                          ********************
100   ALYDHVVKMVEMGKYDNHLLEDYTEEEFKQMDTFIDHDRDMTFSYAAVKQ

150   LEGKYLVQNRVTGEIYESAQFLYILVAACLFSNYPRETRLQYVKRFYDAV

200   STFKISLPTPIMSGVRTPTRQFSSCVLIECGDSLDSINATSSAIVKYVSQ

250   RAGIGINAGRIRALGSPIRGGEAFHTGCIPFYKHFQTAVKSCSQGGVRGG

300   AATLFYPMWHLEVESLLVLKNNRGVEGNRVRHMDYGVQINKLMYTRLLKG
                       *********************************
350   EDITLFSPSDVPGLYDAFFADQEEFERLYTKYEKDDSIRKQRVKAVELFS

400   LMMQERASTGRIYIQNVDHCNTHSPFDPAIAPVRQSNLCLEIALPTKPLN

450   DVNDENGEIALCTLSAFNLGAINNLDELEELAILAVRALDALLDYQDYPI

500   PAAKRGAMGRRTLGIGVINFAYYLAKHGKRYSDGSANNLTHKTFEAIQYY

550   LLKASNELAKEQGACPWFNETTYAKGILPIDTYKKDLDTIANEPLHYDWE

600   ALRESIKTHGLRNSTLSALMPSETSSQISNATNGIEPPRGYVSIKASKDG

650   ILRQVVPDYEHLHDAYELLWEMPGNDGYLQLVGIMQKFIDQSISANTNYD

700   PSRFPSGKVPMQQLLKDLLTAYKFGVKTLYYQNTRDGAEDAQDDLVPSIQ

750   DDGCESGACKI
```

sp|P30850|RNB_ECOLI Exoribonuclease 2 OS=Escherichia coli (strain K12) OX=83333 GN=rnb PE=1 SV=3

```
0     MFQDNPLLAQLKQQLHSQTPRAEGVVKATEKGFGFLEVDAQKSYFIPPPQ
             ****************************
50    MKKVMHGDRIIAVIHSEKERESAEPEELVEPFLTRFVGKVQGKNDRLAIV

100   PDHPLLKDAIPCRAARGLNHEFKEGDWAVAEMRRHPLKGDRSFYAELTQY

150   ITFGDDHFVPWWVTLARHNLEKEAPDGVATEMLDEGLVREDLTALDFVTI
      *********************
200   DSASTEDMDDALFAKALPDDKLQLIVAIADPTAWIAEGSKLDKAAKIRAF

250   TNYLPGFNIPMLPRELSDDLCSLRANEVRPVLACRMTLSADGTIEDNIEF
```

300   FAATIESKAKLVYDQVSDWLENTGDWQPESEAIAEQVRLLAQICQRRGEW

350   RHNHALVFKDRPDYRFILGEKGEVLDIVAEPRRIANRIVEEAMIAANICA

400   ARVLRDKLGFGIYNVHMGFDPANADALAALLKTHGLHVDAEEVLTLDGFC

450   KLRRELDAQPTGFLDSRIRRFQSFAEISTEPGPHFGLGLEAYATWTSPIR

500   KYGDMINHRLLKAVIKGETATRPQDEITVQMAERRRLNRMAERDVGDWLY

550   ARFLKDKAGTDTRFAAEIVDISRGGMRVRLVDNGAIAFIPAPFLHAVRDE

600   LVCSQENGTVQIKGETVYKVTDVIDVTIAEVRMETRSIIARPVA

sp|P22106|ASNB_ECOLI Asparagine synthetase B [glutamine-hydrolyzing] OS=Escherichia coli (strain K12) OX=83333 GN=asnB PE=1 SV=3

0   MCSIFGVFDIKTDAVELRKKALELSRLMRHRGPDWSGIYASDNAILAHER

50   LSIVDVNAGAQPLYNQQKTHVLAVNGEIYNHQALRAEYGDRYQFQTGSDC

100   EVILALYQEKGPEFLDDLQGMFAFALYDSEKDAYLIGRDHLGIIPLYMGY

150   DEHGQLYVASEMKALVPVCRTIKEFPAGSYLWSQDGEIRSYYHRDWFDYD
        ****
200   AVKDNVTDKNELRQALEDSVKSHLMSDVPYGVLLSGGLDSSIISAITKKY

250   AARRVEDQERSEAWWPQLHSFAVGLPGSPDLKAAQEVANHLGTVHHEIHF

300   TVQEGLDAIRDVIYHIETYDVTTIRASTPMYLMSRKIKAMGIKMVLSGEG

350   SDEVFGGYLYFHKAPNAKELHEETVRKLLALHMYDCARANKAMSAWGVEA

400   RVPFLDKKFLDVAMRINPQDKMCGNGKMEKHILRECFEAYLPASVAWRQK

450   EQFSDGVGYSWIDTLKEVAAQQVSDQQLETARFRFPYNTPTSKEAYLYRE
                         ****
500   IFEELFPLPSAAECVPGGPSVACSSAKAIEWDEAFKKMDDPSGRAVGVHQ

550   SAYK

sp|P0ABS1|DKSA_ECOLI RNA polymerase-binding transcription factor DksA OS=Escherichia coli (strain K12) OX=83333 GN=dksA PE=1 SV=1

0   MQEGQNRKTSSLSILAIAGVEPYQEKPGEEYMNEAQLAHFRRILEAWRNQ
                                  ****
50   LRDEVDRTVTHMQDEAANFPDPVDRAAQEEEFSLELRNRDRERKLIKKIE
      ****
100   KTLKKVEDEDFGYCESCGVEIGIRRLEARPTADLCIDCKTLAEIREKQMA

150   G

sp|P46126|YFIM_ECOLI Uncharacterized protein YfiM OS=Escherichia coli (strain K12) OX=83333 GN=yfiM PE=4 SV=2

```
0     MRILFVCSLLLLSGCSHMANDSWSGQDKAQHFIASAMLSAAGNEYSQHQG
                       **********************
50    MSRDRSAMFGLMFSVSLGASKELWDSRPEGSGWSWKDLAWDVAGASTGYT

100   VWQLTRH
```

sp|P39347|INTB_ECOLI Putative protein IntB OS=Escherichia coli (strain K12) OX=83333 GN=intB PE=5 SV=1

```
                                                       *
0     MHLLVHPNGSKYWRLQYRYEGKQKMLALGVYPEITLADARVRRDEARKLL
      *****************************
50    ANGVDPGDKKKNDKVEQSKARTFKEVAIEWHGTNKKWSEDHAHRVLKSLE

100   DNLFAALGERNIAELKTRDLLAPIKAVEMSGRLEVAARLQQRTTAIMRYA

150   VQSGLIDYNPAQEMAGAVASCNRQHRPALELKRIPELLTKIDSYTGRPLT

200   RWAIELTLLIFIRSSELRFARWSEIDFEASIWTIPPEREPIPGVKHSHRG

250   SKMRTTHLVPLSTQALAILKQIKQFYGAHDLIFIGDHDSHKPMSENTVNS

300   ALRVMGYDTKVEVCGHGFRTMACSSLVESGLWSRDAVERQMSHMARNSVR

350   AAYIHKAEHLEERRLMLQWWADFLDVNRERFISPFEYAKINNPLKQ
```

sp|P31554|LPTD_ECOLI LPS-assembly protein LptD OS=Escherichia coli (strain K12) OX=83333 GN=lptD PE=1 SV=2

```
0     MKKRIPTLLATMIATALYSQQGLAADLASQCMLGVPSYDRPLVQGDTNDL

50    PVTINADHAKGDYPDDAVFTGSVDIMQGNSRLQADEVQLHQKEAPGQPEP

100   VRTVDALGNVHYDDNQVILKGPKGWANLNTKDTNVWEGDYQMVGRQGRGK

150   ADLMKQRGENRYTILDNGSFTSCLPGSDTWSVVGSEIIHDREEQVAEIWN

200   ARFKVGPVPIFYSPYLQLPVGDKRRSGFLIPNAKYTTTNYFEFYLPYYWN
                                          **************
250   IAPNMDATITPHYMHRRGNIMWENEFRYLSQAGAGLMELDYLPSDKVYED
      *************                   ******************
300   EHPNDDSSRRWLFYWNHSGVMDQVWRFNVDYTKVSDPSYFNDFDNKYGSS
      **
350   TDGYATQKFSVGYAVQNFNATVSTKQFQVFSEQNTSSYSAEPQLDVNYYQ

400   NDVGPFDTRIYGQAVHFVNTRDDMPEATRVHLEPTINLPLSNNWGSINTE

450   AKLLATHYQQTNLDWYNSRNTTKLDESVNRVMPQFKVDGKMVFERDMEML

500   APGYTQTLEPRAQYLYVPYRDQSDIYNYDSSLLQSDYSGLFRDRTYGGLD

550   RIASANQVTTGVTSRIYDDAAVERFNISVGQIYYFTESRTGDDNITWEND
```

600   DKTGSLVWAGDTYWRISERWGLRGGIQYDTRLDNVATSNSSIEYRRDEDR

650   LVQLNYRYASPEYIQATLPKYYSTAEQYKNGISQVGAVASWPIADRWSIV

700   GAYYYDTNANKQADSMLGVQYSSCCYAIRVGYERKLNGWDNDKQHAVYDN

750   AIGFNIELRGLSSNYGLGTQEMLRSNILPYQNTL

sp|P23882|FMT_ECOLI Methionyl-tRNA formyltransferase OS=Escherichia coli (strain K12) OX=83333 GN=fmt PE=1 SV=4

0   MSESLRIIFAGTPDFAARHLDALLSSGHNVVGVFTQPDRPAGRGKKLMPS

50   PVKVLAEEKGLPVFQPVSLRPQENQQLVAELQADVMVVVAYGLILPKAVL

100   EMPRLGCINVHGSLLPRWRGAAPIQRSLWAGDAETGVTIMQMDVGLDTGD
                                                ****
150   MLYKLSCPITAEDTSGTLYDKLAELGPQGLITTLKQLADGTAKPEVQDET
      ****
200   LVTYAEKLSKEEARIDWSLSAAQLERCIRAFNPWPMSWLEIEGQPVKVWK

250   ASVIDTATNAAPGTILEANKQGIQVATGDGILNLLSLQPAGKKAMSAQDL

300   LNSRREWFVPGNRLV

sp|P0C960|EMTA_ECOLI Endo-type membrane-bound lytic murein transglycosylase A OS=Escherichia coli (strain K12) OX=83333 GN=emtA PE=1 SV=1

0   MKLRWFAFLIVLLAGCSSKHDYTNPPWNAKVPVQRAMQWMPISQKAGAAW

50   GVDPQLITAIIAIESGGNPNAVSKSNAIGLMQLKASTSGRDVYRRMGWSG

100   EPTTSELKNPERNISMGAAYLNILETGPLAGIEDPKVLQYALVVSYANGA
          ****
150   GALLRTFSSDRKKAISKINDLDADEFLEHVARNHPAPQAPRYIYKLEQAL

200   DAM

sp|P37610|TAUD_ECOLI Alpha-ketoglutarate-dependent taurine dioxygenase OS=Escherichia coli (strain K12) OX=83333 GN=tauD PE=1 SV=3

0   MSERLSITPLGPYIGAQISGADLTRPLSDNQFEQLYHAVLRHQVVFLRDQ

50   AITPQQQRALAQRFGELHIHPVYPHAEGVDEIIVLDTHNDNPPDNDNWHT

100   DVTFIETPPAGAILAAKELPSTGGDTLWTSGIAAYEALSVPFRQLLSGLR
      ****
150   AEHDFRKSFPEYKYRKTEEEHQRWREAVAKNPPLLHPVVRTHPVSGKQAL

200   FVNEGFTTRIVDVSEKESEALLSFLFAHITKPEFQVRWRWQPNDIAIWDN

250   RVTQHYANADYLPQRRIMHRATILGDKPFYRAG

```
sp|P32132|TYPA_ECOLI GTP-binding protein TypA/BipA OS=Escherichia coli
(strain K12) OX=83333 GN=typA PE=1 SV=2
                                     *******************
0     MIEKLRNIAIIAHVDHGKTTLVDKLLQQSGTFDSRAETQERVMDSNDLEK
      **
50    ERGITILAKNTAIKWNDYRINIVDTPGHADFGGEVERVMSMVDSVLLVVD

100   AFDGPMPQTRFVTKKAFAYGLKPIVVINKVDRPGARPDWVVDQVFDLFVN

150   LDATDEQLDFPIVYASALNGIAGLDHEDMAEDMTPLYQAIVDHVPAPDVD

200   LDGPFQMQISQLDYNSYVGVIGIGRIKRGKVKPNQQVTIIDSEGKTRNAK

250   VGKVLGHLGLERIETDLAEAGDIVAITGLGELNISDTVCDTQNVEALPAL

300   SVDEPTVSMFFCVNTSPFCGKEGKFVTSRQILDRLNKELVHNVALRVEET

350   EDADAFRVSGRGELHLSVLIENMRREGFELAVSRPKVIFREIDGRKQEPY

400   ENVTLDVEEQHQGSVMQALGERKGDLKNMNPDGKGRVRLDYVIPSRGLIG

450   FRSEFMTMTSGTGLLYSTFSHYDDVRPGEVGQRQNGVLISNGQGKAVAFA

500   LFGLQDRGKLFLGHGAEVYEGQIIGIHSRSNDLTVNCLTGKKLTNMRASG

550   TDEAVVLVPPIRMTLEQALEFIDDDELVEVTPTSIRIRKRHLTENDRRRA

600   NRAPKDD

sp|P0AFH8|OSMY_ECOLI Osmotically-inducible protein Y OS=Escherichia coli
(strain K12) OX=83333 GN=osmY PE=1 SV=1

0     MTMTRLKISKTLLAVMLTSAVATGSAYAENNAQTTNESAGQKVDSSMNKV

50    GNFMDDSAITAKVKAALVDHDNIKSTDISVKTDQKVVTLSGFVESQAQAE

100   EAVKVAKGVEGVTSVSDKLHVRDAKEGSVKGYAGDTATTSEIKAKLLADD
                                 ***********************
150   IVPSRHVKVETTDGVVQLSGTVDSQAQSDRAESIAKAVDGVKSVKNDLKT
      *
200   K

sp|P76085|PAAK_ECOLI Phenylacetate-coenzyme A ligase OS=Escherichia coli
(strain K12) OX=83333 GN=paaK PE=1 SV=1

0     MITNTKLDPIETASVDELQALQTQRLKWTLKHAYENVPMYRRKFDAAGVH

50    PDDFRELSDLRKFPCTTKQDLRDNYPFDTFAVPMEQVVRIHASSGTTGKP

100   TVVGYTQNDIDNWANIVARSLRAAGGSPKDKIHVAYGYGLFTGGLGAHYG

150   AERLGATVIPMSGGQTEKQAQLIRDFQPDMIMVTPSYCLNLIEELERQLG
```

```
200   GDASGCSLRVGVFGAEPWTQAMRKEIERRLGITALDIYGLSEVMGPGVAM

250   ECLETTDGPTIWEDHFYPEIVNPHDGTPLADGEHGELLFTTLTKEALPVI

300   RYRTRDLTRLLPGTARTMRRMDRISGRSDDMLIIRGVNVFPSQLEEEIVK
                       *********************
350   FEHLSPHYQLEVNRRGHLDSLSVKVELKESSLTLTHEQRCQVCHQLRHRI

400   KSMVGISTDVMIVNCGSIPRSEGKACRVFDLRNIVGA

sp|Q47154|LAFU_ECOLI Putative truncated flagellar export/assembly protein
LafU OS=Escherichia coli (strain K12) OX=83333 GN=lafU PE=5 SV=1
         *************************
0     MAVPEETEKKARDVNEKTALLKKKSATELGELATSINTIARDAHMEANLE

50    MEIVPQGLRVLIKDDQNRNMFERGSAKIMPFFKTLLVELAPVFDSLDNKI

100   IITGHTDAMAYKNNIYNNWNLSGDRALSARRVLEEAGMPEDKVMQVSAMA

150   DQMLLDSKNPQSAGNRRIEIMVLTKSASDTLYQYFGQHGDKVVQPLVQKL

200   DKQQVLSQRTR

sp|P77196|YFCU_ECOLI Putative outer membrane usher protein YfcU
OS=Escherichia coli (strain K12) OX=83333 GN=yfcU PE=5 SV=3
                                   *********************
0     MPDHSLFRLRILPWCIALAMSGSYSSVWAEDDIQFDSRFLELKGDTKIDL
      *****
50    KRFSSQGYVEPGKYNLQVQLNKQPLAEEYDIYWYAGEDDVSKSYACLTPE

100   LVAQFGLKEDVAKNLQWSHDGKCLKPGQLEGVEIKADLSQSALVISLPQA

150   YLEYTWPDWDPPSRWDDGISGIIADYSITAQTRHEENGGDDSNEISGNGT
                        ***************************
200   VGVNLGPWRMRADWQTNYQHTRSNDDDDEFGGDDTQKKWEWSRYYAWRAL

250   PSLKAKLALGEDYLNSDIFDGFNYVGGSVSTDDQMLPPNLRGYAPDISGV

300   AHTTAKVTVSQMGRVIYETQVPAGPFRIQDLGDSVSGTLHIRIEEQNGQV

350   QEYDISTASMPYLTRPGQVRYKIMMGRPQEWGHHVEGGFFSGAEASWGIA

400   NGWSLYGGALGDENYQSAALGVGRDLSTFGAVAFDVTHSHTKLDKDTAYG
                                            ************
450   KGSLDGNSFRVSYSKDFDQLNSRVTFAGYRFSEENFMTMSEYLDASDSEM
      *********
500   VRTGNDKEMYTATYNQNFRDAGVSVYLNYTRHTYWDREEQTNYNIMLSHY

550   FNMGSIRNMSVSLTGYRYEYDNRADKGMYISLSMPWGDNSTVSYNGNYGS

600   GTDSSQVGYFSRVDDATHYQLNIGTSDKHTSVDGYYSHDGSLAQVDLSAN

650   YHEGQYTSAGLSLQGGATLTTHGGALHRTQNMGGTRLLIDADGVADVPVE
```

```
700   GNGAAVYTNMFGKAVVSDVNNYYRNQAYIDLNKLPENAEATQSVVQATLT

750   EGAIGYRKFAVISGQKAMAVLRLQDGSHPPFGAEVKNDNEQTVGLVDDDG

800   SVYLAGVKPGEHMSVFWSGVAHCDINLPDPLPADLFNGLLLPCQHKGNVA

850   PVVPDDIKPVIQEQTQQVTPTDPPVSVSANQ
```

sp|P0C0L9|ISCX_ECOLI Protein IscX OS=Escherichia coli (strain K12) OX=83333 GN=iscX PE=1 SV=1

```
                                         ***************
0     MGLKWTDSREIGEALYDAYPDLDPKTVRFTDMHQWICDLEDFDDDPQASN
      *******
50    EKILEAILLVWLDEAE
```

sp|P32693|YJBL_ECOLI Uncharacterized protein YjbL OS=Escherichia coli (strain K12) OX=83333 GN=yjbL PE=4 SV=1

```
                          *********************
0     MLKIIPGATGYFNKTLNSNQFDNEDAIKDKLDNRGSIKGKLNNIYGKSID

50    YAALRHRDIIIAKIDLFIQRITHNLWHARKKMCF
```

sp|P32134|YIHM_ECOLI Uncharacterized protein YihM OS=Escherichia coli (strain K12) OX=83333 GN=yihM PE=4 SV=1

```
0     MVTINNARKILQRVDTLPLYLHAYAFHLNMRLERVLPADLLDIASENNLR

50    GVKIHVLDGERFSLGNMDDKELSAFGDKARRLNLDIHIETSASDKASIDE
                                                      **
100   AVAIALKTGASSVRFYPRYEGNLRDVLSIIANDIAYVRETYQDSGLTFTI
      *****************************
150   EQHEDLKSHELVSLVKESEMESLSLLFDFANMINANEHPIDALKTMAPHI

200   TQVHIKDALIVKEPGGLGHKACISGQGDMPFKALLTHLICLGDDEPQVTA

250   YGLEEEVDYYAPAFRFEDEDDNPWIPYRQMSETPLPENHLLDARLRKEKE

300   DAINQINHVRNVLQQIKQEANHLLNH
```

sp|P31828|PQQL_ECOLI Probable zinc protease PqqL OS=Escherichia coli (strain K12) OX=83333 GN=pqqL PE=3 SV=2

```
0     MEIIMRNLCFLLTLVATLLLPGRLIAAALPQDEKLITGQLDNGLRYMIYP

50    HAHPKDQVNLWLQIHTGSLQEEDNELGVAHFVEHMMFNGTKTWPGNKVIE

100   TFESMGLRFGRDVNAYTSYDETVYQVSLPTTQKQNLQQVMAIFSEWSNAA

150   TFEKLEVDAERGVITEEWRAHQDAKWRTSQARRPFLLANTRNLDREPIGL

200   MDTVATVTPAQLRQFYQRWYQPNNMTFIVVGDIDSKEALALIKDNLSKLP

250   ANKAAENRVWPTKAENHLRFNIINDKENRVNGIALYYRLPMVQVNDEQSF
```

300   IEQAEWSMLVQLFNQRLQERIQSGELKTISGGTARSVKIAPDYQSLFFRV

350   NARDDNMQDAANALMAELATIDQHGFSAEELDDVKSTRLTWLKNAVDQQA

400   ERDLRMLTSRLASSSLNNTPFLSPEETYQLSKRLWQQITVQSLAEKWQQL

450   RKNQDAFWEQMVNNEVAAKKALSPAAILALEKEYANKKLAAYVFPGRNLS

500   LTVDADPQAEISSKETLAENLTSLTLSNGARVILAKSAGEEQKLQIIAVS
                         ****
550   NKGDLSFPAQQKSLIALANKAVSGSGVGELSSSSLKRWSAENSVTMSSKV
      **
600   SGMNTLLSVSARTNNPEPGFQLINQRITHSTINDNIWASLQNAQIQALKT

650   LDQRPAEKFAQQMYETRYADDRTKLLQENQIAQFTAADALAADRQLFSSP

700   ADITFVIVGNVAEDKLVALITRYLGSIKHSDSPLAAGKPLTRATDNASVT

750   VKEQNEPVAQVSQWKRYDSRTPVNLPTRMALDAFNVALAKDLRVNIREQA

800   SGAYSVSSRLSVDPQAKDISHLLAFTCQPERHDELLTLANEVMVKRLAKG

850   ISEQELNEYQQNVQRSLDIQQRSVQQLANTIVNSLIQYDDPAAWTEQEQL

900   LKQMTVENVNTAVKQYLSHPVNTYTGVLLPK

sp|P19769|INSK_ECOLI Putative transposase InsK for insertion sequence element IS150 OS=Escherichia coli (strain K12) OX=83333 GN=insK PE=2 SV=2
                                   ****
0   MKVLNELRQFYPLDELLRAAEIPRSTFYYHLKALSKPDKYADVKKRISEI
      ****
50   YHENRGRYGYRRVTLSLHREGKQINHKAVQRLMGTLSLKAAIKVKRYRSY

100   RGEVGQTAPNVLQRDFKATRPNEKWVTDVTEFAVNGRKLYLSPVIDLFNN

150   EVISYSLSERPVMNMVENMLDQAFKKLNPHEHPVLHSDQGWQYRMRRYQN
                                  ****
200   ILKEHGIKQSMSRKGNCLDNAVVECFFGTLKSECFYLDEFSNISELKDAV

250   TEYIEYYNSRRISLKLKGLTPIEYRNQTYMPRV

sp|P69451|LCFA_ECOLI Long-chain-fatty-acid--CoA ligase OS=Escherichia coli (strain K12) OX=83333 GN=fadD PE=1 SV=1

0   MKKVWLNRYPADVPTEINPDRYQSLVDMFEQSVARYADQPAFVNMGEVMT

50   FRKLEERSRAFAAYLQQGLGLKKGDRVALMMPNLLQYPVALFGILRAGMI

100   VVNVNPLYTPRELEHQLNDSGASAIVIVSNFAHTLEKVVDKTAVQHVILT

150   RMGDQLSTAKGTVVNFVVKYIKRLVPKYHLPDAISFRSALHNGYRMQYVK

200   PELVPEDLAFLQYTGGTTGVAKGAMLTHRNMLANLEQVNATYGPLLHPGK

```
250   ELVVTALPLYHIFALTINCLLFIELGGQNLLITNPRDIPGLVKELAKYPF

300   TAITGVNTLFNALLNNKEFQQLDFSSLHLSAGGGMPVQQVVAERWVKLTG

350   QYLLEGYGLTECAPLVSVNPYDIDYHSGSIGLPVPSTEAKLVDDDDNEVP

400   PGQPGELCVKGPQVMLGYWQRPDATDEIIKNGWLHTGDIAVMDEEGFLRI
                                                     *********
450   VDRKKDMILVSGFNVYPNEIEDVVMQHPGVQEVAAVGVPSGSSGEAVKIF
      ************
500   VVKKDPSLTEESLVTFCRRQLTGYKVPKLVEFRDELPKSNVGKILRRELR

550   DEARGKVDNKA

sp|P56614|YMDF_ECOLI Uncharacterized protein YmdF OS=Escherichia coli
(strain K12) OX=83333 GN=ymdF PE=3 SV=1
                                         ***************
0     MANHRGGSGNFAEDRERASEAGKKGGQHSGGNFKNDPQRASEAGKKGGKS
      *******
50    SHGKSDN

sp|P39368|YJHQ_ECOLI Uncharacterized N-acetyltransferase YjhQ
OS=Escherichia coli (strain K12) OX=83333 GN=yjhQ PE=3 SV=1
                 *********************
0     MTVHHFTFHITDKSDASDIREVETRAFGFSKEADLVASLLEDESARPALS

50    LLARYEGKAVGHILFTRATFKGEMDSPLMHILAPLAVIPEYQGMGVGGRL

100   IRTGIEHLRLMGCQTVFVLGHATYYPRHGFEPCAGDKGYPAPYPIPEEHK

150   ACWMMQSLTAQPMTLTGHIRCADPDETGALT

sp|P75750|YBGQ_ECOLI Uncharacterized outer membrane usher protein YbgQ
OS=Escherichia coli (strain K12) OX=83333 GN=ybgQ PE=3 SV=2

0     MNIYRLSFVSCLVMAMPCAMAVEFNLNVLDKSMRDRIDISLLKEKGVIAP

50    GEYFVSVAVNNNKISNGQKINWQKKGDKTIPCINDSLVDKFGLKPDIRQS

100   LPQIDRCIDFSSRPEMLFNFDQANQQLNISIPQAWLAWHSENWAPPSTWK

150   EGVAGVLMDYNLFASSYRPQDGSSSTNLNAYGTAGINAGAWRLRSDYQLN

200   KTDSEDNHDQSGGISRTYLFRPLPQLGSKLTLGETDFSSNIFDGFSYTGA

250   ALASDDRMLPWELRGYAPQISGIAQTNATVTISQSGRVIYQKKVPPGPFI

300   IDDLNQSVQGTLDVKVTEEDGRVNNFQVSAASTPFLTRQGQVRYKLAAGQ

350   PRPSMSHQTENETFFSNEVSWGMLSNTSLYGGLLISDDDYHSAAMGIGQN

400   MLWLGALSFDVTWASSHFDTQQDERGLSYRFNYSKQVDATNSTISLAAYR
                  ********************
450   FSDRHFHSYANYLDHKYNDSDAQDEKQTISLSVGQPITPLNLNLYANLLH
```

```
500   QTWWNADASTTANITAGFNVDIGDWRDISISTSFNTTHYEDKDRDNQIYL

550   SISLPFGNGGRVGYDMQNSSHSTIHRMSWNDTLDERNSWGMSAGLQSDRP

600   DNGAQVSGNYQHLSSAGEWDISGTYAASDYSSVSSSWSGSFTATQYGAAF

650   HRRSSTNEPRLMVSTDGVADIPVQGNLDYTNHFGIAVVPLISSYQPSTVA

700   VNMNDLPDGVTVAENVIKETWIEGAIGYKSLASRSGKDVNVIIRNASGQF

750   PPLGADIRQDDSGISVGMVGEEGHAWLSGVAENQLFTVVWGEQSCIIHLP

800   ERLEDTTKRLILPCH

sp|P0AB18|TUSE_ECOLI Sulfurtransferase TusE OS=Escherichia coli (strain
K12) OX=83333 GN=tusE PE=1 SV=1
       **************************
0     MLIFEGKEIETDTEGYLKESSQWSEPLAVVIAENEGISLSPEHWEVVRFV

50    RDFYLEFNTSPAIRMLVKAMANKFGEEKGNSRYLYRLFPKGPAKQATKIA

100   GLPKPVKCI

sp|P15070|FLIN_ECOLI Flagellar motor switch protein FliN OS=Escherichia
coli (strain K12) OX=83333 GN=fliN PE=1 SV=1
                  ****************************
0     MSDMNNPADDNNGAMDDLWAEALSEQKSTSSKSAAETVFQQFGGGDVSGT

50    LQDIDLIMDIPVKLTVELGRTRMTIKELLRLTQGSVVALDGLAGEPLDIL

100   INGYLIAQGEVVVVADKYGVRITDIITPSERMRRLSR

sp|P0A7K2|RL7_ECOLI 50S ribosomal protein L7/L12 OS=Escherichia coli
(strain K12) OX=83333 GN=rplL PE=1 SV=2

0     MSITKDQIIEAVAAMSVMDVVELISAMEEKFGVSAAAAVAVAAGPVEAAE
                                     *******************
50    EKTEFDVILKAAGANKVAVIKAVRGATGLGLKEAKDLVESAPAALKEGVS
      *********************
100   KDDAEALKKALEEAGAEVEVK

sp|P45463|TTDR_ECOLI HTH-type transcriptional activator TtdR
OS=Escherichia coli (strain K12) OX=83333 GN=ttdR PE=3 SV=2

0     MLNSWPLAKDLQVLVEIVHSGSFSAAAATLGQTPAFVTKRIQILENTLAT

50    TLLNRSARGVALTESGQRCYEHALEILTQYQRLVDDVTQIKTRPEGMIRI

100   GCSFGFGRSHIAPAITELMRNYPELQVHFELFDRQIDLVQDNIDLDIRIN

150   DEIPDYYIAHLLTKNKRILCAAPEYLQKYPQPQSLQELSRHDCLVTKERD
               ***********************
200   MTHGIWELGNGQEKKSVKVSGHLSSNSGEIVLQWALEGKGIMLRSEWDVL
```

```
250   PFLESGKLVQVLPEYAQSANIWAVYREPLYRSMKLRVCVEFLAAWCQQRL

300   GKPDEGYQVM

sp|P77173|ZIPA_ECOLI Cell division protein ZipA OS=Escherichia coli
(strain K12) OX=83333 GN=zipA PE=1 SV=3
                                 ***********************
0     MMQDLRLILIIVGAIAIIALLVHGFWTSRKERSSMFRDRPLKRMKSKRDD
      ****************
50    DSYDEDVEDDEGVGEVRVHRVNHAPANAQEHEAARPSPQHQYQPPYASAQ

100   PRQPVQQPPEAQVPPQHAPHPAQPVQQPAYQPQPEQPLQQPVSPQVAPAP

150   QPVHSAPQPAQQAFQPAEPVAAPQPEPVAEPAPVMDKPKRKEAVIIMNVA

200   AHHGSELNGELLLNSIQQAGFIFGDMNIYHRHLSPDGSGPALFSLANMVK

250   PGTFDPEMKDFTTPGVTIFMQVPSYGDELQNFKLMLQSAQHIADEVGGVV

300   LDDQRRMMTPQKLREYQDIIREVKDANA

sp|P76394|YEGJ_ECOLI Uncharacterized protein YegJ OS=Escherichia coli
(strain K12) OX=83333 GN=yegJ PE=4 SV=1
                                             ***********
0     MKKILLMLSLLFFTTAGFSEVSDTLVTGGYDKQAMSDAIKHARKETDKFI
      *********
50    EVMNKKDADTFAVKAPITDHGRTEHFWLTDVTYSNGMFIGVISNDPGIVT
           *********************
100   NVEYGQEWKIKKEDISDWMYTRGDKIYGGYTIDPLLVTYPKEEADELRAK

150   LVR

sp|P69910|DCEB_ECOLI Glutamate decarboxylase beta OS=Escherichia coli
(strain K12) OX=83333 GN=gadB PE=1 SV=1
                ********************
0     MDKKQVTDLRSELLDSRFGAKSISTIAESKRFPLHEMRDDVAFQIINDEL

50    YLDGNARQNLATFCQTWDDENVHKLMDLSINKNWIDKEEYPQSAAIDLRC

100   VNMVADLWHAPAPKNGQAVGTNTIGSSEACMLGGMAMKWRWRKRMEAAGK

150   PTDKPNLVCGPVQICWHKFARYWDVELREIPMRPGQLFMDPKRMIEACDE

200   NTIGVVPTFGVTYTGNYEFPQPLHDALDKFQADTGIDIDMHIDAASGGFL

250   APFVAPDIVWDFRLPRVKSISASGHKFGLAPLGCGWVIWRDEEALPQELV

300   FNVDYLGGQIGTFAINFSRPAGQVIAQYYEFLRLGREGYTKVQNASYQVA

350   AYLADEIAKLGPYEFICTGRPDEGIPAVCFKLKDGEDPGYTLYDLSERLR

400   LRGWQVPAFTLGGEATDIVVMRIMCRRGFEMDFAELLLEDYKASLKYLSD
```

450   HPKLQGIAQQNSFKHT

sp|P64467|CNU_ECOLI OriC-binding nucleoid-associated protein OS=Escherichia coli (strain K12) OX=83333 GN=cnu PE=1 SV=1

```
         *******************
0     MTVQDYLLKFRKISSLESLEKLYDHLNYTLTDDQELINMYRAADHRRAEL

50    VSGGRLFDLGQVPKSVWHYVQ
```
