# Supplementary material for: Identification of polyphosphate-binding proteins in Escherichia coli uncovers targets involved in translation control and ribosome biogenesis
Source: mBio. 2025 Jul 7;16(8):e00500-25. doi: 10.1128/mbio.00500-25 (PMC12345225; doi:10.1128/mbio.00500-25)
Supplement: Supplemental Figures — Figures S1–S7. [file mbio.00500-25-s0002.pdf]

SUPPLEMENTAL FIGURES 1-6:

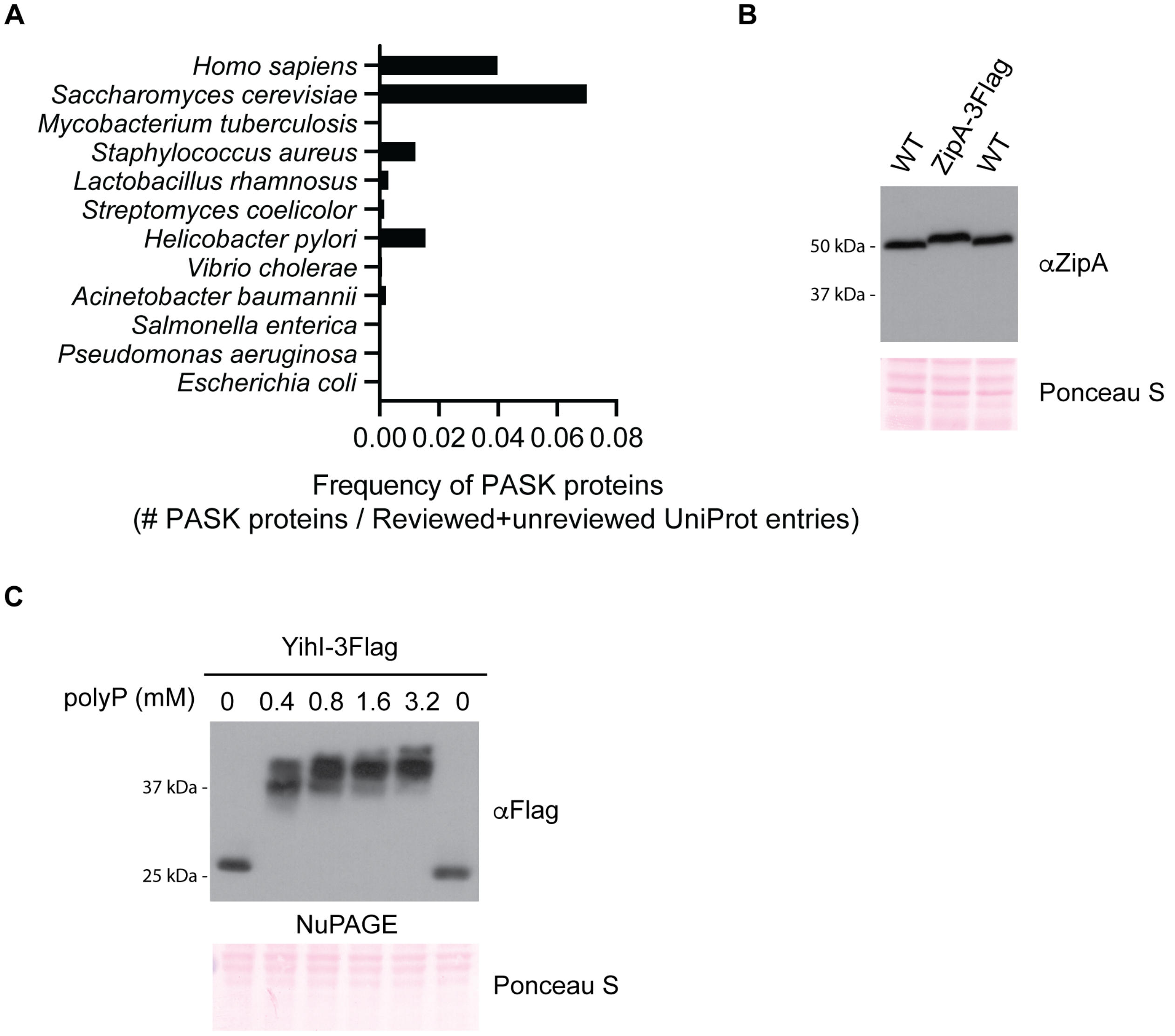

Supplemental Figure 1: The PASK is not a good indicator of polyP-protein binding in bacteria. **(A)** PASK frequency using reviewed+unreviewed proteomes. The number of proteins containing 1 or more PASK motifs (75% D/E/S/K content with at least one lysine within a 20 amino acid window) from reviewed and unreviewed proteomes of the indicated species were normalized by the total number of reviewed+unreviewed UniProt entries for each species. Underlying data for S1A can be found in **Source Data 1** **(B)** Anti-ZipA antibody validation blot. Whole cell extracts from wild-type and ZipA-3Flag tagged strains were resolved on 12% SDS-PAGE, transferred to a PVDF membrane and probed using an anti-ZipA antibody. Ponceau S was used to show equal protein loading and that samples migrated equally. A deletion mutation of Δ*zipA* could not be used because *zipA* is essential. ZipA-3Flag displayed a shift in migration as expected, confirming the antibody recognizes ZipA. **(C)** YihI-polyP binding shifts on NuPAGE are dependent on polyP concentration. Whole cell extract from a YihI-3Flag tagged

strain was used for an *in vitro* polyP binding assay in the presence of increasing concentrations of polyP. Samples were resolved using NuPAGE, transferred to a PVDF membrane and probed using an anti-Flag antibody. Ponceau S was used to show that samples migrated equally. Images are representative of results from ≥3 experiments.

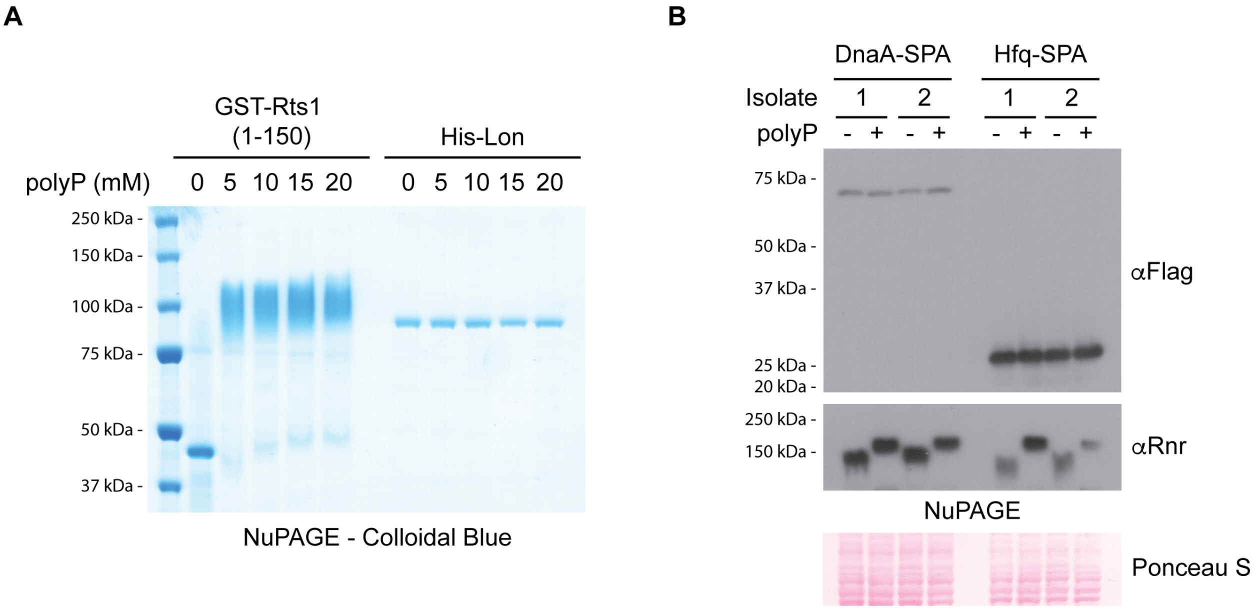

Supplemental Figure 2: NuPAGE electrophoresis is not effective for detecting polyP binding to Lon, DnaA and Hfq. (**A**) Lon does not display the characteristic polyP binding shift on NuPAGE gels. Increasing concentrations of polyP (p700) were incubated with 0.032 mg of purified Rts1 (positive control)(20) or Lon protease. Samples were resolved using NuPAGE and the gel was stained using Colloidal Blue to visualize the proteins. (**B**) DnaA-SPA and Hfq-SPA do not shift by NuPAGE electrophoresis in the presence of polyP. Both, SPA-tagged proteins were screened *in vitro* for polyP binding as described in Figure 1C for YihI-SPA. Samples were resolved using NuPAGE, transferred to a PVDF membrane and probed using an anti-Flag antibody. After visualization of the anti-Flag signal, the same membrane was reprobed with anti-Rnr which served as a positive control for the assay. Images are representative of results from ≥3 experiments.

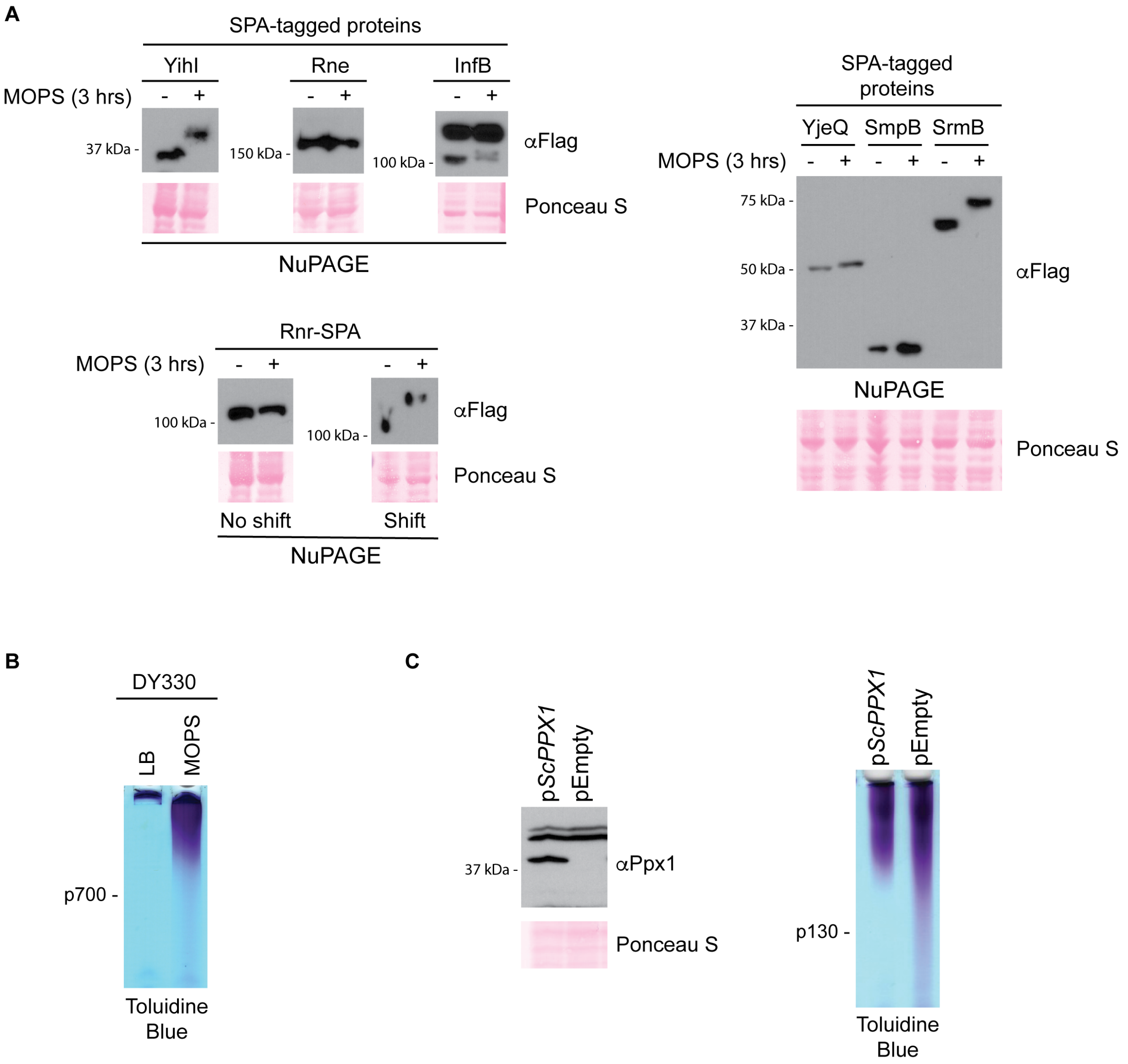

Supplemental Figure 3: PolyP-binding proteins may have limited access to endogenous polyP that accumulates in response to stress. (A) YihI, SmpB and Rnr display NuPAGE shifts in the presence of endogenous polyP. Whole cell extract from SPA-tagged strains that were grown in LB media (- MOPS) or exposed to nutrient downshift (+ MOPS) for 3 hours were resolved using NuPAGE, transferred to a PVDF membrane and probed using an anti-Flag antibody which detects the SPA tag. Ponceau S was used to show that samples migrated equally. Images are representative of results from ≥3 experiments. **(B)** *E. coli* makes long chain polyP after 3 hours in MOPS media. PolyP that was extracted from cells grown in LB media or MOPS for 3 hours (as described for S3A) was resolved using a TBE-urea acrylamide gel and stained using toluidine blue. The gel shows that endogenous polyP is longer than the p700 standard. Images are representative of results from ≥3 experiments. **(C)** Endogenous polyP is not fully degraded by ectopic expression of *S. cerevisiae* exopolyphosphatase Ppx1 (*Sc*Ppx1). Western

blotting (left) and polyP extractions (right) of *E. coli* with p*ScPPX1* or the empty vector. Cells were grown in LB media in the presence of 0.5% arabinose (the inducer) before undergoing a nutrient downshift to MOPS media for 3 hours. PolyP and whole cell extracts were resolved using a TBE-urea acrylamide gel or 12% SDS-PAGE, respectively. The polyP gel was stained using toluidine blue and Ppx1 expression was detected using an anti-Ppx1 antibody. Images are representative of ≥3 experiments.

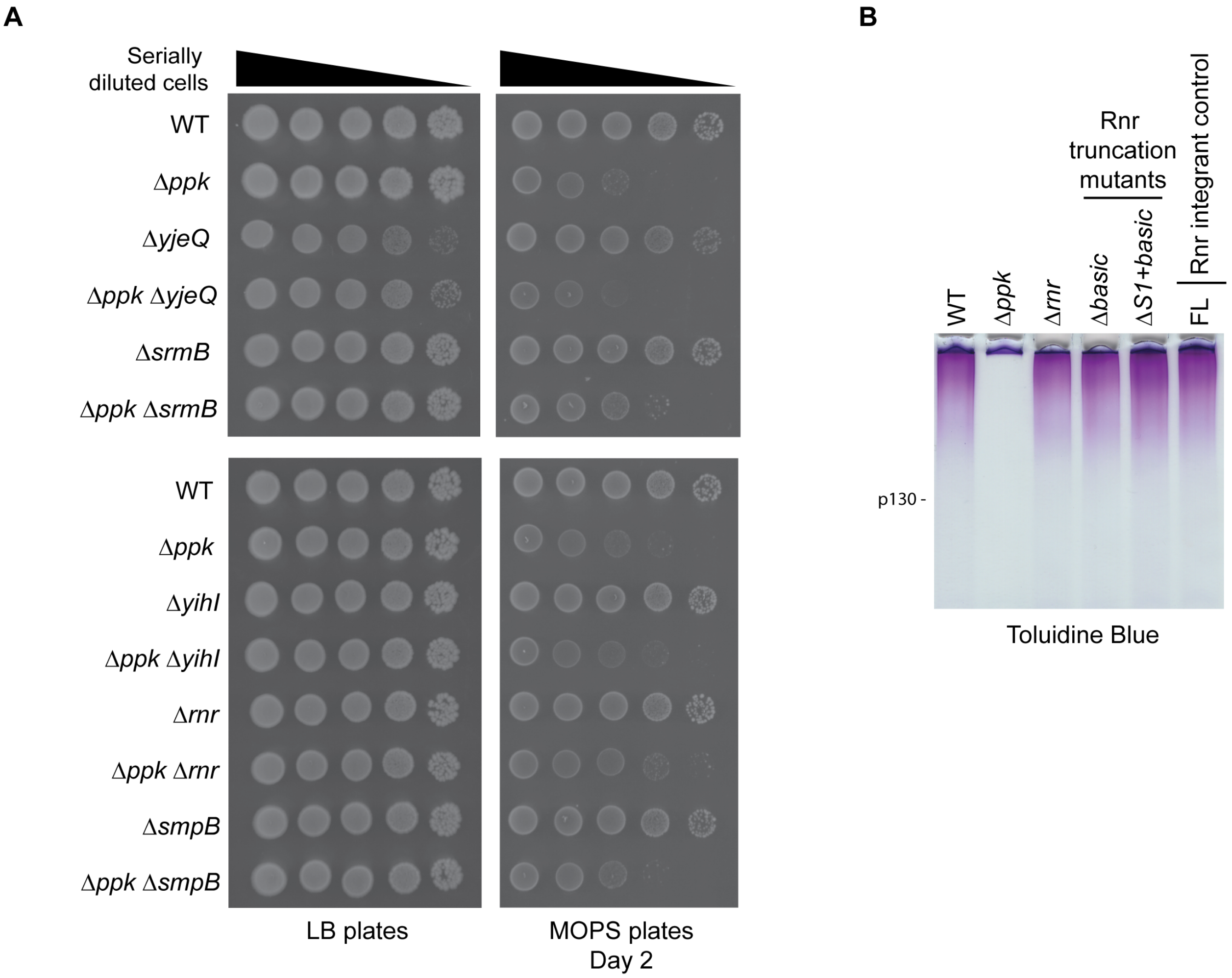

Supplemental Figure 4: Testing if loss of polyP-binding proteins impacts Δ*ppk* mutant growth. (A) Spot test of *E. coli* mutated for genes encoding the polyP-binding proteins. The indicated strains were serially diluted and spotted onto LB or MOPS plates and incubated at 37°C as indicated. Images are representative of results from ≥3 experiments. **(B)** Mutation of *rnr* does not impact polyP accumulation in an otherwise wild-type background. PolyP extracted from cells grown in LB media and exposed to nutrient down shift for 3 hours was resolved using a TBE-urea acrylamide gel and stained using toluidine blue. The migration of a standard of modal length p130 is indicated. FL represents the wild-type Rnr protein expressed in a background that is isogenic to the truncated and mutated strains (see methods *Bacterial strains* section for details on how these strains were made). Images are representative of results from ≥3 experiments.

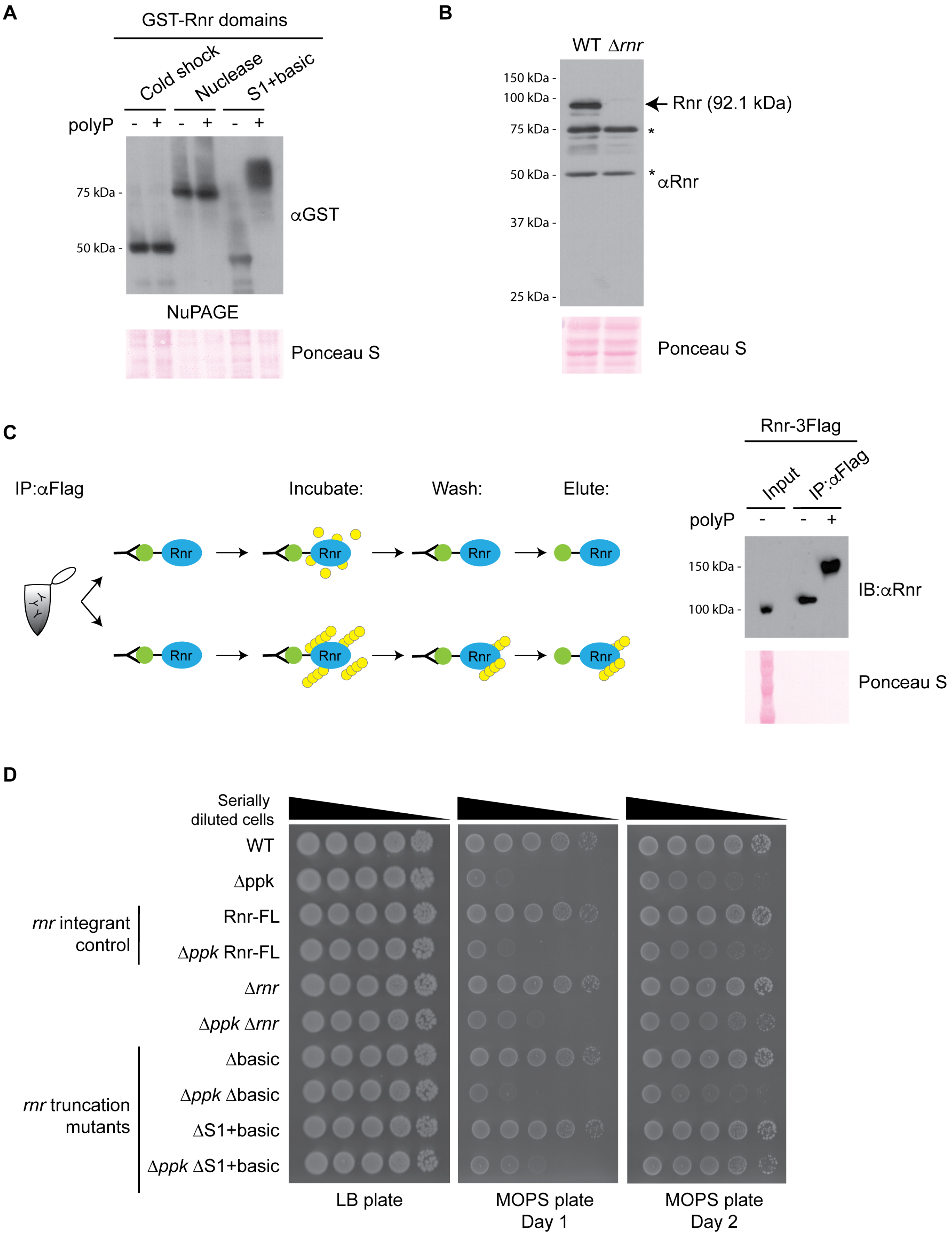
A
GST-Rnr domains
Cold shock
Nuclease
S1+basic
polyP
- + - + - +
75 kDa -
50 kDa -
αGST
NuPAGE
Ponceau S
B
WT Δrnr
150 kDa -
100 kDa -
75 kDa -
50 kDa -
37 kDa -
25 kDa -
Rnr (92.1 kDa)
*
*
αRnr
Ponceau S
C
IP:αFlag
Incubate:
Wash:
Elute:
Rnr
Rnr-3Flag
Input
IP:αFlag
polyP
- - +
150 kDa -
100 kDa -
IB:αRnr
Ponceau S
D
Serially diluted cells
WT
Δppk
rnr integrant control
Rnr-FL
Δppk Rnr-FL
Δrnr
Δppk Δrnr
Δbasic
rnr truncation mutants
Δppk Δbasic
ΔS1+basic
Δppk ΔS1+basic
LB plate
MOPS plate Day 1
MOPS plate Day 2

Supplemental Figure 5: A complex interplay between PPK and the Rnr polyP-binding domain. (A) PolyP binds to the S1 and basic domain of Rnr. Whole cell extract from cells expressing GST-tagged Rnr domains were used to conduct *in vitro* polyP binding assays, resolved using NuPAGE, transferred to a PVDF membrane and probed using an anti-GST antibody. Ponceau S was used to show that samples migrated equally. Images are representative of results from ≥3 experiments. **(B)** Anti-Rnr antibody validation blot. An arrow is used to show the band corresponding to Rnr while asterisks (*) indicate background bands. Whole cell extract from WT and Δ*rnr* strains was resolved on 12% SDS-PAGE, transferred to a PVDF membrane and probed using the anti-Rnr antibody. Ponceau S was used to show equal protein loading. **(C)** PolyP binds the native form of Rnr. Schematic (left): Rnr-3Flag was immunoprecipitated (IP) from whole cell extract under non-denaturing conditions using anti-Flag beads and then incubated with polyP (p700). Excess polyP was then washed away before eluting the protein. IP'ed proteins were resolved using NuPAGE, transferred to a PVDF membrane and probed using an anti-Rnr antibody. Images are representative of results from ≥3 experiments. **(D)** Loss of the Rnr S1 and basic domains rescues *ppk* mutant growth phenotypes comparable to Δ*ppk* Δ*rnr* double mutants. FL represents the wild-type Rnr protein expressed in a background that is isogenic to the truncated and mutated strains (see methods *Bacterial strains* section for details on how these strains were made). The indicated strains were serially diluted and spotted on LB or MOPS plates and incubated at 37°C as indicated. Images are representative of results from ≥3 experiments.

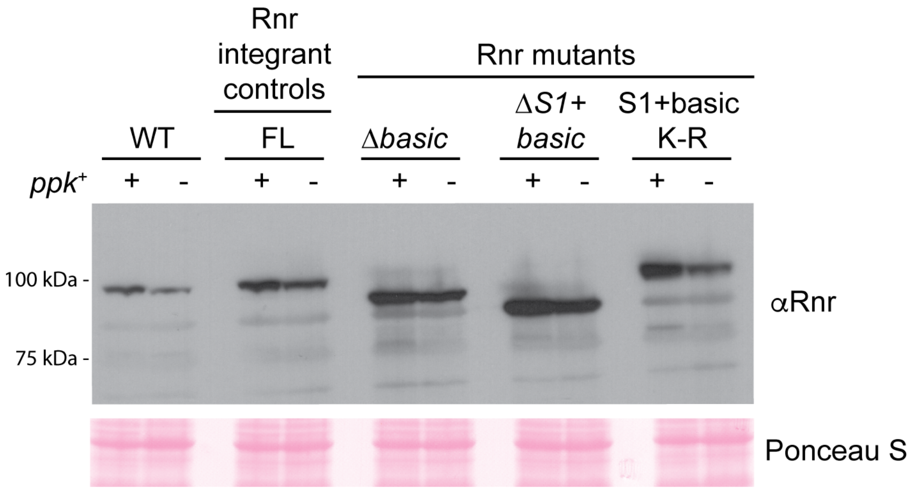

Supplemental Figure 6. Expression of wild-type and mutant Rnr is downregulated in Δ*ppk* mutants compared to wild-type cells during growth in MOPS. FL Rnr is as described in Figure 5. Whole cell extract from wild-type and mutant Rnr strains that were grown in LB media then exposed to nutrient down shift for 3 hours were resolved using 10% SDS-PAGE, transferred to a PVDF membrane and probed using an anti-Rnr antibody. Ponceau S was used to show equal loading. Images are representative of results from ≥3 experiments.

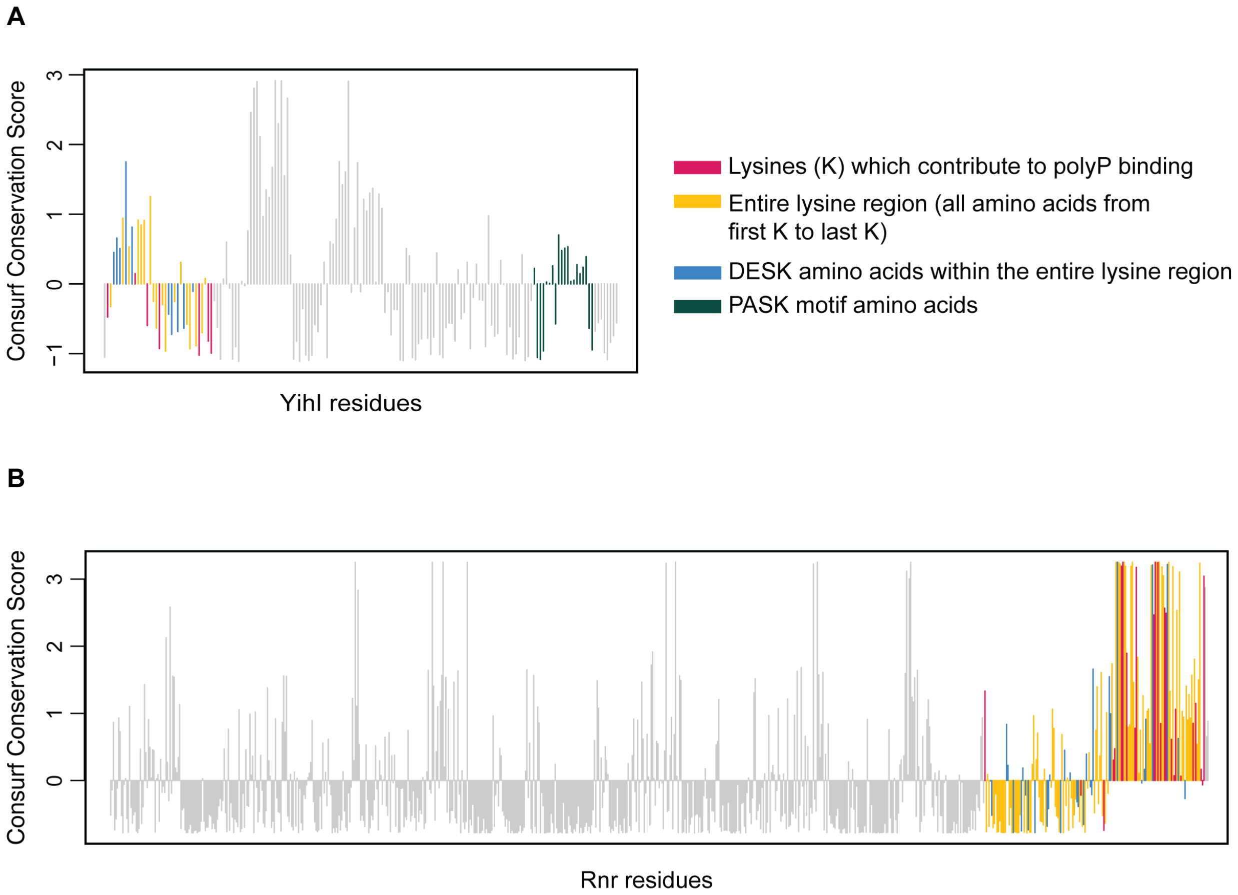

Supplemental Figure 7. Conservation analysis of polyP-binding regions of YihI and Rnr. (A-B) The evolutionary rate of the indicated residues of YihI (A) and Rnr (B) were evaluated across 150 homologs using ConSurf. The residues highlighted in colour represent those corresponding to the C-terminal PASK and N-terminal PASK-like sequences of YihI, and S1+basic domain of Rnr. ConSurf conservation scores are used as a proxy for each residue's evolutionary rate. A positive evolutionary rate indicates more variability while a smaller or negative evolutionary rate points to more conservation. Residue scores can be found in **Source Data 6**.
